# Visible-light photoredox synthesis of unnatural chiral α-amino acids

**DOI:** 10.1038/srep26161

**Published:** 2016-05-17

**Authors:** Min Jiang, Yunhe Jin, Haijun Yang, Hua Fu

**Affiliations:** 1 Key Laboratory of Bioorganic Phosphorus Chemistry and Chemical Biology (Ministry of Education), Department of Chemistry, Tsinghua University, Beijing 100084, P. R. China

## Abstract

Unnatural chiral α-amino acids are widely used in fields of organic chemistry, biochemistry and medicinal chemistry, and their synthesis has attracted extensive attention. Although the asymmetric synthesis provides some efficient protocols, noble and elaborate catalysts, ligands and additives are usually required which leads to high cost. Distinctly, it is attractive to make unnatural chiral α-amino acids from readily available natural α-amino acids through keeping of the existing chiral α-carbon. However, it is a great challenge to construct them under mild conditions. In this paper, 83 unnatural chiral α-amino acids were prepared at room temperature under visible-light assistance. The protocol uses two readily available genetically coded proteinogenic amino acids, L-aspartic acid and glutamic acid derivatives as the chiral sources and radical precursors, olefins, alkynyl and alkenyl sulfones, and 2-isocyanobiphenyl as the radical acceptors, and various unnatural chiral α-amino acids were prepared in good to excellent yields. The simple protocol, mild conditions, fast reactions, and high efficiency make the method an important strategy for synthesis of diverse unnatural chiral α-amino acids.

Unnatural chiral α-amino acids (α-AAs) are important building blocks in synthesis of biological and pharmaceutical peptides, peptidomimetics and complex molecules. Particularly, the peptides incorporating unnatural α-AAs can resist hydrolysis of proteinases and usually exhibit interesting pharmacological activity^1^. In addition, they are also used as the chiral catalysts and ligands^2^. Therefore, the chemical synthesis of these valuable compounds has received tremendous interests^3^. Traditionally, acquirement of optically active unnatural α-AAs is through chemical and enzymatic resolutions of the corresponding racemates. As development of asymmetric synthesis, various useful methods for synthesis of unnatural α-AAs have been developed^4^, and the common strategies include the asymmetric Strecker reaction^5^,^6^, the enantioselective hydrogenation of dehydroamino acid precursors^7^, and the asymmetric alkylation of glycine derivatives employing chiral auxiliaries^8^ or chiral phase-transfer catalysts^9^,^10^. Considering the widespread availability of natural α-AAs, functionalization on their side chains through keeping the existing chirality is a very attractive strategy^11^. Recently, palladium-catalyzed direct C-H functionalization on their side chains of natural α-AAs to access the corresponding unnatural counterparts has attracted much attention. The Corey^12^, Daugulis^13^ and other groups^14^,^15^ have elegantly demonstrated palladium-catalyzed auxiliary-directed functionalization of the *β*-C(sp^3^)-H bonds of natural chiral α-amino acid derivatives using the 8-aminoquinoline *N,N*-bidentate directing group (Fig. 1a). Inspired by the excellent results, palladium-catalyzed directed γ-C(sp^3^)-H functionalization of natural α-amino acid derivatives has been also developed (Fig. 1b)^16^. Very recently, Yu and co-workers have described palladium-catalyzed ligand-controlled C(sp^3^)-H arylations in synthesis of unnatural chiral α-AAs (Fig. 1c)^17^. However, several drawbacks are remained such as harsh reaction conditions and long reaction time, and limited substrate scope and products. The keen demand for unnatural chiral α-AAs has prompted chemists to explore more efficient and general alternatives. Recently, visible light photoredox catalysis has recently attracted much attention, and it has emerged as a powerful activation strategy in new chemical transformations^18^,^19^,^20^,^21^,^22^,^23^. Furthermore, some decarboxylative couplings to the formation C-C bonds have been developed^24^,^25^,^26^,^27^,^28^,^29^,^30^,^31^. To the best of our knowledge, there is no report on synthesis of unnatural chiral α-AAs using natural α-AAs as the precursors under visible light photoredox catalysis thus far. Herein, we first disclose visible-light photoredox synthesis of unnatural optically pure α-AAs based on the derivatives of two genetically coded proteinogenic amino acids, L-aspartic acid and glutamic acid, at room temperature (Fig. 1d), in which the carboxyls on the side chains of *N*-Bis(Boc)-Asp-OMe and *N*-Bis(Boc)-Glu-OMe are activated with *N*-hydroxyphthalimide to get the corresponding active esters (**1**), *N*-Bis(Boc)-Asp(OPht)-OMe and *N*-Bis(Boc)-Glu(OPht)-OMe, and treatment of **1** with olefins (**2**), or alkynyl sulfones (**4**) provides the target products **3** and **5** under visible-light photoredox catalysis.

## Results and Discussion

### Synthesis of compounds 3a-bm

**Figure 2:**
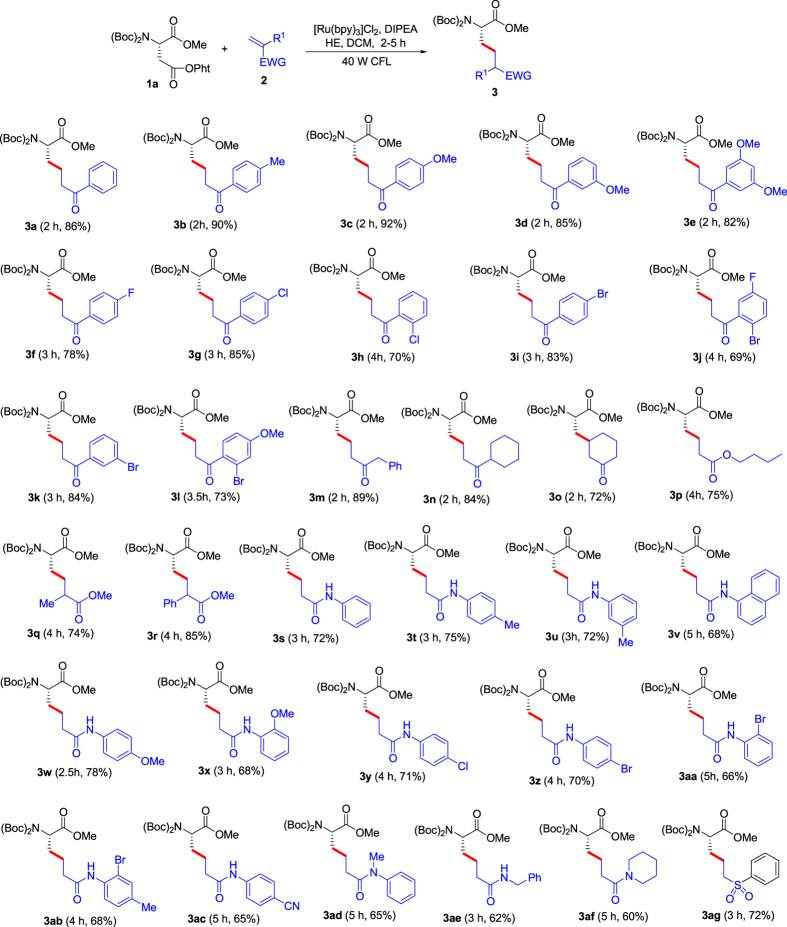
Substrate scope for decarboxylative couplings of *N*-Bis(Boc)-Asp(OPht)-OMe (**1a**) with olefins (2). *Reaction conditions: under vacuum and irradiation of visible light, *N*-Bis(Boc)-Asp(OPht)-OMe (**1a**) (0.10 mmol using ketones or esters as the partners; 0.15 mmol using amides as the partners), olefin (**2**) (0.15 mmol for ketones and esters; 0.10 mmol for amides), [Ru(bpy)_3_]Cl_2_ (1.0 μmol), diisopropylethylamine (DIPEA) (0.25 mmol), Hantzsch ester (HE) (0.15 mmol), DCM (1.0 mL), temperature (rt, ~25 °C), time (1–5 h) in a sealed Schlenk tube. ^†^Isolated yield.

**Figure 3:**
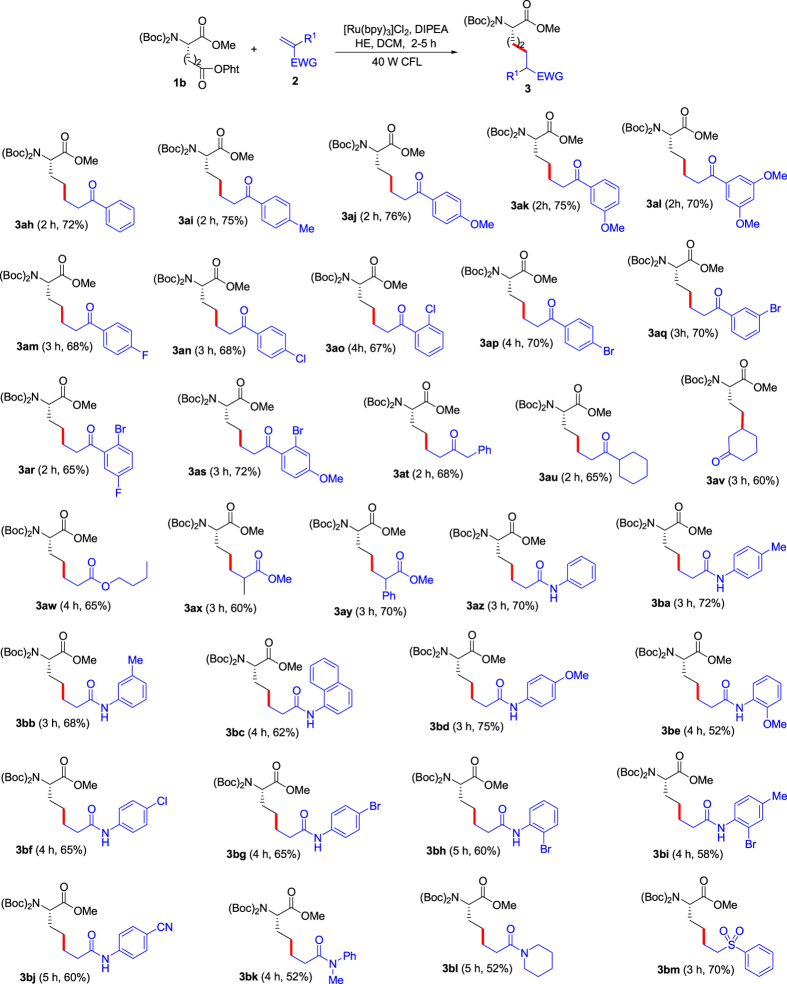
Substrate scope for decarboxylative couplings of *N*-Bis(Boc)-Glu(OPht)-OMe (1b) with olefins (2). *Reaction conditions: under vacuum and irradiation of visible light, *N*-Bis(Boc)-Glu(OPht)-OMe (**1b**) (0.10 mmol using ketones or esters as the partners; 0.15 mmol using amides as the partners), olefin (**2**) (0.15 mmol for ketones and esters; 0.10 mmol for amides), [Ru(bpy)_3_]Cl_2_ (1.0 μmol), diisopropylethylamine (DIPEA) (0.25 mmol), Hantzsch ester (HE) (0.15 mmol), DCM (1.0 mL), temperature (rt, ~25 °C), time (1–5 h) in a sealed Schlenk tube. ^†^Isolated yield.

At first, *N*-Bis(Boc)-Asp(OPht)-OMe (**1a**) was applied as the chiral source and radical precursor, 1-phenylprop-2-en-1-one (**2a**) as the radical receptor to optimize reaction conditions including photocatalysts, solvents, atmosphere, and time (*see* Supplementary Materials *for the details*), and the results showed that the optimal conditions for the decarboxylative coupling are as follows: 1 mol% [Ru(bpy)_3_]Cl_2_ as the photocatalyst, dichloromethane (DCM) as the solvent in the presence of diisopropylethylamine (DIPEA) and Hantzsch ester (HE) under vacuum and irradiation of visible light at room temperature. With the optimized photoredox conditions in hand, we next evaluated the scope of olefins as the radical acceptors, including *α*,*β*-unsaturated ketones, esters, amides and a sulfone (using *N*-Bis(Boc)-Asp(OPht)-OMe (**1a**) as the chiral source and radical precursor) (Fig. 2). For *α*,*β*-unsaturated ketones with aryl, the substrates containing electron-donating groups on the aryl rings provided higher yields than those containing electron-withdrawing groups (see **3a**-**l** in Fig. 2). The *α*,*β*-unsaturated ketones with aliphatic alkyls also exhibited good reactivity (see **3m**-**o** in Fig. 2), but the internal alkene was slightly weaker than terminal alkenes (compare **3n** and **3o** in Fig. 2). Three *α*,*β*-unsaturated esters were employed as the radical acceptors (see **3p**-**r** in Fig. 2), and the substrate with *α*-phenyl provided the highest yield (see **3r** in Fig. 2). Furthermore, *α*,*β*-unsaturated amides were also suitable radical acceptors (see **3s**-**af** in Fig. 2). For the substrates made from primary arylamines, affect of the substituents on the aryl rings was not evident (see **3s**-**ad** in Fig. 2). The substrates from aliphatic amines displayed slightly weaker reactivity (see **3ae** and **3af** in Fig. 2). We attempted a *α*,*β*-unsaturated sulfone, and it afforded the corresponding target product in 72% yield (see **3ag** in Fig. 2). The visible-light photoredox decarboxylative couplings exhibited excellent tolerance of functional groups including amides, esters, ethers, C-F, C-Cl, C-Br bonds and cyan in the substrates. Further, we investigated *N*-Bis(Boc)-Glu(OPht)-OMe (**1b**) as the chiral source and radical precursor using the same olefins (Fig. 3). To our pleasure, the dacarboxylative couplings exhibited similar results to the reactions from *N*-Bis(Boc)-Asp(OPht)-OMe (**1a**). Therefore, the present method showed effective commonality which provides opportunity for construction of diverse chiral amino acids.

We investigated whether the dacarboxylative couplings above led to racemization of unnatural α-amino acid derivatives (**3a-bm**). At first, three racemates, *Rac***-3a**, *Rac*-**3s** and *Rac*-**3w**, were prepared, and then the three racemates, **3a**, **3s** and **3w** in Fig. 2 were determined by HPLC with ID-H chiral column using *n*-hexane/isopropanol (90:10) as the mobile phase (column pressure = 42 bar, flow rate = 1 ml/min), and the results exhibited that no racemization was observed in the synthesis of unnatural chiral α-amino acids (see Supplementary Information for the details).

### Synthesis of compounds 5a-n

**Figure 4:**
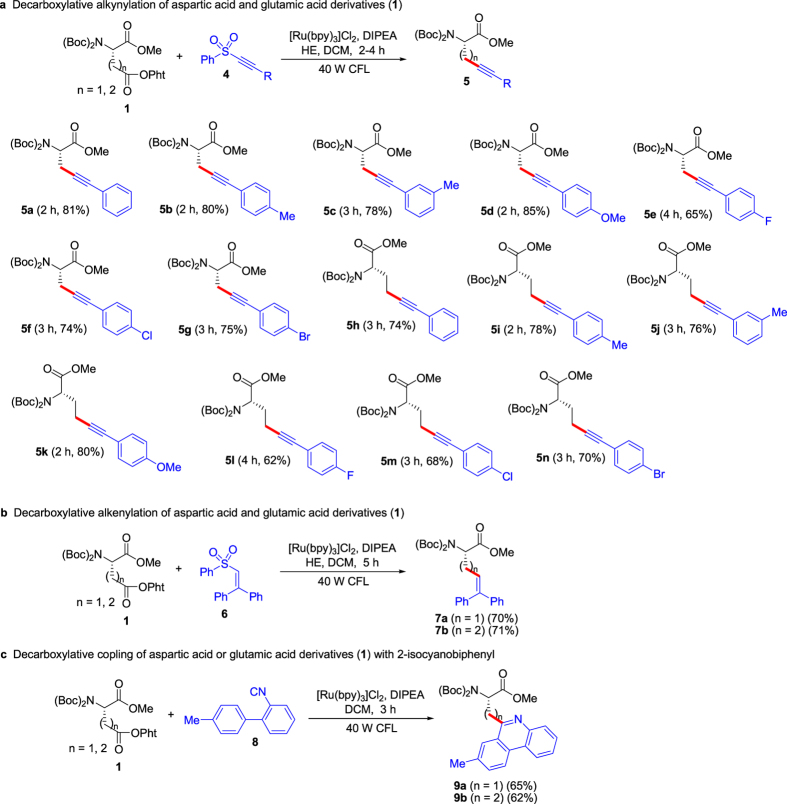
Substrate scope for decarboxylative alkynylation, alkenylation and arylation of L-aspartic acid and glutamic acid derivatives (1). (**a**) Decarboxylative alkynylation of aspartic acid and glutamic acid derivatives (1). (**b**) Decarboxylative alkenylation of aspartic acid and glutamic acid derivatives (1). (**c**) Decarboxylative coupling of aspartic acid or glutamic acid derivatives (1) with 2-isocyanobiphenyl. ^*^Reaction conditions: under vacuum and irradiation of visible light, *N*-Bis(Boc)-Asp(OPht)-OMe (**1a**) or *N*-Bis(Boc)-Glu(OPht)-OMe (**1b**) (0.15 mmol using alkynyl sulfones or 2-isocyanobiphenyl as the partners; 0.10 mmol using alkenyl sulfone as the partner), alkynyl sulfone (4) (0.10 mmol), alkenyl sulfone (6) (0.15 mmol), 2-isocyanobiphenyl (8) (0.10 mmol), [Ru(bpy)_3_]Cl_2_ (1.0 μmol), DIPEA (0.25 mmol), HE (0.15 mmol), DCM (1.0 mL), temperature (rt, ~25 °C), time (2–4 h) in a sealed Schlenk tube. ^†^Isolated yield.

Inspired by the excellent results above, we continued synthesis of diverse unnatural chiral α-AAs. As shown in Fig. 4a, decarboxylative alkynylation of aspartic acid and glutamic acid derivatives with alkynyl sulfones was investigated. The corresponding *α*-AAs (**5a-n**) containing alkynyls were prepared in good yields, and the substrates with electron-donating groups on the aryl rings displayed slightly higher reactivity than those with electron-withdrawing groups. The method showed tolerance of functional groups such as ethers, C-F, C-Cl and C-Br bonds in the substrates. Occurrence of alkynes in the prepared unnatural chiral α-AAs affords opportunity for their further modification.

### Synthesis of compounds 7a, 7b, 9a and 9b

Furthermore, we extended reactions of aspartic acid and glutamic acid derivatives (**1**). As shown in Fig. 4b, coupling of **1** with alkenyl sulfone **6** afforded the corresponding chiral α-AAs (**7a** and **7b**) containing alkene in 70% and 71% yields, respectively. When 2-isocyanobiphenyl **8** was used as the partner, chiral *α*-AAs (**9a** and **9b**) with phenanthridine were obtained in good yields (Fig. 4c). The results above exhibit that the present strategy using aspartic acid and glutamic acid derivatives as the chiral sources and radical precursors can provide diverse unnatural chiral α-AAs for various fields at a lower cost.

**Figure 5:**
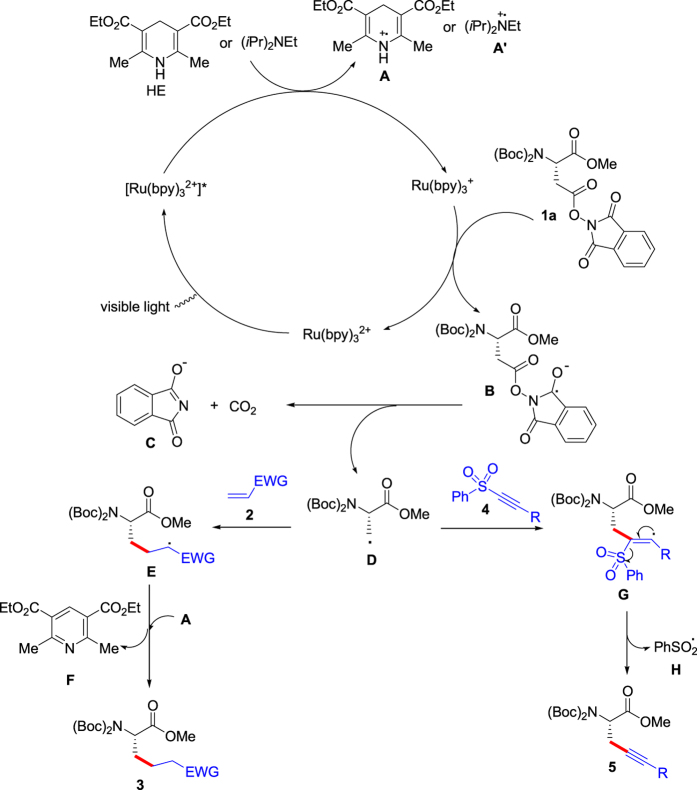
A plausible mechanism for decarboxylative couplings of aspartic acid derivative (1a) under visible-light assistance.

A plausible mechanism on the visible-light photoredox decarboxylative couplings of aspartic acid and glutamic acid derivatives is suggested in Fig. 5 according to the results above and the previous references^18^,^19^,^20^,^21^,^22^,^23^,^24^,^25^,^26^,^27^,^28^,^29^,^30^,^31^. Here, decarboxylative coupling of *N*-Bis(Boc)-Asp(OPht)-OMe (**1a**) was chosen as the example. Irradiation of Ru(bpy)_3_^2+^ with visible light gives the excited-state [Ru(bpy)_3_^2+^]*, and the photoexcited catalyst was reduced by Hantzsch ester (HE) or diisopropylethylamine (DIPEA) to afford Ru(bpy)_3_^+^, in which HE or DIPEA changes into **A** or **A’**. Treatment of **1a** with Ru(bpy)_3_^+^ gives radical anion **B** regenerating catalyst Ru(bpy)_3_^2+^, and subsequent elimination of phthalimide anion (**C**) and CO_2_ from **B** provides radical **D**. Addition of **D** to olefin (**2**) leads to radical **E**, and reaction of **E** with **A** gives product **3** freeing **F**. On the other hand, reaction of **D** with alkynyl sulfone (**4**) donates radical intermediate **G**,^28^ and dissociation of radical **H** from **G** affords product **5**.

In conclusion, we have developed novel and efficient approaches to unnatural chiral α-amino acids at room temperature under visible-light assistance, in which 83 unnatural chiral α-amino acids containing ketones, esters, amides, alkynes, alkene and phenanthridine on the side chains were prepared in good to excellent yields. The protocol uses two readily available genetically coded proteinogenic amino acids, L-aspartic acid and glutamic acid derivatives, as the chiral sources and radical precursors, olefins, alkynyl and alkenyl sulfones, and 2-isocyanobiphenyl as the radical acceptors, and the reactions exhibited excellent tolerance of functional groups. The strategy of keeping chiral α-carbon configuration great decreases cost, improves efficiency and declines waste. Therefore, the present researches pave the way for future synthesis of biological and pharmaceutical molecules containing amino acid and peptide fragments, and we believe that the present strategy will find wide applications in various fields.

## Methods

### General procedure for synthesis of compounds 3a-bm

To a 25-mL Schlenk tube equipped with a Teflon septum and magnetic stir bar were added [Ru(bpy)_3_]Cl_2_ (1.0 μmol, 0.78 mg), *N*-Bis(Boc)-Glu(OPht)-OMe (**1b**) (0.10–0.15 mmol) (using **1b** as the substrate), olefins (**2**) (0.10–0.15 mmol, if solid) (see Fig. 3 for amount of **1b** and **2**) and Hantzsch ester (HE) (0.15 mmol, 38 mg). The tube was evacuated and back-filled with nitrogen for three cycles and then sealed under an atmosphere of nitrogen. *N*-Bis(Boc)-Asp(OPht)-OMe (**1a**) (0.10–0.15 mmol) (using **1a** as the substrate), olefins (**2**) (0.10–0.15 mmol, if liquid) (see Fig. 2 for amount of **1a** and **2**) and DIPEA (0.25 mmol, 42 μL, 32.3 mg) were dissolved in 1.0 mL of dichloromethane (DCM), and then the solution was added to the tube by syringe. The resulting solution was freezed with liquid nitrogen, and the tube was degassed by alternating vacuum evacuation then allowing it to warm to room temperature for three cycles. The tube was irradiated with a 40 W fluorescent lamp at room temperature (approximately 2 cm away from the light source). After the complete conversion of the substrates (monitored by TLC), the reaction mixture was diluted with 20 mL of EtOAc, and the solution was filtered by flash chromatography. The filtrate was evaporated by rotary evaporator, and the residue was purified by silica gel column chromatography or preparative thin layer chromatography (pTLC) to give the desired product (**3a-bm**).

### General procedure for synthesis of compounds 5a-n and 7a,b

To a 25-mL Schlenk tube equipped with a Teflon septum and magnetic stir bar were added [Ru(bpy)_3_]Cl_2_ (1.0 μmol, 0.78 mg), *N*-Bis(Boc)-Glu(OPht)-OMe (**1b**) (0.10–0.15 mmol) (using **1b** as the substrate, see Fig. 4 for amount of **1b**), alkynyl sulfone (**4**) (0.10 mmol) or alkenyl sulfone (**6**) (0.15 mmol, 48 mg) and Hantzsch ester (HE) (0.15 mmol, 38 mg). The tube was evacuated and back-filled with nitrogen for three cycles and then sealed under an atmosphere of nitrogen. *N*-Bis(Boc)-Asp(OPht)-OMe (**1a**) (0.10–0.15 mmol) (using **1a** as the substrate, see Fig. 4 for amount of **1a**) and DIPEA (0.25 mmol, 42 μL, 32.3 mg) were dissolved in 1.0 mL of dichloromethane (DCM), and then the solution was added to the tube by syringe. The resulting solution was freezed with liquid nitrogen, and the tube was degassed by alternating vacuum evacuation then allowing it to warm to room temperature for three cycles. The tube was irradiated with a 40 W fluorescent lamp at room temperature (approximately 2 cm away from the light source). After the complete conversion of the substrates (monitored by TLC), the reaction mixture was diluted with 20 mL of EtOAc, and the solution was filtered by flash chromatography. The filtrate was evaporated by rotary evaporator, and the residue was purified by silica gel column chromatography or preparative thin layer chromatography (pTLC) to give the desired product (**5a-n** or **7a,b**).

### General procedure for synthesis of compounds 9a,b

To a 25-mL Schlenk tube equipped with a Teflon septum and magnetic stir bar were added [Ru(bpy)_3_]Cl_2_ (1.0 μmol, 0.78 mg) and *N*-Bis(Boc)-Glu(OPht)-OMe (**1b**) (0.15 mmol) (using **1b** as the substrate). The tube was evacuated and back-filled with nitrogen for three cycles and then sealed under an atmosphere of nitrogen. *N*-Bis(Boc)-Asp(OPht)-OMe (**1a**) (0.15 mmol) (using **1a** as the substrate), 2-isocyanobiphenyl (**8**) (0.10 mmol, 19.3 mg) and DIPEA (0.25 mmol, 42 μL, 32.3 mg) were dissolved in 1.0 mL of dichloromethane (DCM), and then the solution was added to the tube by syringe. The resulting solution was freezed with liquid nitrogen, and the tube was degassed by alternating vacuum evacuation then allowing it to warm to room temperature for three cycles. The tube was irradiated with a 40 W fluorescent lamp at room temperature (approximately 2 cm away from the light source). Some suspended solids appeared during the reaction. After the complete conversion of the substrates (monitored by TLC), the reaction mixture was diluted with 20 mL of EtOAc, and the solution was filtered by flash chromatography. The filtrate was evaporated by rotary evaporator, and the residue was purified by silica gel column chromatography or preparative thin layer chromatography (pTLC) to give the desired product (**9**).

## Additional Information

**How to cite this article**: Jiang, M. *et al*. Visible-light photoredox synthesis of unnatural chiral α-amino acids. *Sci. Rep.* **6**, 26161; doi: 10.1038/srep26161 (2016).

## Supplementary Material

Supplementary Information

## Figures and Tables

**Figure 1:**
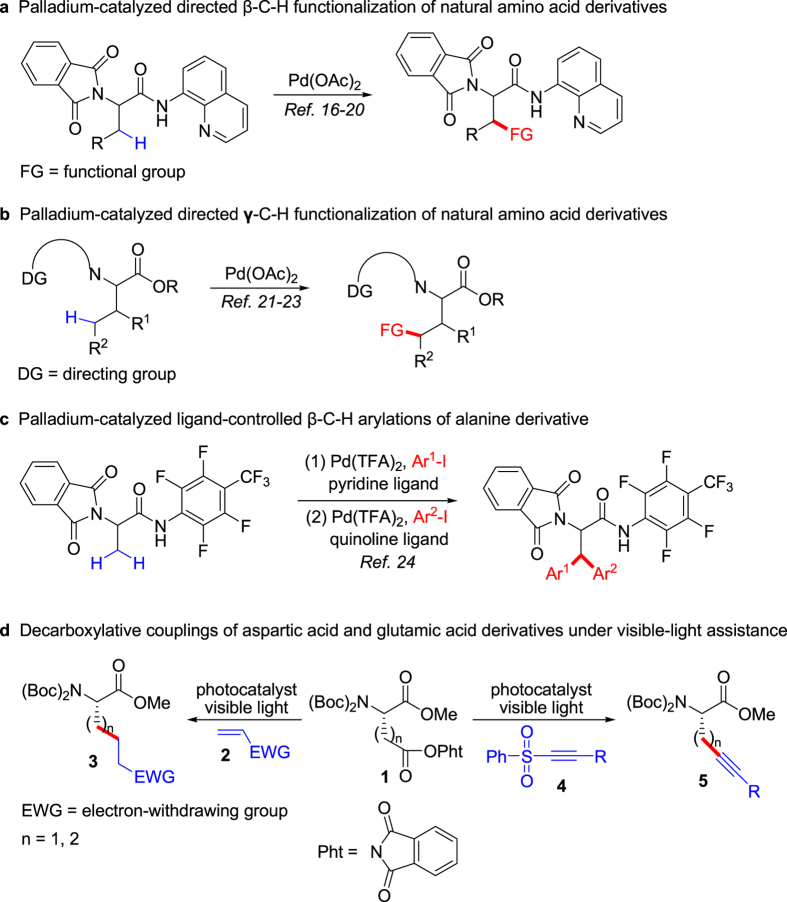
Design for synthesis of unnatural chiral α-amino acids from natural α-amino acids. (**a**) The previous palladium-catalyzed directed *β*-C-H functionalization of natural amino acid derivatives. (**b**) The previous palladium-catalyzed directed *γ*-C-H functionalization of natural amino acid derivatives. (**c**) Yu’s palladium-catalyzed ligand-controlled *β*-C-H arylations of alanine derivative. (**d**) Our strategy on decarboxylative couplings of aspartic acid and glutamic acid derivatives under visible-light assistance.
