# Supplementary material for: Visible-light photoredox synthesis of unnatural chiral α-amino acids
Source: Sci Rep. 2016 May 17;6:26161. doi: 10.1038/srep26161 (PMC4868990; doi:10.1038/srep26161)
Supplement: Supplementary Information [file srep26161-s1.pdf]

Supplementary Information

Visible-light photoredox synthesis of unnatural chiral α-amino acids

Min Jiang, Yunhe Jin, Haijun Yang & Hua Fu

Key Laboratory of Bioorganic Phosphorus Chemistry and Chemical Biology (Ministry of Education), Department of Chemistry, Tsinghua University, Beijing 100084, P. R. China. Correspondence and requests for materials should be addressed to H.F. (fuhua@mail.tsinghua.edu.cn).

Table of contents

General Procedures S2

Experimental Procedures and Characterization Data S2

General Procedure for Synthesis of *N*-Bis(Boc)-Asp(OPht)-OMe (**1a**) and *N*-Bis(Boc)-Glu(OPht)-OMe (**1b**) S2

General Procedure for Synthesis of Vinyl Ketones and Acrylamides (**2**) S6

General Procedure for Synthesis of Alkynyl Sulfones (**4**) and Vinyl Sulfone (**6**) S6

Synthesis of 2-Isocyanobiphenyl (**8**) S8

Optimization Studies S9

General Procedure for Synthesis and Characterization of Compounds **3**, **5**, **7** and **9** S11

General Synthesis and Characterization of *Rac*-**3a**, *Rac*-**3s**, *Rac*-**3w** S62

HPLC of *Rac*-**3a**, **3a**, *Rac*-**3s**, **3s**, *Rac*-**3w**, **3w** S65

References S72

The NMR Spectra of Compounds **1**, **3**, **5**, **7**, **9**, *Rac*-**3a**, *Rac*-**3s** and *Rac*-**3w** S73

General Procedures

All reactions were carried out in dry solvents under a vacuum atmosphere for photocatalysis. Commercial dichloromethane (DCM) was dried over CaH_2 and refluxed over 4 hours. Reagents were purchased and used without further purification. Reactions were monitored by thin layer chromatography (TLC), and the products were obtained by column chromatography on silica gel or preparative thin layer chromatography (pTLC). High resolution mass spectra (HRMS) were recorded on a Shimadzu LCMS-IT/TOF quadrupole-time of flight mass spectrometer. NMR spectra were recorded on JOEL JNM-ECA 600, JNM-ECS 400 and JNM-ECA 300. For proton and carbon magnetic resonance spectra (^{1}H NMR and ^{13}C NMR), tetramethylsilane (TMS) in the solvent of $CDCl_3$ was used as the internal standard (^{1}H NMR: TMS at 0.00 ppm, $CHCl_3$ at 7.26 ppm; ^{13}C NMR: $CDCl_3$ at 77.16 ppm). The following abbreviations were used to explain the multiplicities: s = singlet, d = doublet, t = triplet, q = quartet, m = multiplet, b = broad.

Abbreviations: DMF: *N*,*N*-dimethylformamide; DMAP: *N*,*N*-dimethyl-4-aminopyridine; THF: tetrahydrofuran; EtOAc: ethyl acetate; DCM: dichloromethane; Boc_2O: di-*tert*-butyldicarbonate

Experimental Procedures and Characterization Data

General Procedure for Synthesis of *N*-Bis(Boc)-Asp(OPht)-OMe (1a) and *N*-Bis(Boc)-Glu(OPht)-OMe (1b)

M1, M2 and **M3** were prepared by following the procedures in the reference[1].

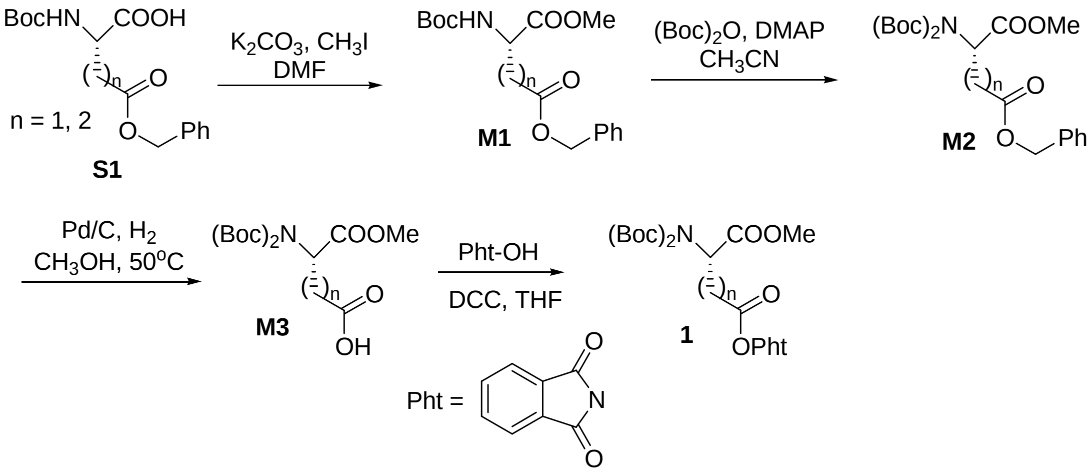

***N*-Boc-Asp(OBn)-OMe (M1a).** Boc-Asp(OBzl)-OH (**S1**) (4.8 g, 14.9 mmol) was dissolved in dry DMF (35 mL) in a 100 mL round bottom flask. Finely ground K_2CO_3 (3.0 g, 22 mmol) was added to the solution. The mixture was cooled to 0 ^oC in an ice bath over five minutes. Methyl iodide (2.0 mL, 30 mmol) was then added to the flask over 30 seconds. The resulting mixture was stirred at 0 ^oC for 3 hours. A yellow color developed within 20-30 minutes. The ice bath was removed, and H_2O (50 mL) was added, producing a grayish white precipitate. The mixture was extracted with EtOAc (3 × 50 mL). The combined organic layer was washed with saturated $NaHCO_3$ (50 mL) and saturated NaCl (4 × 50 mL) and dried over Na_2SO_4, and passed through a plug of silica, eluting with ethylacetate. Evaporation under reduced pressure afforded **M1a** as a white powder, yield: 4.93 g (98 %).

***N*-Boc$_2$-Asp(OBzl)-OMe (M2a)**. Di-*tert*-butyl dicarbonate (4.78g, 22 mmol), DMAP (1.79 g, 14.6 mmol), **M1a** (4.93 g, 14.6 mmol) and dry acetonitrile (30 mL) were added to a dry 100 mL round bottom flask, and the resulting solution was stirred for 24 hours, meanwhile the solution

color changed from light to dark yellow. Water (50 mL) was added, and the solution became cloudy white. The resulting mixture was extracted with EtOAc (3 × 50 mL). The combined organic layer was washed with 1 M HCl (50 mL) and saturated NaCl (4 × 50 mL) and dried over Na_2SO_4, and evaporated under reduced pressure to afford **M2a** as a viscous red oil, yield: 6.17 g (96%).

***N*-Boc₂-Asp-OMe (M3a)**. Palladium on activated carbon (1.2 g, 10% by weight) was added to a dry 100 mL round bottom flask charged with **M2a** (6.17 g, 14.0 mmol) and MeOH (30 mL). A H_2 balloon was attached to the reaction vessel equipped with a three way valve. The mixture was degassed by quickly alternating vacuum evacuation and H_2 backfill three times. Then the flask was placed to a 50 ^{o}C oil bath for 12 hours. After the reaction completed, the mixture was filtered to remove the Pd/C, and the solvent of the remaining solution was evaporated under reduced pressure to afford **M3a** as a light yellow oil, yield: 4.62 g (95%).

***N*-Boc$_2$-Asp(OPht)-OMe (1a). 1a** was synthesized by following the procedure in the reference[2]. *N*-Hydroxyphthalimide (2.39 g, 14.63 mmol) was added to a 100 mL round bottom flask charged with **M3a** (4.62 g, 13.3 mmol) and dry THF (30 mL). After the mixture was stirred for over 10 minutes, DCC (2.74 g, 13.3 mmol) was added to the flask in portions, and the reaction was performed at room temperature for 24 hours. After the reaction completed, the resulting mixture was filtered, the solvent of the remaining solution was removed under reduced pressure, and the crude product was purified by flash silica gel column chromatography (DCM/EtOAc = 60/1, R_f = 0.52) to give **1a** as a viscous pale yellow oil, yield: 4.9 g (75%).

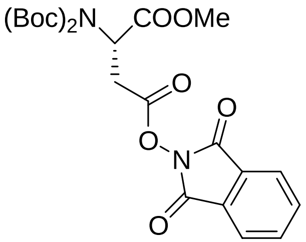

1a

***N*-Boc$_2$-Asp(OPht)-OMe (1a)**. Viscous pale yellow oil. HRMS (ESI-TOF) calculated for $C_{23}H_{28}N_2O_{10}$ $[M+Na]^+$ m/z 515.1636, found 515.1642.

^{1}H NMR ($CDCl_3$, 400 MHz) δ 7.88 (dd, J_1 = 5.5 Hz, J_2 = 3.2 Hz, 2H), 7.79 (dd, J_1 = 5.5 Hz, J_2 = 3.2 Hz, 2H), 5.53 (dd, J_1 = 7.6 Hz, J_2 = 6.0 Hz, 1H), 3.8 (s, 3H), 3.66 (dd, J_1 = 17.2 Hz, J_2 = 5.95 Hz, 1H), 3.11 (dd, J_1 = 17.2 Hz, J_2 = 6.0 Hz, 1H), 1.51 (s, 18H).

^{13}C NMR ($CDCl_3$, 100 MHz) δ 169.5, 167.2, 161.7, 151.5, 134.8, 129.0, 124.0, 84.0, 54.5, 52.9, 33.0, 28.0.

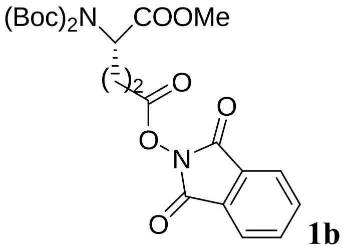

1b

Boc$_2$-Glu(Pht)-OMe (1b). 1b was prepared according to the similar procedures above. Eluent: DCM/EtOAc = 60/1, R_f = 0.52. Pale yellow solid, mp 45-48 °C. HRMS (ESI-TOF) calculated for $C_{24}H_{30}N_2O_{10}$ $[M+Na]^+$ m/z 529.1793, found 529.1792.

^{1}H NMR ($CDCl_3$, 400 MHz) δ 7.88 (dd, J_1 = 5.5 Hz, J_2 = 3.2 Hz, 2H), 7.79 (dd, J_1 = 5.5 Hz, J_2 = 3.2 Hz, 2H), 4.99 (dd, J_1 = 9.2 Hz, J_2 = 5.5 Hz, 1H), 3.7 (s, 3H), 2.87-2.75 (m, 2H), 2.68-2.59 (m, 1H), 2.37-2.27 (m, 1H), 1.51 (s, 18H).

^{13}C NMR ($CDCl_3$, 100 MHz) δ 170.5, 169.0, 161.9, 152.0, 134.8, 128.9, 124.0, 83.7, 57.1, 52.4, 28.0, 27.9, 25.2.

General Procedure for Synthesis of Vinyl Ketones and Acrylamides (2).

Synthesis of Vinyl Ketones (2). They were prepared according to the previous literature[3]. To a 20 mL round bottom flask charged with N_2 added 5 mmol of aryl or aliphatic aldehyde. The flask was cooled to 0 ^oC under ice-bath and 30 mL of dry THF was injected with vigorous stirring, and then 6 mmol of vinyl magnesium bromide solution in THF was injected dropwise. The mixture was stirred under ice-bath for 0.5 hour, and then warmed to room temperature and kept stirring for 3-24 hours. After completion was indicated by TLC, the reaction mixture was cooled to 0 ^oC and quenched with aqueous NH_4Cl, then extracted with ether. The combined organic layer was concentrated to afford allyl alcohol for further transformation without purification. The allyl alcohol was dissolved in 30 mL of THF and cooled to 0 ^oC. 4.5 mL of freshly prepared Jones reagent (1.24 M) was added slowly, and the solution was stirred at room temperature for 2-4 hours. After completion was indicated by TLC, the mixture was extracted with ether (10 mL) three times and organic layer was dried over anhydrate Na_2SO_4, filtrated, concentrated, and the residue was purified by flash chromatography to afford vinyl ketone (**2**).

Synthesis of Acrylamides (2). Arcyloylchloride (492.5 mg, 404 uL, 5 mmol) was dissolved in dry DCM (20 mL), amines (5 mmol) and triehylamine (556.5 mg, 764.5 uL, 5.5 mmol) in 5 mL of DCM was added. After 2 hours, 20 mL of H_2O was added to the reaction mixture, organic layer was washed with water (2 × 10 mL), and the remained organic layer was dried over anhydrate Na_2SO_4, evaporated under reduced pressure to provide products without further purification.

General Procedure for Synthesis of Alkynyl Sulfones (4) and Vinyl Sulfone (6).

Alkynyl sulfones and vinyl sulfones were prepared according to the previous literatures[4,5].

General Procedure for Synthesis of Alkynyl Sulfones (4)

Procedure A: To a mixture of arylacetylene (1 mmol), sodium *p*-toluenesulfinate (1.2 mmol) and NaI (1.2 mmol) in anhydrous CH_3CN (5 mL) was added a solution of CAN (2.5 mmol) in the same solvent (10 mL) under an argon atmosphere. After the completion of the reaction, the reaction mixture was extracted with CH_2Cl_2, and the CH_2Cl_2 layer was separated, washed with brine (50 mL) and dried over anhydrous Na_2SO_4. The residue after removing the solvent was refluxed with K_2CO_3 (2 mmol) in anhydrous acetone (5 mL) for about 3 hours. After the completion of the reaction, the reaction mixture was washed with H_2O (50 mL) and extracted with CH_2Cl_2 (3 × 20 mL). The combined organic phase was washed with brine (2 mL) and dried over anhydrous Na_2SO_4. The solvent was removed in vacuo using a rotary evaporator, and the residue was chromatographed to afford the target product (**4**).

Procedure B: To a solution of terminal alkyne (1 mmol) in THF (0.5 M) was added *n*-BuLi (1.1 mmol, 2.5 M in THF) at -78 ^{o}C. After stirring for 30 minutes, a solution of diphenyl disulfide (1.1 mmol) in THF (0.75 M) was added. The reaction mixture was warmed to 25 ^{o}C and stirred for additional 2 hours. To trap the resulting phenyl thiol, 4-nitrobenzyl bromide (1.2 equiv.) was added after cooling to -40 ^{o}C, and then the reaction mixture was warmed to 25 ^{o}C and stirred for additional 1 hour. After quenched with saturated aqueous NH_4Cl, the solution was extracted with EtOAc, and the organic phase was dried over anhydrous Na_2SO_4, concentrated in vacuo to afford the alkynyl thioether. The crude alkynyl thioether was dissolved in DCM (0.5 M), 3-chloroperbenzoic acid (*m*CPBA, 2.4 mmol, 80%) was added at 0 ^{o}C, and the solution was stirred until TLC indicated the complete consumption of the thioether (typically with 3 hours). The resulting reaction mixture was quenched with saturated $Na_2S_2O_6$ solution, and extracted with DCM. The organic phase was washed with saturated Na_2CO_3 and brine, dried over anhydrous Na_2SO_4, concentrated in vacuo, and the residue was purified by column chromatography to afford the alkynyl sulfone (**4**).

General Procedure for Synthesis of Vinyl Sulfone (6)

A mixture of of styrene (1 mmol), sodium *p*-toluenesulfinate (1.2 mmol) and NaI (1.2 mmol) in anhydrous CH_3CN (10 mL) was treated with CAN (1.37 g, 2.5 mmol) in anhydrous CH_3CN (15 mL) under an argon atmosphere for 45 minutes. After completion of the reaction, the reaction mixture was washed with H_2O (50 mL) and extracted with CH_2Cl_2 (3 × 20 mL). The combined organic phase was washed with saturated $Na_2S_2O_3$ solution (3 mL), brine (2 mL), and dried over anhydrous Na_2SO_4. The solvent in the resulting solution was removed using a rotary evaporator, and the residue was chromatographed to afford the product (**6**).

Synthesis of 2-Isocyanobiphenyl (8)

2-Isocyanobiphenyl (**8**) was prepared according to the previous reference[6]. 2-Aminobiphenyl (1.0 mmol) was dissolved in 4.0 mL of THF. After being cooled to 0 ^{o}C, acetic formic anhydride (10 mmol, 0.9 mL), which was prepared from the reaction of acetic anhydride with formic acid at 55 ^{o}C for 2 hours, was added dropwise to the solution at 0 ^{o}C. After the addition was completed, the mixture was warmed to room temperature and stirred for 1 hour. The mixture was treated with saturated aqueous solution of $NaHCO_3$ and extracted with EtOAc three times (3×5 mL). The combined organic phase was dried over anhydrous Na_2SO_4 and concentrated under vacuum. The residue was dissolved in 3.0 mL of THF, and NEt_3 (6 mmol, 830 μL) was added. The solution was added to an over-dried two-neck flask under N_2 atmosphere. After the reaction mixture was cooled to 0 ^{o}C with ice bath, $POCl_3$ (8.7 mmol, 0.8 mL) was added *via* syring pump for a period of 2 hours. The resulting mixture was stirred at 0 ^{o}C for an additional 1 hour, and then the reaction was quenched with saturated aqueous solution of $NaHCO_3$. The resulting solution was extracted with EtOAc (3×10 mL). The combined organic layer was dried over anhydrous Na_2SO_4, filtered and evaporated, and the residue was purified by a silica gel column chromatography to give substituted 2-isocyanobiphenyl (**8**).

Optimization Studies

Table S1. Optimization of conditions on coupling of *N*-Bis(Boc)-Asp(OPht)-OMe (1a) with 1-phenylprop-2-en-1-one (2a)*

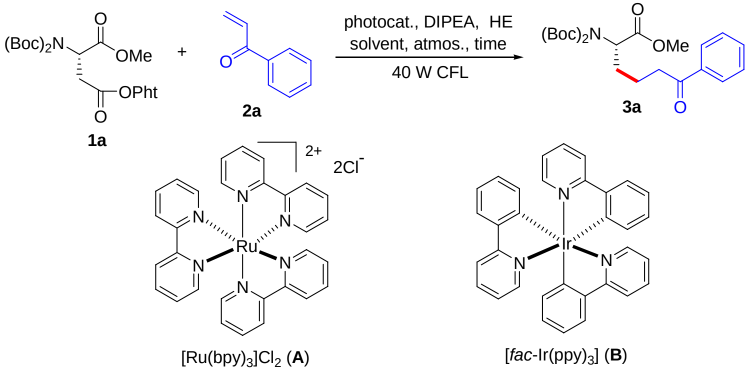

entry	photocat.	solvent	atmos.	time (h)	yield†
1	A	THF	N_2	2	70
2	A	DCM	N_2	2	78
3	A	DMF	N_2	2	42
4	A	DMSO	N_2	2	40
5	A	MeCN	N_2	2	70
6	A	DCM	Ar	2	77
7	A	DCM	air	2	trace
8	A	DCM	vacuum	2	86
9	A	DCM	vacuum	1	75
10	B	DCM	vacuum	2	34
11	‡	DCM	vacuum	2	0
12	A	DCM	vacuum	2	0§

*Reaction conditions: under irradiation of visible light, *N*-Bis(Boc)-Asp(OPht)-OMe (**1a**) (0.1 mmol), 1-phenylprop-2-en-1-one (**2a**) (0.15 mmol), photocatalyst (1 umol), diisopropylethylamine amine (DIPEA) (0.25 mmol), Hantzsch ester (HE) (0.15 mmol), solvent (1.0 mL), temperature (rt, ~25 ^{o}C), time (1-2 h) in a sealed Schlenk tube. [†]Isolated yield. [‡]No addition of photocatalyst. [§]No light. *N*-Boc = *N*-tertbutoxycarbonyl. DCM = dichloromethane. DMF = *N,N*-dimethylformamide. DMSO = dimethyl sulfoxide. CFL = compact fluorescent light.

At first, we chose *N*-Boc-Asp(OPht)-OMe as the chiral source and radical precursor, 1-phenylprop-2-en-1-one (**2a**) as the radical receptor. Unfortunately, the decarboxylative coupling gave poor results under various photoredox conditions. Subsequently, *N*-Bis(Boc)-Asp(OPht)-OMe (**1a**) was applied as the substrate to optimize reaction conditions including photocatalysts, solvents, atmosphere, and time. As shown in Table S1, the reaction provided target product **3a** in 70% yield using 1 mol% $[Ru(bpy)_3]Cl_2$ as the photocatalyst, anhydrous THF as the solvent under nitrogen atmosphere and irradiation of visible light (with a household 40 W fluorescent light bulb) for 2 hours (entry 1). Other solvents, dichloromethane (DCM), DMF, DMSO and MeCN, were attempted (entries 2-5), and the results showed that DCM was optimal solvent (entry 2). We also investigated other atmospheres (entries 6-8), and the reaction was performed well under vacuum condition (entry 8). Shortening of time lead to a lower yield (entry 9). Reaction efficiency obviously decreased when [*fac*-Ir(ppy)$_3$] was used as the photocatalyst (entry 10). The reaction did not work in the absence of photocatalyst (entry 11) or light (entry 12).

General Procedure for Synthesis and Characterization of Compounds 3, 5, 7 and 9

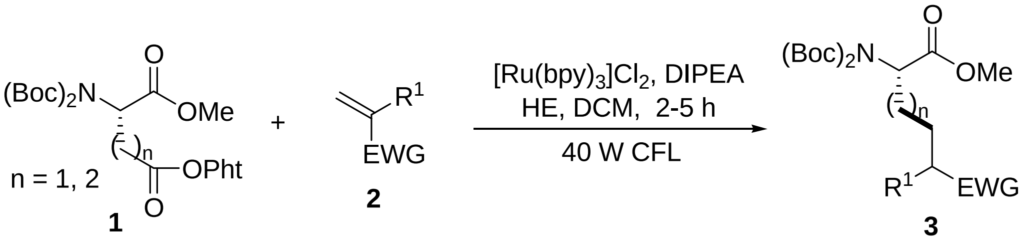

General procedure for synthesis of compounds 3. To a 25-mL Schlenk tube equipped with a Teflon septum and magnetic stir bar were added $[Ru(bpy)_3]Cl_2$ (1.0 μmol, 0.78 mg), *N*-Bis(Boc)-Glu(OPht)-OMe (**1b**) (0.10-0.15 mmol) (using **1b** as the substrate), olefins (**2**) (0.10-0.15 mmol, if solid) (see Fig. 2 for amount of **1b** and **2**) and Hantzsch ester (HE) (0.15 mmol, 38 mg). The tube was evacuated and back-filled with nitrogen for three cycles and then sealed under an atmosphere of nitrogen. *N*-Bis(Boc)-Asp(OPht)-OMe (**1a**) (0.10-0.15 mmol) (using **1a** as the substrate), olefins (**2**) (0.10-0.15 mmol, if liquid) (see Fig. 3 for amount of **1a** and **2**) and DIPEA (0.25 mmol, 42 μL, 32.3 mg) were dissolved in 1.0 mL of dichloromethane (DCM), and then the solution was added to the tube by syringe. The resulting solution was freezed with liquid nitrogen, and the tube was degassed by alternating vacuum evacuation then allowing it to warm to room temperature for three cycles. The tube was irradiated with a 40 W fluorescent lamp at room temperature (approximately 2 cm away from the light source). After the complete conversion of the substrates (monitored by TLC), the reaction mixture was diluted with 20 mL of EtOAc, and the solution was filtered by flash chromatography. The filtrate was evaporated by rotary evaporator, and the residue was purified by silica gel column chromatography or preparative thin layer chromatography (pTLC) to give the desired product (**3**).

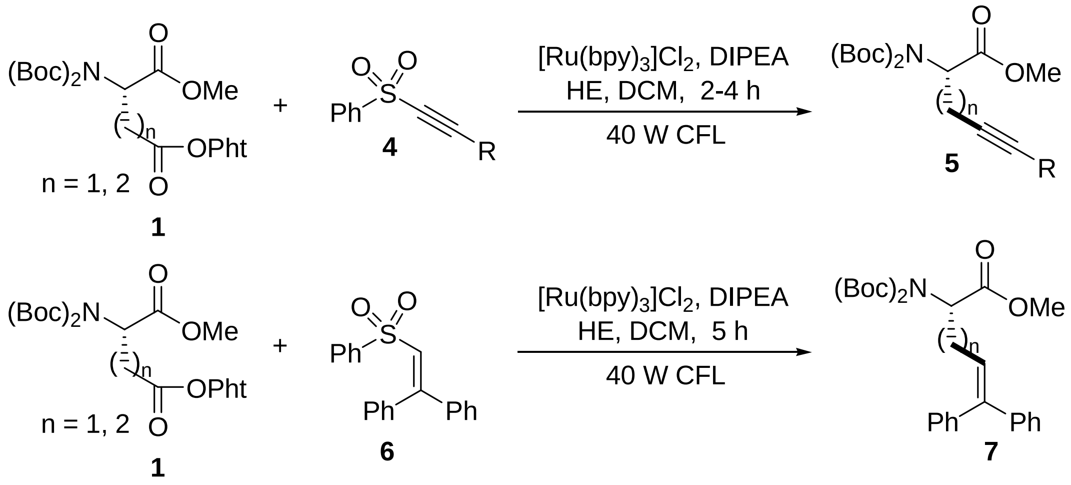

General procedure for synthesis of compounds 5 and 7. To a 25-mL Schlenk tube equipped with a Teflon septum and magnetic stir bar were added $[Ru(bpy)_3]Cl_2$ (1.0 μmol, 0.78 mg), *N*-Bis(Boc)-Glu(OPht)-OMe (**1b**) (0.10-0.15 mmol) (using **1b** as the substrate, see Fig. 4 for amount of **1b**), alkynyl sulfone (**4**) (0.10 mmol) or alkenyl sulfone (**6**) (0.15 mmol, 48 mg) and Hantzsch ester (HE) (0.15 mmol, 38 mg). The tube was evacuated and back-filled with nitrogen for three cycles and then sealed under an atmosphere of nitrogen. *N*-Bis(Boc)-Asp(OPht)-OMe (**1a**) (0.10-0.15 mmol) (using **1a** as the substrate, see Fig. 4 for amount of **1a**) and DIPEA (0.25 mmol, 42 μL, 32.3 mg) were dissolved in 1.0 mL of dichloromethane (DCM), and then the solution was added to the tube by syringe. The resulting solution was freezed with liquid nitrogen, and the tube was degassed by alternating vacuum evacuation then allowing it to warm to room temperature for three cycles. The tube was irradiated with a 40 W fluorescent lamp at room temperature (approximately 2 cm away from the light source). After the complete conversion of the substrates (monitored by TLC), the reaction mixture was diluted with 20 mL of EtOAc, and the solution was filtered by flash chromatography. The filtrate

was evaporated by rotary evaporator, and the residue was purified by silica gel column chromatography or preparative thin layer chromatography (pTLC) to give the desired product (**5** or **7**).

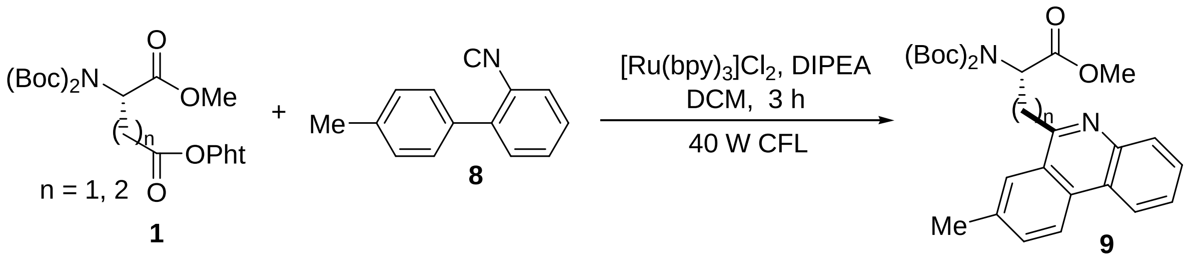

General procedure for synthesis of compounds 9. To a 25-mL Schlenk tube equipped with a Teflon septum and magnetic stir bar were added $[Ru(bpy)_3]Cl_2$ (1.0 μmol, 0.78 mg) and *N*-Bis(Boc)-Glu(OPht)-OMe (**1b**) (0.15 mmol) (using **1b** as the substrate). The tube was evacuated and back-filled with nitrogen for three cycles and then sealed under an atmosphere of nitrogen. *N*-Bis(Boc)-Asp(OPht)-OMe (**1a**) (0.15 mmol) (using **1a** as the substrate), 2-isocyanobiphenyl (**8**) (0.10 mmol, 19.3 mg) and DIPEA (0.25 mmol, 42 μL, 32.3 mg) were dissolved in 1.0 mL of dichloromethane (DCM), and then the solution was added to the tube by syringe. The resulting solution was freezed with liquid nitrogen, and the tube was degassed by alternating vacuum evacuation then allowing it to warm to room temperature for three cycles. The tube was irradiated with a 40 W fluorescent lamp at room temperature (approximately 2 cm away from the light source). Some suspended solids appeared during the reaction. After the complete conversion of the substrates (monitored by TLC), the reaction mixture was diluted with 20 mL of EtOAc, and the solution was filtered by flash chromatography. The filtrate was evaporated by rotary evaporator, and the residue was purified by silica gel column chromatography or preparative thin layer chromatography (pTLC) to give the desired product (**9**).

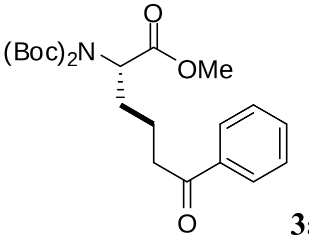

3a

(*S*)-Methyl 2-(bis(*tert*-butoxycarbonyl)amino)-6-oxo-6-phenylhexanoate (3a): Eluent: petroleum ether/ethyl acetate (10:1). Yield: 37.5 mg (86%). Colorless oil. HRMS (ESI-TOF): calculated for $C_{23}H_{33}NO_7$ $[M+Na]^+$ m/z 458.2149, found 458.2146.

^{1}H NMR ($CDCl_3$, 400 MHz) δ 7.94 (d, J = 8.2 Hz, 2H), 7.55 (t, J =7.3 Hz, 1H), 7.45 (t, J =7.8 Hz, 2H), 4.93 (dd, J_1=9.6 Hz, J_2=5.0 Hz, 1H), 3.71 (s, 3H), 3.11-3.03 (m, 1H), 3.00-2.92 (m, 1H), 2.22-2.15 (m, 1H), 2.06-1.97 (m, 1H) , 1.85-1.77 (m, 2H), 1.50 (s, 18H); ^{13}C NMR ($CDCl_3$, 100 MHz) δ 199.7, 171.3, 152.2, 137.1, 133.0, 128.6, 128.1, 83.2, 57.9, 52.2, 38.1, 29.5, 28.1, 20.9.

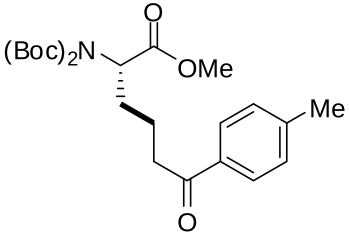

3b

(*S*)-Methyl 2-(bis(*tert*-butoxycarbonyl)amino)-6-oxo-6-*p*-tolylhexanoate (3b): Eluent: petroleum ether/ethyl acetate (10:1). Yield: 40.3 mg (90%). Colorless oil. HRMS (ESI-TOF): calculated for $C_{24}H_{35}NO_7$ $[M+Na]^+$ m/z 472.2306, found 472.2307.

^{1}H NMR ($CDCl_3$, 400 MHz) δ 7.84 (d, J = 8.2 Hz, 2H), 7.24(d, J =8.2 Hz, 2H), 4.93 (dd, J_1=9.6 Hz, J_2=5.0 Hz, 1H), 3.71 (s, 3H), 3.06-2.89 (m, 2H), 2.40 (s, 3H), 2.21-2.14 (m, 1H), 2.04-1.98 (m, 1H), 1.83-1.75 (m, 2H), 1.50 (s, 18H); ^{13}C NMR ($CDCl_3$, 100 MHz) δ 199.3, 171.3,

152.2, 143.7, 134.6, 129.3, 128.2, 83.2, 58.0, 52.2, 38.4, 38.0, 29.5, 28.0, 24.1, 21.7, 21.0.

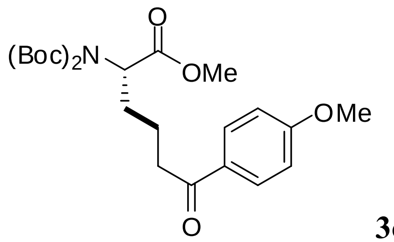

3c

(*S*)-Methyl 2-(bis(*tert*-butoxycarbonyl)amino)-6-(4-methoxyphenyl)-6-oxohexanoate (3c): Eluent: petroleum ether/ethyl acetate (10:1). Yield: 42.9 mg (92%). Light yellow oil. HRMS (ESI-TOF): calculated for $C_{24}H_{35}NO_8$ $[M+Na]^+$ m/z 488.2255, found 488.2257.

^{1}H NMR ($CDCl_3$, 400 MHz) δ 7.93 (d, *J* = 8.7 Hz, 2H), 6.92 (d, *J* =8.7 Hz, 2H), 4.93 (dd, J_1=9.6 Hz, J_2=5.0 Hz, 1H), 3.86 (s, 3H), 3.71 (s, 3H), 3.05-2.85 (m, 2H), 2.23-2.14 (m, 1H), 2.04-1.96 (m, 1H), 1.83-1.76 (m, 2H), 1.50 (s, 18H); ^{13}C NMR ($CDCl_3$, 100 MHz) δ 198.3, 171.4, 163.4, 152.2, 130.3, 130.2, 113.8, 83.2, 58.0, 55.5, 52.2, 37.7, 29.6, 28.0, 21.1.

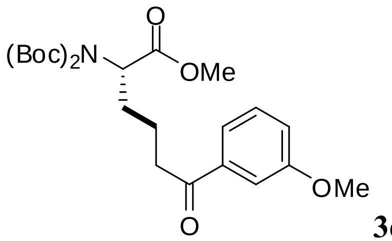

3d

(*S*)-Methyl 2-(bis(*tert*-butoxycarbonyl)amino)-6-(3-methoxyphenyl)-6-oxohexanoate (3d): Eluent: petroleum ether/ethyl acetate (10:1). Yield: 40 mg (85%). Light yellow oil. HRMS (ESI-TOF): calculated for $C_{24}H_{35}NO_8$ $[M+Na]^+$ m/z 488.2255, found 488.2257.

^{1}H NMR ($CDCl_3$, 400 MHz) δ 7.52 (d, *J* =7.79 Hz, 1H), 7.47(s, 1H), 7.35 (t, *J* =8.24 Hz, 1H), 7.09 (dd, J_1 =8.24 Hz, J_2=2.75 Hz, 1H), 4.93 (dd,

J_1=9.6 Hz, J_2=5.0 Hz, 1H), 3.85 (s, 3H), 3.71 (s, 3H), 3.05-2.85 (m, 2H), 2.23-2.14 (m, 1H), 2.06-1.96 (m, 1H), 1.84-1.76 (m, 2H), 1.50 (s, 18H); ^{13}C NMR ($CDCl_3$, 100 MHz) δ 199.5, 171.4, 160.0, 152.3,138.5, 129.6, 120.8, 119.6, 112.3, 83.3, 58.0, 55.5, 52.3, 38.2, 29.5, 28.1, 21.0.

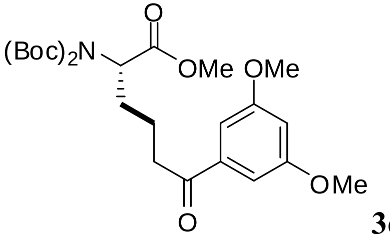

3e

(*S*)-Methyl 2-(bis(*tert*-butoxycarbonyl)amino)-6-(3,5-dimethoxyphenyl)-6-oxohexanoate (3e): Eluent: petroleum ether/ethyl acetate (7:1). Yield: 40.7 mg (82%). Light yellow oil. HRMS (ESI-TOF): calculated for $C_{25}H_{37}NO_9$ $[M+Na]^+$ m/z 518.2391, found 518.2396.

^{1}H NMR ($CDCl_3$, 400 MHz) δ 7.07 (s, 2H), 6.63 (s, 1H), 4.92 (dd, J_1=9.6 Hz, J_2=5.0 Hz, 1H), 3.83 (s, 6H), 3.71 (s, 3H), 3.07-2.86 (m, 2H), 2.24-2.14 (m, 1H), 2.04-1.96 (m, 1H), 1.84-1.74 (m, 2H), 1.49 (s, 18H); ^{13}C NMR ($CDCl_3$, 100 MHz) δ 199.3, 171.3, 160.9, 152.2,139.0, 105.9, 105.3, 83.2, 58.0, 55.6, 52.2, 38.2, 29.5, 28.0, 21.0.

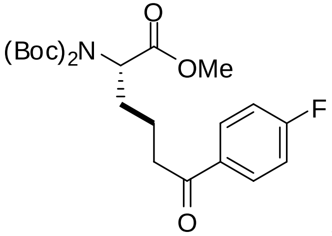

3f

(*S*)-Methyl 2-(bis(*tert*-butoxycarbonyl)amino)-6-(4-fluorophenyl)-6-oxohexanoate (3f): Eluent: petroleum ether/ethyl acetate (10:1). Yield: 35.2 mg (78%). Colorless oil. HRMS (ESI-TOF): calculated for $C_{23}H_{32}FNO_7$ $[M+Na]^+$ m/z 476.2055, found 476.2051.

^{1}H NMR ($CDCl_3$, 400 MHz) δ 7.97 (dd, J_1=8.7 Hz, J_2=5.5 Hz,, 2H), 7.12 (t, J =8.7 Hz, 2H), 4.92 (dd, J_1=9.6 Hz, J_2=5.0 Hz, 1H), 3.71 (s, 3H), 3.08-2.86 (m, 2H), 2.40 (s, 3H), 2.23-2.14 (m, 1H), 2.06-1.96 (m, 1H), 1.86-1.74 (m, 2H), 1.49 (s, 18H); ^{13}C NMR ($CDCl_3$, 100 MHz) δ 198.0, 171.3, 167.0, 164.5, 152.3, 133.54, 133.52, 130.7, 130.6, 115.8, 115.6, 83.3, 57.9, 52.3, 38.0, 29.5, 28.1, 20.9.

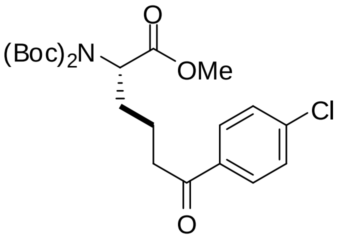

3g

(*S*)-Methyl 2-(bis(*tert*-butoxycarbonyl)amino)-6-(4-chlorophenyl)-6-oxohexanoate (3g):

Eluent: petroleum ether/ethyl acetate (10:1). Yield: 40.0 mg (85%). Colorless oil. HRMS (ESI-TOF): calculated for $C_{23}H_{32}ClNO_7$ $[M+Na]^+$ m/z 492.1760, found 492.1766.

^{1}H NMR ($CDCl_3$, 400 MHz) δ 7.88 (d, J = 8.7 Hz, 2H), 7.42(d, J =8.7 Hz, 2H), 4.93 (dd, J_1=9.6 Hz, J_2=5.0 Hz, 1H), 3.71 (s, 3H), 3.08-2.90 (m, 2H), 2.23-2.14 (m,1H), 2.05-1.96 (m,1H), 1.87-1.74 (m,2H), 1.49 (s, 18H); ^{13}C NMR ($CDCl_3$, 100 MHz) δ 198.4, 171.3, 152.3, 139.4, 135.3, 129.5, 129.0, 83.3, 57.9, 52.32, 38.0, 29.4, 28.0, 20.8.

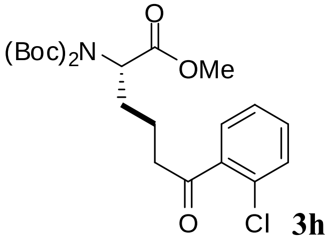

3h

(*S*)-Methyl 2-(bis(*tert*-butoxycarbonyl)amino)-6-(2-chlorophenyl)-6-oxohexanoate (3h): Eluent: petroleum ether/ethyl acetate (10:1). Yield: 32.9 mg (70%). Colorless oil. HRMS (ESI-TOF): calculated for $C_{23}H_{32}ClNO_7$ $[M+Na]^+$ m/z 492.1760, found 492.1766.

^{1}H NMR ($CDCl_3$, 400 MHz) δ 7.43 (d, J = 7.56 Hz, 1H), 7.40-7.35 (m, 2H), 7.30 (t, J = 7.56 Hz, 1H), 4.90 (dd, J_1=9.4 Hz, J_2=5.1 Hz, 1H), 3.71 (s, 3H), 3.04-2.91 (m, 2H), 2.19-2.14 (m, 1H), 2.01-1.99 (m, 1H), 1.80-1.75 (m, 2H), 1.49 (s, 18H); ^{13}C NMR ($CDCl_3$, 100 MHz) δ 202.9, 171.2, 152.2, 139.7, 131.6, 130.8, 130.5, 128.8, 127.0, 83.2, 57.9, 52.2, 42.5, 29.4 28.0, 20.9.

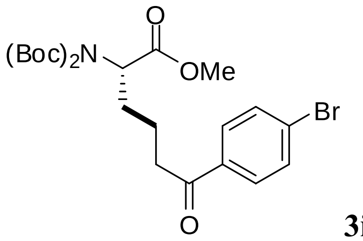

3i

(*S*)-Methyl 2-(bis(*tert*-butoxycarbonyl)amino)-6-(4-bromophenyl)-6-oxohexanoate (3i): Eluent: petroleum ether/ethyl acetate (10:1). Yield: 42.7 mg (83%). Light yellow oil. HRMS (ESI-TOF): calculated for $C_{23}H_{32}BrNO_7$ $[M+Na]^+$ m/z 538.1238, found 538.1238.

^{1}H NMR ($CDCl_3$, 400 MHz) δ 7.81 (d, J = 8.7 Hz, 2H), 7.59 (d, J = 8.7 Hz, 2H), 4.91 (dd, J_1=9.4 Hz, J_2=5.1 Hz, 1H), 3.71 (s, 3H), 3.07-2.88 (m, 2H), 2.22-2.13 (m, 1H), 2.05-1.95 (m, 1H), 1.83-1.76 (m, 2H), 1.49 (s, 18H); ^{13}C NMR ($CDCl_3$, 100 MHz) δ 198.6, 171.3, 152.2, 135.7, 131.9, 129.6, 128.1, 83.3, 57.9, 52.3, 38.0, 29.4, 28.0, 20.8.

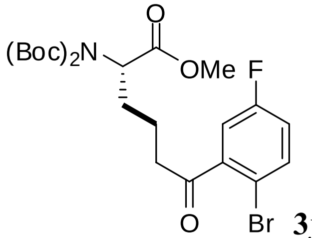

3j

(*S*)-Methyl 2-(bis(*tert*-butoxycarbonyl)amino)-6-(2-bromo-5-fluorophenyl)-6-oxohexanoate (3j): Eluent: petroleum ether/ethyl acetate (10:1). Yield: 36.5 mg (69%). Light yellow oil. HRMS (ESI-TOF): calculated for $C_{23}H_{31}FBrNO_7$ $[M+Na]^+$ m/z 556.1143, found 556.1145.

^{1}H NMR ($CDCl_3$, 400 MHz) δ 7.55 (dd, J_1=8.7 Hz, J_2=5.0 Hz,, 1H), 7.08 (dd, J_1=8.24 Hz, J_2=3.21 Hz, 1H), 7.01 (td, J_1=7.79 Hz, J_2=3.21 Hz, 1H), 4.89 (dd, J_1=9.4 Hz, J_2=5.1 Hz, 1H), 3.71 (s, 3H), 3.04-2.86 (m, 2H), 2.23-2.14 (m, 1H), 2.05-1.95 (m, 1H), 1.82-1.72 (m, 2H), 1.50 (s, 18H); ^{13}C NMR ($CDCl_3$, 100 MHz) δ 202.3, 171.2, 163.0, 160.5, 152.2, 143.4, 143.3, 135.3, 135.2, 118.9, 118.6, 115.8, 115.5, 112.86, 112.82, 83.3, 57.8, 52.3, 42.1, 29.3, 28.1, 20.7.

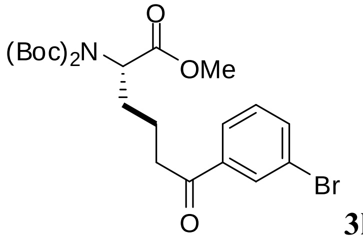

3k

(*S*)-Methyl 2-(bis(*tert*-butoxycarbonyl)amino)-6-(3-bromophenyl)-6-oxohexanoate (3k): Eluent: petroleum ether/ethyl acetate (10:1). Yield: 43.1 mg (84%). Light yellow oil. HRMS (ESI-TOF): calculated for $C_{23}H_{32}BrNO_7$ $[M+Na]^+$ m/z 538.1238, found 538.1238.

^{1}H NMR ($CDCl_3$, 400 MHz) δ 8.07 (s, 1H), 7.86 (d, J_1=7.79 Hz, 1H), 7.67 (d, J_1=7.79 Hz, 1H), 7.33 (t, J_1=7.79 Hz, 1H), 4.91 (dd, J_1=9.4 Hz,

J_2=5.1 Hz, 1H), 3.71 (s, 3H), 3.08-2.87 (m, 2H), 2.23-2.14 (m, 1H), 2.05-1.96 (m, 1H), 1.87-1.74 (m, 2H), 1.49 (s, 18H); ^{13}C NMR ($CDCl_3$, 100 MHz) δ 198.2, 171.3, 152.2, 138.8, 135.9, 131.2, 130.3, 126.6, 123.0, 83.3, 57.9, 52.3, 38.1, 29.4, 28.1, 20.7.

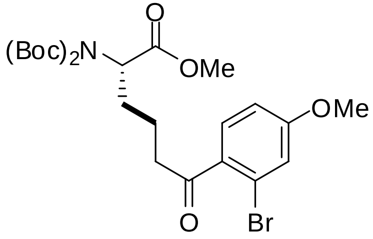

3l

(*S*)-Methyl 2-(bis(*tert*-butoxycarbonyl)amino)-6-(2-bromo-4-methoxyphenyl)-6-oxohexanoate (2l): Eluent: petroleum ether/ethyl acetate (8:1). Yield: 40 mg (73%). Light yellow oil. HRMS (ESI-TOF): calculated for $C_{24}H_{34}BrNO_8$ $[M+Na]^+$ m/z 568.1344, found 568.1341.

^{1}H NMR ($CDCl_3$, 400 MHz) δ 7.47 (d, *J*=8.24 Hz, 1H), 7.13 (d, *J*=2.75 Hz, 1H), 6.86 (dd, J_1=8.47 Hz, J_1=2.75 Hz, 1H), 4.89 (dd, J_1=9.4 Hz, J_2=5.1 Hz, 1H), 3.83 (s, 3H), 3.71 (s, 3H), 3.04-2.87 (m, 2H), 2.21-2.12 (m, 1H), 2.04-1.94 (m, 1H), 1.83-1.70 (m, 2H), 1.49 (s, 18H); ^{13}C NMR ($CDCl_3$, 100 MHz) δ 201.7, 171.3, 161.7, 152.2, 133.1, 130.8, 120.7, 119.4, 113.2, 83.2, 57.9, 55.8, 52.2, 41.7, 29.4, 28.1, 21.2.

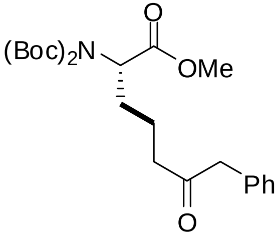

3m

(*S*)-Methyl 2-(bis(*tert*-butoxycarbonyl)amino)-6-oxo-7-phenylheptanoate (3m): Eluent: petroleum ether/ethyl acetate (10:1). Yield: 40.1 mg (89%). Colorless oil. HRMS (ESI-TOF): calculated for $C_{24}H_{35}NO_7$ $[M+Na]^+$ m/z 472.2306, found 472.2308.

^{1}H NMR ($CDCl_3$, 400 MHz) δ 7.34-7.30 (t, J=8.24 Hz, 3H), 7.19 (d, J=6.87 Hz, 2H), 4.81 (dd, J_1=9.4 Hz, J_2=5.1 Hz, 1H), 3.72-3.67 (m, 5H), 2.55-2.42 (m, 2H), 2.08-1.98 (m, 1H), 1.90-1.80 (m, 1H), 1.60 (m, 2H), 1.48 (s, 18H); ^{13}C NMR ($CDCl_3$, 100 MHz) δ 207.9, 171.2, 152.2, 134.4, 129.5, 128.8, 127.1, 83.2, 57.8, 50.2, 41.4, 29.4, 28.0, 20.4.

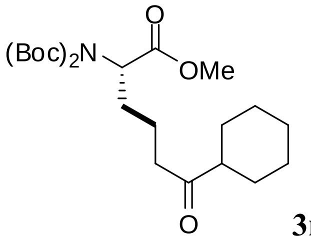

3n

(*S*)-Methyl 2-(bis(*tert*-butoxycarbonyl)amino)-6-cyclohexyl-6-oxohexanoate (3n): Eluent: petroleum ether/ethyl acetate (10:1). Yield: 37 mg (84%). Colorless oil. HRMS (ESI-TOF): calculated for $C_{23}H_{39}NO_7$ $[M+Na]^+$ m/z 464.2619, found 464.2619.

^{1}H NMR ($CDCl_3$, 400 MHz) δ 4.85 (dd, J_1=9.4 Hz, J_2=5.1 Hz, 1H), 3.70 (s, 3H), 2.57-2.38 (m, 2H), 2.33-2.28 (m, 1H), 2.07-2.02 (m, 1H), 1.82-1.76 (m, 2H), 1.67-1.58 (m, 4H), 1.49 (s, 18H), 1.36-1.21 (m, 6H); ^{13}C NMR ($CDCl_3$, 100 MHz) δ 213.6, 171.3, 152.2, 83.2, 57.9, 52.3, 50.9, 40.1, 29.5, 28.6, 28.1, 25.9, 25.8, 20.4.

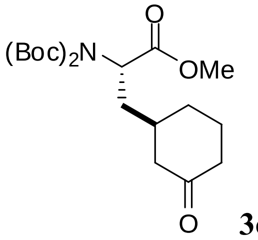

3o

(*S*)-Methyl 2-(bis(*tert*-butoxycarbonyl)amino)-3-((R)-3-oxocyclohexyl)propanoate (3o): Eluent: petroleum ether/ethyl acetate (10:1). Yield:

28.9 mg (72%). Colorless oil. Diastereomeric ratio of γ–C (1:1). HRMS (ESI-TOF): calculated for $C_{20}H_{33}NO_7$ $[M+Na]^+$ m/z 422.2149, found 422.2151.

^1H NMR ($CDCl_3$, 400 MHz) δ 4.99-4.89 (dd, J_1=9.4 Hz, J_2=5.1 Hz, 1H), 3.71 (s, 3H), 2.57-2.21 (m, 2H), 2.29-2.21 (m, 1H), 2.16-2.02 (m, 4H), 1.98-1.87 (m, 2H), 1.64-1.61 (m, 2H), 1.49 (s, 18H); ^{13}C NMR ($CDCl_3$, 100 MHz) δ 211.2, 171.5, 152.2, 83.5, 55.7, 52.4, 41.4, 36.9, 36.0, 31.7, 30.7, 28.1, 25.1.

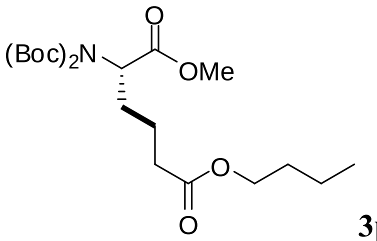

3p

(*S*)-6-Butyl 1-methyl 2-(bis(*tert*-butoxycarbonyl)amino)hexanedioate (3p): Eluent: petroleum ether/ethyl acetate (10:1). Yield: 32.5 mg (75%). Colorless oil. HRMS (ESI-TOF): calculated for $C_{20}H_{33}NO_7$ $[M+Na]^+$ m/z 454.2411, found 454.2411.

^1H NMR ($CDCl_3$, 400 MHz) δ 4.88 (dd, J_1= 9.4 Hz, J_2= 5.1 Hz, 1H), 4.06 (t, J = 6.41 Hz, 2H), 3.71 (s, 3H), 2.42-2.24 (m, 2H), 2.17-2.05 (m, 1H), 1.98-1.84 (m, 1H), 1.74 – 1.65 (m, 2H), 1.63-1.57 (m, 2H), 1.49 (s, 18H), 1.43-1.32 (m, 2H), 0.92 (t, J = 7.33 Hz, 3H); ^{13}C NMR ($CDCl_3$, 100 MHz) δ 173.3, 171.2, 152.1, 83.2, 64.3, 57.8, 52.3, 34.0, 30.7, 29.4, 28.0, 21.8, 19.2, 13.8.

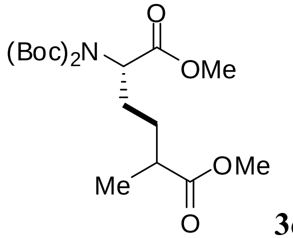

3q

(*S*)-Dimethyl 2-(bis(*tert*-butoxycarbonyl)amino)-5-methylhexanedioate (3q): Eluent: petroleum ether/ethyl acetate (10:1). Yield: 30 mg (74%). Colorless oil. Diastereomeric ratio of δ–C (1:1). HRMS (ESI-TOF): calculated for $C_{19}H_{33}NO_8$ $[M+Na]^+$ m/z 426.2098, found 426.2098. ^{1}H NMR ($CDCl_3$, 400 MHz) δ 4.86-4.80 (m, 1H), 3.68 (s, 3H), 3.64 (s, 3H), 2.51-2.00 (m, 1H), 1.92-1.81 (m, 1H), 1.76-1.60 (m, 2H), 1.47 (s, 18H), 1.15-1.13 (m, 3H); ^{13}C NMR ($CDCl_3$, 100 MHz) δ 176.8, 171.3, 152.1, 83.3, 57.9, 52.2, 51.6, 39.0, 30.4, 30.2, 29.87, 28.1, 27.7, 27.5, 17.3, 16.9.

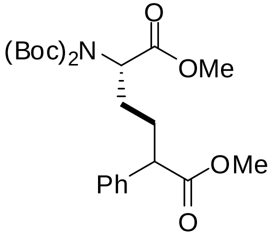

3r

(*S*)-Dimethyl 2-(bis(*tert*-butoxycarbonyl)amino)-5-phenylhexanedioate (3r): Eluent: petroleum ether/ethyl acetate (10:1). Yield: 39.6 mg (85%). Colorless oil. Diastereomeric ratio of δ–C (1:1). HRMS (ESI-TOF): calculated for $C_{24}H_{35}NO_8$ $[M+Na]^+$ m/z 488.2255, found 488.2251. ^{1}H NMR ($CDCl_3$, 400 MHz) δ 7.32-7.22 (m, 5H), 4.91 (m, 1H), 3.74-3.69 (m, 1H), 3.68-3.67 (d, 3H), 3.64 (s, 3H), 3.61-3.54 (m, 1H), 2.17-1.97 (m, 2H), 1.94-174 (m, 2H), 1.50-1.44 (m, 18H); ^{13}C NMR ($CDCl_3$, 100 MHz) δ 174.2, 174.1, 171.2,171.9, 152.1, 152.90, 138.9, 138.5, 128.8,

128.7, 128.0, 127.9, 127.5, 127.4, 83.3, 57.8, 52.2, 52.15, 52.11, 51.1,51.0, 30.3, 29.7, 28.09, 28.04, 27.6.

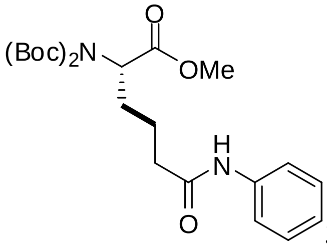

3s

(*S*)-Methyl 2-(bis(*tert*-butoxycarbonyl)amino)-6-oxo-6-(phenylamino)hexanoate (3s): Eluent: petroleum ether/ethyl acetate (5:1). Yield: 32.5 mg (72%). Yellow oil. HRMS (ESI-TOF): calculated for $C_{23}H_{34}N_2O_7$ $[M+Na]^+$ m/z 473.2258, found 473.2264.

^{1}H NMR ($CDCl_3$, 400 MHz) δ 7.52 (d, *J*=6.87 Hz, 3H), 7.30 (t, *J*=7.79 Hz, 2H), 7.08 (t, J_1=6.87 Hz, 1H), 4.89 (dd, J_1=8.2 Hz, J_2=5.5 Hz, 1H), 3.71 (s, 3H), 2.47-2.30 (m, 2H), 2.24-2.18 (m, 1H), 2.00-1.91 (m, 1H), 1.85-1.78 (m, 2H), 1.49 (s, 18H); ^{13}C NMR ($CDCl_3$, 100 MHz) δ 171.4, 170.8, 152.4, 138.1, 129.0, 124.2, 119.9, 83.5, 57.7, 52.3, 37.0, 29.4, 28.1, 22.4.

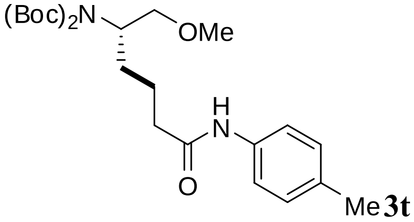

3t

(*S*)-Methyl 2-(bis(*tert*-butoxycarbonyl)amino)-6-oxo-6-(p-tolylamino)hexanoate (3t): Eluent: petroleum ether/ethyl acetate (5:1). Yield: 35 mg (75%). Yellow oil. HRMS (ESI-TOF): calculated for $C_{24}H_{36}N_2O_7$ $[M+Na]^+$ m/z 487.2415, found 487.2415.

^{1}H NMR ($CDCl_3$, 400 MHz) δ 7.39 (d, *J*=7.79 Hz, 3H), 7.10 (d, *J*=7.79 Hz, 2H), 4.89 (dd, J_1=8.2 Hz, J_2=5.5 Hz, 1H), 3.71 (s, 3H), 2.45-2.33 (m,

2H), 2.30 (s, 3H), 2.25-2.16 (m, 1H), 2.00-1.91 (m, 1H), 1.86-1.73 (m, 2H), 1.49 (s, 18H); ^{13}C NMR ($CDCl_3$, 100 MHz) δ 171.4, 170.6, 152.4, 135.6, 133.8, 129.5, 120.0, 83.4, 57.7, 52.3, 37.0, 29.5, 28.1, 22.5, 20.9.

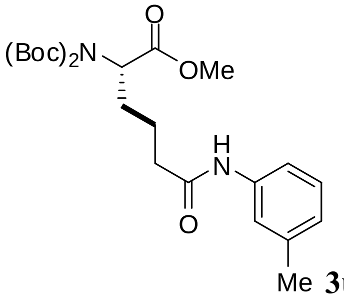

3u

(*S*)-Methyl 2-(bis(*tert*-butoxycarbonyl)amino)-6-oxo-6-(m-tolylamino)hexanoate (3u): Eluent: petroleum ether/ethyl acetate (5:1). Yield: 33.5 mg (72%). Yellow oil. HRMS (ESI-TOF): calculated for $C_{24}H_{36}N_2O_7$ $[M+Na]^+$ m/z 487.2415, found 487.2415.

^{1}H NMR ($CDCl_3$, 400 MHz) δ 7.44 (s, 1H), 7.38 (s, 1h), 7.28 (t, *J*=8.24 Hz, 1H), 7.18 (t, *J*=7.79 Hz, 1H),6.90 (d, *J*=7.33 Hz, 1H), 4.89 (dd, J_1=8.2 Hz, J_2=5.5 Hz, 1H), 3.72 (s, 3H), 2.48-2.35 (m, 2H), 2.32 (s, 3H), 2.25-2.16 (m, 1H), 2.00-1.91 (m, 1H), 1.86-1.73 (m, 2H), 1.49 (s, 18H); ^{13}C NMR ($CDCl_3$, 100 MHz) δ 171.4, 170.8, 152.4, 138.9, 138.0, 128.8, 125.0, 120.6, 117.0, 83.5, 57.6, 52.3, 37.0, 29.4, 28.1, 22.4, 21.5.

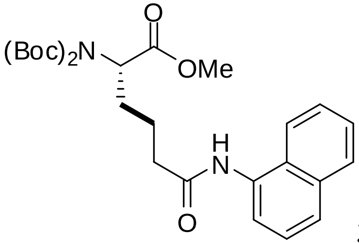

3v

(*S*)-Methyl 2-(bis(*tert*-butoxycarbonyl)amino)-6-(naphthalen-1-ylamino)-6-oxohexanoate (3v): Eluent: petroleum ether/ethyl acetate (5:1). Yield: 34.1 mg (72%). Yellow oil. HRMS (ESI-TOF): calculated for $C_{27}H_{36}N_2O_7$ $[M+Na]^+$ m/z 523.2415, found 523.2414.

^{1}H NMR ($CDCl_3$, 400 MHz) δ 7.89-7.84 (t, *J*=7.79 Hz, 3H), 7.77 (s, 1h), 7.68 (d, *J*=8.24 Hz, 1H), 7.49-7.43 (m, 3H) , 4.95 (dd, J_1=8.2 Hz, J_2=5.5 Hz, 1H), 3.72 (s, 3H), 2.60-2.49 (m, 2H), 2.32-2.22 (m, 1H), 2.205-2.00 (m, 1H), 1.94-1.86 (m, 2H), 1.49 (s, 18H); ^{13}C NMR ($CDCl_3$, 100 MHz) δ 171.4, 171.3, 152.4, 134.2, 132.5, 128.7, 127.4, 126.3, 126.0, 125.9, 125.8, 121.2, 121.0, 83.4, 57.7, 52.3, 36.9, 29.5, 28.1, 22.7.

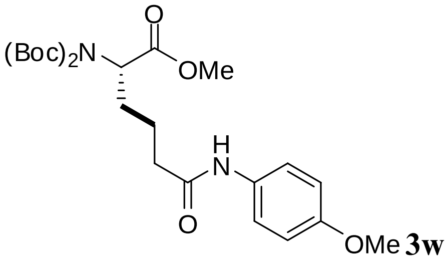

3w

(*S*)-Methyl 2-(bis(*tert*-butoxycarbonyl)amino)-6-(4-methoxyphenylamino)-6-oxohexanoate (3w): Eluent: petroleum ether/ethyl acetate (4:1). Yield: 37.5 mg (75%). Yellow oil. HRMS (ESI-TOF): calculated for $C_{24}H_{36}N_2O_8$ $[M+Na]^+$ m/z 503.2364, found 503.2366.

^{1}H NMR ($CDCl_3$, 400 MHz) δ 7.42 (d, *J*=9.16 Hz, 2H), 7.38(s, 1h), 6.84 (d, *J*=9.16 Hz, 2H), 4.89 (dd, J_1=8.2 Hz, J_2=5.5 Hz, 1H), 3.78 (s, 3H), 3.72 (s, 3H), 2.45-2.30 (m, 2H), 2.27-2.17 (m, 1H), 2.00-1.90 (m, 1H), 1.88-1.73 (m, 2H), 1.49 (s, 18H); ^{13}C NMR ($CDCl_3$, 100 MHz) δ 171.4, 170.6, 156.4, 152.3, 131.2, 121.8, 114.2, 83.5, 57.6, 55.6, 52.3, 36.9, 29.5, 28.1, 22.5.

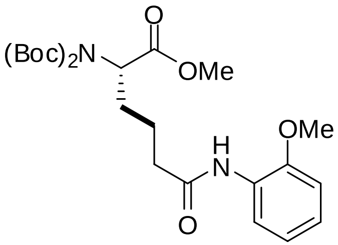

3x

(*S*)-Methyl 2-(bis(*tert*-butoxycarbonyl)amino)-6-(2-methoxyphenylamino)-6-oxohexanoate (3x): Eluent: petroleum ether/ethyl acetate (4:1). Yield: 32.8mg (68%). Yellow oil. HRMS (ESI-TOF): calculated for $C_{24}H_{36}N_2O_8$ $[M+Na]^+$ m/z 503.2364, found 503.2366.

^{1}H NMR ($CDCl_3$, 400 MHz) δ 8.35 (d, *J*=7.79 Hz, 1H), 7.76 (s, 1h), 7.02 (t, *J*=7.33 Hz, 1H), 6.94 (t, *J*=7.79 Hz, 1H), 6.86 (d, *J*=7.79 Hz, 1H), 4.89 (dd, J_1=8.2 Hz, J_2=5.5 Hz, 1H), 3.87 (s, 3H), 3.71 (s, 3H), 2.50-2.35 (m, 2H), 2.24-2.15 (m, 1H), 2.04-1.94 (m, 1H), 1.88-1.75 (m, 2H), 1.49 (s, 18H); ^{13}C NMR ($CDCl_3$, 100 MHz) δ 171.3, 170.5, 152.3, 147.8, 127.7, 123.6, 121.1, 119.9, 109.9, 83.3, 57.8, 55.7, 52.3, 37.5, 29.5, 28.1, 22.4.

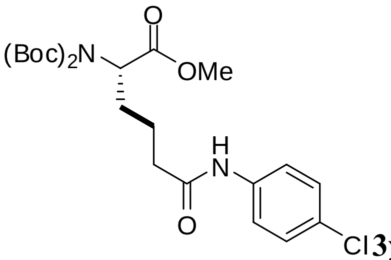

3y

(*S*)-Methyl 2-(bis(*tert*-butoxycarbonyl)amino)-6-(4-chlorophenylamino)-6-oxohexanoate (3y): Eluent: petroleum ether/ethyl acetate (5:1). Yield: 32.8mg (68%). Yellow oil. HRMS (ESI-TOF): calculated for $C_{23}H_{33}ClN_2O_2$ $[M+Na]^+$ m/z 506.1916, found 506.1913.

^{1}H NMR ($CDCl_3$, 400 MHz) δ 7.69 (s, 1h), 7.49 (d, J=8.7 Hz, 2H), 7.25 (d, J=8.7 Hz, 2H), 4.88 (dd, J_1=8.2 Hz, J_2=5.5 Hz, 1H), 3.72 (s, 3H), 2.47-2.30 (m, 2H), 2.25-2.16 (m, 1H), 1.99-1.89 (m, 1H), 1.87-1.73 (m, 2H), 1.48 (s, 18H); ^{13}C NMR ($CDCl_3$, 100 MHz) δ 171.4, 170.9, 152.4, 136.8, 129.1, 129.0, 121.1, 83.5, 57.6, 52.4, 36.9, 29.4, 28.1, 22.3

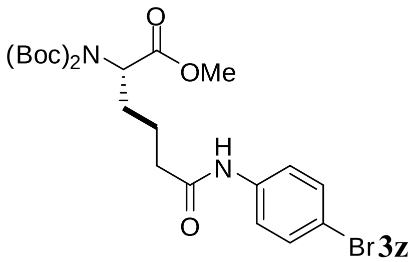

3z

(*S*)-Methyl 2-(bis(*tert*-butoxycarbonyl)amino)-6-(4-bromophenylamino)-6-oxohexanoate (3z): Eluent: petroleum ether/ethyl acetate (5:1). Yield: 37mg (70%). Yellow oil. HRMS (ESI-TOF): calculated for $C_{23}H_{33}BrN_2O_7$ $[M+Na]^+$ m/z 533.1347, found 533.1347.

^{1}H NMR ($CDCl_3$, 400 MHz) δ 7.64 (s, 1h), 7.45 (d, J=8.7 Hz, 2H), 7.40 (d, J=8.7 Hz, 2H), 4.87 (dd, J_1=8.2 Hz, J_2=5.5 Hz, 1H), 3.72 (s, 3H), 2.47-2.33 (m, 2H), 2.27-2.16 (m, 1H), 1.99-1.88 (m, 1H), 1.86-1.75 (m, 2H), 1.49 (s, 18H); ^{13}C NMR ($CDCl_3$, 100 MHz) δ171.4, 170.8, 152.4, 137.2, 131.9, 121.4, 116.7, 83.5, 57.6, 52.4, 36.9, 29.4, 28.1, 22.3

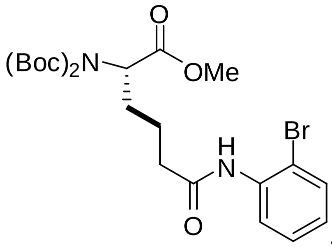

3aa

(*S*)-Methyl 2-(bis(*tert*-butoxycarbonyl)amino)-6-(2-bromophenylamino)-6-oxohexanoate (3aa): Eluent: petroleum ether/ethyl acetate (5:1). Yield: 37mg (70%). Yellow oil. HRMS (ESI-TOF): calculated for $C_{23}H_{33}BrN_2O_7$ $[M+Na]^+$ m/z 533.1347, found 533.1347.

^{1}H NMR ($CDCl_3$, 400 MHz) δ 8.31 (d, *J*=8.09 Hz, 1H), 7.63 (s, 1H), 7.52 (d, *J*=8.24 Hz, 1H), 7.30 (t, *J*=8.24 Hz, 1H), 6.96 (t, *J*=7.79 Hz, 1H) , 4.91 (dd, J_1=8.2 Hz, J_2=5.5 Hz, 1H), 3.71 (s, 3H), 2.55-2.40 (m, 2H), 2.26-2.17 (m, 1H), 2.06-1.96 (m, 1H), 1.90-1.76 (m, 2H), 1.49 (s, 18H); ^{13}C NMR ($CDCl_3$, 100 MHz) δ 171.2, 170.7, 152.3, 135.7, 132.3, 128.4, 125.2, 122.2, 113.5, 83.3, 57.7, 52.3, 37.3, 29.4, 28.1, 22.2.

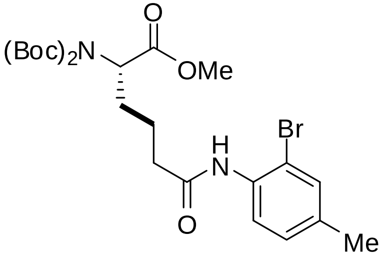

3ab

(*S*)-Methyl 2-(bis(*tert*-butoxycarbonyl)amino)-6-(2-bromo-4-methylphenylamino)-6-oxohexanoate (3ab): Eluent: petroleum ether/ethyl acetate (5:1). Yield: 37mg (68%). Yellow oil. HRMS (ESI-TOF): calculated for $C_{24}H_{35}BrN_2O_7$ $[M+Na]^+$ m/z 567.1503, found 567.1501.

^{1}H NMR ($CDCl_3$, 400 MHz) δ 8.11 (d, *J*=8.24 Hz, 1H), 7.51 (s, 1H), 7.32 (s, 1H), 7.30 (t, *J*=8.24 Hz, 1H), 7.07 (t, *J*=8.24 Hz, 1H) , 4.89 (dd, J_1=8.2 Hz, J_2=5.5 Hz, 1H), 3.69 (s, 3H), 2.50-2.35 (m, 2H), 2.26 (s, 3H), 2.23-2.15 (m, 1H), 2.03-1.93 (m, 1H), 1.83-1.74 (m, 2H), 1.47 (s, 18H); ^{13}C NMR ($CDCl_3$, 100 MHz) δ 171.2, 170.6, 152.3, 135.3, 133.2, 132.5, 129.0, 122.1, 113.5, 83.3, 57.7, 52.3, 37.3, 29.4, 28.1, 22.3, 20.6.

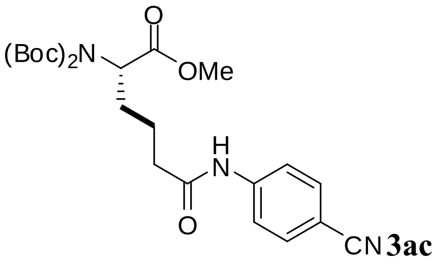
3ac

(*S*)-Methyl 2-(bis(*tert*-butoxycarbonyl)amino)-6-(4-cyanophenylamino)-6-oxohexanoate (3ac): Eluent: petroleum ether/ethyl acetate (3:1). Yield: 31mg (65%). Yellow oil. HRMS (ESI-TOF): calculated for $C_{24}H_{33}N_3O_7$ $[M+Na]^+$ m/z 498.2211, found 498.2212.

^1H NMR ($CDCl_3$, 400 MHz) δ 8.10 (s, 1H), 7.71 (d, *J*=8.70 Hz, 2H), 7.58 (d, *J*=8.70 Hz, 2H), 4.87 (dd, J_1=8.2 Hz, J_2=5.5 Hz, 1H), 3.73 (s, 3H), 2.52-2.38 (m, 2H), 2.27-2.17 (m, 1H), 1.98-1.91 (m, 1H), 1.89-1.74 (m, 2H), 1.49 (s, 18H); ^{13}C NMR ($CDCl_3$, 100 MHz) δ 171.5, 171.3, 152.4, 142.4, 133.3, 119.6, 119.0, 106.8, 83.7, 57.6, 52.4, 36.9, 29.4, 28.1, 22.2.

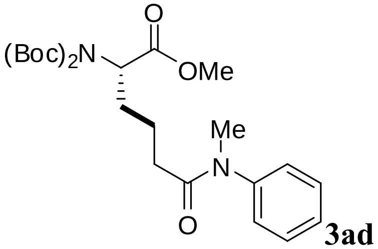
3ad

(*S*)-Methyl 2-(bis(*tert*-butoxycarbonyl)amino)-6-(methyl(phenyl)amino)-6-oxohexanoate (3ad): Eluent: petroleum ether/ethyl acetate (6:1). Yield: 30mg (65%). Yellow oil. HRMS (ESI-TOF): calculated for $C_{24}H_{36}N_2O_7$ $[M+Na]^+$ m/z 487.2415, found 487.2416.

^1H NMR ($CDCl_3$, 400 MHz) δ 7.41 (t, *J*=7.33 Hz , 2H), 7.32 (t, *J*=7.33 Hz, 1H), 4.78 (dd, J_1=8.2 Hz, J_2=5.5 Hz, 1H), 3.67 (s, 3H), 3.25 (s, 3H),

2.13-2.06(m, 2H), 2.00-1.94 (m, 1H), 1.84-1.75 (m, 1H), 1.66-1.61 (m, 2H), 1.47 (s, 18H); ^{13}C NMR ($CDCl_3$, 100 MHz) δ 172.6, 171.3, 152.2, 144.2, 129.9, 127.7, 127.4, 83.1, 58.0, 52.2, 37.3, 33.8, 29.6, 28.1, 22.2.

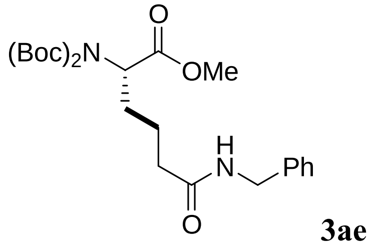

3ae

(*S*)-Methyl 6-(benzylamino)-2-(bis(*tert*-butoxycarbonyl)amino)-6-oxohexanoate (3ae): Eluent: petroleum ether/ethyl acetate (5:1). Yield: 27.8 mg (62%). Yellow oil. HRMS (ESI-TOF): calculated for $C_{24}H_{36}N_2O_7$ $[M+Na]^+$ m/z 487.2415, found 487.2416.

^{1}H NMR ($CDCl_3$, 400 MHz) δ 7.32 (t, *J*=5.5 Hz , 2H), 7.27-7.24 (m, 3H), 5.93 (s, 1H) 4.86 (dd, J_1=8.2 Hz, J_2=5.5 Hz, 1H), 4.43 (dd, J_1=5.5 Hz, J_2=3.21 Hz, 2H), 3.70 (s, 3H), 2.33-2.21(m, 2H), 2.20-2.13 (m, 1H), 1.97-1.87 (m, 1H), 1.83-1.67 (m, 2H), 1.48 (s, 18H); ^{13}C NMR ($CDCl_3$, 100 MHz) δ 172.4, 171.3, 152.3, 138.4, 128.8, 127.9, 127.5, 83.4, 57.6, 52.3, 43.7, 36.1, 29.5, 28.0, 22.5.

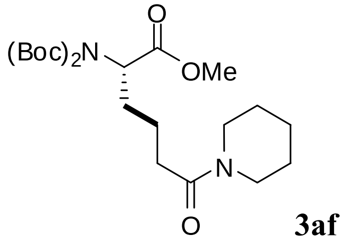

3af

(*S*)-Methyl 2-(bis(*tert*-butoxycarbonyl)amino)-6-oxo-6-(piperidin-1-yl)hexanoate (3af): Eluent: petroleum ether/ethyl acetate (5:1). Yield: 26.6 mg (60%). Yellow oil. HRMS (ESI-TOF): calculated for $C_{22}H_{38}N_2O_7$ $[M+Na]^+$ m/z 465.2571, found 465.2575.

^{1}H NMR ($CDCl_3$, 400 MHz) δ 4.88 (dd, J_1=8.2 Hz, J_2=5.5 Hz, 1H), 3.70 (s, 3H), 3.55 (t, J=5.5 Hz, 2H), 3.3 (t, J=5.5 Hz, 2H), 2.48-2.3 (m, 2H), 2.20-2.11 (m, 1H), 2.00-1.90 (m,1H), 1.77-1.68 (m,2H), 1.66-1.60 (m, 2H), 1.58-1.51 (m, 4H), 1.49 (s, 18H); ^{13}C NMR ($CDCl_3$, 100 MHz) δ 171.4, 152.2, 83.2, 57.9, 52.2, 46.9, 42.9, 33.0, 29.7, 28.1, 26.6, 25.6, 24.6, 22.2.

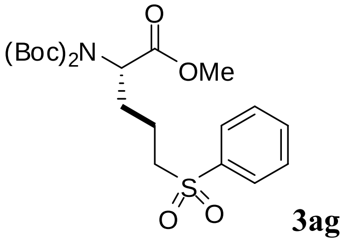

3ag

(*S*)-Methyl 2-(bis(*tert*-butoxycarbonyl)amino)-5-(phenylsulfonyl)pentanoate (3ag): Eluent: petroleum ether/ethyl acetate (8:1). Yield: 34 mg (72%). Yellow oil. HRMS (ESI-TOF): calculated for $C_{22}H_{33}NO_8S$ $[M+Na]^+$ m/z 494.1819, found 494.1814.

^{1}H NMR ($CDCl_3$, 400 MHz) δ 7.89 (d, J=7.79 Hz, 2H), 7.65 (t, J=7.33 Hz, 1H), 7.56 (t, J=7.33 Hz, 2H), 4.80 (dd, J_1=9.16 Hz, J_2=5.04 Hz, 1H), 3.69 (s, 3H), 3.22-3.04 (m, 2H), 2.20-2.13 (m, 1H), 2.04-1.95 (m, 1H), 1.84-1.72 (m, 2H), 1.48 (s, 18H); ^{13}C NMR ($CDCl_3$, 100 MHz) δ 170.8, 152.1, 139.1, 133.8, 129.4, 128.1, 83.6, 57.4, 55.8, 52.4, 28.8, 28.0, 19.8.

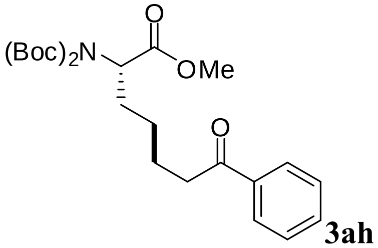

3ah

(*S*)-Methyl 2-(bis(*tert*-butoxycarbonyl)amino)-7-oxo-7-phenylheptanoate (3ah): Eluent: petroleum ether/ethyl acetate (10:1). Yield: 32.5 mg

(72%). Colorless oil. HRMS (ESI-TOF): calculated for $C_{24}H_{35}NO_7$ $[M+Na]^+$ m/z 472.2306, found 472.2304.

^{1}H NMR ($CDCl_3$, 400 MHz) δ 7.94 (d, *J* = 8.24 Hz, 2H), 7.55 (t, *J* =7.33 Hz, 1H), 7.45 (t, *J* =7.33 Hz, 2H), 4.87 (dd, J_1=9.39Hz, J_2=5.50 Hz, 1H), 3.71 (s, 3H), 2.97 (t, *J* =7.33 Hz, 2H), 2.22-2.13 (m, 1H), 1.98-1.87 (m, 1H), 1.84-1.72 (m, 2H), 1.49 (s, 18H), 1.47-1.41 (m, 2H); ^{13}C NMR ($CDCl_3$, 100 MHz) δ 200.1, 171.4, 152.2, 137.1, 133.0, 128.6, 128.1, 83.2, 58.1, 52.1, 38.4, 29.9, 28.1, 26.1, 24.0.

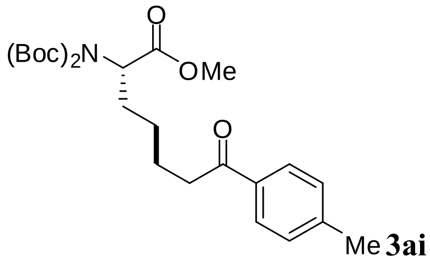

3ai

(*S*)-Methyl 2-(bis(*tert*-butoxycarbonyl)amino)-7-oxo-7-*p*-tolylheptanoate (3ai): Eluent: petroleum ether/ethyl acetate (10:1). Yield: 34.8 mg (75%). Colorless oil. HRMS (ESI-TOF): calculated for $C_{24}H_{35}NO_7$ $[M+Na]^+$ m/z 486.2462, found 486.2462.

^{1}H NMR ($CDCl_3$, 400 MHz) δ 7.84 (d, *J* = 8.24 Hz, 2H), 7.24 (d, *J* =8.24 Hz, 2H), 4.86 (dd, J_1=9.39Hz, J_2=5.50 Hz, 1H), 3.70 (s, 3H), 2.94 (t, *J* =7.33 Hz, 2H), 2.4 (s, 3H), 2.21-2.12 (m, 1H), 1.96-1.88 (m, 1H), 1.83-1.72 (m, 2H), 1.49 (s, 18H), 1.46-1.39 (m, 2H); ^{13}C NMR ($CDCl_3$, 100 MHz) δ 199.8, 171.4, 152.2, 143.7, 134.6, 129.3, 128.2, 83.1, 58.1, 52.2, 38.3, 29.9, 28.1, 26.1, 24.1, 21.7.

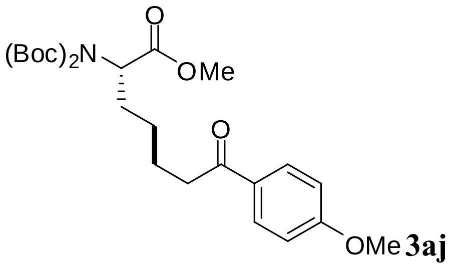

3aj

(*S*)-Methyl 2-(bis(*tert*-butoxycarbonyl)amino)-7-(4-methoxyphenyl)-7-oxoheptanoate (3aj): Eluent: petroleum ether/ethyl acetate (8:1). Yield: 36.5 mg (76%). Colorless oil. HRMS (ESI-TOF): calculated for $C_{25}H_{37}NO_8$ $[M+Na]^+$ m/z 502.2411, found 502.2410.

^{1}H NMR ($CDCl_3$, 400 MHz) δ 7.93 (d, *J* = 9.16 Hz, 2H), 6.92 (d, *J* = 9.16 Hz, 2H), 4.86 (dd, J_1= 9.39Hz, J_2=5.50 Hz, 1H), 3.86 (s, 3H), 3.71 (s, 3H), 2.92 (t, *J* =7.33 Hz, 2H), 2.21-2.10 (m, 1H), 1.97-1.87 (m, 1H), 1.83-1.69 (m, 2H), 1.49 (s, 18H), 1.45-1.30 (m, 2H); ^{13}C NMR ($CDCl_3$, 100 MHz) δ 198.7, 171.4, 163.4, 152.2, 130.3, 130.1, 113.7, 83.1, 58.0, 55.5, 52.2, 38.0, 29.8, 28.0, 26.1, 24.2.

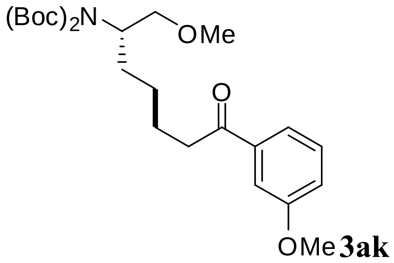

3ak

(*S*)-Methyl 2-(bis(*tert*-butoxycarbonyl)amino)-7-(3-methoxyphenyl)-7-oxoheptanoate (3ak): Eluent: petroleum ether/ethyl acetate (8:1). Yield: 36.0 mg (75%). Colorless oil. HRMS (ESI-TOF): calculated for $C_{25}H_{37}NO_8$ $[M+Na]^+$ m/z 502.2411, found 502.2410.

^{1}H NMR ($CDCl_3$, 400 MHz) δ 7.52 (d, *J* = 7.33 Hz, 1H), 7.47 (s, 1H), 7.35 (t, *J* = 7.79 Hz, 1H), 7.10 (d, *J* = 8.24 Hz, 1H), 4.87 (dd, J_1= 9.39Hz,

J_2=5.50 Hz, 1H), 3.85 (s, 3H), 3.71 (s, 3H), 2.95 (t, J =7.33 Hz, 2H), 2.22-2.13 (m,1H), 1.97-1.86 (m, 1H), 1.84-1.70 (m, 2H), 1.49 (s, 18H), 1.45-1.40 (m, 2H); ^{13}C NMR ($CDCl_3$, 100 MHz) δ 199.9, 171.4, 159.9, 152.2, 138.5, 129.6, 120.8, 119.4, 112.4, 83.1, 58.1, 55.5, 52.2, 38.5, 29.9, 28.0, 26.1, 24.1.

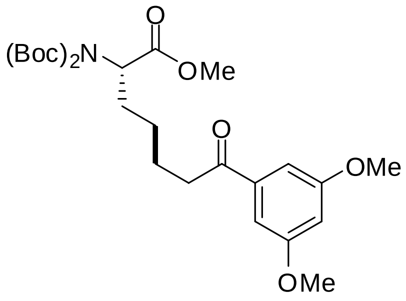

3al

(*S*)-Methyl 2-(bis(*tert*-butoxycarbonyl)amino)-7-(3,5-dimethoxyphenyl)-7-oxoheptanoate (3al): Eluent: petroleum ether/ethyl acetate (7:1). Yield: 35.5 mg (70%). Light yellow oil. HRMS (ESI-TOF): calculated for $C_{26}H_{39}NO_9$ $[M+Na]^+$ m/z 532.2517, found 532.2512.

^{1}H NMR ($CDCl_3$, 400 MHz) δ 7.08 (d, J = 2.29 Hz, 2H), 6.63 (t, J = 2.29 Hz, 1H), 4.87 (dd, J_1= 9.39Hz, J_2=5.50 Hz, 1H), 3.83 (s, 6H), 3.71 (s, 3H), 2.92 (t, J =7.33 Hz, 2H), 2.21-2.12 (m, 1H), 1.96-1.87 (m, 1H), 1.81-1.70 (m, 2H), 1.49 (s, 18H), 1.47-1.39 (m, 2H); ^{13}C NMR ($CDCl_3$, 100 MHz) δ 199.8, 171.4, 161.1, 152.2, 139.1, 106.0, 105.3, 83.2, 58.1, 55.7, 52.2, 38.5, 29.9, 28.1, 26.1, 24.1.

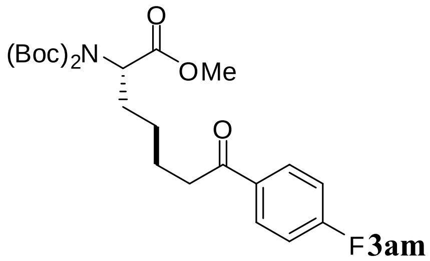

3am

(*S*)-Methyl 2-(bis(*tert*-butoxycarbonyl)amino)-7-(4-fluorophenyl)-7-oxoheptanoate (3am): Eluent: petroleum ether/ethyl acetate (10:1). Yield: 31.9 mg (68%). Colorless oil. HRMS (ESI-TOF): calculated for $C_{24}H_{34}FNO_7$ $[M+Na]^+$ m/z 490.2212, found 490.2214.

^{1}H NMR ($CDCl_3$, 400 MHz) δ 7.97 (dd, J_1= 8.59 Hz, J_2=5.50 Hz, 2H), 7.12 (t, J = 8.59 Hz, 2H), 4.87 (dd, J_1= 9.39Hz, J_2=5.50 Hz, 1H), 3.71 (s, 3H), 2.94 (t, J =7.33 Hz, 2H), 2.20-2.14 (m, 1H), 1.95-1.88 (m, 1H), 1.83-1.71 (m, 2H), 1.49 (s, 18H), 1.47-1.40 (m, 2H); ^{13}C NMR ($CDCl_3$, 100 MHz) δ 198.5, 171.4, 166.6, 164.9, 152.2, 133.5,130.7, 115.8, 115.6, 83.2, 58.0, 52.2, 38.3, 29.8, 28.2, 26.0, 24.0.

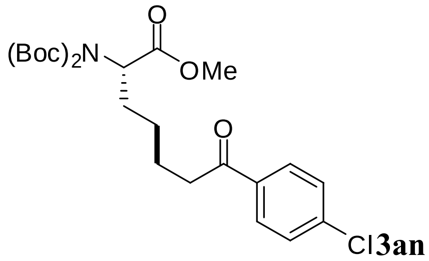

3an

(*S*)-Methyl 2-(bis(*tert*-butoxycarbonyl)amino)-7-(4-chlorophenyl)-7-oxoheptanoate (3an): Eluent: petroleum ether/ethyl acetate (10:1). Yield: 33 mg (68%). Colorless oil. HRMS (ESI-TOF): calculated for $C_{24}H_{34}ClNO_7$ $[M+Na]^+$ m/z 506.1916, found 506.1913.

^{1}H NMR ($CDCl_3$, 400 MHz) δ 7.88 (d, J=8.7 Hz, 2H), 7.42 (d, J = 8.7 Hz, 2H), 4.87 (dd, J_1= 9.39 Hz, J_2=5.50 Hz, 1H), 3.71 (s, 3H), 2.94 (t, J

=7.33 Hz, 2H), 2.27-2.14 (m, 1H), 1.96-1.86 (m, 1H), 1.83-1.71 (m, 2H), 1.49 (s, 18H), 1.46-1.40 (m, 2H); ^{13}C NMR ($CDCl_3$, 100 MHz) δ 198.9, 171.4, 152.2, 139.4, 135.4,129.5, 129.0, 83.2, 58.0, 52.2, 38.4, 29.8, 28.1, 26.0, 23.9.

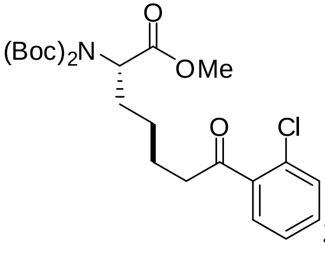

3ao

(*S*)-Methyl 2-(bis(*tert*-butoxycarbonyl)amino)-7-(2-chlorophenyl)-7-oxoheptanoate (3ao): Eluent: petroleum ether/ethyl acetate (10:1). Yield: 30 mg (67%). Colorless oil. HR-MS:

^{1}H NMR ($CDCl_3$, 400 MHz) δ 7.42-7.28 (m, 4H), 4.86 (dd, J_1= 9.39Hz, J_2=5.50 Hz, 1H), 3.70 (s, 3H), 2.93 (t, J =7.33 Hz, 2H), 2.19-2.10 (m,1H), 1.95-1.86 (m, 1H), 1.79-1.67 (m, 2H), 1.49 (s, 18H), 1.45-1.38 (m, 2H); ^{13}C NMR ($CDCl_3$, 100 MHz) δ 203.4, 171.4, 152.2, 139.8, 131.5,130.8, 130.5, 128.7, 127.0, 83.1, 58.0, 52.2, 42.8, 29.8, 28.0, 25.9, 23.9.

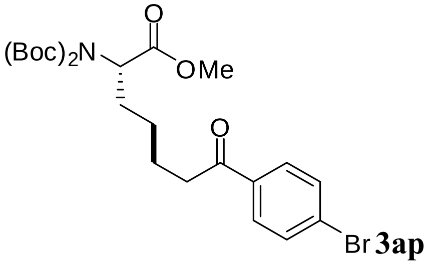

3ap

(*S*)-Methyl 2-(bis(*tert*-butoxycarbonyl)amino)-7-(4-bromophenyl)-7-oxoheptanoate (3ap): Eluent: petroleum ether/ethyl acetate (10:1).

Yield: 37 mg (70%): Light yellow oil. HRMS (ESI-TOF): calculated for $C_{24}H_{34}BrNO_7$ $[M+Na]^+$ m/z 568.1344, found 568.1344.

^1H NMR ($CDCl_3$, 400 MHz) δ 7.42-7.28 (m, 4H), 4.86 (dd, J_1 = 9.39Hz, J_2 =5.50 Hz, 1H), 3.70 (s, 3H), 2.93 (t, J = 7.33 Hz, 2H), 2.19-2.10 (m,1H), 1.95-1.86 (m, 1H), 1.79-1.67 (m, 2H), 1.49 (s, 18H), 1.45-1.38 (m, 2H); ^{13}C NMR ($CDCl_3$, 100 MHz) δ 199.9, 171.4, 152.2, 135.7, 132.0,129.7, 128.1, 83.2, 58.0, 52.2, 38.4, 29.8, 28.1, 26.0, 23.9.

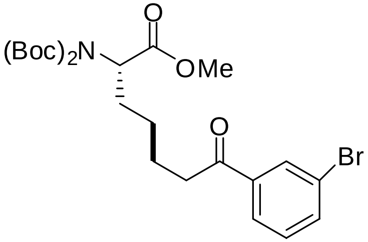

(3aq)

(*S*)-Methyl 2-(bis(*tert*-butoxycarbonyl)amino)-7-(3-bromophenyl)-7-oxoheptanoate (3aq): Eluent: petroleum ether/ethyl acetate (10:1). Yield: 37 mg (70%): Light yellow oil. HRMS (ESI-TOF): calculated for $C_{24}H_{34}BrNO_7$ $[M+Na]^+$ m/z 568.1344, found 568.1344.

^1H NMR ($CDCl_3$, 400 MHz) δ 7.80 (d, J = 8.7 Hz, 2H), 7.59 (d, J = 8.7 Hz, 2H), 4.86 (dd, J_1 = 9.39 Hz, J_2 = 5.50 Hz, 1H), 3.71 (s, 3H), 2.93 (t, J = 7.33 Hz, 2H), 2.17-2.12 (m,1H), 1.96-1.86 (m, 1H), 1.83-1.71 (m, 2H), 1.49 (s, 18H), 1.47-1.44 (m, 2H); ^{13}C NMR ($CDCl_3$, 100 MHz) δ 198.6, 171.4, 152.3, 138.9, 135.9, 131.2, 130.3, 126.6, 123.1, 83.2, 58.0, 52.2, 38.5, 29.8, 28.1, 26.0, 23.8.

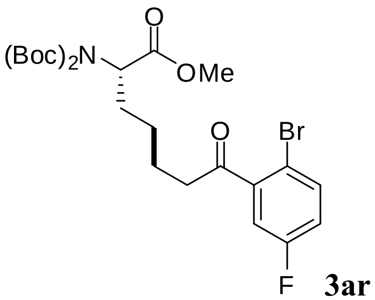

3ar

(*S*)-Methyl 2-(bis(*tert*-butoxycarbonyl)amino)-7-(2-bromo-5-fluorophenyl)-7-oxoheptanoate (3ar): Eluent: petroleum ether/ethyl acetate (10:1). Yield: 35.5 mg (65%): Light yellow oil. HRMS (ESI-TOF): calculated for $C_{24}H_{34}FBrNO_7$ $[M+Na]^+$ m/z 570.1300, found 570.1304.

^{1}H NMR ($CDCl_3$, 400 MHz) δ 7.55 (dd, J_1 = 8.7 Hz, J_2 =5.04 Hz, 1H), 7.09-6.98 (m, 2H), 4.86 (dd, J_1 = 9.39Hz, J_2 = 5.50 Hz, 1H), 3.71 (s, 3H), 2.90 (t, J = 7.33 Hz, 2H), 2.20-2.11 (m, 1H), 1.95-1.86 (m, 1H), 1.80-1.69 (m, 2H), 1.49 (s, 18H), 1.46-1.41 (m, 2H); ^{13}C NMR ($CDCl_3$, 100 MHz) δ 202.8, 171.4, 162.6, 160.9, 152.2, 143.5, 135.28, 135.23, 118.8, 118.6, 115.7, 115.5, 112.8, 112.7, 83.2, 58.0, 52.2, 42.4, 29.8, 28.1, 25.9, 23.7.

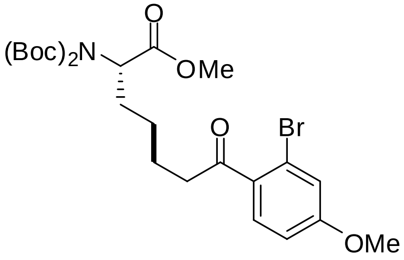

3as

(*S*)-Methyl 2-(bis(*tert*-butoxycarbonyl)amino)-7-(2-bromo-4-methoxyphenyl)-7-oxoheptanoate (3as): Eluent: petroleum ether/ethyl acetate

(8:1). Yield: 35.5 mg (65%): Light yellow oil. HRMS (ESI-TOF): calculated for $C_{25}H_{36}BrNO_8$ $[M+Na]^+$ m/z 582.1500, found 582.1504.

^{1}H NMR ($CDCl_3$, 400 MHz) δ 7.45 (d, J = 8.7 Hz, 1H), 7.13 (d, J = 2.29 Hz, 1H), 6.86 (dd, J_1 = 8.7 Hz, J_2 = 2.29 Hz, 1H), 4.86 (dd, J_1 = 9.39 Hz, J_2 = 5.50 Hz, 1H), 3.83 (s, 3H), 3.70 (s, 3H), 2.92 (t, J =7.33 Hz, 2H), 2.19-2.10 (m, 1H), 1.95-1.85 (m, 1H), 1.78-1.68 (m, 2H), 1.49 (s, 18H), 1.45-1.39 (m, 2H); ^{13}C NMR ($CDCl_3$, 100 MHz) δ 202.2, 171.4, 161.6, 152.2, 133.4, 130.7, 120.7, 119.4, 113.2, 83.1, 58.1, 55.7, 52.2, 42.0, 29.8, 28.1, 26.0, 24.2.

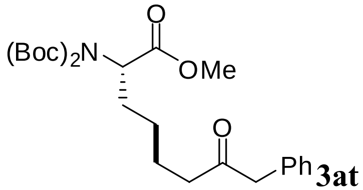

3at

(*S*)-Methyl 2-(bis(*tert*-butoxycarbonyl)amino)-7-oxo-8-phenyloctanoate (3at): Eluent: petroleum ether/ethyl acetate (10:1). Yield: 31.6 mg (68%): Colorless oil. HRMS (ESI-TOF): calculated for $C_{25}H_{37}NO_7$ $[M+Na]^+$ m/z 486.2462, found 486.2462.

^{1}H NMR ($CDCl_3$, 400 MHz) δ 7.31 (t, J= 6.87 Hz, 2H), 7.25 (t, J= 7.33 Hz, 1H), 7.18 (d, J= 6.87 Hz, 2H), 4.81 (dd, J_1= 9.39Hz, J_2=5.50 Hz, 1H), 3.69 (s, 3H), 3.66 (s, 2H), 2.44 (t, J =7.33 Hz, 2H), 2.11-2.02 (m, 1H), 1.87-1.78 (m, 1H), 1.66-1.51 (m, 2H), 1.48 (s, 18H), 1.35-1.25 (m, 2H); ^{13}C NMR ($CDCl_3$, 100 MHz) δ 208.1, 171.4, 152.2, 134.3, 129.4, 128.8, 127.0, 83.1, 58.0, 50.2, 41.8, 29.7, 28.0, 25.8, 23.4.

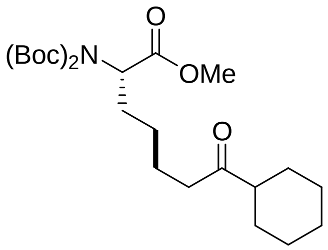

3au

(*S*)-Methyl 2-(bis(*tert*-butoxycarbonyl)amino)-8-cyclohexyl-7-oxooctanoate (3au): Eluent: petroleum ether/ethyl acetate (10:1). Yield: 30.6 mg (65%): Colorless oil. HRMS (ESI-TOF): calculated for $C_{25}H_{43}NO_7$ $[M+Na]^+$ m/z 478.2775, found 478.2778.

^{1}H NMR ($CDCl_3$, 400 MHz) δ4.82 (dd, J_1= 8.13 Hz, J_2=5.04 Hz, 1H), 3.70 (s, 3H), 2.42 (t, J =7.33 Hz, 2H), 2.34-2.27 (m, 1H), 2.15-2.04 (m, 1H), 1.91-1.84 (m, 1H), 1.81-1.76 (m, 4H), 1.67-1.53 (m, 2H), 1.49 (s, 18H), 1.37-1.21 (m, 8H); ^{13}C NMR ($CDCl_3$, 100 MHz) δ 214.0, 171.5, 152.2, 83.1, 58.1, 52.2, 50.9, 40.4, 29.8, 28.6, 28.0, 26.0, 25.9, 25.7, 23.4.

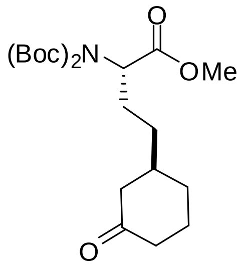

3av

(*S*)-Methyl 2-(bis(*tert*-butoxycarbonyl)amino)-4-(3-oxocyclohexyl)butanoate (3av): Eluent: petroleum ether/ethyl acetate (10:1). Yield: 25 mg (60%): Colorless oil. Diastereomeric ratio of δ–C (1:1). HRMS (ESI-TOF): calculated for $C_{21}H_{35}NO_7$ $[M+Na]^+$ m/z 436.2306, found 436.2306.

^{1}H NMR ($CDCl_3$, 400 MHz) δ 4.84 (dd, J_1= 9.39 Hz, J_2=5.50 Hz, 1H), 3.71 (s, 3H), 2.41 (t, J =7.33 Hz, 2H), 2.28-2.20 (m, 1H), 2.18-2.10 (m, 1H), 2.03 (t, J =11.91 Hz, 2H), 1.96-1.85 (m, 2H), 1.79 (m, 1H), 1.65-1.59 (m, 2H), 1.49 (s, 18H), 1.44-1.31 (m, 2H); ^{13}C NMR ($CDCl_3$, 100 MHz) δ 211.6, 171.3, 152.2, 83.3, 58.1, 52.3, 48.2, 41.5, 38.9, 33.3, 31.3, 28.1, 27.2, 25.3.

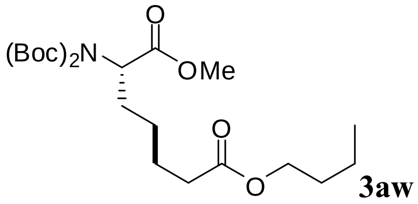

3aw

(*S*)-7-Butyl 1-methyl 2-(bis(*tert*-butoxycarbonyl)amino)heptanedioate (3aw): Eluent: petroleum ether/ethyl acetate (10:1). Yield: 29 mg (65%): Colorless oil. HRMS (ESI-TOF): calculated for $C_{22}H_{39}NO_8$ $[M+Na]^+$ m/z 468.2568, found 468.2571.

^{1}H NMR ($CDCl_3$, 400 MHz) δ 4.85 (dd, J_1= 9.39 Hz, J_2=5.50 Hz, 1H), 4.06 (t, J =6.87 Hz, 2H), 3.71 (s, 3H), 2.41-2.35 (m, 1H), 2.30 (t, J =7.68 Hz, 2H), 2.17-2.03 (m, 1H), 1.96-1.77 (m, 2H), 1.71-1.61 (m, 2H), 1.60-1.53 (m, 2H), 1.49 (s, 18H), 1.45-1.32 (m, 4H), 0.93 (t, J = 7.33 Hz, 3H); ^{13}C NMR ($CDCl_3$, 100 MHz) δ 173.7, 171.3, 152.2, 83.2, 64.3, 58.0, 52.2, 44.9, 34.2, 32.1, 30.8, 29.7, 28.1, 25.9, 24.8, 19.2, 13.8.

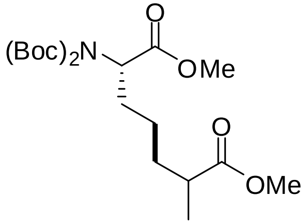

3ax

(S)-Dimethyl 2-(bis(*tert*-butoxycarbonyl)amino)-6-methylheptanedioate (3ax): Eluent: petroleum ether/ethyl acetate (10:1). Yield: 25 mg

(60%): Colorless oil. Diastereomeric ratio of ε–C (1:1). HRMS (ESI-TOF): calculated for $C_{20}H_{35}NO_8$ $[M+Na]^+$ m/z 440.2255, found 440.2254.

^{1}H NMR ($CDCl_3$, 400 MHz) δ 4.86-4.81 (m, 1H), 3.70 (s, 3H), 3.65 (s, 3H), 2.49-2.41 (m, 1H), 2.14-2.05 (m, 1H), 1.92-1.85 (m, 1H), 1.76-1.68 (m, 1H), 1.49 (s, 18H), 1.46-1.41 (m, 1H), 1.39-1.33 (m, 2H), 1.14 (s, 3H); ^{13}C NMR ($CDCl_3$, 100 MHz) δ 177.1, 171.4, 152.1, 83.2, 58.1, 52.2, 51.6, 39.3, 33.4, 29.9, 28.1, 24.0, 17.1.

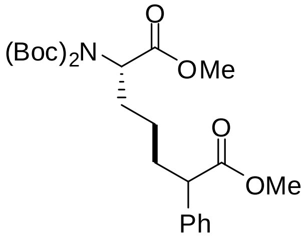

3ay

(*S*)-Dimethyl 2-(bis(*tert*-butoxycarbonyl)amino)-6-phenylheptanedioate (3ay): Eluent: petroleum ether/ethyl acetate (10:1). Yield: 33.6 mg (70%): Light yellow oil. Diastereomeric ratio of ε–C (1:1). HRMS (ESI-TOF): calculated for $C_{20}H_{35}NO_8$ $[M+Na]^+$ m/z 502.2411, found 502.2411.

^{1}H NMR ($CDCl_3$, 400 MHz) δ 7.36-7.22 (m, 5H), 4.80 (dd, J_1= 9.62 Hz, J_2=5.04 Hz, 1H), 3.69 (s, 3H), 3.63 (s, 3H), 3.53 (t, J= 7.79 Hz, 1H), 2.18-2.02 (m, 2H), 1.96-1.73 (m, 2H), 1.46 (s, 18H), 1.33-1.23 (m, 2H); ^{13}C NMR ($CDCl_3$, 100 MHz) δ 174.4, 171.3, 152.1, 139.0, 128.7, 127.9, 127.3, 83.1, 58.0, 52.2, 52.0, 51.4, 33.2, 29.8, 28.0, 24.3.

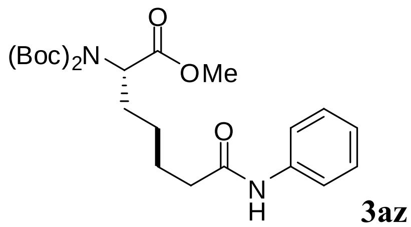

3az

(*S*)-Methyl 2-(bis(*tert*-butoxycarbonyl)amino)-7-oxo-7-(phenylamino)heptanoate (3az): Eluent: petroleum ether/ethyl acetate (5:1). Yield: 32.5 mg (70%). Yellow oil. HRMS (ESI-TOF): calculated for $C_{24}H_{36}N_2O_7$ $[M+Na]^+$ m/z 487.2415, found 487.2419.

^{1}H NMR ($CDCl_3$, 400 MHz) δ 7.52 (d, *J*= 7.79 Hz, 2H), 7.41 (s, 1H), 7.30 (t, *J*= 7.79 Hz, 2H), 7.08 (t, *J*=7.56 Hz, 2H), 4.85 (dd, J_1=8.7 Hz, J_2=5.5 Hz, 1H), 3.71 (s, 3H), 2.35 (t, *J*=7.79 Hz, 2H), 2.21-2.14 (m, 1H), 1.93-1.85 (m,1H), 1.81-1.74 (m, 2H), 1.48 (s, 18H), 1.46-1.44 (m, 2H); ^{13}C NMR ($CDCl_3$, 100 MHz) δ 171.4, 171.1, 152.2, 135.5, 133.7, 129.5, 120.0, 83.3, 57.9, 52.2, 37.4, 29.6, 28.0, 25.9, 25.3, 20.9.

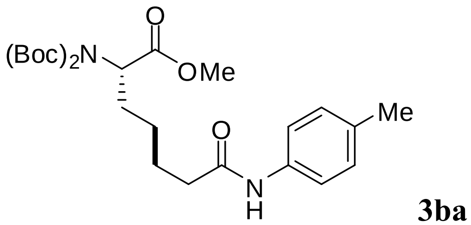

3ba

(*S*)-Methyl 2-(bis(*tert*-butoxycarbonyl)amino)-7-oxo-7-(*p*-tolylamino)heptanoate (3ba): Eluent: petroleum ether/ethyl acetate (5:1). Yield: 34.5 mg (72%). Yellow oil. HRMS (ESI-TOF): calculated for $C_{25}H_{38}N_2O_7$ $[M+Na]^+$ m/z 501.2571, found 501.2571.

^{1}H NMR ($CDCl_3$, 400 MHz) δ 7.39 (d, *J*= 7.24 Hz, 2H), 7.35 (s, 1H), 7.10 (d, *J*= 7.24 Hz, 2H), 4.85 (dd, J_1=8.7 Hz, J_2=5.5 Hz, 1H), 3.71 (s, 3H), 2.34 (t, *J*=7.79 Hz, 2H), 2.30 (s, 3H), 2.20-2.13 (m, 1H), 1.93-1.85 (m, 1H), 1.78-1.73 (m, 2H), 1.48 (s, 18H), 1.45-1.44 (m, 2H); ^{13}C NMR

($CDCl_3$, 100 MHz) δ 171.5, 171.1, 152.2, 135.5, 133.8, 129.5, 120.0, 83.3, 57.9, 52.3, 37.4, 29.6, 28.0, 25.9, 25.3, 20.9.

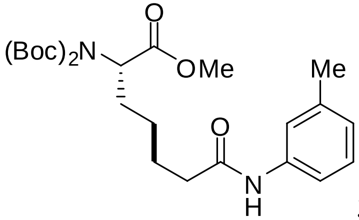

3bb

(*S*)-Methyl 2-(bis(*tert*-butoxycarbonyl)amino)-7-oxo-7-(m-tolylamino)heptanoate (3bb): Eluent: petroleum ether/ethyl acetate (5:1). Yield: 32.5 mg (68%). Yellow oil. HRMS (ESI-TOF): calculated for $C_{25}H_{38}N_2O_7$ $[M+Na]^+$ m/z 501.2571, found 501.2571.

^1H NMR ($CDCl_3$, 400 MHz) δ 7.38 (s, 1H), 7.33 (s, 1H), (d, *J*= 8.24 Hz, 1H), 7.18 (t, *J*= 7.56 Hz, 1H), 6.90 (d, *J*= 7.33 Hz, 1H), 4.85 (dd, J_1=8.7 Hz, J_2=5.5 Hz, 1H), 3.71 (s, 3H), 2.34 (t, *J*=7.79 Hz, 2H), 2.32 (s, 3H), 2.23-2.14 (m, 1H), 1.93-1.83 (m,1H), 1.80-1.75 (m, 2H), 1.48 (s, 18H), 1.45-1.43 (m, 2H); ^{13}C NMR ($CDCl_3$, 100 MHz) δ 171.5, 171.1, 152.3, 139.0, 137.9, 128.8, 125.0, 120.5, 116.9, 83.3, 57.9, 52.3, 37.5, 29.6, 28.1, 25.9, 25.3, 21.5.

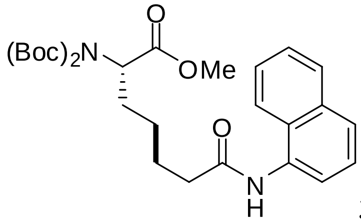

3bc

(*S*)-Methyl 2-(bis(*tert*-butoxycarbonyl)amino)-7-(naphthalen-1-ylamino)-7-oxoheptanoate (3bc): Eluent: petroleum ether/ethyl acetate (5:1). Yield: 32 mg (62%). Yellow solid, mp. 52-55℃. HRMS (ESI-TOF): calculated for $C_{28}H_{38}N_2O_7$ $[M+Na]^+$ m/z 537.2571, found 537.2572.

^{1}H NMR ($CDCl_3$, 400 MHz) δ 7.89-7.84 (m, 3H), (s, 1H), 7.68 (s, 2H), 7.49 (s, 2H), 7.45 (t, *J*= 8.33 Hz, 1H), 4.88 (s, 1H), 3.70 (s, 3H), 2.50 (t, *J*=6.41 Hz, 2H), 2.25-2.16 (m, 1H), 1.95-1.89 (m, 1H), 1.89-1.81 (m, 2H), 1.48 (s, 20H); ^{13}C NMR ($CDCl_3$, 100 MHz) δ 171.8, 171.4, 152.3, 134.2, 132.4, 128.7, 127.3, 126.3, 126.0, 125.9, 125.88, 121.2, 120.9, 83.3, 57.9, 52.2, 37.4, 29.7, 28.0, 26.0, 25.5.

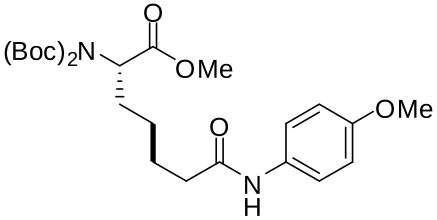

3bd

(*S*)-Methyl 2-(bis(*tert*-butoxycarbonyl)amino)-7-(4-methoxyphenylamino)-7-oxoheptanoate (3bd): Eluent: petroleum ether/ethyl acetate (4:1). Yield: 37.1 mg (75%). Yellow oil. HRMS (ESI-TOF): calculated for $C_{25}H_{38}N_2O_8$ $[M+Na]^+$ m/z 517.2520, found 517.2521.

^{1}H NMR ($CDCl_3$, 400 MHz) δ 7.41 (d, *J*= 8.79 Hz, 2H), 7.29 (s, 1H), 6.84 (d, *J*= 8.79 Hz, 2H), 4.85 (s, 1H), 3.78 (s, 3H), 3.71 (s, 3H), 2.33 (t, *J*=7.67 Hz, 2H), 2.22-2.14 (m, 1H), 1.93-1.83 (m, 1H), 1.80-1.73 (m, 2H), 1.48 (s, 18H), 1.45-1.42 (m, 2H); ^{13}C NMR ($CDCl_3$, 100 MHz) δ 171.4, 171.2, 156.3, 152.2, 131.3, 121.8, 114.1, 83.3, 57.9, 55.5, 54.8, 52.2, 37.2, 29.6, 28.0, 25.9, 25.3.

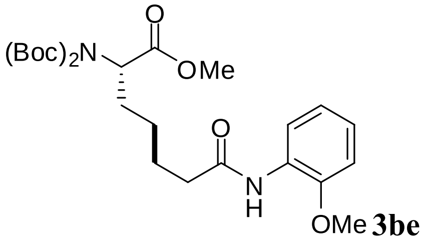

3be

(*S*)-Methyl 2-(bis(*tert*-butoxycarbonyl)amino)-7-(2-methoxyphenylamino)-7-oxoheptanoate (3be): Eluent: petroleum ether/ethyl acetate

(4:1). Yield: 25.8 mg (52%). Yellow oil. HRMS (ESI-TOF): calculated for $C_{25}H_{38}N_2O_8$ $[M+Na]^+$ m/z 517.2520, found 517.2521.

^1H NMR ($CDCl_3$, 400 MHz) δ 8.37 (d, *J*= 7.79 Hz, 1H), 7.75 (s, 1H), 7.02 (t, *J*= 7.79 Hz, 1H), 6.94 (t, *J*= 7.79 Hz, 1H), 6.86 (d, *J*= 9.16 Hz, 1H), 4.86 (dd, J_1= 9.39 Hz, J_2= 5.5 Hz, 1H), 3.88 (s, 3H), 3.70 (s, 3H), 2.39 (t, *J*=7.79 Hz, 2H), 2.21-2.10 (m, 1H), 1.96-1.86 (m, 1H), 1.83-1.66 (m, 2H), 1.48 (s, 18H), 1.45-1.43 (m, 2H); ^{13}C NMR ($CDCl_3$, 100 MHz) δ 171.4, 170.9, 152.2, 147.7, 127.8, 123.5, 121.2, 119.8, 109.9, 83.2, 58.1, 55.7, 52.3, 37.9, 29.8, 28.4, 28.0, 26.0, 25.4.

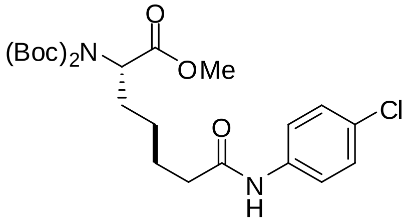

3bf

(*S*)-Methyl 2-(bis(*tert*-butoxycarbonyl)amino)-7-(4-chlorophenylamino)-7-oxoheptanoate (3bf): Eluent: petroleum ether/ethyl acetate (5:1). Yield: 32.5 mg (65%). Yellow oil. HRMS (ESI-TOF): calculated for $C_{24}H_{35}ClN_2O_7$ $[M+Na]^+$ m/z 521.2025, found 521.2028.

^1H NMR ($CDCl_3$, 400 MHz) δ 7.48 (d, *J*= 8.7 Hz, 3H), 7.25 (d, *J*= 8.7 Hz, 2H), 4.85 (dd, J_1= 9.39 Hz, J_2= 5.5 Hz, 1H), 3.71 (s, 3H), 2.34 (t, *J*=7.79 Hz, 2H), 2.23-2.14 (m, 1H), 1.91-1.82 (m, 1H), 1.80-1.73 (m, 2H), 1.48 (s, 18H), 1.45-1.43 (m, 2H); ^{13}C NMR ($CDCl_3$, 100 MHz) δ 171.5, 171.3, 152.3, 136.7, 129.1, 129.0, 121.1, 83.4, 57.9, 52.3, 37.3, 29.6, 28.0, 25.8, 25.2.

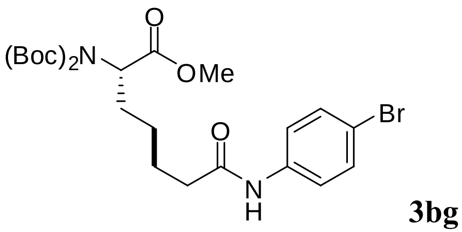

3bg

(*S*)-Methyl 2-(bis(*tert*-butoxycarbonyl)amino)-7-(4-bromophenylamino)-7-oxoheptanoate (3bg): Eluent: petroleum ether/ethyl acetate (5:1). Yield: 35.4 mg (65%). Yellow oil. HRMS (ESI-TOF): calculated for $C_{24}H_{35}BrN_2O_7$ $[M+Na]^+$ m/z 567.1503, found 567.1504.

^{1}H NMR ($CDCl_3$, 400 MHz) δ 7.54 (s, 1H), 7.44 (d, *J*= 8.7 Hz, 2H), 7.40 (d, *J*= 8.7 Hz, 2H), 4.84 (dd, J_1= 9.39 Hz, J_2= 5.5 Hz, 1H), 3.71 (s, 3H), 2.34 (t, *J*=7.79 Hz, 2H), 2.23-2.14 (m, 1H), 1.91-1.81 (m, 1H), 1.80-1.67 (m, 2H), 1.48 (s, 18H), 1.45-1.41 (m, 2H); ^{13}C NMR ($CDCl_3$, 100 MHz) δ 171.5, 171.3, 152.3, 137.2, 131.9, 121.4, 116.6, 83.4, 57.9, 52.3, 37.3, 29.6, 28.4, 28.0, 25.8, 25.1.

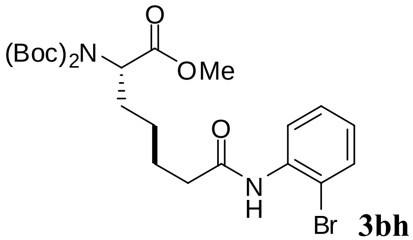

3bh

(*S*)-Methyl 2-(bis(*tert*-butoxycarbonyl)amino)-7-(2-bromophenylamino)-7-oxoheptanoate (3bh): Eluent: petroleum ether/ethyl acetate (5:1). Yield: 32.6 mg (60%). Yellow oil. HRMS (ESI-TOF): calculated for $C_{24}H_{35}BrN_2O_7$ $[M+Na]^+$ m/z 567.1503, found 567.1504.

^{1}H NMR ($CDCl_3$, 400 MHz) δ 8.33 (d, *J*= 7.79 Hz, 1H),7.61 (s, 1H), 7.52 (d, *J*= 7.79 Hz, 1H), 7.30 (t, *J*= 7.79 Hz, 1H), 6.96 (t, *J*= 7.56 Hz, 1H), 4.87 (dd, J_1= 9.39 Hz, J_2= 5.5 Hz, 1H), 3.71 (s, 3H), 2.43 (t, *J*=7.79 Hz, 2H), 2.22-2.13 (m, 1H), 1.97-1.86 (m, 1H), 1.84-1.75 (m, 2H), 1.48 (s,

20H); ^{13}C NMR ($CDCl_3$, 100 MHz) δ 171.4, 171.0, 152.2, 135.7, 132.2, 128.4, 125.1, 122.0, 113.3, 83.2, 58.0, 52.2, 37.8, 29.8, 28.1, 26.0, 25.2.

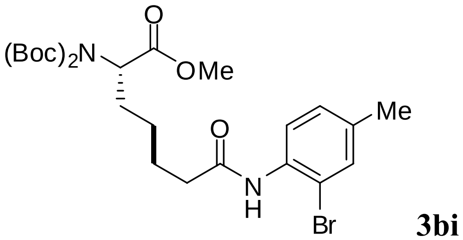

3bi

(*S*)-Methyl 2-(bis(*tert*-butoxycarbonyl)amino)-7-(2-bromo-4-methylphenylamino)-7-oxoheptanoate (3bi): Eluent: petroleum ether/ethyl acetate (5:1). Yield: 32.5 mg (58%). Yellow oil. HRMS (ESI-TOF): calculated for $C_{25}H_{37}BrN_2O_7$ $[M+Na]^+$ m/z 581.1660, found 581.1660.

^{1}H NMR ($CDCl_3$, 400 MHz) δ 8.16 (d, *J*= 8.24 Hz, 1H), 7.51 (s, 1H), 7.34 (s, 1H), 7.10 (d, *J*= 8.24 Hz, 1H), 4.87 (dd, J_1= 9.39 Hz, J_2= 5.5 Hz, 1H), 3.71 (s, 3H), 2.41 (t, *J*=7.79 Hz, 2H), 2.29 (s, 3H), 2.20-2.13 (m, 1H), 1.97-1.87 (m,1H), 1.81-1.76 (m, 2H), 1.48 (s, 20H); ^{13}C NMR ($CDCl_3$, 100 MHz) δ 171.4, 170.9, 152.2, 135.2, 133.2, 132.5, 129.0, 122.0, 113.4, 83.2, 58.0, 52.2, 37.7, 29.8, 28.1, 26.0, 25.3, 20.6.

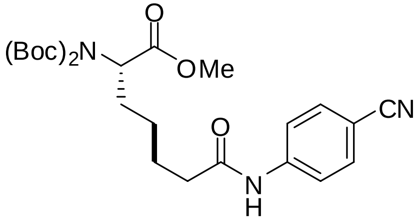

3bj

(*S*)-Methyl 2-(bis(*tert*-butoxycarbonyl)amino)-7-(4-cyanophenylamino)-7-oxoheptanoate (3bj): Eluent: petroleum ether/ethyl acetate (3:1). Yield: 29.4 mg (60%). Yellow oil. HRMS (ESI-TOF): calculated for $C_{25}H_{35}N_3O_7$ $[M+Na]^+$ m/z 512.2367, found 512.2367.

^{1}H NMR ($CDCl_3$, 400 MHz) δ 8.03 (s, 1H), 7.71 (d, *J*= 8.7 Hz, 2H), 7.58 (d, *J*= 8.7 Hz, 1H), 4.84 (dd, J_1= 9.39 Hz, J_2= 5.5 Hz, 1H), 3.71 (s, 3H),

2.39 (t, *J*=7.79 Hz, 2H), 2.24-2.15 (m, 1H), 1.91-1.83 (m, 1H), 1.81-1.74 (m, 2H), 1.48 (s, 20H); ^{13}C NMR ($CDCl_3$, 100 MHz) δ 171.8, 171.5, 152.3, 142.4, 133.2, 119.5, 119.0, 106.7, 83.5, 57.8, 52.3, 37.3, 29.5, 28.0, 25.7, 25.0.

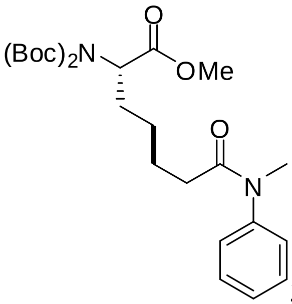

3bk

(*S*)-Methyl 7-(benzylamino)-2-(bis(*tert*-butoxycarbonyl)amino)-7-oxoheptanoate (3bk): Eluent: petroleum ether/ethyl acetate (5:1). Yield: 25 mg (52%). Yellow oil. HRMS (ESI-TOF): calculated for $C_{25}H_{38}N_2O_7$ $[M+H]^+$ m/z 479.2752, found 479.2750.

^{1}H NMR ($CDCl_3$, 400 MHz) δ 7.41 (t, *J*= 7.33 Hz, 2H), 7.32 (t, *J*= 7.33 Hz, 1H), 7.16 (d, *J*= 7.79 Hz, 2H), 4.77 (dd, J_1= 9.39 Hz, J_2= 5.5 Hz, 1H), 3.68 (s, 3H), 3.25 (s, 3H), 2.06 (t, *J*=7.79 Hz, 2H), 2.01-1.96 (m, 1H), 1.81-1.74 (m, 1H), 1.64-1.57 (m, 2H), 1.46 (s, 20H); ^{13}C NMR ($CDCl_3$, 100 MHz) δ 173.0, 171.4, 152.1, 144.3, 129.8, 127.8, 127.4, 83.q, 58.0, 52.2, 37.4, 34.0, 29.7, 28.0, 26.1, 25.3.

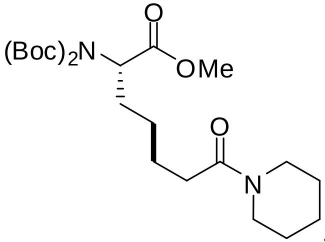

3bl

(*S*)-Methyl 2-(bis(*tert*-butoxycarbonyl)amino)-7-oxo-7-(piperidin-1-yl)heptanoate (3bl): Eluent: petroleum ether/ethyl acetate (5:1). Yield: 23.9 mg (52%). Yellow oil. HRMS (ESI-TOF): calculated for $C_{23}H_{40}N_2O_7$ $[M+Na]^+$ m/z 479.2728, found 479.2729.

^{1}H NMR ($CDCl_3$, 400 MHz) δ 4.85 (dd, J_1= 9.39 Hz, J_2= 5.5 Hz, 1H), 3.70 (s, 3H), 3.53 (t, *J*=5.5 Hz, 2H), 3.37 (t, *J*=5.95 Hz, 2H), 2.31 (t, *J*=7.33 Hz, 2H), 2.18-2.09 (m, 1H), 1.93-1.86 (m, 1H), 1.69-1.60 (m, 6H), 1.54-1.52 (m, 2H), 1.49 (s, 18H), 1.45-1.36 (m, 2H); ^{13}C NMR ($CDCl_3$, 100 MHz) δ 171.5, 171.2, 152.2, 83.2, 58.1, 52.2, 46.7, 42.7, 33.4, 29.8, 28.1, 26.7, 26.3, 25.7, 25.3, 24.7.

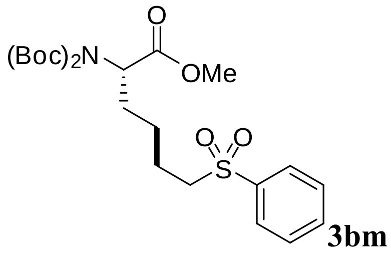

3bm

(*S*)-Methyl 2-(bis(*tert*-butoxycarbonyl)amino)-6-(phenylsulfonyl)hexanoate (3bm): Eluent: petroleum ether/ethyl acetate (8:1). Yield: 34 mg (70%). Yellow oil. HRMS (ESI-TOF): calculated for $C_{23}H_{35}NO_8S$ $[M+Na]^+$ m/z 508.1976, found 508.1974.

^{1}H NMR ($CDCl_3$, 400 MHz) δ 7.90 (d, *J*=7.33 Hz, 2H), 7.66 (t, *J*=7.33 Hz, 1H), 7.57 (t, *J*=7.79 Hz, 2H), 4.78 (dd, J_1=9.16 Hz, J_2=5.04 Hz, 1H), 3.69 (s, 3H), 3.08 (t, *J*=8.24 Hz, 2H), 2.12-2.03 (m, 1H), 1.87-1.80 (m, 1H), 1.75-1.72 (m, 2H), 1.65-1.55 (m, 2H), 1.48 (s, 18H); ^{13}C NMR ($CDCl_3$, 100 MHz) δ 171.1, 152.2, 139.1, 133.8, 129.4, 128.1, 83.4, 57.8, 56.2, 52.3, 29.6, 28.1, 25.2, 22.6.

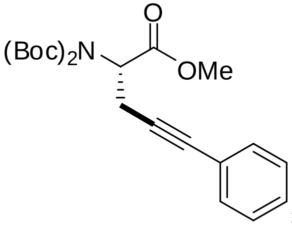

5a

(*S*)-Methyl 2-(bis(*tert*-butoxycarbonyl)amino)-5-phenylpent-4-ynoate (5a): Eluent: petroleum ether/ethyl acetate (15:1). Yield: 32.8 mg (81%). Colorless oil. HRMS (ESI-TOF): calculated for $C_{22}H_{29}NO_6$ $[M+Na]^+$ m/z 426.1887, found 426.1882.

^{1}H NMR ($CDCl_3$, 400 MHz) δ 7.34 (s, 2H), 7.24 (m, 3H), 5.23 (dd, J_1=8.47 Hz, J_2=6.41 Hz, 1H), 3.73 (s, 3H), 3.16 (d, J=3.85 Hz, 1H), 3.14 (s, 1H), 1.45 (s, 18H); ^{13}C NMR ($CDCl_3$, 100 MHz) δ 170.2, 151.9, 131.8, 128.2, 127.9, 123.6, 85.5, 83.4, 82.7, 56.8, 52.5, 28.1, 21.5.

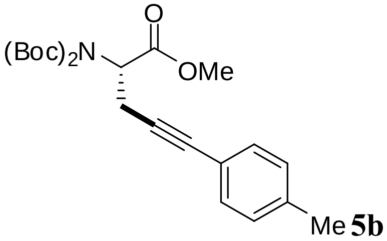

5b

(*S*)-Methyl 2-(bis(*tert*-butoxycarbonyl)amino)-5-*p*-tolylpent-4-ynoate (5b): Eluent: petroleum ether/ethyl acetate (15:1). Yield: 33.5 mg (80%). Colorless oil. HRMS (ESI-TOF): calculated for $C_{23}H_{31}NO_6$ $[M+Na]^+$ m/z 440.2044, found 440.2036.

^{1}H NMR ($CDCl_3$, 400 MHz) δ 7.26 (d, J=8.24 Hz, 2H), 7.06 (d, J=8.24 Hz, 2H), 5.24 (dd, J_1=8.47 Hz, J_2=6.41 Hz, 1H), 3.74 (s, 3H), 3.16 (d, J=3.85 Hz, 1H), 3.14 (s, 1H), 2.31 (s, 3H), 1.45 (s, 18H); ^{13}C NMR ($CDCl_3$, 100 MHz) δ 170.3, 151.9, 137.9, 131.6, 128.9, 120.5, 84.7, 83.4, 82.7, 56.9, 52.5, 28.1, 21.5.

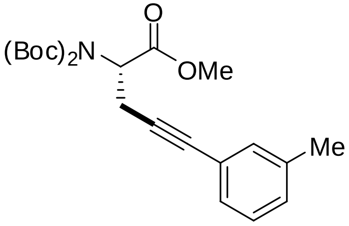

5c

(*S*)-Methyl 2-(bis(*tert*-butoxycarbonyl)amino)-5-*m*-tolylpent-4-ynoate (5c): Eluent: petroleum ether/ethyl acetate (15:1). Yield: 32.5 mg (78%). Colorless oil. HRMS (ESI-TOF): calculated for $C_{23}H_{31}NO_6$ $[M+Na]^+$ m/z 440.2044, found 440.2036.

^{1}H NMR ($CDCl_3$, 400 MHz) δ 7.20-7.06 (m, 5H), 7.24 (m, 3H), 5.24 (dd, J_1=8.47 Hz, J_2=6.41 Hz, 1H), 3.74 (s, 3H), 3.17 (d, *J*=3.85 Hz, 1H), 3.15 (s, 1H), 2.28 (s, 3H), 1.47 (s, 18H); ^{13}C NMR ($CDCl_3$, 100 MHz) δ 170.2, 151.9, 137.8, 132.4, 128.84, 128.80, 128.1, 123.2, 85.1, 83.4, 82.8, 56.8, 52.8, 28.1, 21.5, 21.2.

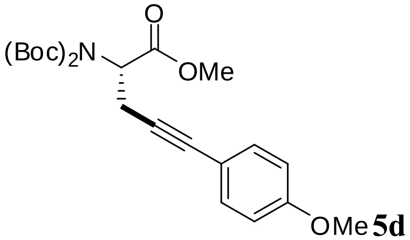

5d

(*S*)-Methyl 2-(bis(*tert*-butoxycarbonyl)amino)-5-(4-methoxyphenyl)pent-4-ynoate (5d): Eluent: petroleum ether/ethyl acetate (10:1). Yield: 37 mg (85%). Colorless oil. HRMS (ESI-TOF): calculated for $C_{23}H_{31}NO_7$ $[M+Na]^+$ m/z 456.1993, found 456.1997.

^{1}H NMR ($CDCl_3$, 400 MHz) δ 7.30 (d, *J*=8.7 Hz, 2H), 6.78 (d, *J*=8.7 Hz, 2H), 5.23 (dd, J_1=8.82 Hz, J_2=6.64 Hz, 1H), 3.79 (s, 3H), 3.74 (s, 3H), 3.16 (d, *J*=3.96 Hz, 1H), 3.14 (d, *J*=1.37 Hz, 1H), 1.47 (s, 18H); ^{13}C NMR ($CDCl_3$, 100 MHz) δ 170.3, 159.3, 151.9, 133.1, 115.8, 113.8, 83.9,

83.4, 82.5, 56.9, 55.3, 52.5, 28.1, 21.5.

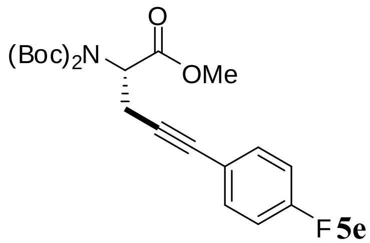
5e

(*S*)-Methyl 2-(bis(*tert*-butoxycarbonyl)amino)-5-(4-fluorophenyl)pent-4-ynoate (5e): Eluent: petroleum ether/ethyl acetate (15:1). Yield: 27.4 mg (65%). Colorless oil. HRMS (ESI-TOF): calculated for $C_{22}H_{28}FNO_6$ $[M+Na]^+$ m/z 444.1793, found 444.1791.

^{1}H NMR ($CDCl_3$, 400 MHz) δ 7.35 (dd, J_1=8.7 Hz, J_2=5.5 Hz, 2H), 6.95 (t, J=8.7 Hz, 2H), 5.23 (dd, J_1=8.82 Hz, J_2=6.64 Hz, 1H), 3.74 (s, 3H), 3.16 (d, J=3.21 Hz, 1H), 3.14 (s, 1H), 1.47 (s, 18H); ^{13}C NMR ($CDCl_3$, 100 MHz) δ 170.2, 163.5, 161.1, 151.9, 133.6, 133.5, 119.7, 119.6, 115.5, 115.3, 85.3, 83.5, 81.6, 56.8, 52.5, 28.1, 21.5.

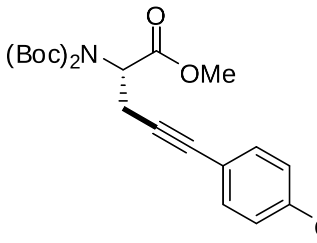
5f

(*S*)-Methyl 2-(bis(*tert*-butoxycarbonyl)amino)-5-(4-chlorophenyl)pent-4-ynoate (5f): Eluent: petroleum ether/ethyl acetate (15:1). Yield: 32.5 mg (74%). Colorless oil. HRMS (ESI-TOF): calculated for $C_{22}H_{28}ClNO_6$ $[M+Na]^+$ m/z 460.1497, found 460.1491.

^{1}H NMR ($CDCl_3$, 400 MHz) δ 7.30 (d, *J*=8.24 Hz, 2H), 7.23 (d, *J*=8.24 Hz, 2H), 5.23 (dd, J_1=8.82 Hz, J_2=6.64 Hz, 1H), 3.74 (s, 3H), 3.17 (d, *J*=3.21 Hz, 1H), 3.15 (s, 1H), 1.47 (s, 18H); ^{13}C NMR ($CDCl_3$, 100 MHz) δ 170.1, 151.9, 133.9, 133.0, 128.5, 122.1, 86.7, 83.5, 81.6, 56.67, 52.6, 28.1, 21.5.

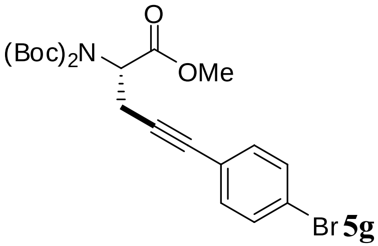

5g

(*S*)-Methyl 2-(bis(*tert*-butoxycarbonyl)amino)-5-(4-bromophenyl)pent-4-ynoate (5g): Eluent: petroleum ether/ethyl acetate (15:1). Yield: 36.2 mg (75%). Colorless oil. HRMS (ESI-TOF): calculated for $C_{22}H_{28}BrNO_6$ $[M+Na]^+$ m/z 506.0975, found 506.0979.

^{1}H NMR ($CDCl_3$, 400 MHz) δ 7.39 (d, *J*=8.70 Hz, 2H), 7.23 (d, *J*=8.24 Hz, 2H), 5.23 (dd, J_1=8.82 Hz, J_2=6.64 Hz, 1H), 3.74 (s, 3H), 3.17 (d, *J*=3.21 Hz, 1H), 3.14 (s, 1H), 1.47 (s, 18H); ^{13}C NMR ($CDCl_3$, 100 MHz) δ 170.1, 151.9, 133.2, 131.4, 122.5, 122.1, 86.9, 83.5, 81.6, 56.7, 52.5, 28.1, 21.5.

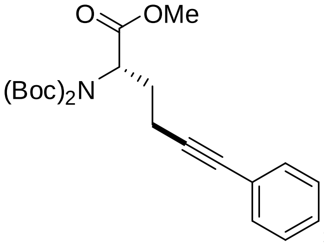

5h

(*S*)-Methyl 2-(bis(*tert*-butoxycarbonyl)amino)-6-phenylhex-5-ynoate (5h): Eluent: petroleum ether/ethyl acetate (15:1). Yield: 31 mg (74%).

Colorless oil. HRMS (ESI-TOF): calculated for $C_{23}H_{31}NO_6$ $[M+Na]^+$ m/z 440.2044, found 440.2044.

^{1}H NMR ($CDCl_3$, 400 MHz) δ 7.42-7.39 (m, 2H), 7.27-7.25 (m, 3H), 5.10 (dd, J_1=9.39 Hz, J_2=5.04 Hz, 1H), 3.73 (s, 3H), 2.55-2.4 (m, 3H), 2.25-2.15 (m, 1H), 1.48 (s, 18H); ^{13}C NMR ($CDCl_3$, 100 MHz) δ 171.2, 152.1, 131.8, 128.2, 127.7, 123.8, 88.8, 83.3, 81.4, 57.6, 52.3, 29.5, 28.0, 16.8.

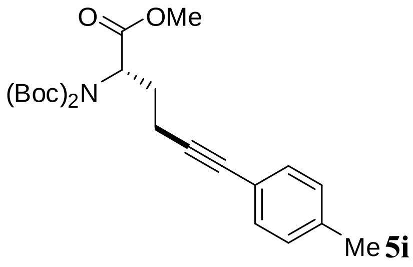

5i

(*S*)-Methyl 2-(bis(*tert*-butoxycarbonyl)amino)-6-*p*-tolylhex-5-ynoate (5i): Eluent: petroleum ether/ethyl acetate (15:1). Yield: 33.7 mg (78%). Colorless oil. HRMS (ESI-TOF): calculated for $C_{24}H_{33}NO_6$ $[M+Na]^+$ m/z 454.2200, found 454.2204.

^{1}H NMR ($CDCl_3$, 400 MHz) δ 8.24 (d, J=8.24 Hz, 2H), 7.07 (d, J=8.24 Hz, 2H), 5.09 (dd, J_1=9.39 Hz, J_2=5.04 Hz, 1H), 3.72 (s, 3H), 2.53-2.4 (m, 3H), 2.32 (s, 3H), 2.23-2.14 (m, 1H), 1.48 (s, 18H); ^{13}C NMR ($CDCl_3$, 100 MHz) δ 171.2, 152.1, 137.7, 131.6, 129.0, 120.7, 88.0, 83.3, 81.4, 57.6, 52.3, 29.6, 28.1, 21.5, 16.8.

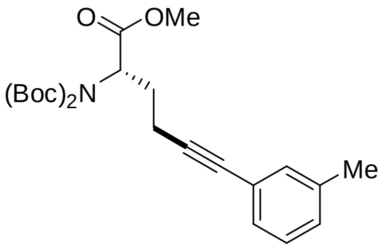

5j

(*S*)-Methyl 2-(bis(*tert*-butoxycarbonyl)amino)-6-*m*-tolylhex-5-ynoate (5j): Eluent: petroleum ether/ethyl acetate (15:1). Yield: 32.7 mg (76%). Colorless oil. HRMS (ESI-TOF): calculated for $C_{24}H_{33}NO_6$ $[M+Na]^+$ m/z 454.2200, found 454.2204.

^{1}H NMR ($CDCl_3$, 400 MHz) δ 7.24 (s, 1H), 7.21(d, *J*=7.45 Hz, 1H), 7.15 (t, *J*=7.98 Hz, 1H), 7.07 (d, *J*=7.33 Hz, 1H), 5.09 (dd, J_1=9.39 Hz, J_2=5.04 Hz, 1H), 3.73 (s, 3H), 2.54-2.41 (m, 3H), 2.30 (s, 3H), 2.24-2.12 (m, 1H), 1.48 (s, 18H); ^{13}C NMR ($CDCl_3$, 100 MHz) δ 171.2, 152.1, 137.8, 132.4, 128.8, 128.6, 128.1, 123.5, 88.4, 83.3, 81.5, 57.6, 52.3, 29.6, 28.1, 21.3, 16.8.

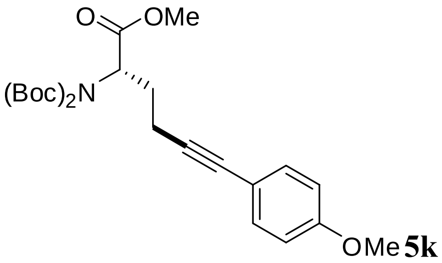

5k

(*S*)-Methyl 2-(bis(*tert*-butoxycarbonyl)amino)-6-(4-methoxyphenyl)hex-5-ynoate (5k): Eluent: petroleum ether/ethyl acetate (10:1). Yield: 35.9 mg (80%). Colorless oil. HRMS (ESI-TOF): calculated for $C_{24}H_{33}NO_7$ $[M+Na]^+$ m/z 470.2149, found 470.2151.

^{1}H NMR ($CDCl_3$, 400 MHz) δ 7.34 (d, J=8.7 Hz, 2H), 6.80 (d, J=8.7 Hz, 2H), 5.09 (dd, J_1=9.39 Hz, J_2=5.04 Hz, 1H), 3.79 (s, 3H), 3.72 (s, 3H), 2.55-2.39 (m, 3H), 2.23-2.14 (m, 1H), 1.48 (s, 18H); ^{13}C NMR ($CDCl_3$, 100 MHz) δ 171.2, 159.1, 152.1, 133.1, 116.0, 113.8, 87.1, 83.2, 81.1, 57.6, 55.3, 52.3, 29.6, 28.0, 16.8.

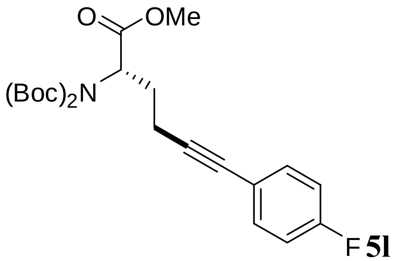

5l

(*S*)-Methyl 2-(bis(*tert*-butoxycarbonyl)amino)-6-(4-fluorophenyl)hex-5-ynoate (5l): Eluent: petroleum ether/ethyl acetate (15:1). Yield: 27 mg (62%). Colorless oil. HRMS (ESI-TOF): calculated for $C_{23}H_{30}FNO_6$ $[M+Na]^+$ m/z 458.1949, found 458.1953.

^{1}H NMR ($CDCl_3$, 400 MHz) δ 7.39 (dd, J_1=8.93 Hz, J_2=5.5 Hz, 2H), 6.96 (t, J=8.7 Hz, 2H), 5.10 (dd, J_1=9.39 Hz, J_2=5.04 Hz, 1H), 3.74 (s, 3H), 2.53-2.40 (m, 3H), 2.24-2.15 (m, 1H), 1.48 (s, 18H); ^{13}C NMR ($CDCl_3$, 100 MHz) δ 171.2, 163.4, 160.9, 152.1, 133.6, 133.5, 119.9, 119.8, 115.5, 115.3, 88.4, 83.3, 80.4, 57.5, 52.3, 29.4, 28.0, 16.7.

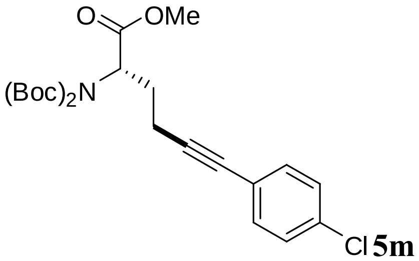

5m

(*S*)-Methyl 2-(bis(*tert*-butoxycarbonyl)amino)-6-(4-chlorophenyl)hex-5-ynoate (5m): Eluent: petroleum ether/ethyl acetate (15:1). Yield: 30.8 mg (68%). Colorless oil. HRMS (ESI-TOF): calculated for $C_{23}H_{30}ClNO_6$ $[M+Na]^+$ m/z 474.1654, found 474.1655.

^{1}H NMR (CDCl$_3$, 400 MHz) δ 7.34 (d, *J*=8.24 Hz , 2H), 7.24 (d, *J*=8.7 Hz, 2H), 5.10 (dd, J_1=9.39 Hz, J_2=5.04 Hz, 1H), 3.74 (s, 3H), 2.54-2.40 (m, 3H), 2.24-2.14 (m, 1H), 1.48 (s, 18H); ^{13}C NMR (CDCl$_3$, 100 MHz) δ 171.2, 152.1, 133.6, 133.0, 128.5, 122.3, 89.9, 83.3, 80.4, 57.5, 52.3, 29.4, 28.1, 16.8.

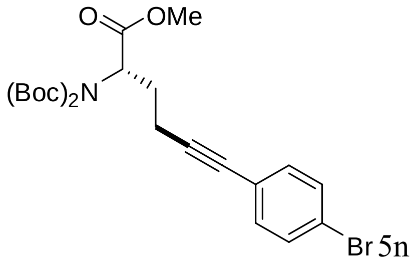

5n

(*S*)-Methyl 2-(bis(*tert*-butoxycarbonyl)amino)-6-(4-bromophenyl)hex-5-ynoate (5n): Eluent: petroleum ether/ethyl acetate (15:1). Yield: 34.8 mg (70%). Colorless oil. HRMS (ESI-TOF): calculated for $C_{23}H_{30}ClNO_6$ $[M+Na]^+$ m/z 520.1132, found 520.1135.

^{1}H NMR (CDCl$_3$, 400 MHz) δ 7.39 (d, *J*=8.24 Hz , 2H), 7.27 (d, *J*=8.24 Hz, 2H), 5.10 (dd, J_1=9.39 Hz, J_2=5.04 Hz, 1H), 3.74 (s, 3H), 2.53-2.40 (m, 3H), 2.23-2.16 (m, 1H), 1.48 (s, 18H); ^{13}C NMR (CDCl$_3$, 100 MHz) δ 171.2, 152.1, 133.3, 131.4, 122.8, 121.8, 90.1, 83.3, 80.4, 57.5, 52.3, 29.3, 28.1, 16.8.

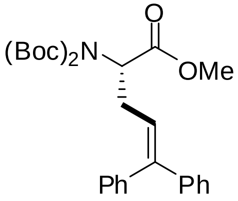

7a

(*S*)-Methyl 2-(bis(*tert*-butoxycarbonyl)amino)-5,5-diphenylpent-4-enoate (5a): Eluent: petroleum ether/ethyl acetate (15:1). Yield: 33.7 mg (70%). Viscous oil. HRMS (ESI-TOF): calculated for $C_{28}H_{35}NO_6$ $[M+Na]^+$ m/z 504.2357, found 504.2359.

^{1}H NMR ($CDCl_3$, 400 MHz) δ 7.36-7.27 (m, 4H), 7.22-7.15 (m, 6H), 6.04 (dd, J_1=9.06 Hz, J_2=5.98 Hz, 1H), 5.10 (dd, J_1=10.07 Hz, J_2=5.04 Hz, 1H), 3.68 (s, 3H), 2.96-2.78 (m, 2H), 1.39 (s, 18H); ^{13}C NMR ($CDCl_3$, 100 MHz) δ 170.9, 152.0, 144.4, 142.6, 139.7, 130.0, 129.8, 128.3, 128.1, 127.5, 127.2, 125.0, 83.2, 58.3, 52.3, 30.7, 28.0.

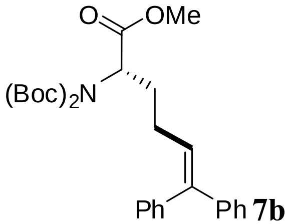

7b

(*S*)-Methyl 2-(bis(*tert*-butoxycarbonyl)amino)-6,6-diphenylhex-5-enoate (7b): Eluent: petroleum ether/ethyl acetate (15:1). Yield: 35.2 mg (71%). Viscous oil. HRMS (ESI-TOF): calculated for $C_{29}H_{37}NO_6$ $[M+Na]^+$ m/z 518.2513, found 518.2518.

^{1}H NMR ($CDCl_3$, 400 MHz) δ 7.36-7.27 (m, 4H), 7.22-7.20 (m, 4H), 7.16-7.13 (m, 2H), 6.07 (t, J=7.33 Hz, 1H), 4.86 (dd, J_1=10.07 Hz, J_2=5.04 Hz, 1H), 3.68 (s, 3H), 2.31-2.22 (m, 1H), 2.20-2.13 (m, 2H), 2.06-1.97 (m, 1H), 1.44 (s, 18H); ^{13}C NMR ($CDCl_3$, 100 MHz) δ 171.3,

152.1, 142.6, 140.0, 129.9, 128.4, 128.37, 128.34, 128.1, 127.4, 127.1, 127.0, 83.1, 57.9, 52.2, 30.4, 28.0, 26.8.

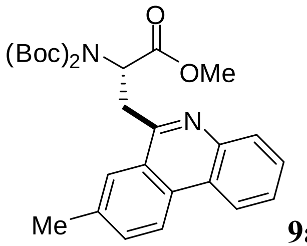

9a

(*S*)-Methyl 2-(bis(*tert*-butoxycarbonyl)amino)-3-(8-methylphenanthridin-6-yl)propanoate (9a): Eluent: petroleum ether/ethyl acetate (5:1). Yield: 32.2 mg (65%). Viscous oil. HRMS (ESI-TOF): calculated for $C_{28}H_{34}N_2O_6$ $[M+Na]^+$ m/z 517.2309, found 517.2302.

^{1}H NMR ($CDCl_3$, 400 MHz) δ 8.51 (d, *J*=8.24 Hz , 1H), 8.48 (d, *J*=7.79 Hz , 1H), 8.70 (s, 1H), 8.03 (s, 1H), 7.64 (t, *J*=7.79 Hz , 2H), 7.58 (t, *J*=7.56 Hz , 1H), 5.96 (dd, J_1=8.93 Hz, J_2=5.04 Hz, 1H), 4.28-4.24 (m, 1H), 3.96-3.90 (m, 1H), 3.77 (s, 3H), 2.58 (s, 3H), 1.32 (s, 18H); ^{13}C NMR ($CDCl_3$, 100 MHz) δ 171.3, 157.7, 152.0, 137.3, 132.2, 130.8, 130.0, 128.0, 126.5, 125.9, 125.4, 123.8, 122.4, 121.7, 82.9, 57.8, 52.5, 35.6, 27.9, 21.9.

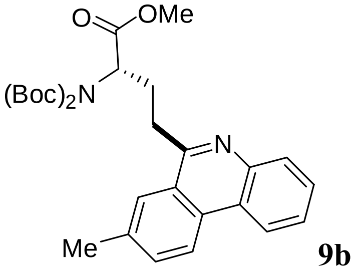

9b

(*S*)-Methyl 2-(bis(*tert*-butoxycarbonyl)amino)-4-(8-methylphenanthridin-6-yl)butanoate (9b): Eluent: petroleum ether/ethyl acetate (5:1).

Yield: 32.2 mg (65%). Viscous oil. HRMS (ESI-TOF): calculated for $C_{29}H_{36}N_2O_6$ $[M+Na]^+$ m/z 531.2466, found 531.2460.

^{1}H NMR ($CDCl_3$, 400 MHz) δ 8.49 (t, *J*=8.7 Hz , 2H), 8.10-8.06 (m, 2H), 7.67-7.63 (m, 2H), 7.58 (t, *J*=8.7 Hz , 2H), 7.64 (t, *J*=7.79 Hz , 2H), 7.58 (t, *J*=7.87 Hz , 1H), 5.25 (dd, J_1=8.7 Hz, J_2=5.5 Hz, 1H), 3.76 (s, 3H), 3.49-3.36 (m, 2H), 2.94-2.85 (m, 1H), 2.59 (s, 3H), 2.54-2.43 (m, 1H), 1.45 (s, 18H); ^{13}C NMR ($CDCl_3$, 100 MHz) δ 171.4, 160.2, 152.2, 137.3, 132.1, 130.7, 129.7, 128.1, 126.4, 125.6, 125.4, 123.8, 122.4, 121.7, 83.2, 58.3, 52.2, 32.5, 29.1, 28.0, 21.9.

General Synthesis and Characterization of *Rac*-3a, *Rac*-3s and *Rac*-3w

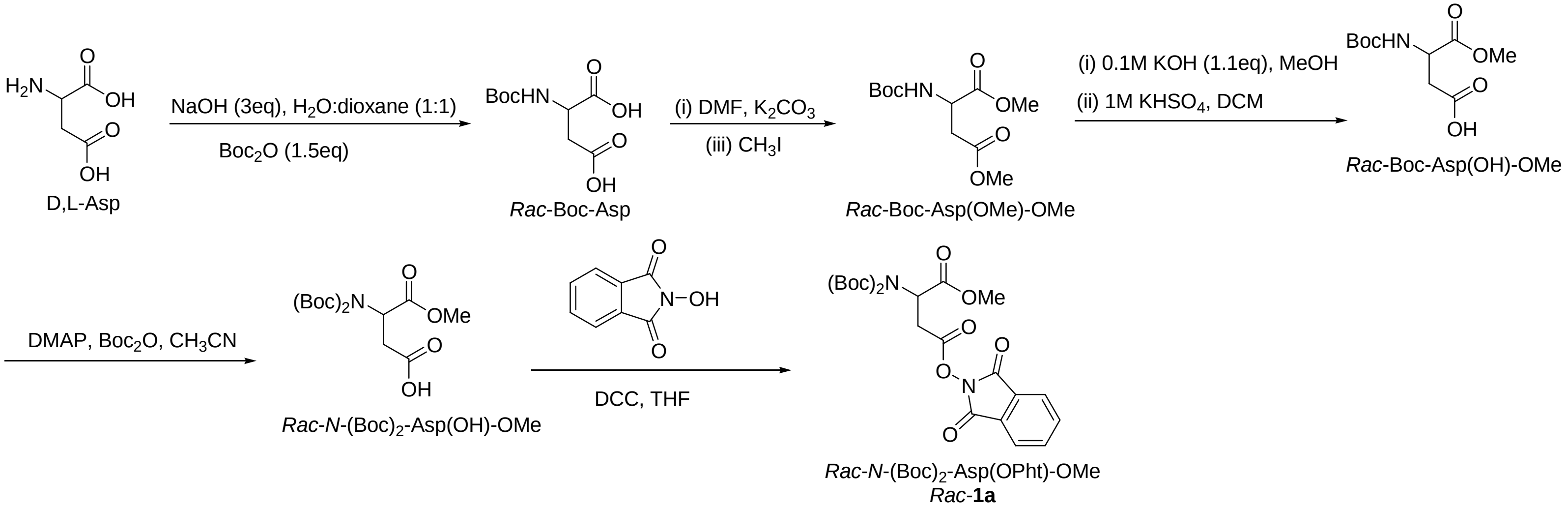

Synthesis of *Rac*-*N*-(Boc)$_2$-Asp(OPht)-OMe. D,L- aspartic acid (1.33 g, 10 mmol) and dioxane 20 mL were added to a 100 mL round bottom glass bottle equipped with a magnetic stir bar, then 20 mL NaOH (3 M) solution was added. After the mixture was stirred for 20 min, Boc_2O

(3.28 g, 15 mmol) in dioxane was added in dropwise, and the mixture was stirred at room temperature overnight. Dioxane was evaporated under reduced pressure, the remained aqueous was washed with Et_2O (2 × 50 mL), and the organic phase was exactracted with saturated $NaHCO_3$ (2 × 50 mL). The combined aqueous was acidified with 1M $KHSO_4$ and exactrated with etheyl acetate (3 × 100 mL). The combined organic phase was dried over Na_2SO_4, and evaporated to give Boc-Asp as a white solid (2.3 g, 98%). The white solid was dissolved in 30 mL DMF, and a well grounded K_2CO_3 (6 g) was added to the mixture. After the mixture was tured to a white suspension, CH_3I (4 mL) was added dropwise, and the mixture was stirred at room temperature for over 8 h. Water (50 mL) was added, and the resulting solution was exactracted with ethyl acetate (3 × 100 mL), and the combined organic phase was washed with water (3 × 50 mL), brine, dried over Na_2SO_4, evaporated under reduced pressure to give *Rac*-Boc-Asp(OMe)-OMe as a colorless oil (2.48 g, 97%). According to the previous reference[2], to a 55 mL of 0.1 M KOH solution was added a solution of *Rac*-Boc-Asp(OMe)-OMe (5 mmol, 1.3 g) in 55 mL of methanol. Reaction mixture was stirred at room temperature overnight, and then methanol was evaporated. The remained aqueous phase was acidified with 6 mL of 1M $KHSO_4$. The solution was exactracted with CH_2Cl_2, and the combined organic phase was dried over anhydrous Na_2SO_4, and the solvent was evaporated to provide the desired procuct (*Rac*-Boc-Asp(OH)-OMe) as a white solid (1.04g, 85%). The solid was dissolved in 30 mL dry acetonitrile, and DMAP (1.037 g, 8.5 mmol) and Boc_2O (1.40 g, 6.4 mmol) were added to the mixture in one portion. After a stirring at room temperature overnight, water 50 mL was added to the mixture to make a white suspension, and then a solution of 1M HCl (10.2 mL) was added. The mixture was exactracted with ethyl acetate (3 × 50 mL), the combined organic was washed with saturated NH_4Cl (2 × 40 mL), dried with Na_2SO_4, evaporated, and the residue was purified by silica gel column chromatography to give a light brown oil *Rac*-*N*-$(Boc)_2$-Asp(OH)-OMe 1.18g, 80%. *Rac*-*N*-$(Boc)_2$-Asp(OPht)-OMe was synthsized according to the procedures described above (see S4), *Rac*-*N*-$(Boc)_2$-Asp(OH)-OMe (1g, 2.9

mmol) was added to a solution of *N*-hydroxyphthalimide (0.52 g, 3.2 mmol) in dry THF (10mL) in a 25 mL round bottom flask charged. After the mixture was stirred for over 10 minutes, DCC (0.6 g, 2.9 mmol) was added to the flask in portions, and the reaction was performed at room temperature for 24 hours. After the reaction completed, the resulting mixture was filtered, the solvent of the remaining solution was removed under reduced pressure, and the crude product was purified by flash silica gel column chromatography (DCM/EtOAc = 60/1, R_f = 0.52) to give *Rac*-*N*-$(Boc)_2$-Asp(OPht)-OMe (*Rac*-**1a**) as a viscous pale yellow oil, yield: 0.97 g (68%).^{1}H NMR ($CDCl_3$, 400 MHz) δ 7.88 (dd, J_1 = 5.5 Hz, J_2 = 3.2 Hz, 2H), 7.79 (dd, J_1 = 5.5 Hz, J_2 = 3.2 Hz, 2H), 5.53 (dd, J_1 = 7.6 Hz, J_2 = 6.0 Hz, 1H), 3.8 (s, 3H), 3.66 (dd, J_1 = 17.2 Hz, J_2 = 5.95 Hz, 1H), 3.11 (dd, J_1 = 17.2 Hz, J_2 = 6.0 Hz, 1H), 1.51 (s, 18H). ^{13}C NMR ($CDCl_3$, 100 MHz) δ 169.5, 167.2, 161.7, 151.5, 134.8, 129.0, 124.0, 84.0, 54.5, 52.9, 33.0, 28.0.

Synthesis of *Rac*-3a, *Rac*-3s, *Rac*-3w: To a 25-mL Schlenk tube equipped with a Teflon septum and magnetic stir bar were added $[Ru(bpy)_3]Cl_2$ (1.0 μmol, 0.78 mg), and Hantzsch ester (HE) (0.15 mmol, 38 mg). The tube was evacuated and back-filled with nitrogen for three cycles and then sealed under an atmosphere of nitrogen. *Rac*-**1a** (*Rac*-*N*-Bis(Boc)-Asp(OPht)-OMe) (0.15 mmol), olefins (**2a**, **2s**, **2w**) (0.10-0.15 mmol, if liquid) (see Fig. 2 for amount of **1a** and **2**) and DIPEA (0.25 mmol, 42 μL, 32.3 mg) were dissolved in 1.0 mL of dichloromethane (DCM), and then the solution was added to the tube by syringe. The resulting solution was freezed with liquid nitrogen, and the tube was degassed by alternating vacuum evacuation then allowing it to warm to room temperature for three cycles. The tube was irradiated with a 40 W fluorescent lamp at room temperature (approximately 2 cm away from the light source). After the complete conversion of the substrates (monitored by TLC), the reaction mixture was diluted with 20 mL of EtOAc, and the solution was filtered by flash chromatography. The filtrate was evaporated by rotary evaporator, and the residue was purified by silica gel column chromatography or preparative thin layer chromatography (pTLC) to give

the desired product (*Rac*-**3a,** *Rac*-**3s,** *Rac*-**3w**).

Rac-**3a:** ^{1}H NMR ($CDCl_3$, 400 MHz) δ 7.94 (d, *J* = 8.2 Hz, 2H), 7.55 (t, *J* =7.3 Hz, 1H), 7.45 (t, *J* =7.8 Hz, 2H), 4.93 (dd, J_1=9.6 Hz, J_2=5.0 Hz, 1H), 3.71 (s, 3H), 3.11-3.03 (m, 1H), 3.00-2.92 (m, 1H), 2.22-2.15 (m, 1H), 2.06-1.97 (m, 1H) , 1.85-1.77 (m, 2H), 1.50 (s, 18H); ^{13}C NMR ($CDCl_3$, 100 MHz) δ 199.7, 171.3, 152.2, 137.1, 133.0, 128.6, 128.1, 83.2, 57.9, 52.2, 38.1, 29.5, 28.1, 20.9.

Rac-**3s:** ^{1}H NMR ($CDCl_3$, 400 MHz) δ 7.52 (d, *J*=6.87 Hz, 3H), 7.30 (t, *J*=7.79 Hz, 2H), 7.08 (t, J_1=6.87 Hz, 1H), 4.89 (dd, J_1=8.2 Hz, J_2=5.5 Hz, 1H), 3.71 (s, 3H), 2.47-2.30 (m, 2H), 2.24-2.18 (m, 1H), 2.00-1.91 (m, 1H), 1.85-1.78 (m, 2H), 1.49 (s, 18H); ^{13}C NMR ($CDCl_3$, 100 MHz) δ 171.4, 170.8, 152.4, 138.1, 129.0, 124.2, 119.9, 83.5, 57.7, 52.3, 37.0, 29.4, 28.1, 22.4.

Rac-**3w:** ^{1}H NMR ($CDCl_3$, 400 MHz) δ 7.42 (d, *J*=9.16 Hz, 2H), 7.38(s, 1h), 6.84 (d, *J*=9.16 Hz, 2H), 4.89 (dd, J_1=8.2 Hz, J_2=5.5 Hz, 1H), 3.78 (s, 3H), 3.72 (s, 3H), 2.45-2.30 (m, 2H), 2.27-2.17 (m, 1H), 2.00-1.90 (m, 1H), 1.88-1.73 (m, 2H), 1.49 (s, 18H); ^{13}C NMR ($CDCl_3$, 100 MHz) δ 171.4, 170.6, 156.4, 152.3, 131.2, 121.8, 114.2, 83.5, 57.6, 55.6, 52.3, 36.9, 29.5, 28.1, 22.5.

HPLC of *Rac*-3a, 3a, *Rac*-3s, 3s, *Rac*-3w and 3w.

Rac-**3a**, *Rac*-**3s**, *Rac*-**3w** and the synthesized **3a**, **3s**, **3w** were determined by HPLC with ID-H chiral column using n-hexane/isopropanol (90:10) as the mobile phase (column pressure = 42 bar, flow rate = 1 ml/min).

HPLC of *Rac*-**3a** and **3a**

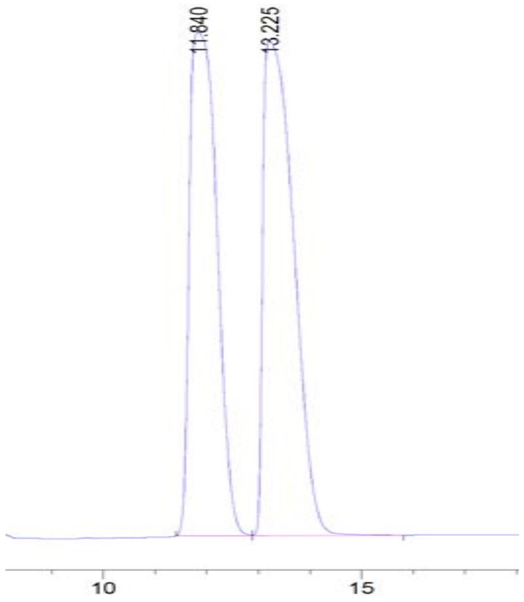

峰 #	保留时间 [min]	类型	峰宽 [min]	峰面积 mAU *s	峰高 [mAU]	峰面积 %
1	11.840	BV	0.5707	8.04917e4	2179.44409	47.8321
2	13.225	VB	0.6683	8.77879e4	2137.91235	52.1679

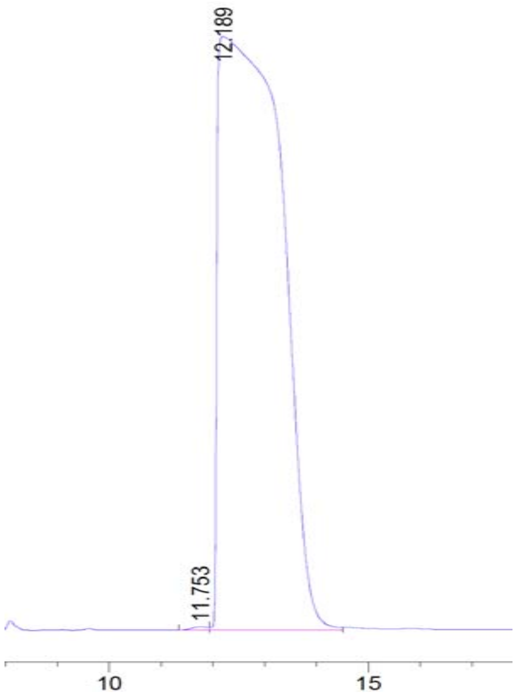

峰 #	保留时间 [min]	类型	峰宽 [min]	峰面积 mAU *s	峰高 [mAU]	峰面积 %
1	11.753	BV	0.3108	270.64417	13.26651	0.1370
2	12.189	VV	1.0701	1.97324e5	2332.09009	99.8630

HPLC of *Rac*-**3s** and **3s**

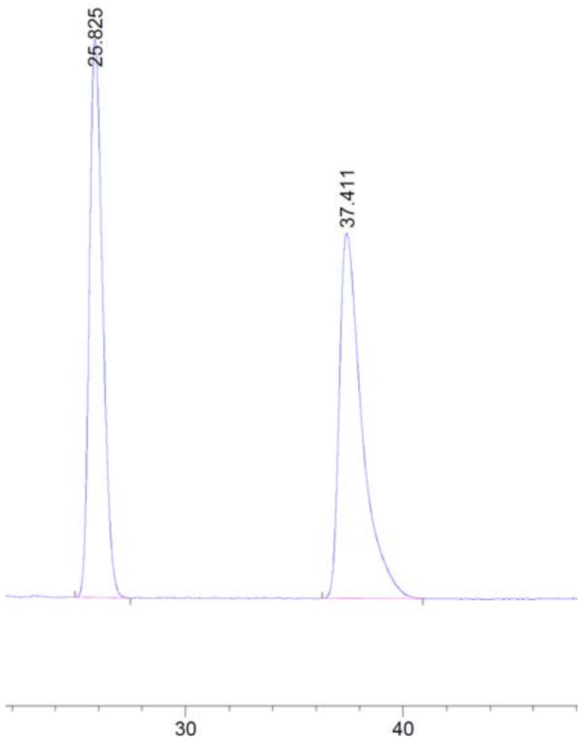

峰 #	保留时间 [min]	类型	峰宽 [min]	峰面积 mAU *s	峰高 [mAU]	峰面积 %
1	25.825	BB	0.6678	5856.63330	137.35669	47.2943
2	37.411	BB	1.0590	6526.75635	89.81219	52.7057

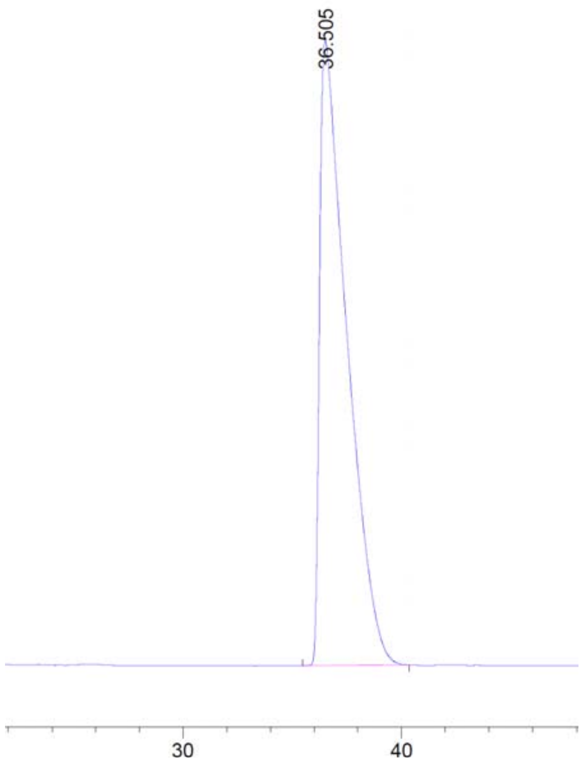

峰 #	保留时间 [min]	类型	峰宽 [min]	峰面积 mAU *s	峰高 [mAU]	峰面积 %
1	36.505	BB	1.2094	3.08124e4	354.92783	100.0000

HPLC of *Rac*-**3w** and **3w**

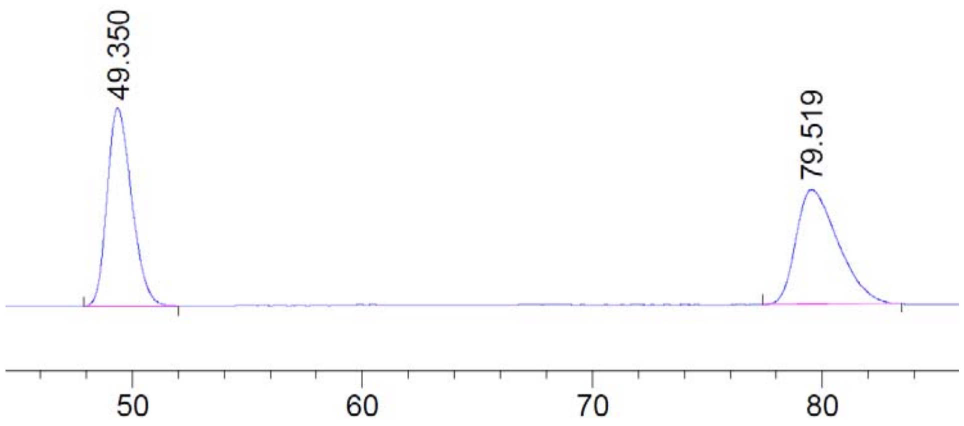

峰 #	保留时间 [min]	类型	峰宽 [min]	峰面积 mAU *s	峰高 [mAU]	峰面积 %
1	49. 350	BB	1. 1465	4584. 55664	61. 59241	50. 2292
2	79. 519	BB	1. 7249	4542. 72021	35. 73280	49. 7708

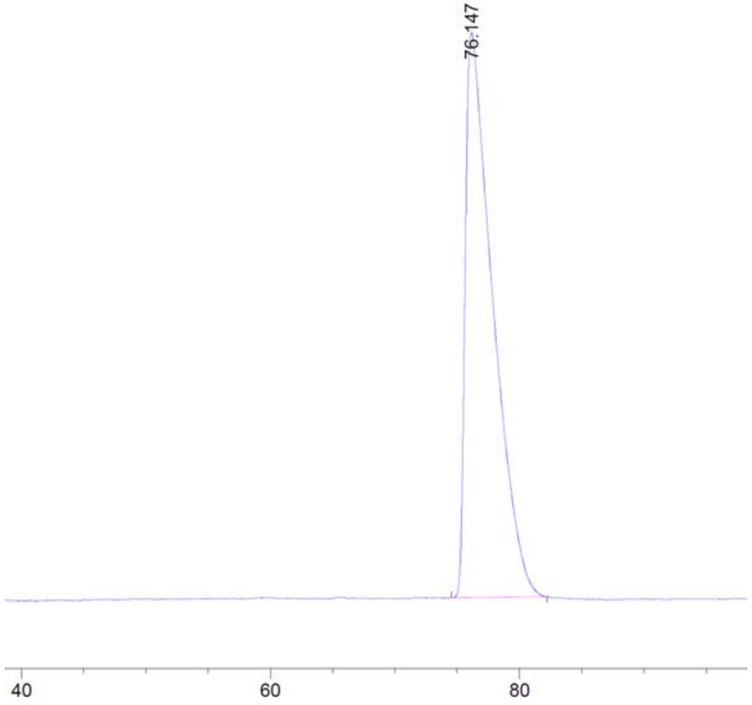

峰 #	保留时间 [min]	类型	峰宽 [min]	峰面积 mAU *s	峰高 [mAU]	峰面积 %
1	76.147	BB	2.0697	2.20859e4	144.05566	100.0000

References

1. Englund, E. A., Gopi, H. N. & Appella, D. H. *Org. Lett.* **6**, 213-215 (2004).
2. Kachkovskyi, G., Faderl, C. & Reiser, O. *Adv. Synth. Catal.* **355**, 2440-2248 (2013).
3. Cheng, S. & Yu, S. *Org. Biomol. Chem.* **12**, 8607-8610 (2014).
4. Yang, J., Zhang, J., Qi, L., Hu, C. & Chen, Y. *Chem. Comm.* **51**, 5275-5278 (2015).
5. Nair, V., Augustine, A. & Suja, T. D. *Synthesis* 2259-2265 (2014).
6. Leifert, D., Daniliuc, C. G. & Studer, A. *Org. Lett.* **15**, 6286-6289 (2013).

The ^{1}H and ^{13}C NMR spectra of compounds 1, 3, 5, 7, 9, *Rac*-**3a**, *Rac*-**3s** and *Rac*-**3w**

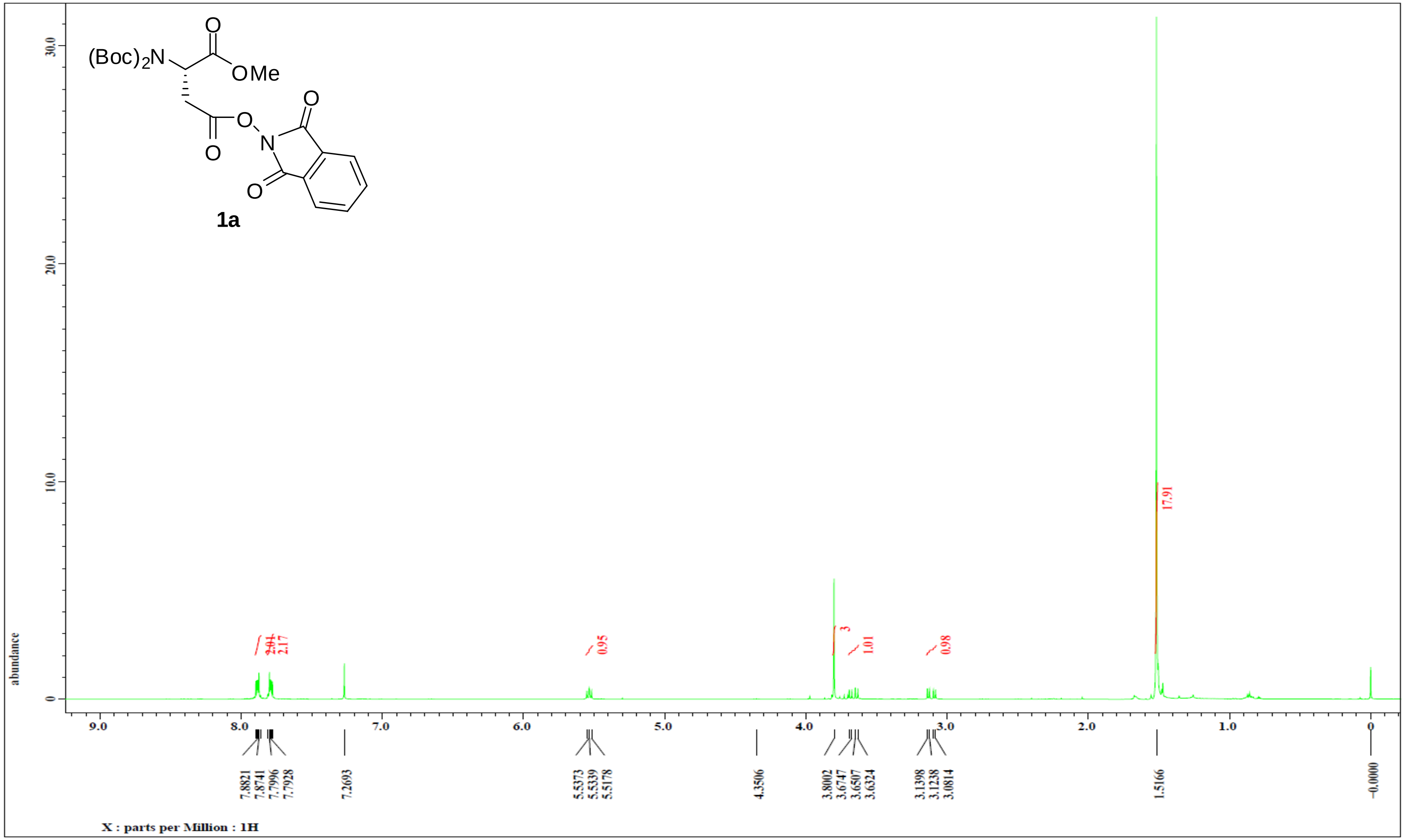

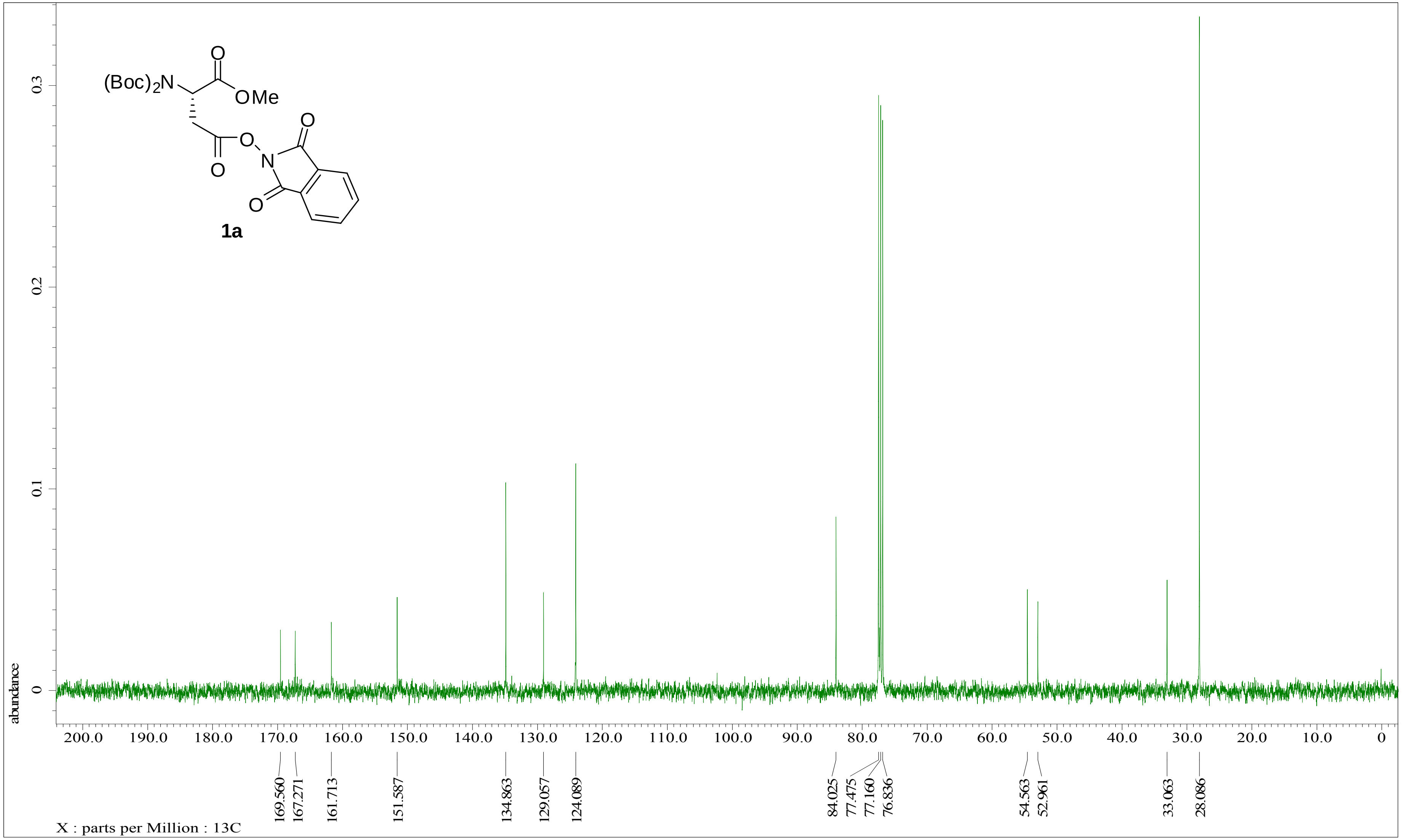
(Boc)2N
OMe
1a
abundance
0.3
0.2
0.1
0
200.0 190.0 180.0 170.0 160.0 150.0 140.0 130.0 120.0 110.0 100.0 90.0 80.0 70.0 60.0 50.0 40.0 30.0 20.0 10.0 0
169.560
167.271
161.713
151.587
134.863
129.057
124.089
84.025
77.475
77.160
76.836
54.563
52.961
33.063
28.086
X : parts per Million : 13C

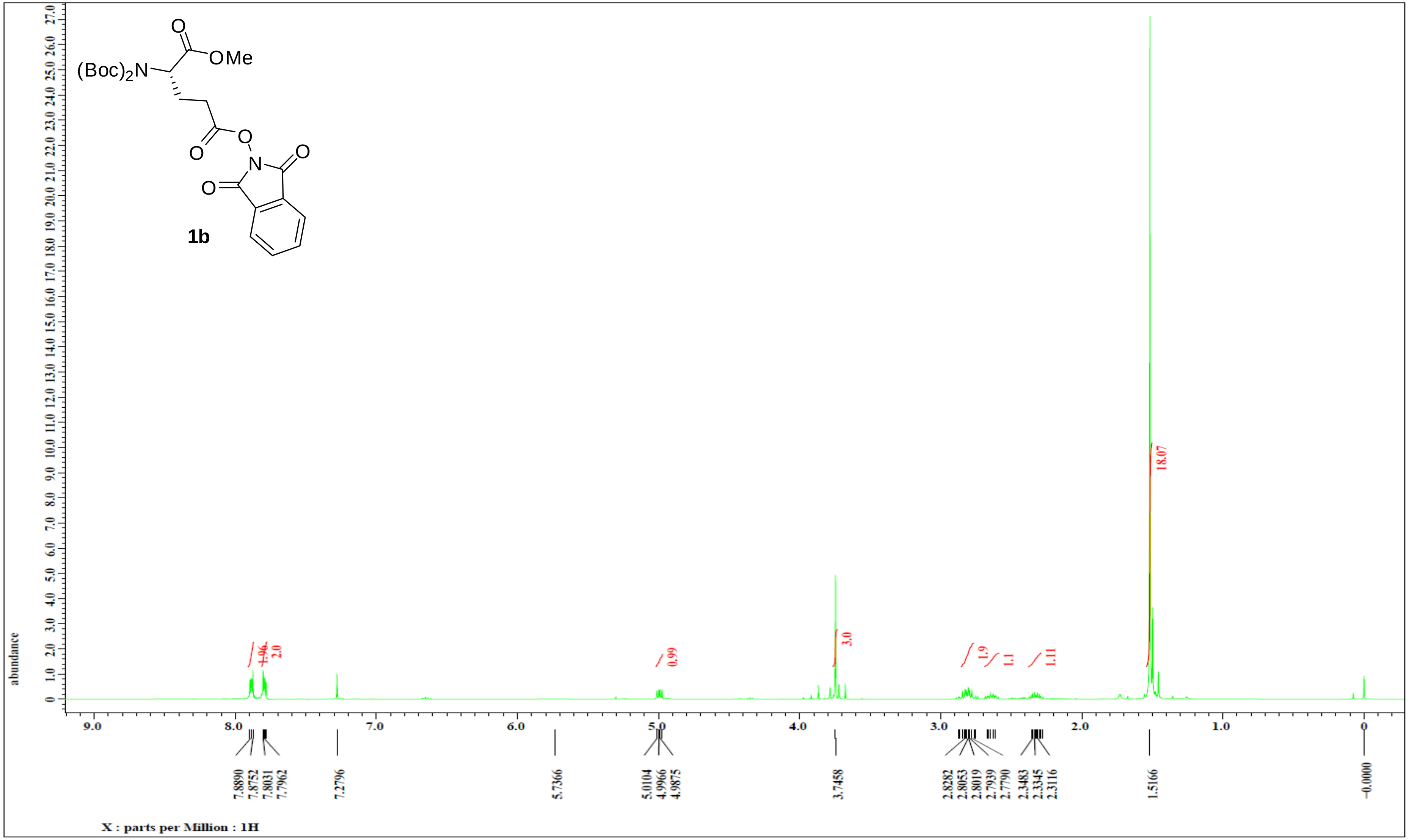
(Boc)2N
OMe
1b
abundance
X : parts per Million : 1H
18.07
3.0
0.99
1.96
2.0
1.9
1.1
1.11
7.8890
7.8752
7.8031
7.7962
7.2796
5.7366
5.0104
4.9966
4.9875
3.7458
2.8282
2.8053
2.8019
2.7939
2.7790
2.3483
2.3345
2.3116
1.5166
-0.0000

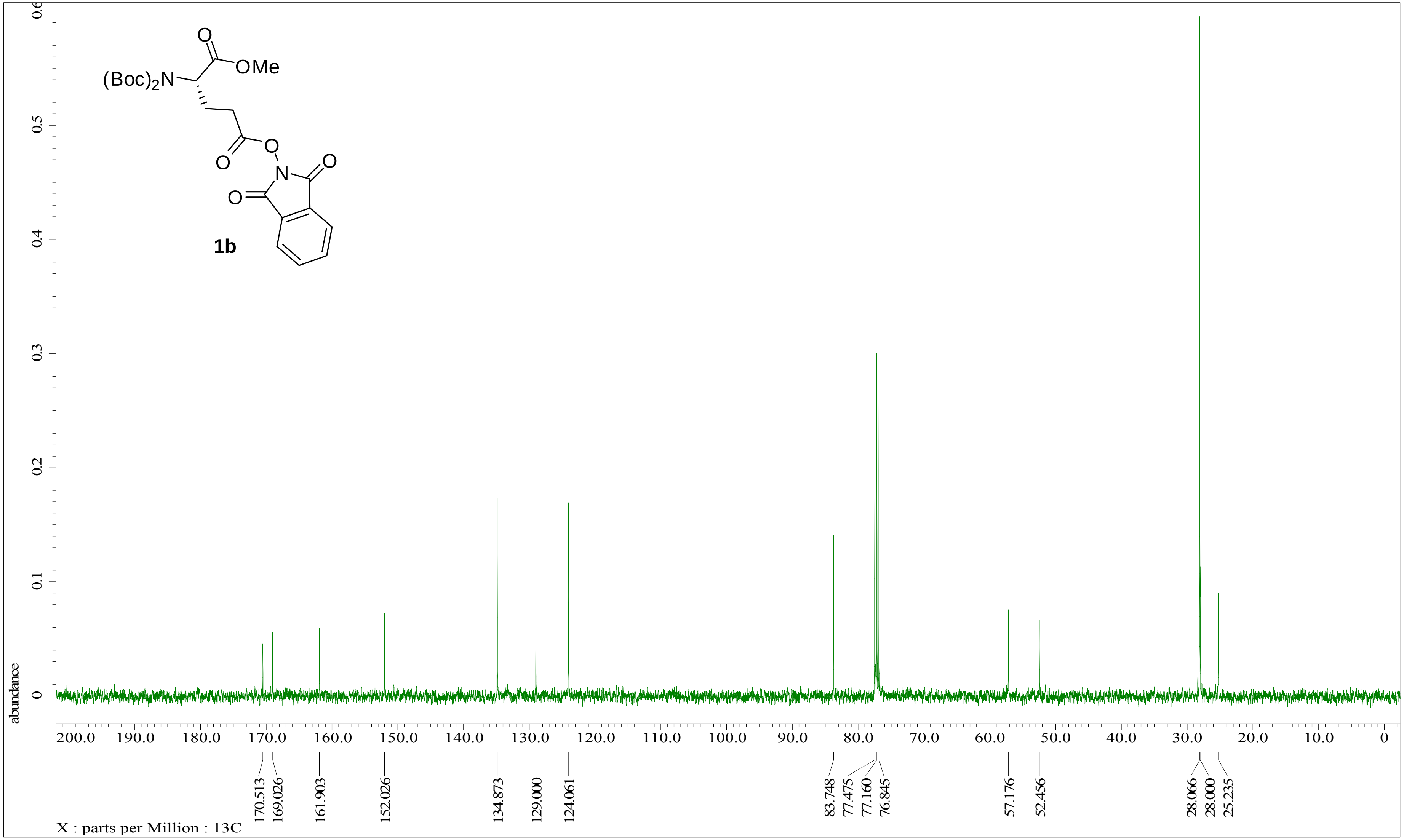
(Boc)2N
OMe
O
N
1b
abundance
X : parts per Million : 13C
170.513
169.026
161.903
152.026
134.873
129.000
124.061
83.748
77.475
77.160
76.845
57.176
52.456
28.066
28.000
25.235

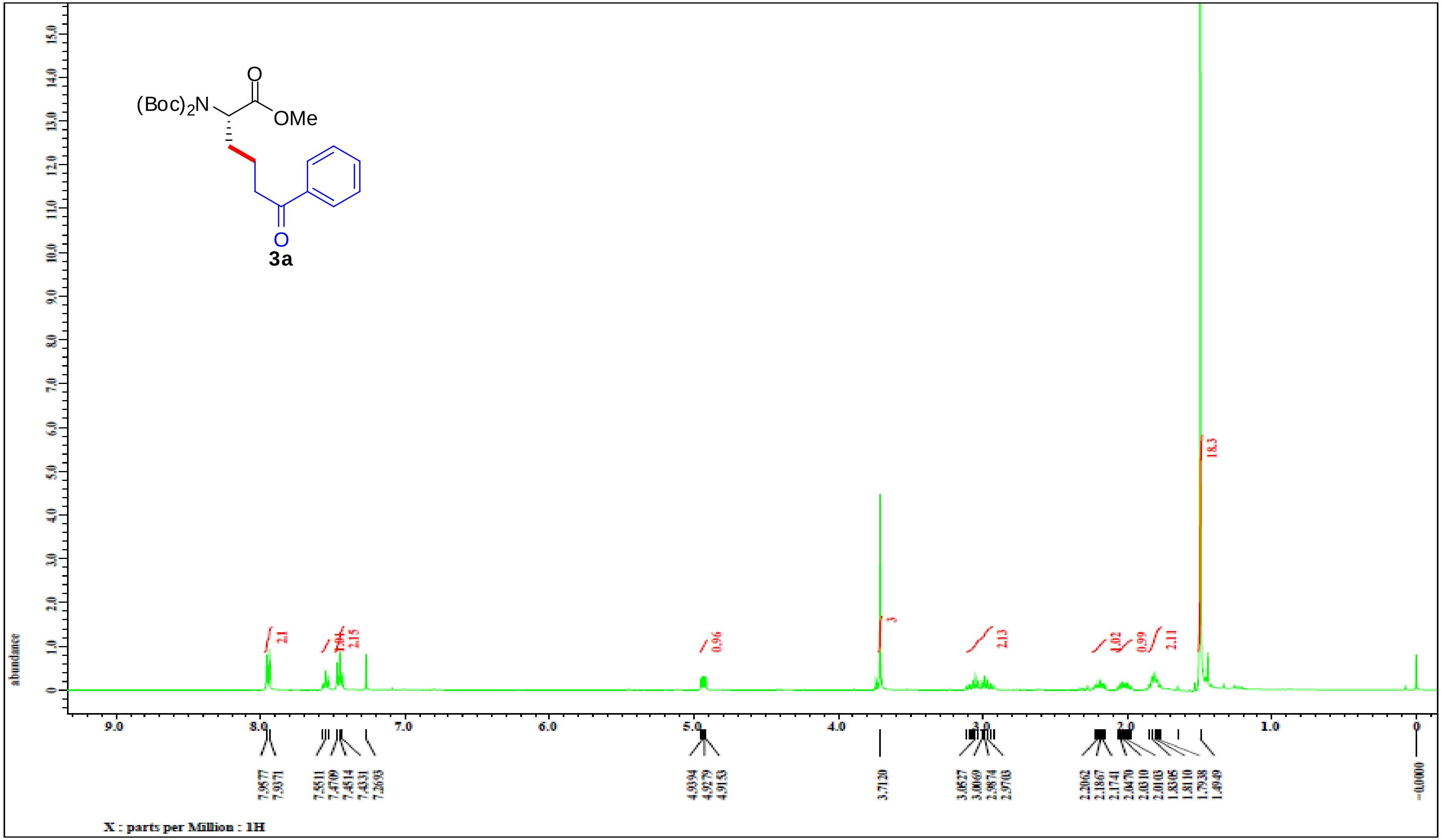
(Boc)2N
O
OMe
O
3a
abundance
X : parts per Million : 1H
7.9577
7.9371
7.5511
7.4709
7.4514
7.4331
7.2693
4.9394
4.9279
4.9153
3.7120
3.0527
3.0069
2.9874
2.9703
2.2062
2.1867
2.1741
2.0470
2.0310
2.0103
1.8305
1.8110
1.7938
1.4949
-0.0000
2.1
2.15
0.96
3
2.13
1.02
0.99
2.11
18.3

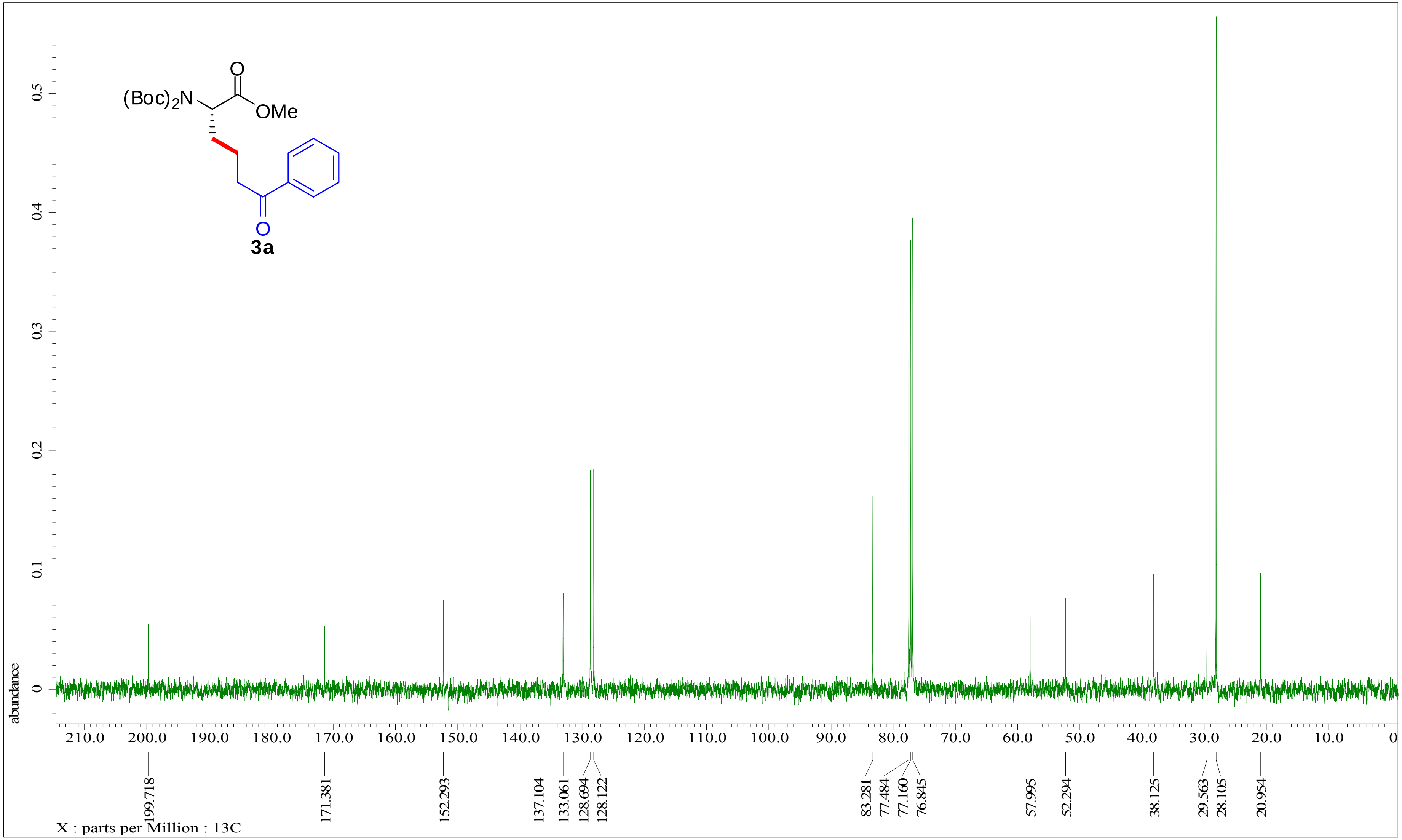
(Boc)2N
O
OMe
O
3a
abundance
0
0.1
0.2
0.3
0.4
0.5
210.0 200.0 190.0 180.0 170.0 160.0 150.0 140.0 130.0 120.0 110.0 100.0 90.0 80.0 70.0 60.0 50.0 40.0 30.0 20.0 10.0 0
199.718
171.381
152.293
137.104
133.061
128.694
128.122
83.281
77.484
77.160
76.845
57.995
52.294
38.125
29.563
28.105
20.954
X : parts per Million : 13C

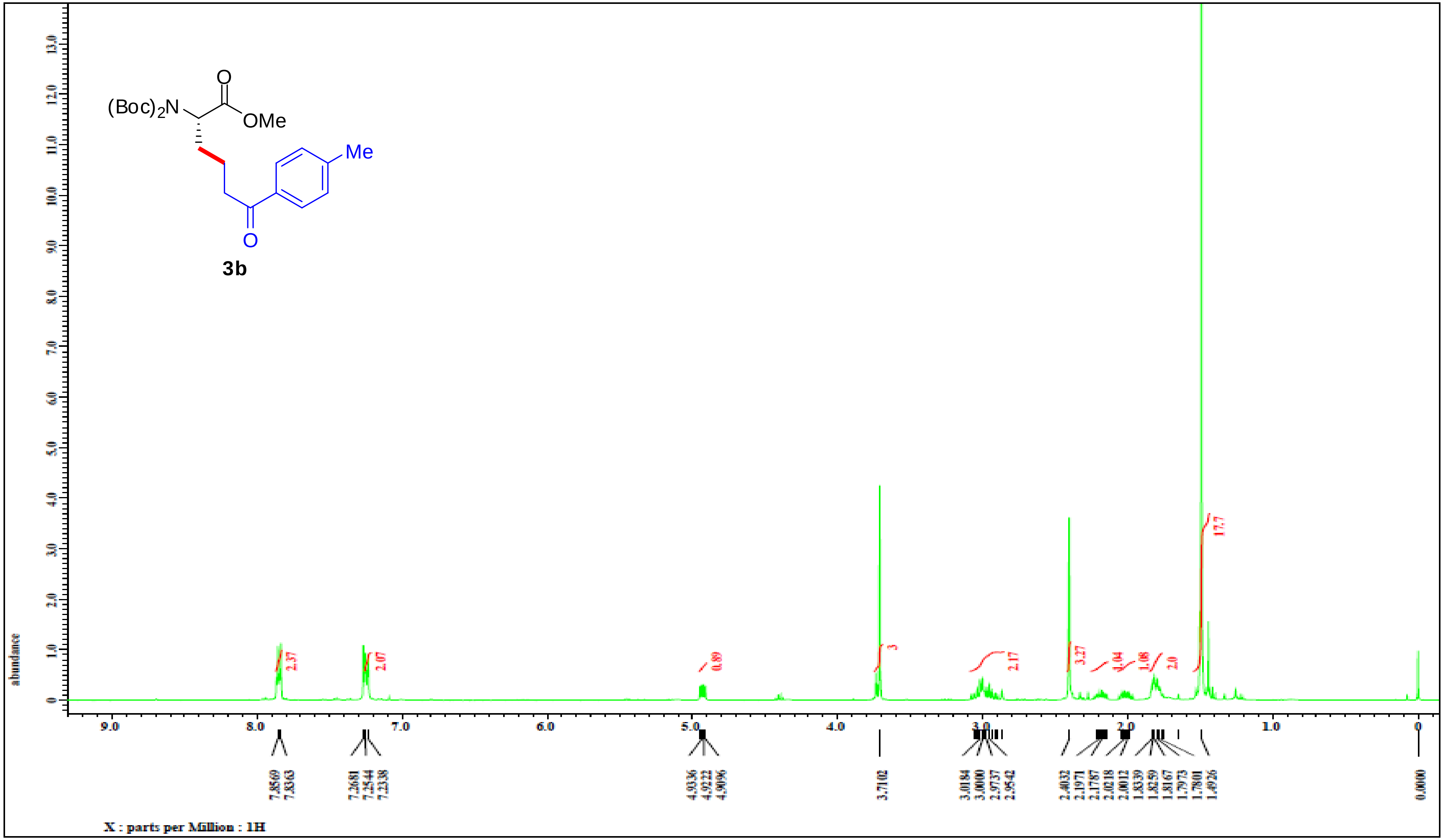

(Boc)2N
O
OMe
Me
O
3b
abundance
X : parts per Million : 1H
7.8569
7.8363
7.2681
7.2544
7.2338
4.9336
4.9222
4.9096
3.7102
3.0184
3.0000
2.9737
2.9542
2.4032
2.1971
2.1787
2.0218
2.0012
1.8339
1.8259
1.8167
1.7973
1.7801
1.4926
0.0000
2.37
2.07
0.89
3
2.17
3.27
1.04
1.08
2.0
17.7

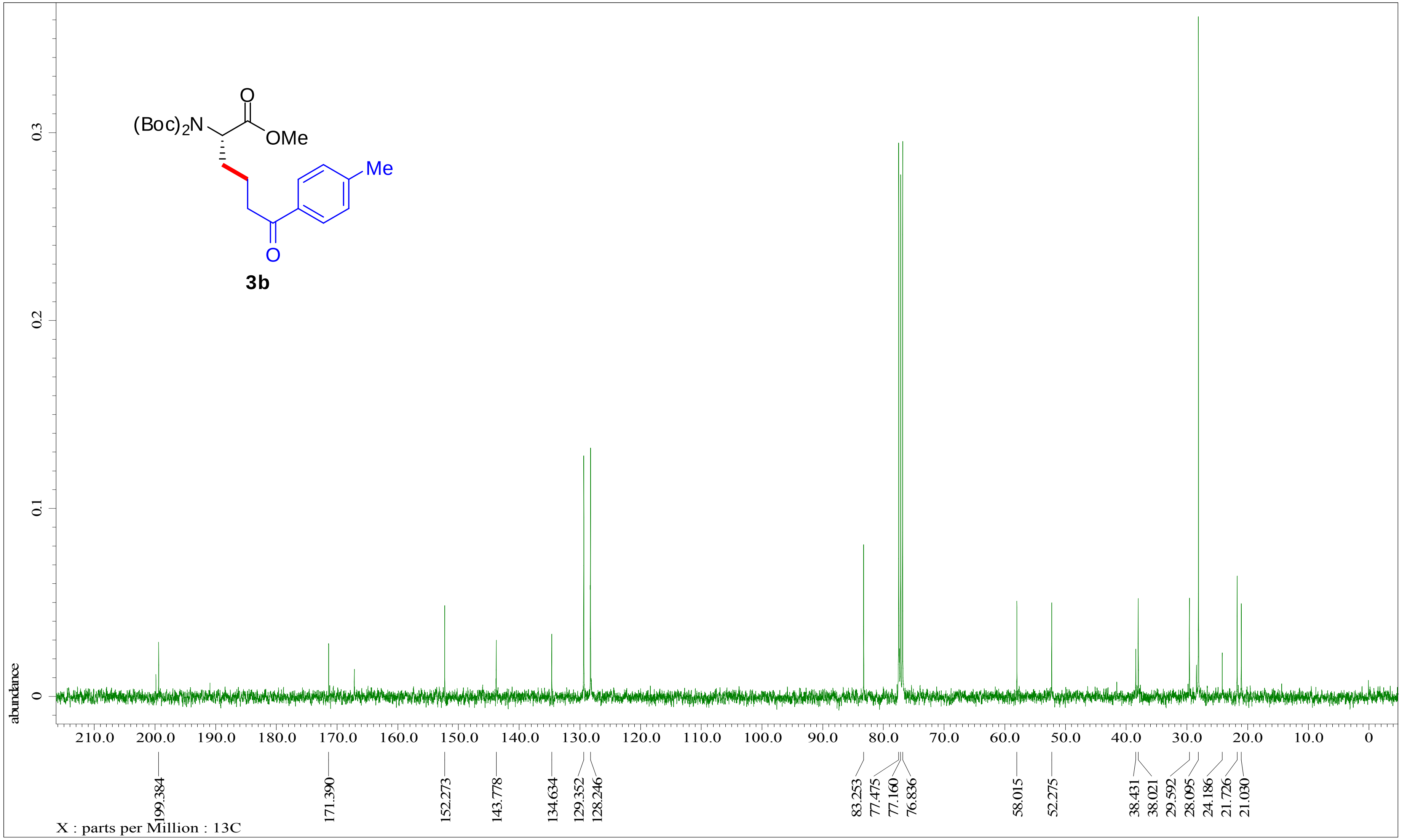
(Boc)2N
O
OMe
Me
O
3b
abundance
0
0.1
0.2
0.3
210.0
200.0
190.0
180.0
170.0
160.0
150.0
140.0
130.0
120.0
110.0
100.0
90.0
80.0
70.0
60.0
50.0
40.0
30.0
20.0
10.0
0
199.384
171.390
152.273
143.778
134.634
129.352
128.246
83.253
77.475
77.160
76.836
58.015
52.275
38.431
38.021
29.592
28.095
24.186
21.726
21.030
X : parts per Million : 13C

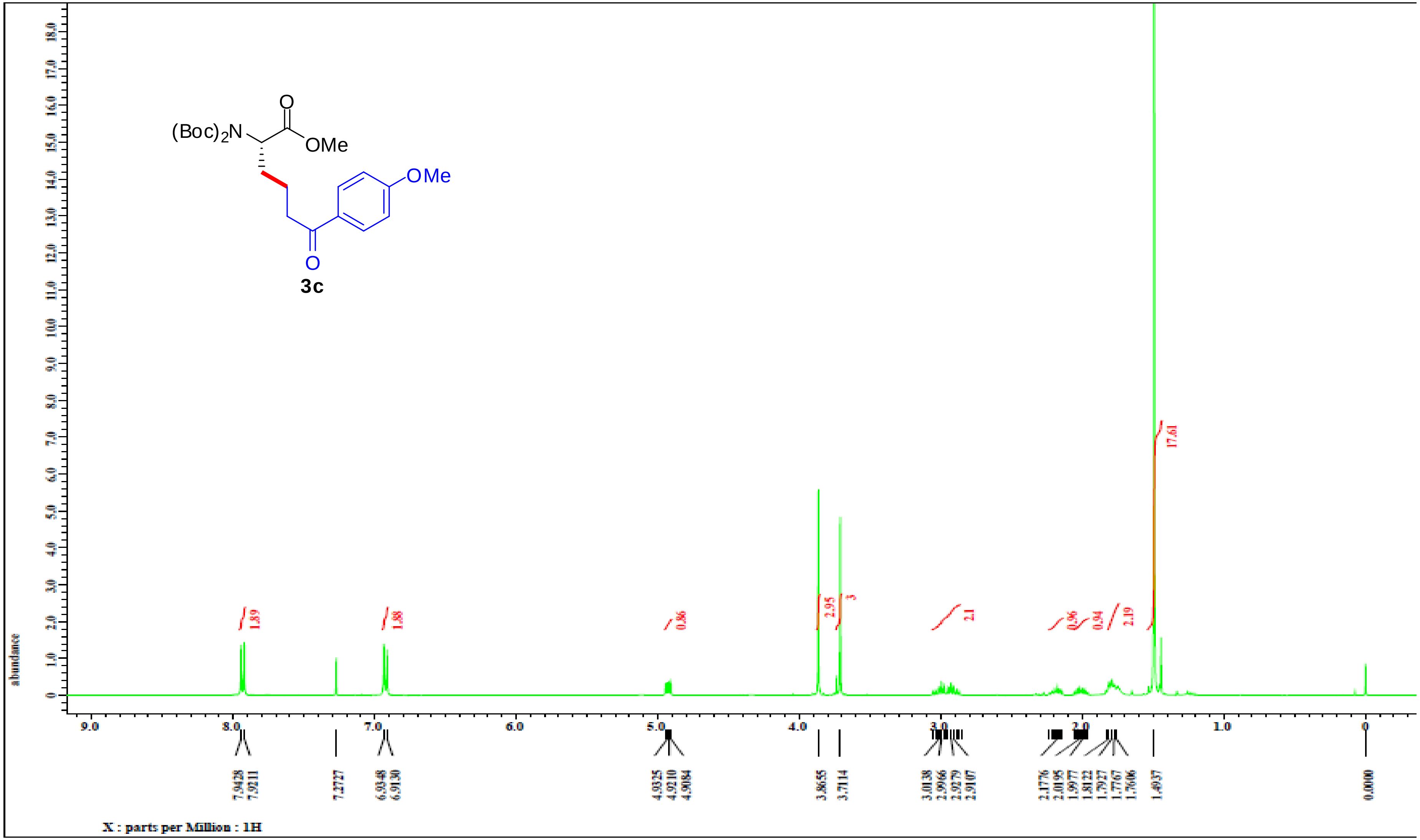

(Boc)$_2$N
OMe
OMe
3c
X : parts per Million : 1H
abundance

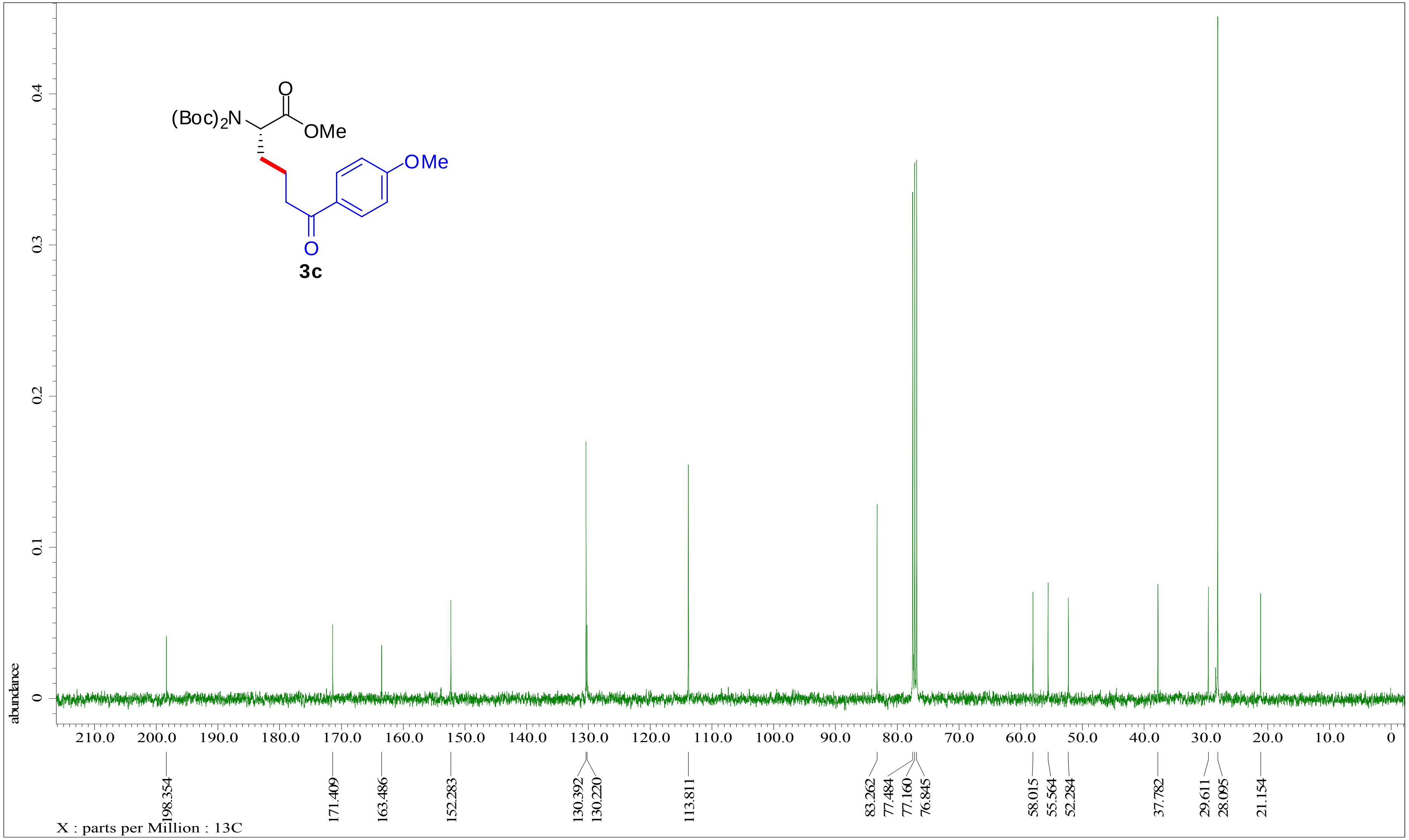

(Boc)2N
O
OMe
OMe
O
3c
abundance
0.4
0.3
0.2
0.1
0
210.0 200.0 190.0 180.0 170.0 160.0 150.0 140.0 130.0 120.0 110.0 100.0 90.0 80.0 70.0 60.0 50.0 40.0 30.0 20.0 10.0 0
198.354
171.409
163.486
152.283
130.392
130.220
113.811
83.262
77.484
77.160
76.845
58.015
55.564
52.284
37.782
29.611
28.095
21.154
X : parts per Million : 13C

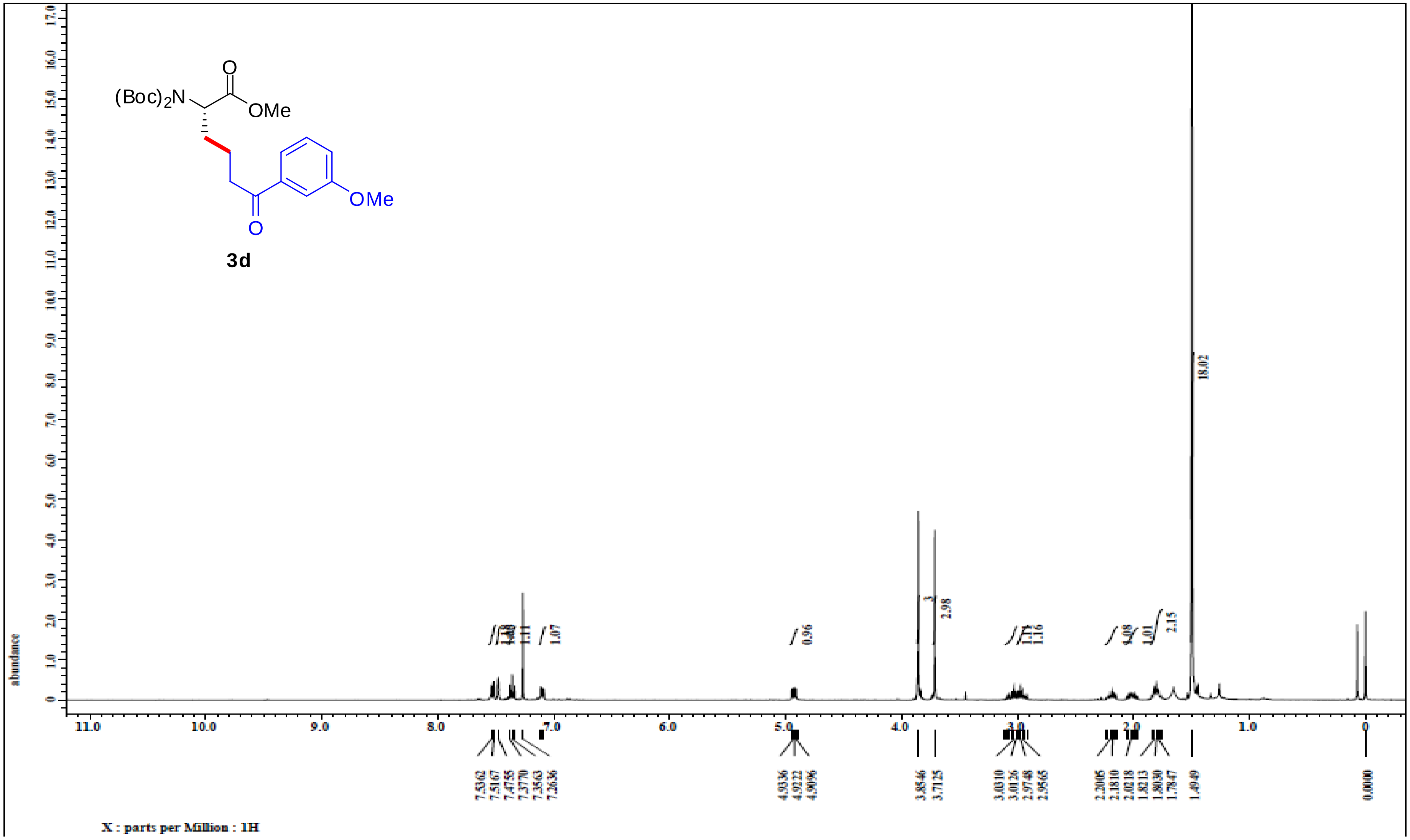

(Boc)2N
OMe
O
OMe
3d
abundance
18.02
X : parts per Million : 1H

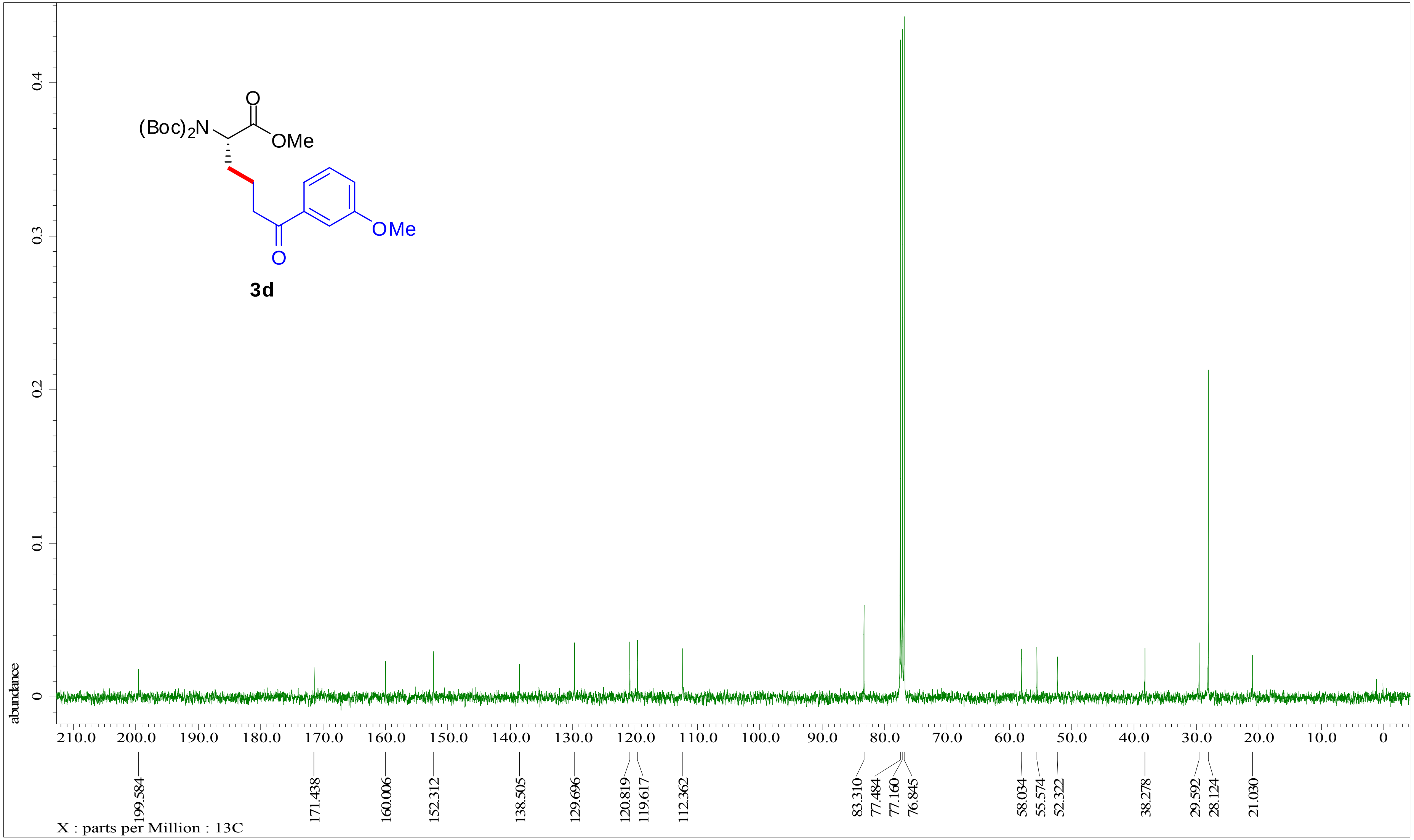
(Boc)2N
OMe
O
OMe
O
3d
abundance
0.4
0.3
0.2
0.1
0
210.0
200.0
190.0
180.0
170.0
160.0
150.0
140.0
130.0
120.0
110.0
100.0
90.0
80.0
70.0
60.0
50.0
40.0
30.0
20.0
10.0
0
199.584
171.438
160.006
152.312
138.505
129.696
120.819
119.617
112.362
83.310
77.484
77.160
76.845
58.034
55.574
52.322
38.278
29.592
28.124
21.030
X : parts per Million : 13C

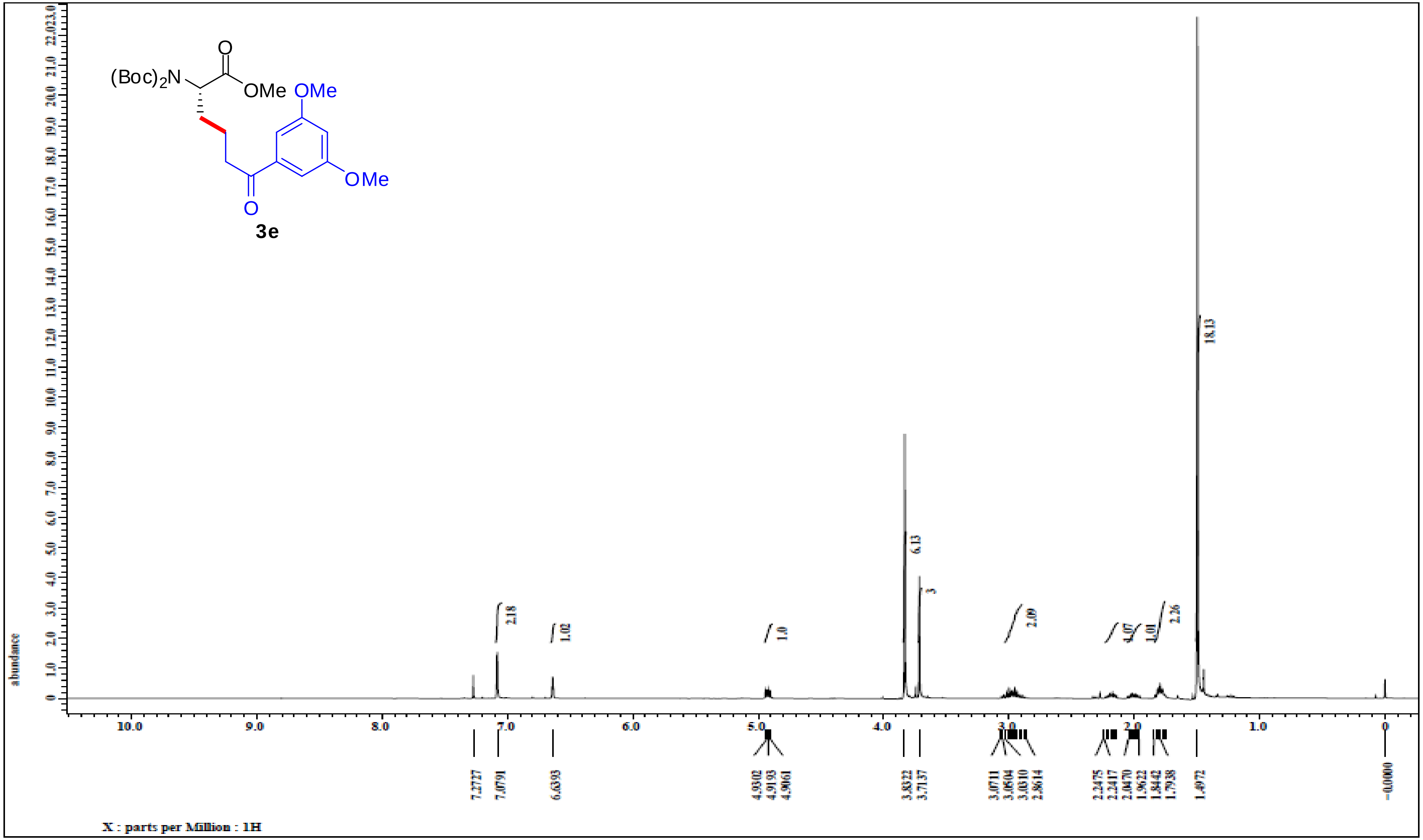
(Boc)2N
OMe
OMe
OMe
O
O
3e
abundance
X : parts per Million : 1H
2.18
1.02
1.0
6.13
3
2.09
4.07
1.01
2.26
18.13
7.2727
7.0791
6.6393
4.9302
4.9193
4.9061
3.8322
3.7137
3.0711
3.0504
3.0310
2.8614
2.2475
2.2417
2.0470
1.9622
1.8442
1.7938
1.4972
-0.0000

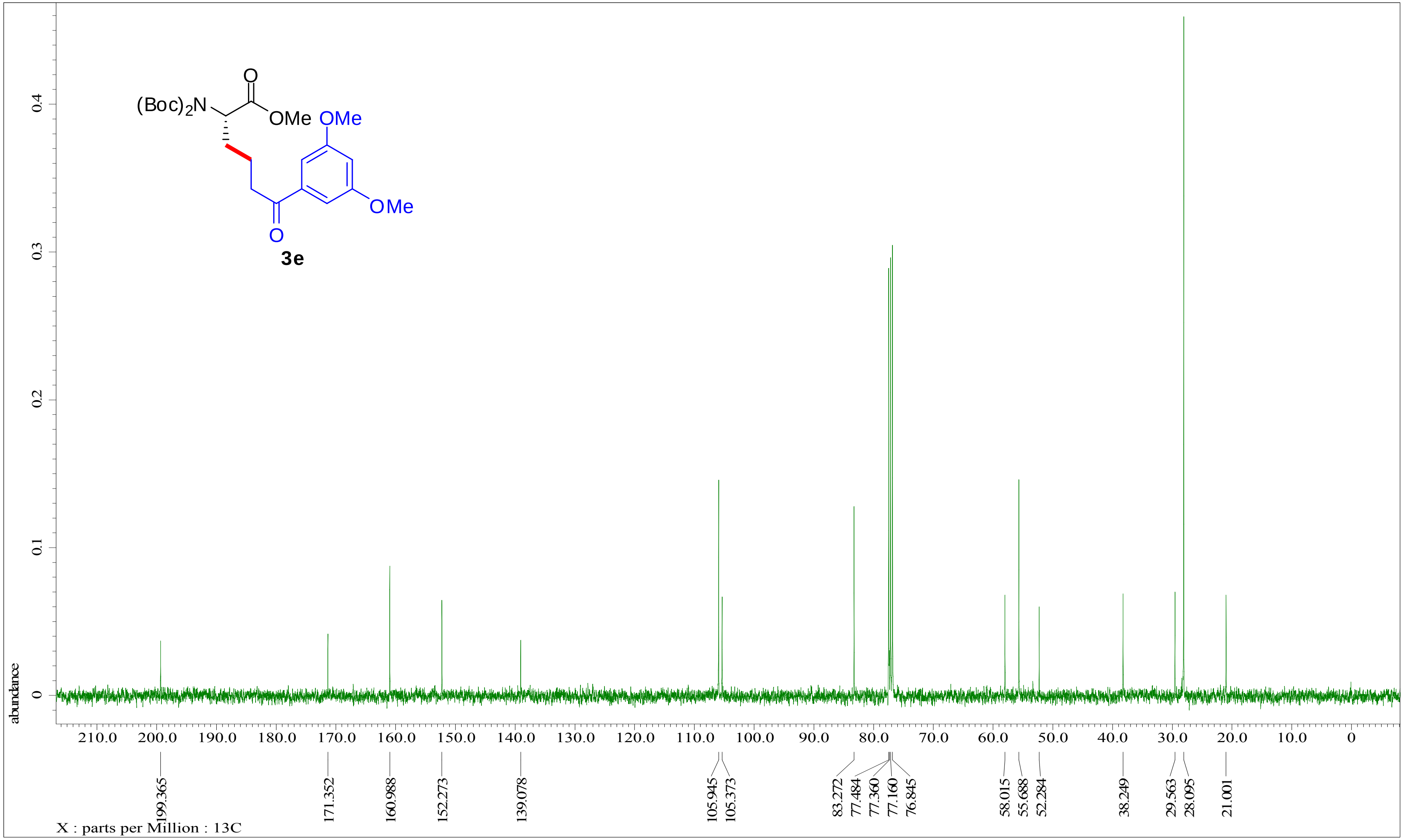

(Boc)2N
O
OMe
OMe
OMe
O
3e
abundance
0
0.1
0.2
0.3
0.4
210.0
200.0
190.0
180.0
170.0
160.0
150.0
140.0
130.0
120.0
110.0
100.0
90.0
80.0
70.0
60.0
50.0
40.0
30.0
20.0
10.0
0
199.365
171.352
160.988
152.273
139.078
105.945
105.373
83.272
77.484
77.360
77.160
76.845
58.015
55.688
52.284
38.249
29.563
28.095
21.001
X : parts per Million : 13C

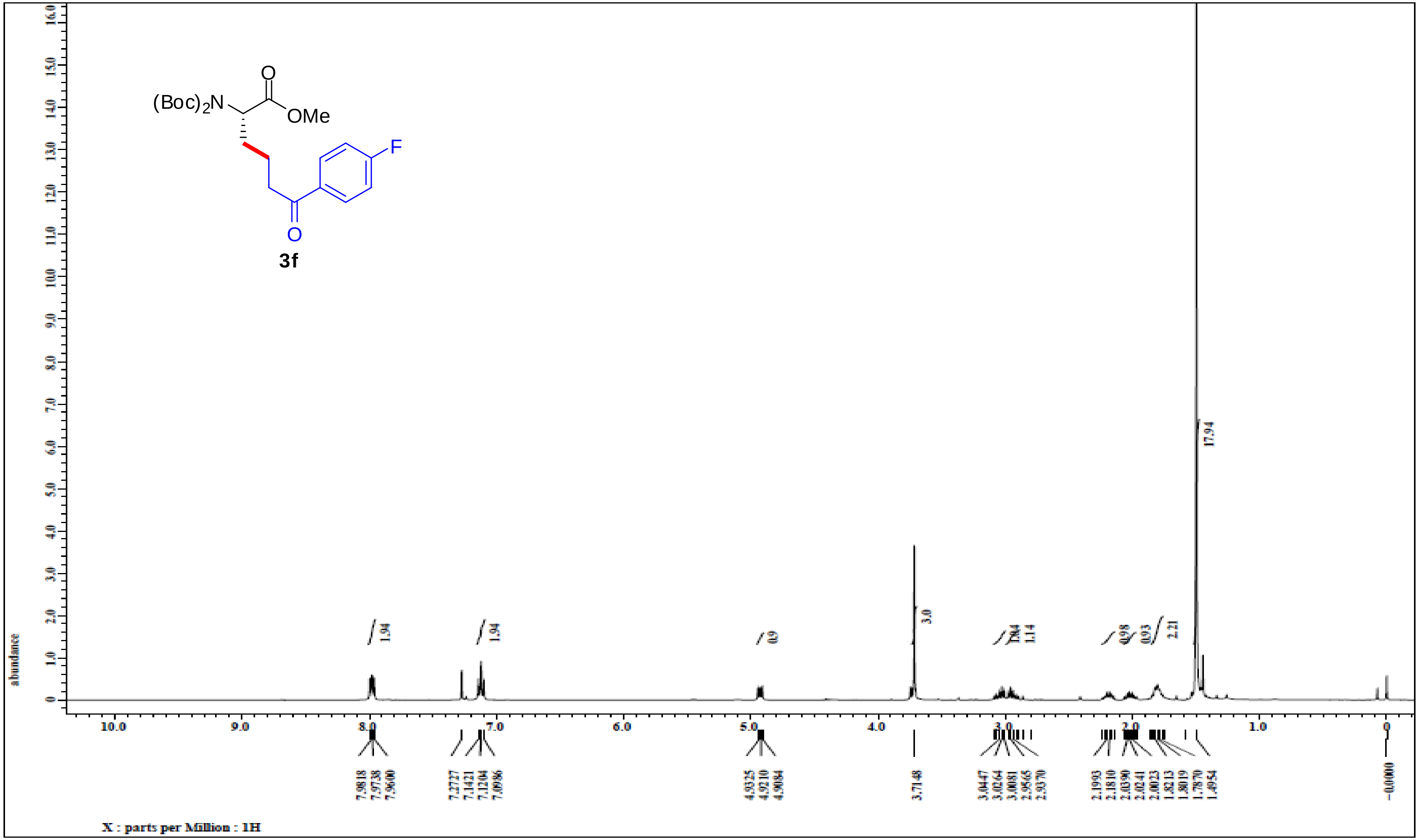

(Boc)2N
O
OMe
F
O
3f
abundance
X : parts per Million : 1H

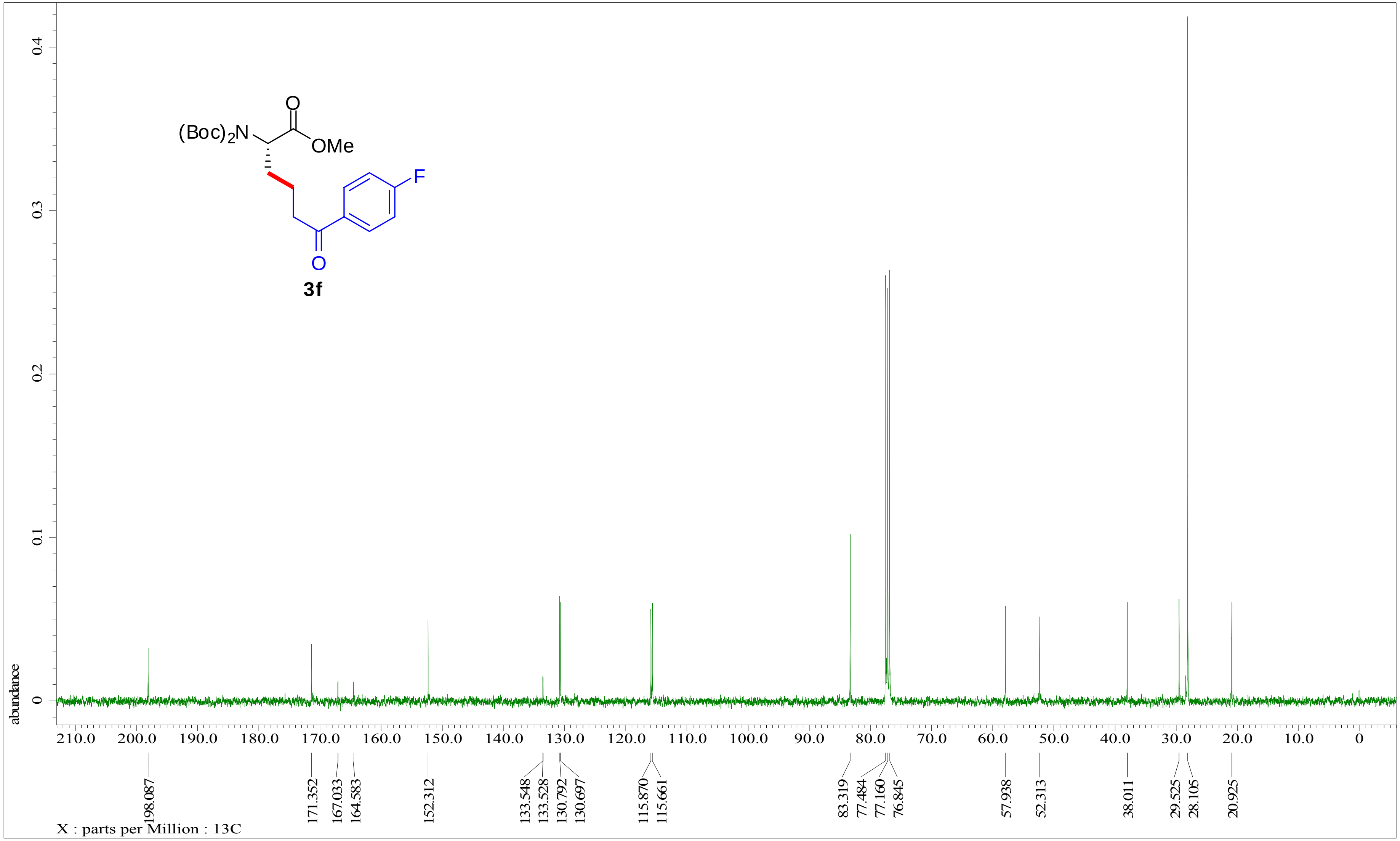
(Boc)2N
O
OMe
F
O
3f
abundance
0.4
0.3
0.2
0.1
0
210.0
200.0
190.0
180.0
170.0
160.0
150.0
140.0
130.0
120.0
110.0
100.0
90.0
80.0
70.0
60.0
50.0
40.0
30.0
20.0
10.0
0
198.087
171.352
167.033
164.583
152.312
133.548
133.528
130.792
130.697
115.870
115.661
83.319
77.484
77.160
76.845
57.938
52.313
38.011
29.525
28.105
20.925
X : parts per Million : 13C

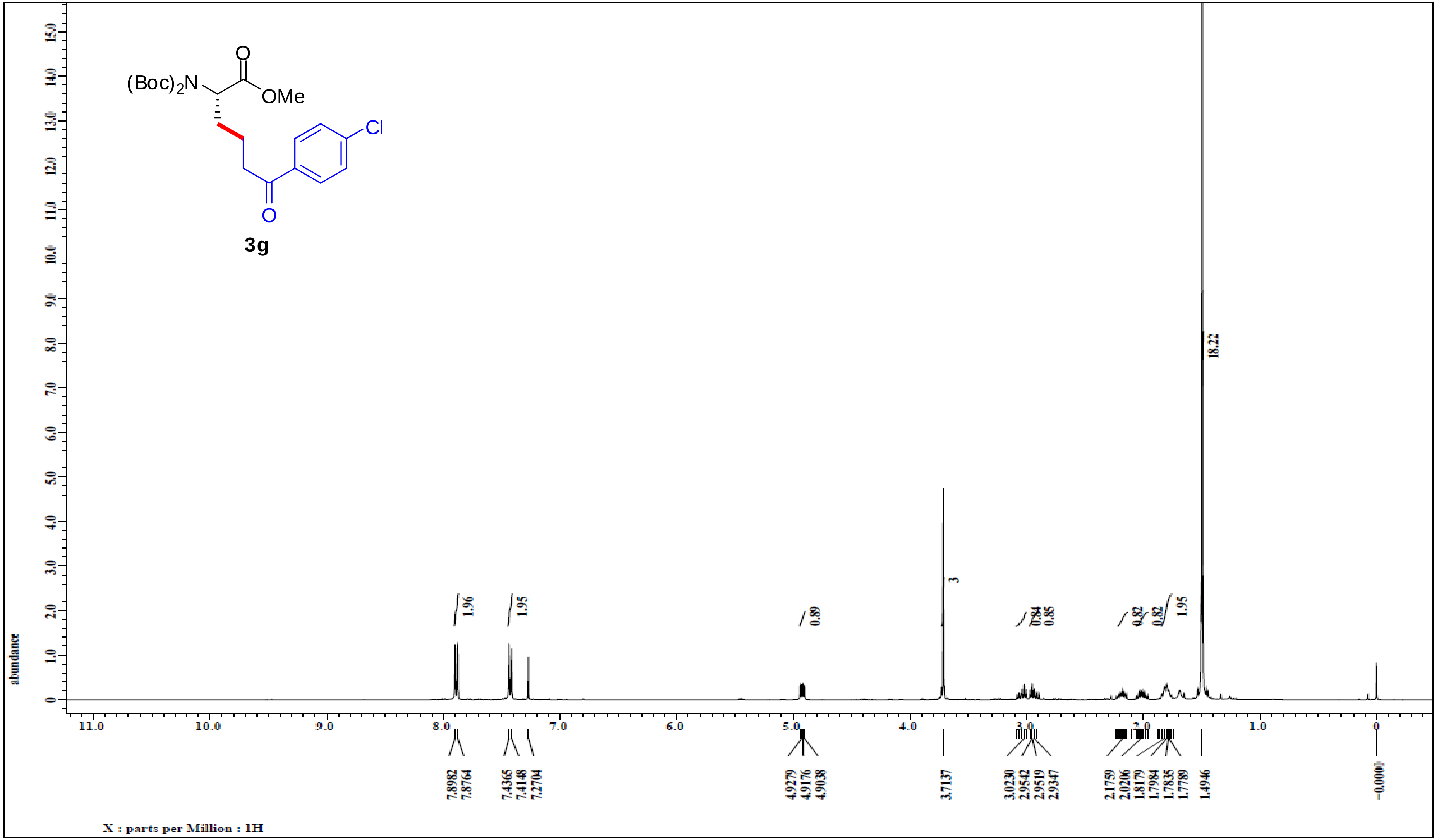
(Boc)2N
O
OMe
Cl
O
3g
abundance
X : parts per Million : 1H
7.8982
7.8764
7.4365
7.4148
7.2704
4.9279
4.9176
4.9038
3.7137
3.0230
2.9542
2.9519
2.9347
2.1759
2.0206
1.8179
1.7984
1.7835
1.7789
1.4946
-0.0000
1.96
1.95
0.89
3
0.84
0.85
0.82
0.82
1.95
18.22

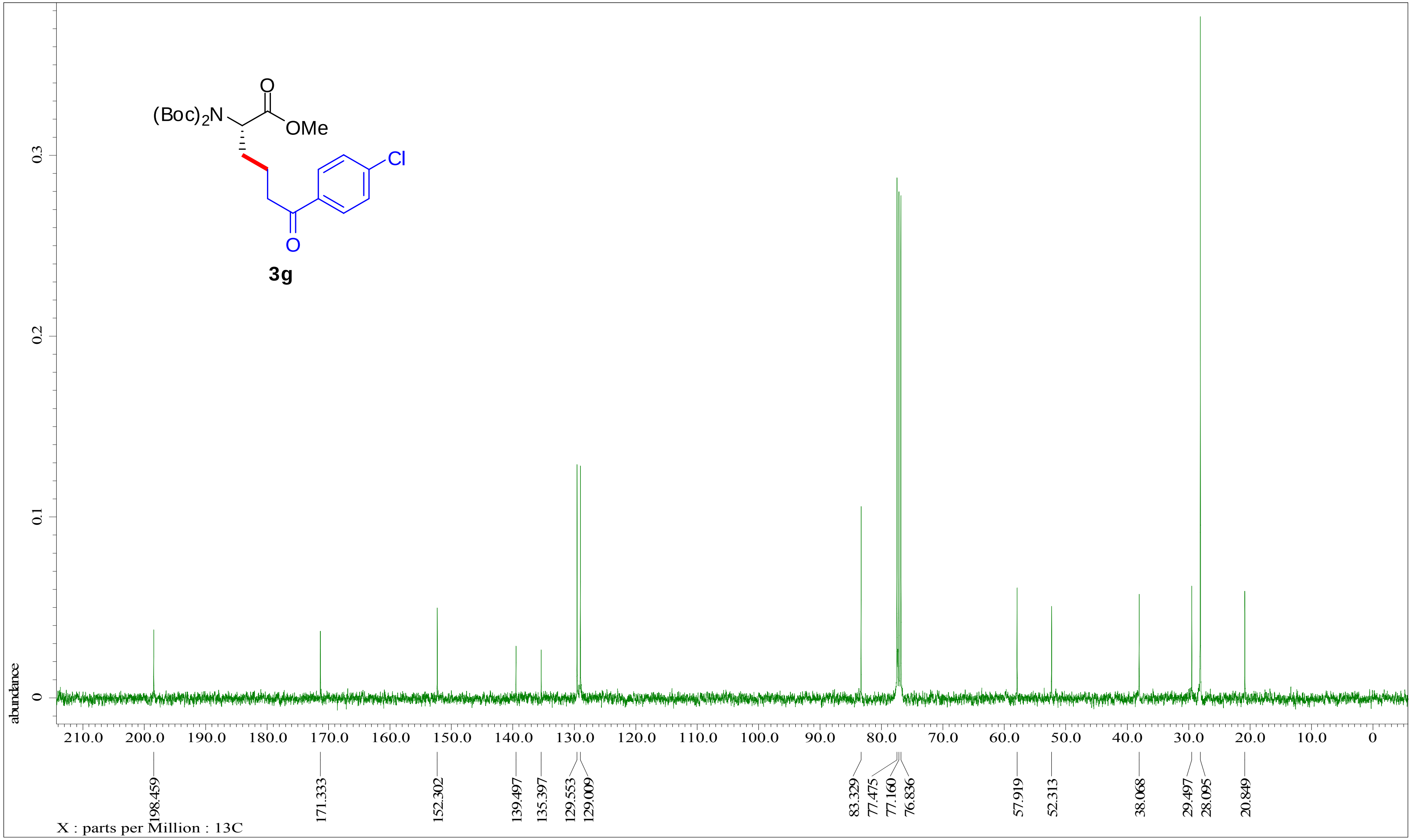
(Boc)2N
O
OMe
Cl
O
3g
abundance
0
0.1
0.2
0.3
210.0 200.0 190.0 180.0 170.0 160.0 150.0 140.0 130.0 120.0 110.0 100.0 90.0 80.0 70.0 60.0 50.0 40.0 30.0 20.0 10.0 0
198.459
171.333
152.302
139.497
135.397
129.553
129.009
83.329
77.475
77.160
76.836
57.919
52.313
38.068
29.497
28.095
20.849
X : parts per Million : 13C

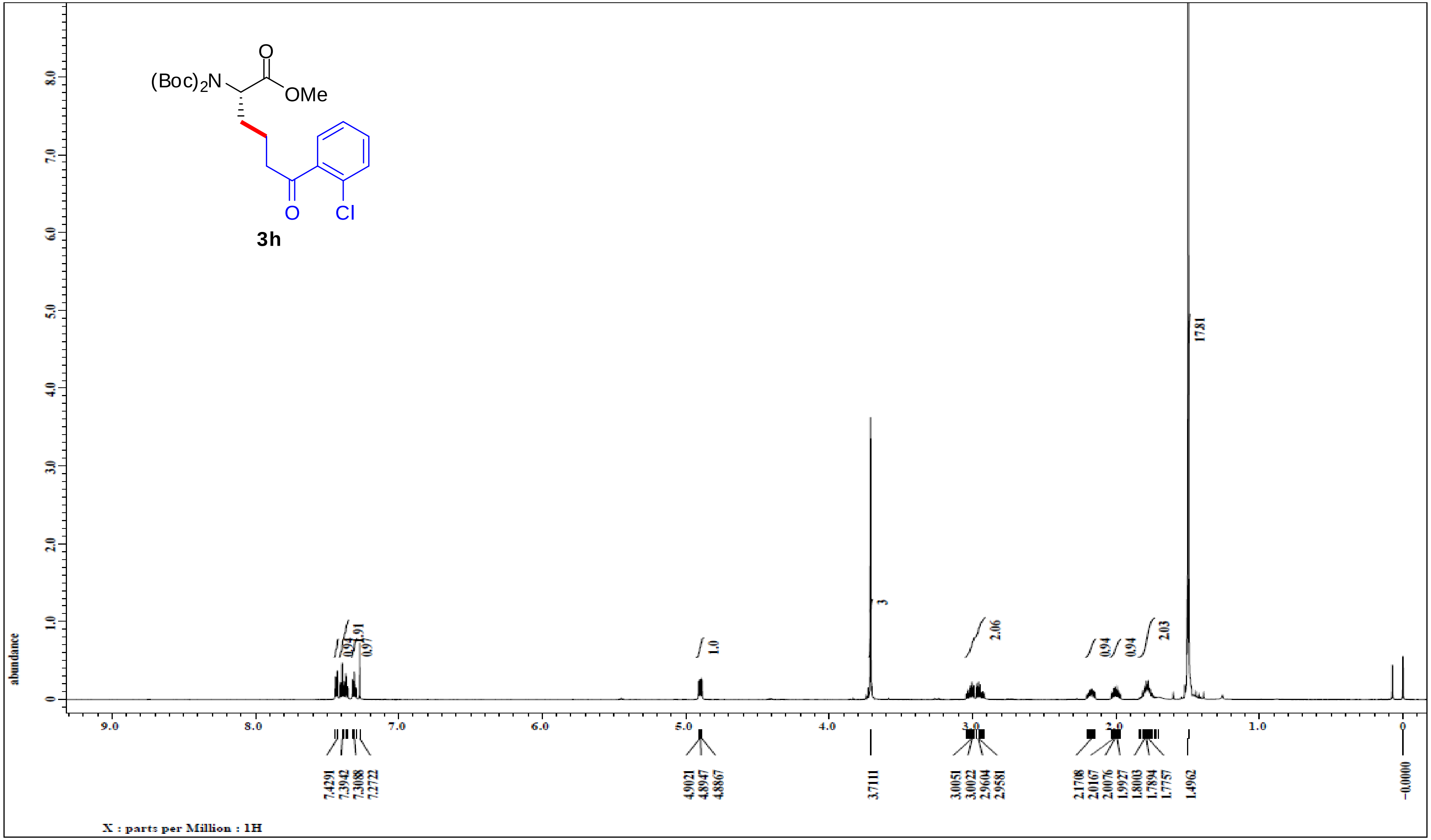
(Boc)2N
O
OMe
O
Cl
3h
abundance
X : parts per Million : 1H
9.0
8.0
7.0
6.0
5.0
4.0
3.0
2.0
1.0
0
17.81
3
2.06
2.03
0.94
0.94
1.0
0.91
0.97
7.4291
7.3942
7.3088
7.2722
4.9021
4.8947
4.8867
3.7111
3.0051
3.0022
2.9604
2.9581
2.1708
2.0167
2.0076
1.9927
1.8003
1.7894
1.7757
1.4962
-0.0000

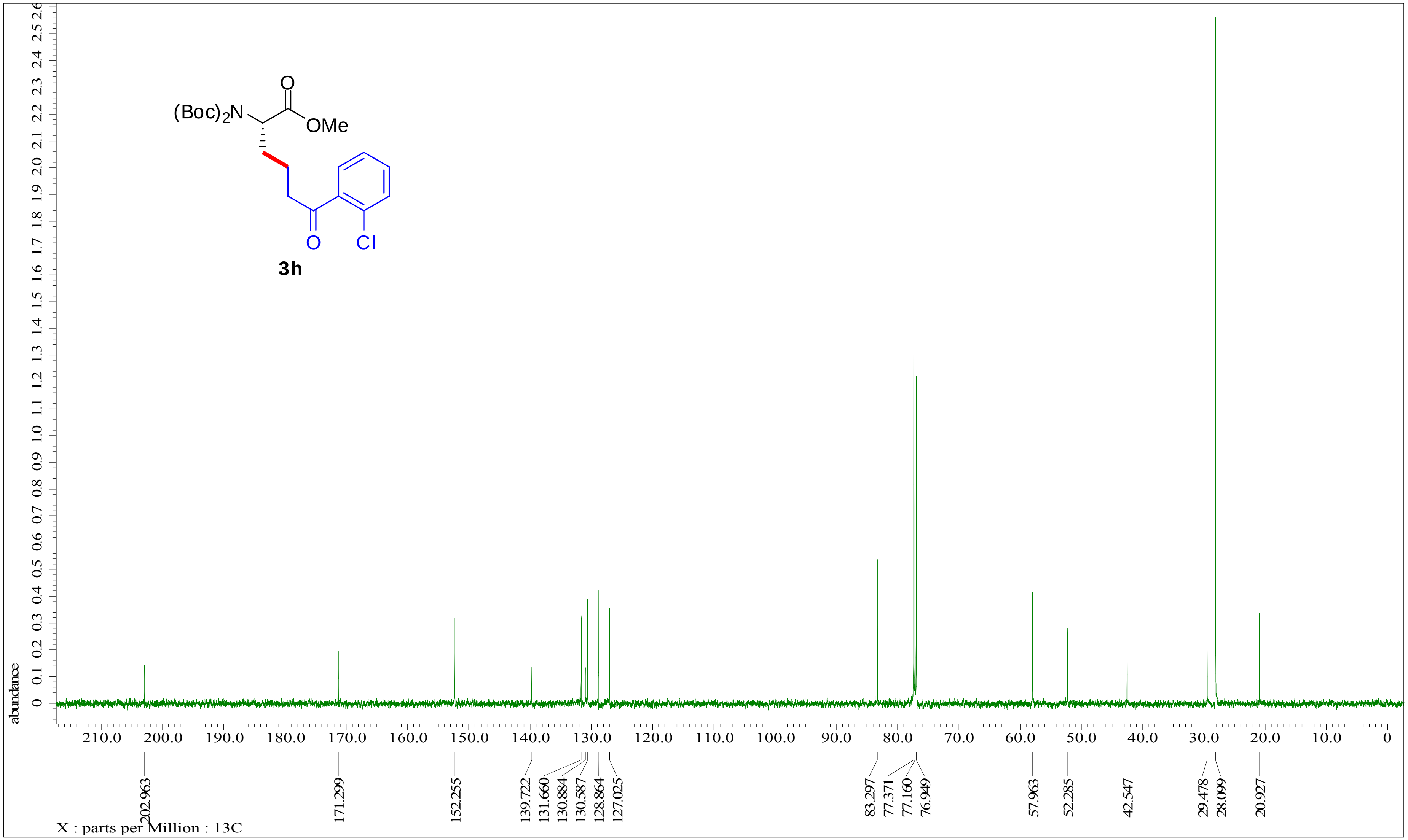

(Boc)2N
O
OMe
O
Cl
3h
abundance
X : parts per Million : 13C
202.963
171.299
152.255
139.722
131.660
130.884
130.587
128.864
127.025
83.297
77.371
77.160
76.949
57.963
52.285
42.547
29.478
28.099
20.927

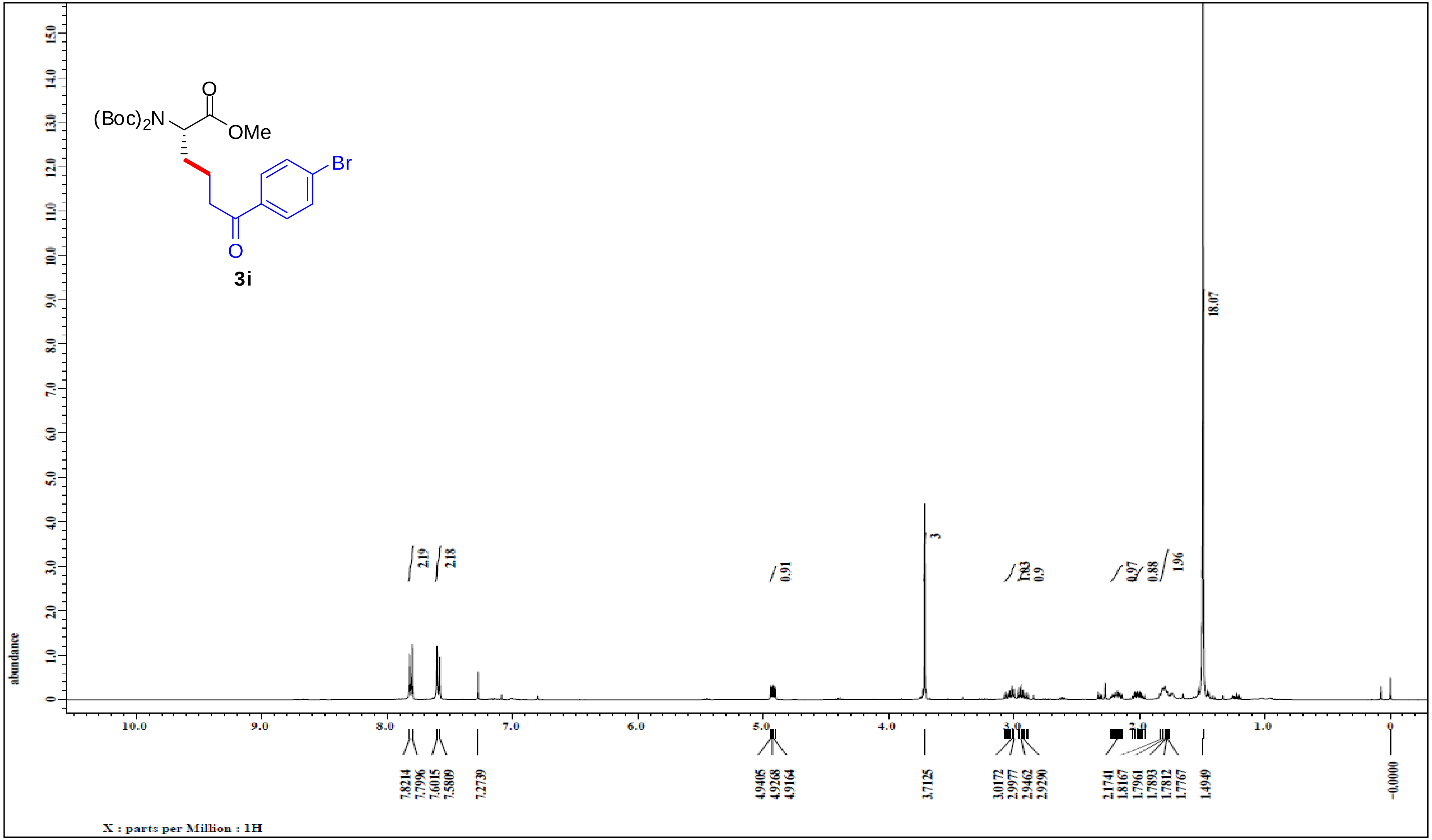

(Boc)2N
O
OMe
Br
O
3i
18.07
3
2.19
2.18
0.91
1.83
0.9
0.97
0.88
1.96
abundance
X : parts per Million : 1H
7.8214
7.7996
7.6015
7.5809
7.2739
4.9405
4.9268
4.9164
3.7125
3.0172
2.9977
2.9462
2.9290
2.1741
1.8167
1.7961
1.7893
1.7812
1.7767
1.4949
-0.0000

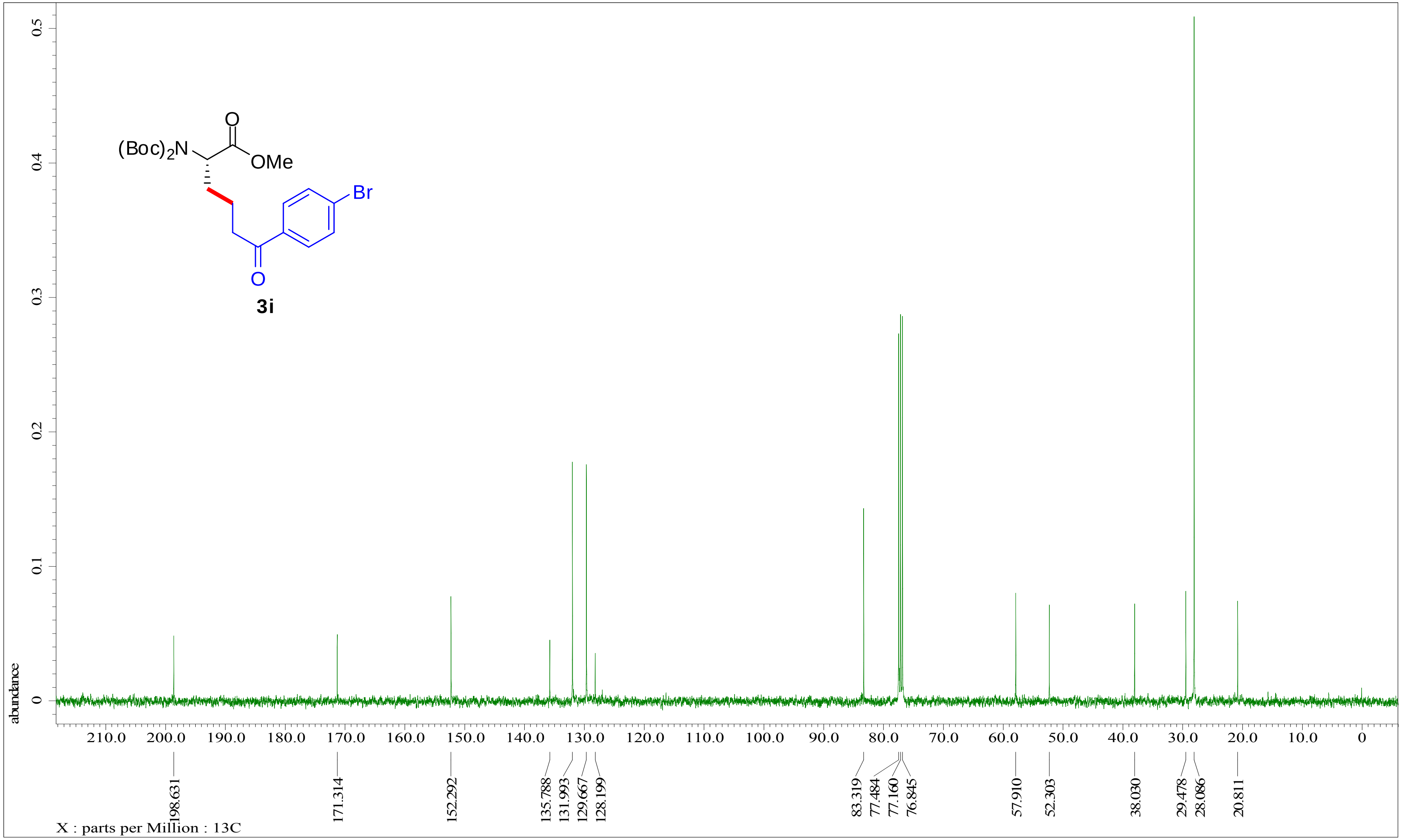
(Boc)2N
O
OMe
Br
O
3i
0.5
0.4
0.3
0.2
0.1
0
abundance
210.0 200.0 190.0 180.0 170.0 160.0 150.0 140.0 130.0 120.0 110.0 100.0 90.0 80.0 70.0 60.0 50.0 40.0 30.0 20.0 10.0 0
198.631
171.314
152.292
135.788
131.993
129.667
128.199
83.319
77.484
77.160
76.845
57.910
52.303
38.030
29.478
28.086
20.811
X : parts per Million : 13C

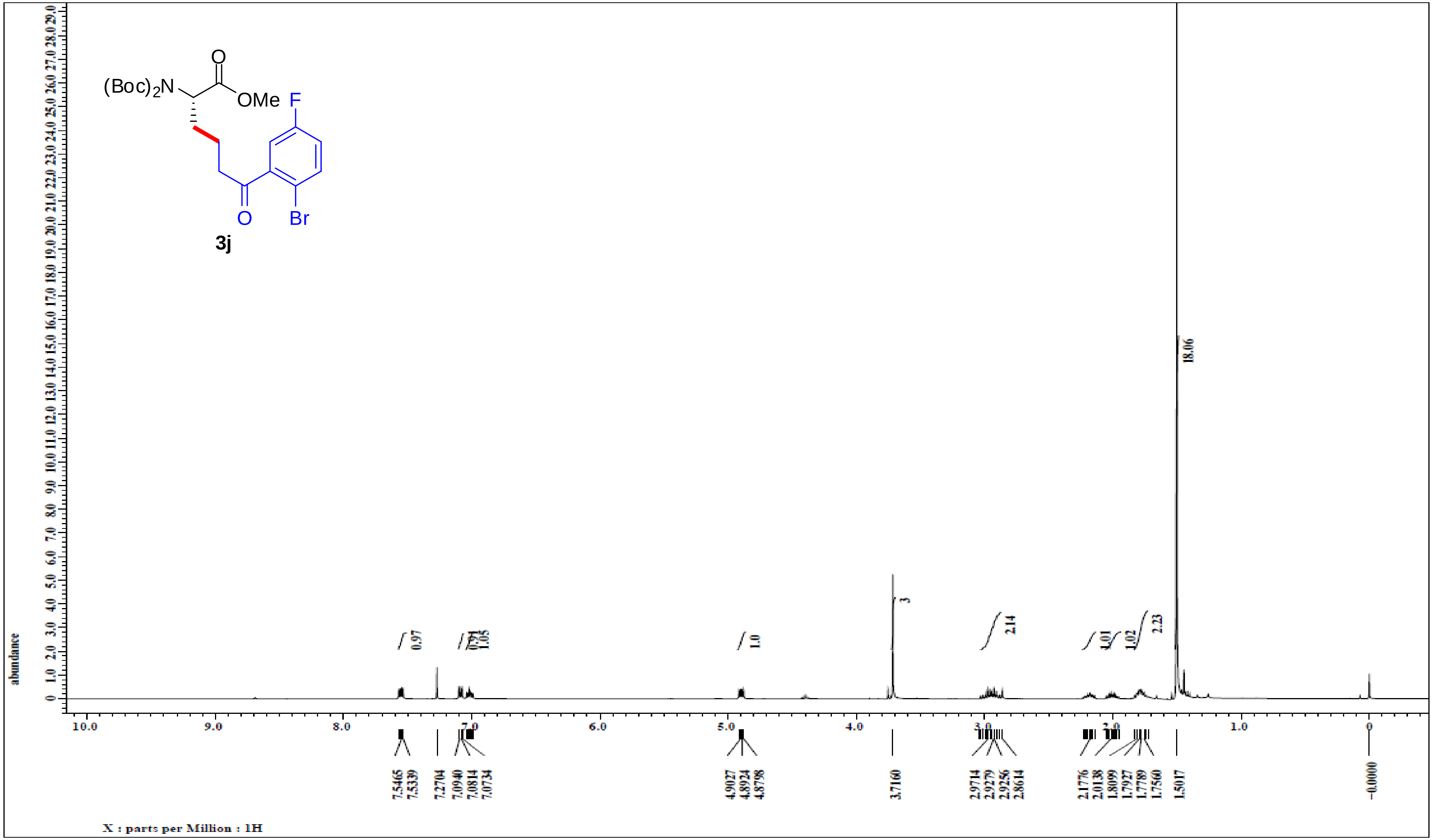
(Boc)2N
O
OMe
F
O
Br
3j
abundance
0.97
1.05
1.0
3
2.14
1.01
1.02
2.23
18.06
10.0
9.0
8.0
7.0
6.0
5.0
4.0
3.0
2.0
1.0
0
7.5465
7.5339
7.2704
7.0940
7.0814
7.0734
4.9027
4.8924
4.8798
3.7160
2.9714
2.9279
2.9256
2.8614
2.1776
2.0138
1.8099
1.7927
1.7789
1.7560
1.5017
-0.0000
X : parts per Million : 1H

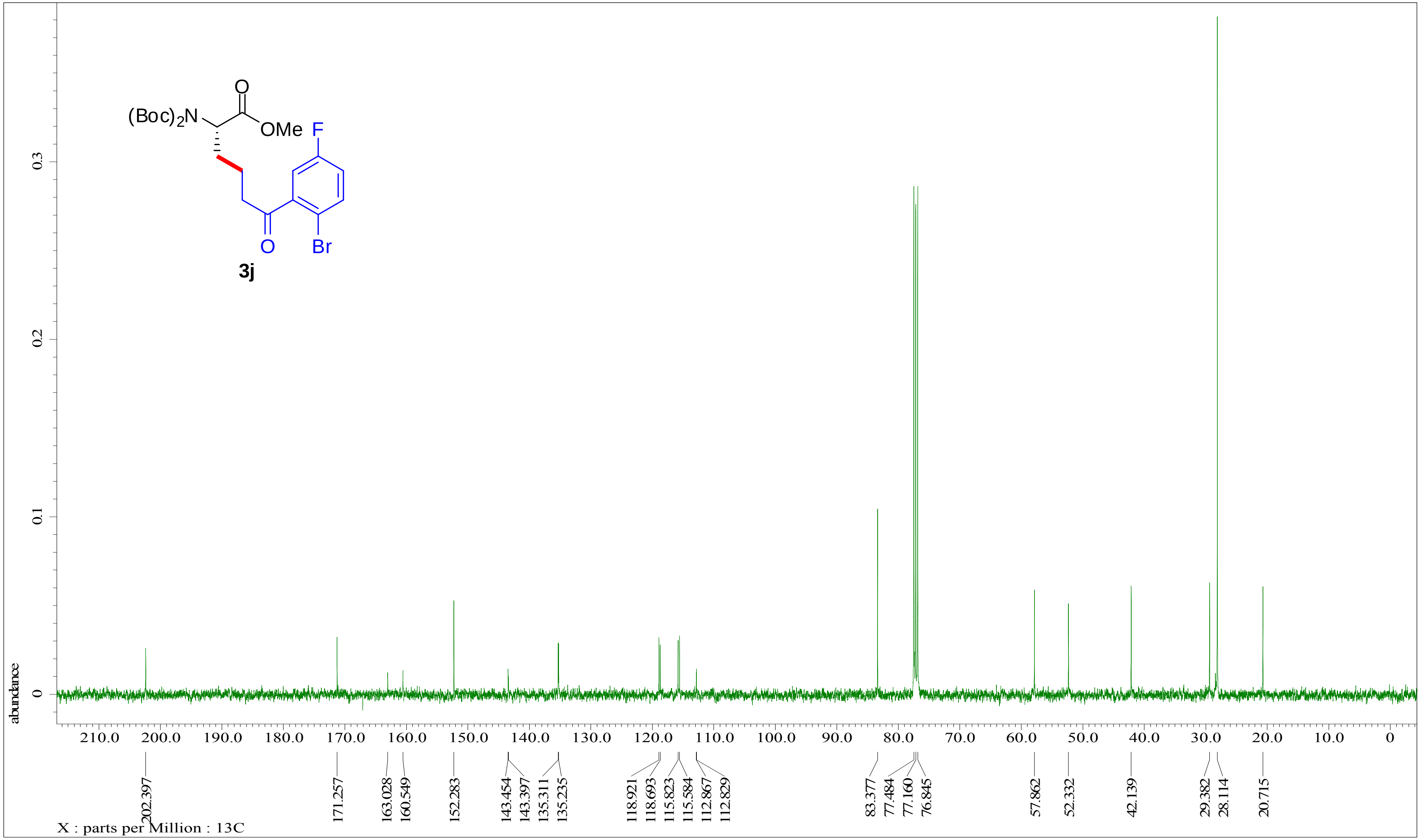
(Boc)2N
OMe
F
O
Br
3j
abundance
0
0.1
0.2
0.3
210.0 200.0 190.0 180.0 170.0 160.0 150.0 140.0 130.0 120.0 110.0 100.0 90.0 80.0 70.0 60.0 50.0 40.0 30.0 20.0 10.0 0
202.397
171.257
163.028
160.549
152.283
143.454
143.397
135.311
135.235
118.921
118.693
115.823
115.584
112.867
112.829
83.377
77.484
77.160
76.845
57.862
52.332
42.139
29.382
28.114
20.715
X : parts per Million : 13C

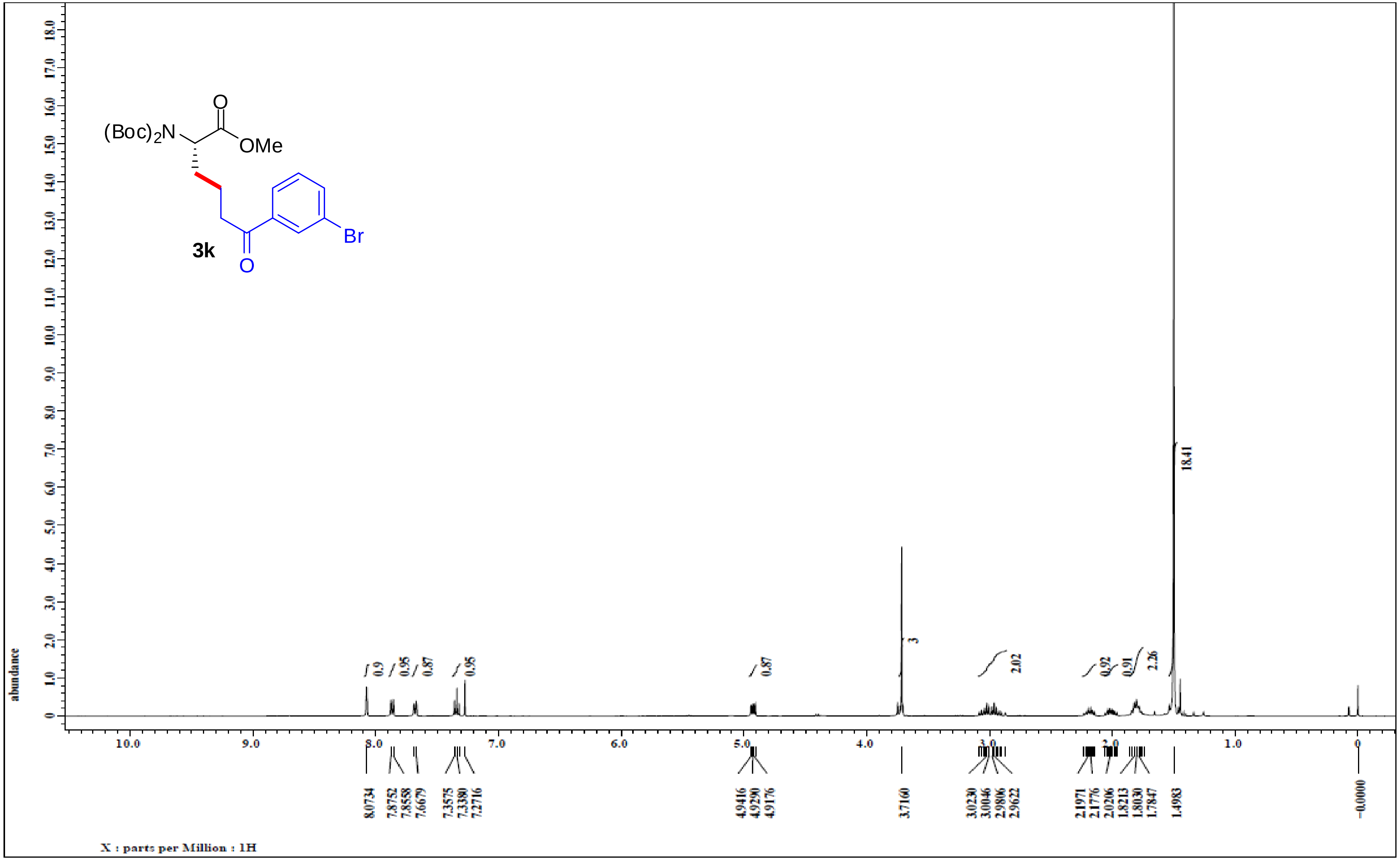
(Boc)2N
O
OMe
Br
O
3k
abundance
X : parts per Million : 1H
8.0734
7.8752
7.8558
7.6679
7.3575
7.3380
7.2716
4.9416
4.9290
4.9176
3.7160
3.0230
3.0046
2.9806
2.9622
2.1971
2.1776
2.0206
1.8213
1.8030
1.7847
1.4983
-0.0000
0.9
0.95
0.87
0.95
0.87
3
2.02
0.92
0.91
2.26
18.41

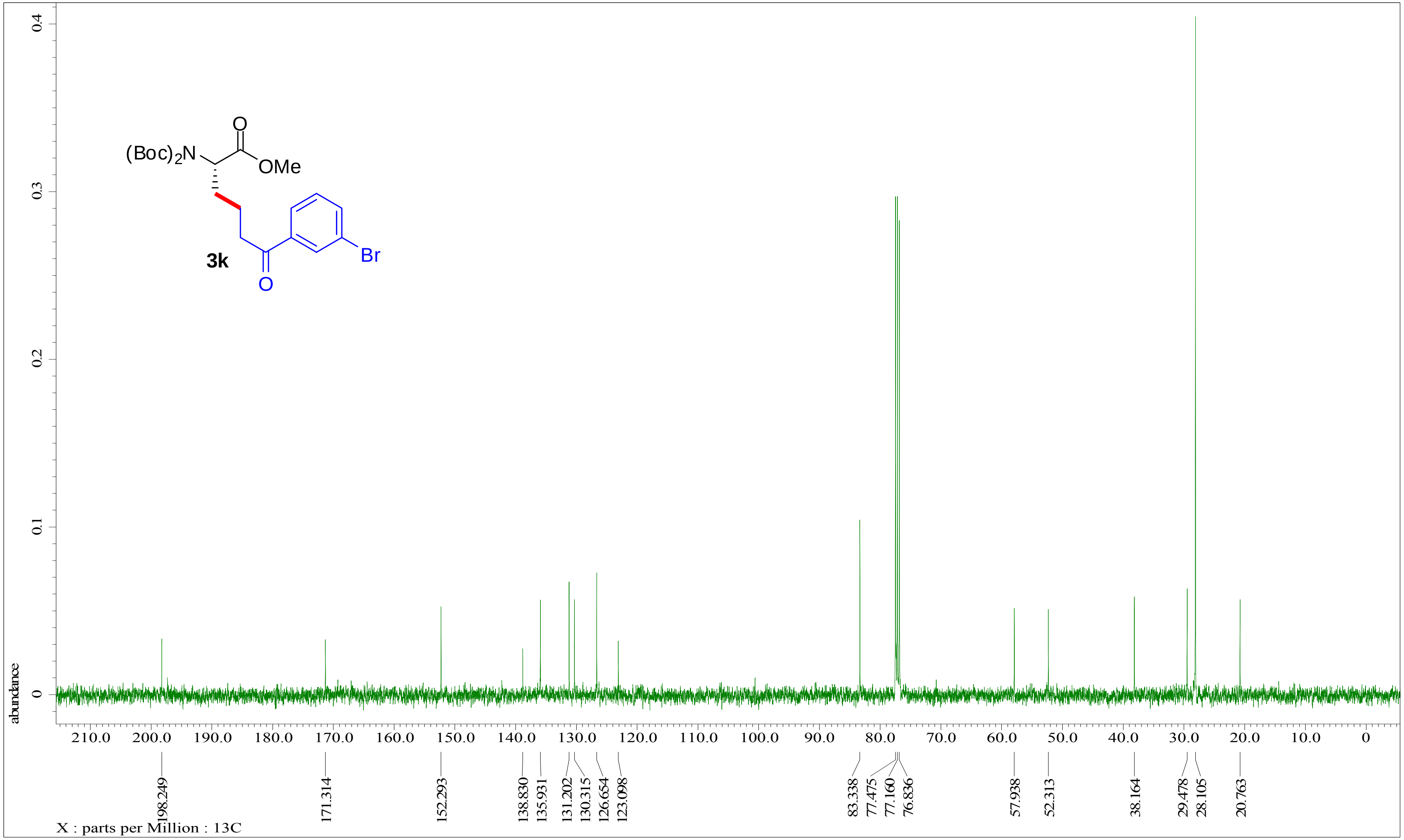
(Boc)2N
O
OMe
Br
O
3k
0.4
0.3
0.2
0.1
0
abundance
210.0 200.0 190.0 180.0 170.0 160.0 150.0 140.0 130.0 120.0 110.0 100.0 90.0 80.0 70.0 60.0 50.0 40.0 30.0 20.0 10.0 0
198.249
171.314
152.293
138.830
135.931
131.202
130.315
126.654
123.098
83.338
77.475
77.160
76.836
57.938
52.313
38.164
29.478
28.105
20.763
X : parts per Million : 13C

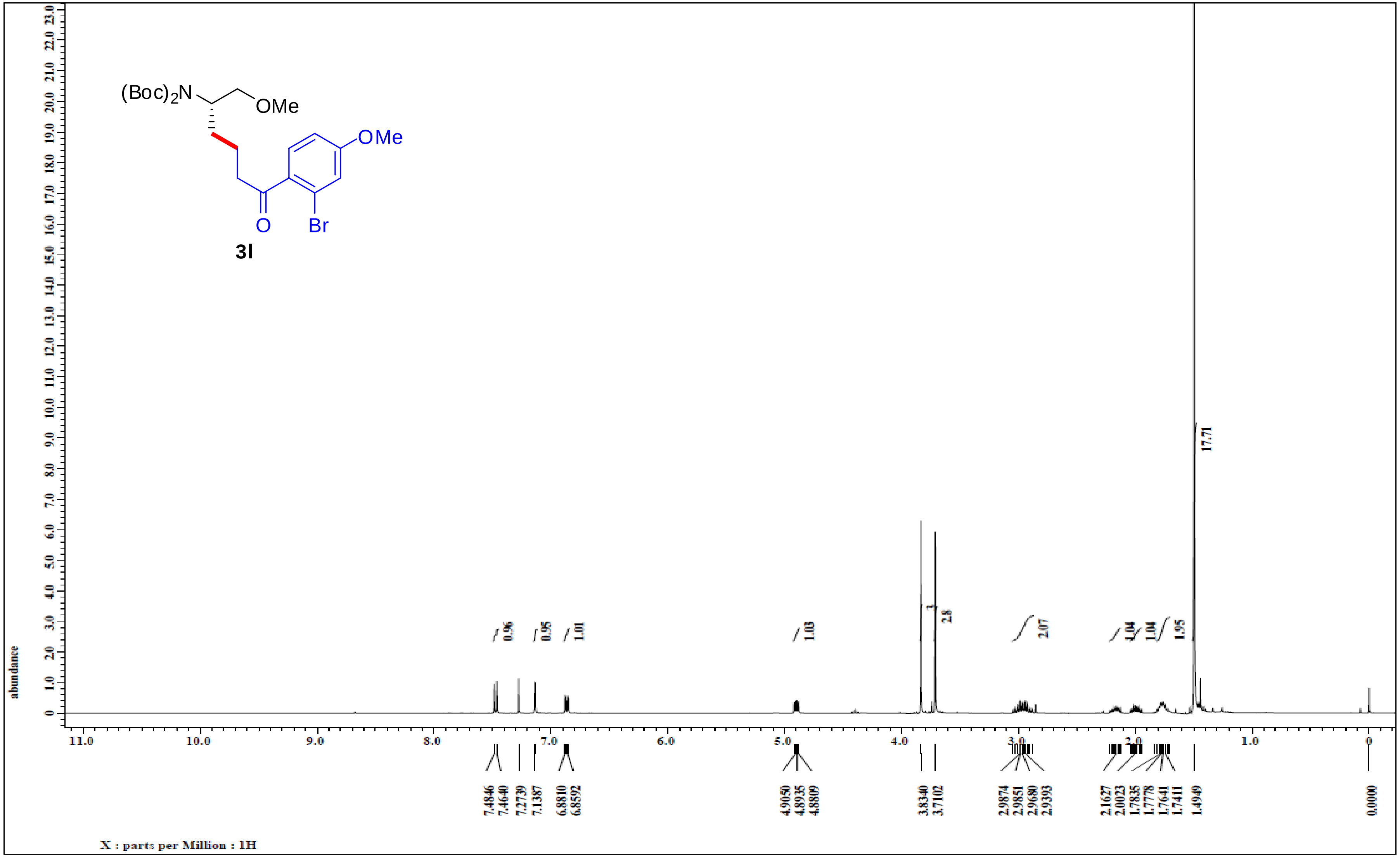
(Boc)2N
OMe
OMe
O
Br
3l
X : parts per Million : 1H
abundance

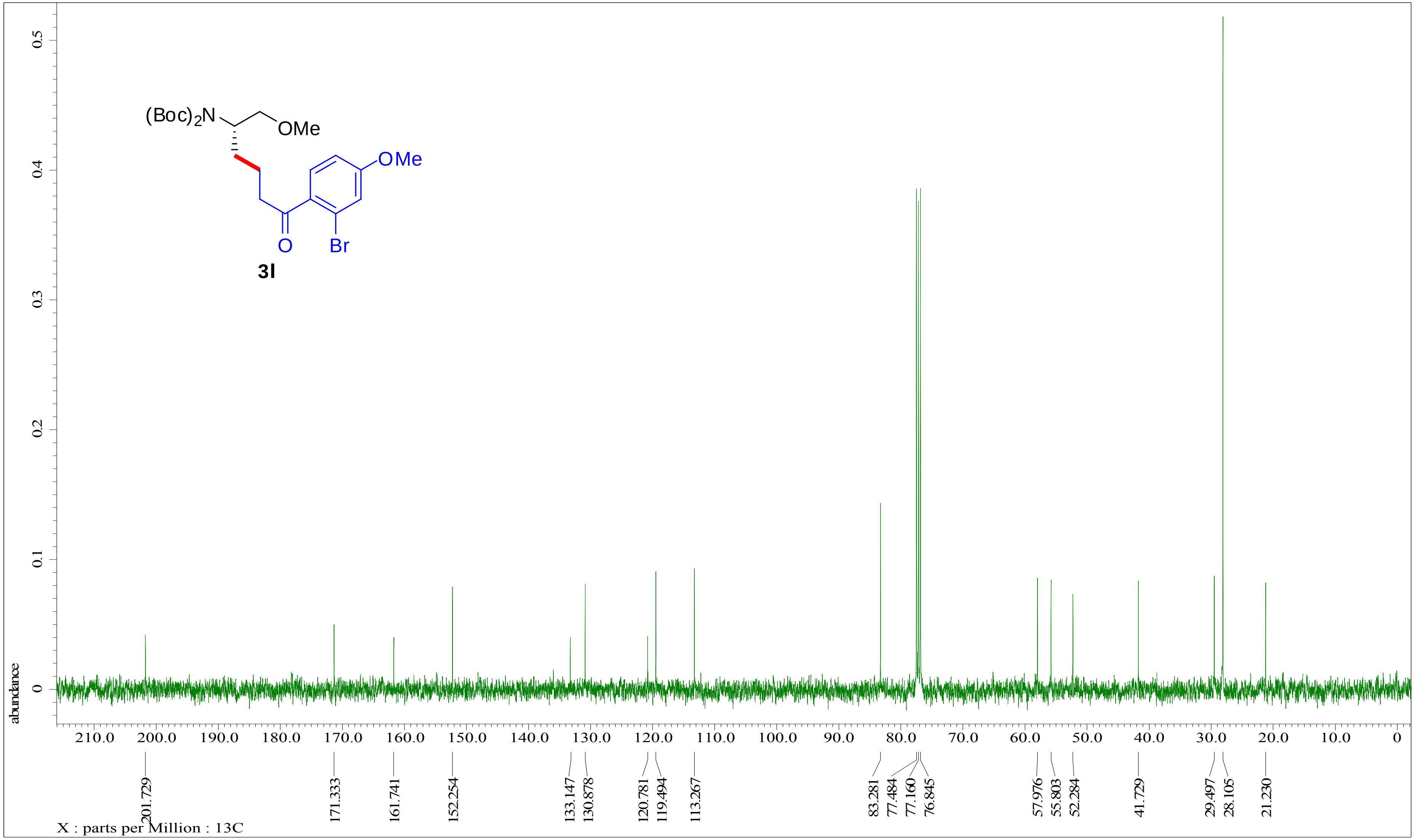
(Boc)2N
OMe
OMe
O
Br
3l
abundance
0
0.1
0.2
0.3
0.4
0.5
210.0 200.0 190.0 180.0 170.0 160.0 150.0 140.0 130.0 120.0 110.0 100.0 90.0 80.0 70.0 60.0 50.0 40.0 30.0 20.0 10.0 0
201.729
171.333
161.741
152.254
133.147
130.878
120.781
119.494
113.267
83.281
77.484
77.160
76.845
57.976
55.803
52.284
41.729
29.497
28.105
21.230
X : parts per Million : 13C

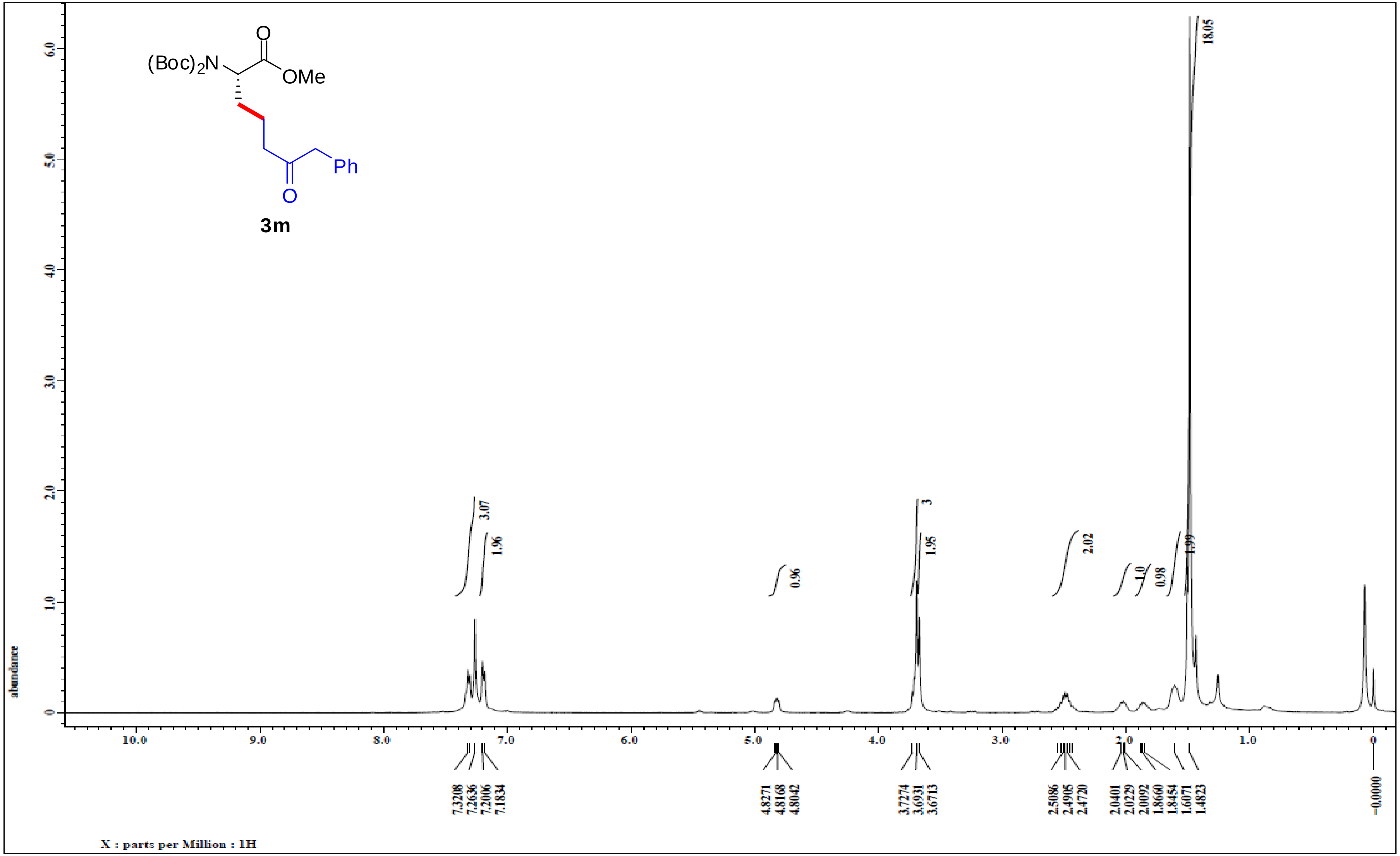
(Boc)2N
O
OMe
Ph
O
3m
X : parts per Million : 1H
abundance
18.05
3.07
1.96
0.96
3
1.95
2.02
1.0
0.98
1.99
7.3208
7.2636
7.2006
7.1834
4.8271
4.8168
4.8042
3.7274
3.6931
3.6713
2.5086
2.4905
2.4720
2.0401
2.0229
2.0092
1.8660
1.8454
1.6071
1.4823
0.0000

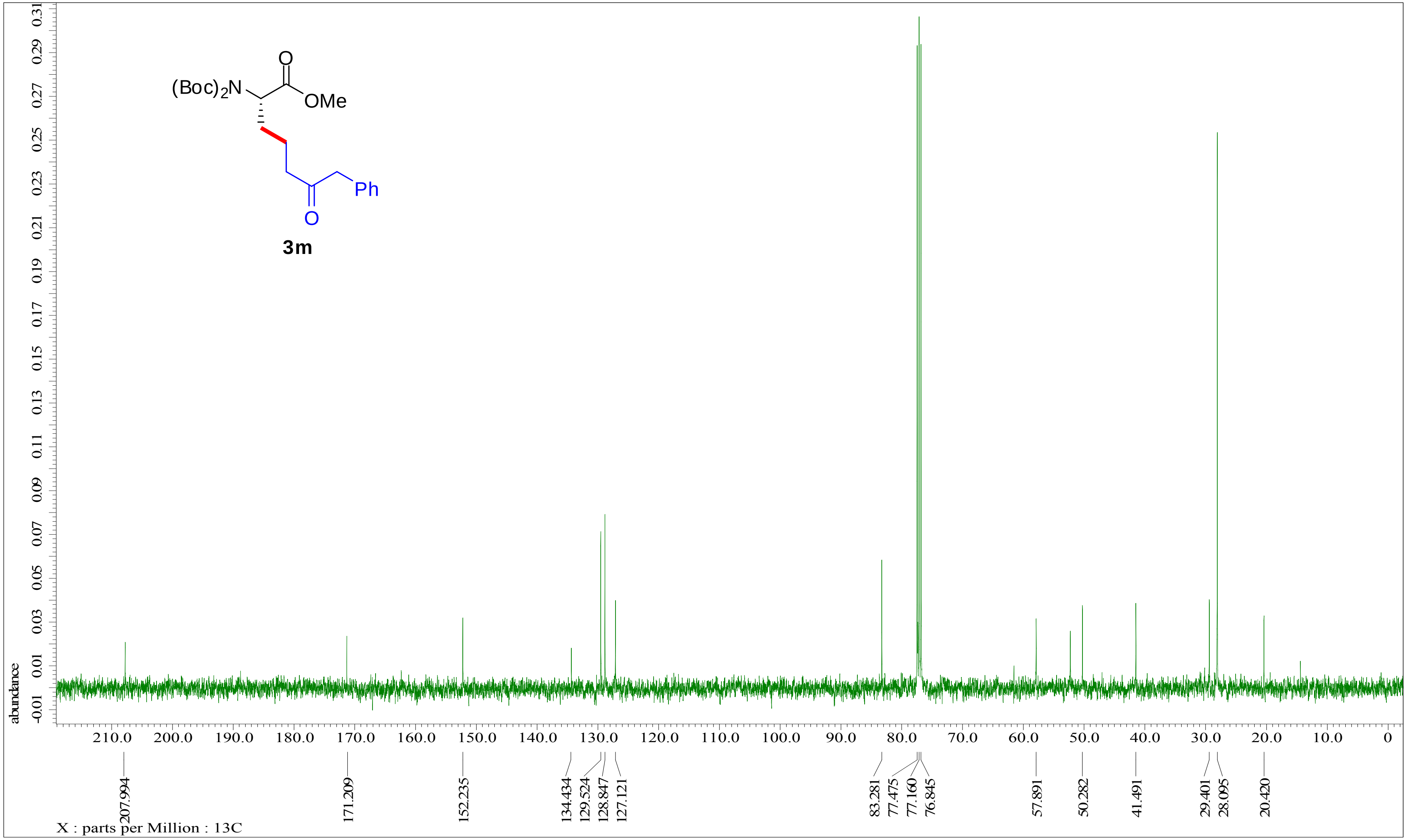
(Boc)2N
O
OMe
Ph
O
3m
abundance
0.31
0.29
0.27
0.25
0.23
0.21
0.19
0.17
0.15
0.13
0.11
0.09
0.07
0.05
0.03
0.01
-0.01
210.0
200.0
190.0
180.0
170.0
160.0
150.0
140.0
130.0
120.0
110.0
100.0
90.0
80.0
70.0
60.0
50.0
40.0
30.0
20.0
10.0
0
207.994
171.209
152.235
134.434
129.524
128.847
127.121
83.281
77.475
77.160
76.845
57.891
50.282
41.491
29.401
28.095
20.420
X : parts per Million : 13C

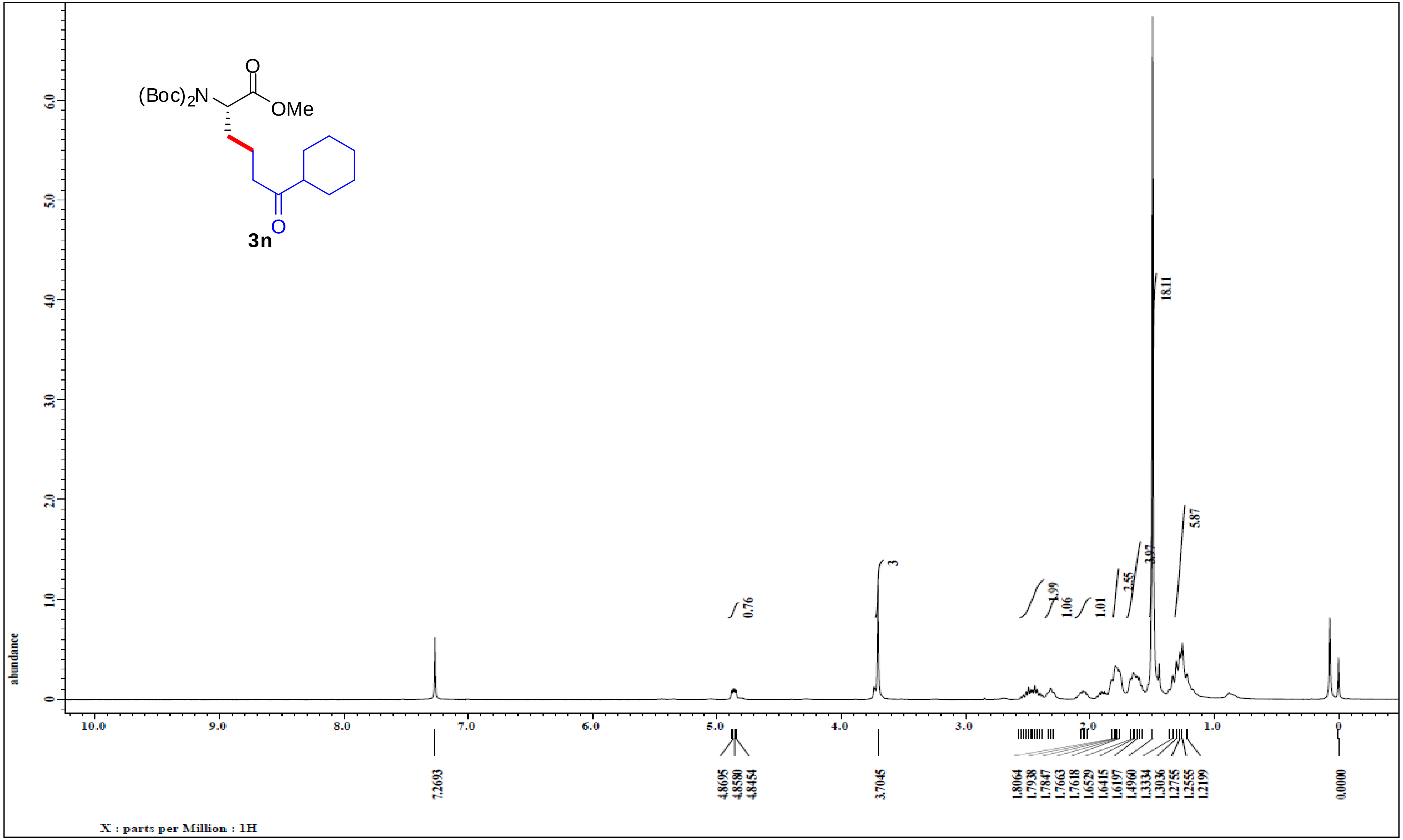
(Boc)2N
O
OMe
O
3n
18.11
5.87
3.97
2.55
1.01
1.06
1.99
3
0.76
7.2693
4.8695
4.8580
4.8454
3.7045
1.8064
1.7938
1.7847
1.7663
1.7618
1.6529
1.6415
1.6197
1.4960
1.3334
1.3036
1.2755
1.2555
1.2199
0.0000
abundance
X : parts per Million : 1H

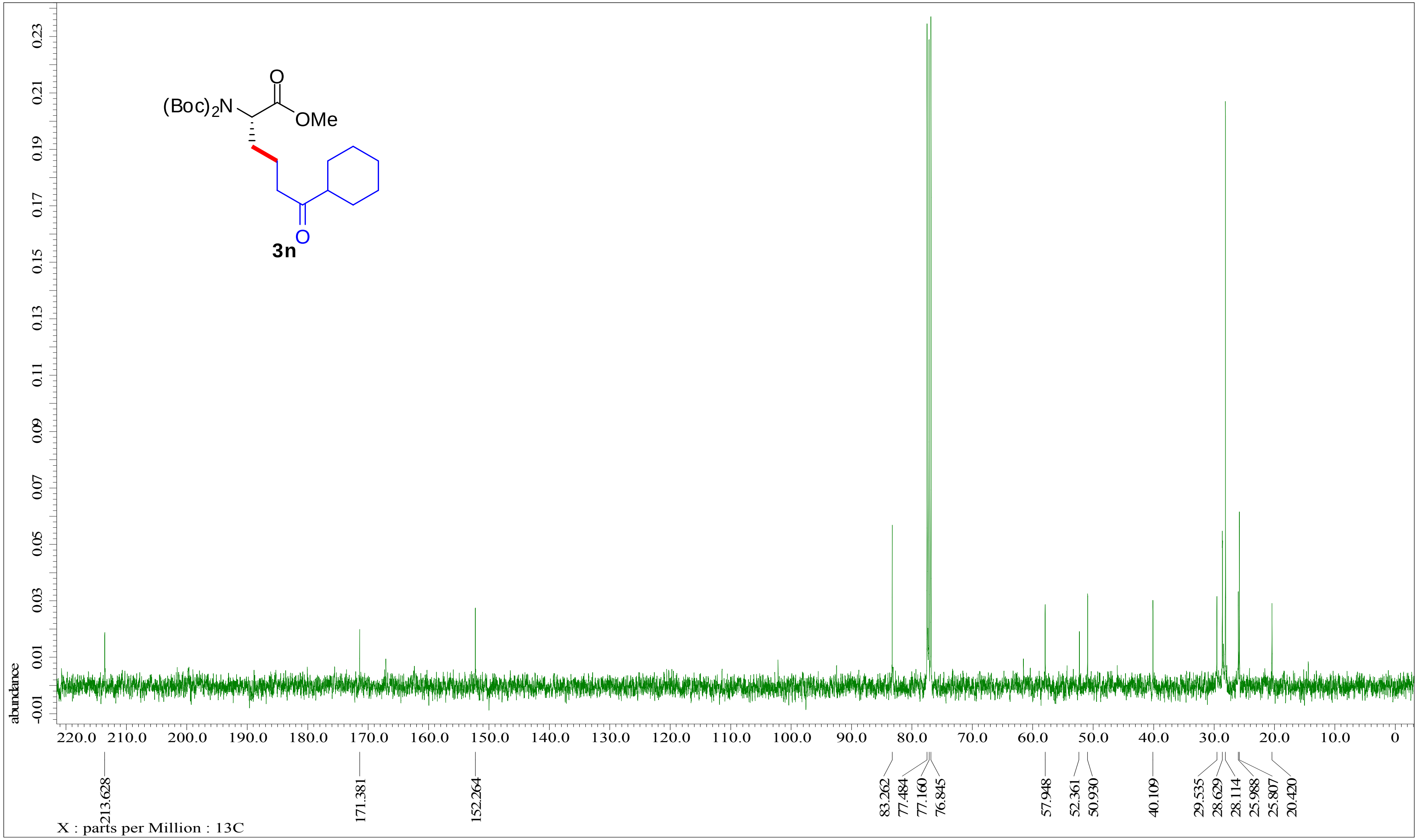

(Boc)2N
O
OMe
O
3n
abundance
0.23
0.21
0.19
0.17
0.15
0.13
0.11
0.09
0.07
0.05
0.03
0.01
-0.01
220.0 210.0 200.0 190.0 180.0 170.0 160.0 150.0 140.0 130.0 120.0 110.0 100.0 90.0 80.0 70.0 60.0 50.0 40.0 30.0 20.0 10.0 0
213.628
171.381
152.264
83.262
77.484
77.160
76.845
57.948
52.361
50.930
40.109
29.535
28.629
28.114
25.988
25.807
20.420
X : parts per Million : 13C

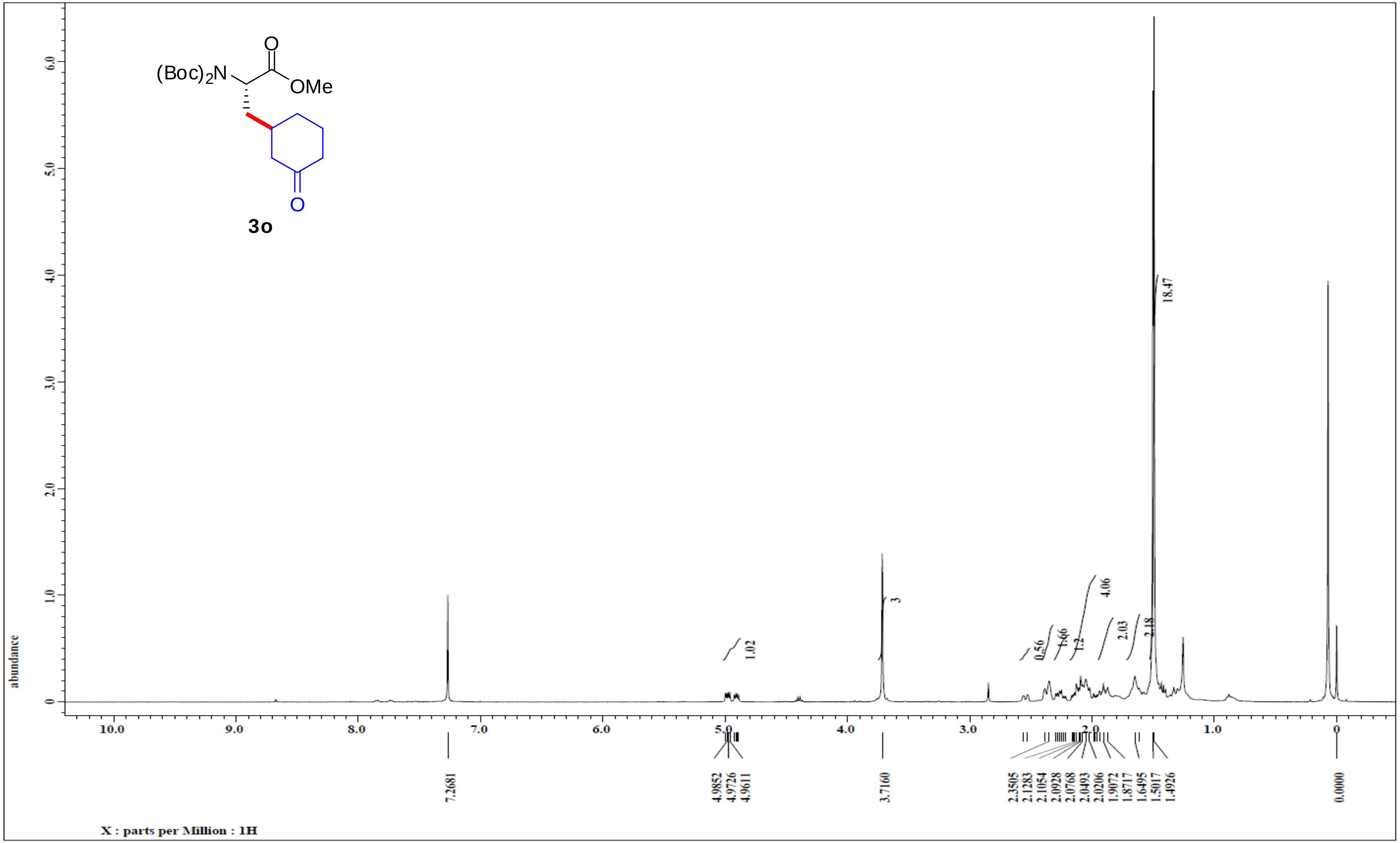
(Boc)2N
O
OMe
O
3o
abundance
X : parts per Million : 1H
7.2681
4.9852
4.9726
4.9611
3.7160
2.3505
2.1283
2.1054
2.0928
2.0768
2.0493
2.0206
1.9072
1.8717
1.6495
1.5017
1.4926
0.0000
1.02
3
0.56
1.66
1.2
4.06
2.03
2.18
18.47

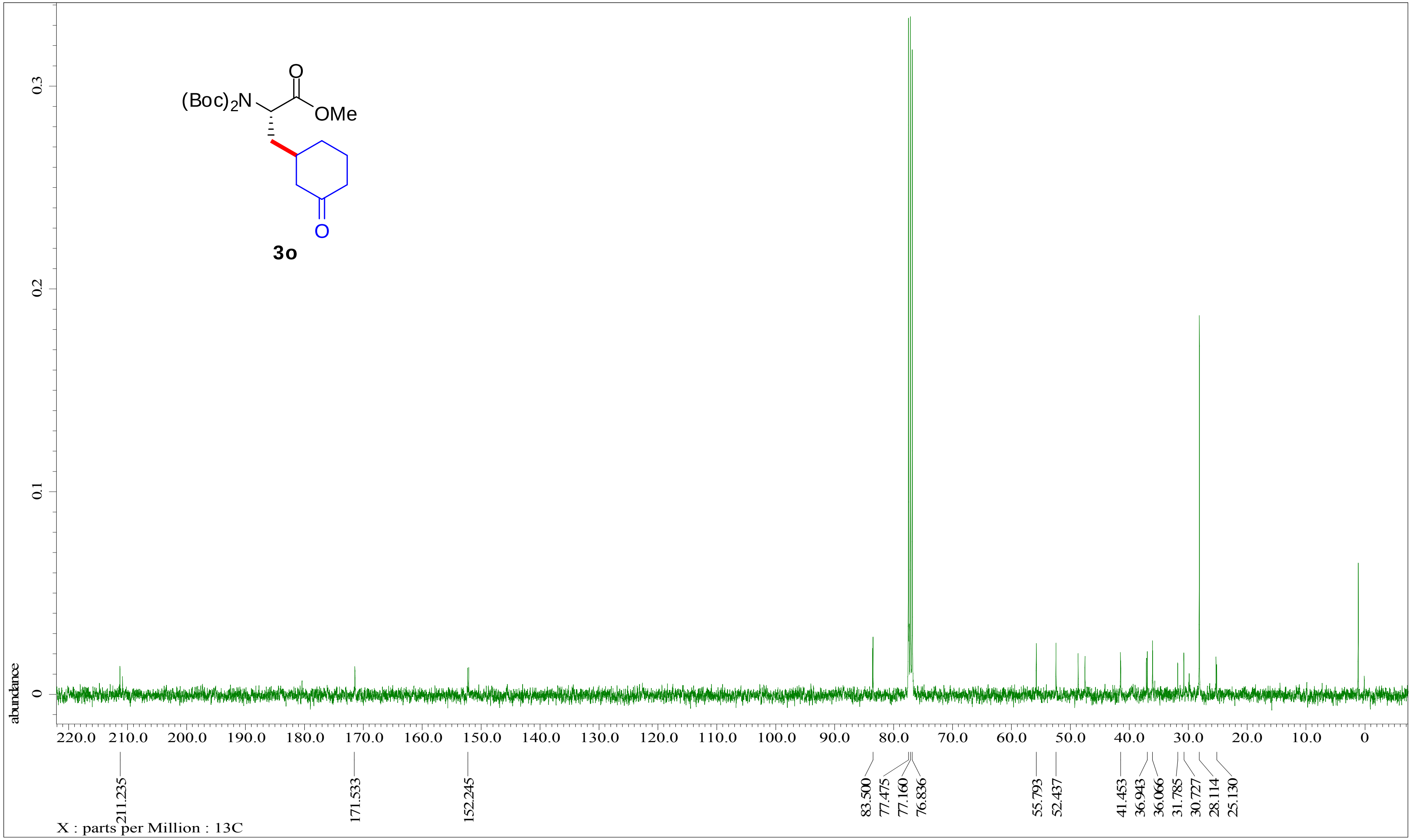
(Boc)2N
O
OMe
O
3o
abundance
0
0.1
0.2
0.3
220.0 210.0 200.0 190.0 180.0 170.0 160.0 150.0 140.0 130.0 120.0 110.0 100.0 90.0 80.0 70.0 60.0 50.0 40.0 30.0 20.0 10.0 0
211.235
171.533
152.245
83.500
77.475
77.160
76.836
55.793
52.437
41.453
36.943
36.066
31.785
30.727
28.114
25.130
X : parts per Million : 13C

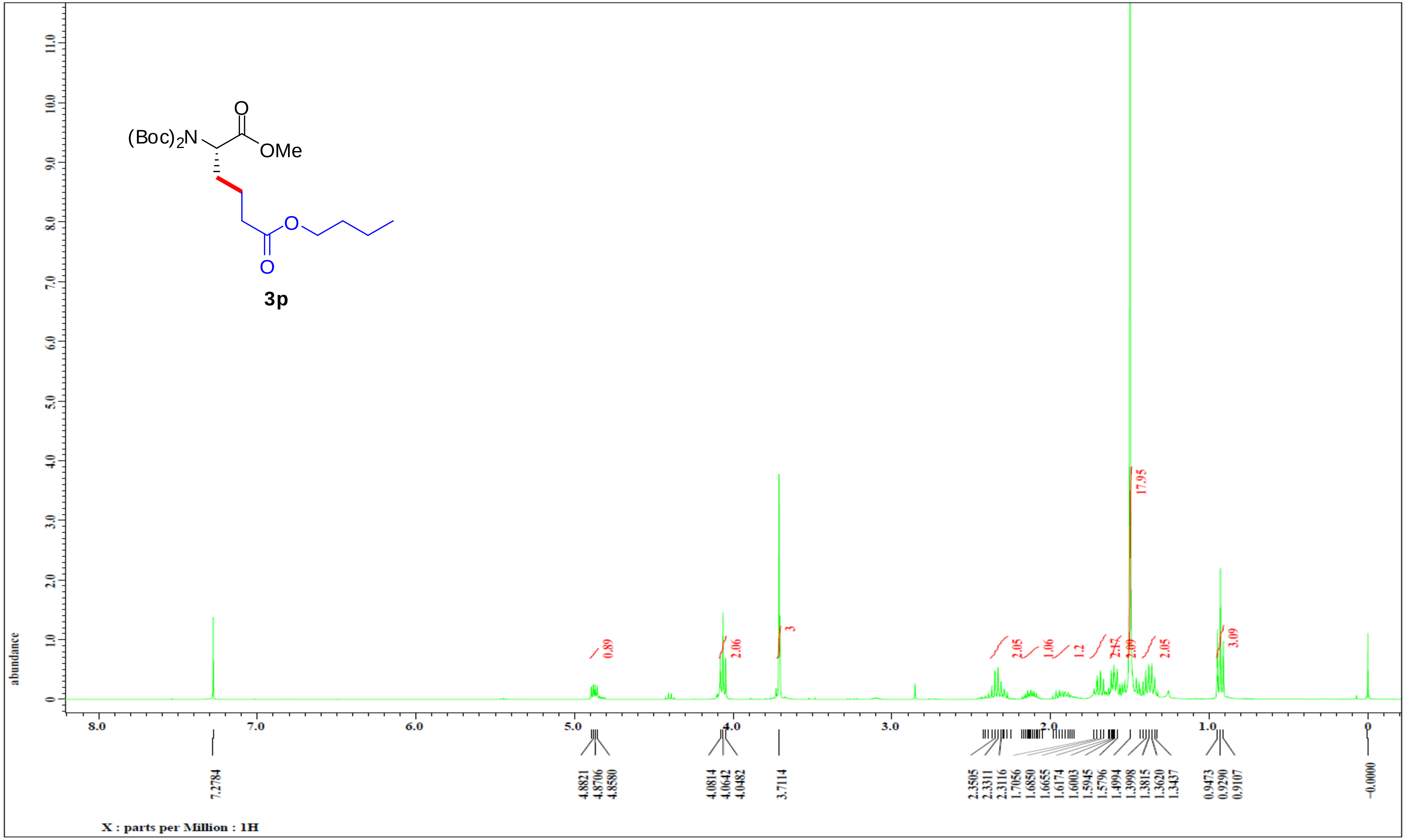

(Boc)2N
O
OMe
O
O
3p
abundance
X : parts per Million : 1H
0.89
2.06
3
2.05
1.06
1.2
17.95
2.05
3.09
7.2784
4.8821
4.8706
4.8580
4.0814
4.0642
4.0482
3.7114
2.3505
2.3311
2.3116
1.7056
1.6850
1.6655
1.6174
1.6003
1.5945
1.5796
1.4994
1.3998
1.3815
1.3620
1.3437
0.9473
0.9290
0.9107
-0.0000

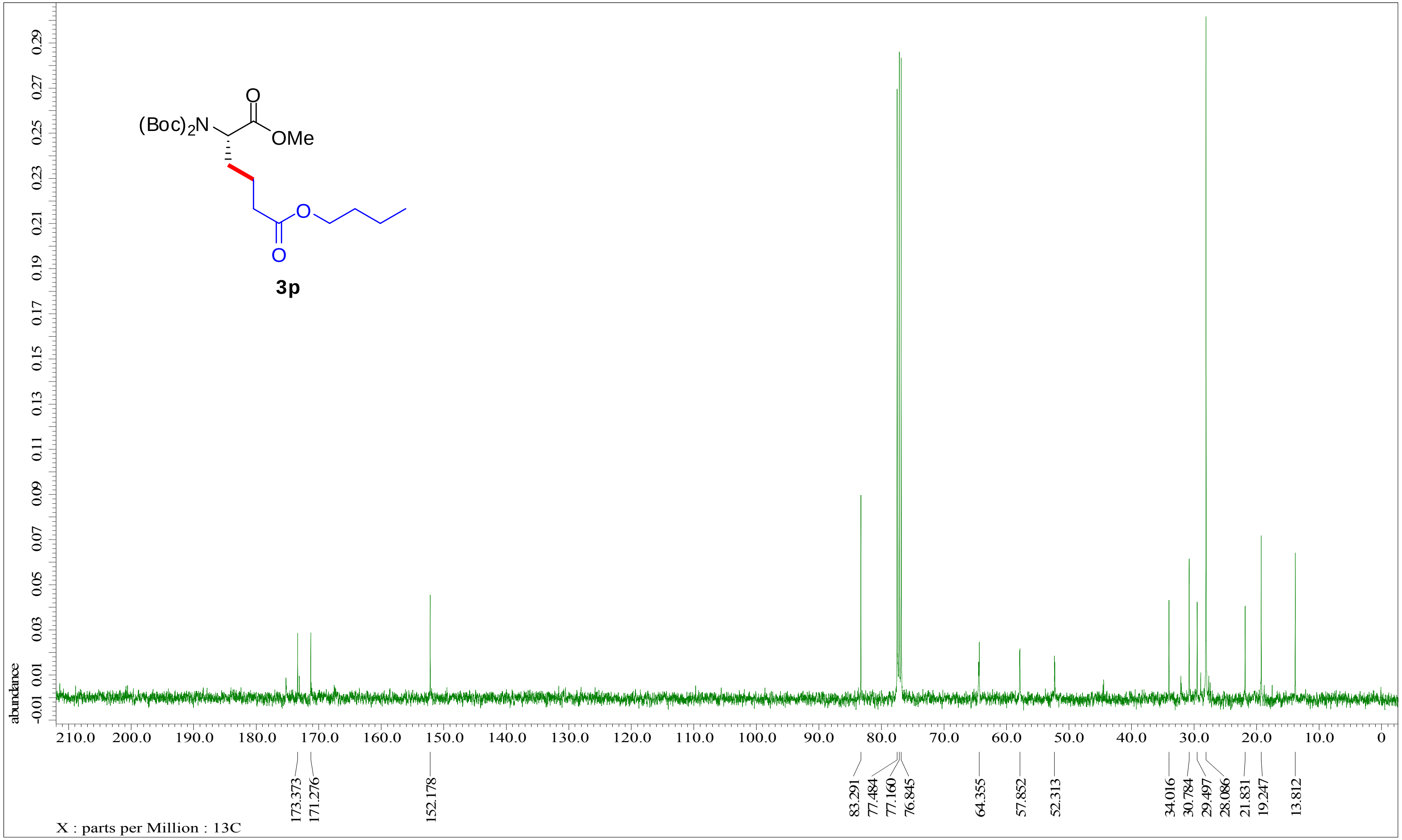

(Boc)2N
OMe
O
O
O
3p
abundance
X : parts per Million : 13C
173.373
171.276
152.178
83.291
77.484
77.160
76.845
64.355
57.852
52.313
34.016
30.784
29.497
28.086
21.831
19.247
13.812

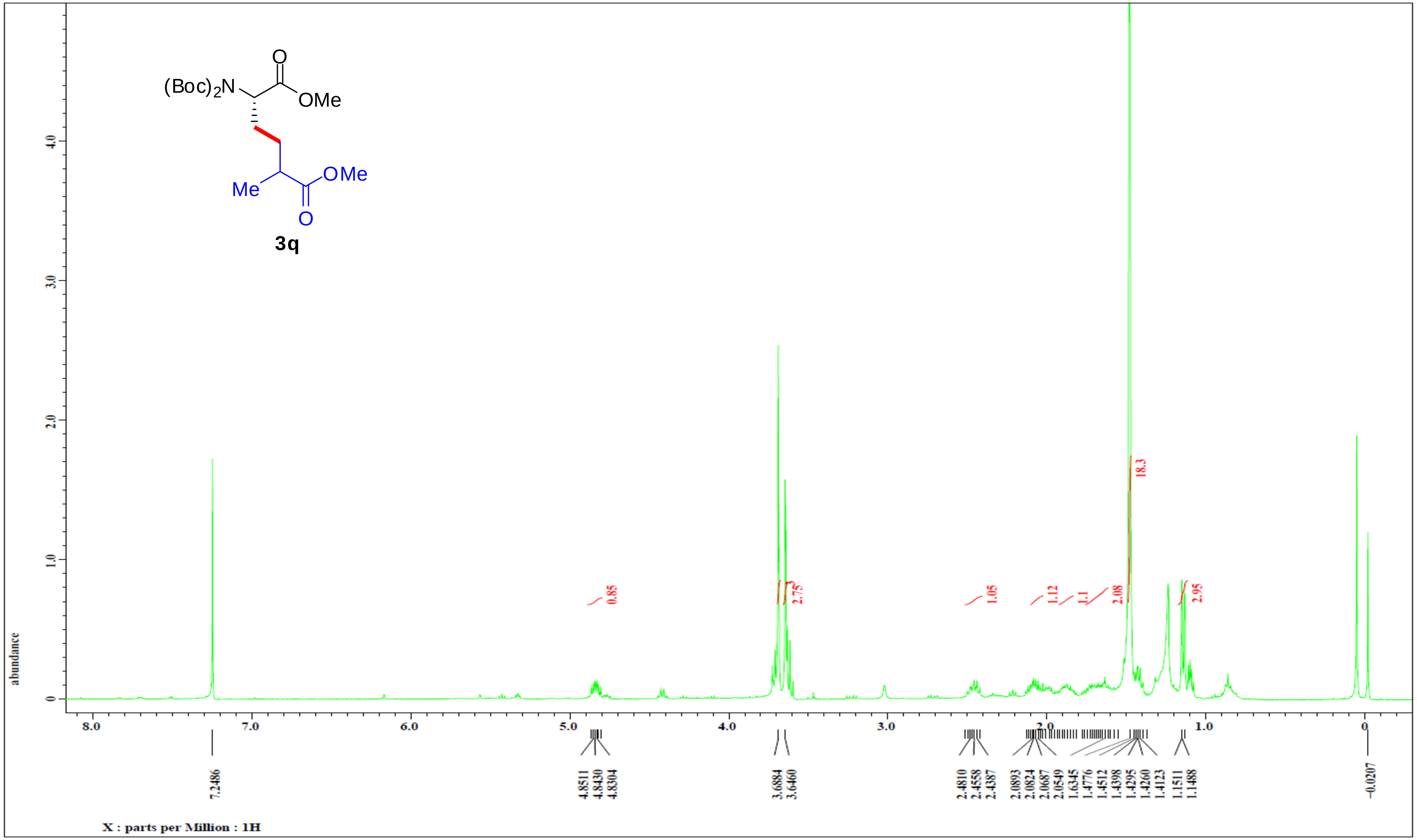

(Boc)2N
O
OMe
Me
OMe
O
3q
abundance
X : parts per Million : 1H
8.0
7.0
6.0
5.0
4.0
3.0
2.0
1.0
0
7.2486
4.8511
4.8430
4.8304
3.6884
3.6460
2.4810
2.4558
2.4387
2.0893
2.0824
2.0687
2.0549
1.6345
1.4776
1.4512
1.4398
1.4295
1.4260
1.4123
1.1511
1.1488
-0.0207
0.85
2.75
1.05
1.12
1.1
2.08
18.3
2.95

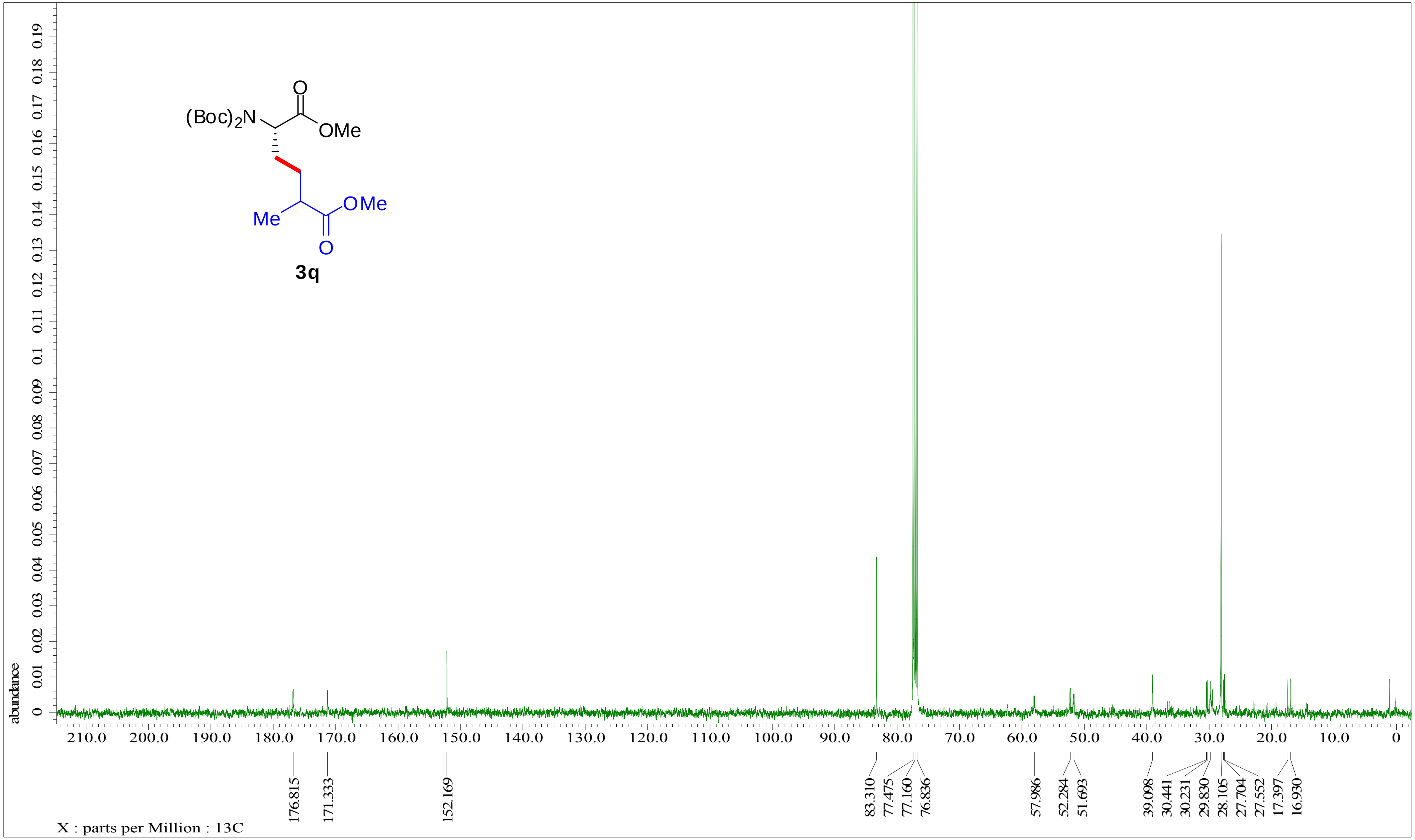
(Boc)2N
OMe
Me
OMe
3q
abundance
X : parts per Million : 13C
176.815
171.333
152.169
83.310
77.475
77.160
76.836
57.986
52.284
51.693
39.098
30.441
30.231
29.830
28.105
27.704
27.552
17.397
16.930

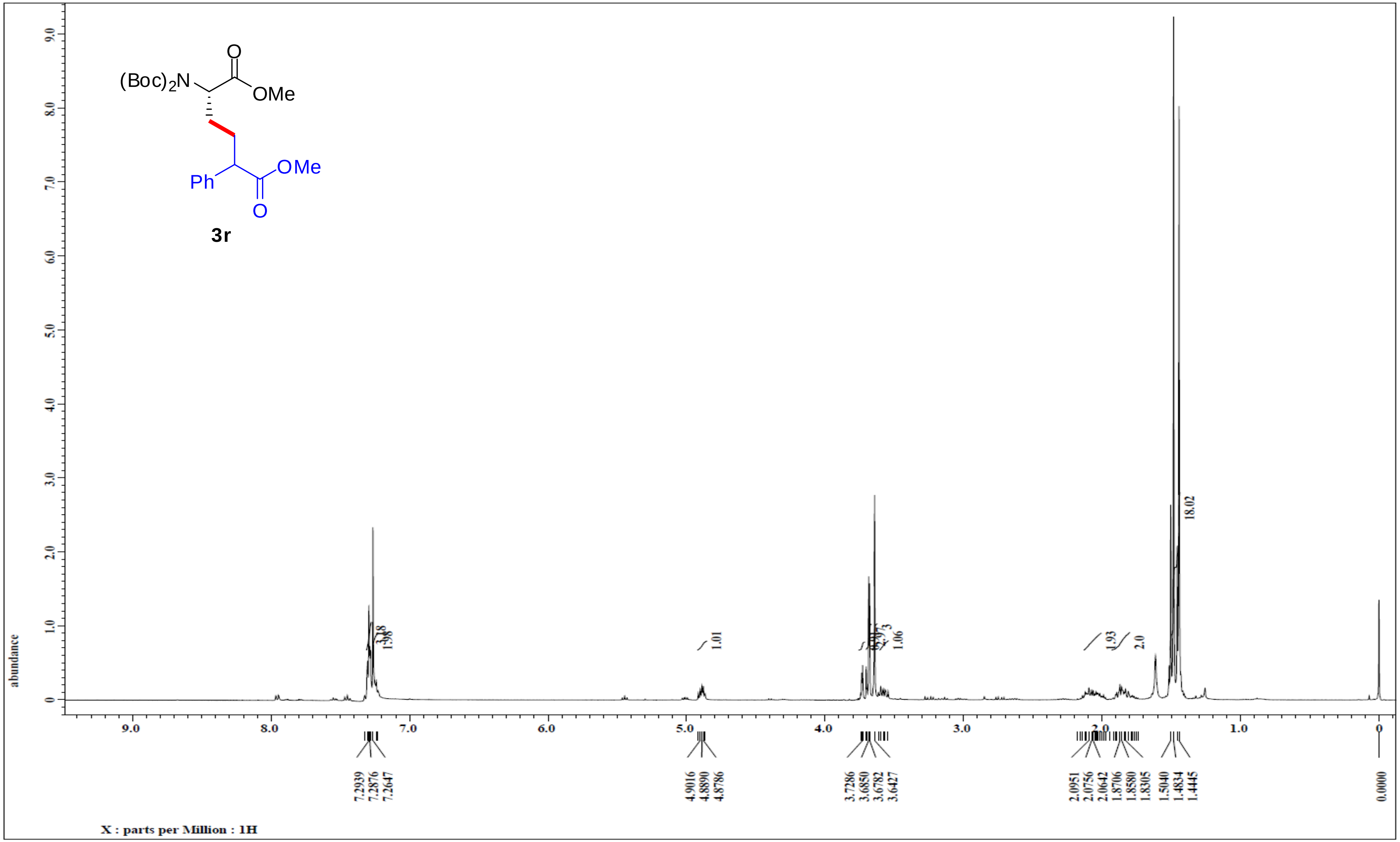
(Boc)2N
OMe
Ph
OMe
3r
abundance
X : parts per Million : 1H
18.02
7.2939
7.2876
7.2647
4.9016
4.8890
4.8786
3.7286
3.6850
3.6782
3.6427
2.0951
2.0756
2.0642
1.8706
1.8580
1.8305
1.5040
1.4834
1.4445
0.0000

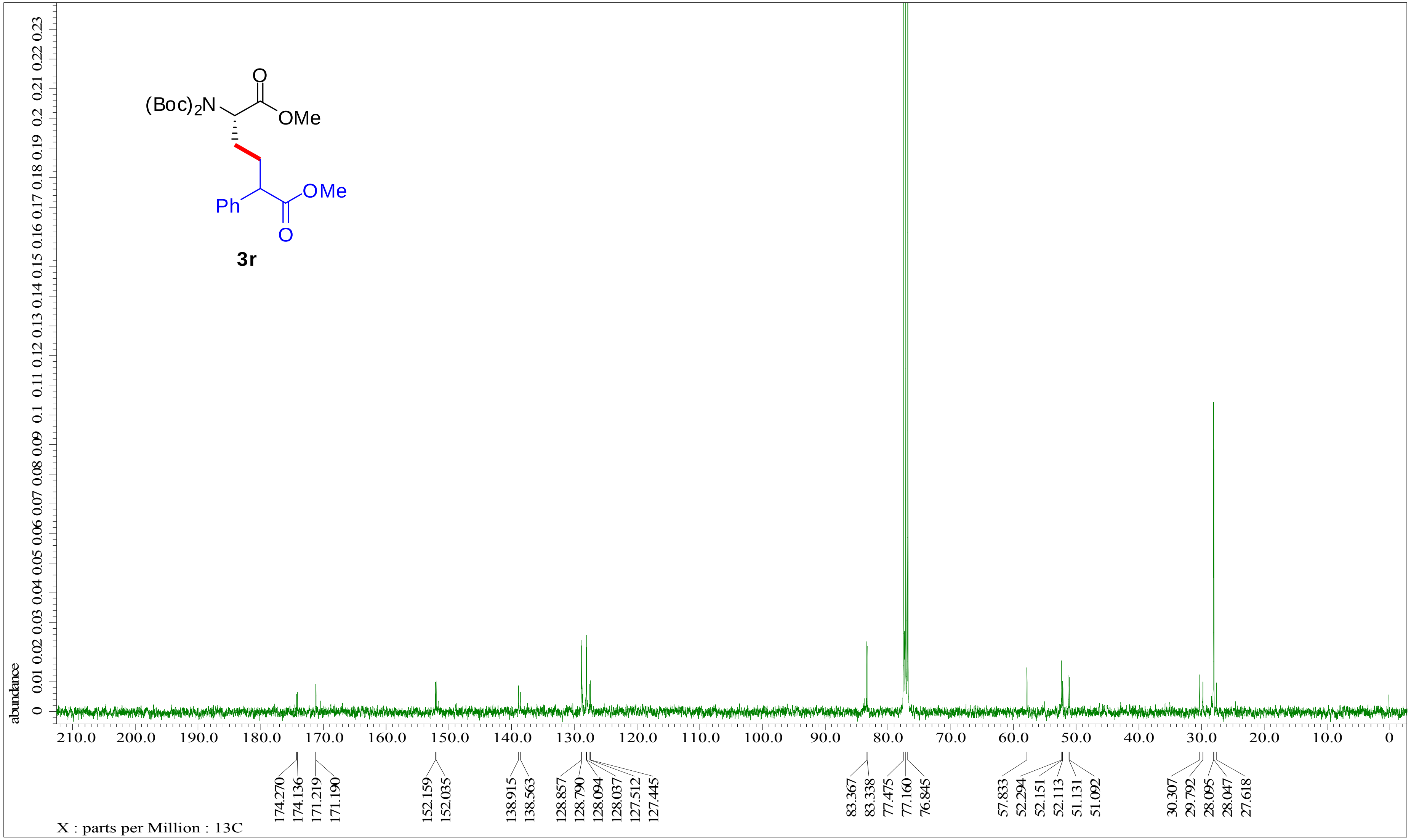
(Boc)2N
O
OMe
Ph
OMe
O
3r
abundance
0 0.01 0.02 0.03 0.04 0.05 0.06 0.07 0.08 0.09 0.1 0.11 0.12 0.13 0.14 0.15 0.16 0.17 0.18 0.19 0.2 0.21 0.22 0.23
210.0 200.0 190.0 180.0 170.0 160.0 150.0 140.0 130.0 120.0 110.0 100.0 90.0 80.0 70.0 60.0 50.0 40.0 30.0 20.0 10.0 0
174.270 174.136 171.219 171.190
152.159 152.035
138.915 138.563
128.857 128.790 128.094 128.037 127.512 127.445
83.367 83.338 77.475 77.160 76.845
57.833 52.294 52.151 52.113 51.131 51.092
30.307 29.792 28.095 28.047 27.618
X : parts per Million : 13C

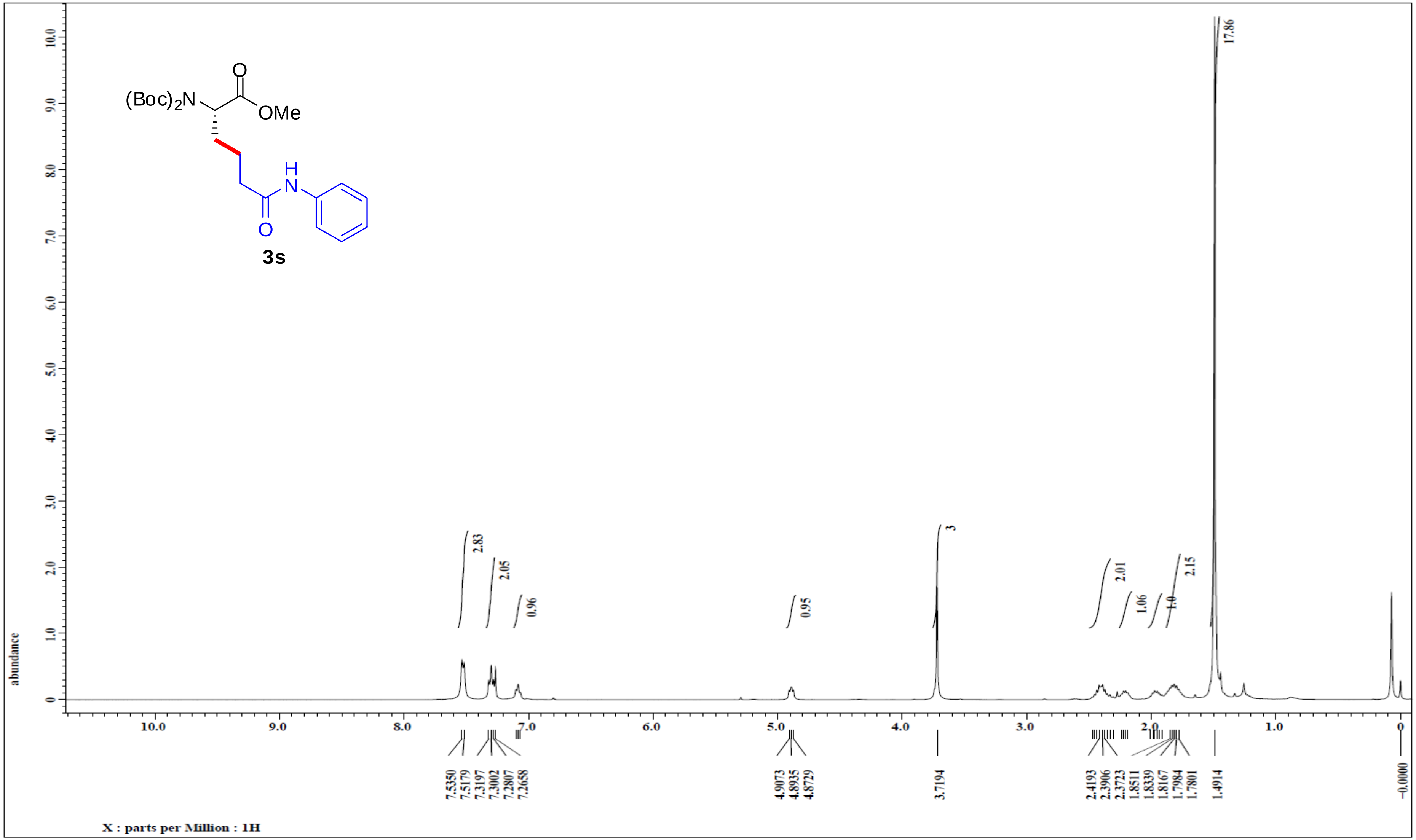
(Boc)2N
OMe
O
H
N
O
3s
abundance
X : parts per Million : 1H
17.86
2.83
2.05
0.96
0.95
3
2.01
1.06
1.0
2.15
7.5350
7.5179
7.3197
7.3002
7.2807
7.2658
4.9073
4.8935
4.8729
3.7194
2.4193
2.3906
2.3723
1.8511
1.8339
1.8167
1.7984
1.7801
1.4914
-0.0000

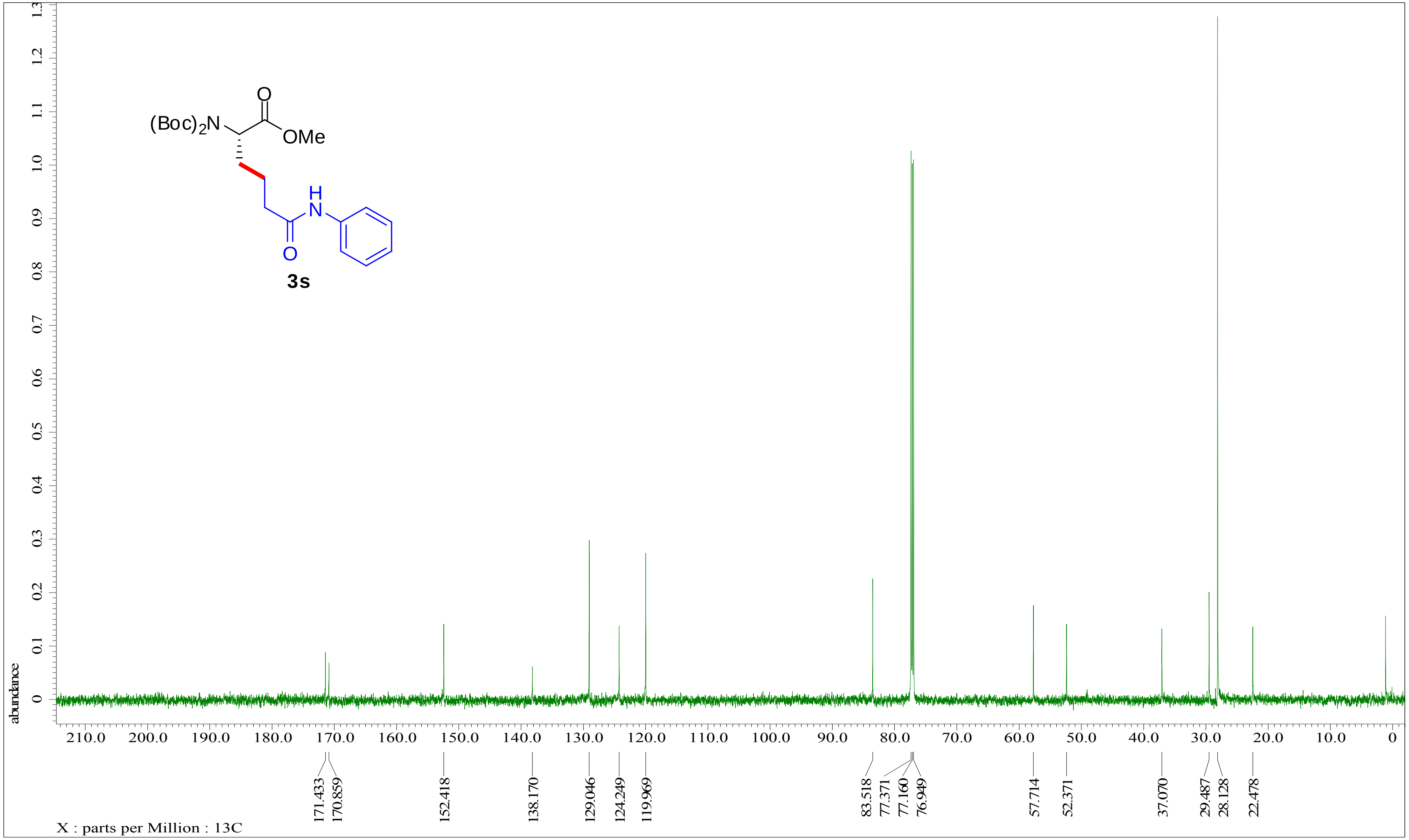
(Boc)2N
OMe
O
H
N
O
3s
abundance
X : parts per Million : 13C
171.433
170.859
152.418
138.170
129.046
124.249
119.969
83.518
77.371
77.160
76.949
57.714
52.371
37.070
29.487
28.128
22.478

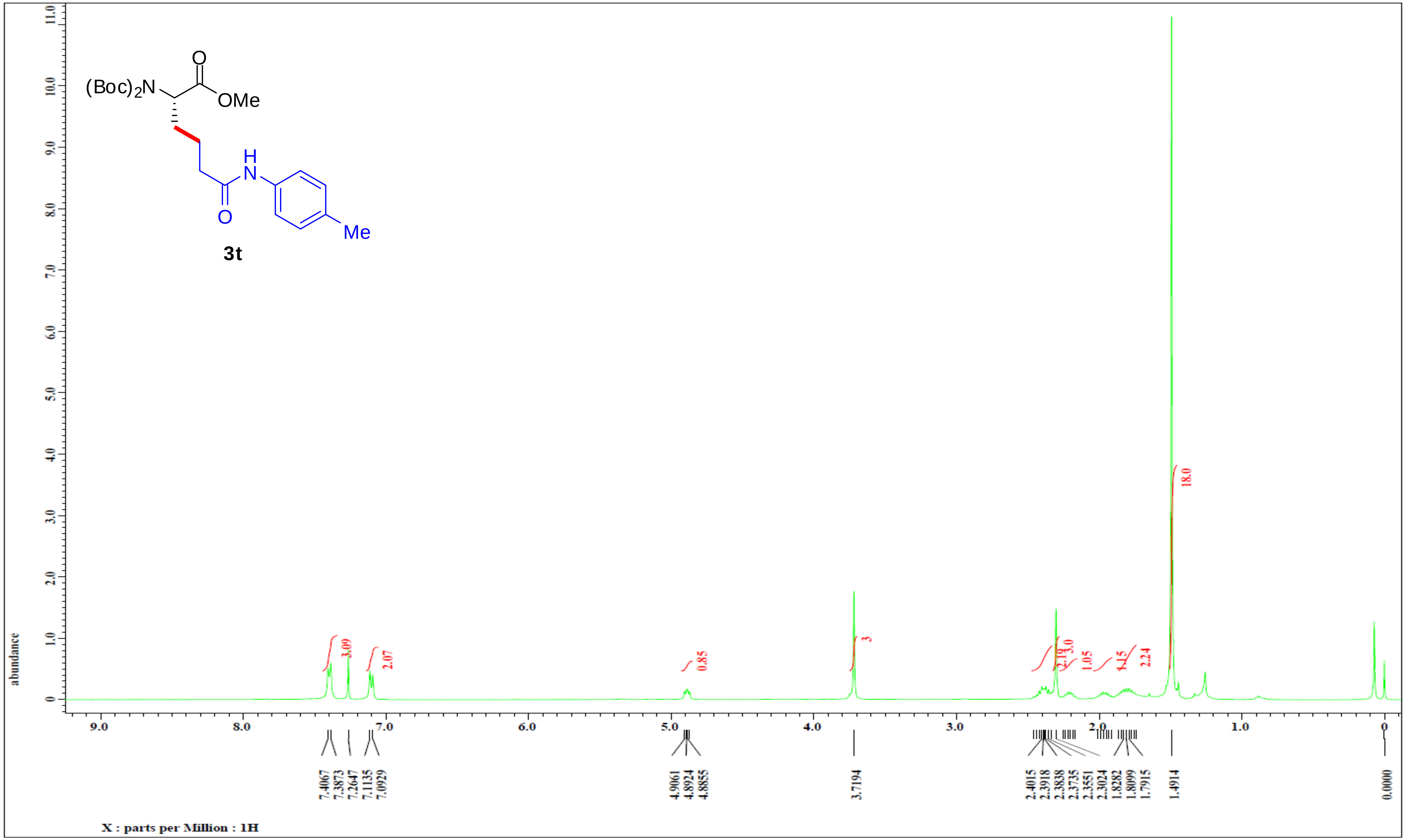
(Boc)2N
OMe
O
H
N
Me
3t
abundance
X : parts per Million : 1H
3.09
2.07
0.85
3
2.19
3.0
1.05
1.15
2.24
18.0
7.4067
7.3873
7.2647
7.1135
7.0929
4.9061
4.8924
4.8855
3.7194
2.4015
2.3918
2.3838
2.3735
2.3551
2.3024
1.8282
1.8099
1.7915
1.4914
0.0000

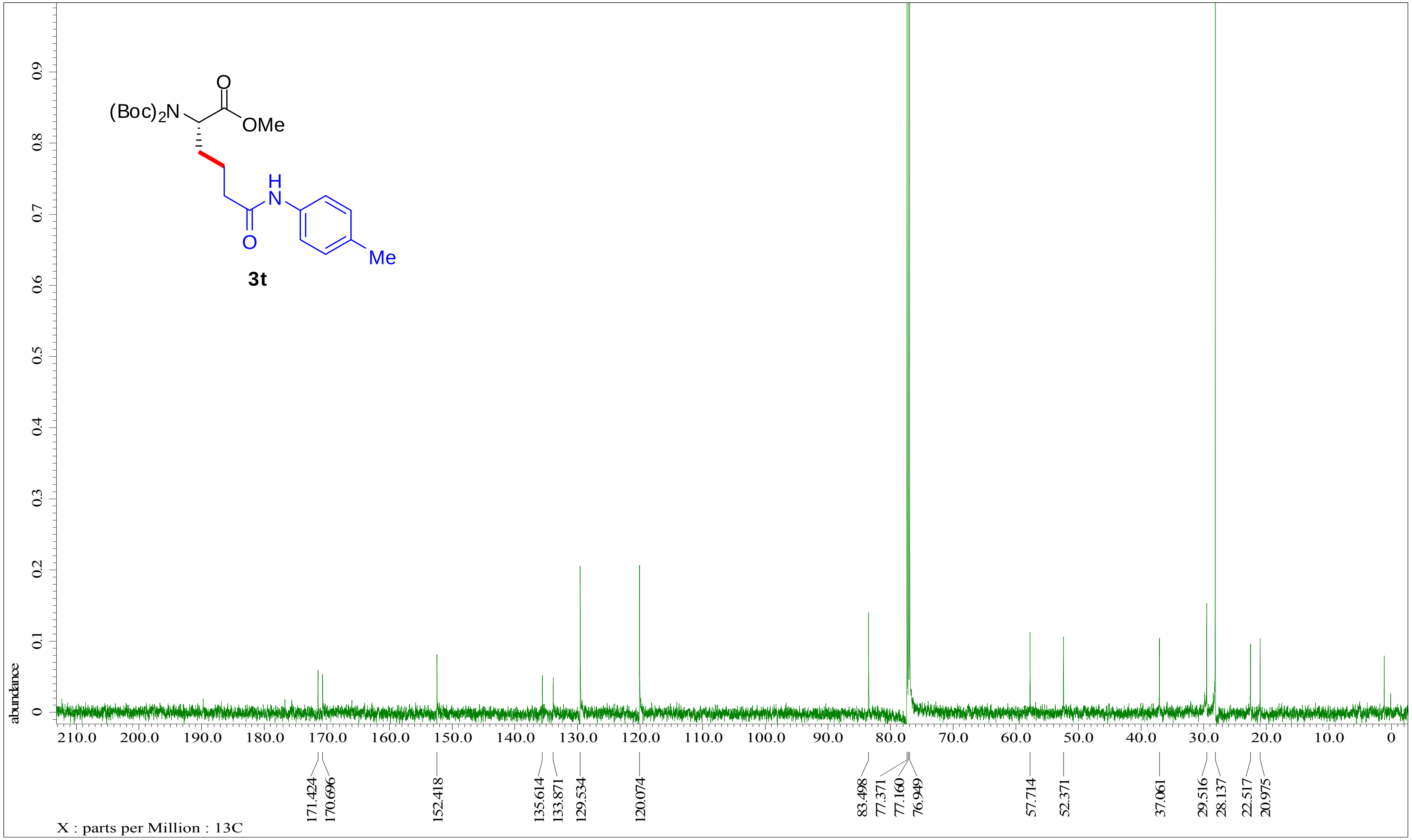
(Boc)2N
O
OMe
H
N
O
Me
3t
abundance
0.9
0.8
0.7
0.6
0.5
0.4
0.3
0.2
0.1
0
210.0 200.0 190.0 180.0 170.0 160.0 150.0 140.0 130.0 120.0 110.0 100.0 90.0 80.0 70.0 60.0 50.0 40.0 30.0 20.0 10.0 0
171.424
170.696
152.418
135.614
133.871
129.534
120.074
83.498
77.371
77.160
76.949
57.714
52.371
37.061
29.516
28.137
22.517
20.975
X : parts per Million : 13C

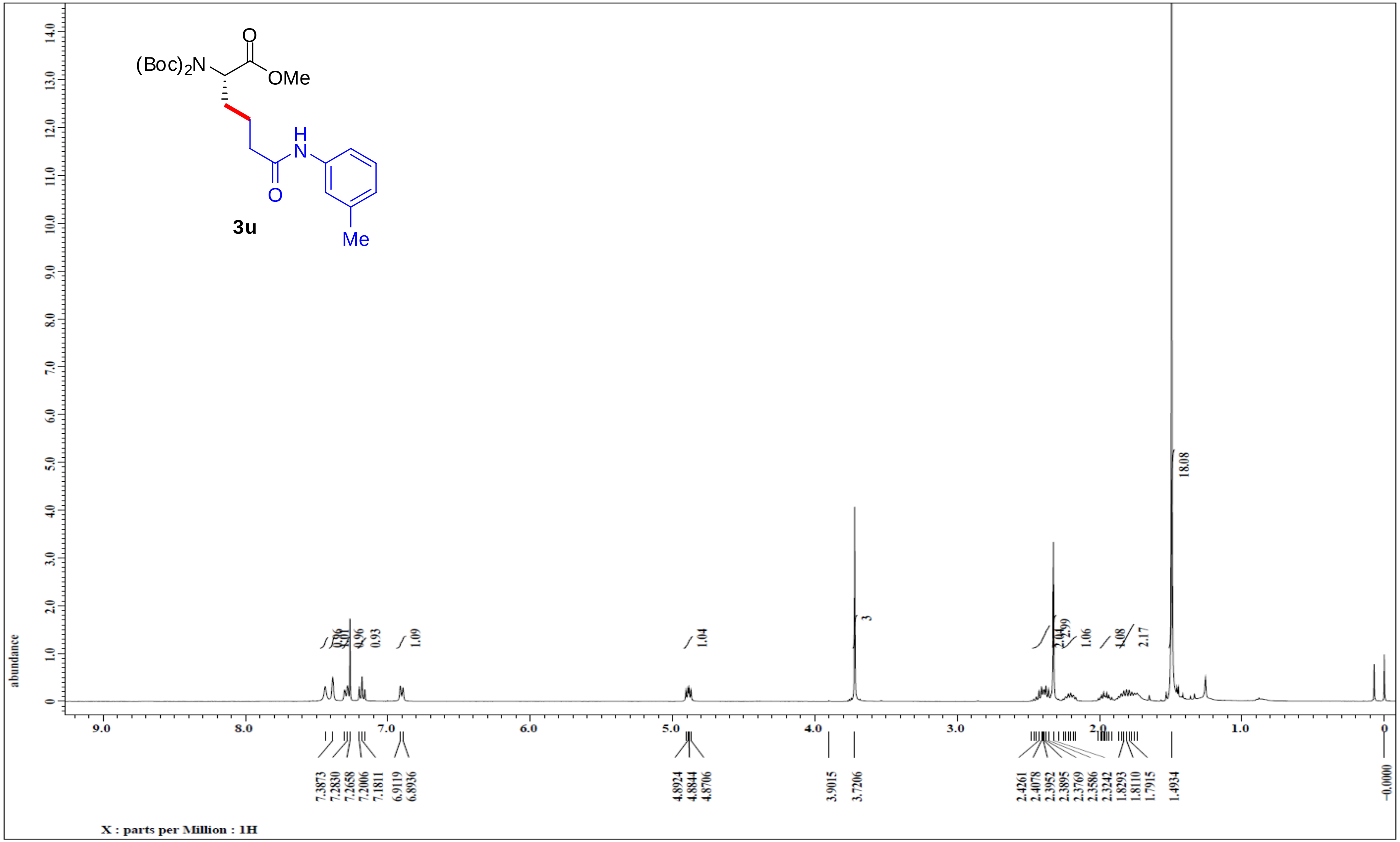
(Boc)2N
O
OMe
H
N
O
3u
Me
abundance
X : parts per Million : 1H
7.3873
7.2830
7.2658
7.2006
7.1811
6.9119
6.8936
4.8924
4.8844
4.8706
3.9015
3.7206
2.4261
2.4078
2.3952
2.3895
2.3769
2.3586
2.3242
1.8293
1.8110
1.7915
1.4934
-0.0000
1.09
0.93
0.96
1.04
3
1.06
1.08
2.17
18.08

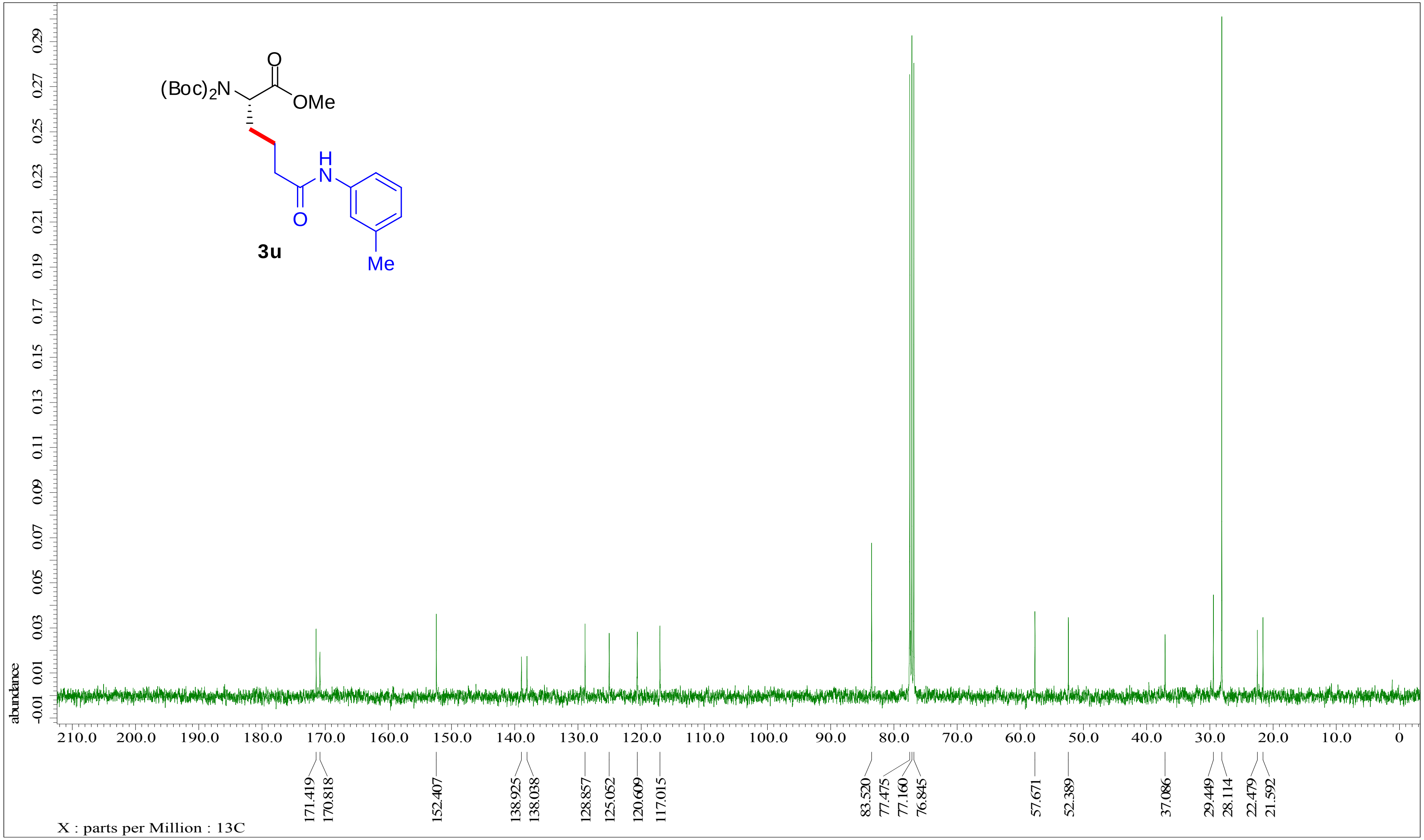

(Boc)2N
O
OMe
H
N
O
Me
3u
171.419
170.818
152.407
138.925
138.038
128.857
125.052
120.609
117.015
83.520
77.475
77.160
76.845
57.671
52.389
37.086
29.449
28.114
22.479
21.592
X : parts per Million : 13C
abundance

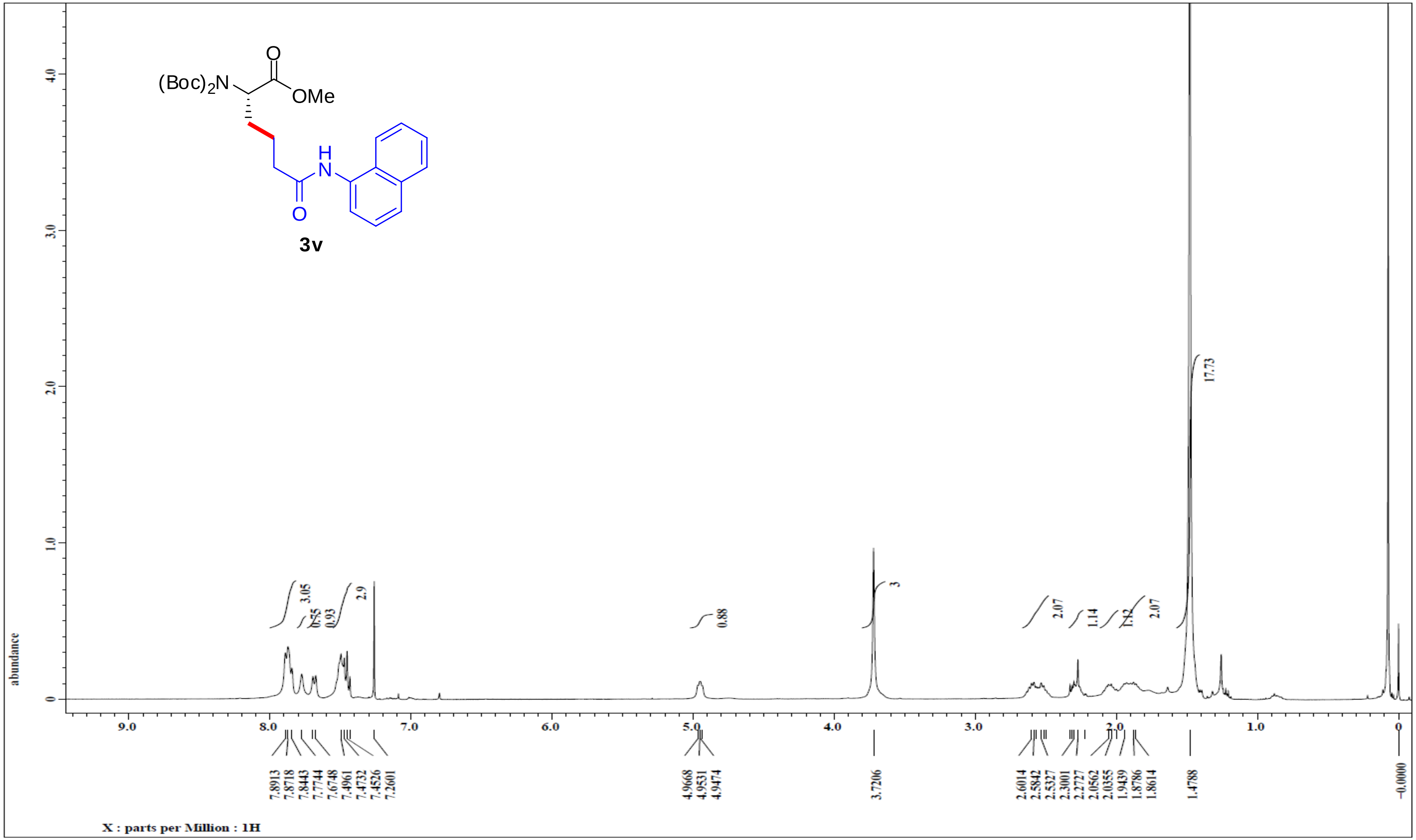
(Boc)2N
O
OMe
H
N
O
3v
abundance
X : parts per Million : 1H
3.05
0.75
0.93
2.9
0.88
3
2.07
1.14
1.12
2.07
17.73
7.8913
7.8718
7.8443
7.7744
7.6748
7.4961
7.4732
7.4526
7.2601
4.9668
4.9531
4.9474
3.7206
2.6014
2.5842
2.5327
2.3001
2.2727
2.0562
2.0355
1.9439
1.8786
1.8614
1.4788
-0.0000

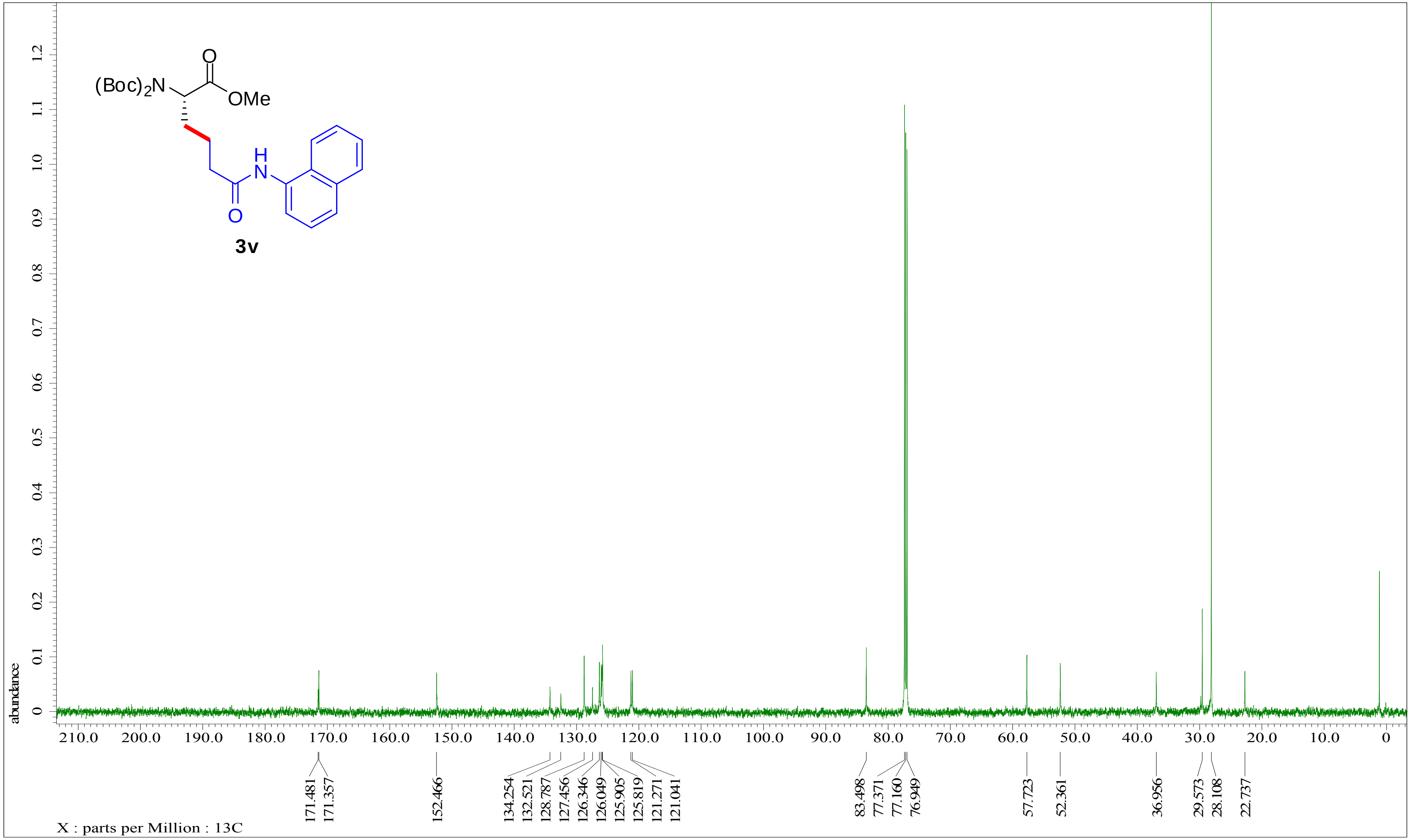

(Boc)2N
O
OMe
H
N
O
3v
abundance
0
0.1
0.2
0.3
0.4
0.5
0.6
0.7
0.8
0.9
1.0
1.1
1.2
210.0
200.0
190.0
180.0
170.0
160.0
150.0
140.0
130.0
120.0
110.0
100.0
90.0
80.0
70.0
60.0
50.0
40.0
30.0
20.0
10.0
0
171.481
171.357
152.466
134.254
132.521
128.787
127.456
126.346
126.049
125.905
125.819
121.271
121.041
83.498
77.371
77.160
76.949
57.723
52.361
36.956
29.573
28.108
22.737
X : parts per Million : 13C

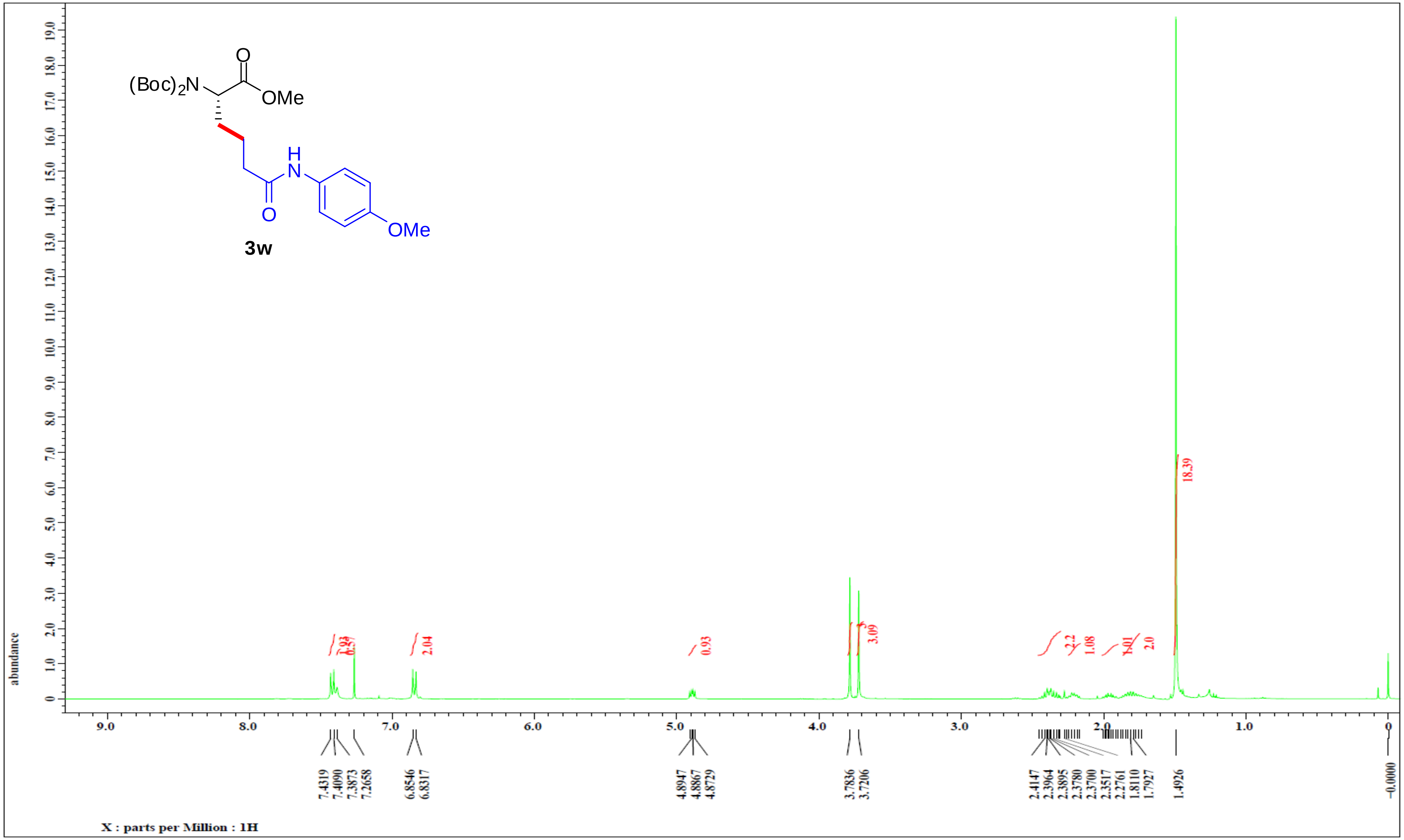
(Boc)2N
OMe
H
N
O
OMe
3w
abundance
X : parts per Million : 1H
7.4319
7.4090
7.3873
7.2658
6.8546
6.8317
4.8947
4.8867
4.8729
3.7836
3.7206
2.4147
2.3964
2.3895
2.3780
2.3700
2.3517
2.2761
1.8110
1.7927
1.4926
-0.0000
18.39
3.09
2.04
0.93
1.08
2.0

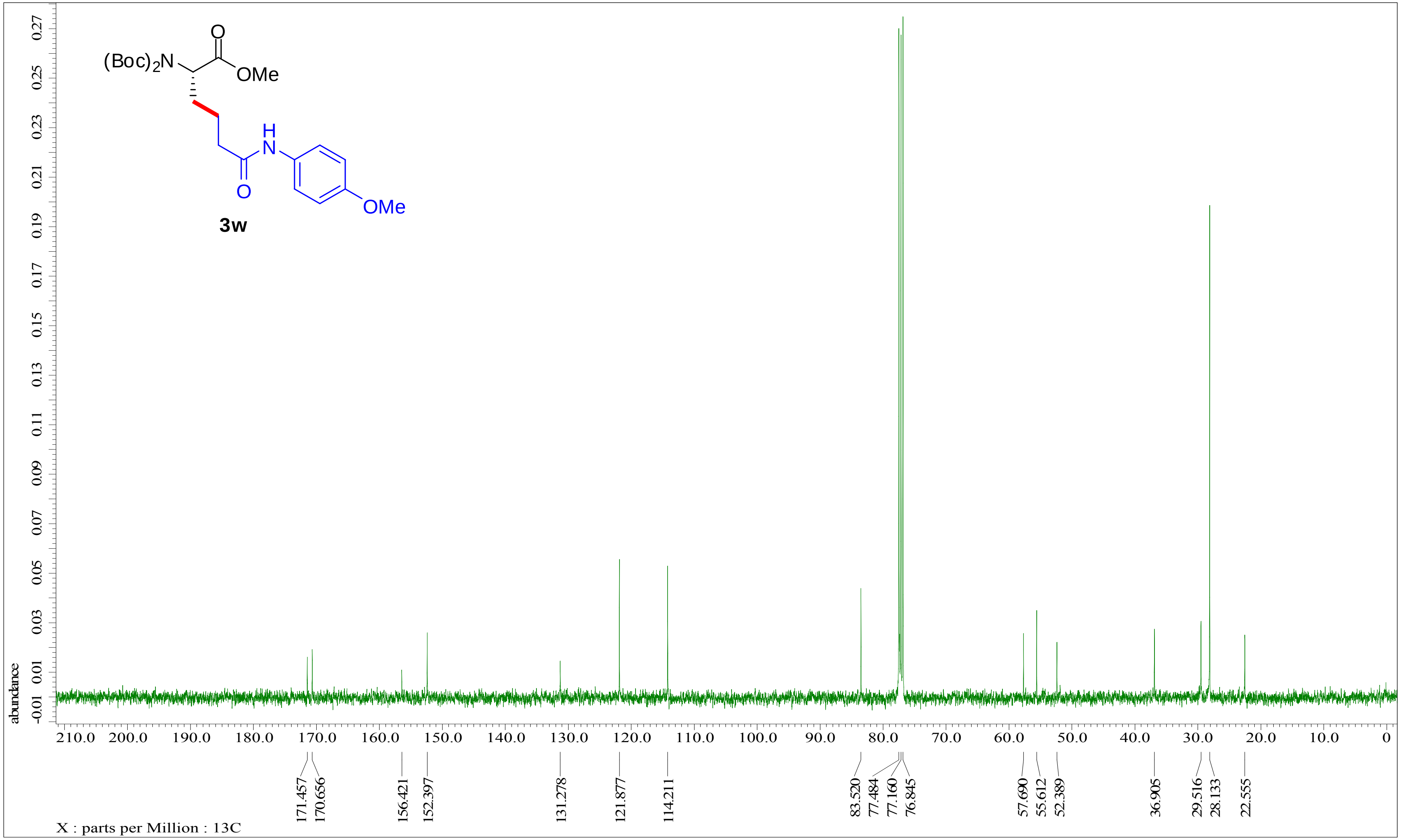

(Boc)$_2$N
O
OMe
H
N
O
OMe
3w
abundance
X : parts per Million : 13C
171.457
170.656
156.421
152.397
131.278
121.877
114.211
83.520
77.484
77.160
76.845
57.690
55.612
52.389
36.905
29.516
28.133
22.555

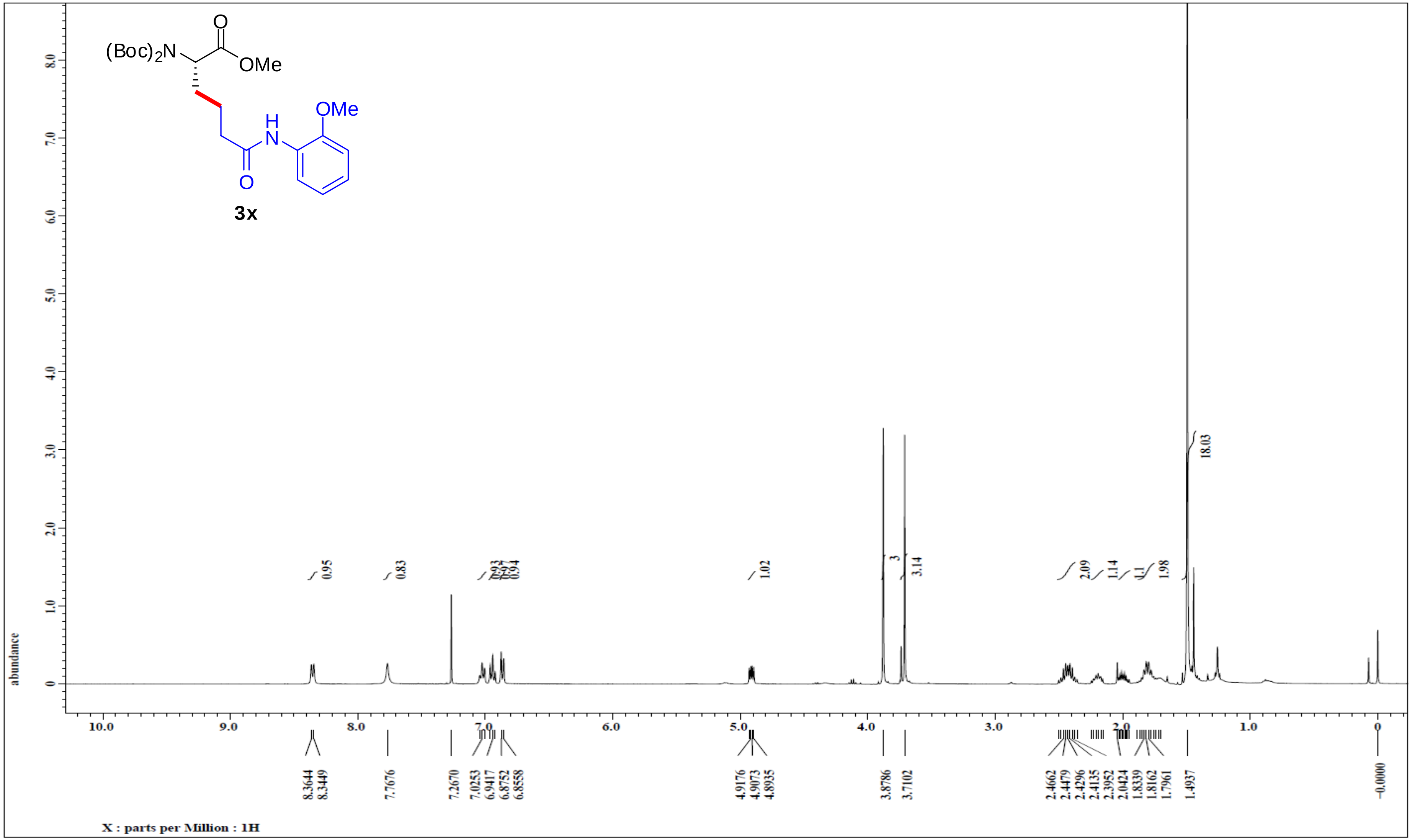
(Boc)2N
OMe
O
H
N
OMe
O
3x
abundance
X : parts per Million : 1H
0.95
0.83
1.02
3
3.14
2.09
1.14
1.1
1.98
18.03
8.3644
8.3449
7.7676
7.2670
7.0253
6.9417
6.8752
6.8558
4.9176
4.9073
4.8935
3.8786
3.7102
2.4662
2.4479
2.4296
2.4135
2.3952
2.0424
1.8339
1.8162
1.7961
1.4937
-0.0000

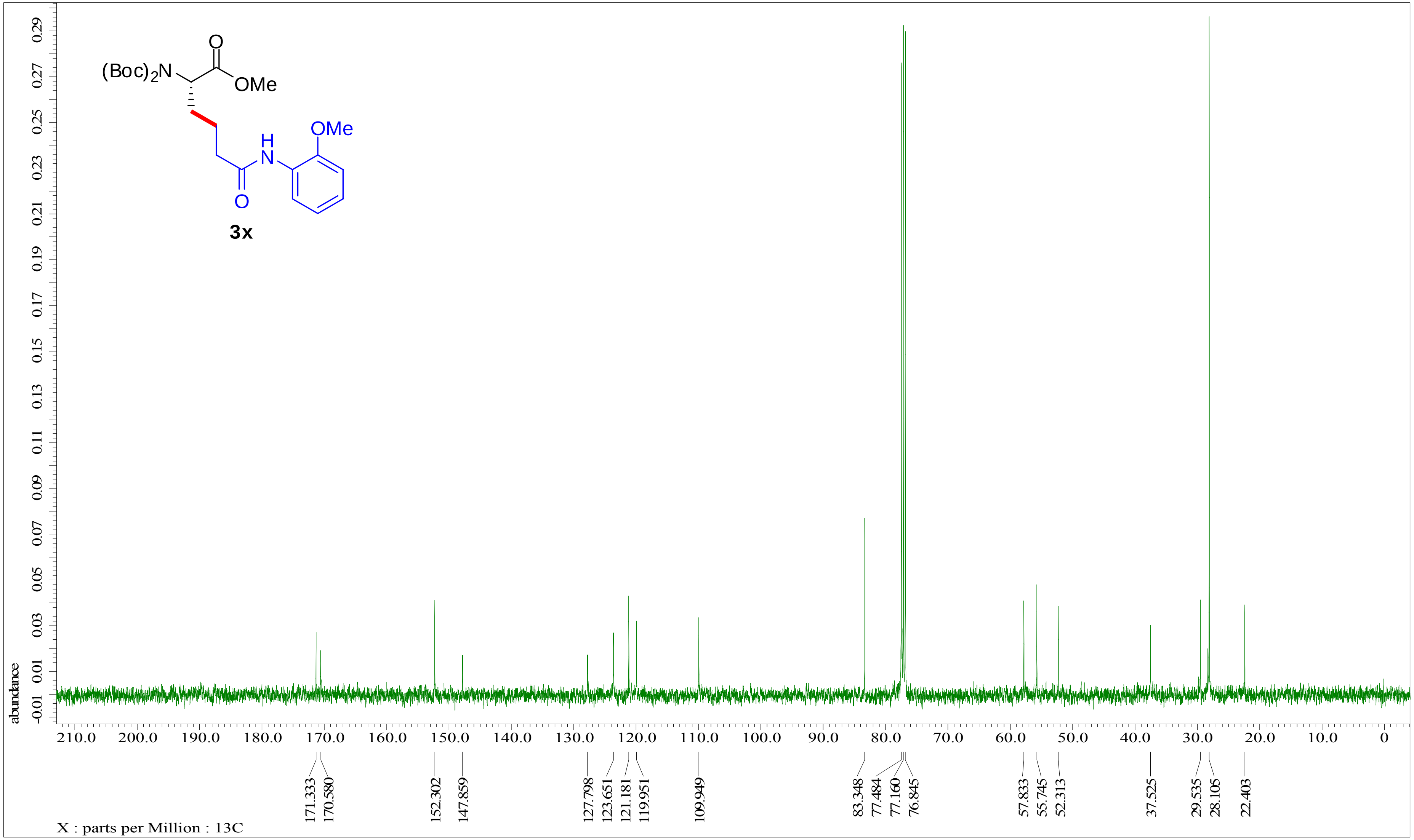
(Boc)2N
O
OMe
H
N
OMe
O
3x
abundance
X : parts per Million : 13C
171.333
170.580
152.302
147.859
127.798
123.651
121.181
119.951
109.949
83.348
77.484
77.160
76.845
57.833
55.745
52.313
37.525
29.535
28.105
22.403

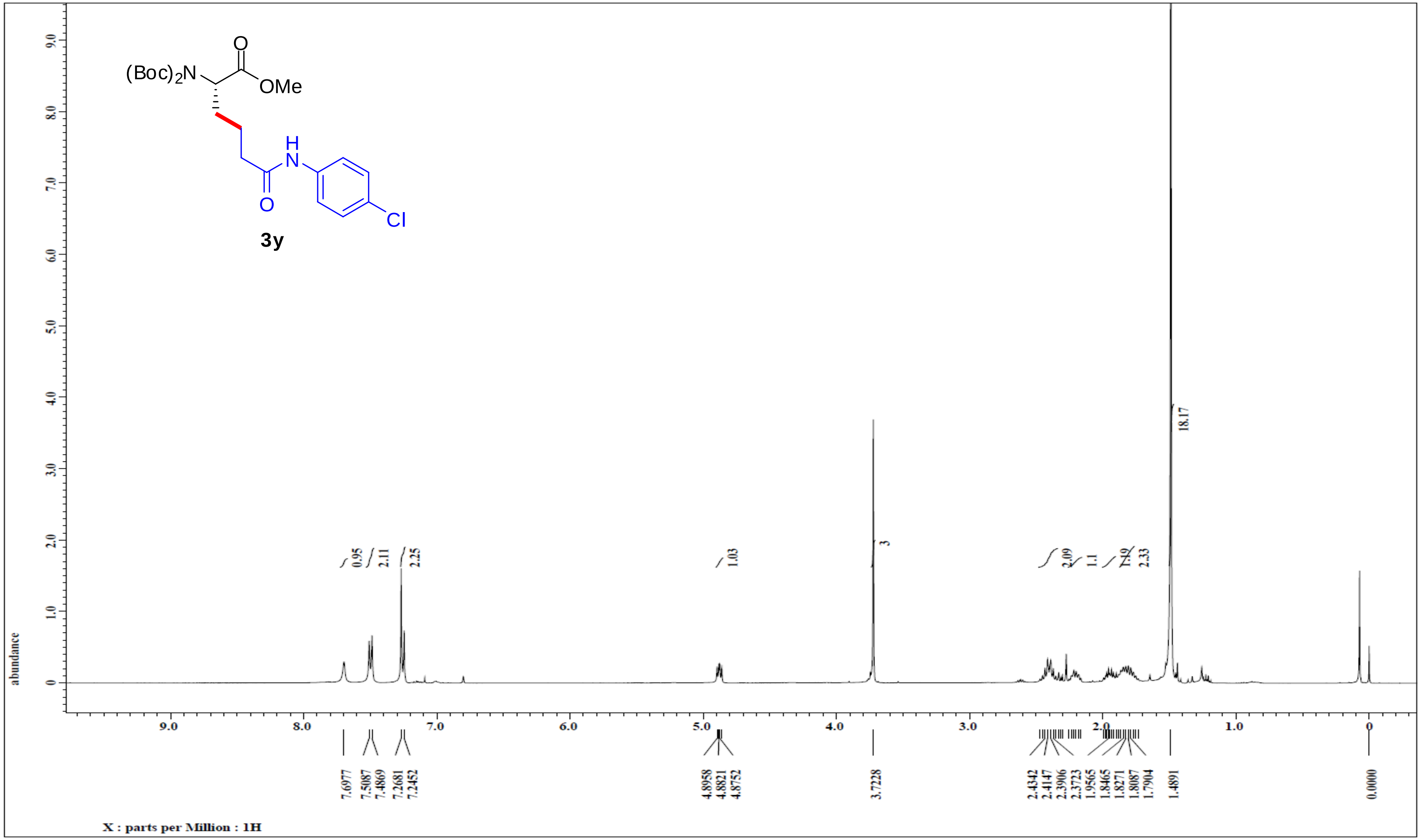
(Boc)2N
OMe
O
H
N
O
Cl
3y
abundance
X : parts per Million : 1H
0.95
2.11
2.25
1.03
3
2.09
1.1
1.19
2.33
18.17
7.6977
7.5087
7.4869
7.2681
7.2452
4.8958
4.8821
4.8752
3.7228
2.4342
2.4147
2.3906
2.3723
1.9565
1.8465
1.8271
1.8087
1.7904
1.4891
0.0000

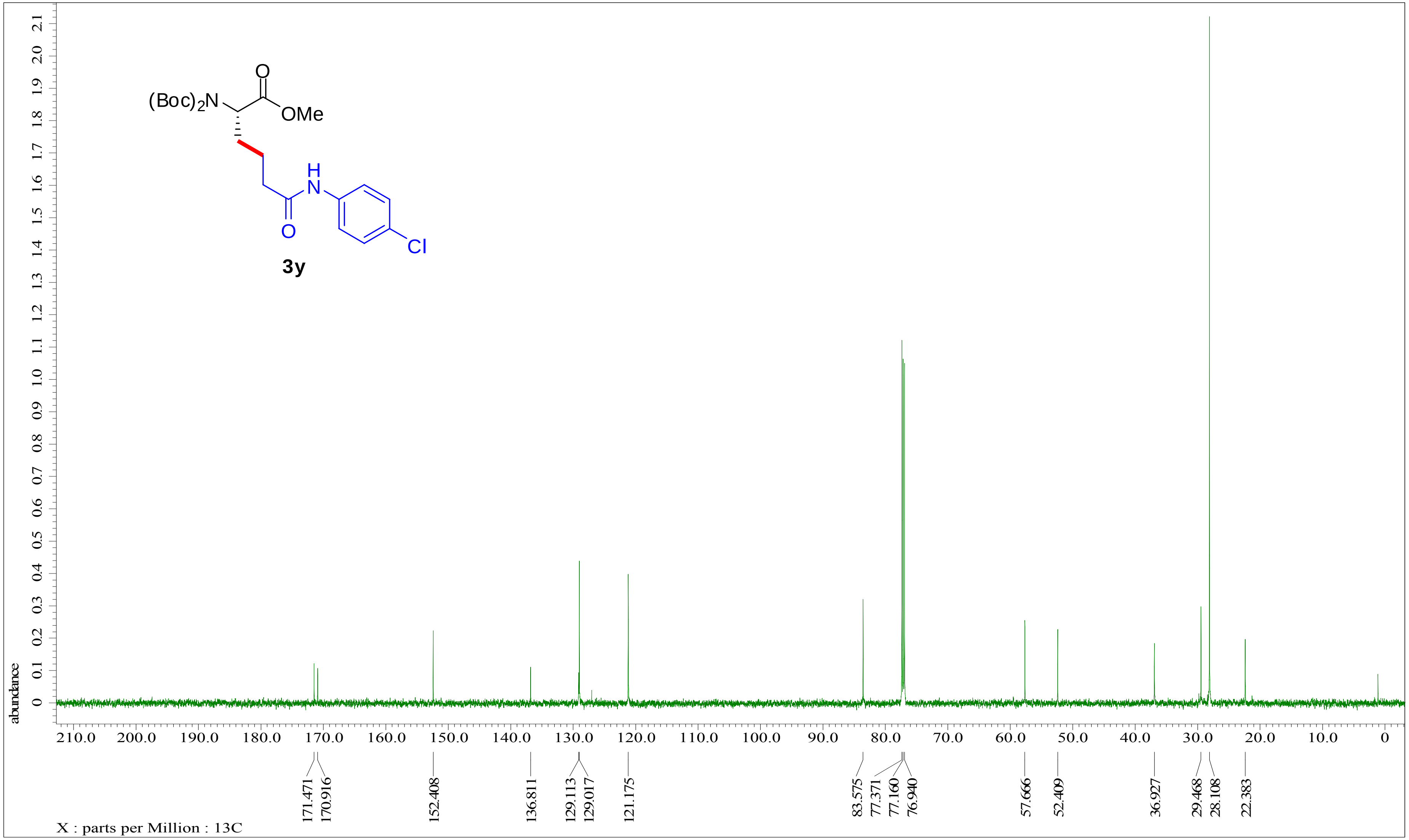

(Boc)2N
O
OMe
H
N
O
Cl
3y
abundance
171.471
170.916
152.408
136.811
129.113
129.017
121.175
83.575
77.371
77.160
76.940
57.666
52.409
36.927
29.468
28.108
22.383
X : parts per Million : 13C

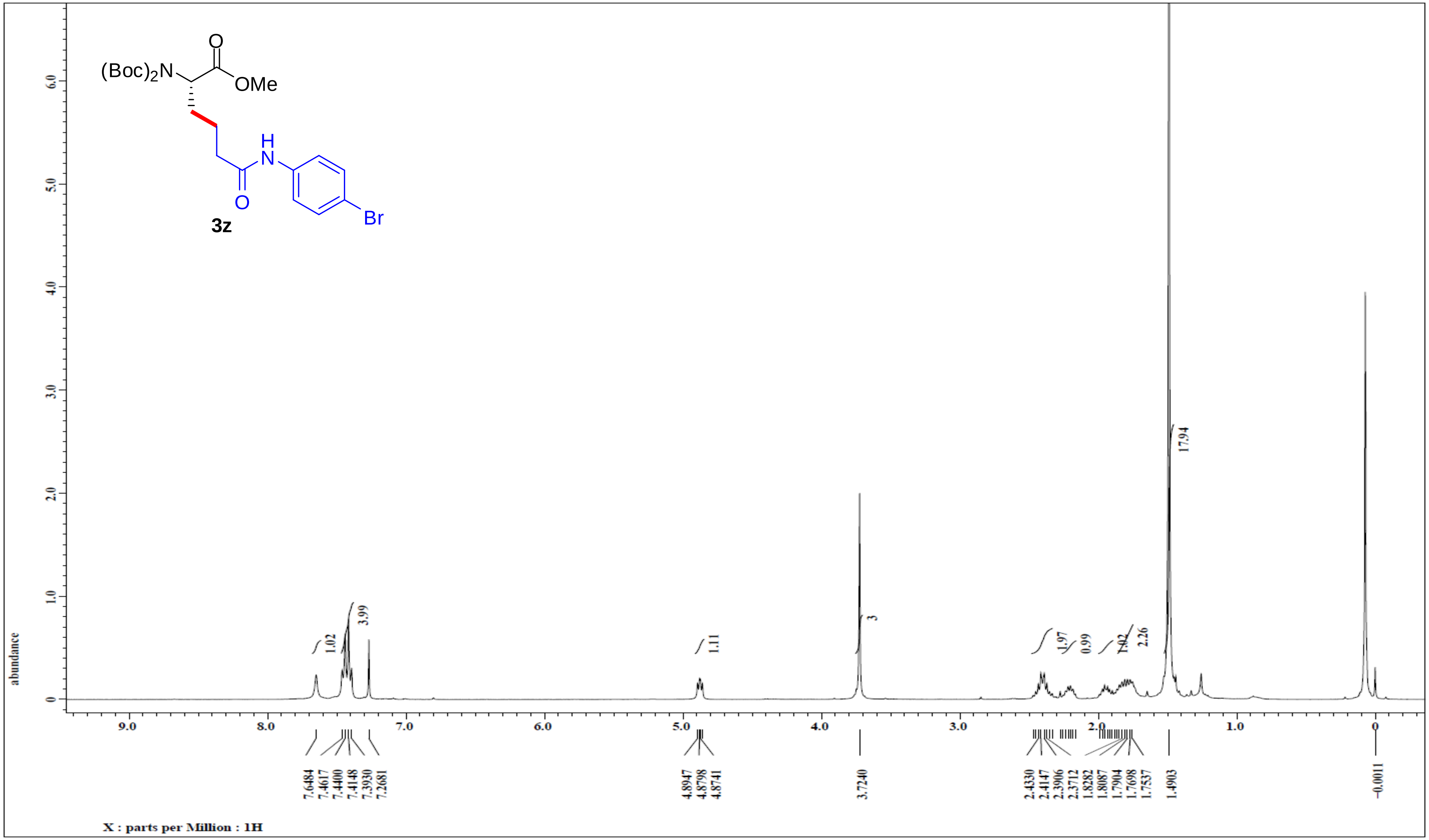
(Boc)2N
OMe
O
H
N
O
Br
3z
abundance
17.94
3.99
1.02
1.11
3
1.97
0.99
1.02
2.26
7.6484
7.4617
7.4400
7.4148
7.3930
7.2681
4.8947
4.8798
4.8741
3.7240
2.4330
2.4147
2.3906
2.3712
1.8282
1.8087
1.7904
1.7698
1.7537
1.4903
-0.0011
X : parts per Million : 1H

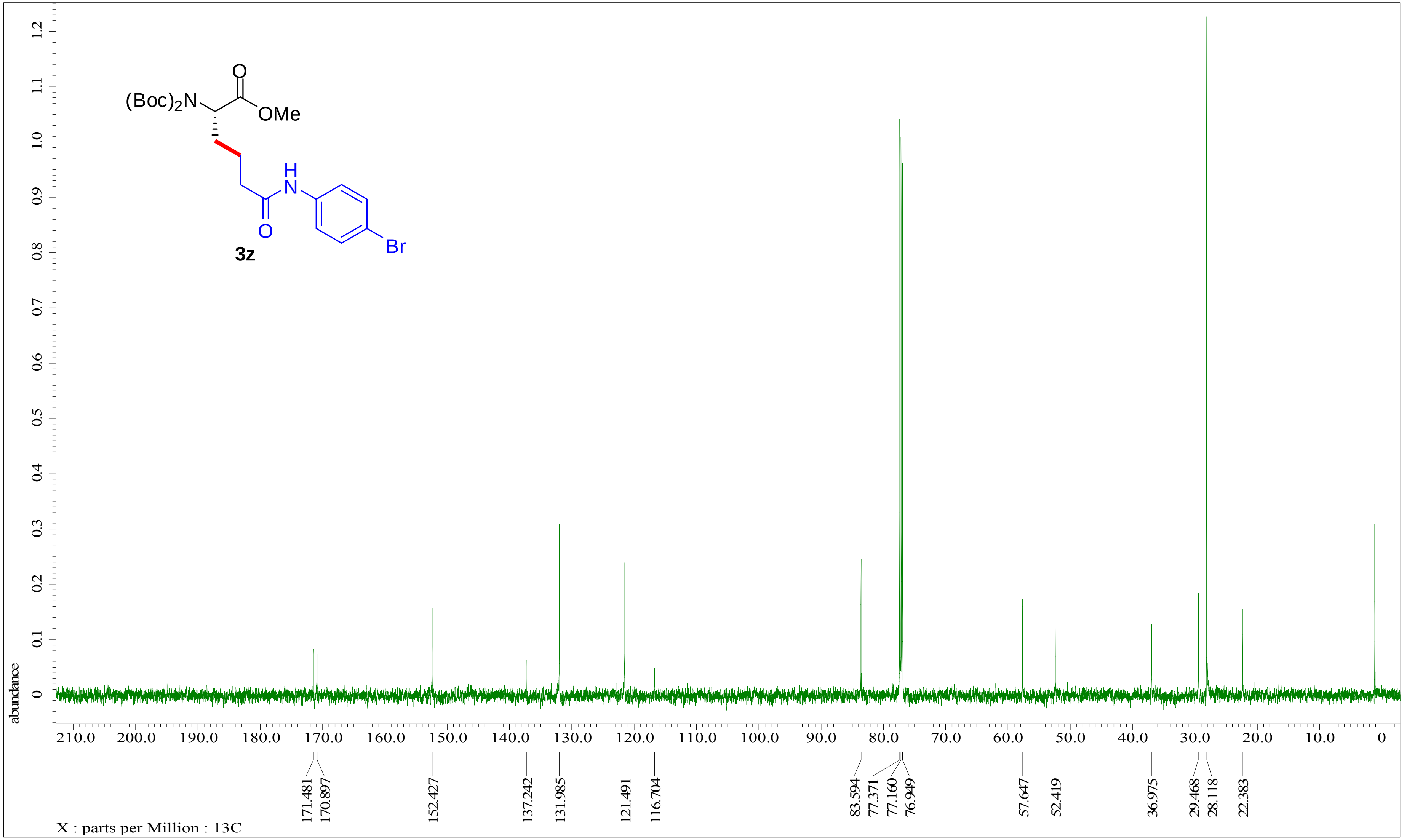
(Boc)2N
O
OMe
H
N
O
Br
3z
abundance
X : parts per Million : 13C
171.481
170.897
152.427
137.242
131.985
121.491
116.704
83.594
77.371
77.160
76.949
57.647
52.419
36.975
29.468
28.118
22.383

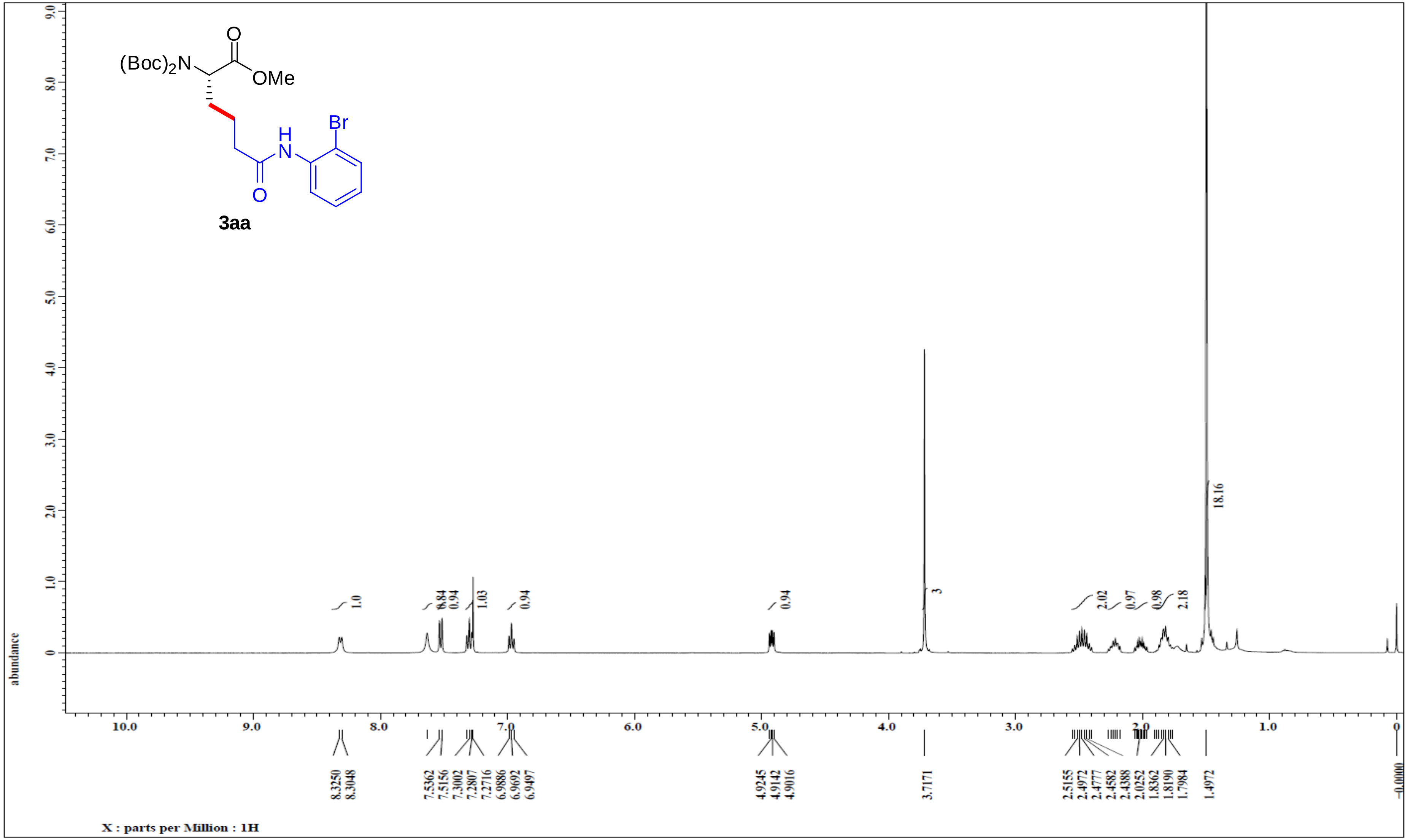

(Boc)2N
O
OMe
H
N
Br
O
3aa
abundance
X : parts per Million : 1H
8.3250
8.3048
7.5362
7.5156
7.3002
7.2807
7.2716
6.9886
6.9692
6.9497
4.9245
4.9142
4.9016
3.7171
2.5155
2.4972
2.4777
2.4582
2.4388
2.0252
1.8362
1.8190
1.7984
1.4972
0.0000
1.0
0.84
0.94
1.03
0.94
0.94
3
2.02
0.97
0.98
2.18
18.16

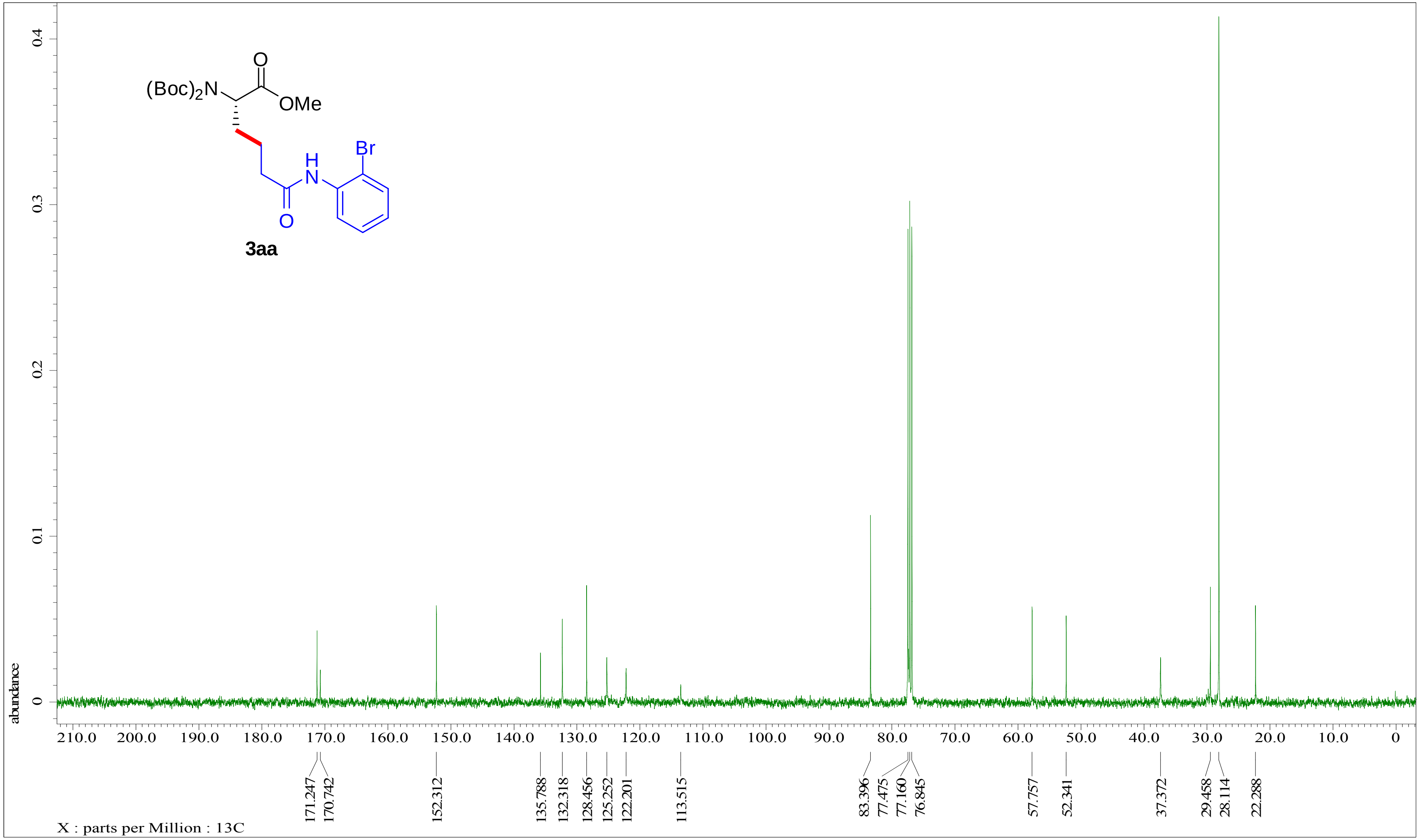
(Boc)2N
O
OMe
H
N
Br
O
3aa
abundance
0.4
0.3
0.2
0.1
0
210.0 200.0 190.0 180.0 170.0 160.0 150.0 140.0 130.0 120.0 110.0 100.0 90.0 80.0 70.0 60.0 50.0 40.0 30.0 20.0 10.0 0
171.247
170.742
152.312
135.788
132.318
128.456
125.252
122.201
113.515
83.396
77.475
77.160
76.845
57.757
52.341
37.372
29.458
28.114
22.288
X : parts per Million : 13C

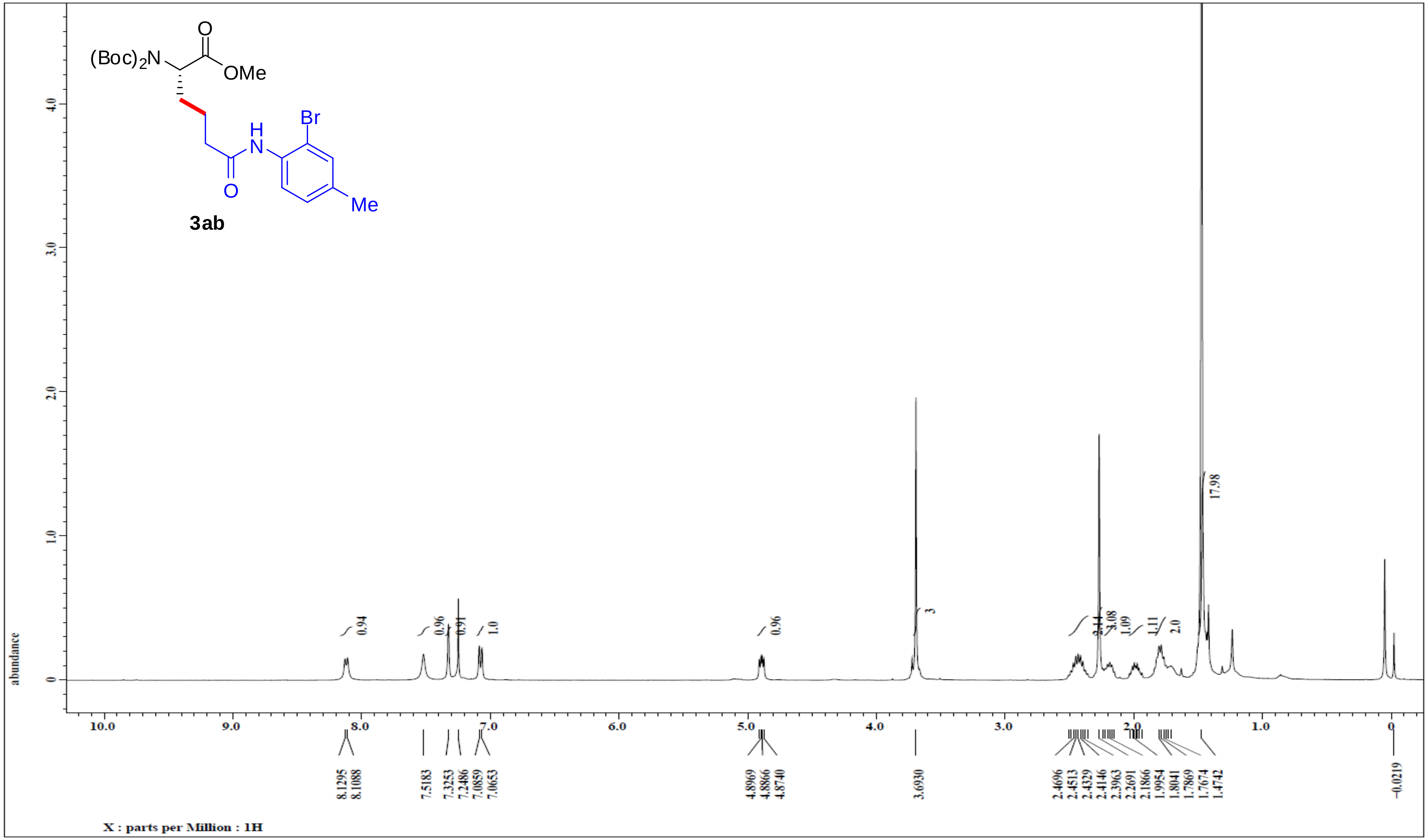

(Boc)2N
O
OMe
H
N
Br
O
Me
3ab
abundance
X : parts per Million : 1H
0.94
0.96
0.91
1.0
0.96
3
2.14
3.08
1.09
1.11
2.0
17.98
8.1295
8.1088
7.5183
7.3253
7.2486
7.0859
7.0653
4.8969
4.8866
4.8740
3.6930
2.4696
2.4513
2.4329
2.4146
2.3963
2.2691
2.1866
1.9954
1.8041
1.7869
1.7674
1.4742
-0.0219

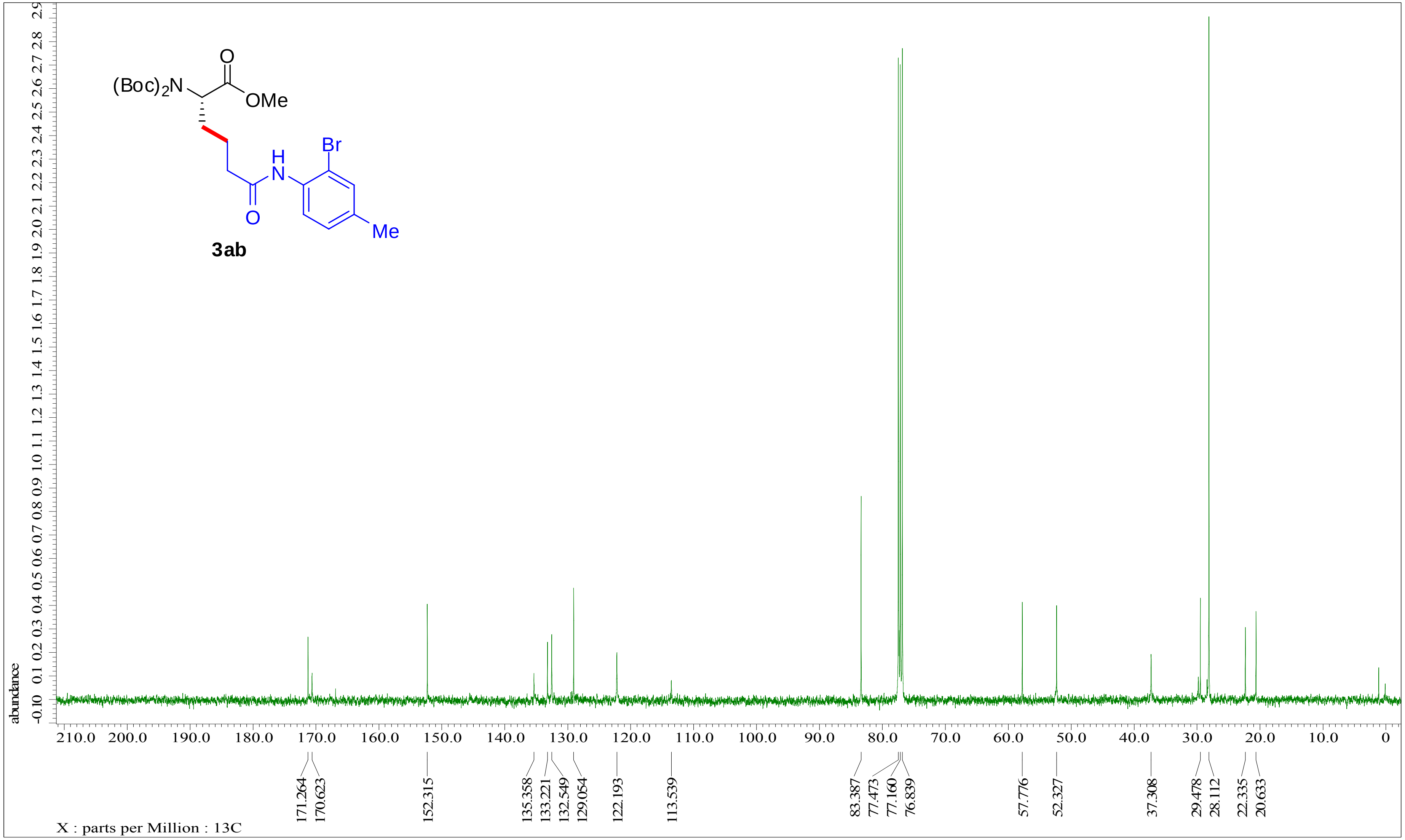

(Boc)2N
O
OMe
H
N
Br
O
Me
3ab
abundance
X : parts per Million : 13C
171.264
170.623
152.315
135.358
133.221
132.549
129.054
122.193
113.539
83.387
77.473
77.160
76.839
57.776
52.327
37.308
29.478
28.112
22.335
20.633

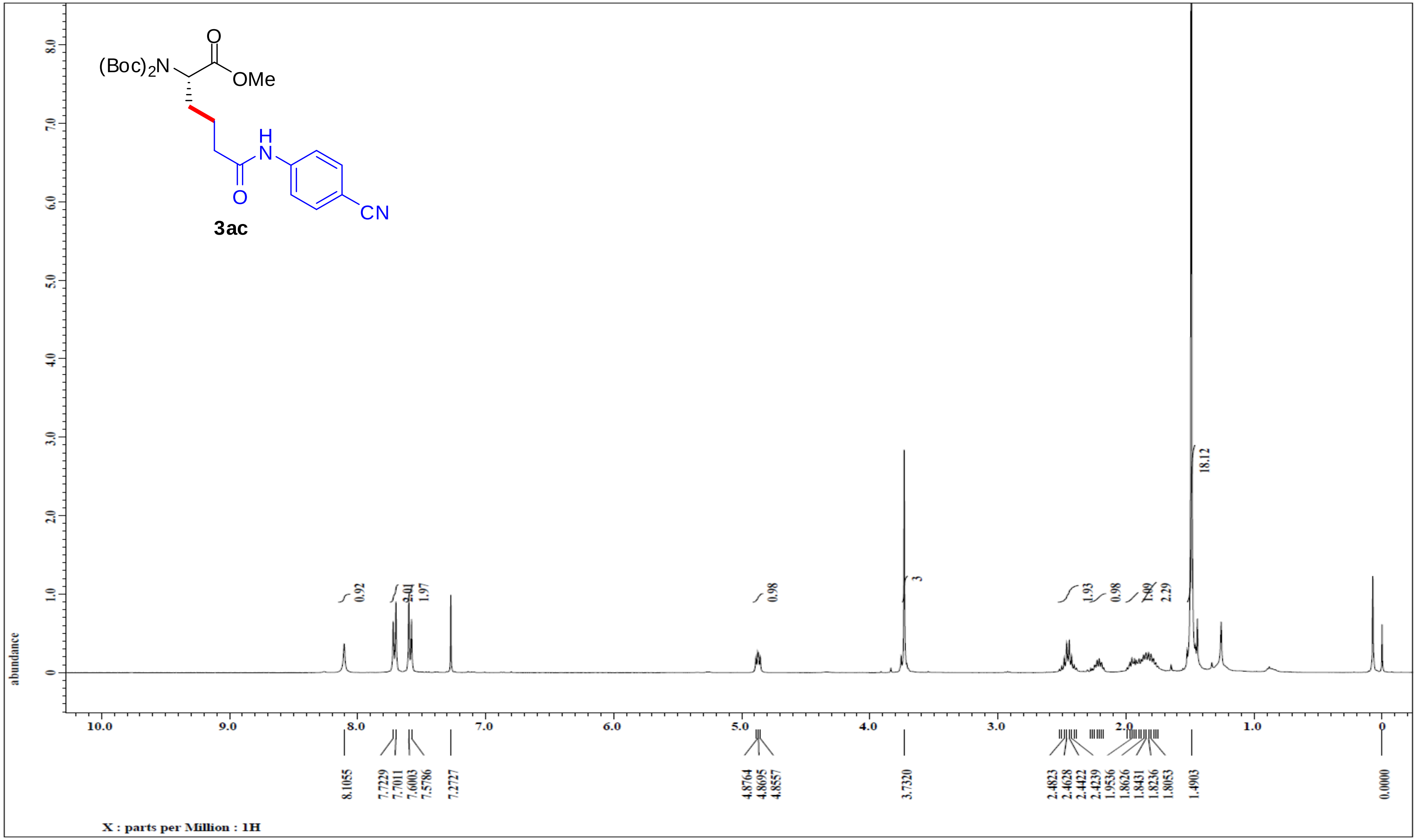
(Boc)2N
OMe
O
H
N
O
CN
3ac
abundance
X : parts per Million : 1H
0.92
2.01
1.97
0.98
3
1.93
0.98
18.12
2.29
8.1055
7.7229
7.7011
7.6003
7.5786
7.2727
4.8764
4.8695
4.8557
3.7320
2.4823
2.4628
2.4422
2.4239
1.9536
1.8626
1.8431
1.8236
1.8053
1.4903
0.0000

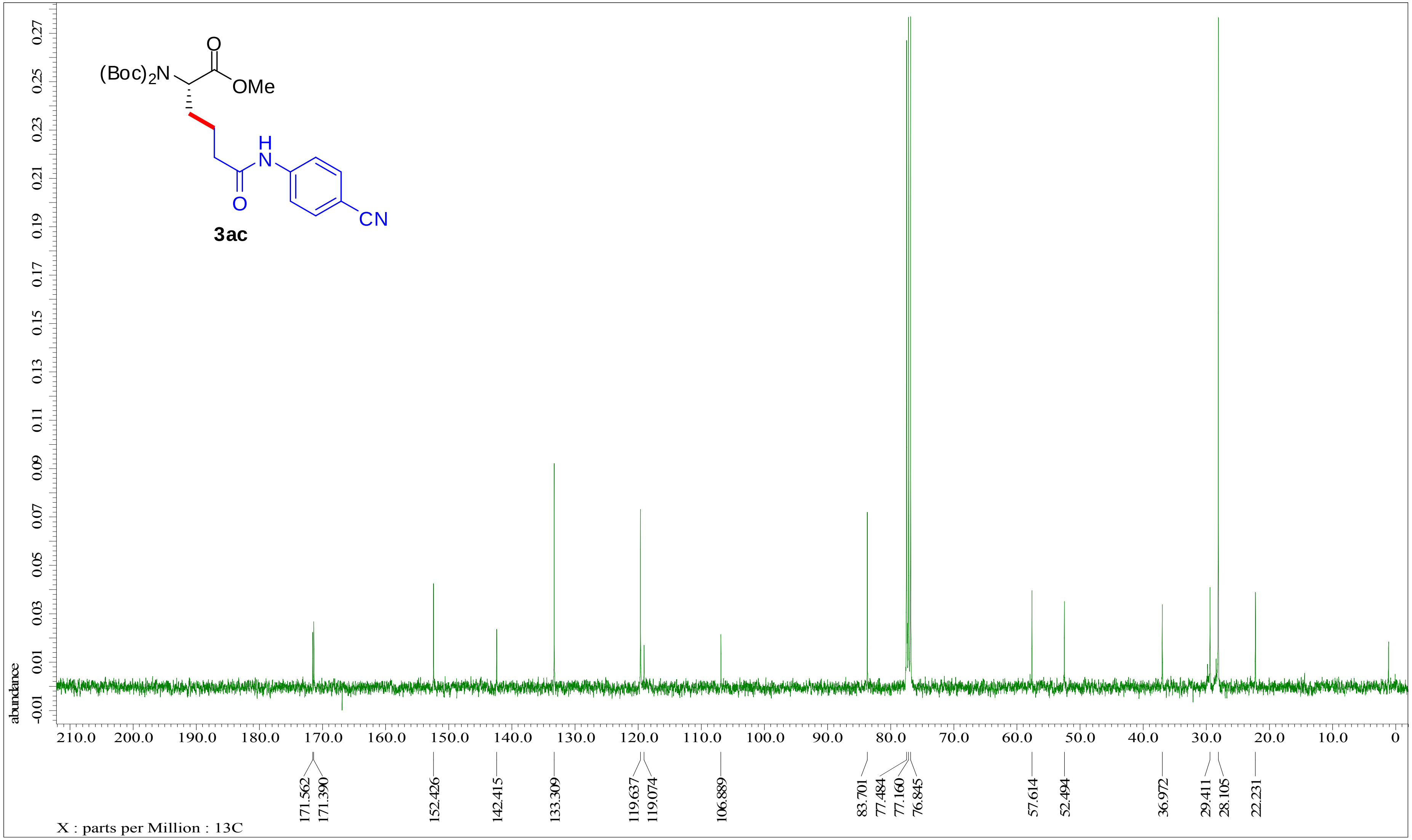
(Boc)2N
O
OMe
H
N
O
CN
3ac
abundance
0.27
0.25
0.23
0.21
0.19
0.17
0.15
0.13
0.11
0.09
0.07
0.05
0.03
0.01
-0.01
210.0 200.0 190.0 180.0 170.0 160.0 150.0 140.0 130.0 120.0 110.0 100.0 90.0 80.0 70.0 60.0 50.0 40.0 30.0 20.0 10.0 0
171.562
171.390
152.426
142.415
133.309
119.637
119.074
106.889
83.701
77.484
77.160
76.845
57.614
52.494
36.972
29.411
28.105
22.231
X : parts per Million : 13C

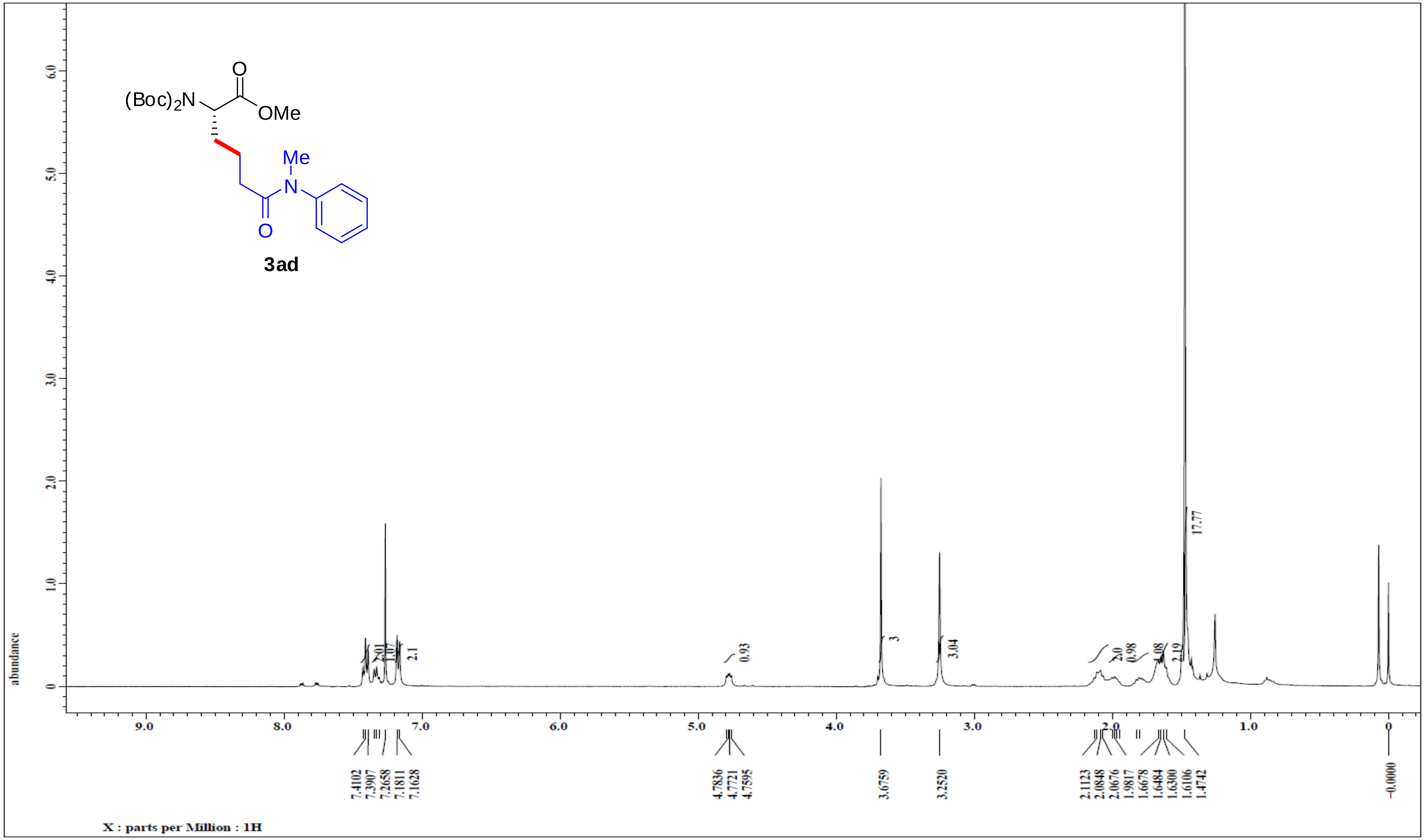

(Boc)2N
O
OMe
Me
N
O
3ad
abundance
X : parts per Million : 1H
7.4102
7.3907
7.2658
7.1811
7.1628
4.7836
4.7721
4.7595
3.6759
3.2520
2.1123
2.0848
2.0676
1.9817
1.6678
1.6484
1.6300
1.6106
1.4742
-0.0000
17.77

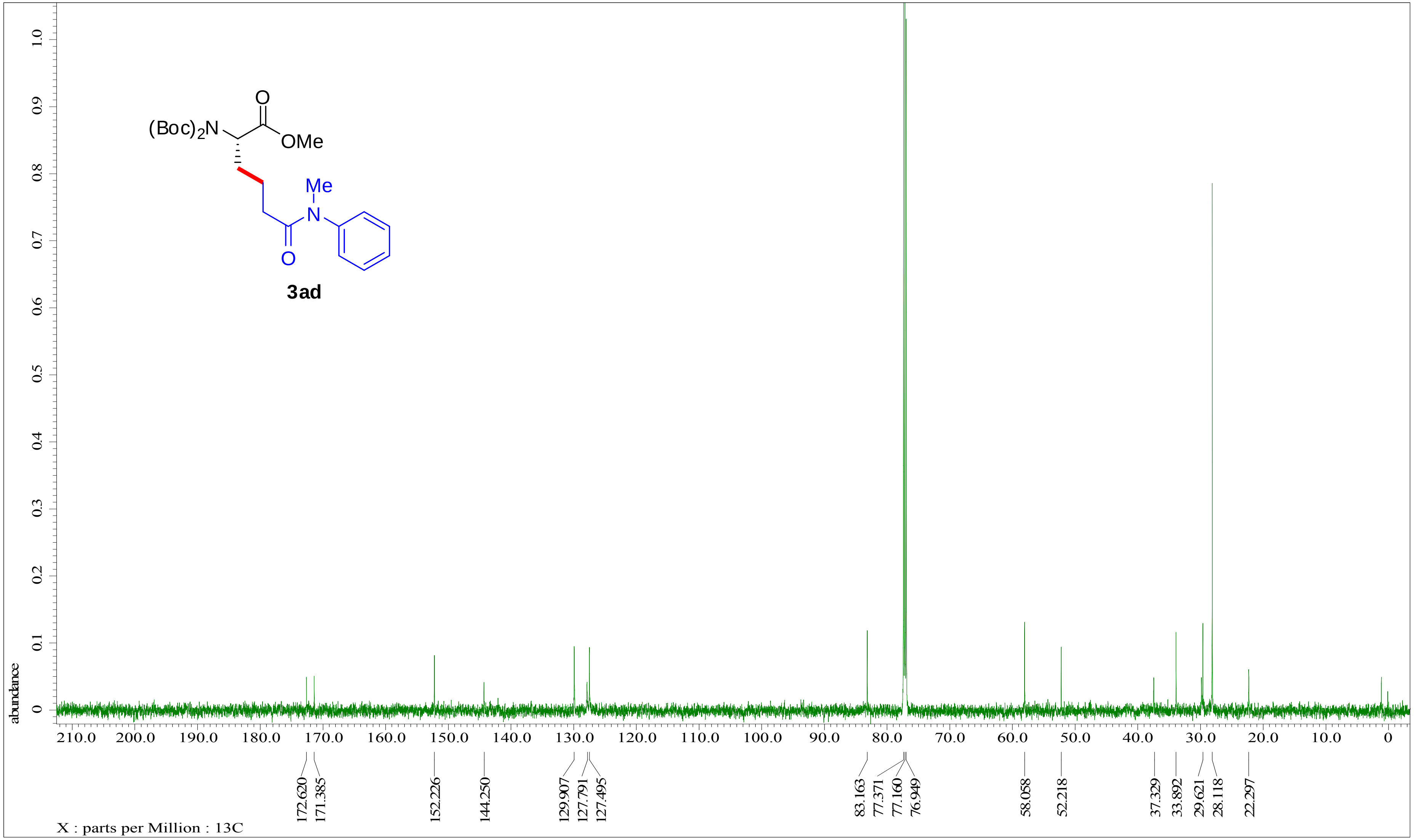
(Boc)$_2$N
O
OMe
Me
N
O
3ad
abundance
1.0
0.9
0.8
0.7
0.6
0.5
0.4
0.3
0.2
0.1
0
210.0 200.0 190.0 180.0 170.0 160.0 150.0 140.0 130.0 120.0 110.0 100.0 90.0 80.0 70.0 60.0 50.0 40.0 30.0 20.0 10.0 0
172.620
171.385
152.226
144.250
129.907
127.791
127.495
83.163
77.371
77.160
76.949
58.058
52.218
37.329
33.892
29.621
28.118
22.297
X : parts per Million : 13C

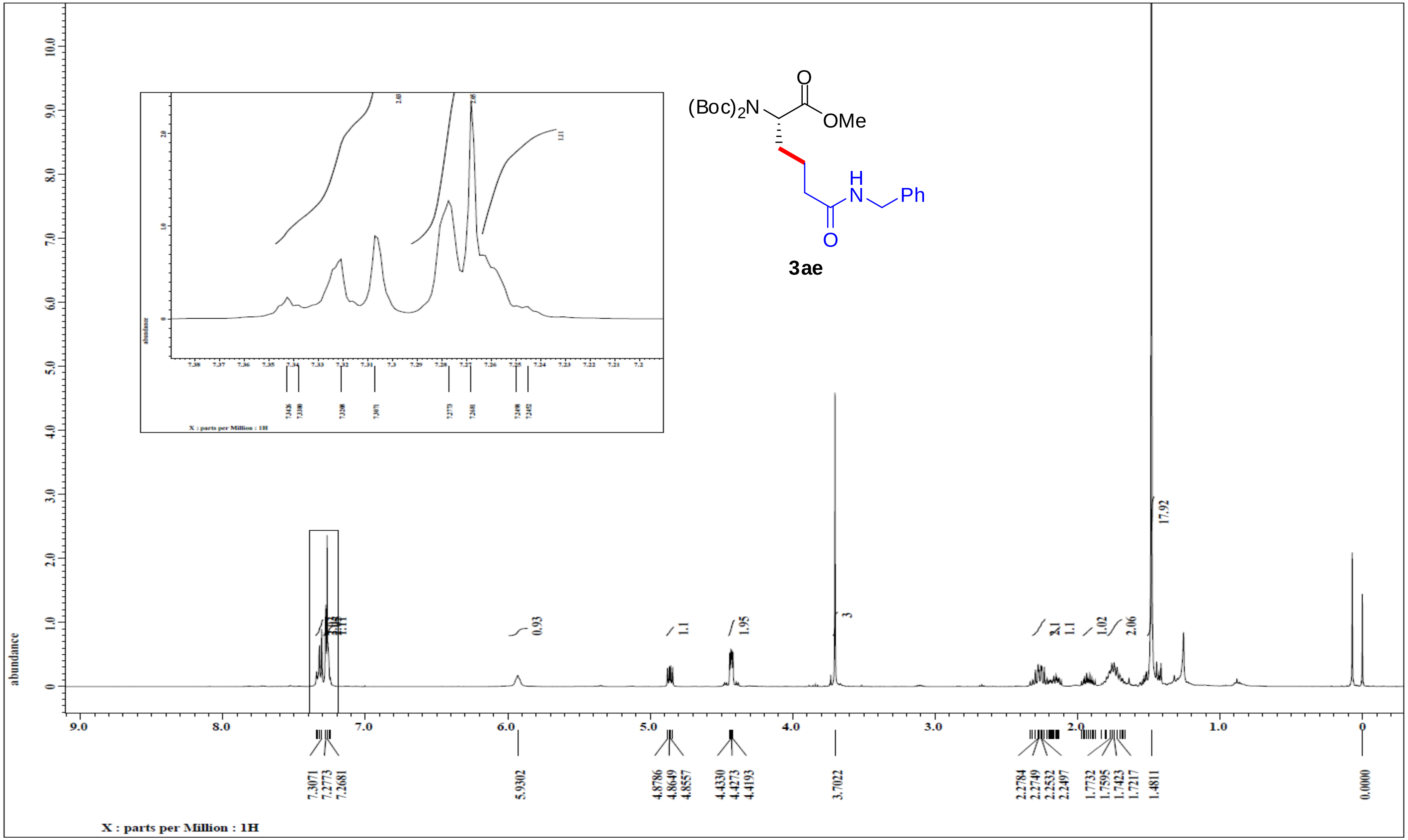
(Boc)2N
O
OMe
H
N
Ph
O
3ae
X : parts per Million : 1H
abundance
17.92
3
1.95
1.1
0.93
1.02
2.06
7.3071
7.2773
7.2681
5.9302
4.8786
4.8649
4.8557
4.4330
4.4273
4.4193
3.7022
2.2784
2.2749
2.2532
2.2497
1.7732
1.7595
1.7423
1.7217
1.4811
0.0000

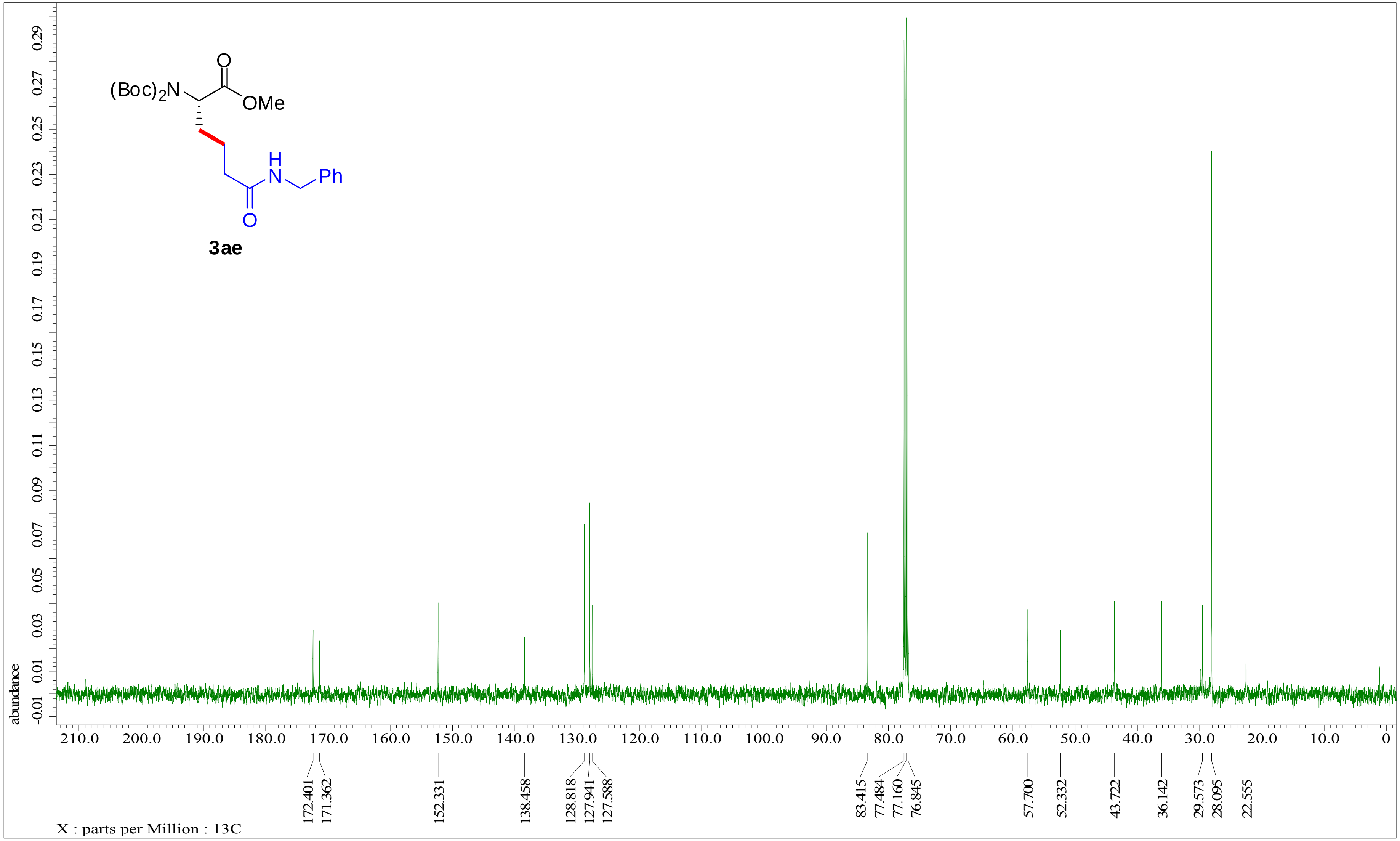
(Boc)2N
O
OMe
H
N
Ph
O
3ae
abundance
0.29
0.27
0.25
0.23
0.21
0.19
0.17
0.15
0.13
0.11
0.09
0.07
0.05
0.03
0.01
-0.01
210.0
200.0
190.0
180.0
170.0
160.0
150.0
140.0
130.0
120.0
110.0
100.0
90.0
80.0
70.0
60.0
50.0
40.0
30.0
20.0
10.0
0
172.401
171.362
152.331
138.458
128.818
127.941
127.588
83.415
77.484
77.160
76.845
57.700
52.332
43.722
36.142
29.573
28.095
22.555
X : parts per Million : 13C

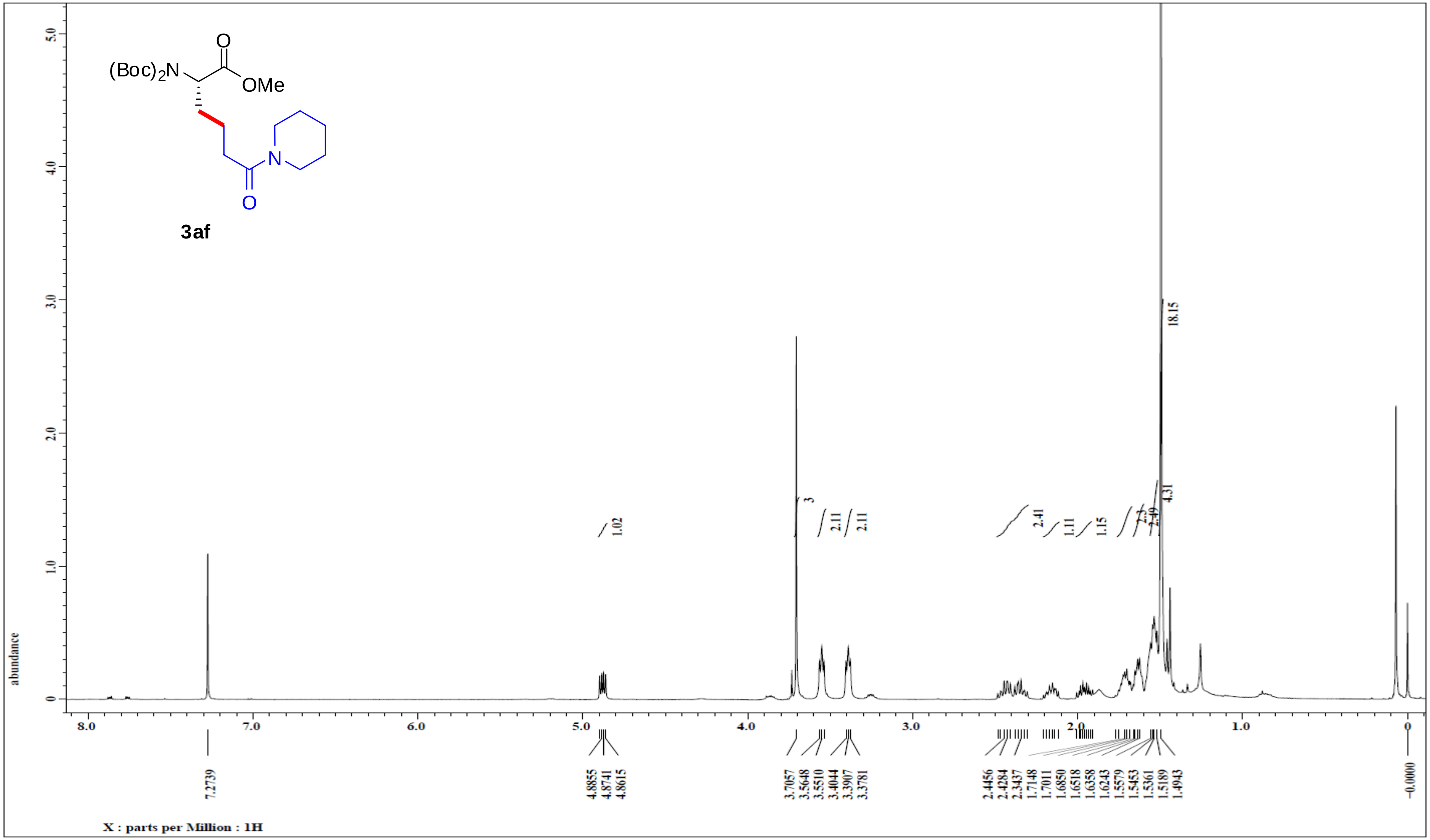

(Boc)2N
O
OMe
N
O
3af
X : parts per Million : 1H
abundance

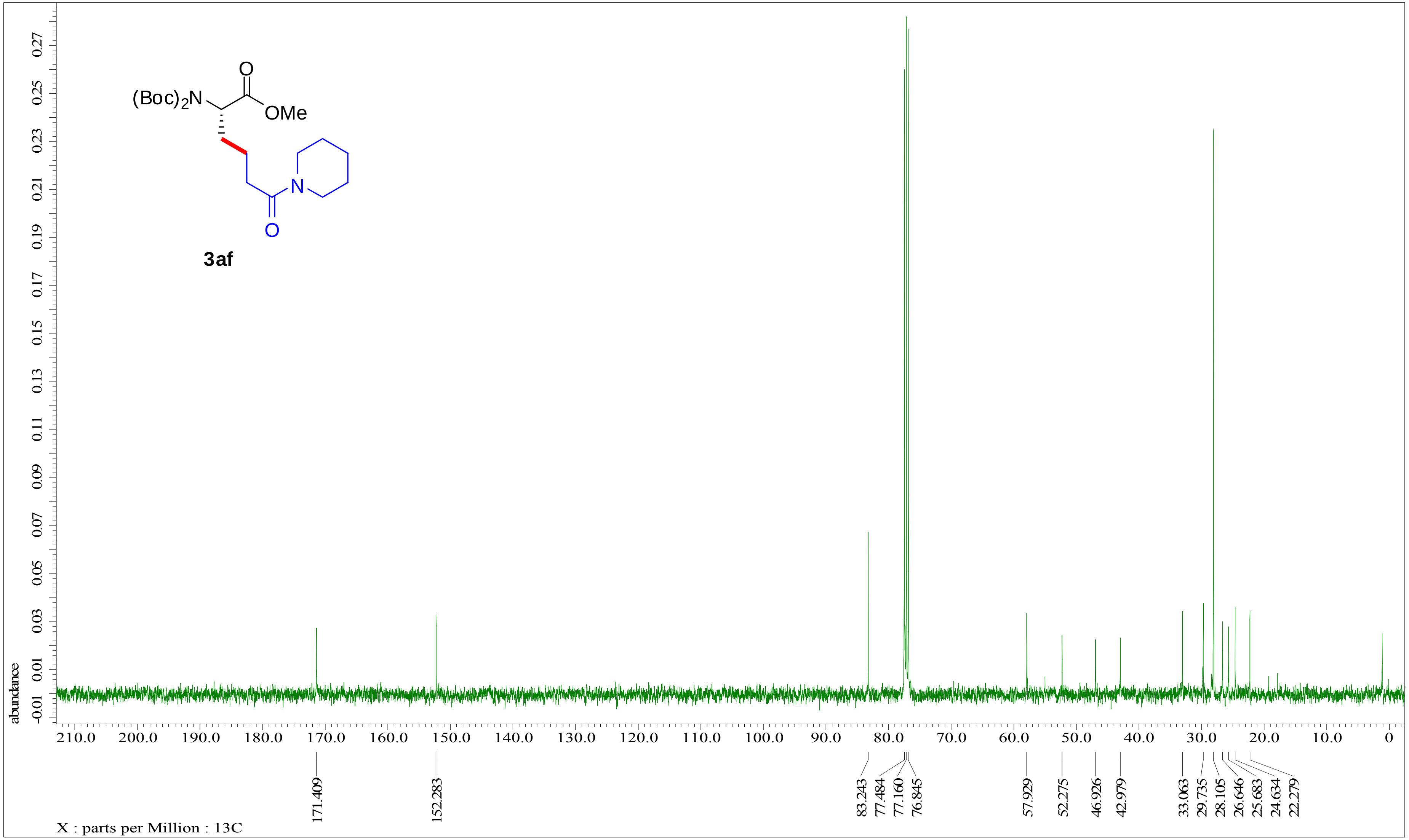
(Boc)2N
O
OMe
N
O
3af
abundance
0.27
0.25
0.23
0.21
0.19
0.17
0.15
0.13
0.11
0.09
0.07
0.05
0.03
0.01
-0.01
210.0 200.0 190.0 180.0 170.0 160.0 150.0 140.0 130.0 120.0 110.0 100.0 90.0 80.0 70.0 60.0 50.0 40.0 30.0 20.0 10.0 0
171.409
152.283
83.243
77.484
77.160
76.845
57.929
52.275
46.926
42.979
33.063
29.735
28.105
26.646
25.683
24.634
22.279
X : parts per Million : 13C

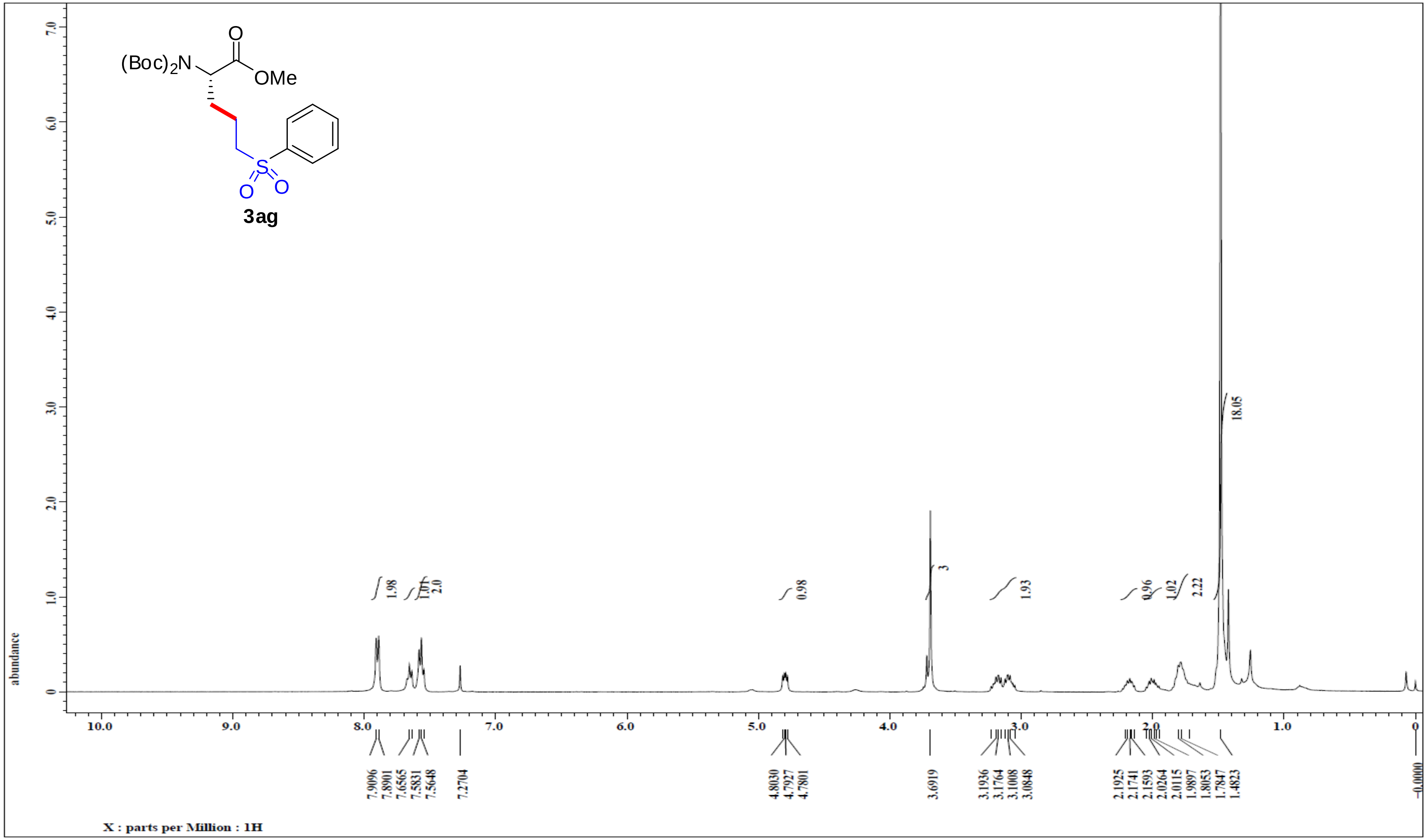
(Boc)2N
O
OMe
S
O
O
3ag
abundance
7.0
6.0
5.0
4.0
3.0
2.0
1.0
0
10.0
9.0
8.0
7.0
6.0
5.0
4.0
3.0
2.0
1.0
0
1.98
1.01
2.0
0.98
3
1.93
0.96
1.02
2.22
18.05
7.9096
7.8901
7.6565
7.5831
7.5648
7.2704
4.8030
4.7927
4.7801
3.6919
3.1936
3.1764
3.1008
3.0848
2.1925
2.1741
2.1593
2.0264
2.0115
1.9897
1.8053
1.7847
1.4823
-0.0000
X : parts per Million : 1H

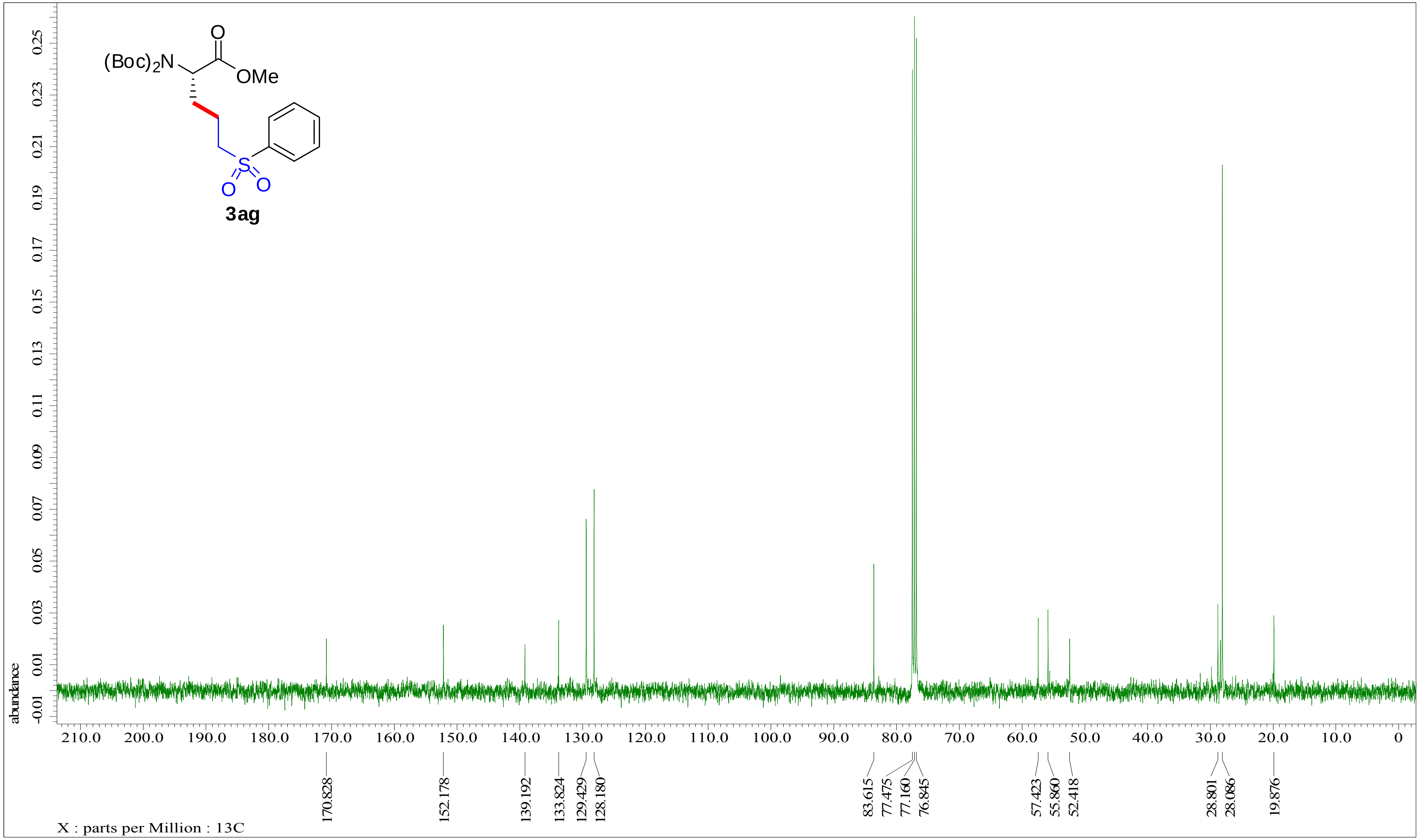

(Boc)$_2$N
O
OMe
S
O
O
3ag
abundance
0.25
0.23
0.21
0.19
0.17
0.15
0.13
0.11
0.09
0.07
0.05
0.03
0.01
-0.01
210.0 200.0 190.0 180.0 170.0 160.0 150.0 140.0 130.0 120.0 110.0 100.0 90.0 80.0 70.0 60.0 50.0 40.0 30.0 20.0 10.0 0
170.828
152.178
139.192
133.824
129.429
128.180
83.615
77.475
77.160
76.845
57.423
55.860
52.418
28.801
28.086
19.876
X : parts per Million : 13C

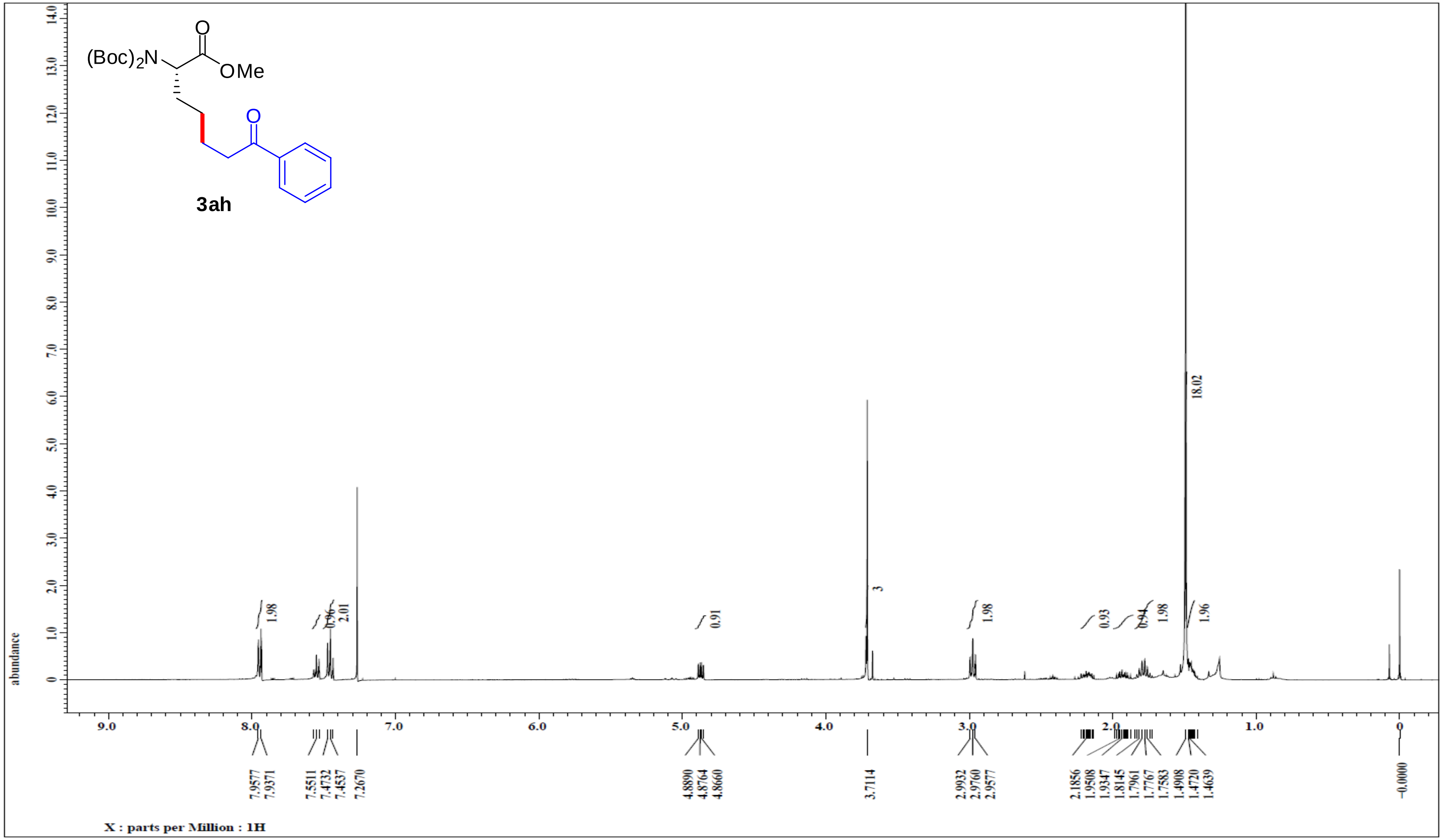
(Boc)2N
OMe
3ah
X : parts per Million : 1H
abundance

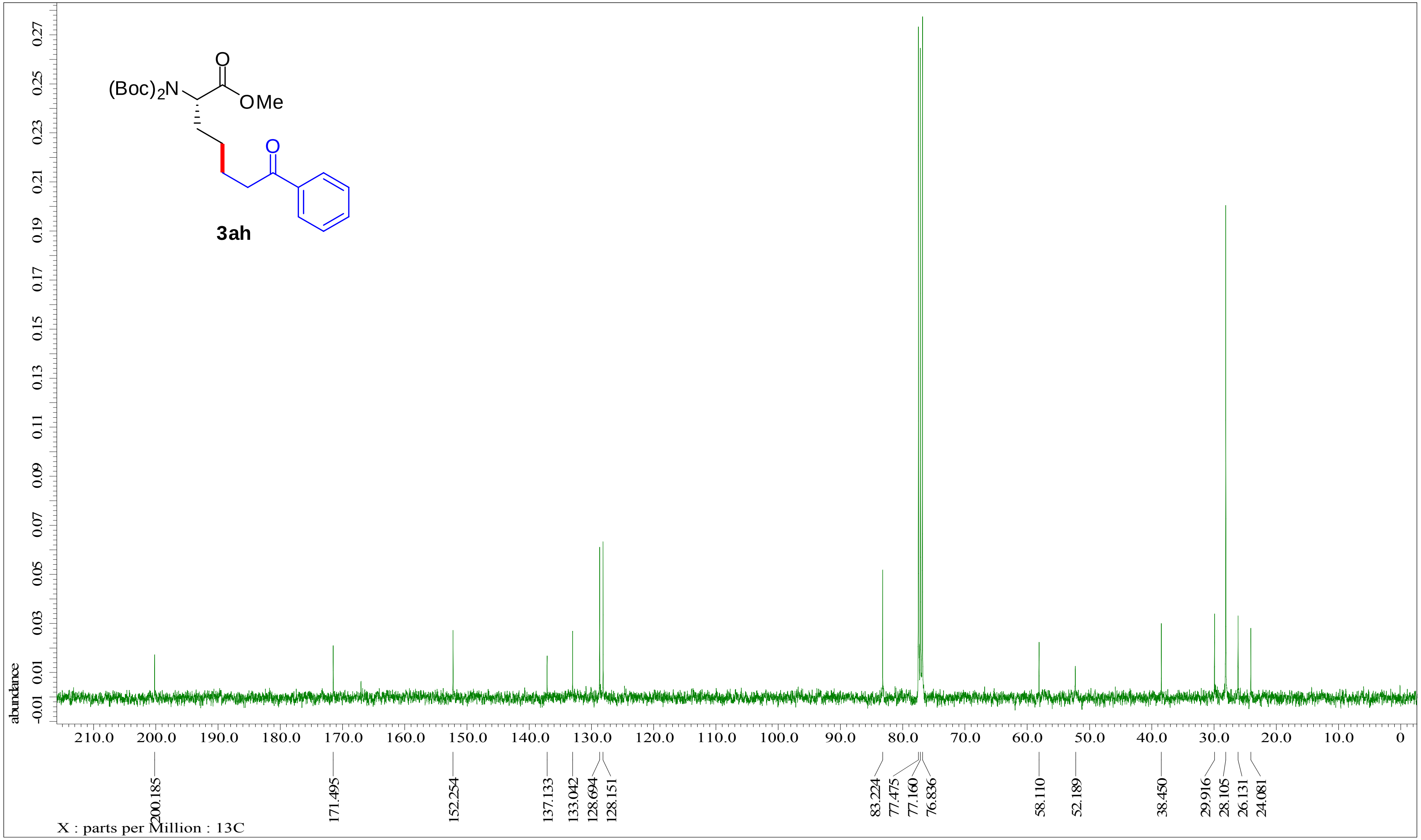

(Boc)2N
O
OMe
O
3ah
abundance
0.27
0.25
0.23
0.21
0.19
0.17
0.15
0.13
0.11
0.09
0.07
0.05
0.03
0.01
-0.01
210.0
200.0
190.0
180.0
170.0
160.0
150.0
140.0
130.0
120.0
110.0
100.0
90.0
80.0
70.0
60.0
50.0
40.0
30.0
20.0
10.0
0
200.185
171.495
152.254
137.133
133.042
128.694
128.151
83.224
77.475
77.160
76.836
58.110
52.189
38.450
29.916
28.105
26.131
24.081
X : parts per Million : 13C

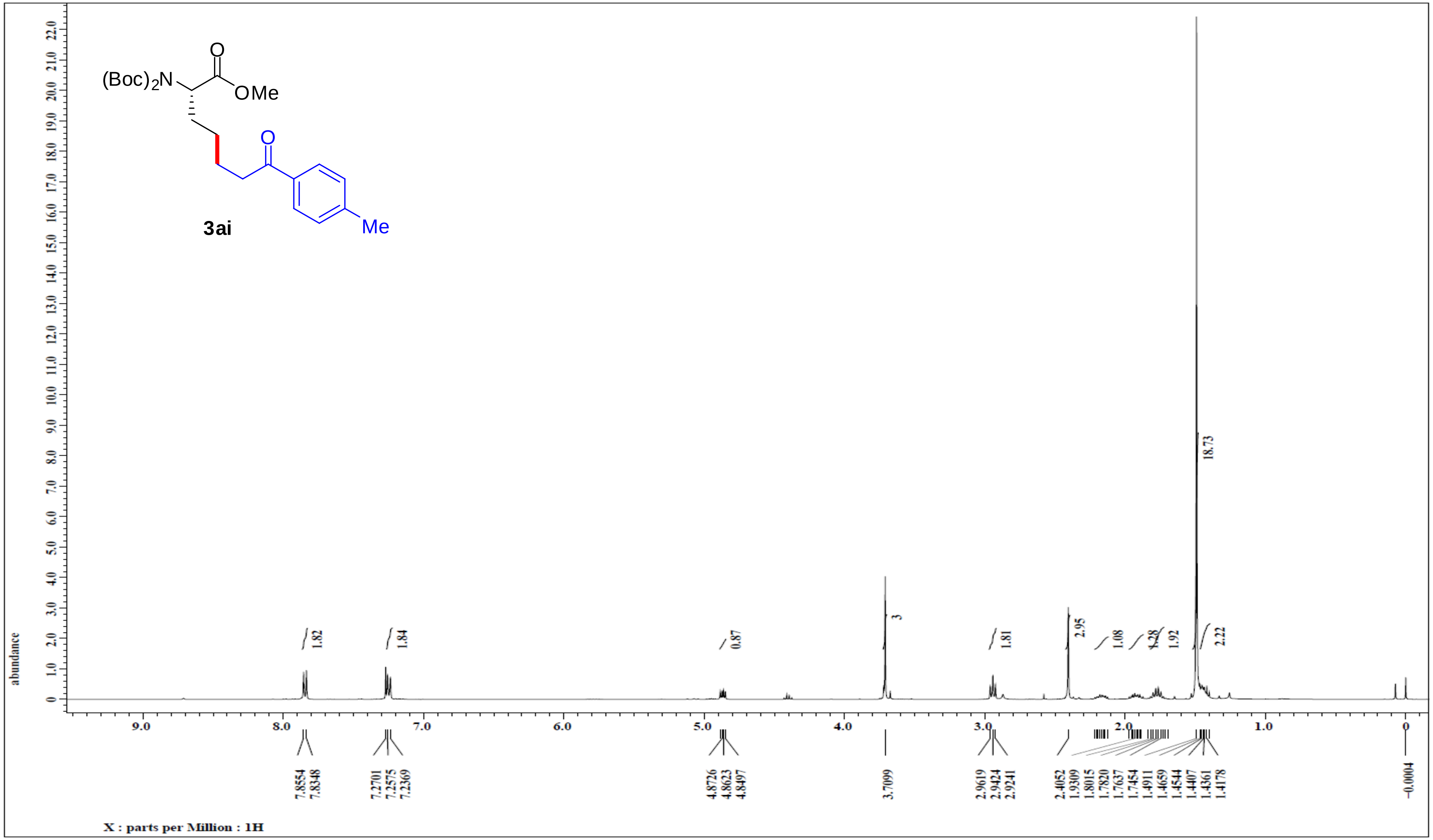

(Boc)2N
O
OMe
O
Me
3ai
abundance
X : parts per Million : 1H

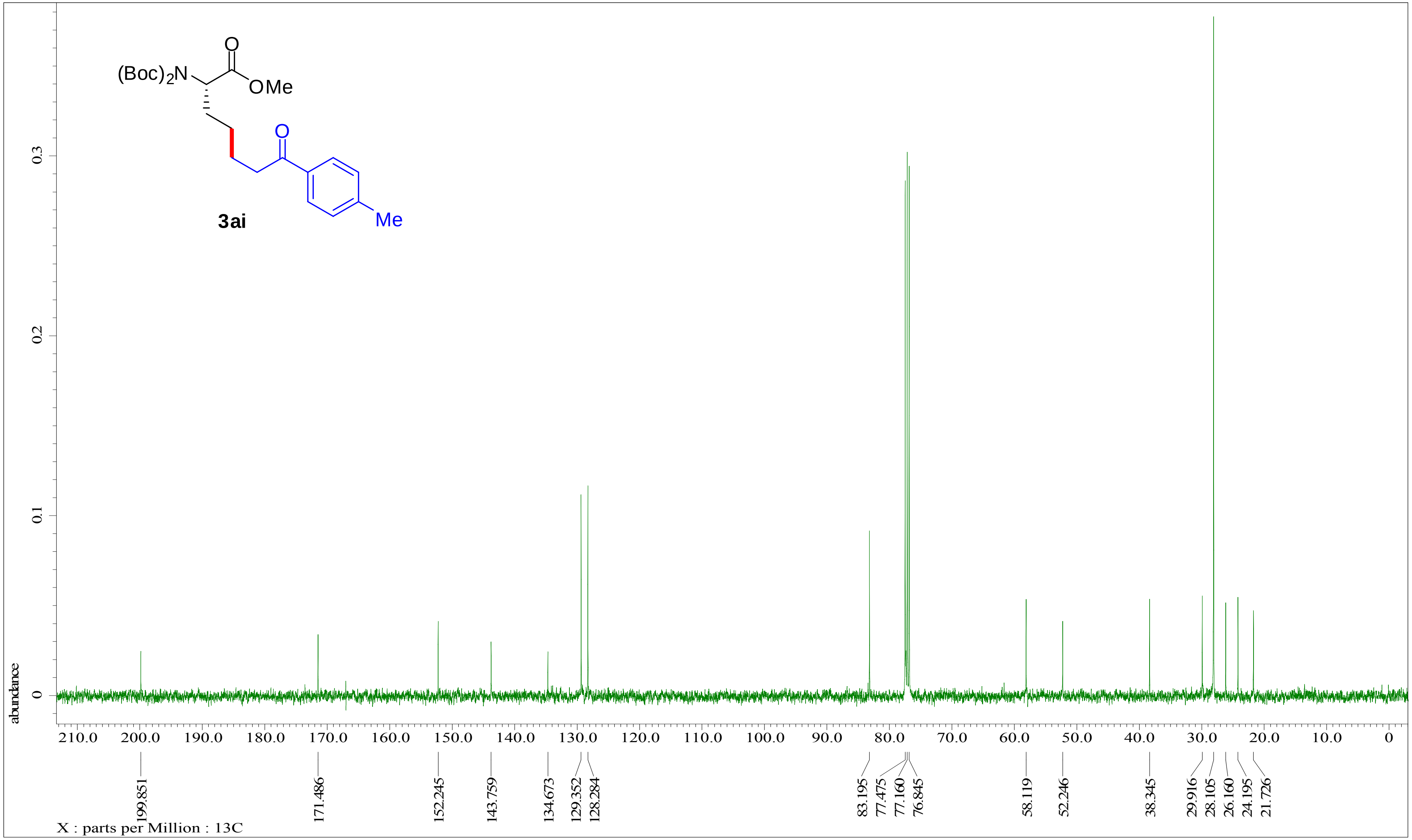

(Boc)2N
O
OMe
O
Me
3ai
abundance
0.3
0.2
0.1
0
210.0
200.0
190.0
180.0
170.0
160.0
150.0
140.0
130.0
120.0
110.0
100.0
90.0
80.0
70.0
60.0
50.0
40.0
30.0
20.0
10.0
0
199.851
171.486
152.245
143.759
134.673
129.352
128.284
83.195
77.475
77.160
76.845
58.119
52.246
38.345
29.916
28.105
26.160
24.195
21.726
X : parts per Million : 13C

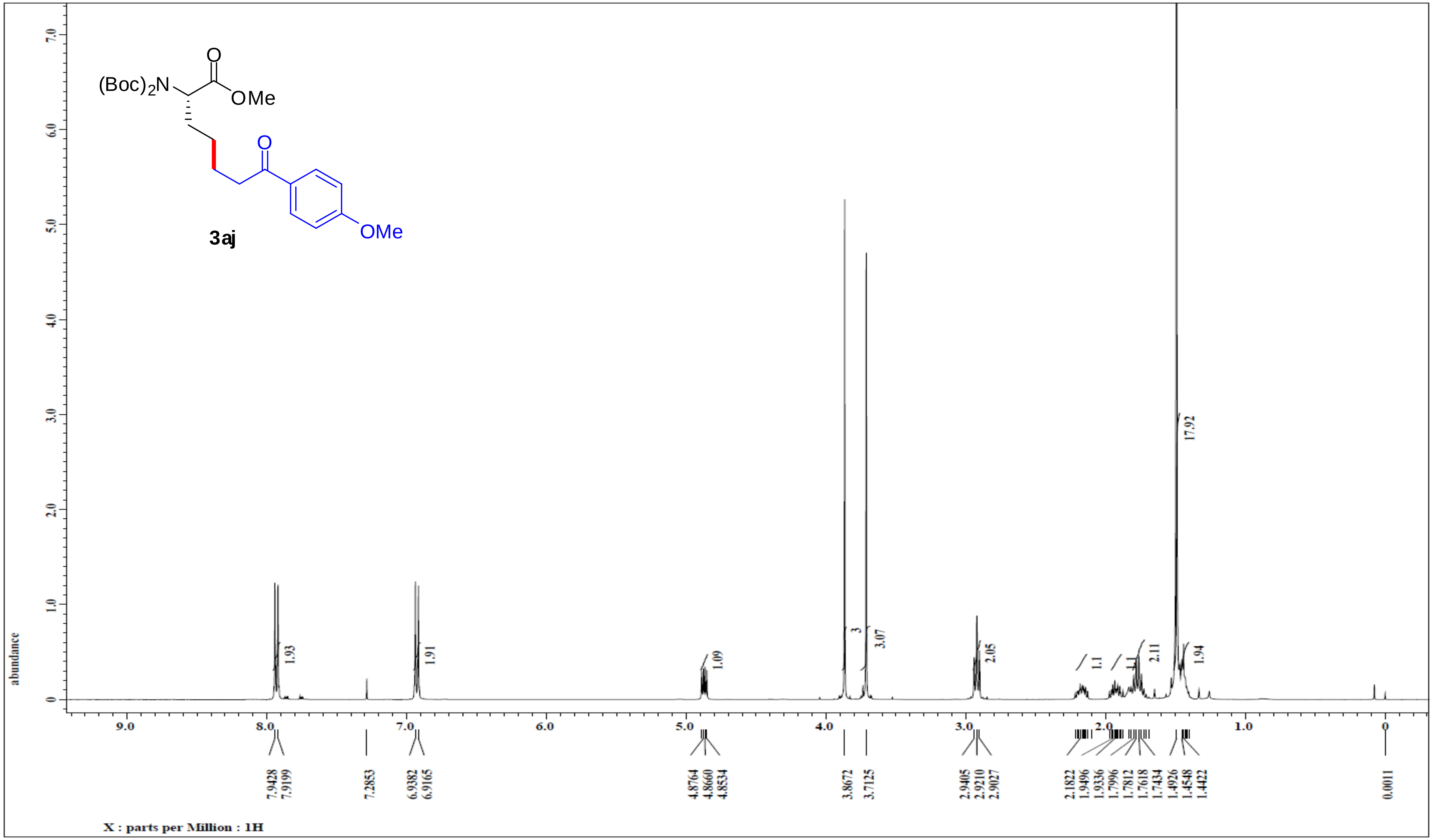
(Boc)2N
OMe
O
OMe
3aj
abundance
X : parts per Million : 1H
7.9428
7.9199
7.2853
6.9382
6.9165
4.8764
4.8660
4.8534
3.8672
3.7125
2.9405
2.9210
2.9027
2.1822
1.9496
1.9336
1.7996
1.7812
1.7618
1.7434
1.4926
1.4548
1.4422
0.0011
1.93
1.91
1.09
3
3.07
2.05
1.1
1.1
2.11
17.92
1.94

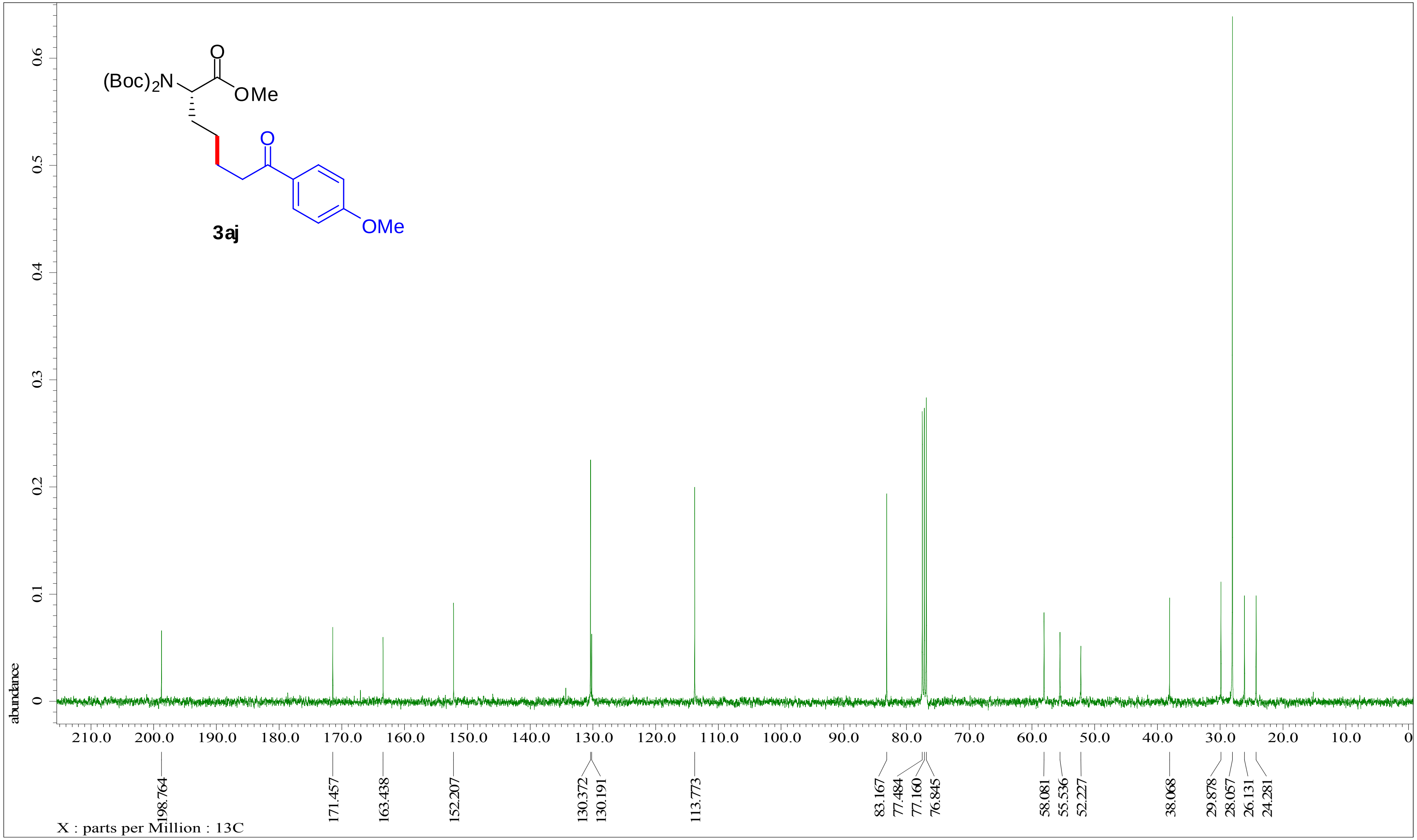

(Boc)$_2$N
O
OMe
O
OMe
3aj
abundance
0.6
0.5
0.4
0.3
0.2
0.1
0
210.0
200.0
190.0
180.0
170.0
160.0
150.0
140.0
130.0
120.0
110.0
100.0
90.0
80.0
70.0
60.0
50.0
40.0
30.0
20.0
10.0
0
198.764
171.457
163.438
152.207
130.372
130.191
113.773
83.167
77.484
77.160
76.845
58.081
55.536
52.227
38.068
29.878
28.057
26.131
24.281
X : parts per Million : 13C

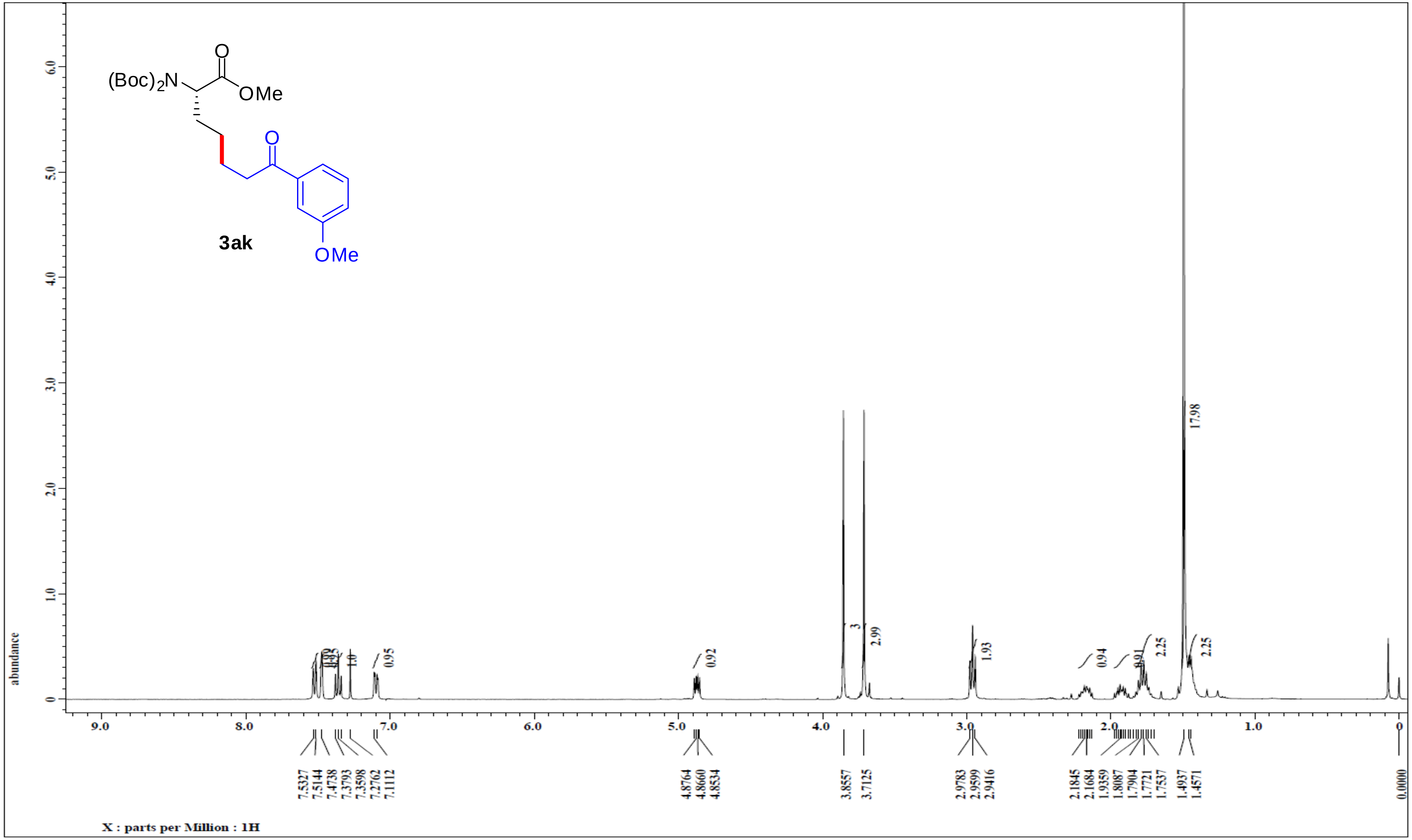
(Boc)2N
OMe
O
OMe
3ak
abundance
X : parts per Million : 1H
17.98
3
2.99
1.93
0.94
0.91
2.25
2.25
0.92
0.95
1.0
7.5327
7.5144
7.4738
7.3793
7.3598
7.2762
7.1112
4.8764
4.8660
4.8534
3.8557
3.7125
2.9783
2.9599
2.9416
2.1845
2.1684
1.9359
1.8087
1.7904
1.7721
1.7537
1.4937
1.4571
0.0000

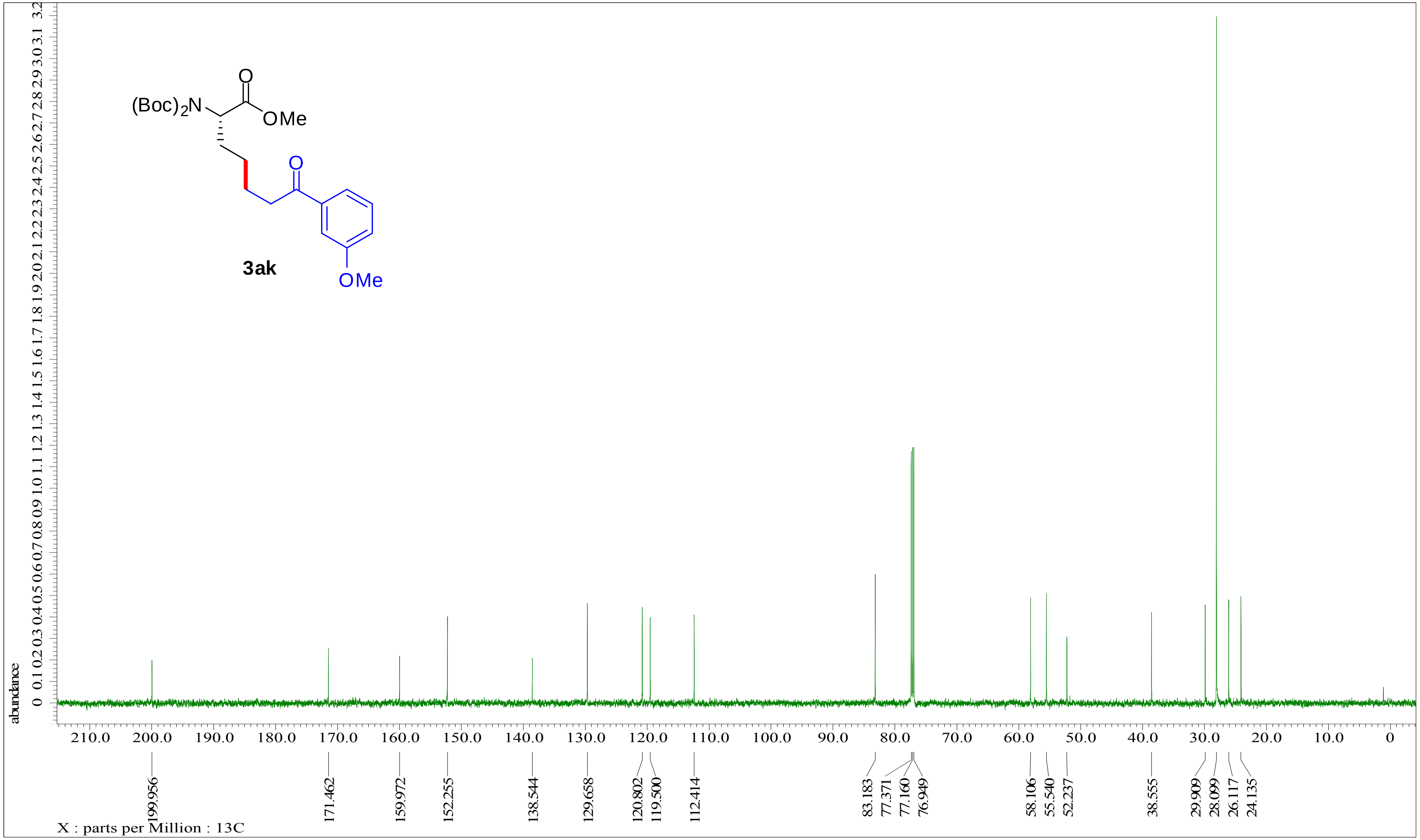
(Boc)2N
O
OMe
O
OMe
3ak
abundance
0 0.1 0.2 0.3 0.4 0.5 0.6 0.7 0.8 0.9 1.0 1.1 1.2 1.3 1.4 1.5 1.6 1.7 1.8 1.9 2.0 2.1 2.2 2.3 2.4 2.5 2.6 2.7 2.8 2.9 3.0 3.1 3.2
210.0 200.0 190.0 180.0 170.0 160.0 150.0 140.0 130.0 120.0 110.0 100.0 90.0 80.0 70.0 60.0 50.0 40.0 30.0 20.0 10.0 0
199.956
171.462
159.972
152.255
138.544
129.658
120.802
119.500
112.414
83.183
77.371
77.160
76.949
58.106
55.540
52.237
38.555
29.909
28.099
26.117
24.135
X : parts per Million : 13C

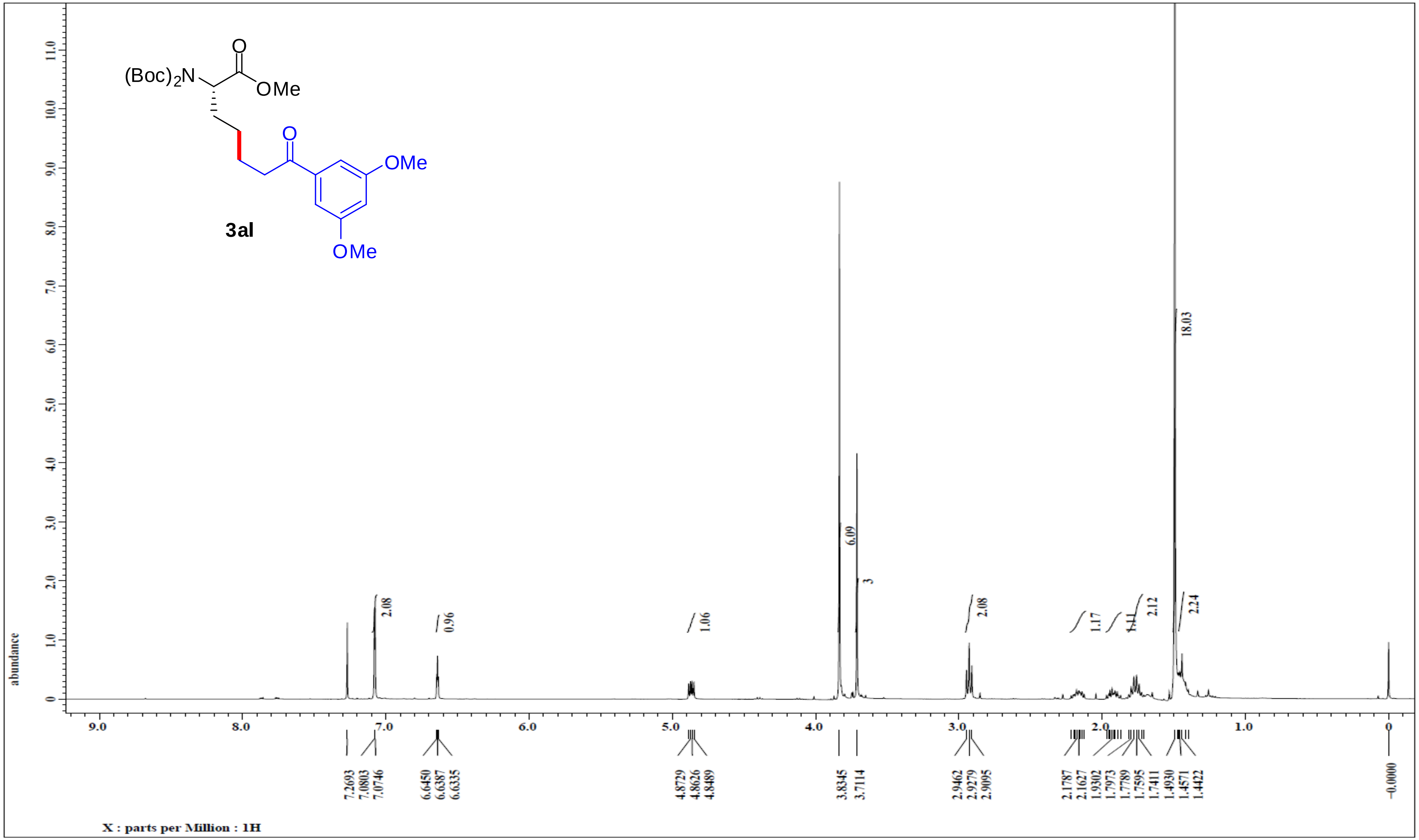
(Boc)2N
OMe
O
OMe
OMe
3al
abundance
X : parts per Million : 1H
2.08
0.96
1.06
6.09
3
2.08
1.17
1.11
2.12
2.24
18.03
7.2693
7.0803
7.0746
6.6450
6.6387
6.6335
4.8729
4.8626
4.8489
3.8345
3.7114
2.9462
2.9279
2.9095
2.1787
2.1627
1.9302
1.7973
1.7789
1.7595
1.7411
1.4930
1.4571
1.4422
-0.0000

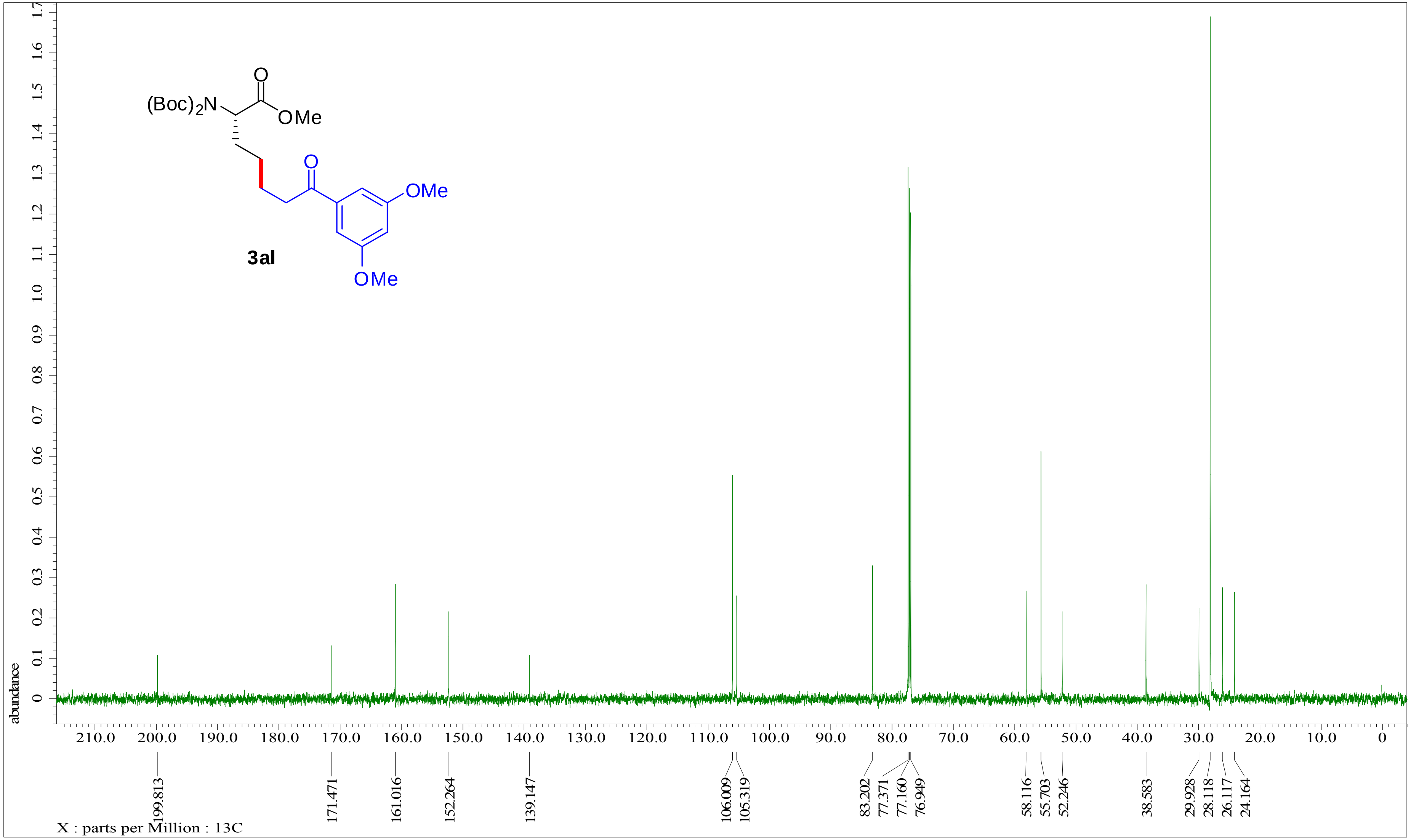

(Boc)2N
O
OMe
O
OMe
OMe
3al
abundance
0
0.1
0.2
0.3
0.4
0.5
0.6
0.7
0.8
0.9
1.0
1.1
1.2
1.3
1.4
1.5
1.6
1.7
210.0
200.0
190.0
180.0
170.0
160.0
150.0
140.0
130.0
120.0
110.0
100.0
90.0
80.0
70.0
60.0
50.0
40.0
30.0
20.0
10.0
0
199.813
171.471
161.016
152.264
139.147
106.009
105.319
83.202
77.371
77.160
76.949
58.116
55.703
52.246
38.583
29.928
28.118
26.117
24.164
X : parts per Million : 13C

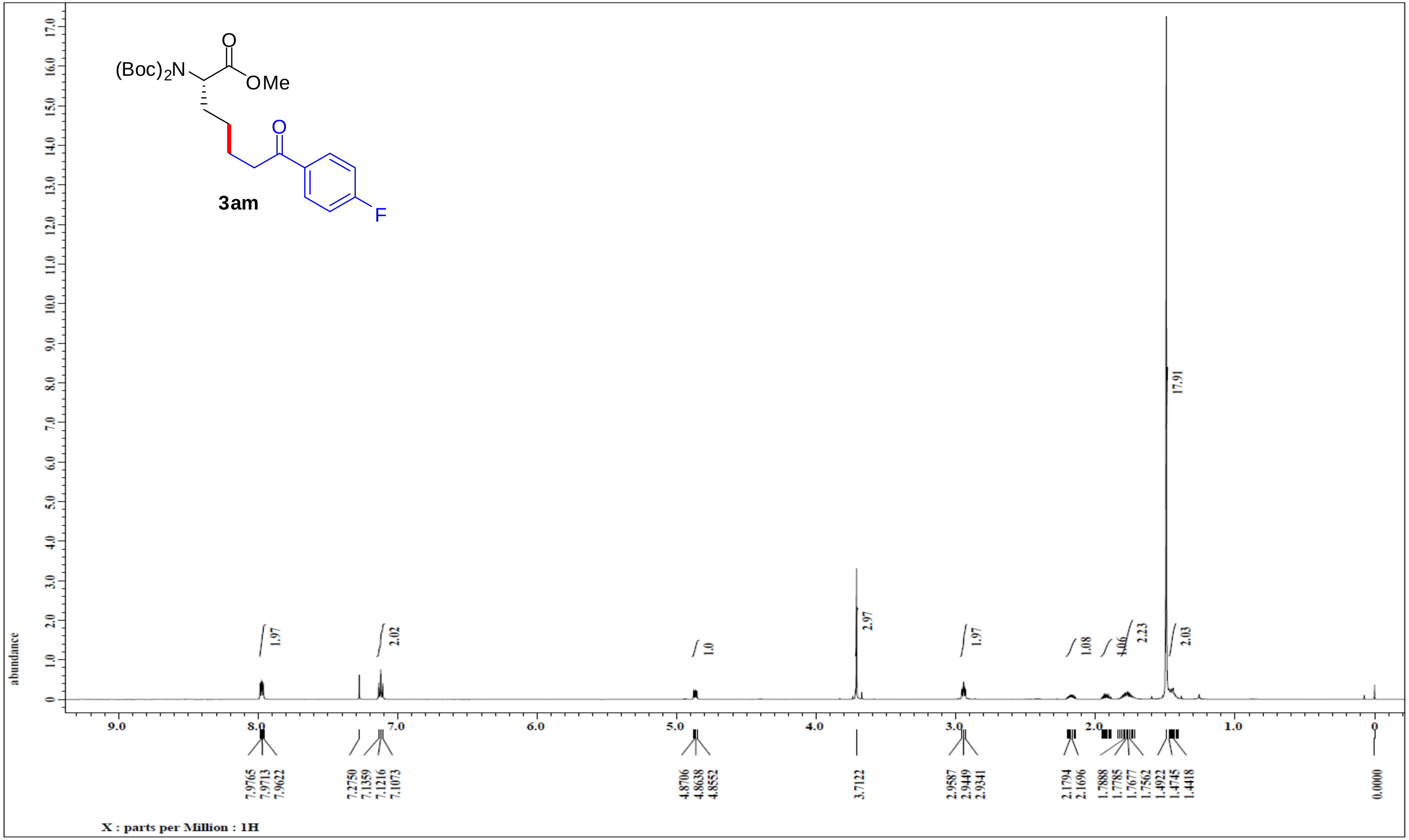
(Boc)2N
OMe
O
F
3am
abundance
X : parts per Million : 1H
17.91
2.97
1.97
2.02
1.0
1.97
1.08
1.06
2.23
2.03
7.9765
7.9713
7.9622
7.2750
7.1359
7.1216
7.1073
4.8706
4.8638
4.8552
3.7122
2.9587
2.9449
2.9341
2.1794
2.1696
1.7888
1.7785
1.7677
1.7562
1.4922
1.4745
1.4418
0.0000

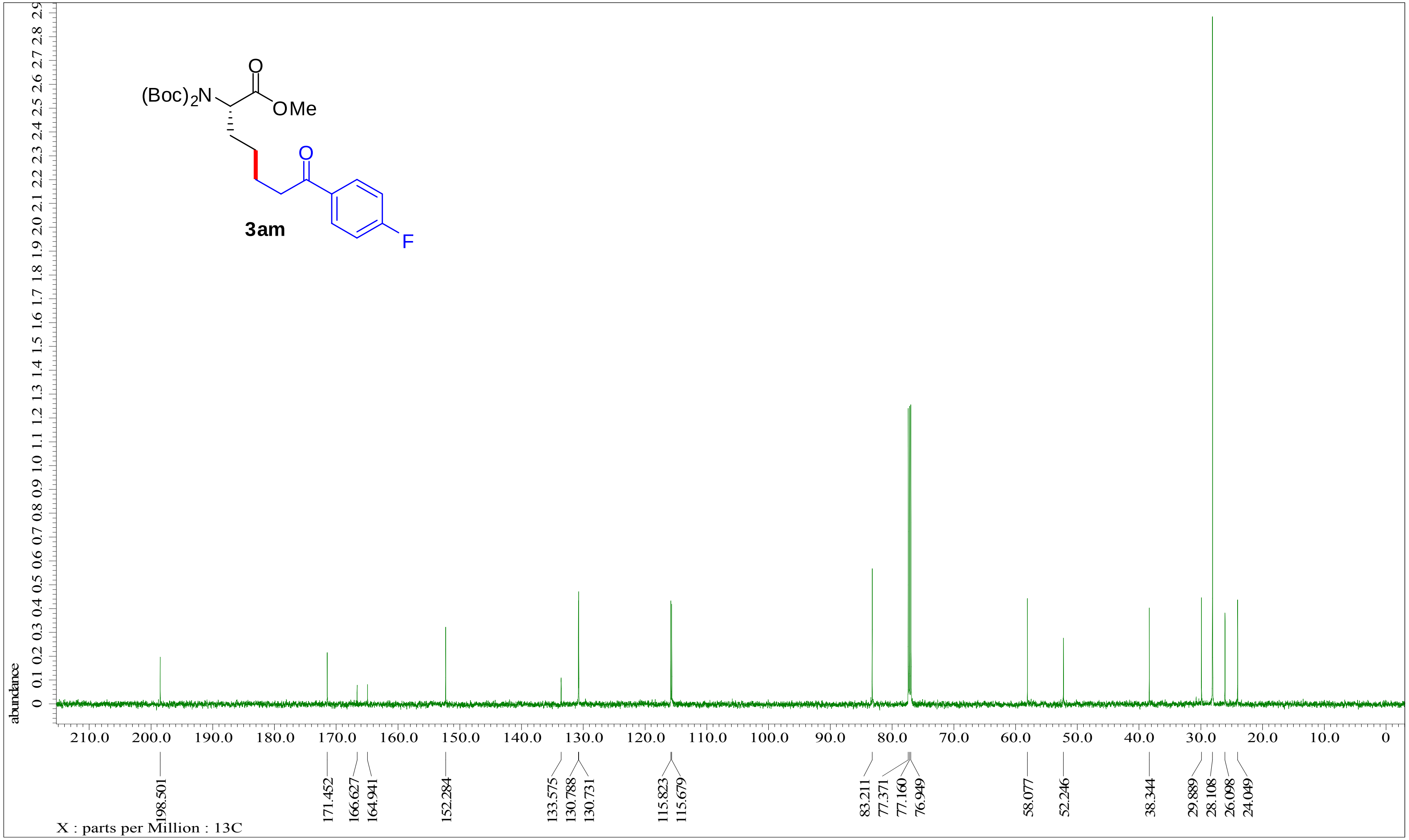
(Boc)2N
O
OMe
O
F
3am
abundance
X : parts per Million : 13C
198.501
171.452
166.627
164.941
152.284
133.575
130.788
130.731
115.823
115.679
83.211
77.371
77.160
76.949
58.077
52.246
38.344
29.889
28.108
26.098
24.049

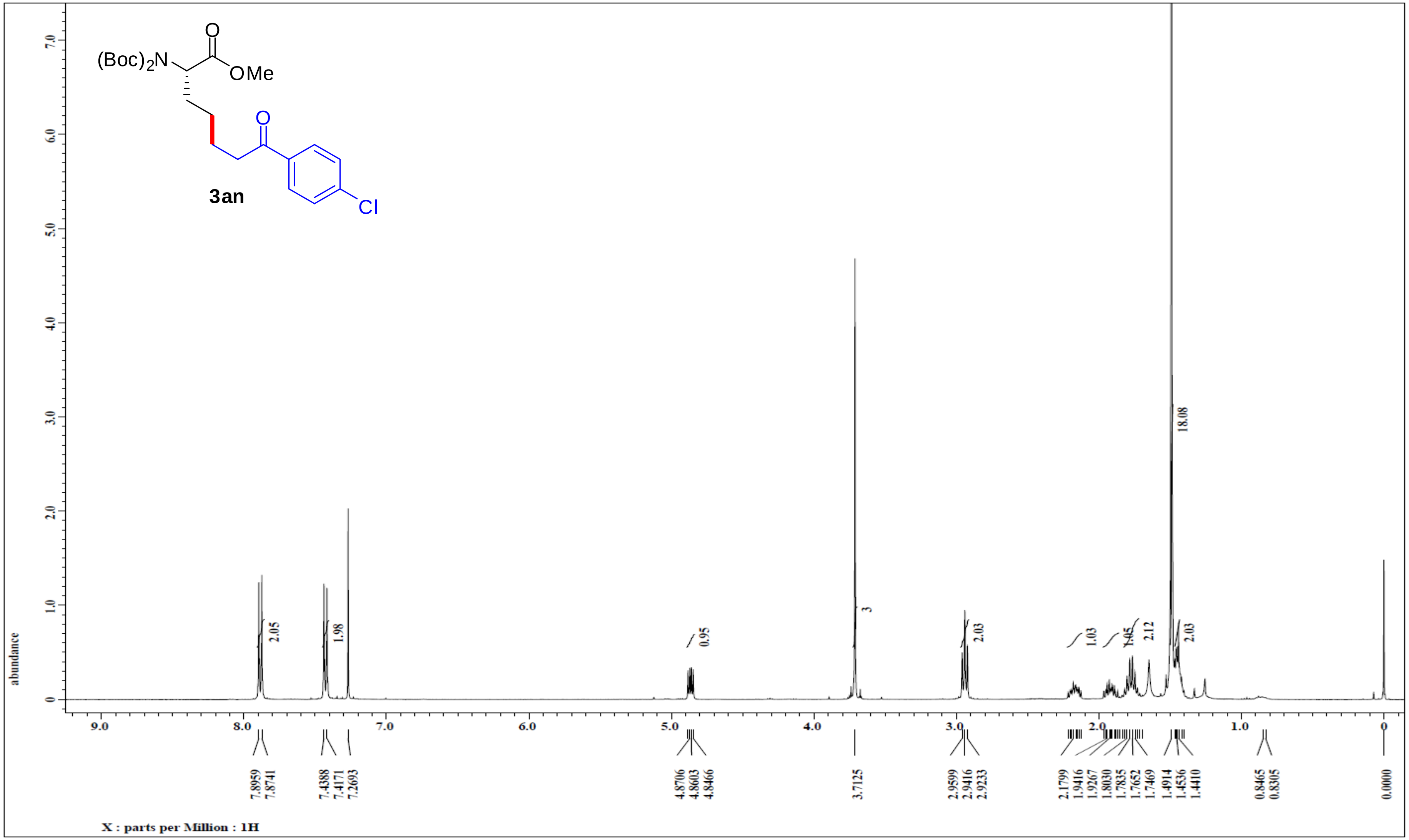
(Boc)2N
OMe
O
O
Cl
3an
abundance
X : parts per Million : 1H
9.0
8.0
7.0
6.0
5.0
4.0
3.0
2.0
1.0
0
2.05
1.98
0.95
3
2.03
1.03
1.05
2.12
2.03
18.08
7.8959
7.8741
7.4388
7.4171
7.2693
4.8706
4.8603
4.8466
3.7125
2.9599
2.9416
2.9233
2.1799
1.9416
1.9267
1.8030
1.7835
1.7652
1.7469
1.4914
1.4536
1.4410
0.8465
0.8305
0.0000

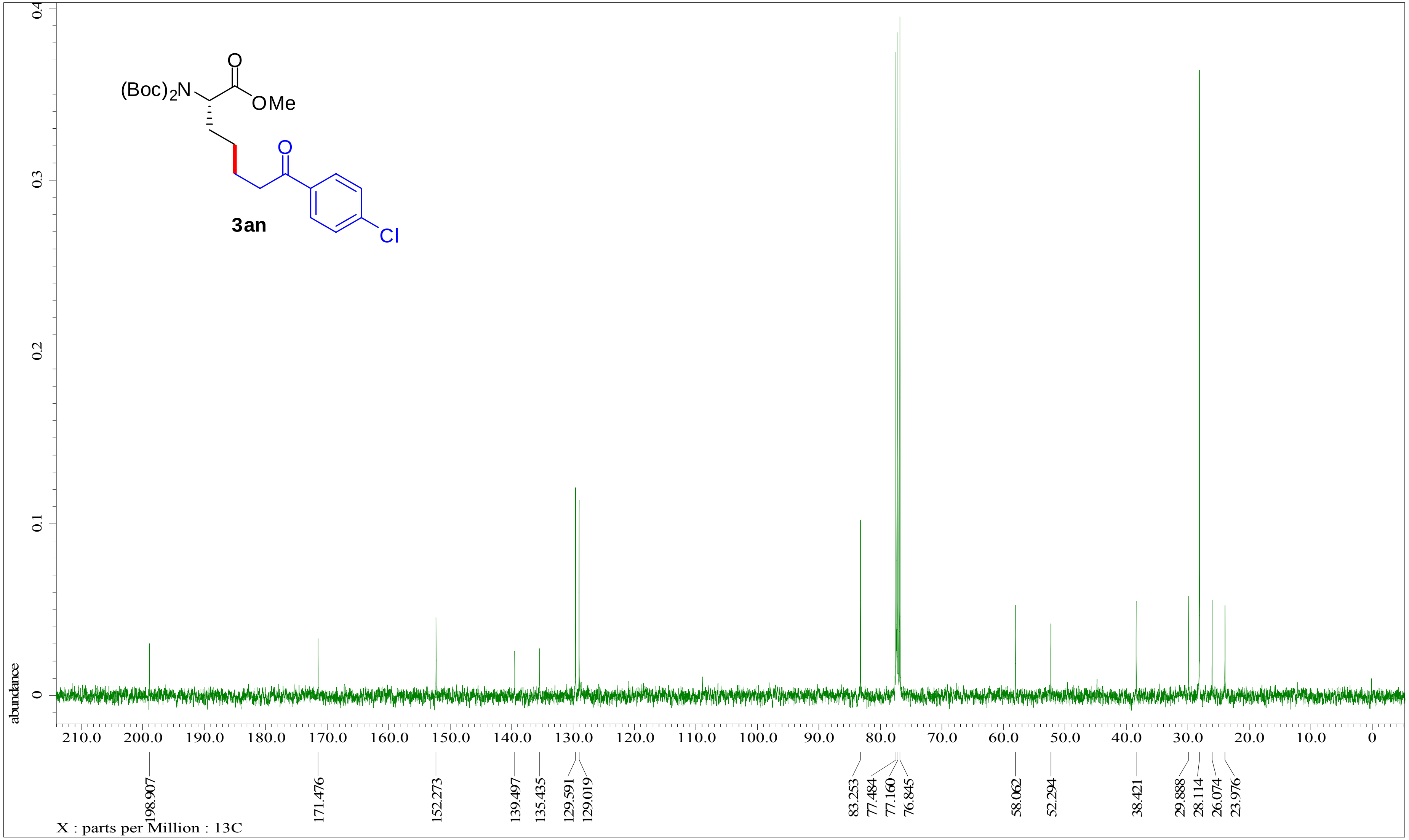

(Boc)2N
OMe
O
Cl
3an
abundance
0
0.1
0.2
0.3
0.4
210.0
200.0
190.0
180.0
170.0
160.0
150.0
140.0
130.0
120.0
110.0
100.0
90.0
80.0
70.0
60.0
50.0
40.0
30.0
20.0
10.0
0
198.907
171.476
152.273
139.497
135.435
129.591
129.019
83.253
77.484
77.160
76.845
58.062
52.294
38.421
29.888
28.114
26.074
23.976
X : parts per Million : 13C

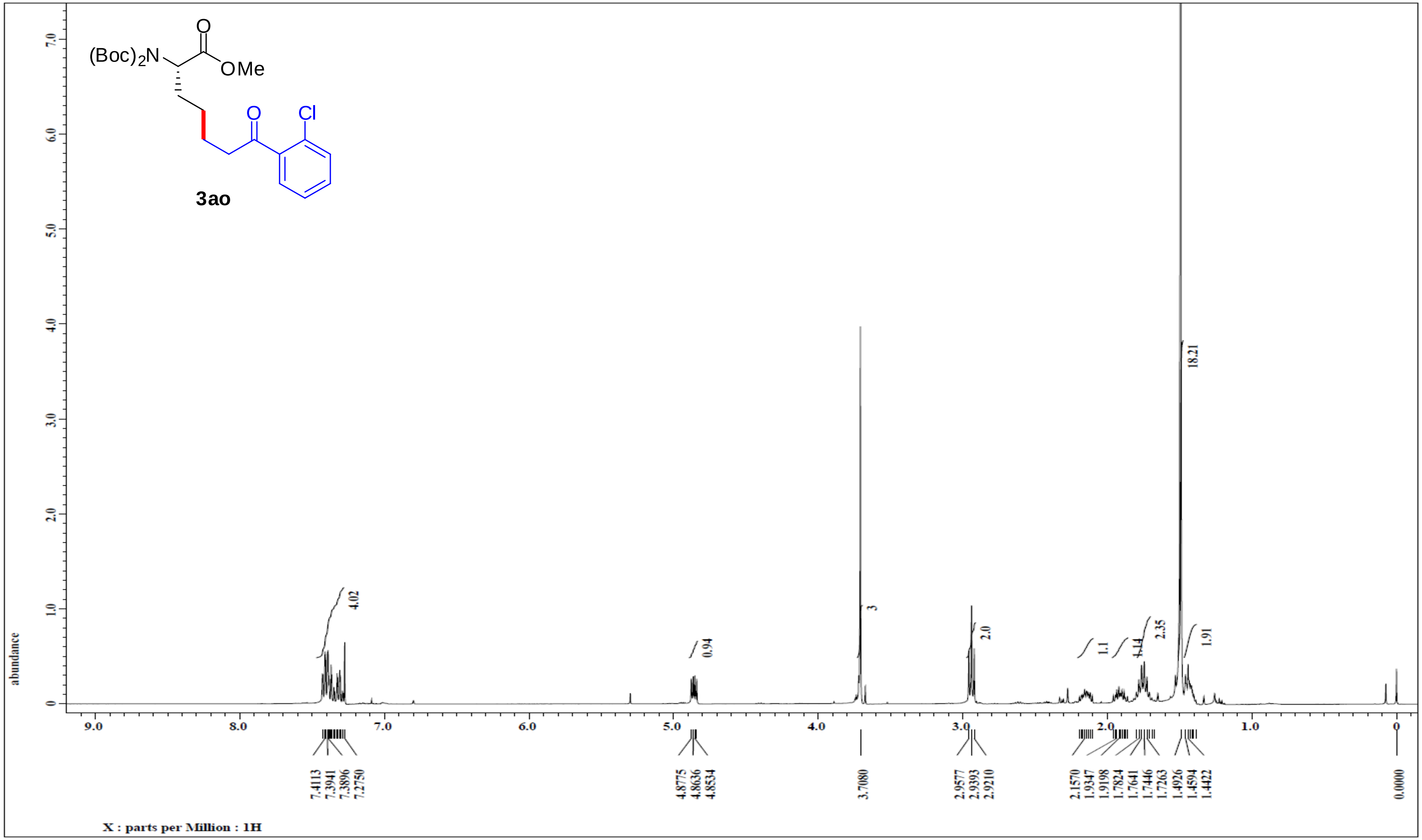

(Boc)2N
O
OMe
O
Cl
3ao
abundance
X : parts per Million : 1H
4.02
0.94
3
2.0
1.1
1.14
2.35
18.21
1.91
7.4113
7.3941
7.3896
7.2750
4.8775
4.8636
4.8534
3.7080
2.9577
2.9393
2.9210
2.1570
1.9347
1.9198
1.7824
1.7641
1.7446
1.7263
1.4926
1.4594
1.4422
0.0000

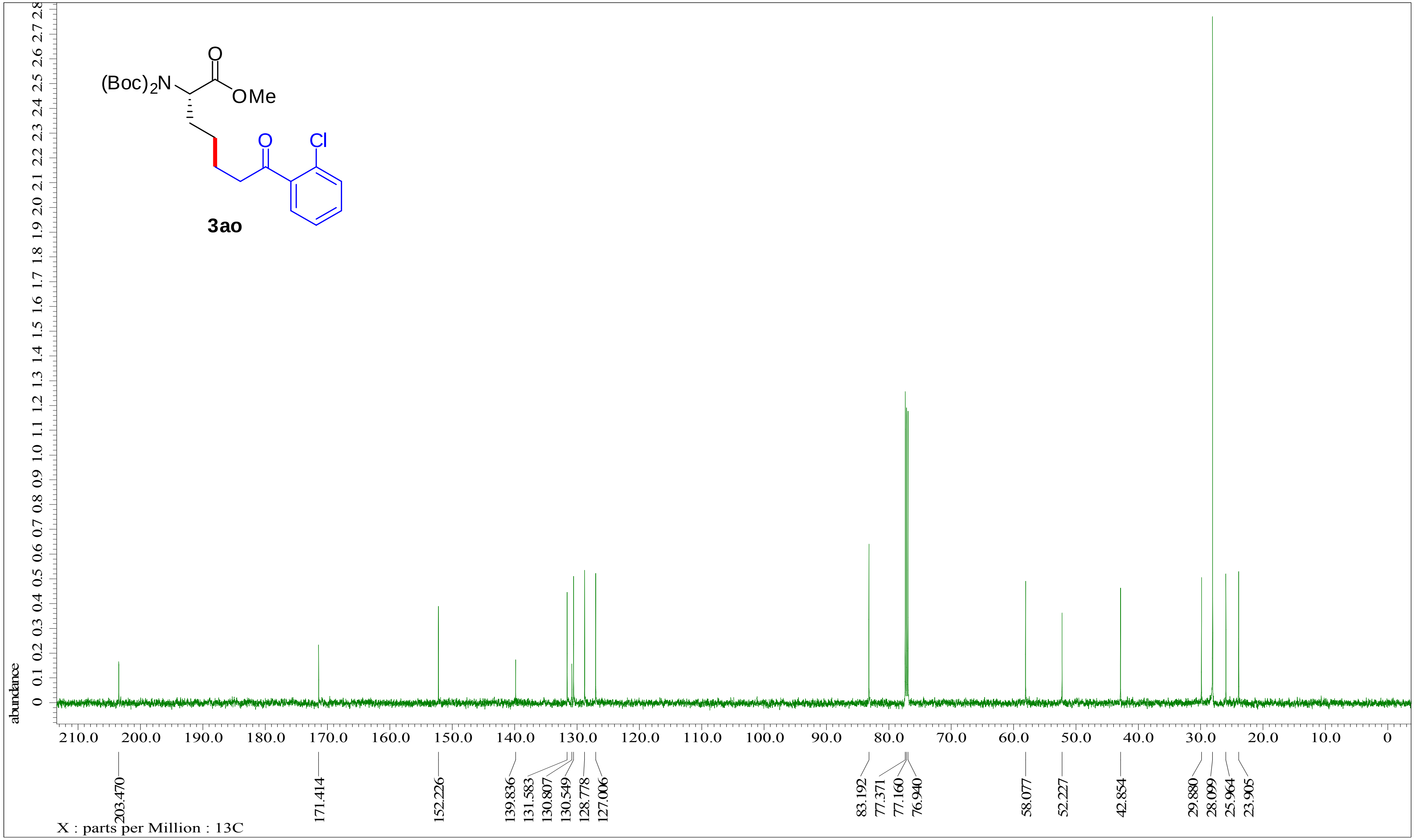

(Boc)2N
O
OMe
O
Cl
3ao
abundance
X : parts per Million : 13C
203.470
171.414
152.226
139.836
131.583
130.807
130.549
128.778
127.006
83.192
77.371
77.160
76.940
58.077
52.227
42.854
29.880
28.099
25.964
23.905

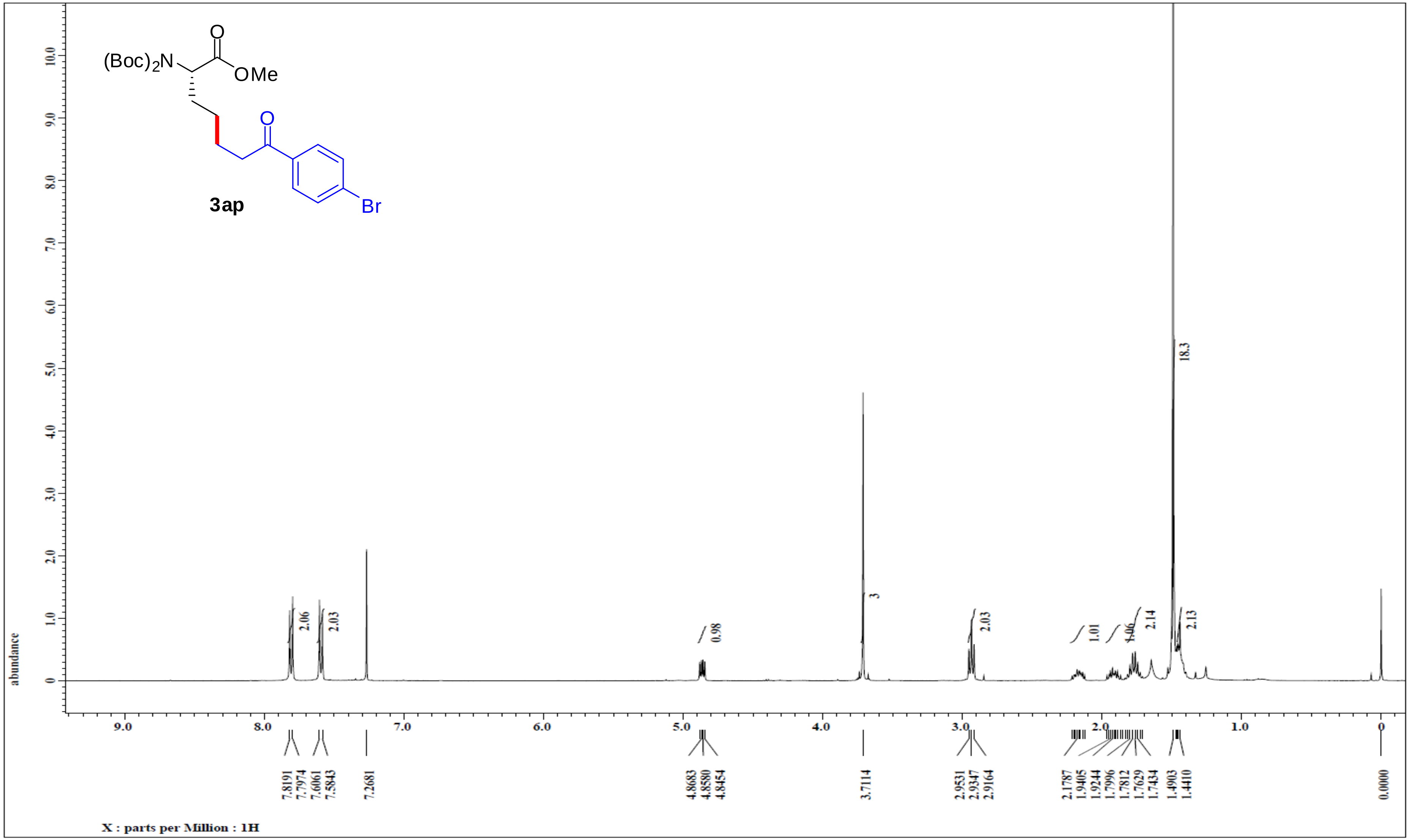

(Boc)2N
OMe
O
O
Br
3ap
abundance
X : parts per Million : 1H
2.06
2.03
0.98
3
2.03
1.01
1.06
2.14
18.3
2.13
7.8191
7.7974
7.6061
7.5843
7.2681
4.8683
4.8580
4.8454
3.7114
2.9531
2.9347
2.9164
2.1787
1.9405
1.9244
1.7996
1.7812
1.7629
1.7434
1.4903
1.4410
0.0000

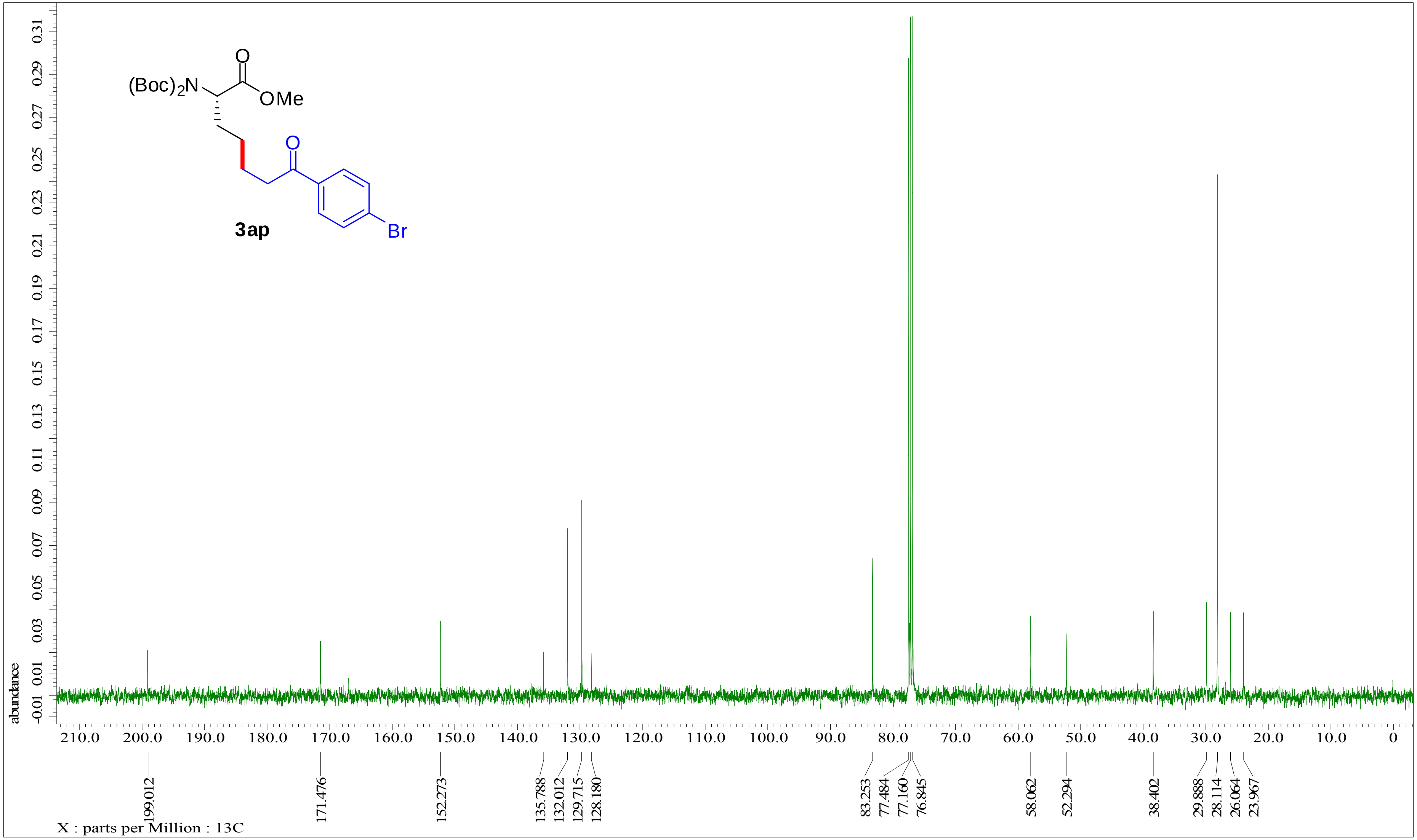
(Boc)$_2$N
O
OMe
O
Br
3ap
abundance
-0.01 0.01 0.03 0.05 0.07 0.09 0.11 0.13 0.15 0.17 0.19 0.21 0.23 0.25 0.27 0.29 0.31
210.0 200.0 190.0 180.0 170.0 160.0 150.0 140.0 130.0 120.0 110.0 100.0 90.0 80.0 70.0 60.0 50.0 40.0 30.0 20.0 10.0 0
199.012
171.476
152.273
135.788
132.012
129.715
128.180
83.253
77.484
77.160
76.845
58.062
52.294
38.402
29.888
28.114
26.064
23.967
X : parts per Million : 13C

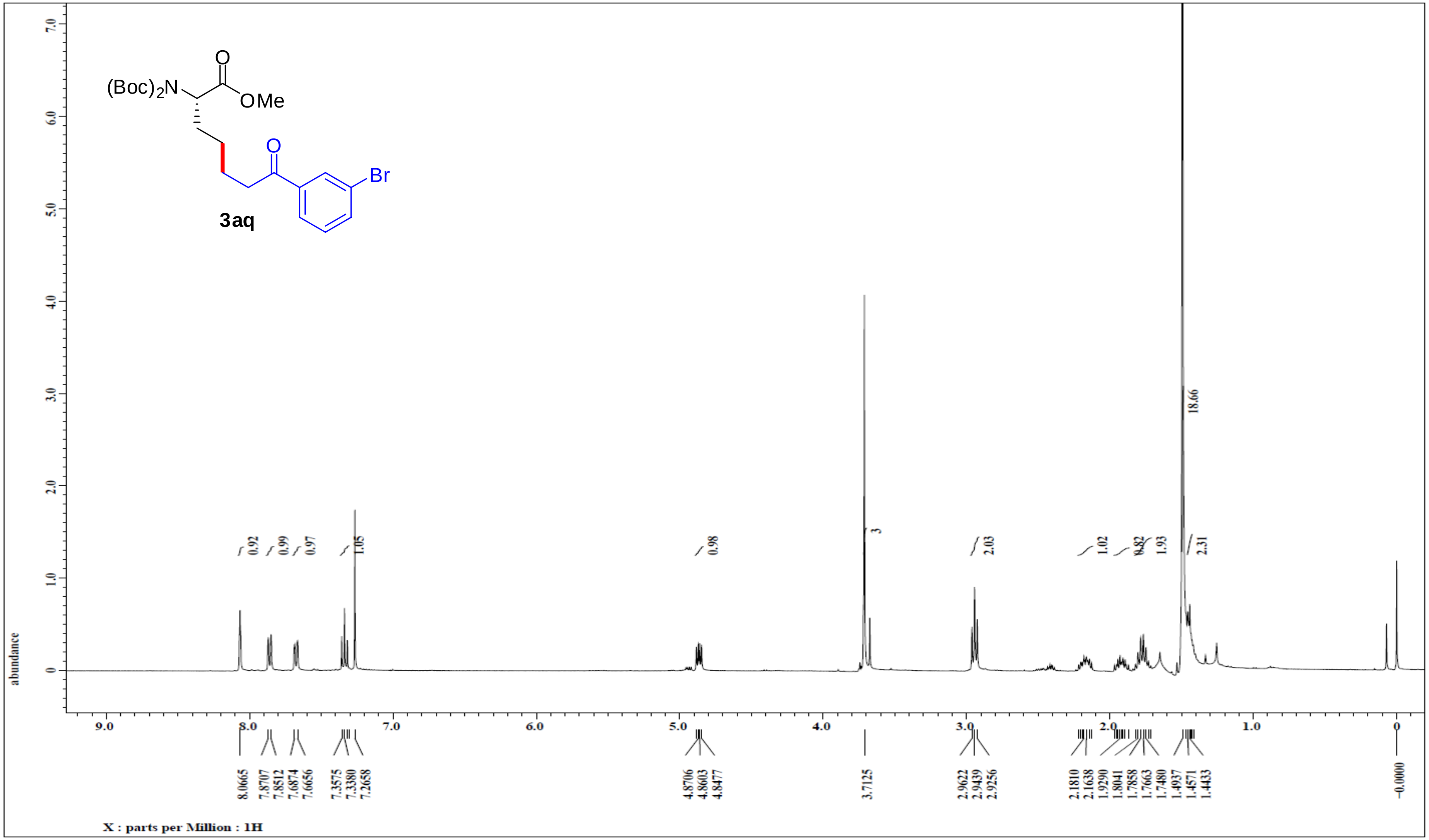
(Boc)2N
OMe
Br
3aq
abundance
X : parts per Million : 1H
0.92
0.99
0.97
1.05
0.98
3
2.03
1.02
0.82
1.93
18.66
2.31
8.0665
7.8707
7.8512
7.6874
7.6656
7.3575
7.3380
7.2658
4.8706
4.8603
4.8477
3.7125
2.9622
2.9439
2.9256
2.1810
2.1638
1.9290
1.8041
1.7858
1.7663
1.7480
1.4937
1.4571
1.4433
-0.0000

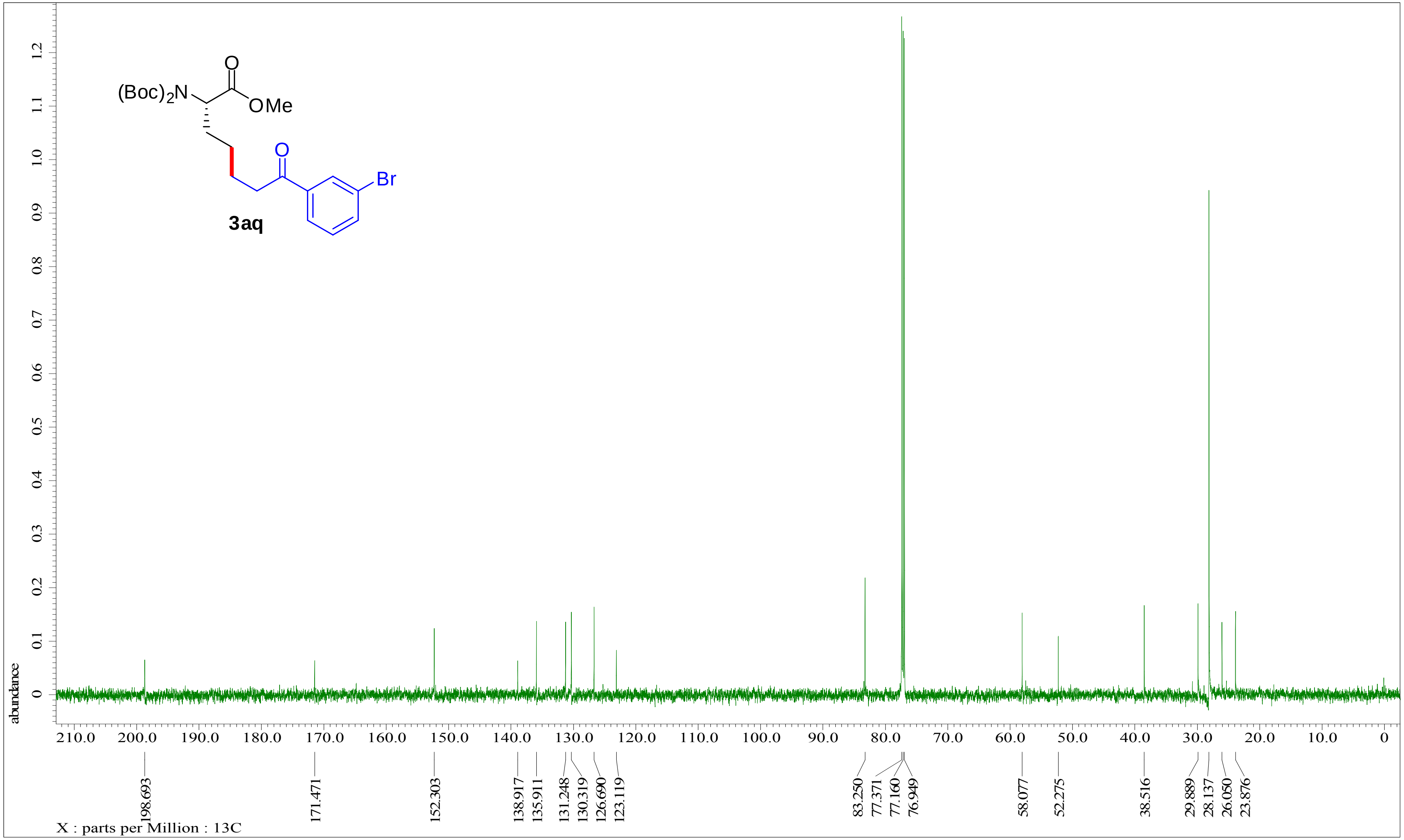

(Boc)2N
O
OMe
O
Br
3aq
abundance
0
0.1
0.2
0.3
0.4
0.5
0.6
0.7
0.8
0.9
1.0
1.1
1.2
210.0
200.0
190.0
180.0
170.0
160.0
150.0
140.0
130.0
120.0
110.0
100.0
90.0
80.0
70.0
60.0
50.0
40.0
30.0
20.0
10.0
0
198.693
171.471
152.303
138.917
135.911
131.248
130.319
126.690
123.119
83.250
77.371
77.160
76.949
58.077
52.275
38.516
29.889
28.137
26.050
23.876
X : parts per Million : 13C

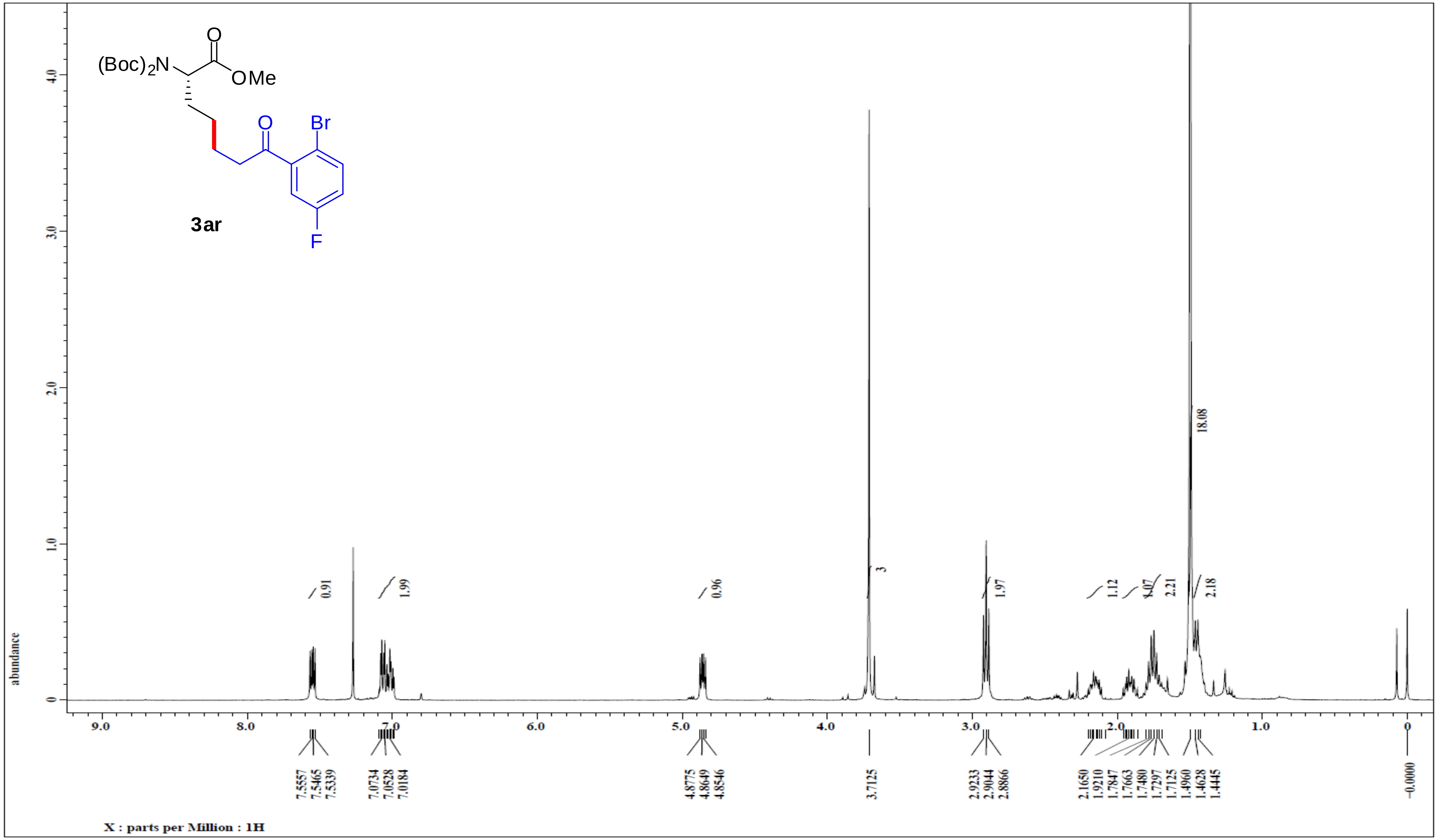
(Boc)2N
OMe
O
Br
F
3ar
abundance
X : parts per Million : 1H
0.91
1.99
0.96
3
1.97
1.12
1.07
2.21
2.18
18.08
7.5557
7.5465
7.5339
7.0734
7.0528
7.0184
4.8775
4.8649
4.8546
3.7125
2.9233
2.9044
2.8866
2.1650
1.9210
1.7847
1.7663
1.7480
1.7297
1.7125
1.4960
1.4628
1.4445
-0.0000

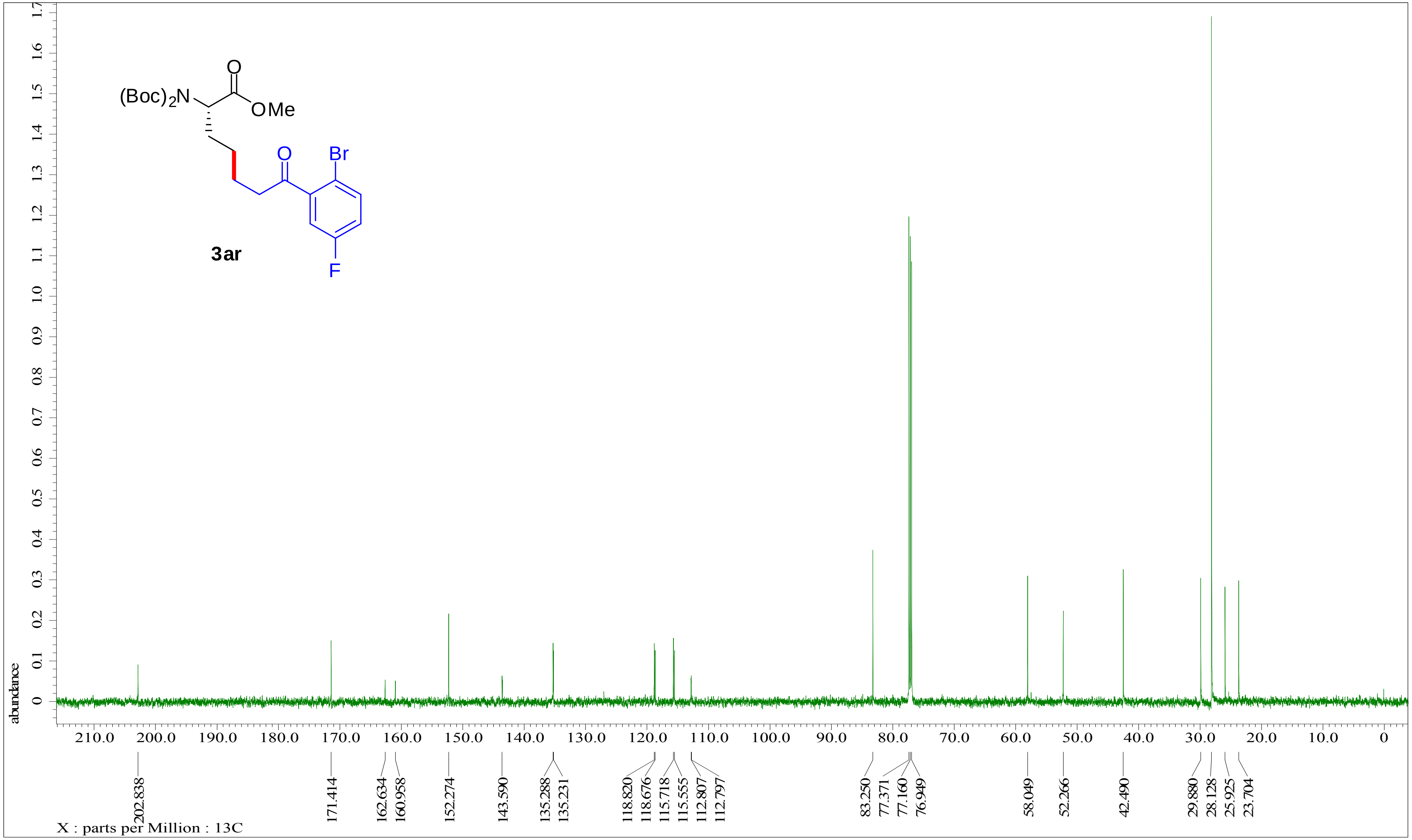
(Boc)2N
OMe
O
Br
F
3ar
abundance
X : parts per Million : 13C
202.838
171.414
162.634
160.958
152.274
143.590
135.288
135.231
118.820
118.676
115.718
115.555
112.807
112.797
83.250
77.371
77.160
76.949
58.049
52.266
42.490
29.880
28.128
25.925
23.704

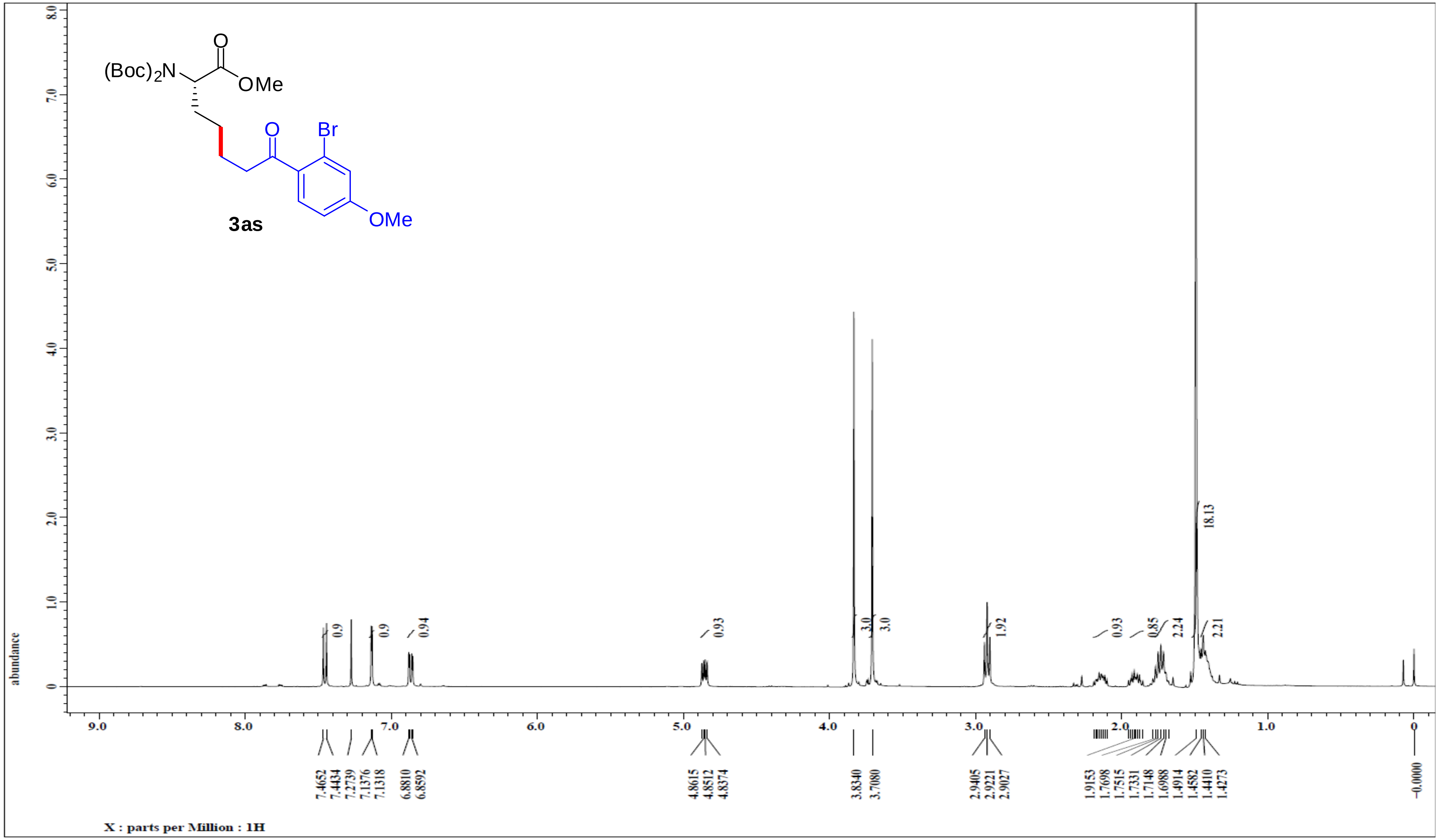
(Boc)2N
O
OMe
O
Br
OMe
3as
abundance
X : parts per Million : 1H

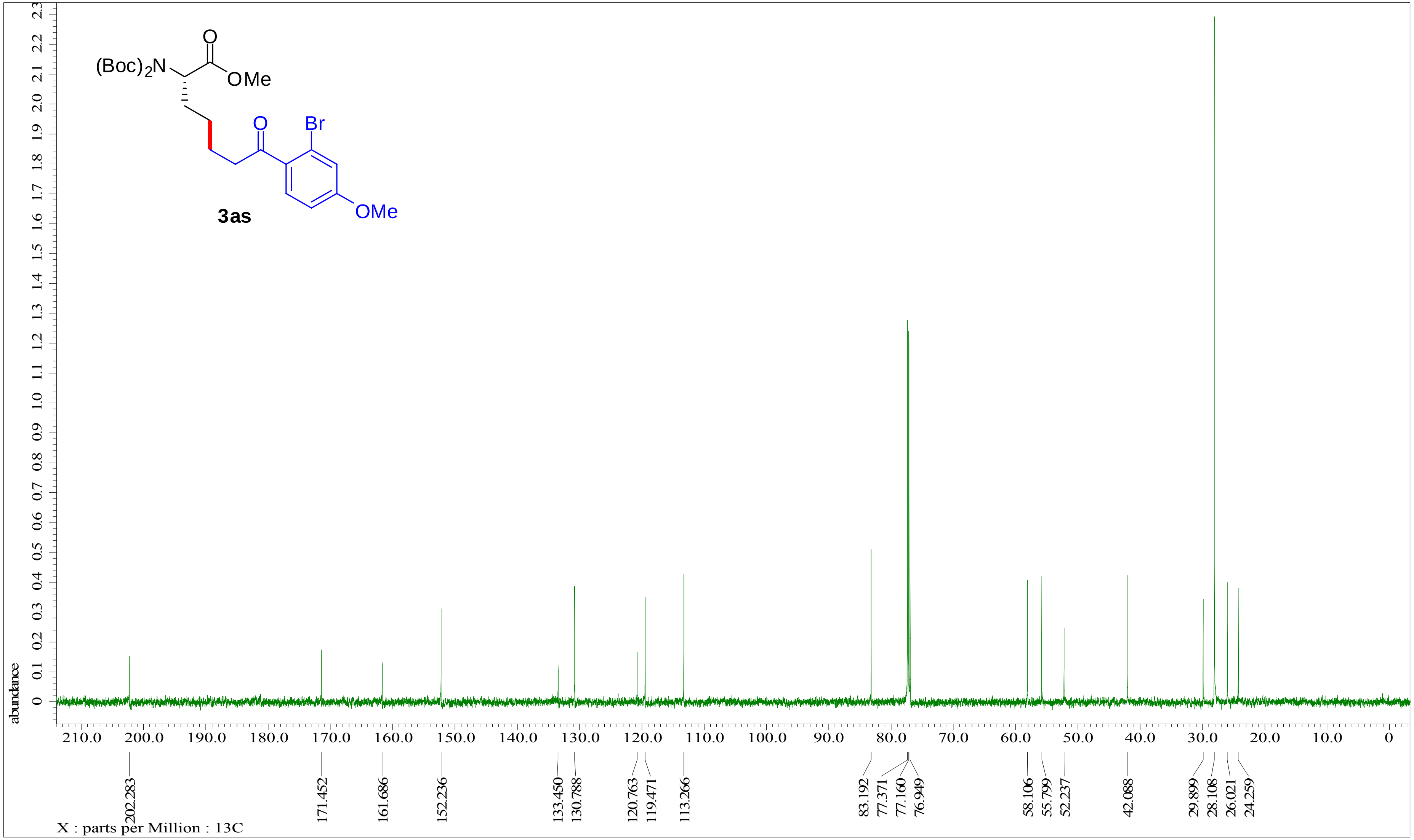

(Boc)$_2$N
O
OMe
O
Br
OMe
3as
abundance
X : parts per Million : 13C
202.283
171.452
161.686
152.236
133.450
130.788
120.763
119.471
113.266
83.192
77.371
77.160
76.949
58.106
55.799
52.237
42.088
29.899
28.108
26.021
24.259

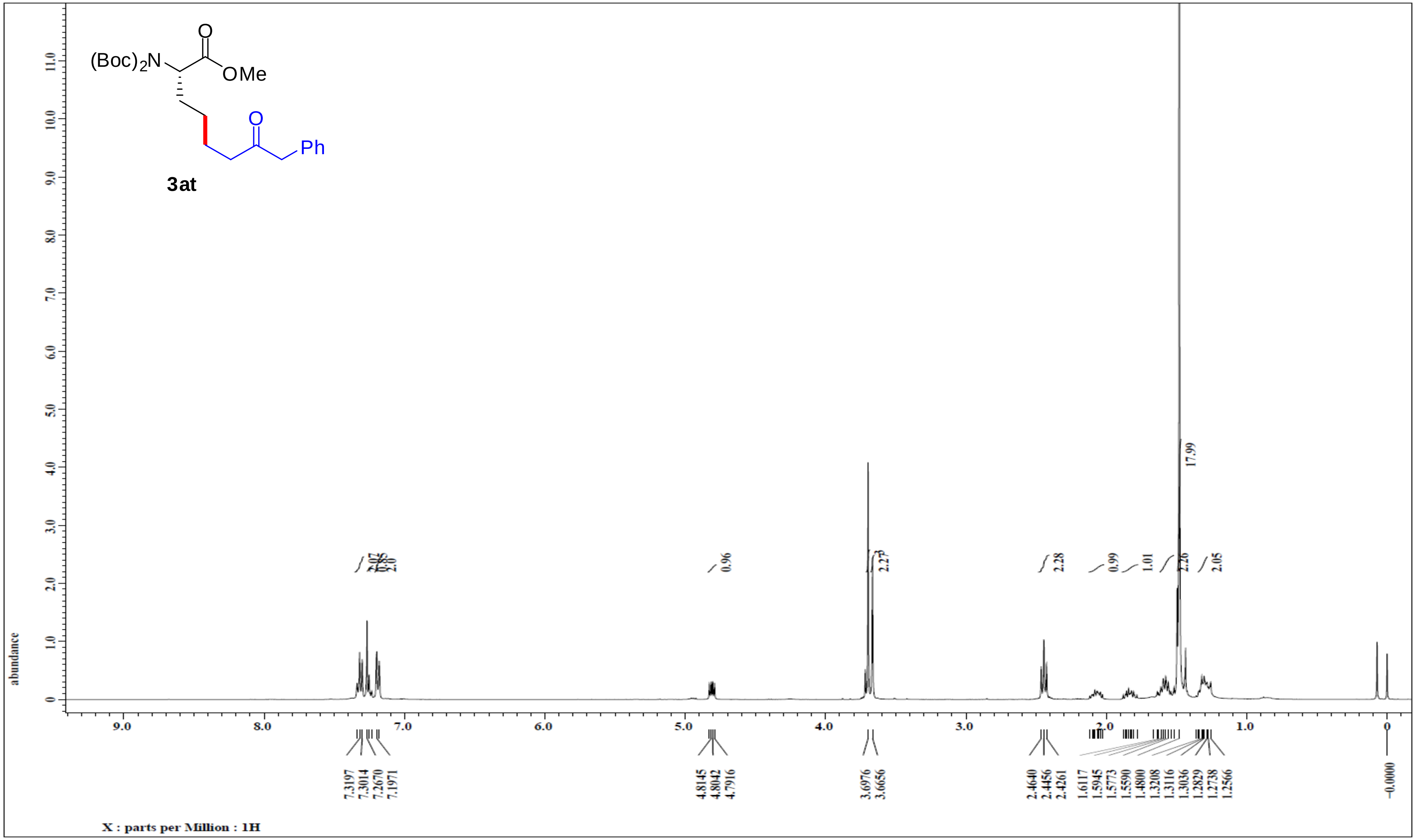
(Boc)2N
O
OMe
O
Ph
3at
abundance
X : parts per Million : 1H
17.99

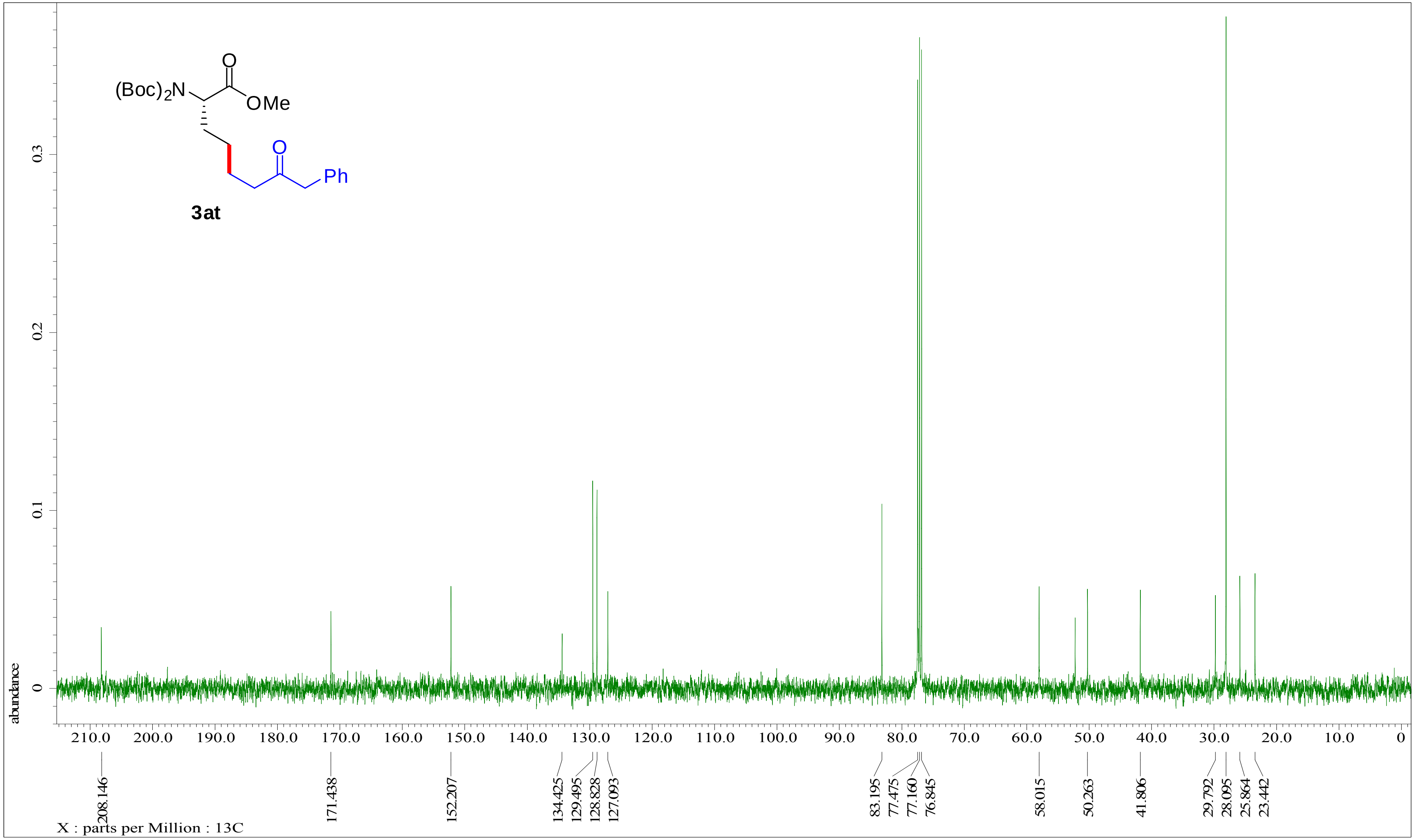
(Boc)2N
OMe
O
O
Ph
3at
abundance
0
0.1
0.2
0.3
210.0 200.0 190.0 180.0 170.0 160.0 150.0 140.0 130.0 120.0 110.0 100.0 90.0 80.0 70.0 60.0 50.0 40.0 30.0 20.0 10.0 0
208.146
171.438
152.207
134.425
129.495
128.828
127.093
83.195
77.475
77.160
76.845
58.015
50.263
41.806
29.792
28.095
25.864
23.442
X : parts per Million : 13C

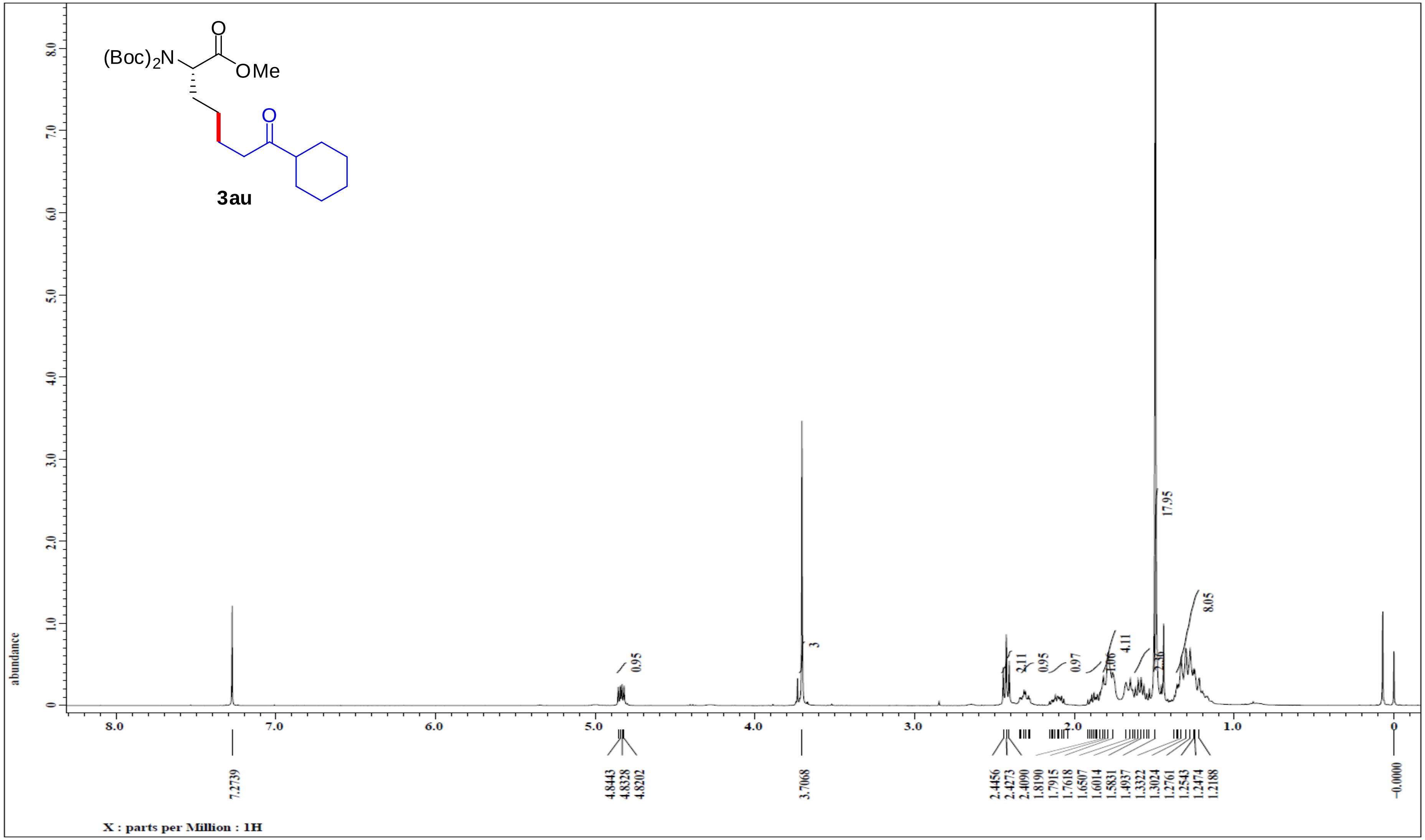

(Boc)2N
OMe
3au
abundance
X : parts per Million : 1H
7.2739
4.8443
4.8328
4.8202
3.7068
2.4456
2.4273
2.4090
1.8190
1.7915
1.7618
1.6507
1.6014
1.5831
1.4937
1.3322
1.3024
1.2761
1.2543
1.2474
1.2188
-0.0000
0.95
3
2.11
0.95
0.97
4.11
17.95
8.05

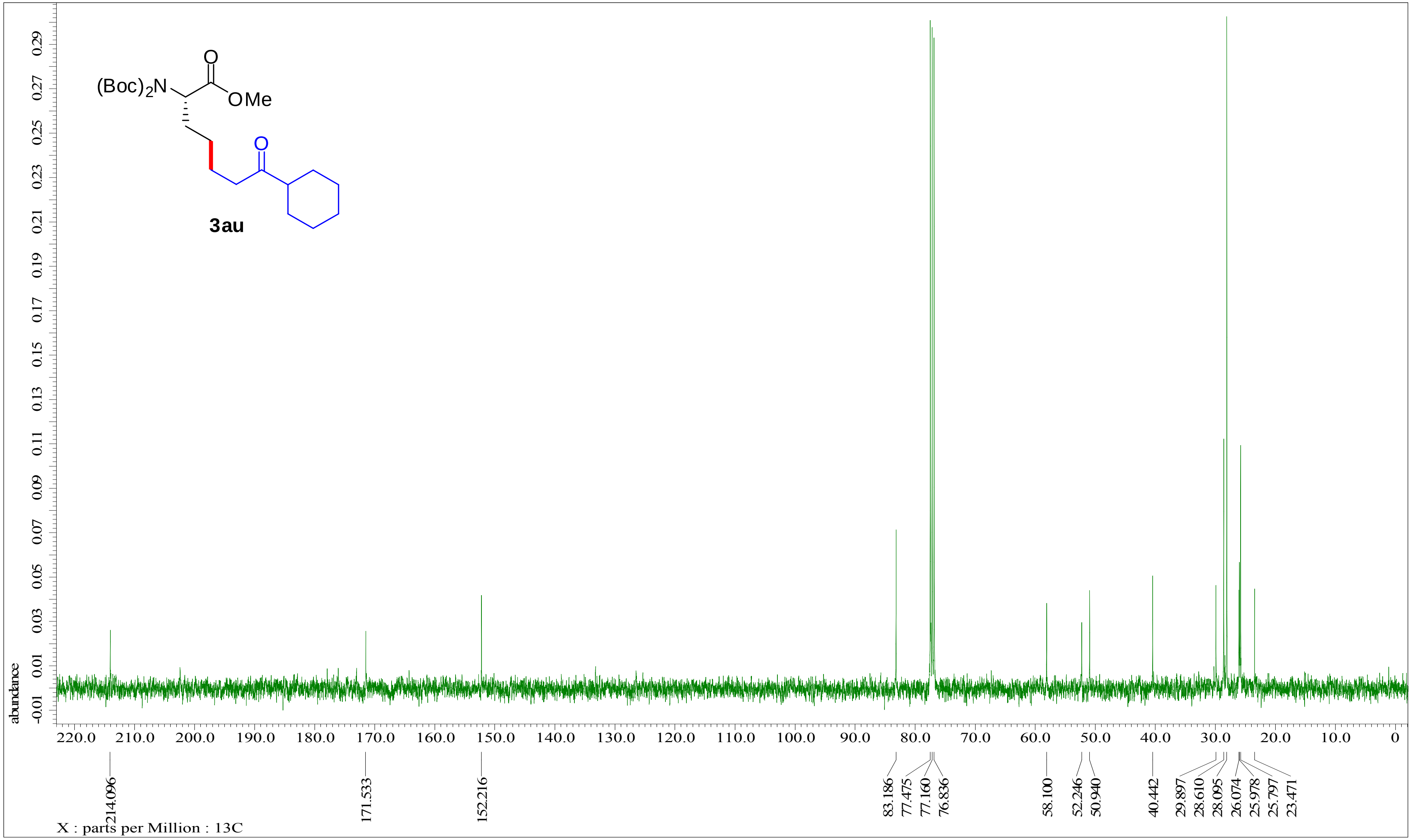

(Boc)2N
O
OMe
O
3au
abundance
0.29
0.27
0.25
0.23
0.21
0.19
0.17
0.15
0.13
0.11
0.09
0.07
0.05
0.03
0.01
-0.01
220.0 210.0 200.0 190.0 180.0 170.0 160.0 150.0 140.0 130.0 120.0 110.0 100.0 90.0 80.0 70.0 60.0 50.0 40.0 30.0 20.0 10.0 0
214.096
171.533
152.216
83.186
77.475
77.160
76.836
58.100
52.246
50.940
40.442
29.897
28.610
28.095
26.074
25.978
25.797
23.471
X : parts per Million : 13C

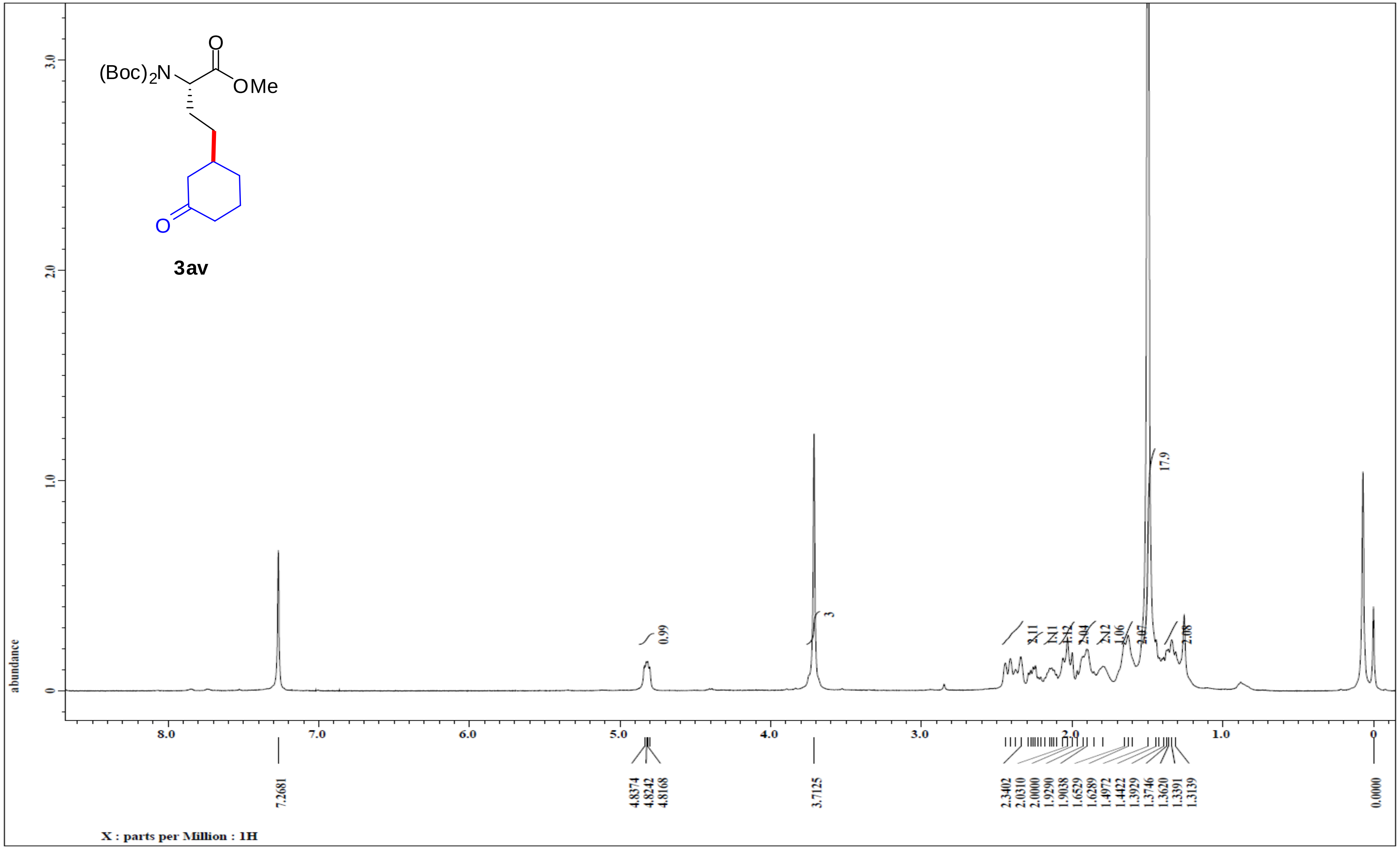
(Boc)2N
O
OMe
O
3av
abundance
X : parts per Million : 1H
7.2681
4.8374
4.8242
4.8168
3.7125
2.3402
2.0310
2.0000
1.9290
1.9038
1.6529
1.6289
1.4972
1.4422
1.3929
1.3746
1.3620
1.3391
1.3139
0.0000
0.99
3
17.9

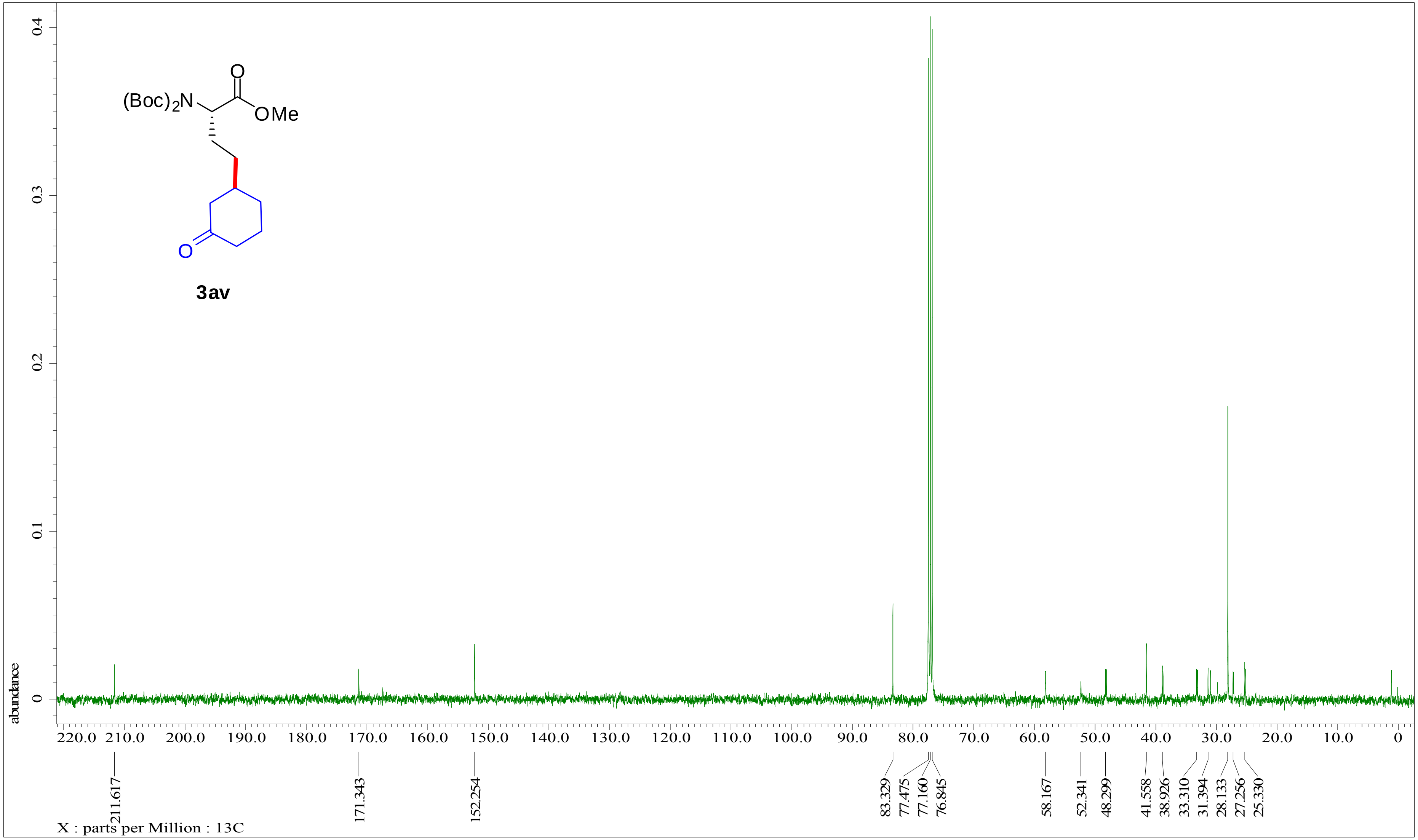

(Boc)2N
O
OMe
O
3av
abundance
0.4
0.3
0.2
0.1
0
220.0 210.0 200.0 190.0 180.0 170.0 160.0 150.0 140.0 130.0 120.0 110.0 100.0 90.0 80.0 70.0 60.0 50.0 40.0 30.0 20.0 10.0 0
211.617
171.343
152.254
83.329
77.475
77.160
76.845
58.167
52.341
48.299
41.558
38.926
33.310
31.394
28.133
27.256
25.330
X : parts per Million : 13C

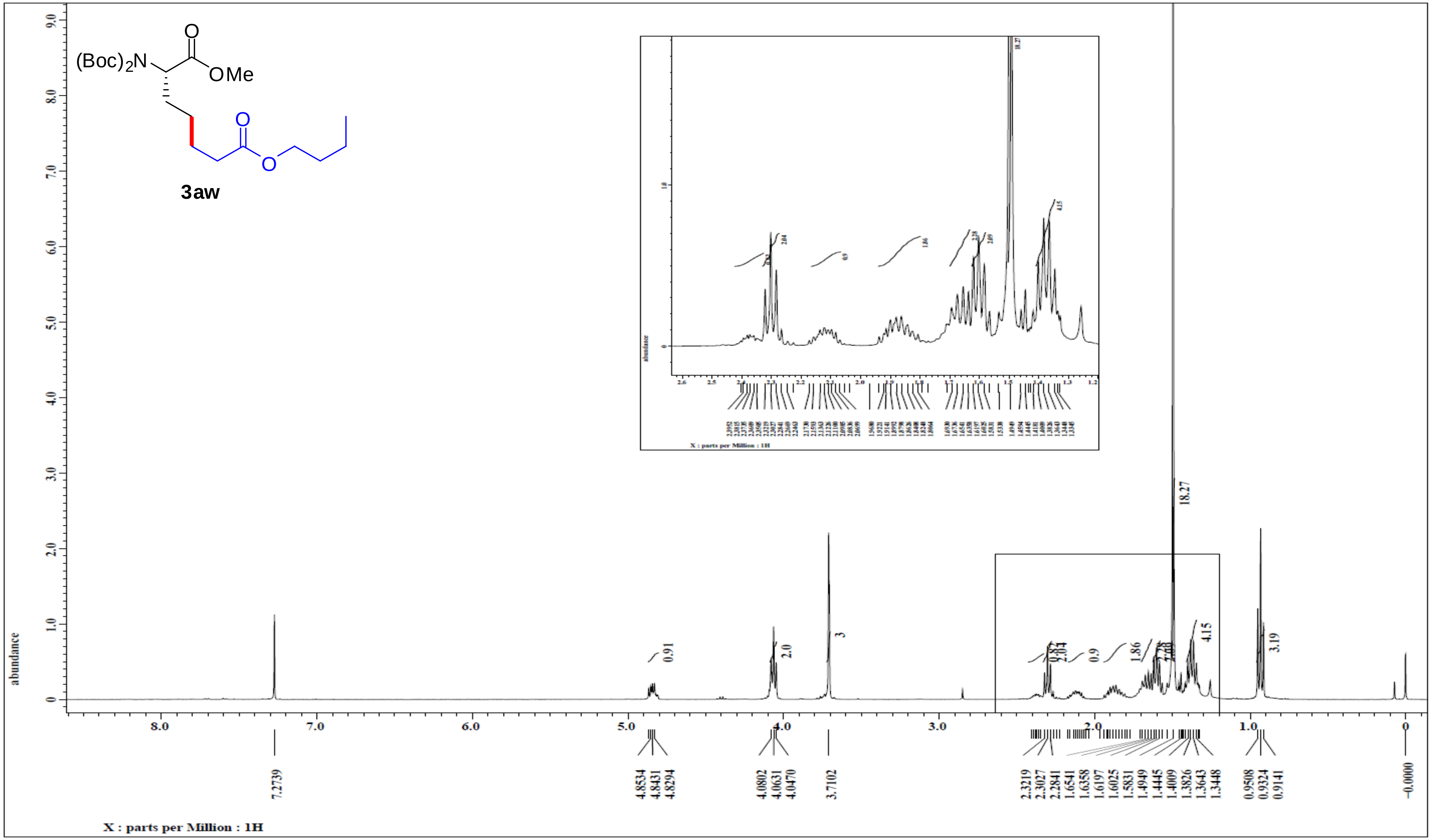

(Boc)$_2$N
O
OMe
O
O
3aw
abundance
X : parts per Million : 1H
18.27
0.91
2.0
3
4.15
3.19
1.86
0.9
7.2739
4.8534
4.8431
4.8294
4.0802
4.0631
4.0470
3.7102
2.3219
2.3027
2.2841
1.6541
1.6358
1.6197
1.6025
1.5831
1.4949
1.4445
1.4009
1.3826
1.3643
1.3448
0.9508
0.9324
0.9141
-0.0000

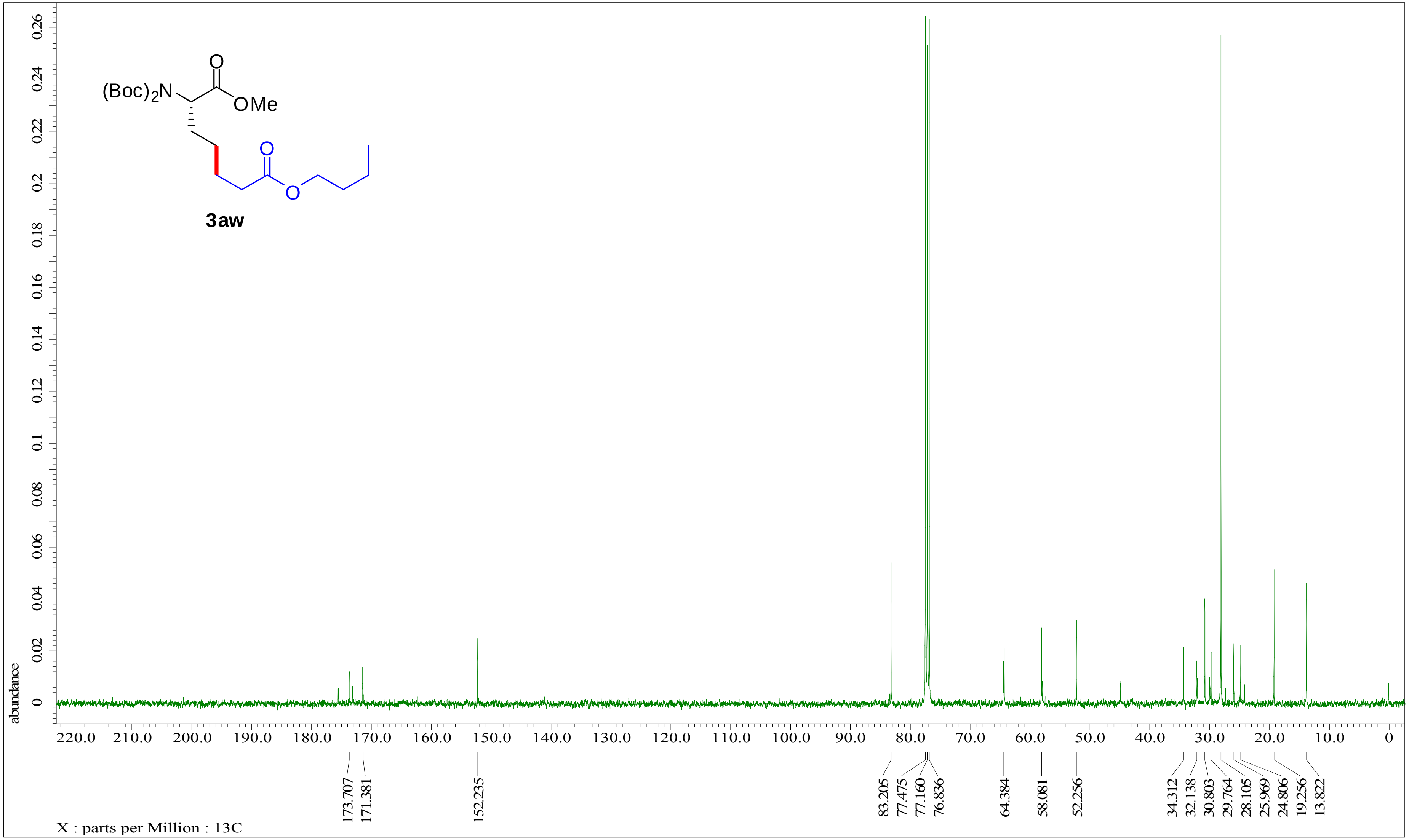
(Boc)2N
OMe
O
O
O
3aw
abundance
X : parts per Million : 13C
173.707
171.381
152.235
83.205
77.475
77.160
76.836
64.384
58.081
52.256
34.312
32.138
30.803
29.764
28.105
25.969
24.806
19.256
13.822

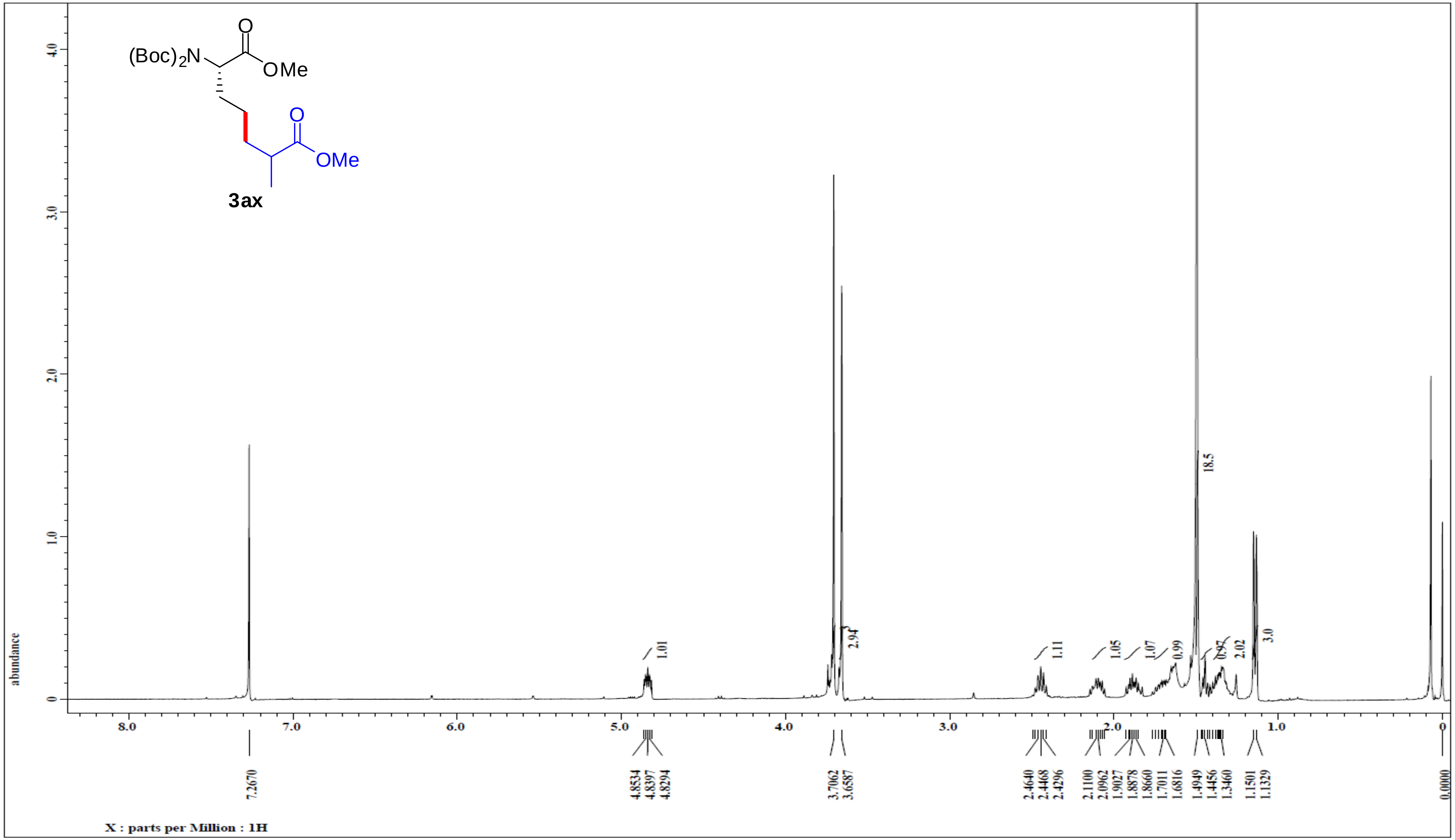
(Boc)2N
OMe
OMe
3ax
abundance
X : parts per Million : 1H
1.01
2.94
1.11
1.05
1.07
0.99
18.5
0.97
2.02
3.0
7.2670
4.8534
4.8397
4.8294
3.7062
3.6587
2.4640
2.4468
2.4296
2.1100
2.0962
1.9027
1.8878
1.8660
1.7011
1.6816
1.4949
1.4456
1.3460
1.1501
1.1329
0.0000

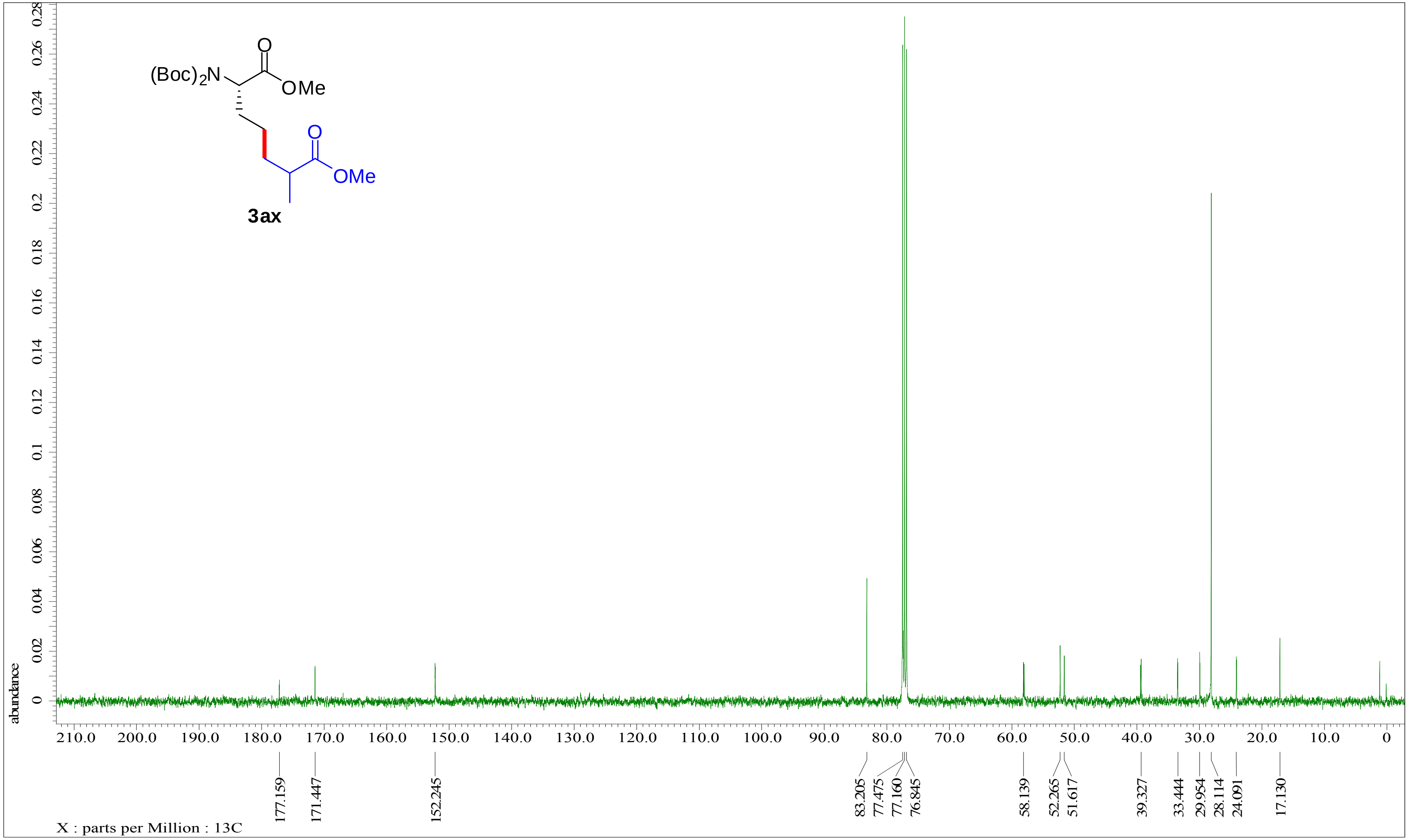
(Boc)2N
OMe
OMe
3ax
abundance
0.28
0.26
0.24
0.22
0.2
0.18
0.16
0.14
0.12
0.1
0.08
0.06
0.04
0.02
0
210.0
200.0
190.0
180.0
170.0
160.0
150.0
140.0
130.0
120.0
110.0
100.0
90.0
80.0
70.0
60.0
50.0
40.0
30.0
20.0
10.0
0
177.159
171.447
152.245
83.205
77.475
77.160
76.845
58.139
52.265
51.617
39.327
33.444
29.954
28.114
24.091
17.130
X : parts per Million : 13C

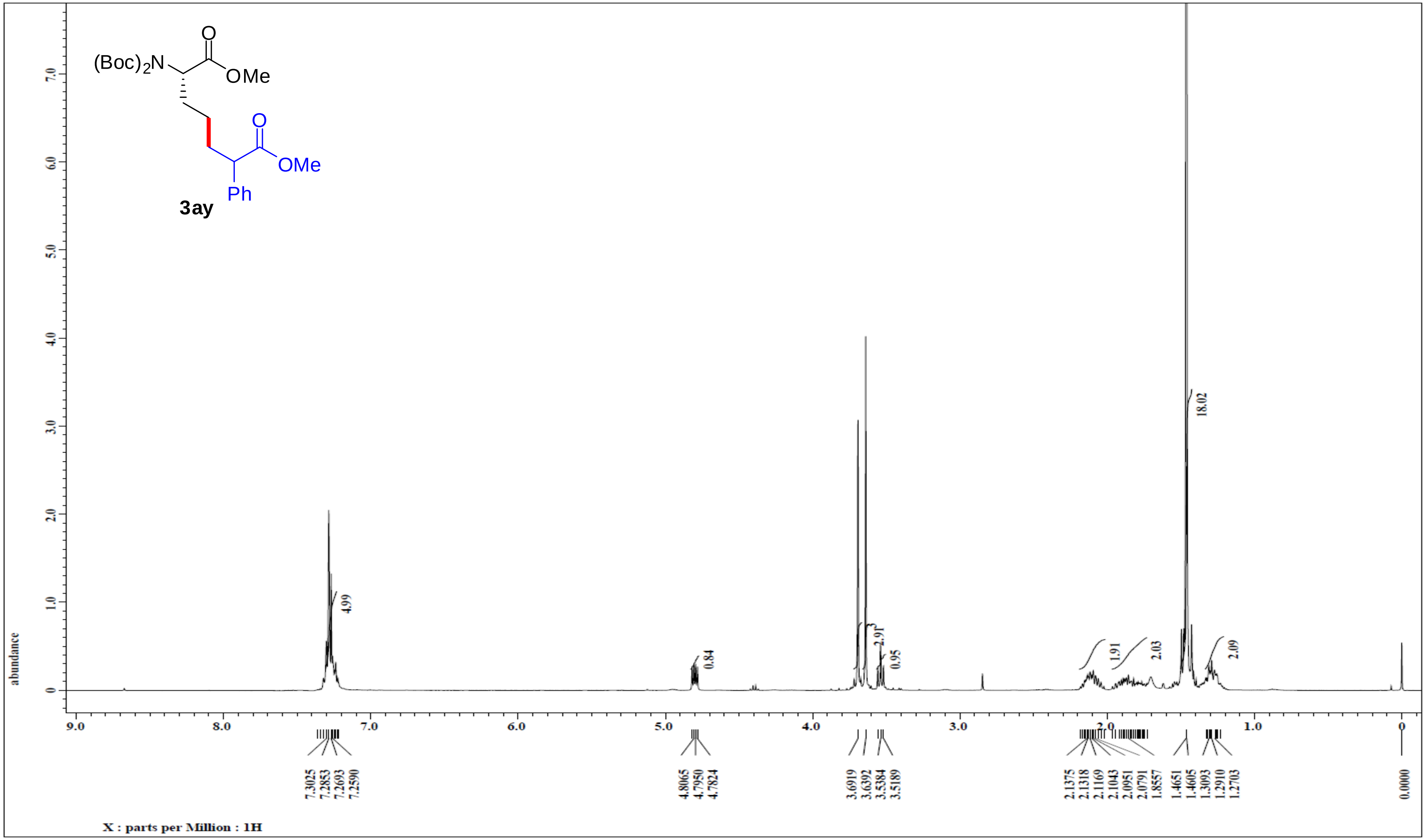

(Boc)2N
O
OMe
O
OMe
Ph
3ay
abundance
7.0
6.0
5.0
4.0
3.0
2.0
1.0
0
4.99
0.84
2.91
0.95
1.91
2.03
18.02
2.09
9.0
8.0
7.0
6.0
5.0
4.0
3.0
2.0
1.0
0
7.3025
7.2853
7.2693
7.2590
4.8065
4.7950
4.7824
3.6919
3.6392
3.5384
3.5189
2.1375
2.1318
2.1169
2.1043
2.0951
2.0791
1.8557
1.4651
1.4605
1.3093
1.2910
1.2703
0.0000
X : parts per Million : 1H

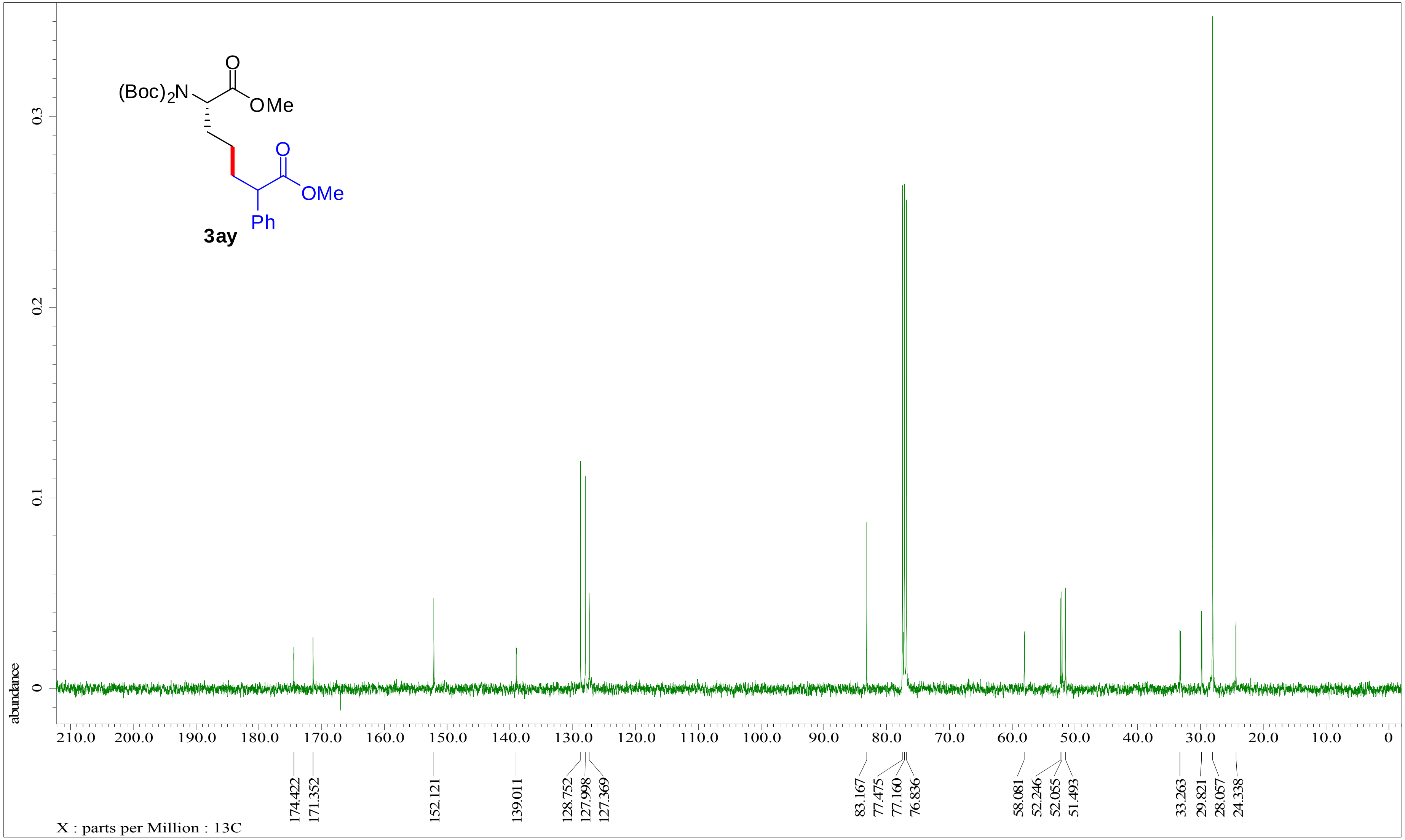
(Boc)2N
O
OMe
O
OMe
Ph
3ay
0.3
0.2
0.1
0
abundance
210.0
200.0
190.0
180.0
170.0
160.0
150.0
140.0
130.0
120.0
110.0
100.0
90.0
80.0
70.0
60.0
50.0
40.0
30.0
20.0
10.0
0
174.422
171.352
152.121
139.011
128.752
127.998
127.369
83.167
77.475
77.160
76.836
58.081
52.246
52.055
51.493
33.263
29.821
28.057
24.338
X : parts per Million : 13C

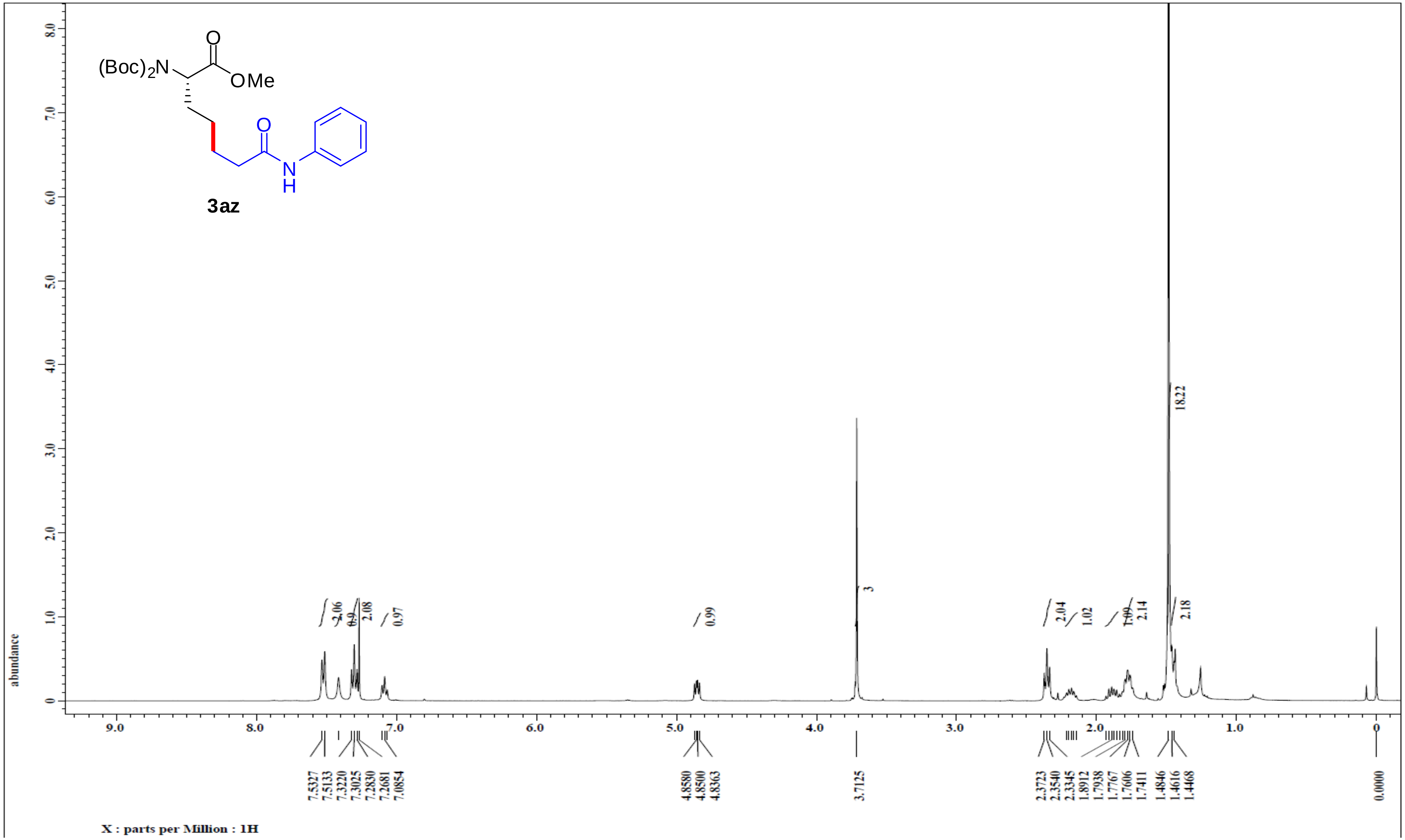
(Boc)2N
O
OMe
O
N
H
3az
abundance
X : parts per Million : 1H
18.22
3
2.06
2.08
0.97
0.99
2.04
1.02
2.14
2.18
7.5327
7.5133
7.3220
7.3025
7.2830
7.2681
7.0854
4.8580
4.8500
4.8363
3.7125
2.3723
2.3540
2.3345
1.8912
1.7938
1.7767
1.7606
1.7411
1.4846
1.4616
1.4468
0.0000

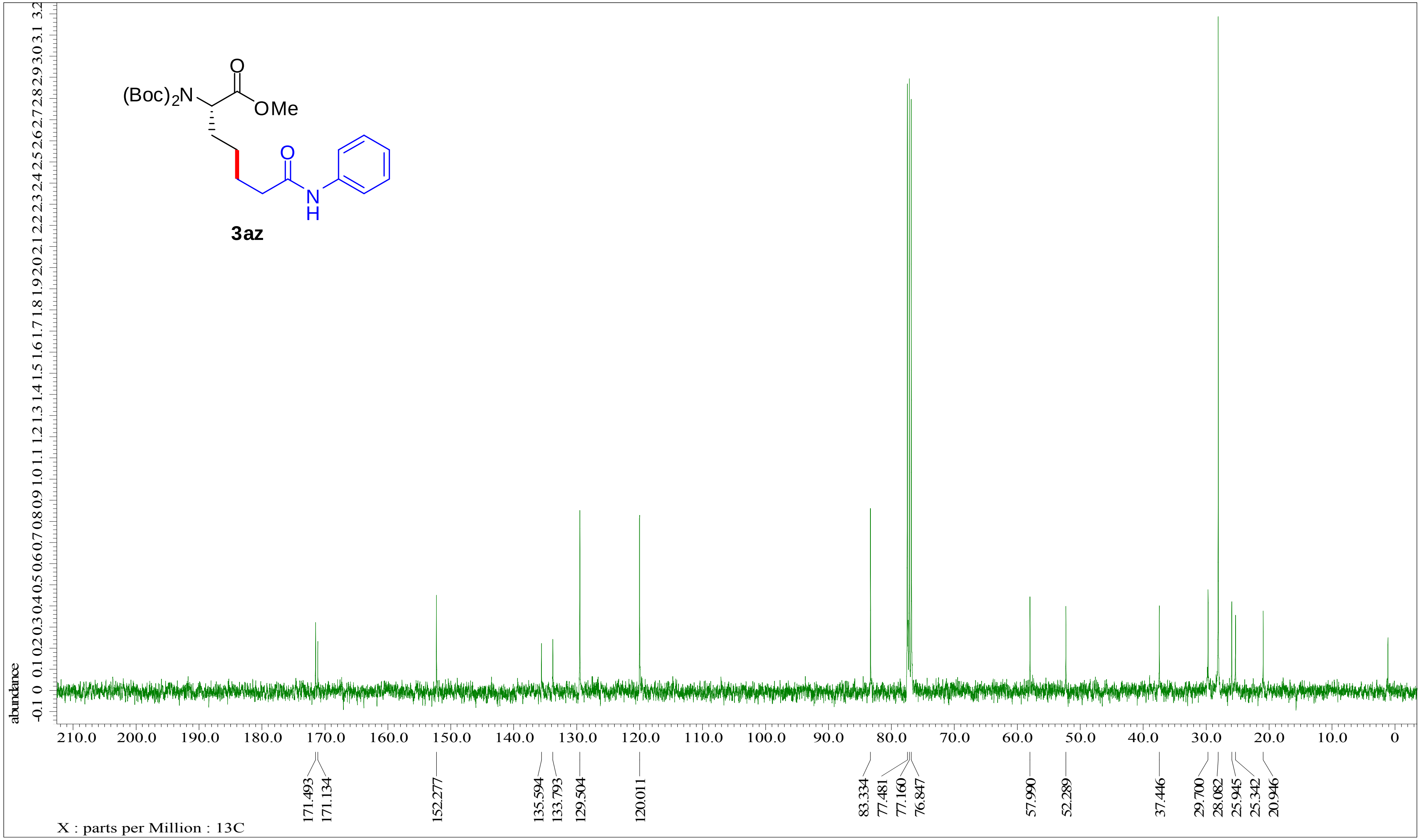
(Boc)2N
O
OMe
O
N
H
3az
abundance
210.0 200.0 190.0 180.0 170.0 160.0 150.0 140.0 130.0 120.0 110.0 100.0 90.0 80.0 70.0 60.0 50.0 40.0 30.0 20.0 10.0 0
171.493
171.134
152.277
135.594
133.793
129.504
120.011
83.334
77.481
77.160
76.847
57.990
52.289
37.446
29.700
28.082
25.945
25.342
20.946
X : parts per Million : 13C

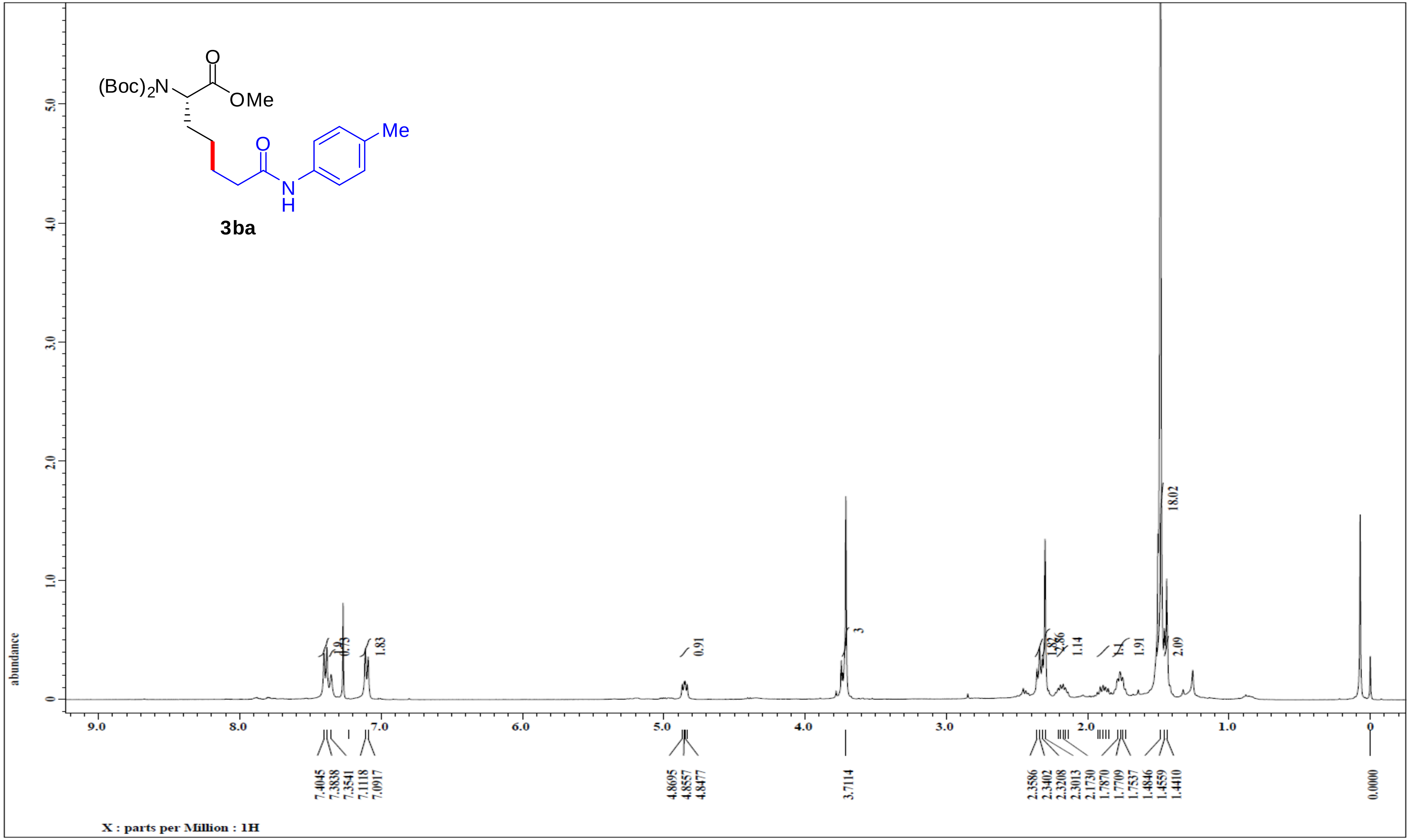
(Boc)2N
OMe
O
Me
N
H
3ba
abundance
X : parts per Million : 1H
7.4045
7.3838
7.3541
7.1118
7.0917
4.8695
4.8557
4.8477
3.7114
2.3586
2.3402
2.3208
2.3013
2.1730
1.7870
1.7709
1.7537
1.4846
1.4559
1.4410
0.0000
1.83
0.91
3
1.14
1.91
2.09
18.02

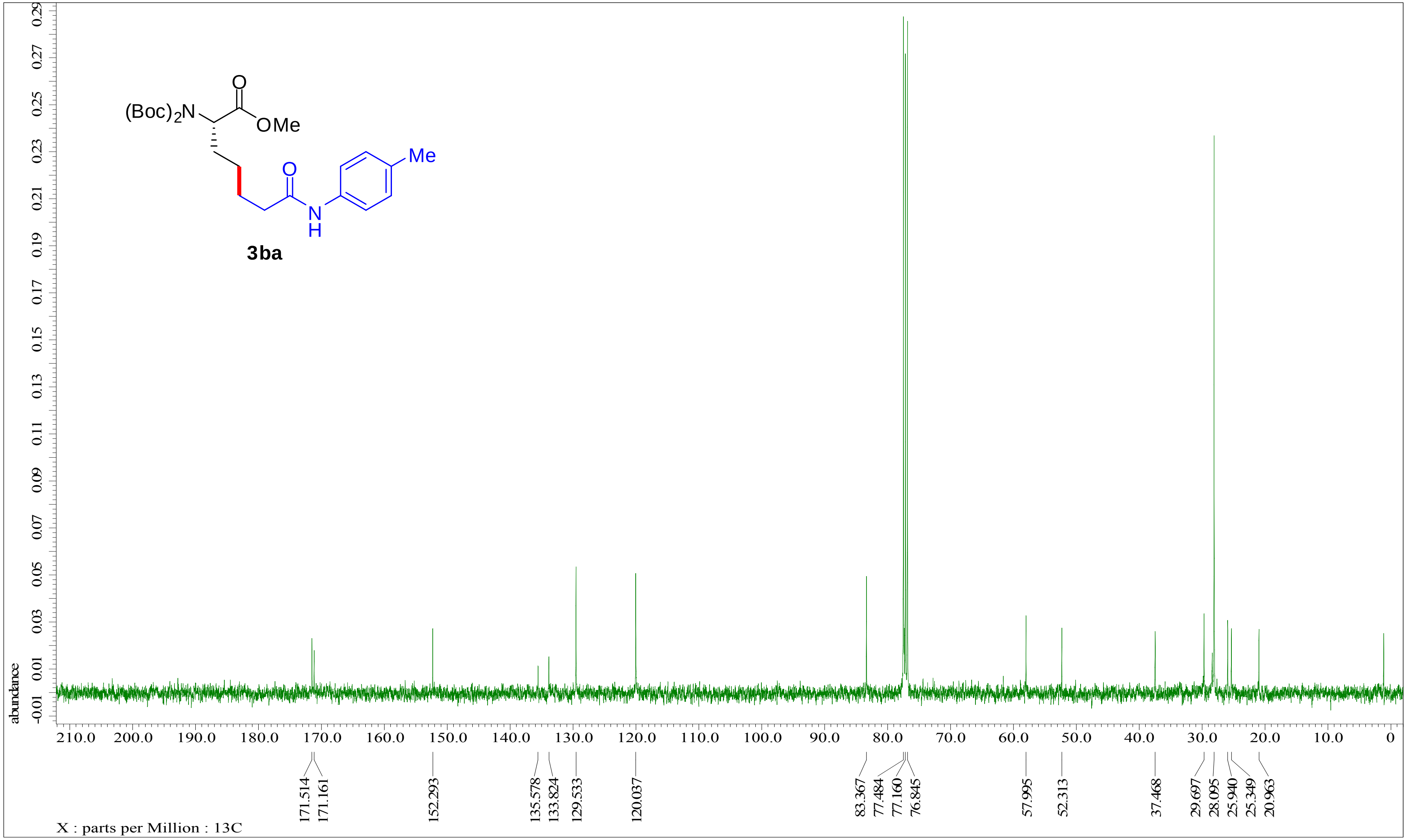
(Boc)2N
O
OMe
O
N
H
Me
3ba
abundance
X : parts per Million : 13C
171.514
171.161
152.293
135.578
133.824
129.533
120.037
83.367
77.484
77.160
76.845
57.995
52.313
37.468
29.697
28.095
25.940
25.349
20.963

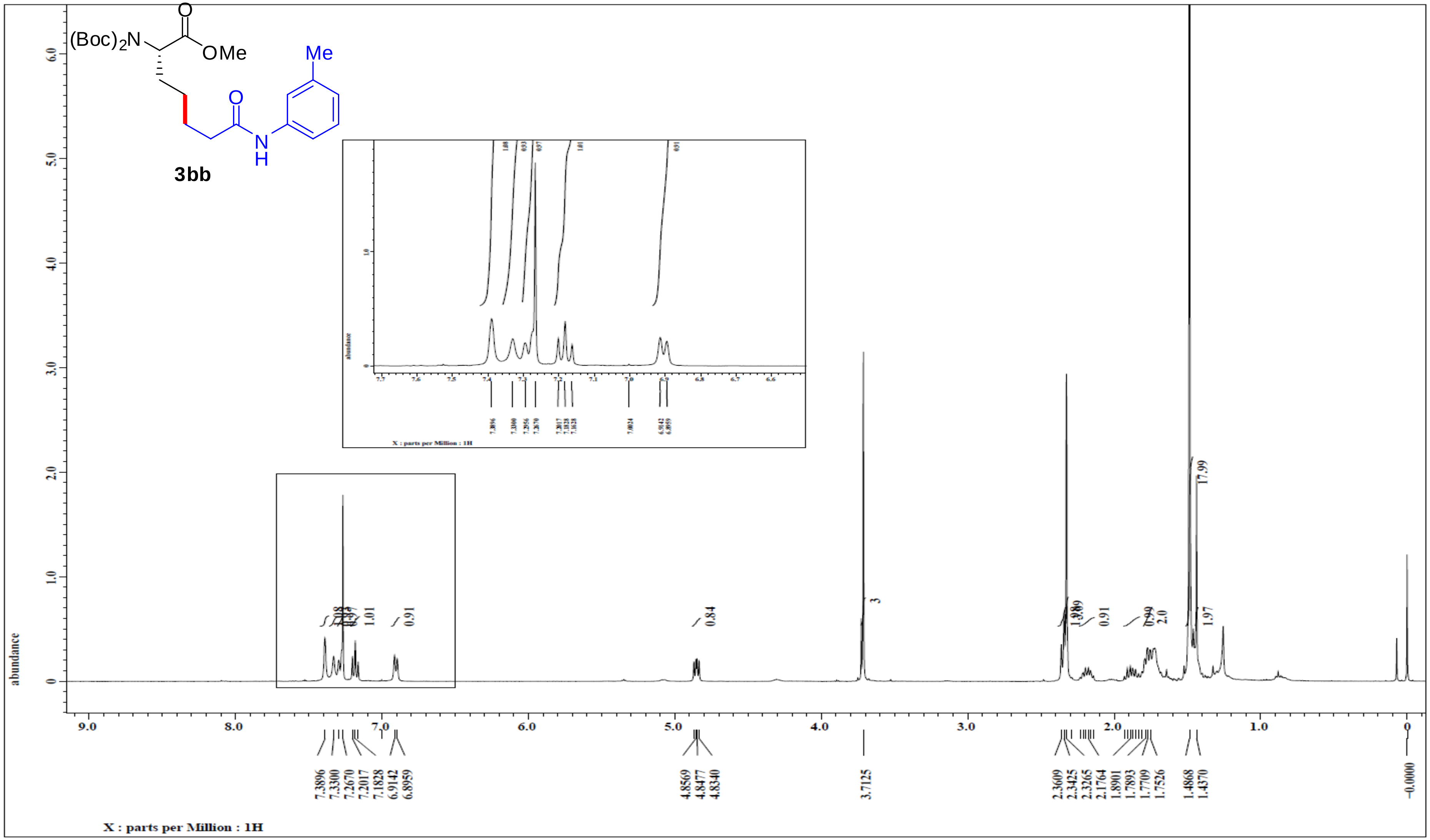
(Boc)2N
OMe
Me
O
N
H
3bb
abundance
X : parts per Million : 1H
7.3896
7.3300
7.2670
7.2017
7.1828
6.9142
6.8959
4.8569
4.8477
4.8340
3.7125
2.3609
2.3425
2.3265
2.1764
1.8901
1.7893
1.7709
1.7526
1.4868
1.4370
-0.0000
1.01
0.91
0.84
3
0.91
2.0
1.97
17.99

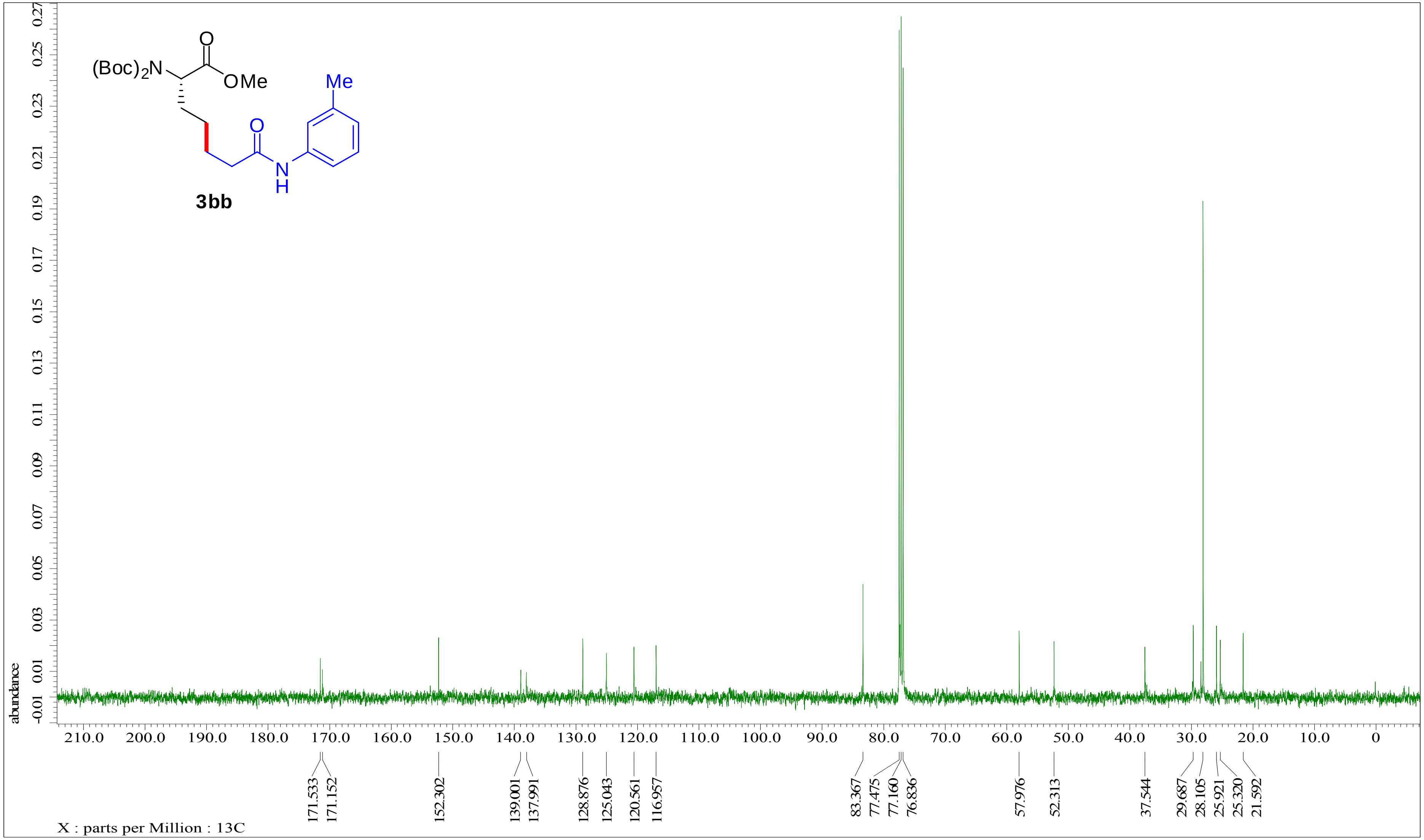
(Boc)2N
OMe
Me
O
N
H
3bb
abundance
X : parts per Million : 13C
171.533
171.152
152.302
139.001
137.991
128.876
125.043
120.561
116.957
83.367
77.475
77.160
76.836
57.976
52.313
37.544
29.687
28.105
25.921
25.320
21.592

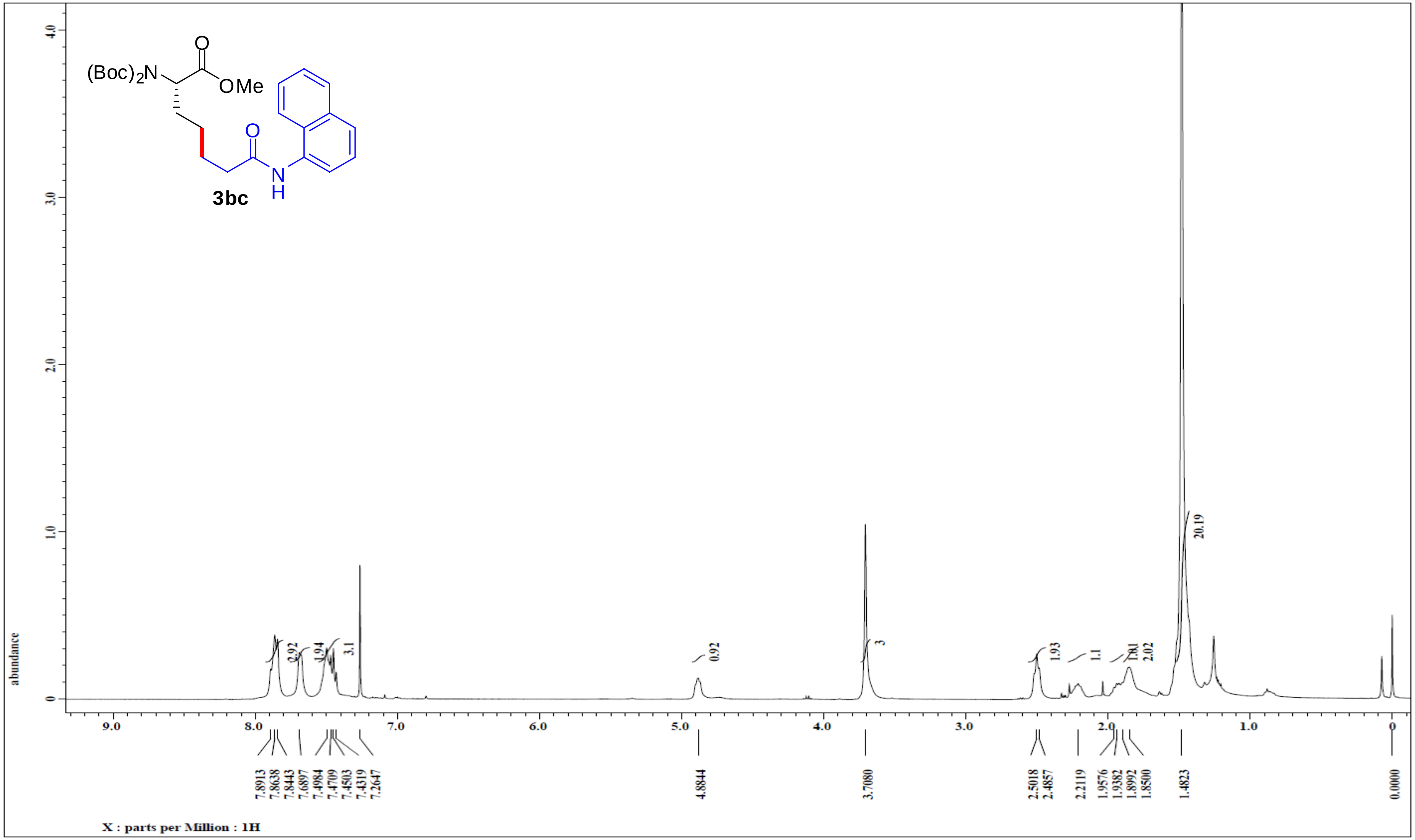
(Boc)2N
O
OMe
O
N
H
3bc
abundance
X : parts per Million : 1H
2.92
1.94
3.1
0.92
3
1.93
1.1
1.81
2.02
20.19
7.8913
7.8638
7.8443
7.6897
7.4984
7.4709
7.4503
7.4319
7.2647
4.8844
3.7080
2.5018
2.4857
2.2119
1.9576
1.9382
1.8992
1.8500
1.4823
0.0000

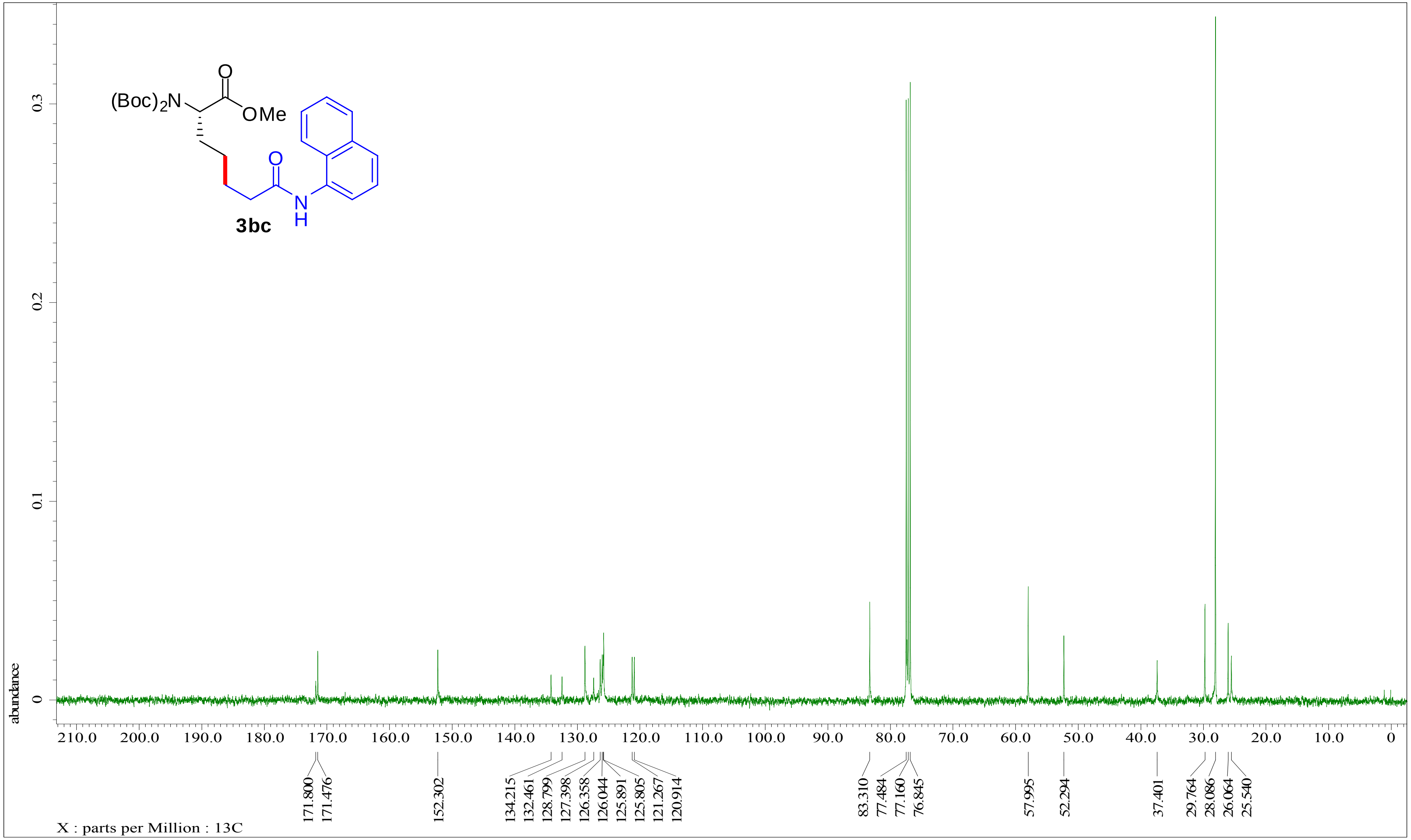
(Boc)2N
O
OMe
O
N
H
3bc
abundance
0.3
0.2
0.1
0
210.0
200.0
190.0
180.0
170.0
160.0
150.0
140.0
130.0
120.0
110.0
100.0
90.0
80.0
70.0
60.0
50.0
40.0
30.0
20.0
10.0
0
171.800
171.476
152.302
134.215
132.461
128.799
127.398
126.358
126.044
125.891
125.805
121.267
120.914
83.310
77.484
77.160
76.845
57.995
52.294
37.401
29.764
28.086
26.064
25.540
X : parts per Million : 13C

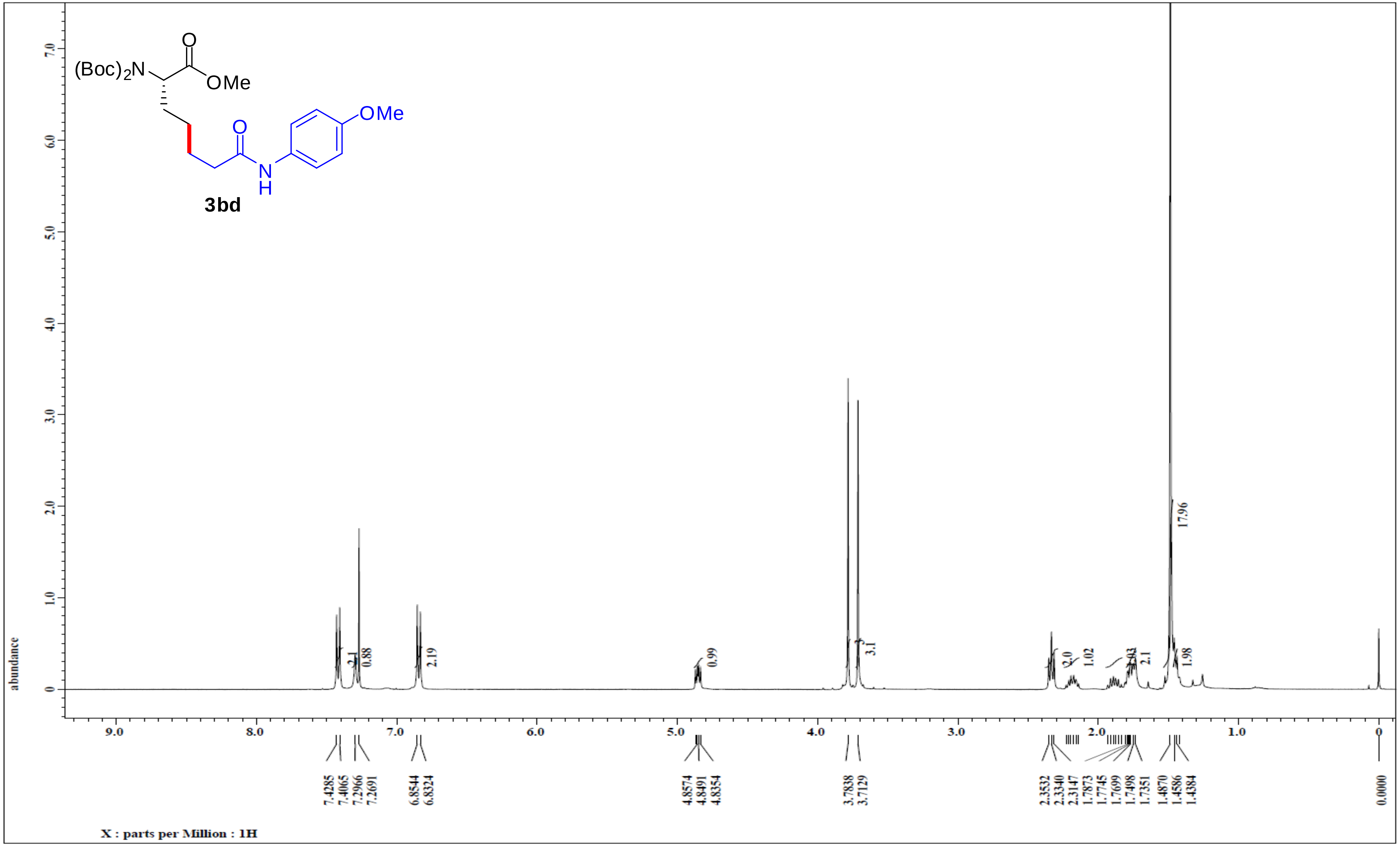
(Boc)2N
OMe
OMe
N
H
O
O
3bd
X : parts per Million : 1H
abundance
7.4285
7.4065
7.2966
7.2691
6.8544
6.8324
4.8574
4.8491
4.8354
3.7838
3.7129
2.3532
2.3340
2.3147
1.7873
1.7745
1.7699
1.7498
1.7351
1.4870
1.4586
1.4384
0.0000
2.1
0.88
2.19
0.99
3.1
2.0
1.02
2.1
1.98
17.96

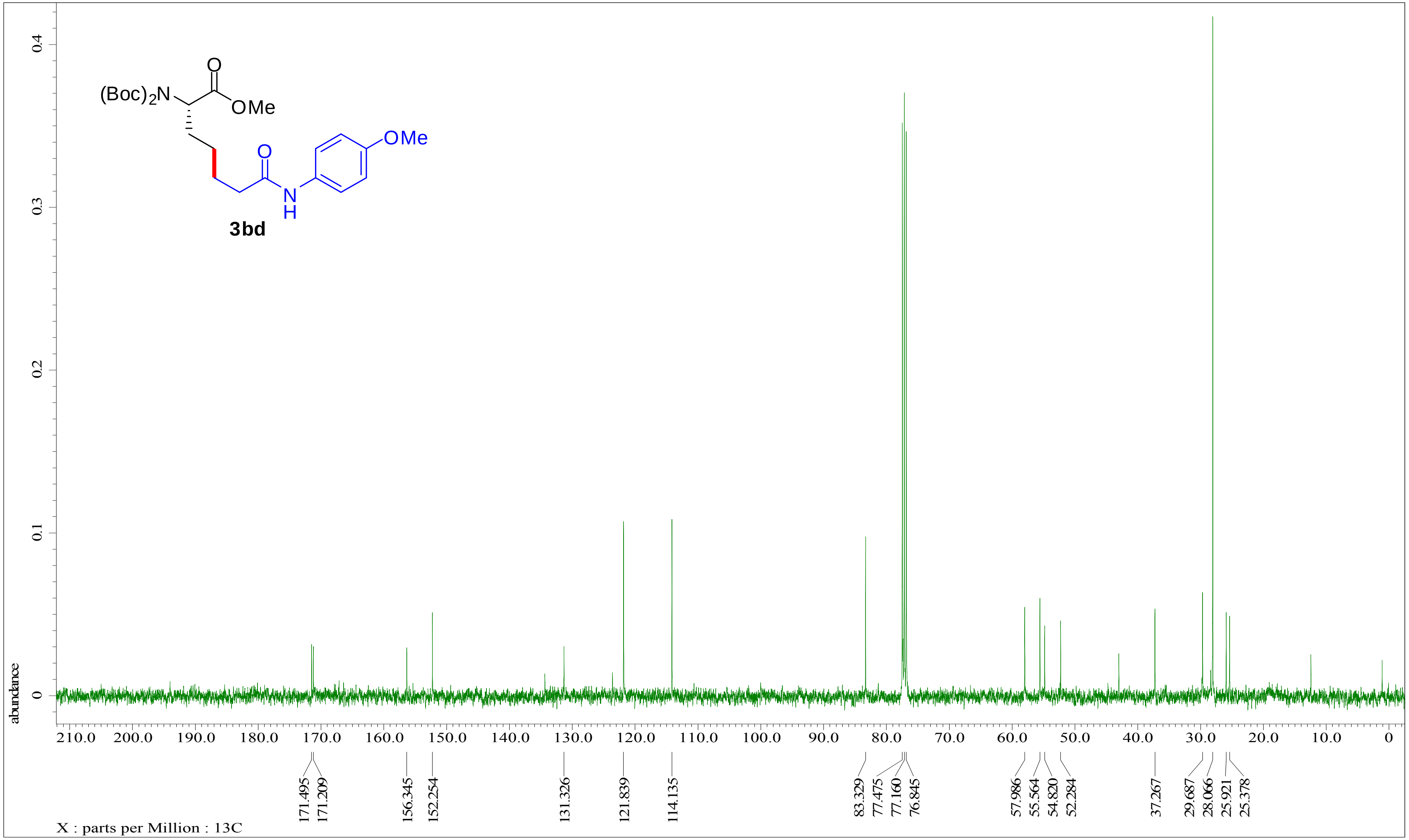
(Boc)2N
OMe
OMe
N
H
3bd
abundance
0.4
0.3
0.2
0.1
0
210.0 200.0 190.0 180.0 170.0 160.0 150.0 140.0 130.0 120.0 110.0 100.0 90.0 80.0 70.0 60.0 50.0 40.0 30.0 20.0 10.0 0
171.495
171.209
156.345
152.254
131.326
121.839
114.135
83.329
77.475
77.160
76.845
57.986
55.564
54.820
52.284
37.267
29.687
28.066
25.921
25.378
X : parts per Million : 13C

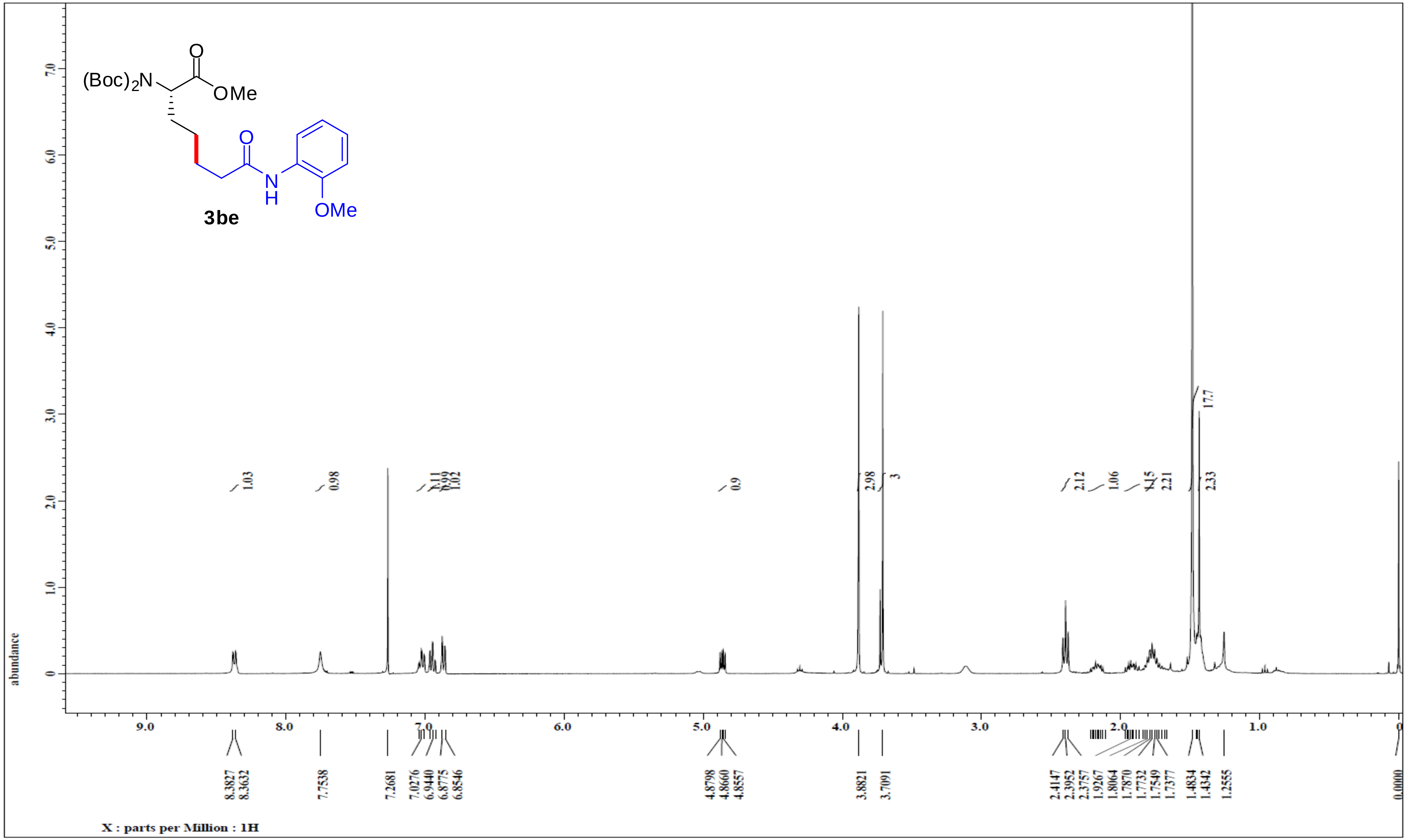
(Boc)2N
O
OMe
O
N
H
OMe
3be
abundance
X : parts per Million : 1H
1.03
0.98
1.11
0.99
1.02
0.9
2.98
3
2.12
1.06
1.15
2.21
17.7
2.33
8.3827
8.3632
7.7538
7.2681
7.0276
6.9440
6.8775
6.8546
4.8798
4.8660
4.8557
3.8821
3.7091
2.4147
2.3952
2.3757
1.9267
1.8064
1.7870
1.7732
1.7549
1.7377
1.4834
1.4342
1.2555
0.0000

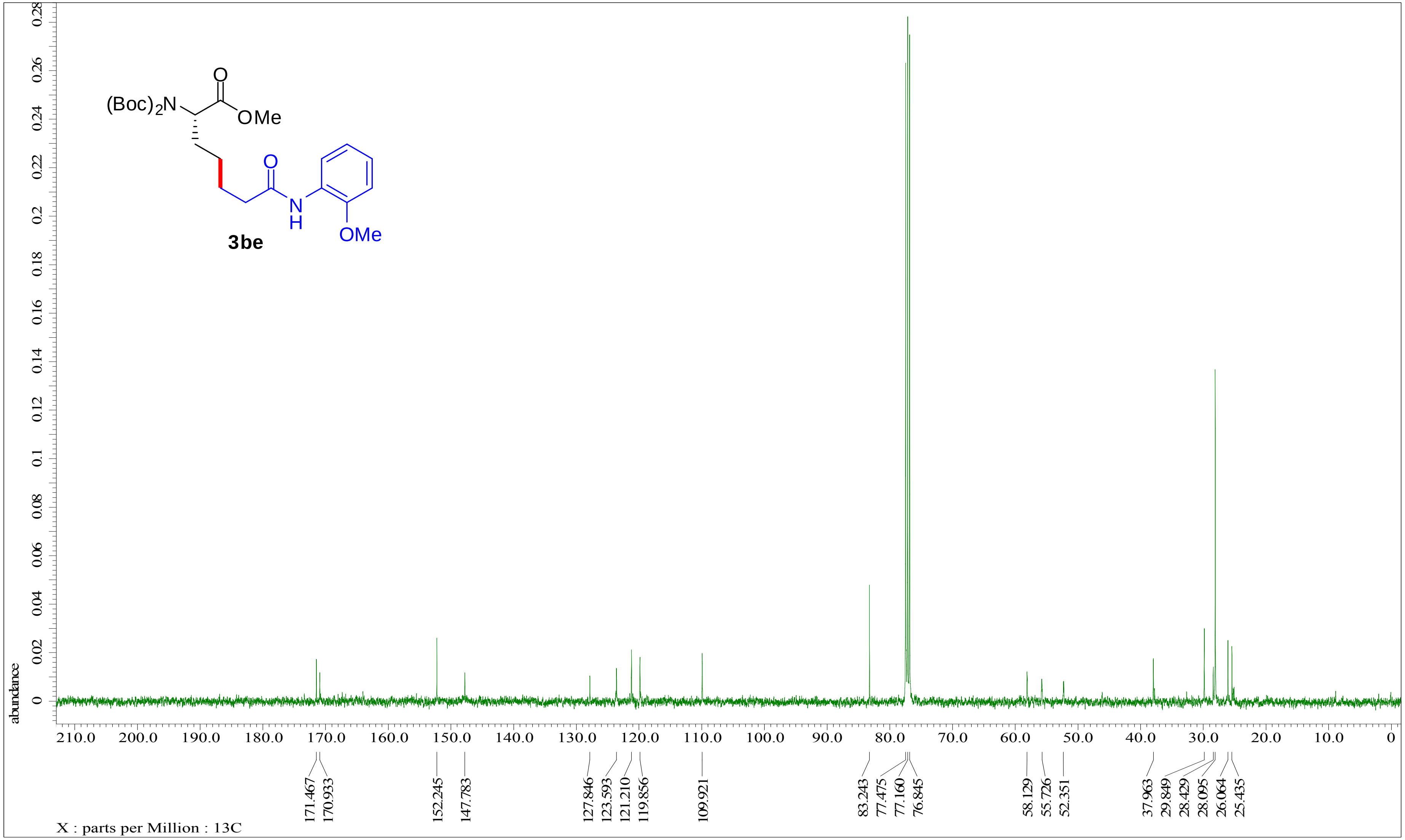
(Boc)2N
O
OMe
O
N
H
OMe
3be
abundance
X : parts per Million : 13C
171.467
170.933
152.245
147.783
127.846
123.593
121.210
119.856
109.921
83.243
77.475
77.160
76.845
58.129
55.726
52.351
37.963
29.849
28.429
28.095
26.064
25.435

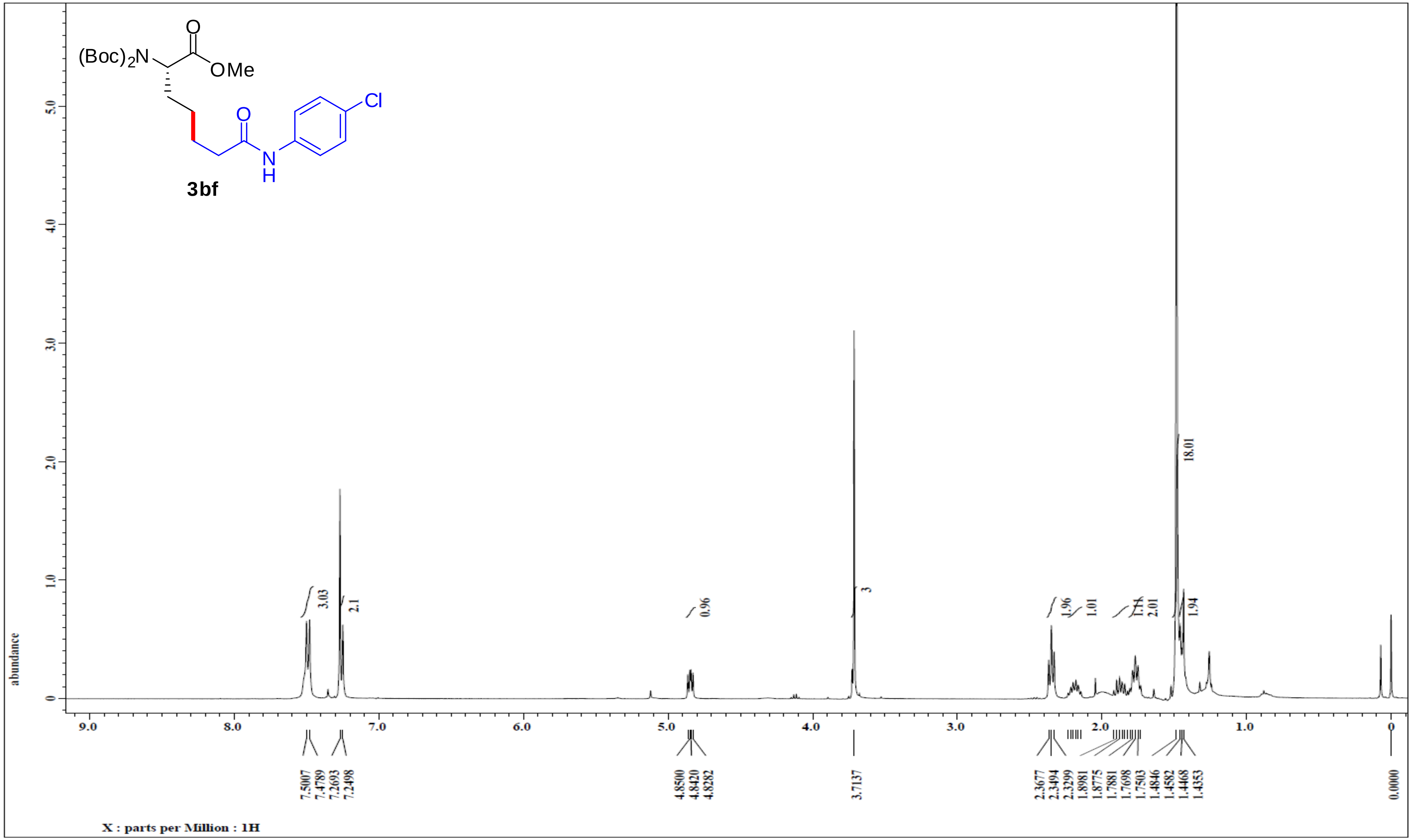

(Boc)2N
O
OMe
O
N
H
Cl
3bf
abundance
X : parts per Million : 1H

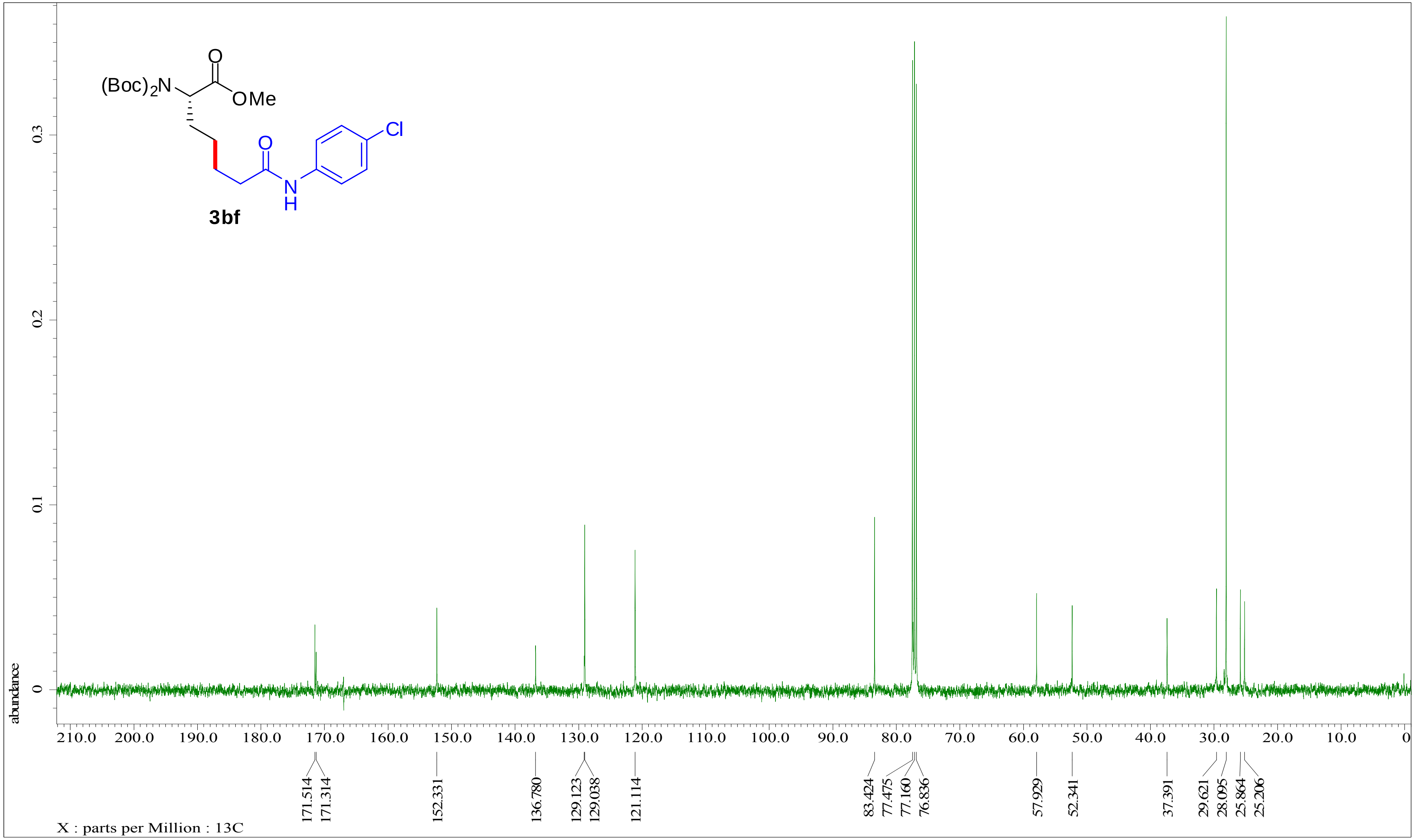
(Boc)2N
O
OMe
O
N
H
Cl
3bf
abundance
0
0.1
0.2
0.3
210.0 200.0 190.0 180.0 170.0 160.0 150.0 140.0 130.0 120.0 110.0 100.0 90.0 80.0 70.0 60.0 50.0 40.0 30.0 20.0 10.0 0
171.514
171.314
152.331
136.780
129.123
129.038
121.114
83.424
77.475
77.160
76.836
57.929
52.341
37.391
29.621
28.095
25.864
25.206
X : parts per Million : 13C

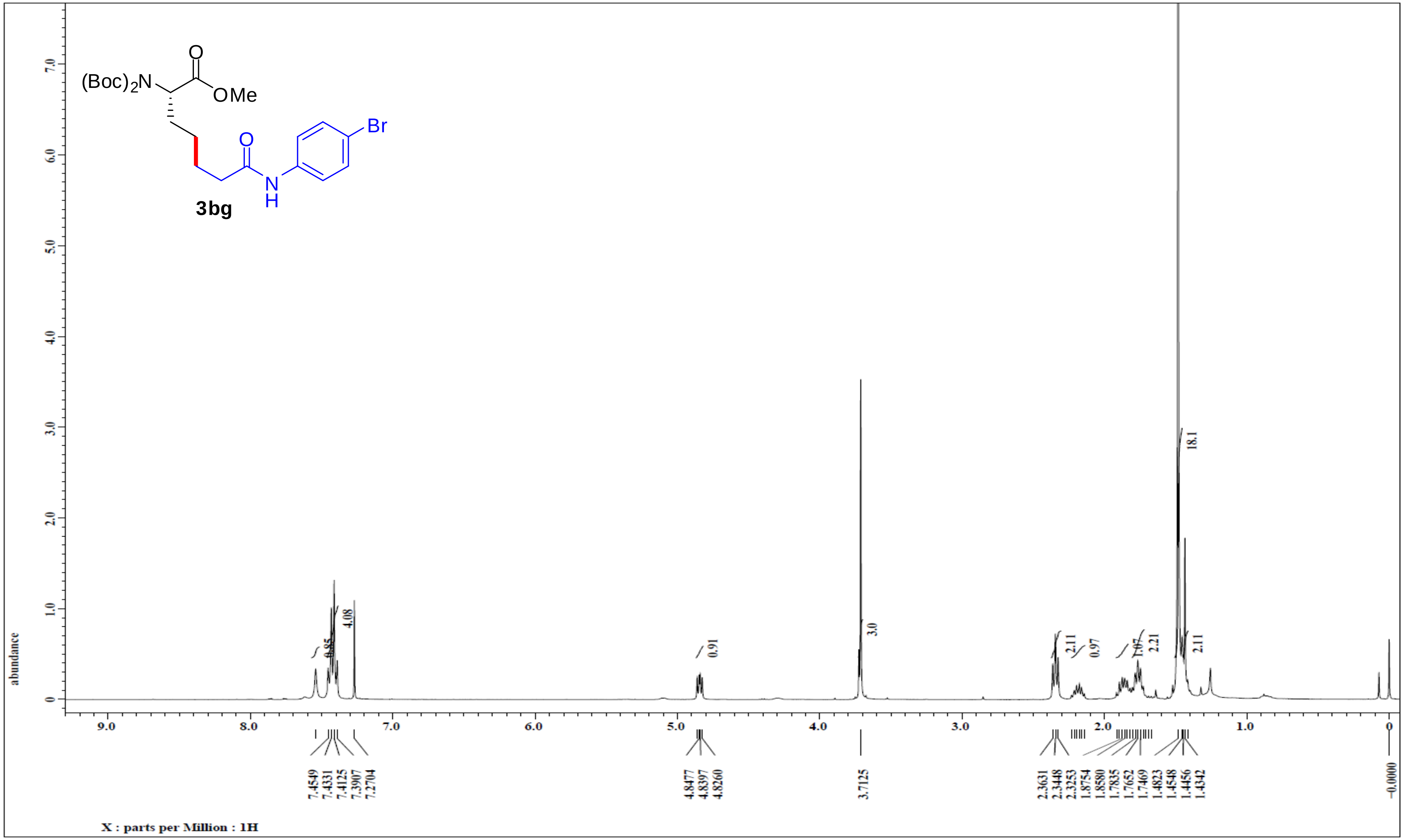

(Boc)2N
O
OMe
O
Br
N
H
3bg
abundance
7.0
6.0
5.0
4.0
3.0
2.0
1.0
0
0.85
4.08
0.91
3.0
2.11
0.97
1.07
2.21
18.1
2.11
9.0
8.0
7.0
6.0
5.0
4.0
3.0
2.0
1.0
0
7.4549
7.4331
7.4125
7.3907
7.2704
4.8477
4.8397
4.8260
3.7125
2.3631
2.3448
2.3253
1.8754
1.8580
1.7835
1.7652
1.7469
1.4823
1.4548
1.4456
1.4342
-0.0000
X : parts per Million : 1H

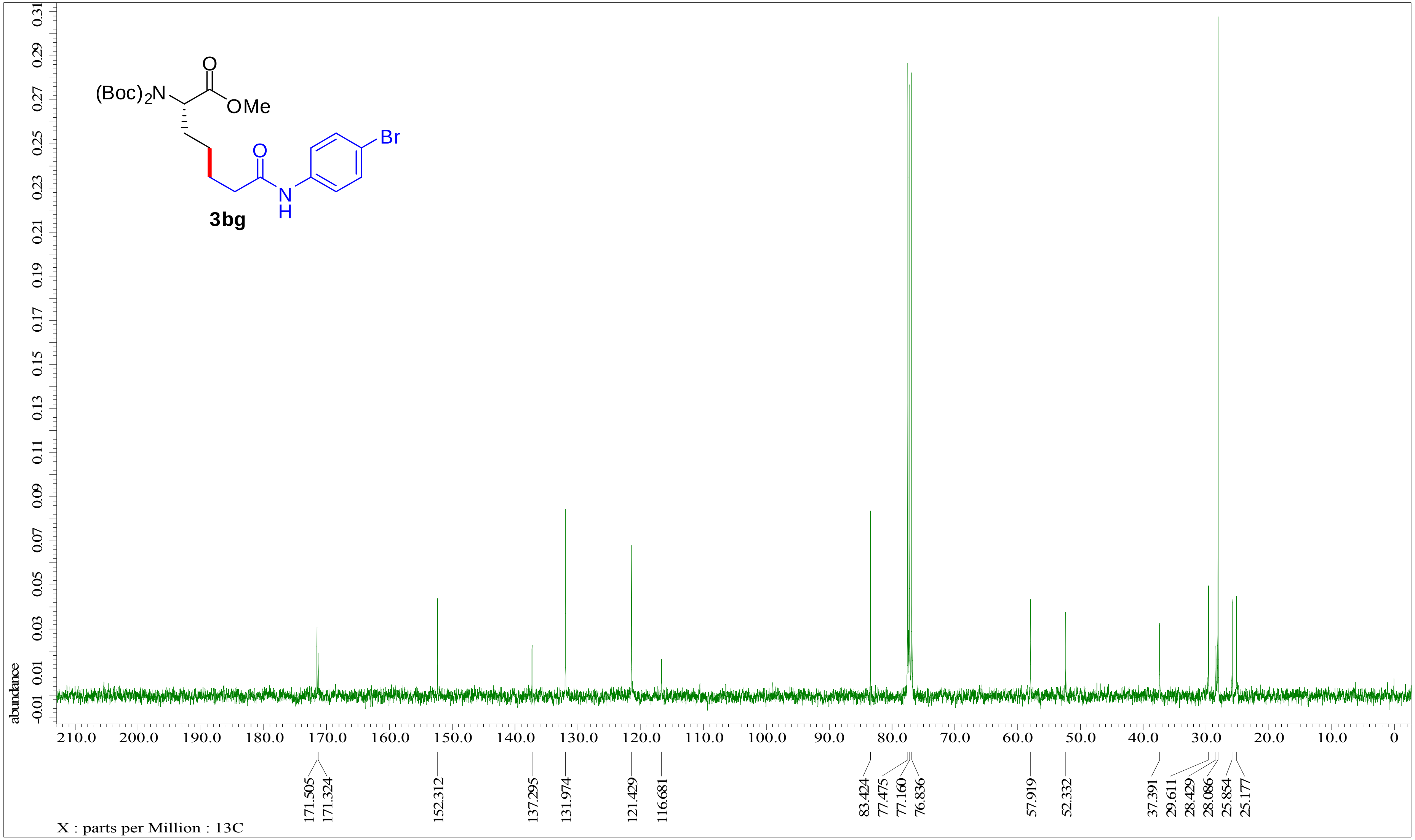
(Boc)2N
O
OMe
O
N
H
Br
3bg
abundance
X : parts per Million : 13C
171.505
171.324
152.312
137.295
131.974
121.429
116.681
83.424
77.475
77.160
76.836
57.919
52.332
37.391
29.611
28.429
28.086
25.854
25.177

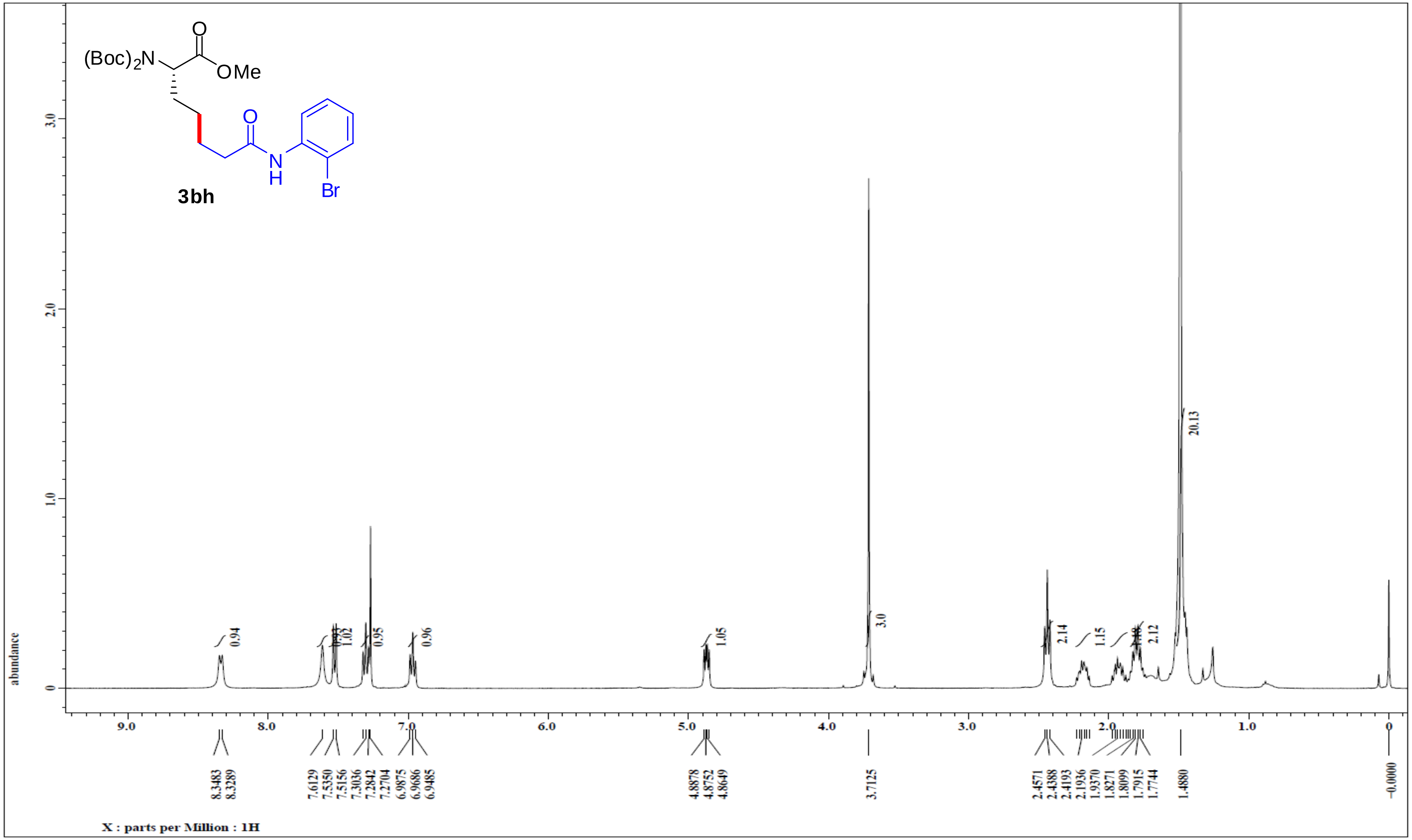

(Boc)2N
O
OMe
O
N
H
Br
3bh
abundance
3.0
2.0
1.0
0
0.94
1.02
0.95
0.96
1.05
3.0
2.14
1.15
2.12
20.13
9.0
8.0
7.0
6.0
5.0
4.0
3.0
2.0
1.0
0
8.3483
8.3289
7.6129
7.5350
7.5156
7.3036
7.2842
7.2704
6.9875
6.9686
6.9485
4.8878
4.8752
4.8649
3.7125
2.4571
2.4388
2.4193
2.1936
1.9370
1.8271
1.8099
1.7915
1.7744
1.4880
-0.0000
X : parts per Million : 1H

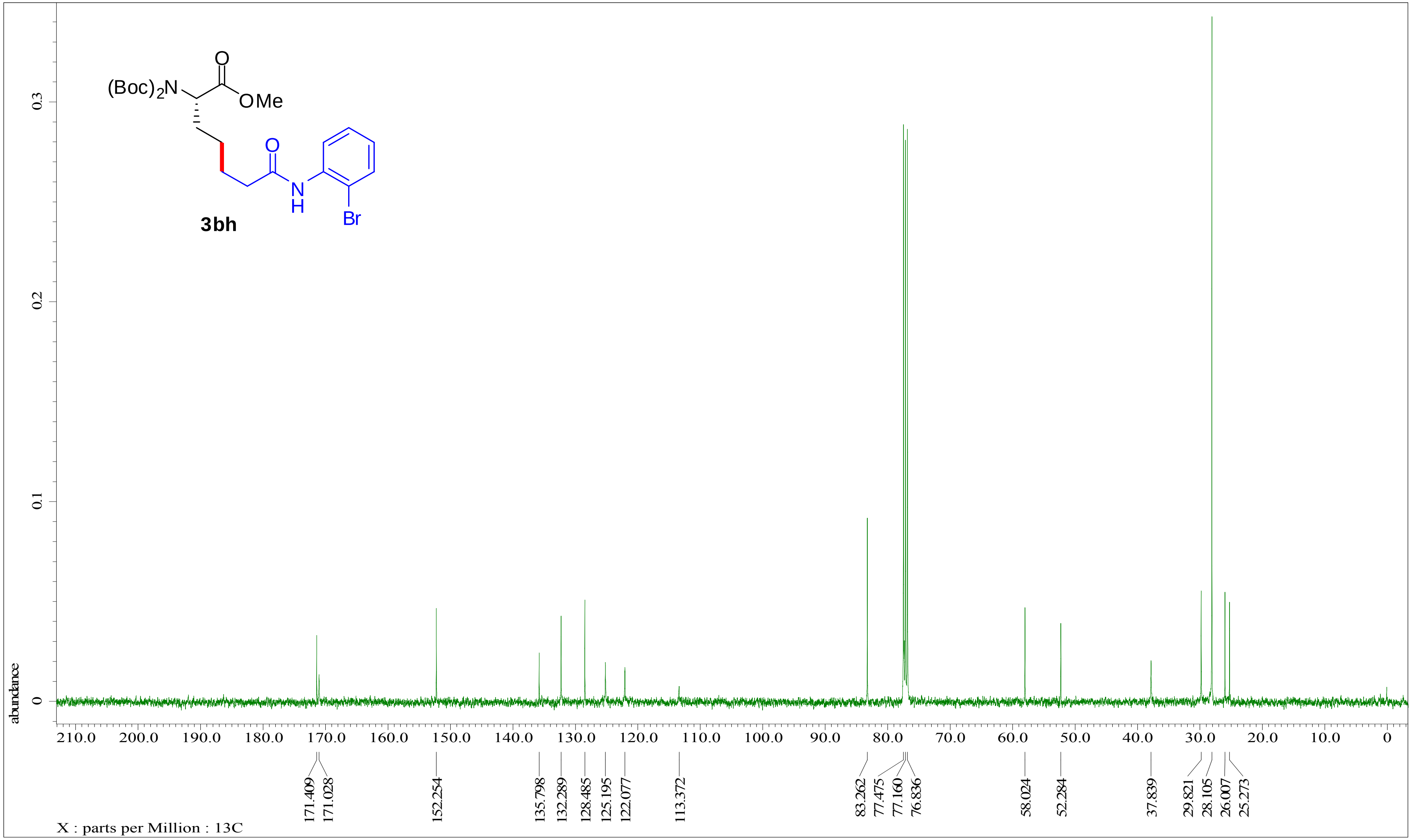
(Boc)2N
O
OMe
O
N
H
Br
3bh
abundance
0.3
0.2
0.1
0
210.0
200.0
190.0
180.0
170.0
160.0
150.0
140.0
130.0
120.0
110.0
100.0
90.0
80.0
70.0
60.0
50.0
40.0
30.0
20.0
10.0
0
171.409
171.028
152.254
135.798
132.289
128.485
125.195
122.077
113.372
83.262
77.475
77.160
76.836
58.024
52.284
37.839
29.821
28.105
26.007
25.273
X : parts per Million : 13C

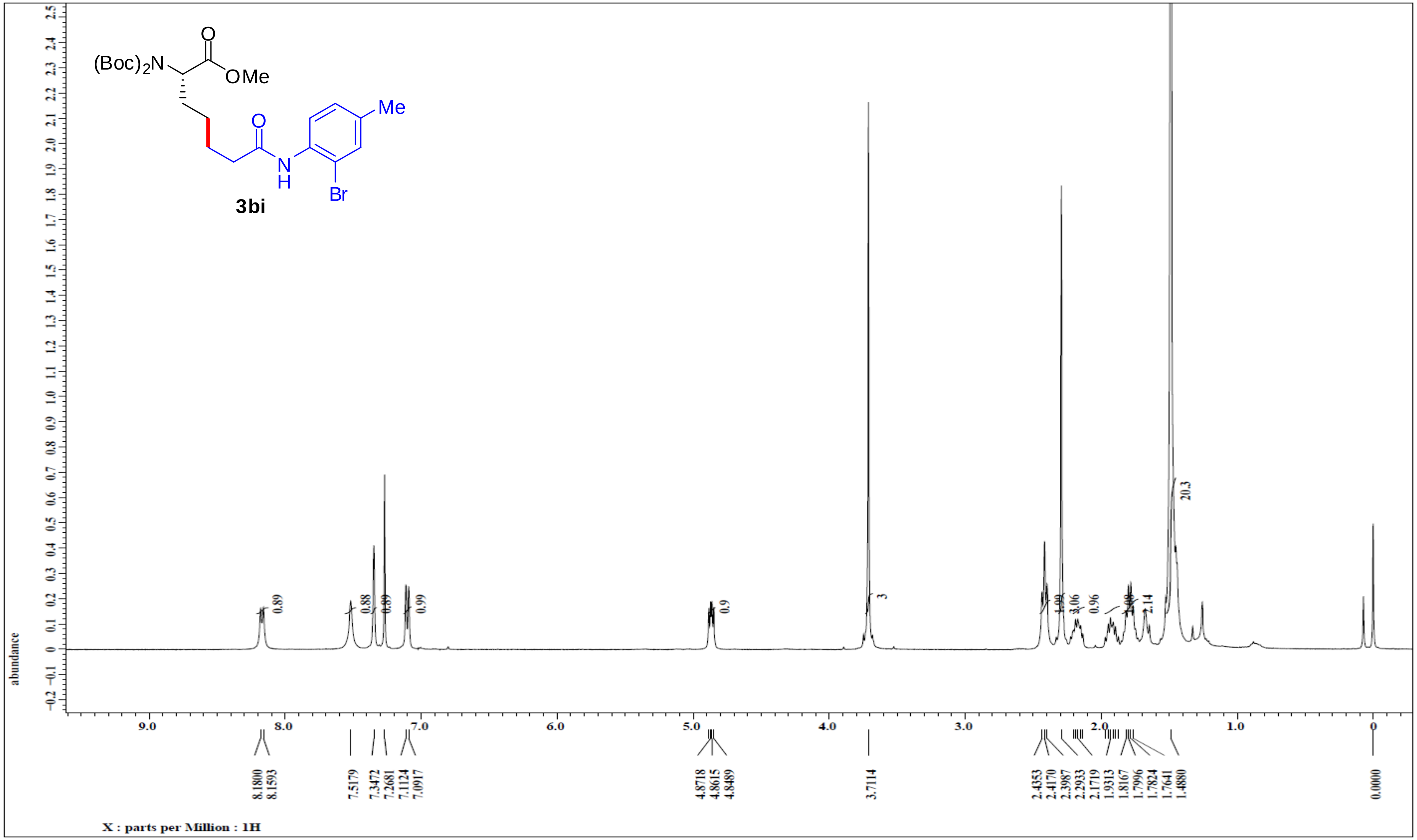
(Boc)2N
O
OMe
O
N
H
Me
Br
3bi
abundance
X : parts per Million : 1H
0.89
0.88
0.89
0.99
0.9
3
3.06
0.96
2.14
20.3
8.1800
8.1593
7.5179
7.3472
7.2681
7.1124
7.0917
4.8718
4.8615
4.8489
3.7114
2.4353
2.4170
2.3987
2.2933
2.1719
1.9313
1.8167
1.7996
1.7824
1.7641
1.4880
0.0000

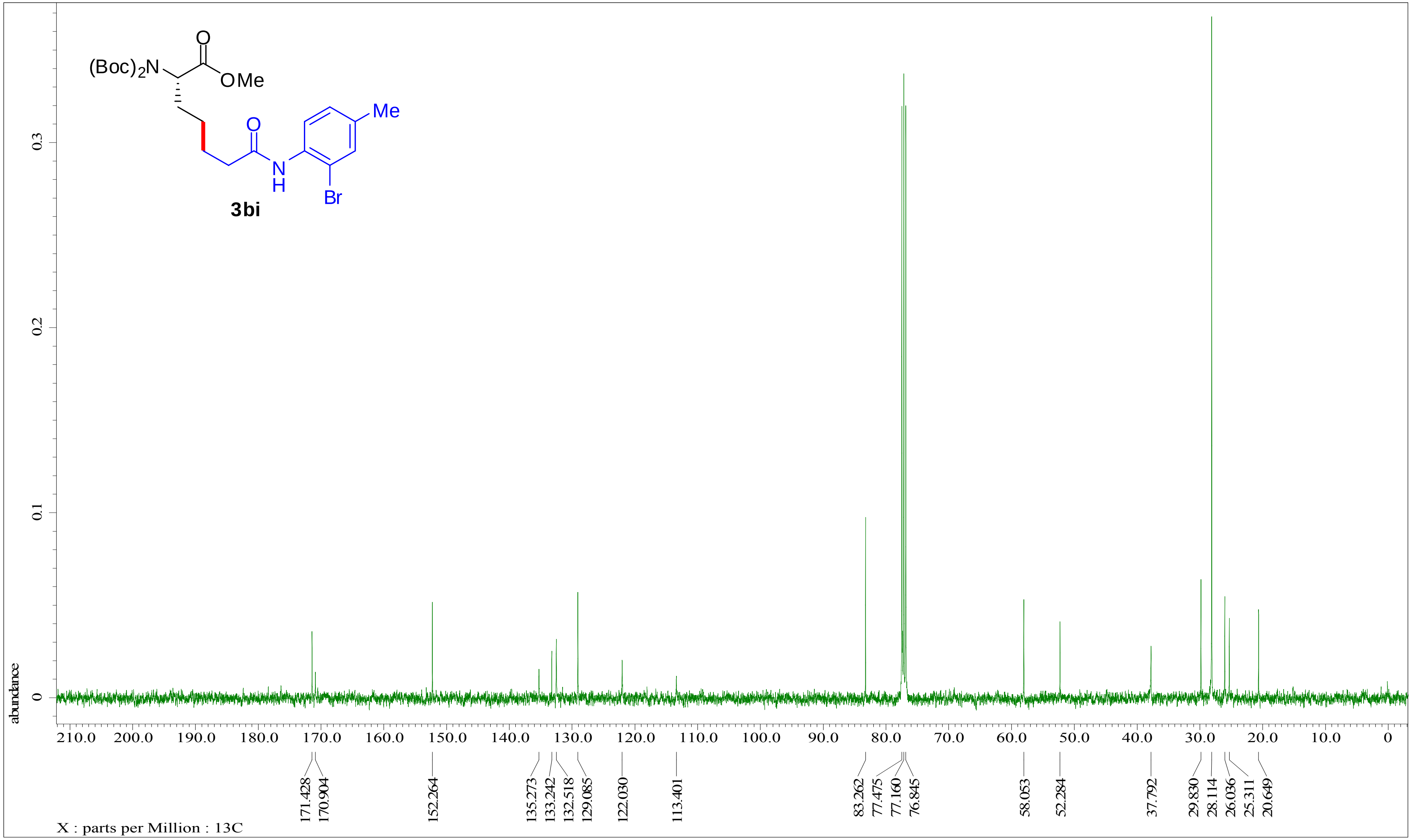
(Boc)2N
O
OMe
O
Me
N
H
Br
3bi
abundance
0.3
0.2
0.1
0
210.0
200.0
190.0
180.0
170.0
160.0
150.0
140.0
130.0
120.0
110.0
100.0
90.0
80.0
70.0
60.0
50.0
40.0
30.0
20.0
10.0
0
171.428
170.904
152.264
135.273
133.242
132.518
129.085
122.030
113.401
83.262
77.475
77.160
76.845
58.053
52.284
37.792
29.830
28.114
26.036
25.311
20.649
X : parts per Million : 13C

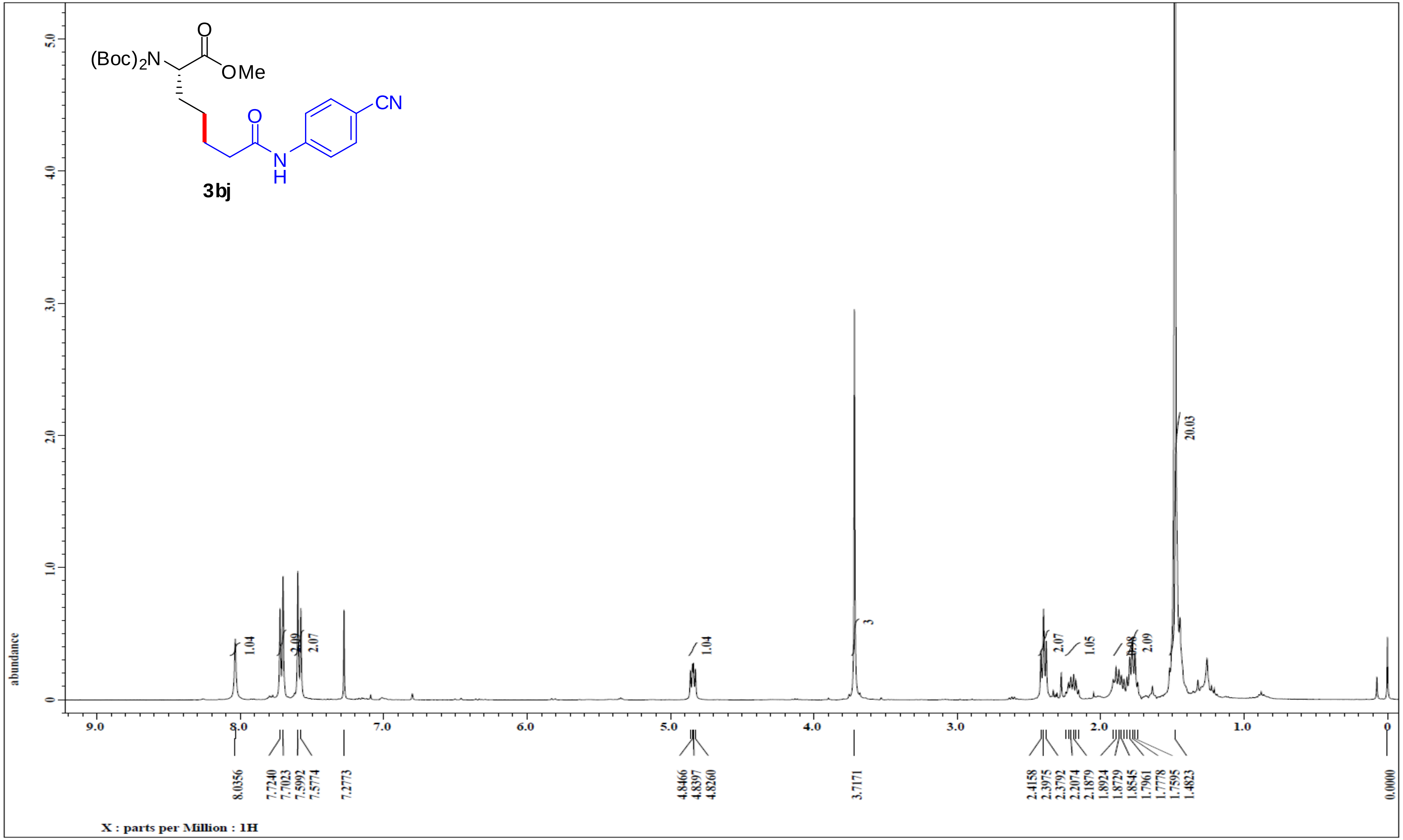

(Boc)2N
O
OMe
O
CN
N
H
3bj
abundance
X : parts per Million : 1H
1.04
2.09
2.07
1.04
3
2.07
1.05
2.08
2.09
20.03
8.0356
7.7240
7.7023
7.5992
7.5774
7.2773
4.8466
4.8397
4.8260
3.7171
2.4158
2.3975
2.3792
2.2074
2.1879
1.8924
1.8729
1.8545
1.7961
1.7778
1.7595
1.4823
0.0000

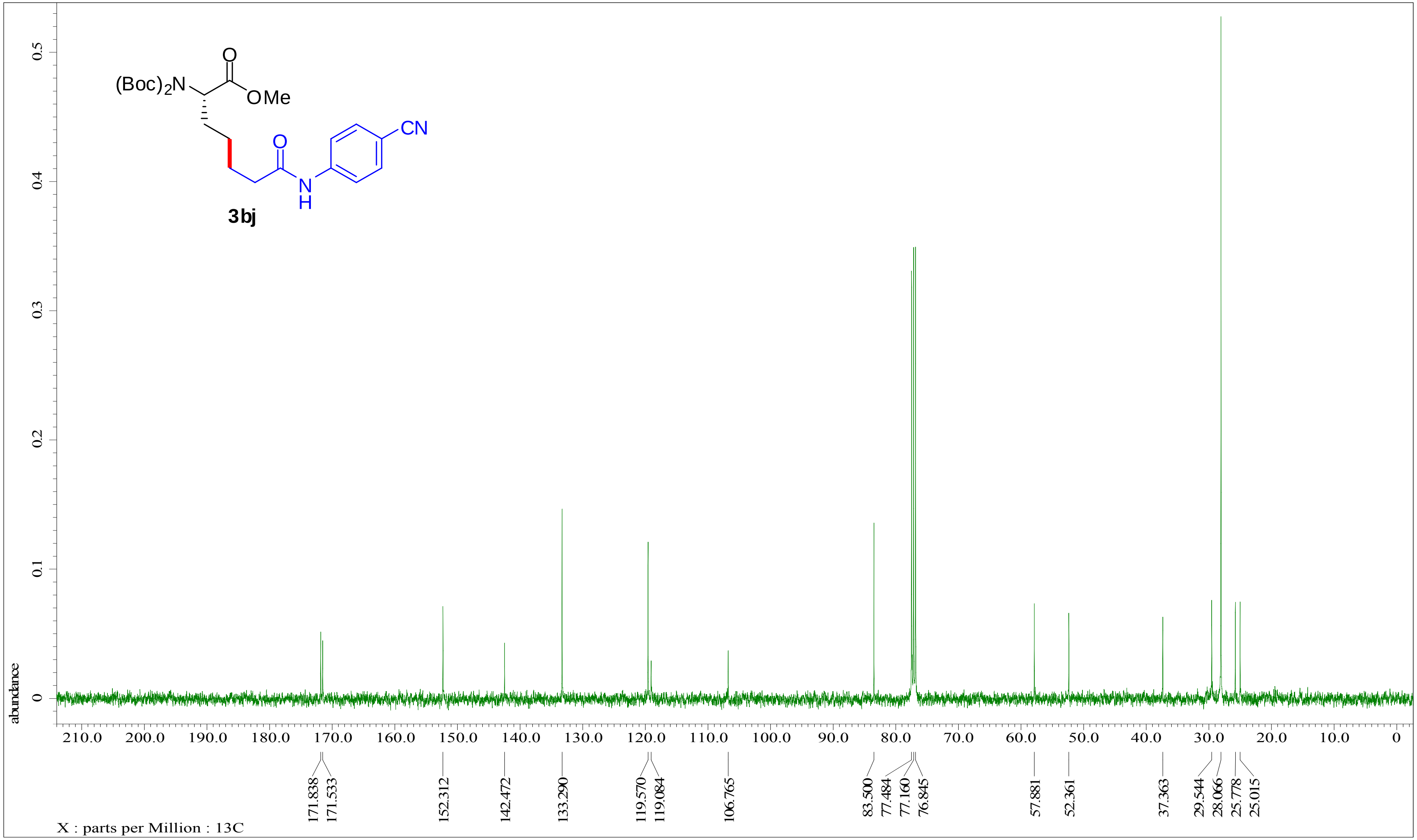
(Boc)2N
O
OMe
O
N
H
CN
3bj
abundance
0
0.1
0.2
0.3
0.4
0.5
210.0
200.0
190.0
180.0
170.0
160.0
150.0
140.0
130.0
120.0
110.0
100.0
90.0
80.0
70.0
60.0
50.0
40.0
30.0
20.0
10.0
0
171.838
171.533
152.312
142.472
133.290
119.570
119.084
106.765
83.500
77.484
77.160
76.845
57.881
52.361
37.363
29.544
28.066
25.778
25.015
X : parts per Million : 13C

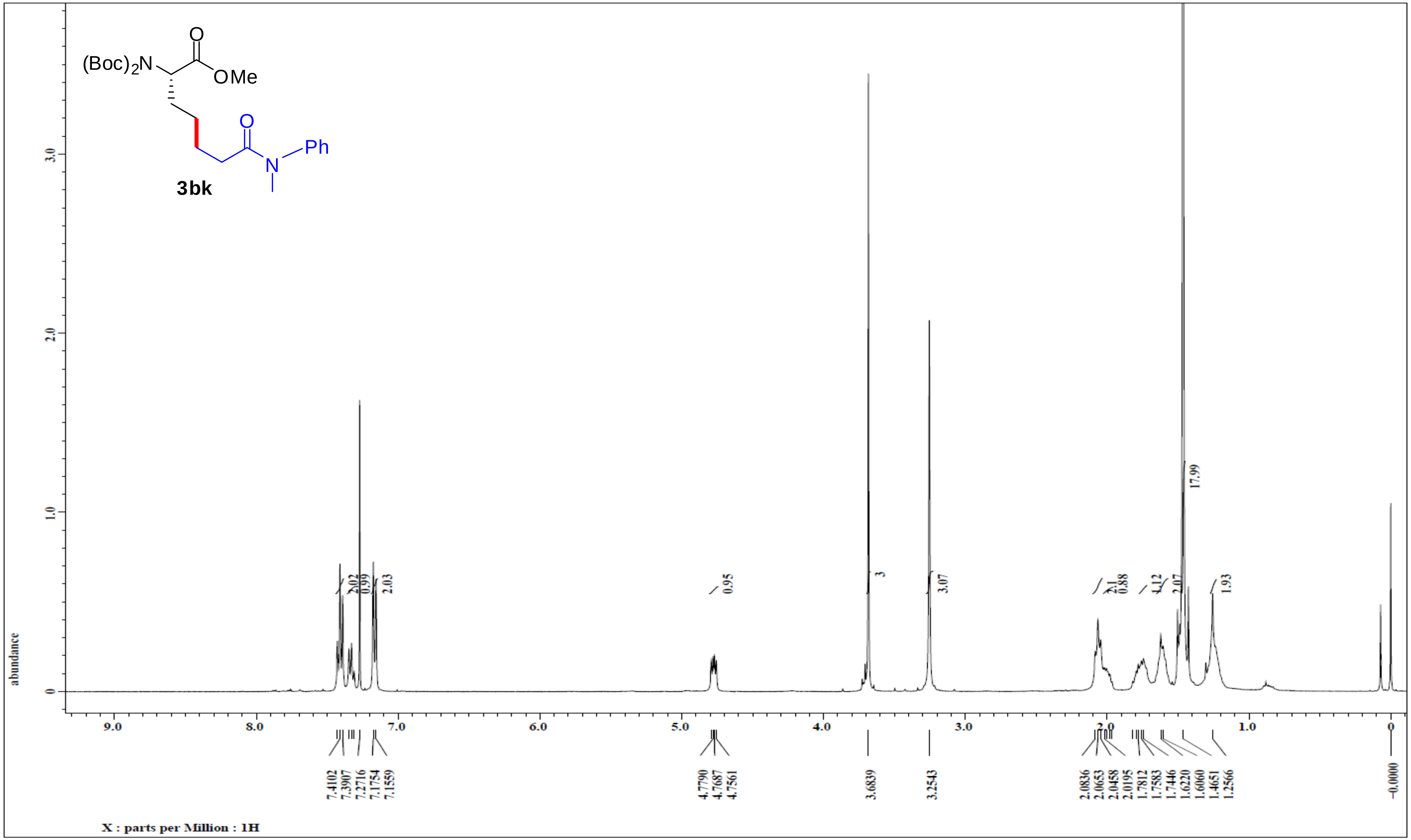
(Boc)2N
OMe
O
O
N
Ph
3bk
abundance
X : parts per Million : 1H
7.4102
7.3907
7.2716
7.1754
7.1559
4.7790
4.7687
4.7561
3.6839
3.2543
2.0836
2.0653
2.0458
2.0195
1.7812
1.7583
1.7446
1.6220
1.6060
1.4651
1.2566
-0.0000
2.02
0.99
2.03
0.95
3
3.07
2.1
0.88
1.12
2.07
17.99
1.93

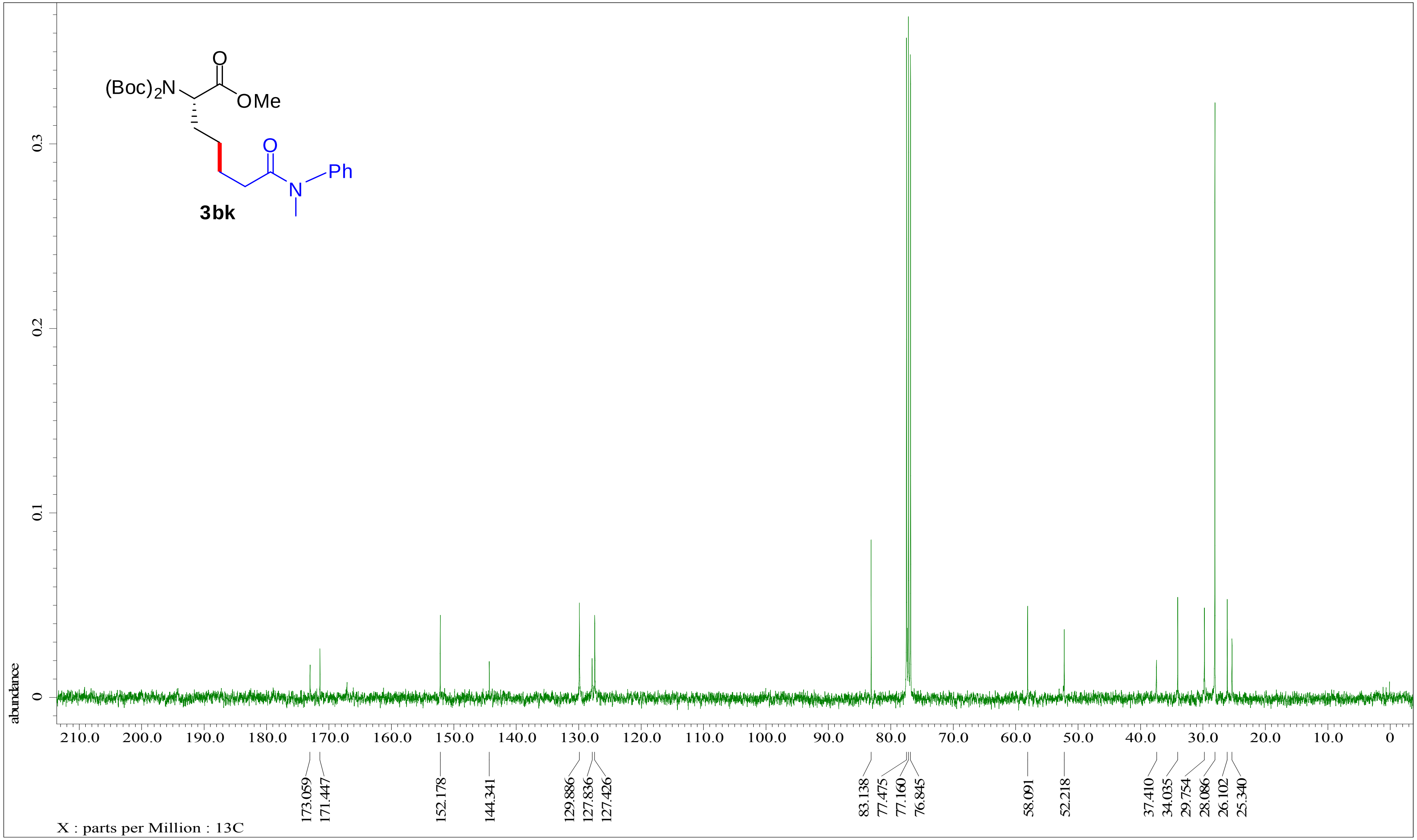
(Boc)2N
O
OMe
O
N
Ph
3bk
abundance
0
0.1
0.2
0.3
210.0
200.0
190.0
180.0
170.0
160.0
150.0
140.0
130.0
120.0
110.0
100.0
90.0
80.0
70.0
60.0
50.0
40.0
30.0
20.0
10.0
0
173.059
171.447
152.178
144.341
129.886
127.836
127.426
83.138
77.475
77.160
76.845
58.091
52.218
37.410
34.035
29.754
28.086
26.102
25.340
X : parts per Million : 13C

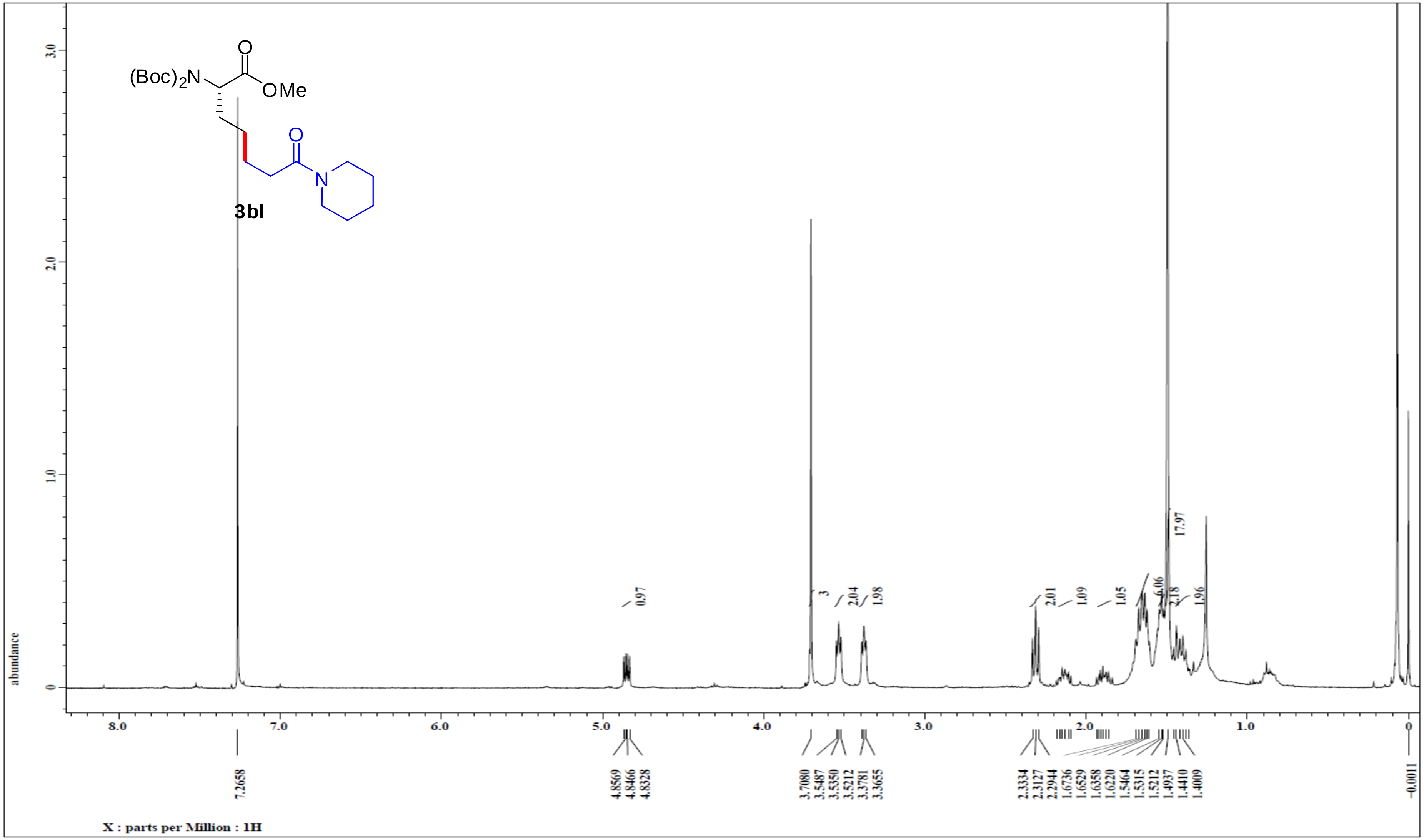
(Boc)$_2$N
OMe
O
N
3bl
abundance
X : parts per Million : 1H
0.97
3
2.04
1.98
2.01
1.09
1.05
6.06
17.97
2.18
1.96
7.2658
4.8569
4.8466
4.8328
3.7080
3.5487
3.5350
3.5212
3.3781
3.3655
2.3334
2.3127
2.2944
1.6736
1.6529
1.6358
1.6220
1.5464
1.5315
1.5212
1.4937
1.4410
1.4009
-0.0011

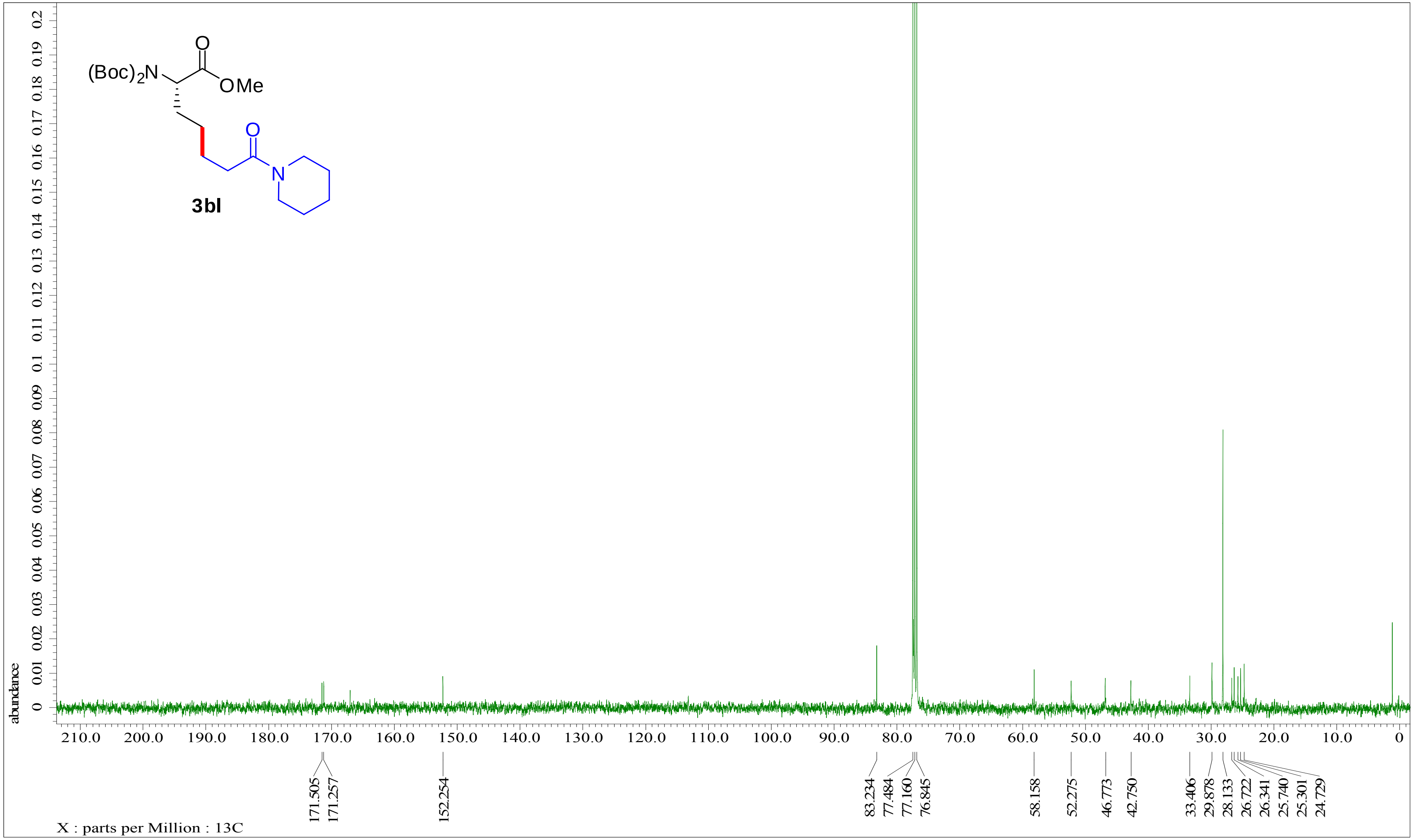
(Boc)2N
OMe
3bl
abundance
X : parts per Million : 13C
171.505
171.257
152.254
83.234
77.484
77.160
76.845
58.158
52.275
46.773
42.750
33.406
29.878
28.133
26.722
26.341
25.740
25.301
24.729

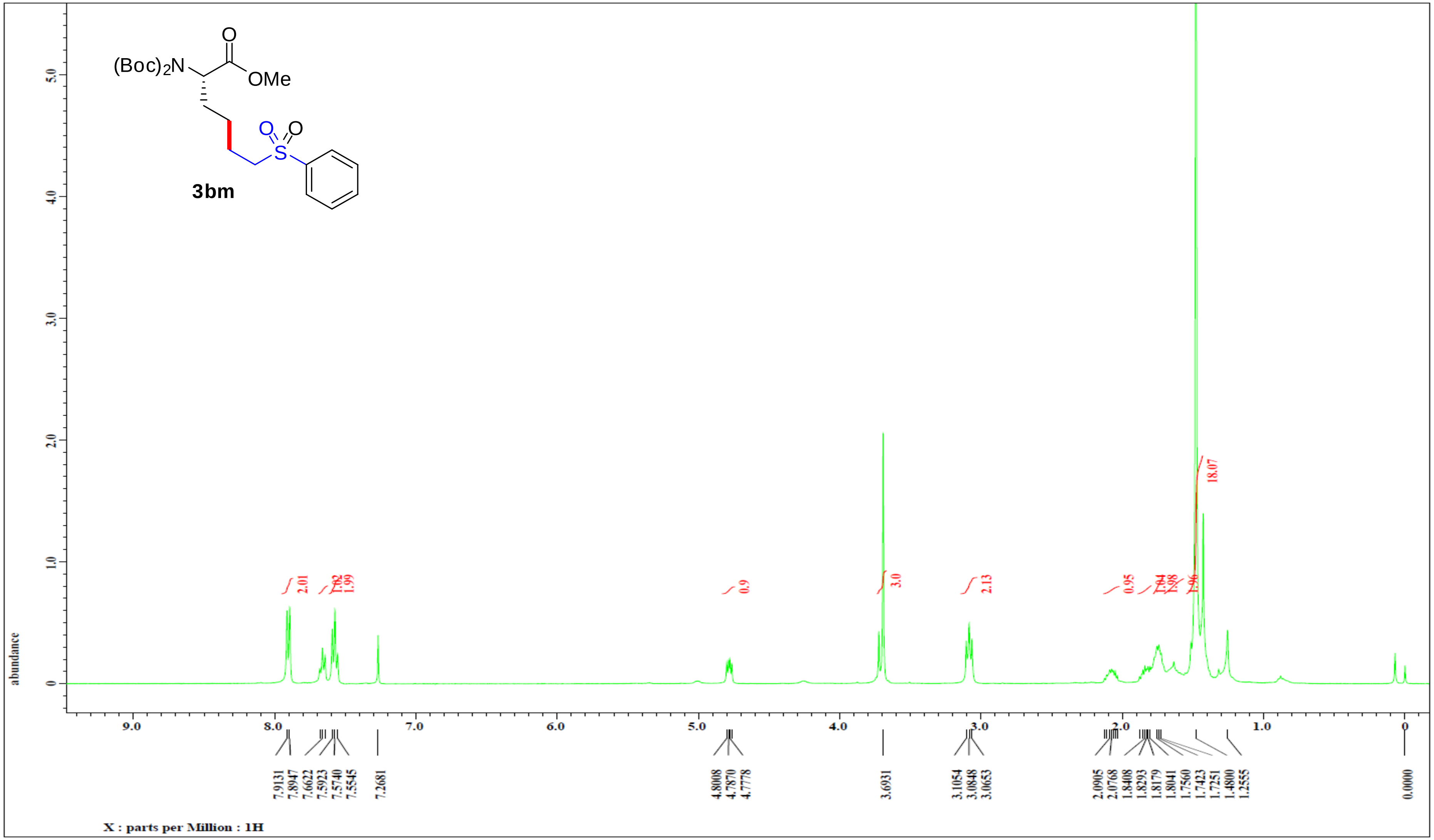
(Boc)2N
O
OMe
O
O
S
3bm
abundance
X : parts per Million : 1H
2.01
1.99
0.9
3.0
2.13
0.95
18.07
7.9131
7.8947
7.6622
7.5923
7.5740
7.5545
7.2681
4.8008
4.7870
4.7778
3.6931
3.1054
3.0848
3.0653
2.0905
2.0768
1.8408
1.8293
1.8179
1.8041
1.7560
1.7423
1.7251
1.4800
1.2555
0.0000

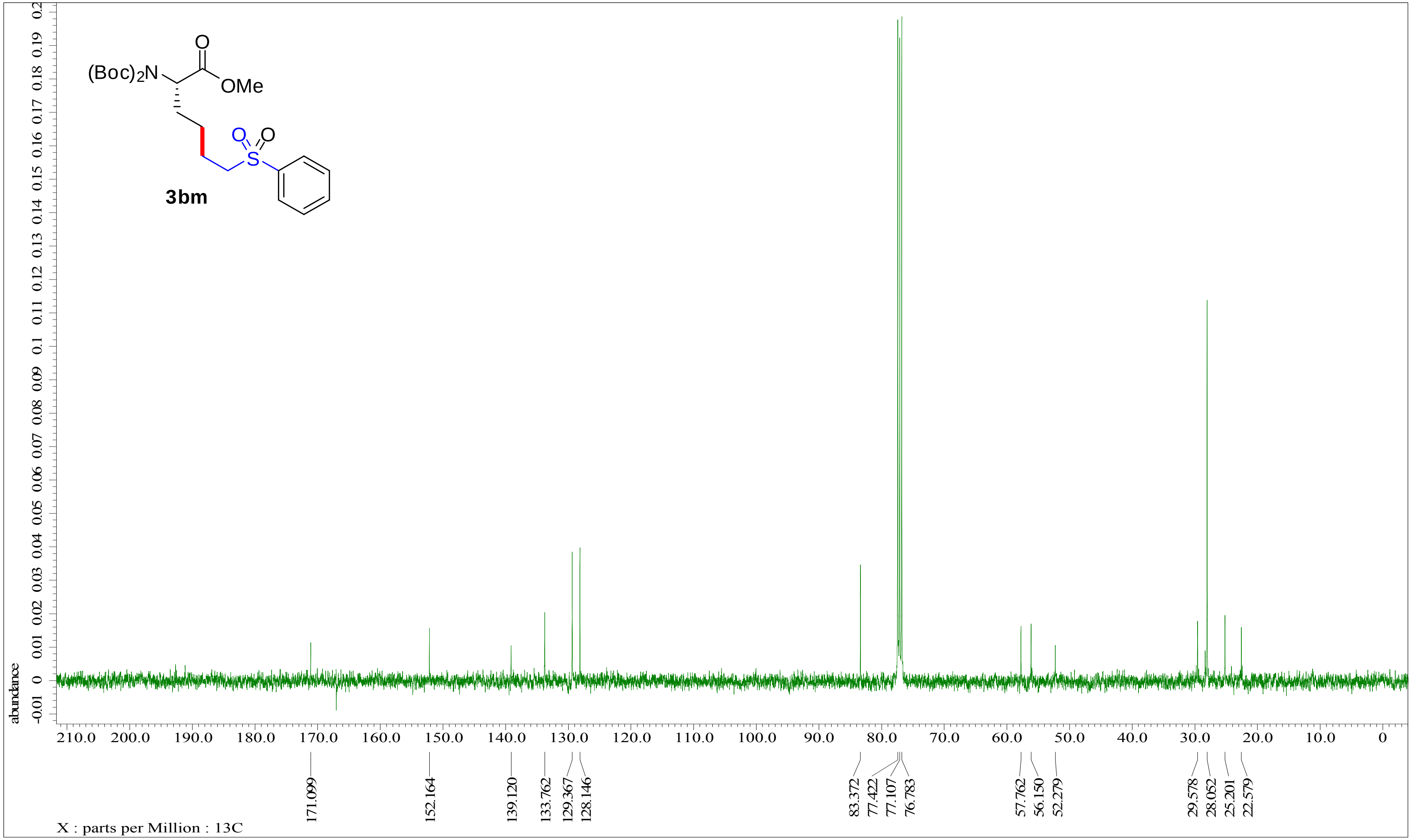
(Boc)2N
OMe
3bm
abundance
X : parts per Million : 13C
171.099
152.164
139.120
133.762
129.367
128.146
83.372
77.422
77.107
76.783
57.762
56.150
52.279
29.578
28.052
25.201
22.579

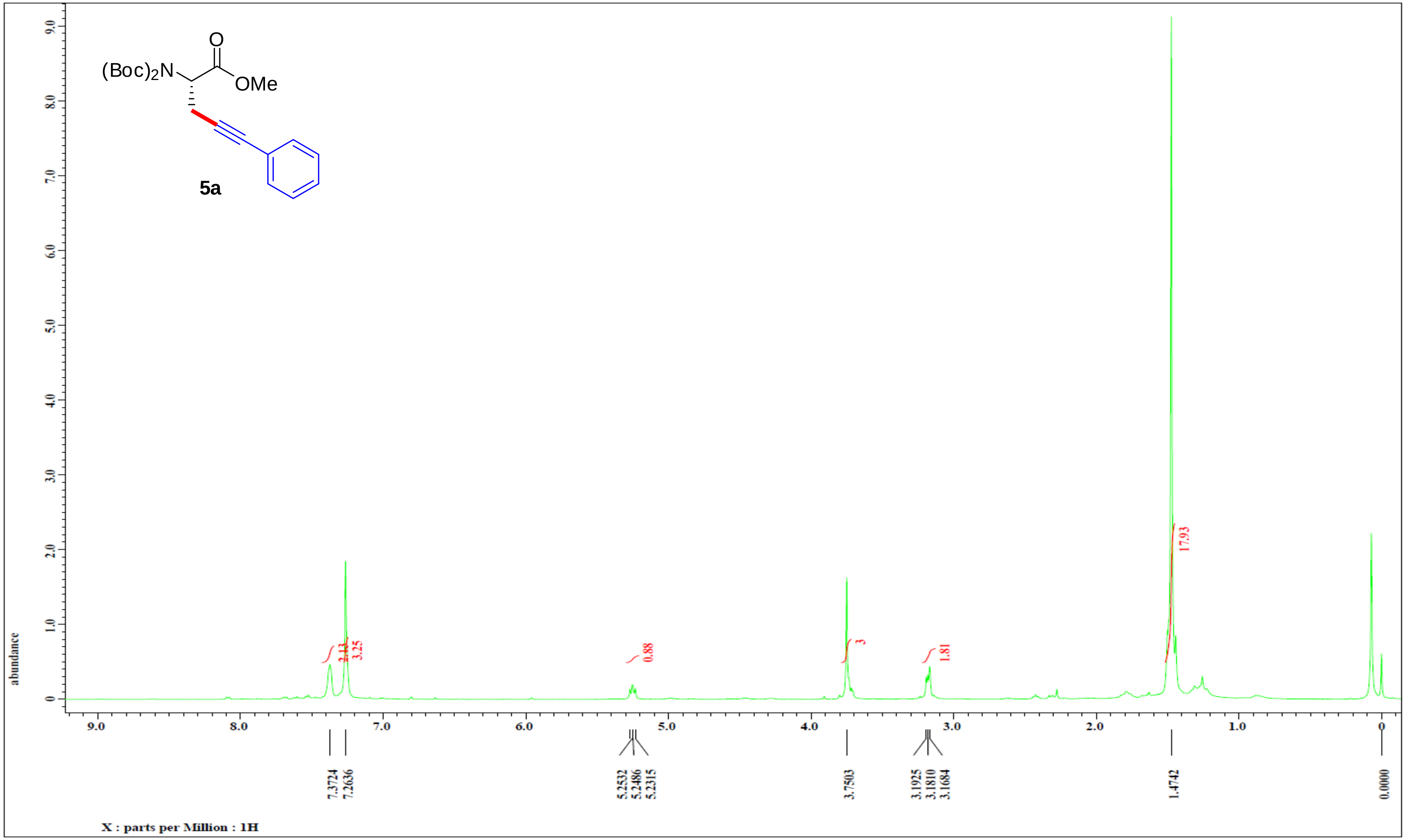
(Boc)2N
O
OMe
5a
abundance
X : parts per Million : 1H
7.3724
7.2636
5.2532
5.2486
5.2315
3.7503
3.1925
3.1810
3.1684
1.4742
0.0000
2.13
3.25
0.88
3
1.81
17.93

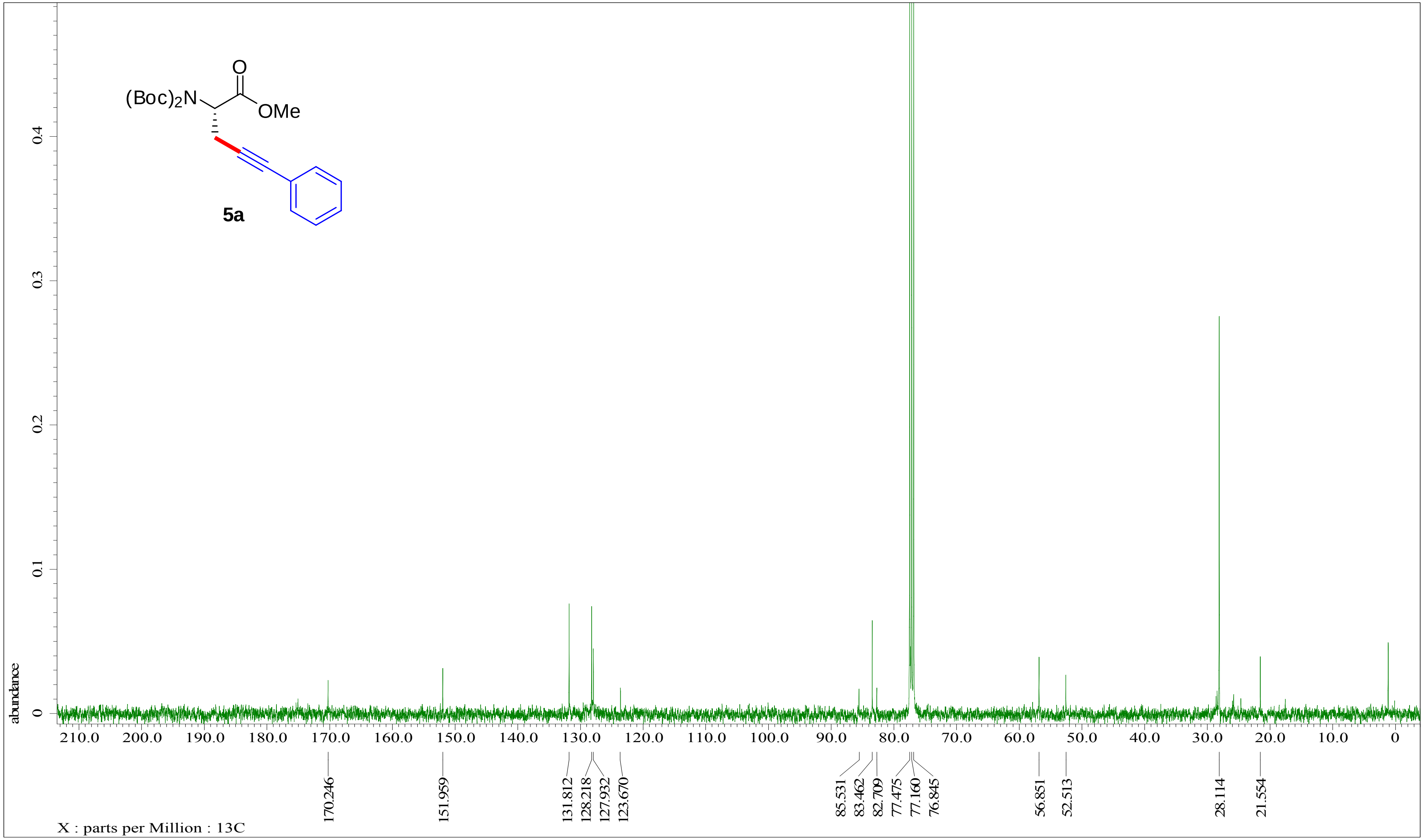
(Boc)2N
O
OMe
5a
abundance
0
0.1
0.2
0.3
0.4
210.0
200.0
190.0
180.0
170.0
160.0
150.0
140.0
130.0
120.0
110.0
100.0
90.0
80.0
70.0
60.0
50.0
40.0
30.0
20.0
10.0
0
170.246
151.959
131.812
128.218
127.932
123.670
85.531
83.462
82.709
77.475
77.160
76.845
56.851
52.513
28.114
21.554
X : parts per Million : 13C

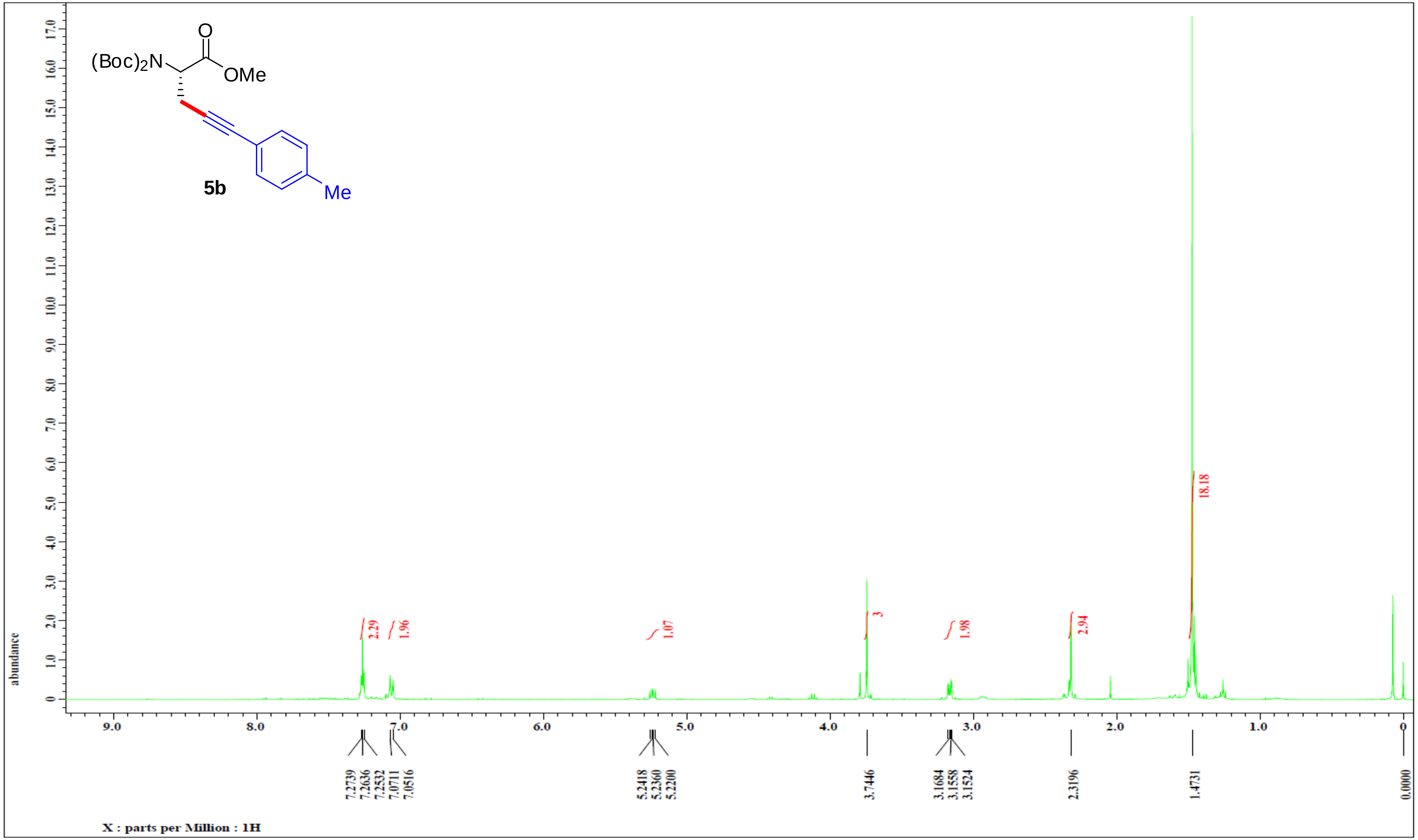

(Boc)2N
O
OMe
Me
5b
abundance
X : parts per Million : 1H
7.2739
7.2636
7.2532
7.0711
7.0516
5.2418
5.2360
5.2200
3.7446
3.1684
3.1558
3.1524
2.3196
1.4731
0.0000
2.29
1.96
1.07
3
1.98
2.94
18.18

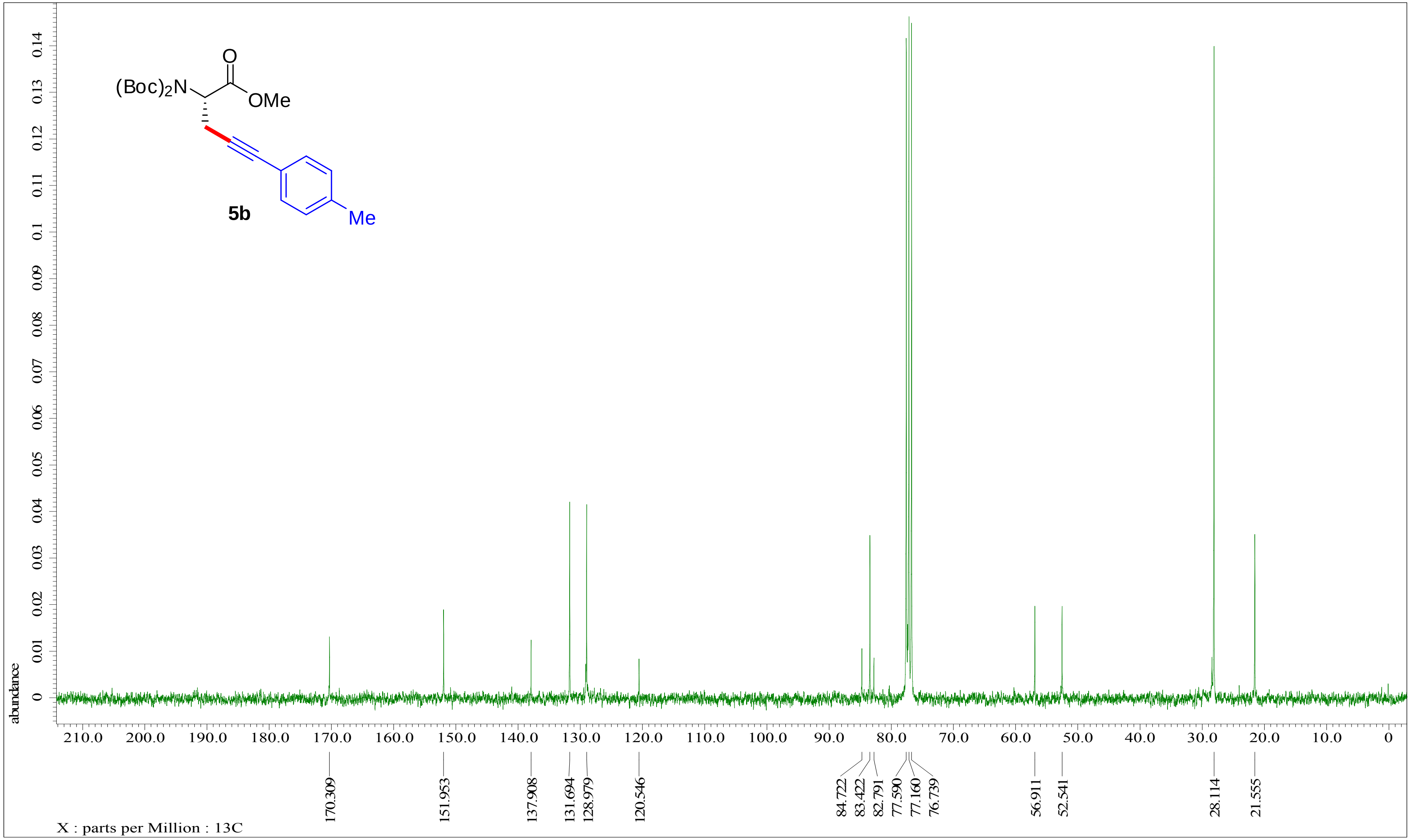
(Boc)2N
O
OMe
5b
Me
abundance
X : parts per Million : 13C
170.309
151.953
137.908
131.694
128.979
120.546
84.722
83.422
82.791
77.590
77.160
76.739
56.911
52.541
28.114
21.555

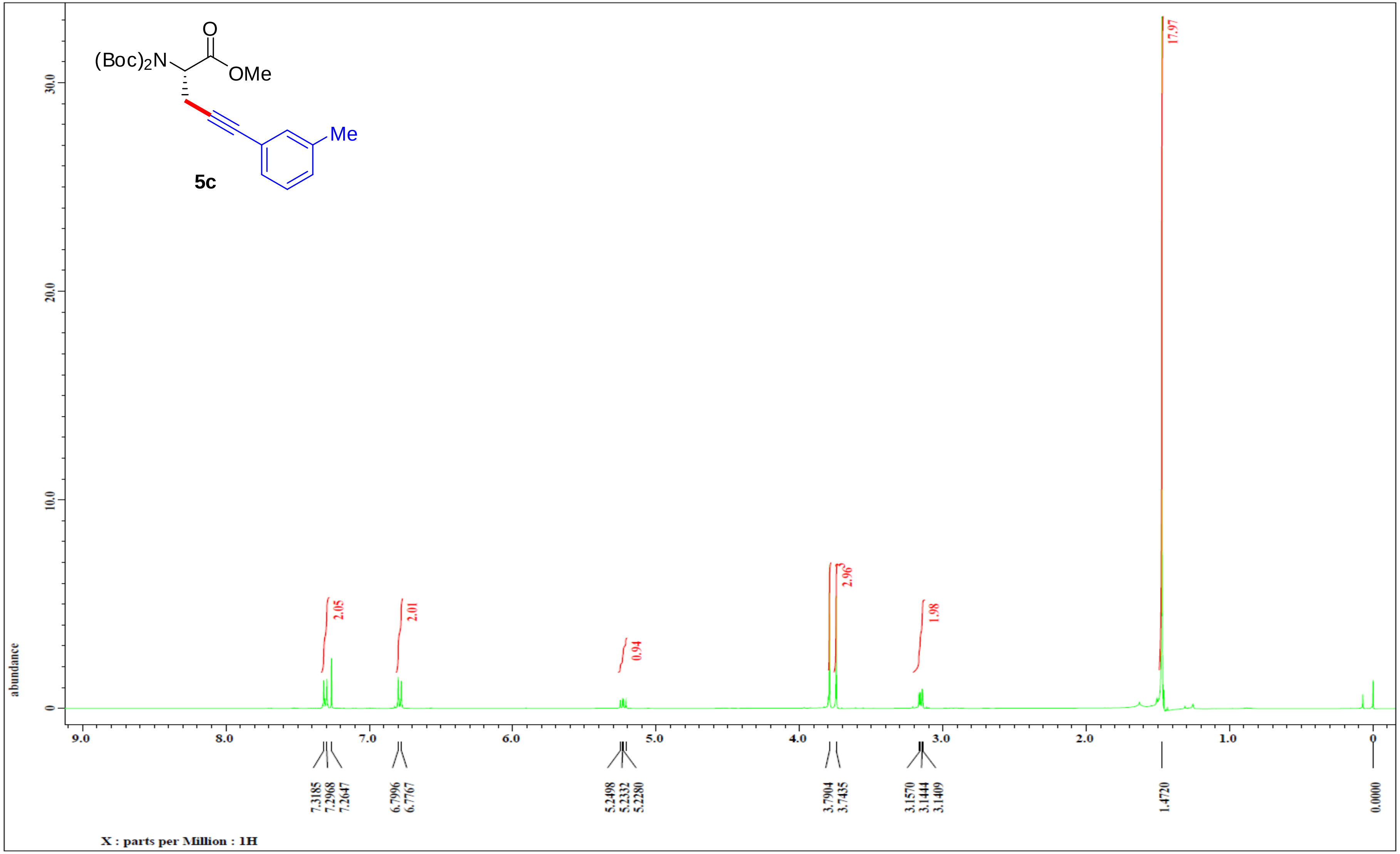
(Boc)2N
O
OMe
Me
5c
30.0
20.0
10.0
0
abundance
17.97
2.05
2.01
0.94
2.96
1.98
9.0
8.0
7.0
6.0
5.0
4.0
3.0
2.0
1.0
0
7.3185
7.2968
7.2647
6.7996
6.7767
5.2498
5.2332
5.2280
3.7904
3.7435
3.1570
3.1444
3.1409
1.4720
0.0000
X : parts per Million : 1H

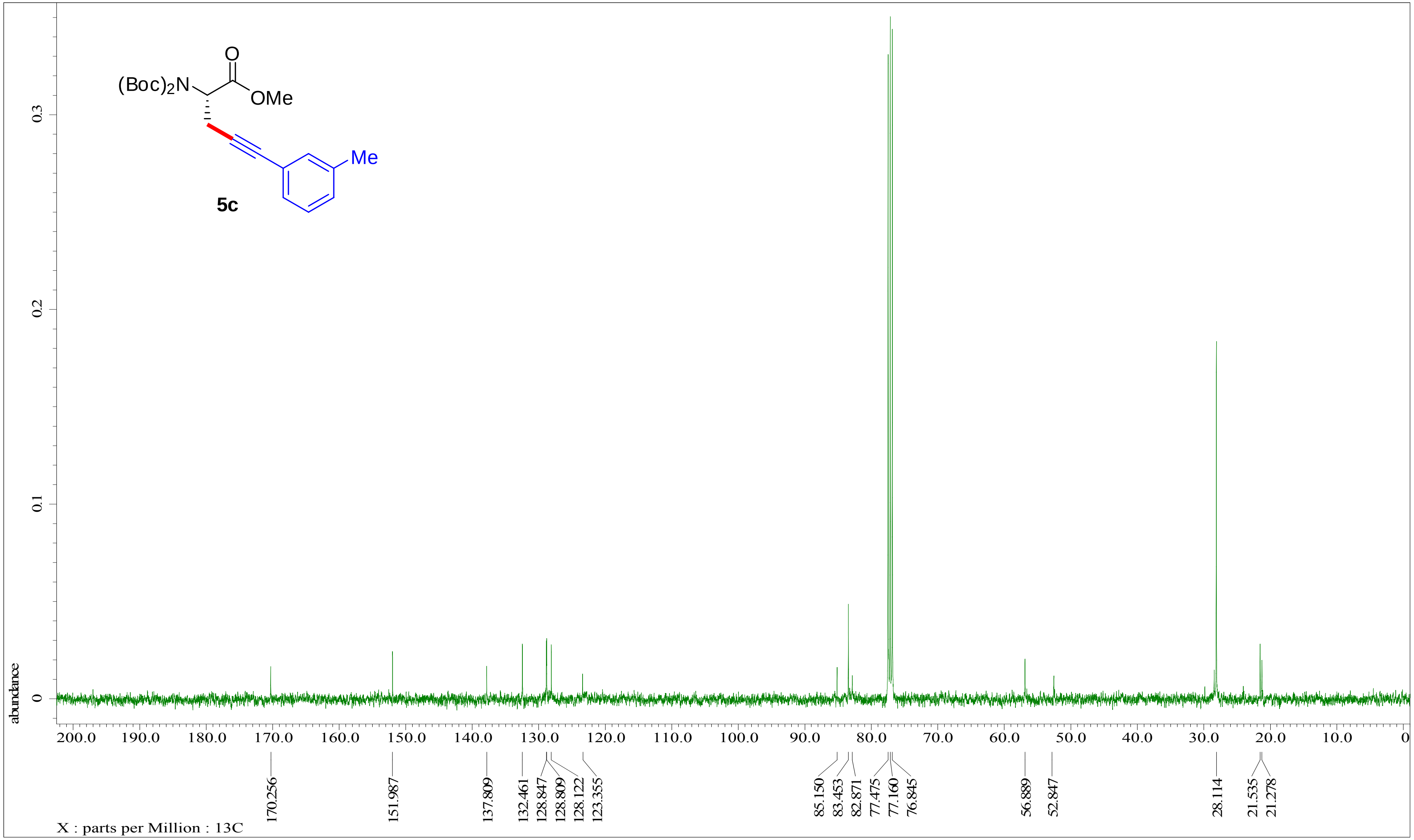
(Boc)2N
OMe
Me
5c
abundance
0.3
0.2
0.1
0
200.0 190.0 180.0 170.0 160.0 150.0 140.0 130.0 120.0 110.0 100.0 90.0 80.0 70.0 60.0 50.0 40.0 30.0 20.0 10.0 0
170.256
151.987
137.809
132.461
128.847
128.809
128.122
123.355
85.150
83.453
82.871
77.475
77.160
76.845
56.889
52.847
28.114
21.535
21.278
X : parts per Million : 13C

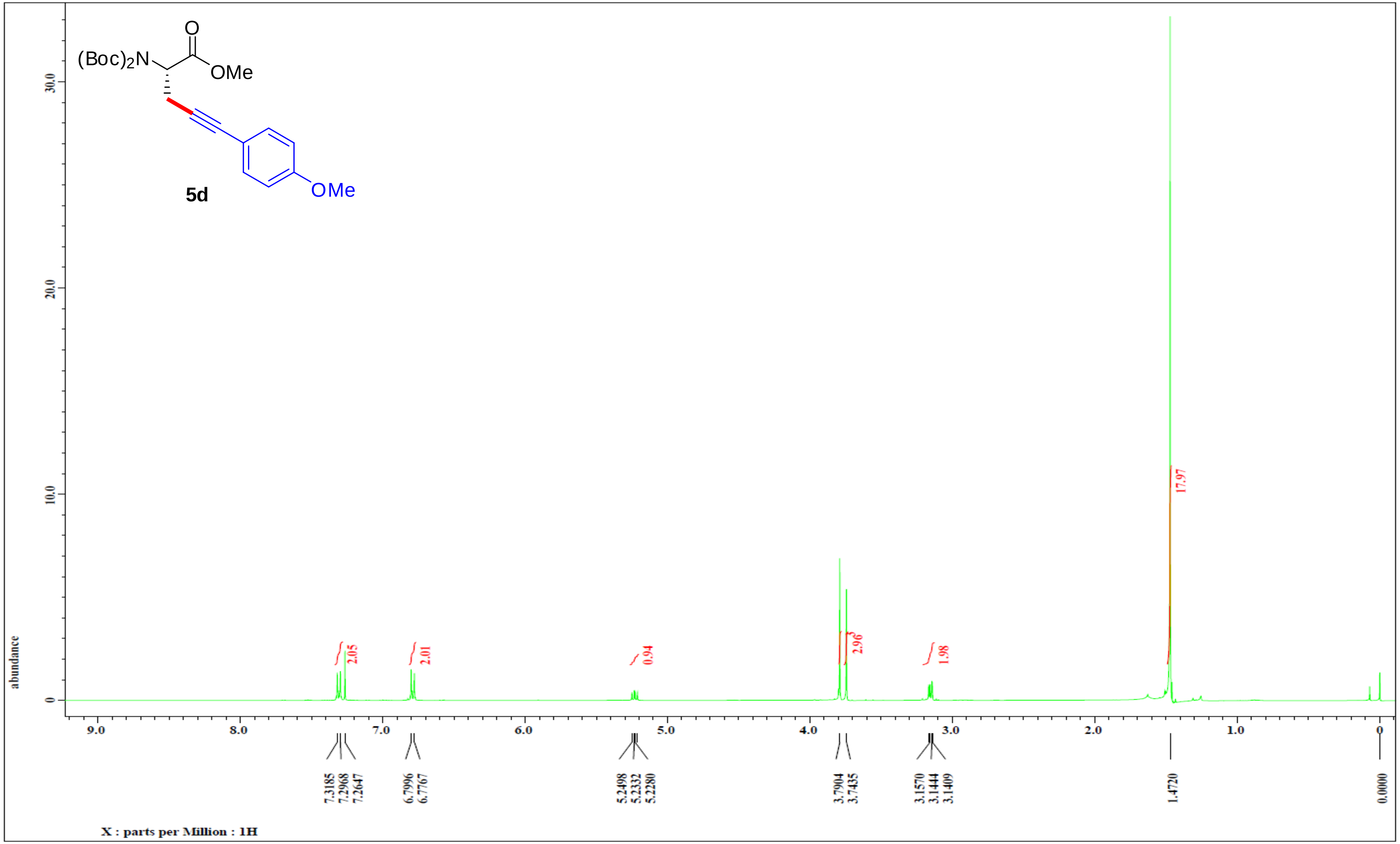
(Boc)2N
O
OMe
OMe
5d
30.0
20.0
10.0
0
abundance
2.05
2.01
0.94
2.96
1.98
17.97
9.0
8.0
7.0
6.0
5.0
4.0
3.0
2.0
1.0
0
7.3185
7.2968
7.2647
6.7996
6.7767
5.2498
5.2332
5.2280
3.7904
3.7435
3.1570
3.1444
3.1409
1.4720
0.0000
X : parts per Million : 1H

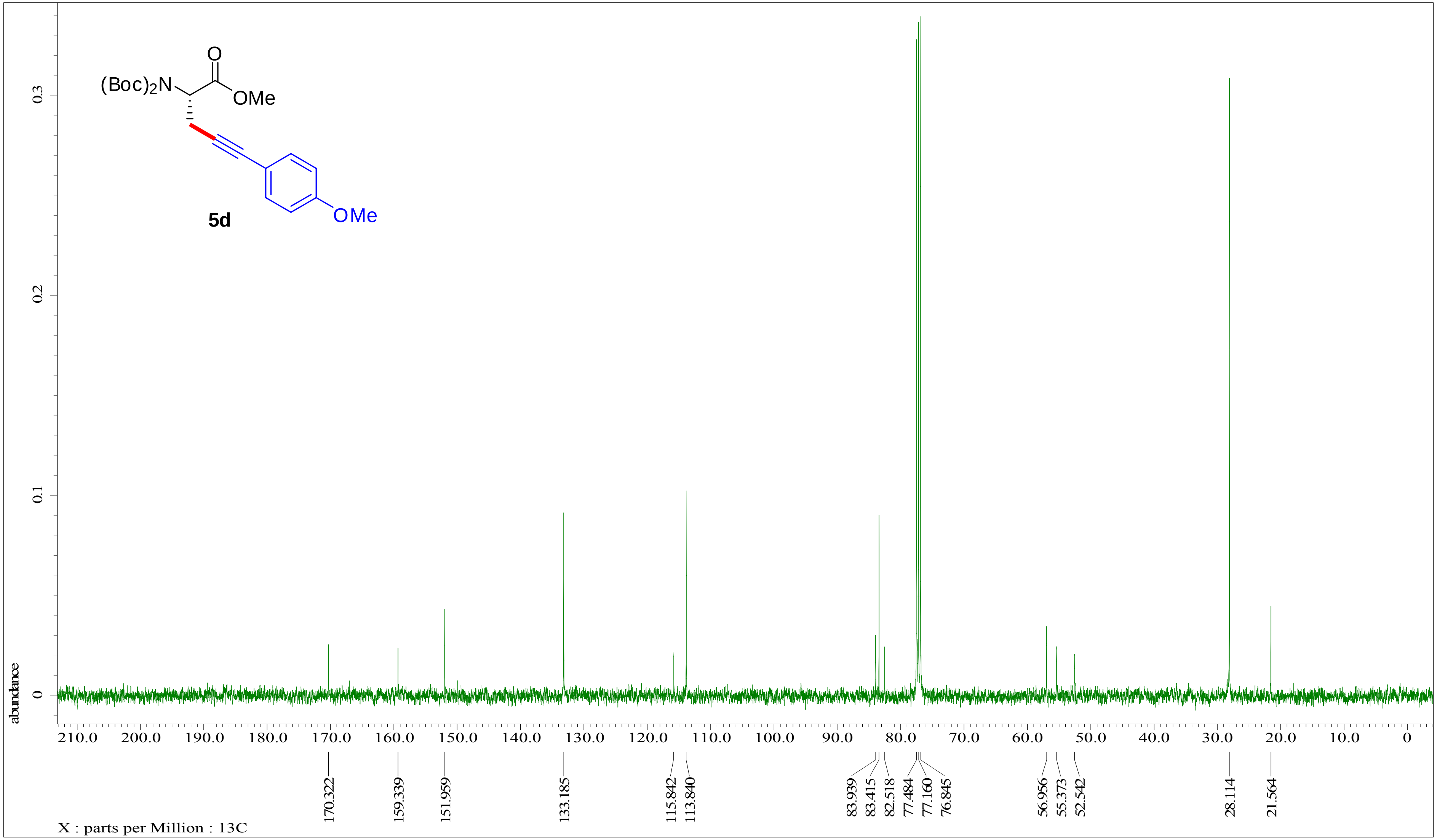
(Boc)2N
O
OMe
5d
OMe
abundance
0.3
0.2
0.1
0
210.0
200.0
190.0
180.0
170.0
160.0
150.0
140.0
130.0
120.0
110.0
100.0
90.0
80.0
70.0
60.0
50.0
40.0
30.0
20.0
10.0
0
170.322
159.339
151.959
133.185
115.842
113.840
83.939
83.415
82.518
77.484
77.160
76.845
56.956
55.373
52.542
28.114
21.564
X : parts per Million : 13C

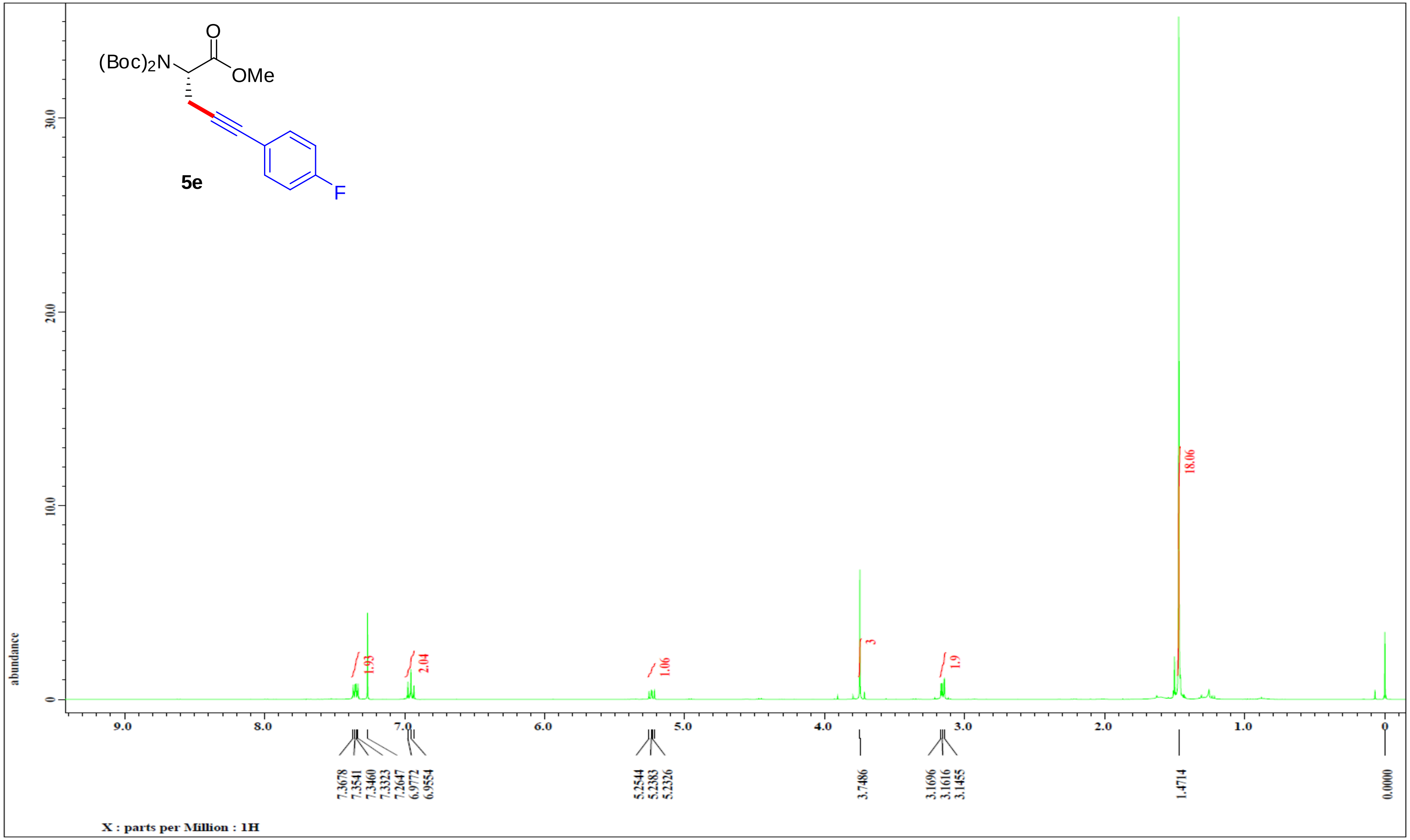

(Boc)2N
O
OMe
F
5e
abundance
30.0
20.0
10.0
0
9.0
8.0
7.0
6.0
5.0
4.0
3.0
2.0
1.0
0
1.93
2.04
1.06
3
1.9
18.06
7.3678
7.3541
7.3460
7.3323
7.2647
6.9772
6.9554
5.2544
5.2383
5.2326
3.7486
3.1696
3.1616
3.1455
1.4714
0.0000
X : parts per Million : 1H

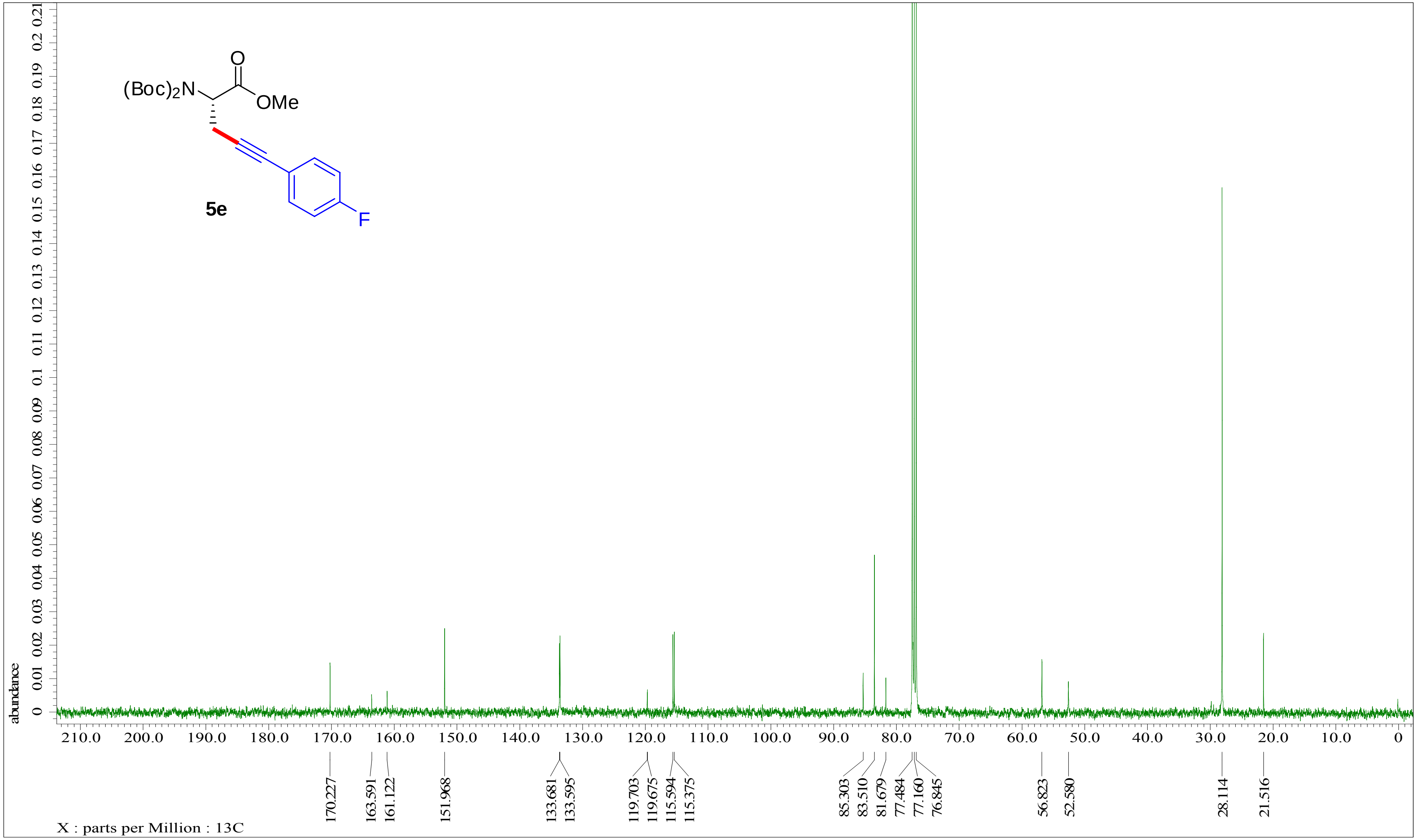
(Boc)2N
O
OMe
F
5e
abundance
X : parts per Million : 13C
170.227
163.591
161.122
151.968
133.681
133.595
119.703
119.675
115.594
115.375
85.303
83.510
81.679
77.484
77.160
76.845
56.823
52.580
28.114
21.516

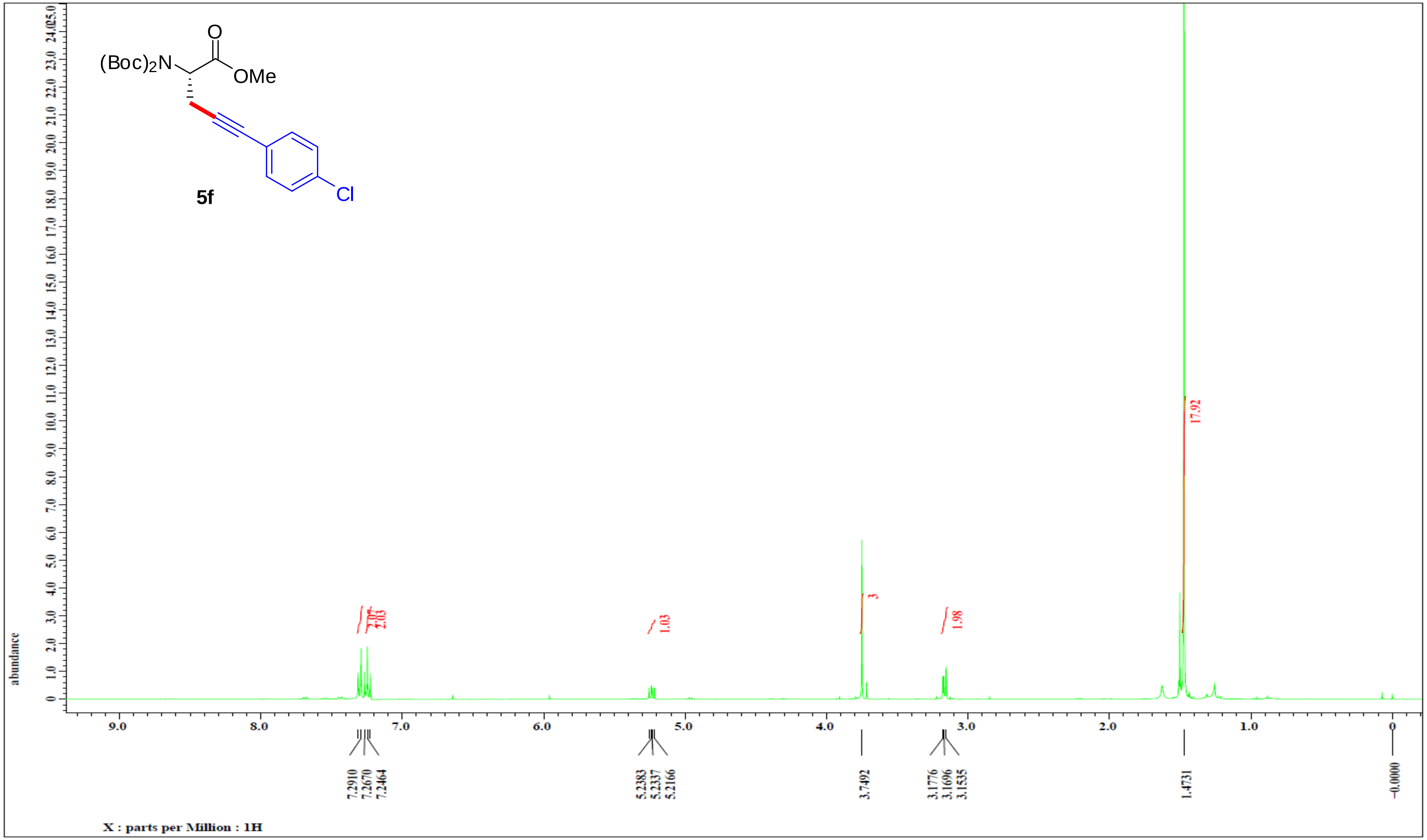
(Boc)2N
O
OMe
Cl
5f
abundance
X : parts per Million : 1H
7.2910
7.2670
7.2464
5.2383
5.2337
5.2166
3.7492
3.1776
3.1696
3.1535
1.4731
-0.0000
1.03
3
1.98
17.92

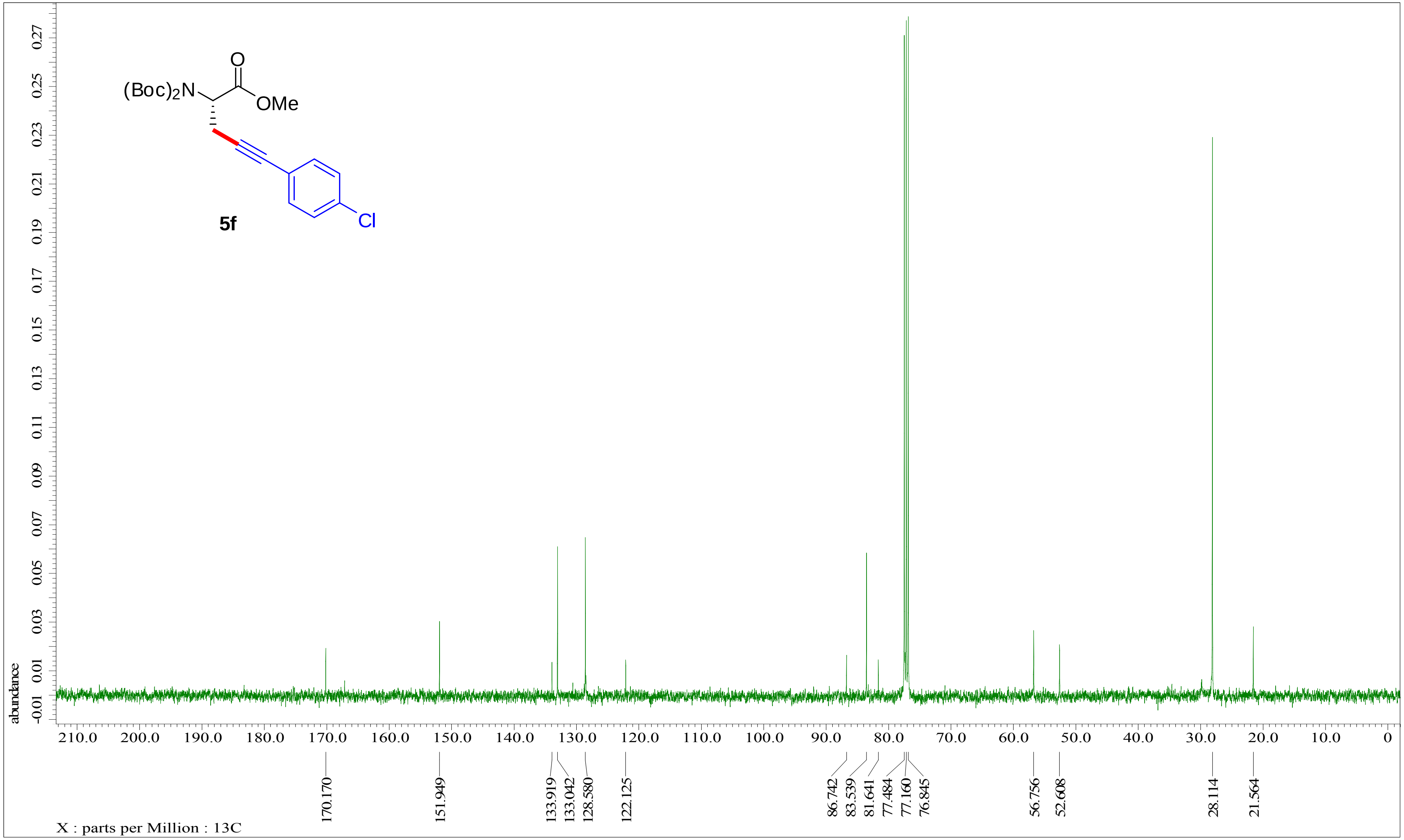

(Boc)2N
O
OMe
5f
Cl
abundance
0.27
0.25
0.23
0.21
0.19
0.17
0.15
0.13
0.11
0.09
0.07
0.05
0.03
0.01
-0.01
210.0 200.0 190.0 180.0 170.0 160.0 150.0 140.0 130.0 120.0 110.0 100.0 90.0 80.0 70.0 60.0 50.0 40.0 30.0 20.0 10.0 0
170.170
151.949
133.919
133.042
128.580
122.125
86.742
83.539
81.641
77.484
77.160
76.845
56.756
52.608
28.114
21.564
X : parts per Million : 13C

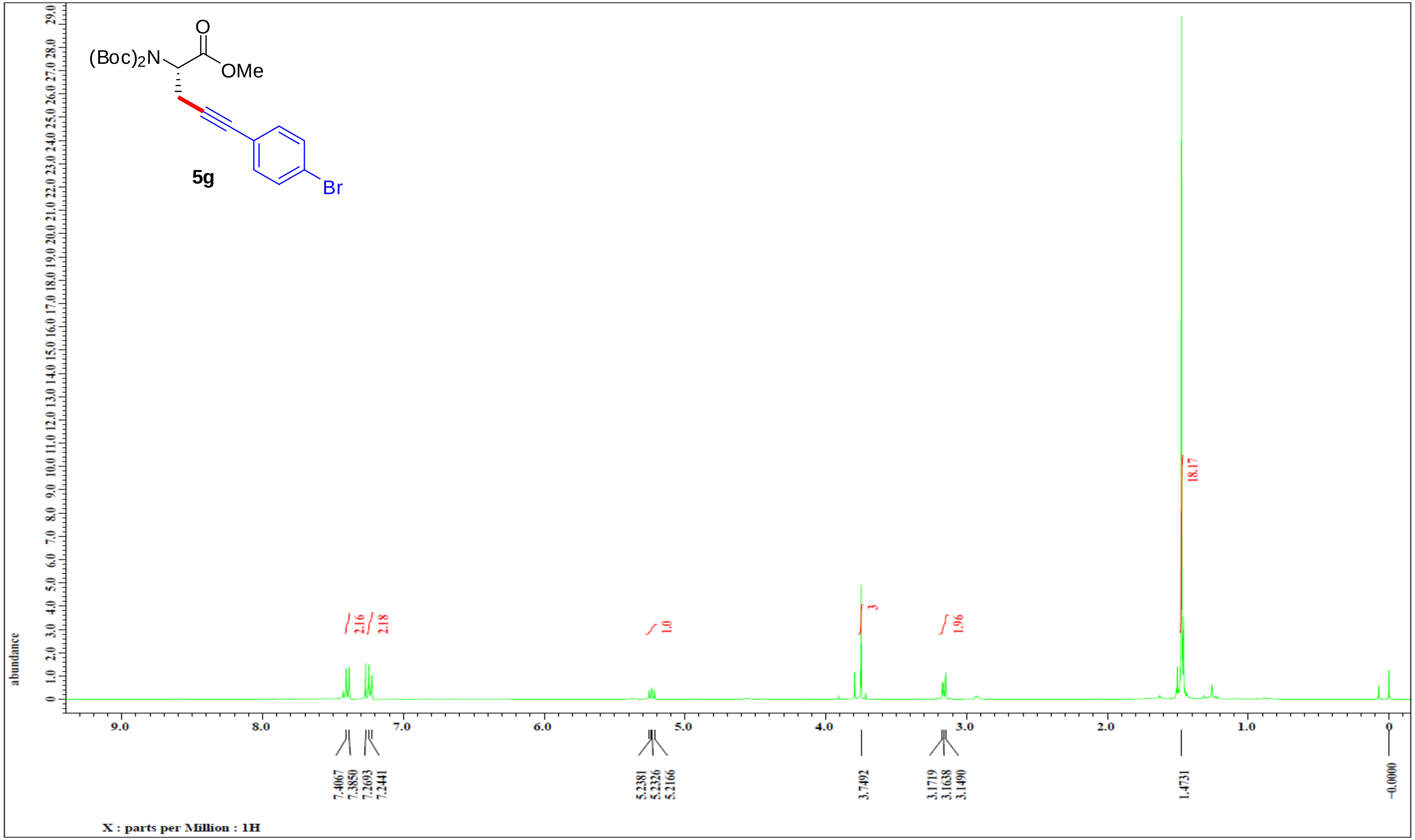
(Boc)2N
O
OMe
Br
5g
abundance
2.16
2.18
1.0
3
1.96
18.17
7.4067
7.3850
7.2693
7.2441
5.2381
5.2326
5.2166
3.7492
3.1719
3.1638
3.1490
1.4731
-0.0000
9.0
8.0
7.0
6.0
5.0
4.0
3.0
2.0
1.0
0
X : parts per Million : 1H

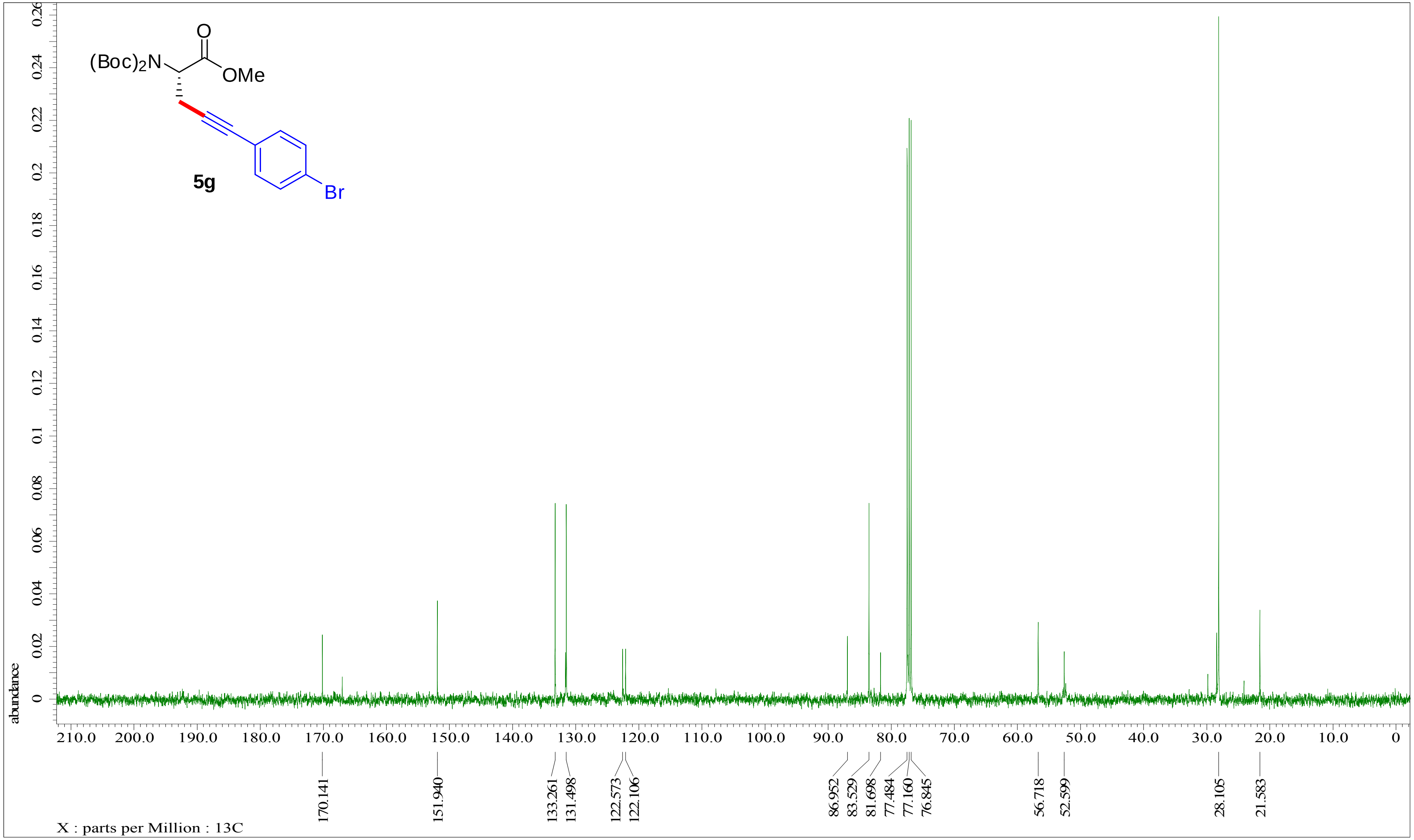

(Boc)2N
O
OMe
5g
Br
abundance
0.26
0.24
0.22
0.2
0.18
0.16
0.14
0.12
0.1
0.08
0.06
0.04
0.02
0
210.0 200.0 190.0 180.0 170.0 160.0 150.0 140.0 130.0 120.0 110.0 100.0 90.0 80.0 70.0 60.0 50.0 40.0 30.0 20.0 10.0 0
170.141
151.940
133.261
131.498
122.573
122.106
86.952
83.529
81.698
77.484
77.160
76.845
56.718
52.599
28.105
21.583
X : parts per Million : 13C

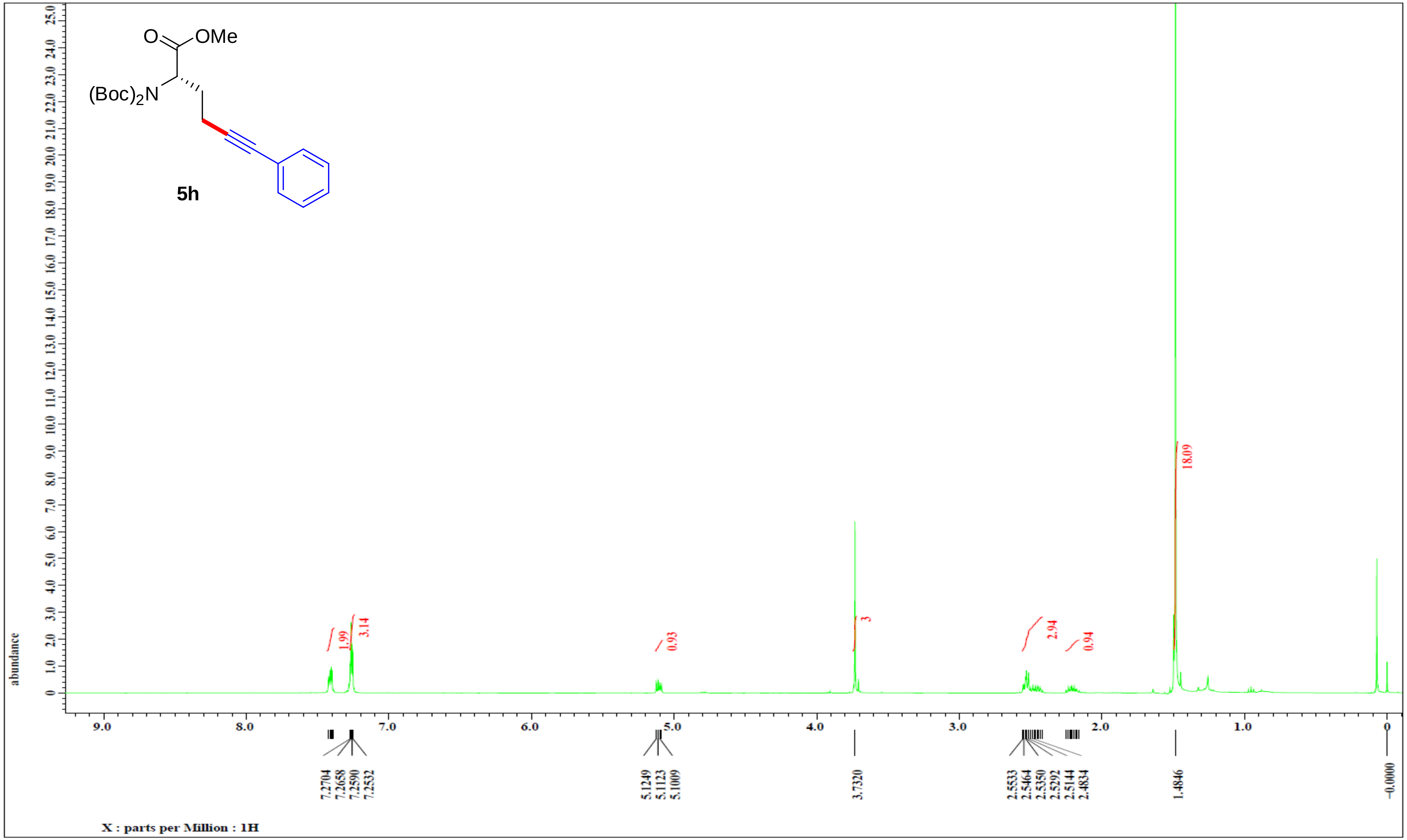
OMe
O
(Boc)2N
5h
abundance
X : parts per Million : 1H
1.99
3.14
0.93
3
2.94
0.94
18.09
7.2704
7.2658
7.2590
7.2532
5.1249
5.1123
5.1009
3.7320
2.5533
2.5464
2.5350
2.5292
2.5144
2.4834
1.4846
-0.0000

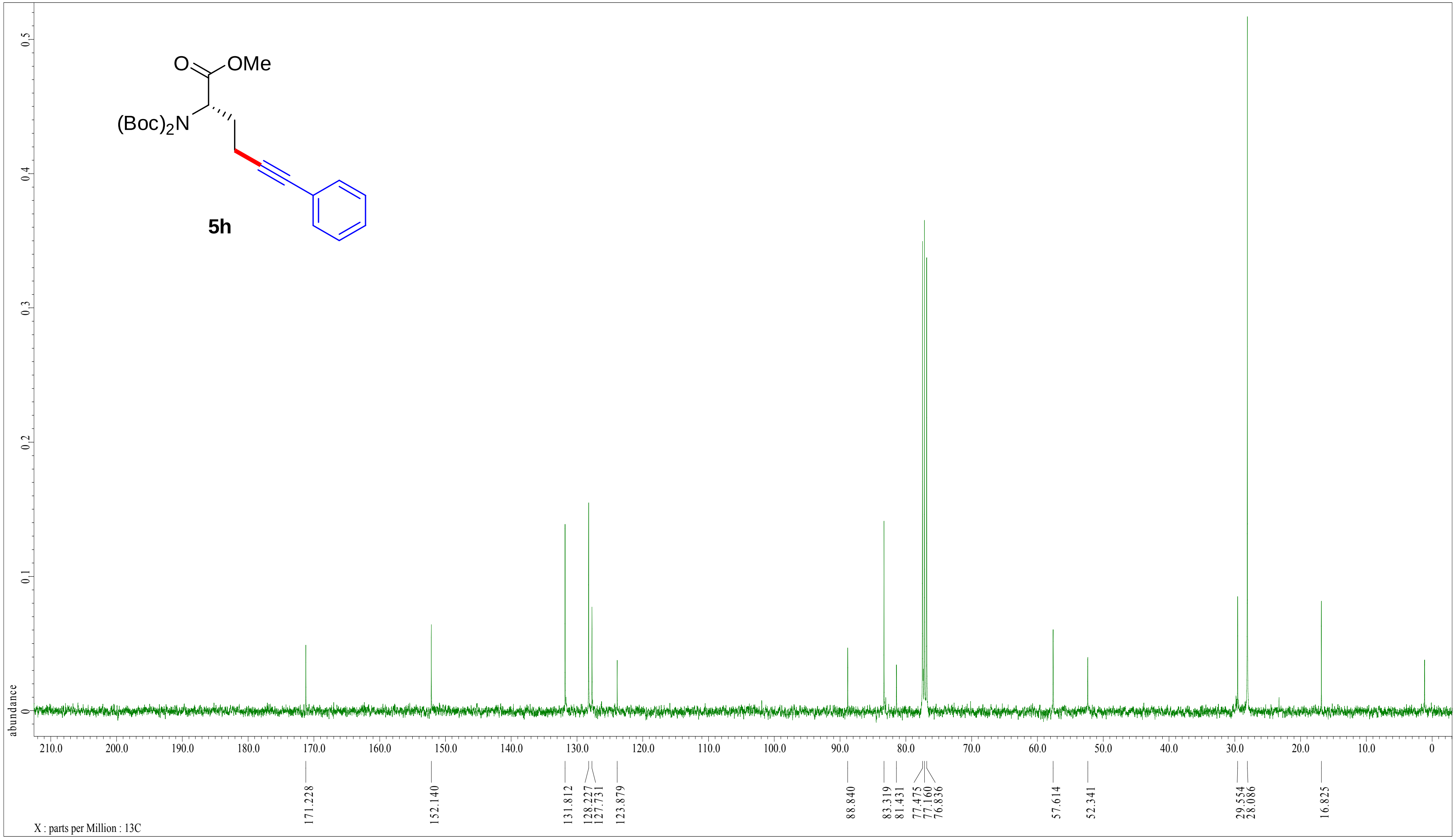

O
OMe
(Boc)2N
5h
abundance
0.5
0.4
0.3
0.2
0.1
0
210.0
200.0
190.0
180.0
170.0
160.0
150.0
140.0
130.0
120.0
110.0
100.0
90.0
80.0
70.0
60.0
50.0
40.0
30.0
20.0
10.0
0
171.228
152.140
131.812
128.227
127.731
123.879
88.840
83.319
81.431
77.475
77.160
76.836
57.614
52.341
29.554
28.086
16.825
X : parts per Million : 13C

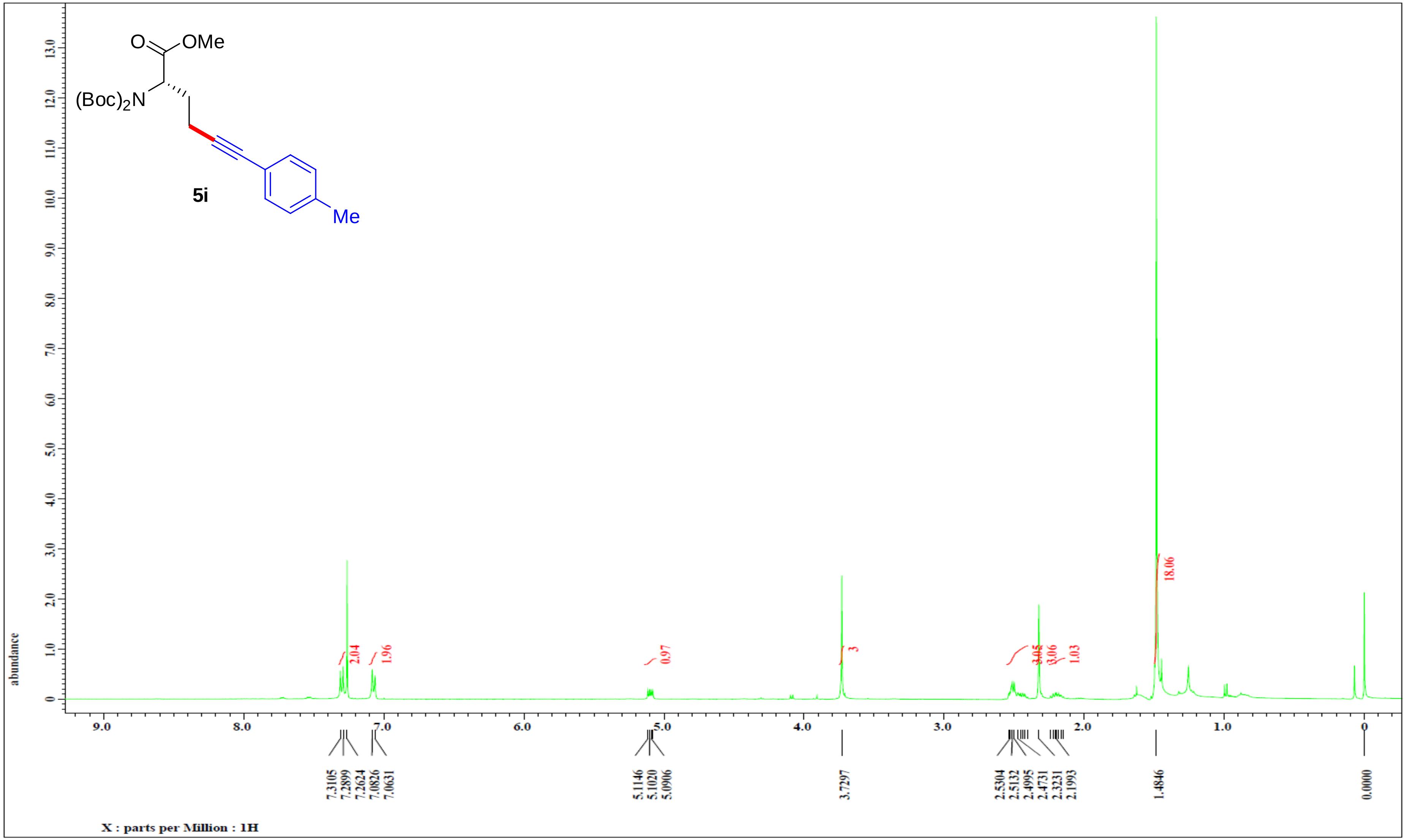
O
OMe
(Boc)2N
5i
Me
abundance
X : parts per Million : 1H
7.3105
7.2899
7.2624
7.0826
7.0631
5.1146
5.1020
5.0906
3.7297
2.5304
2.5132
2.4995
2.4731
2.3231
2.1993
1.4846
0.0000
2.04
1.96
0.97
3
3.05
3.06
1.03
18.06

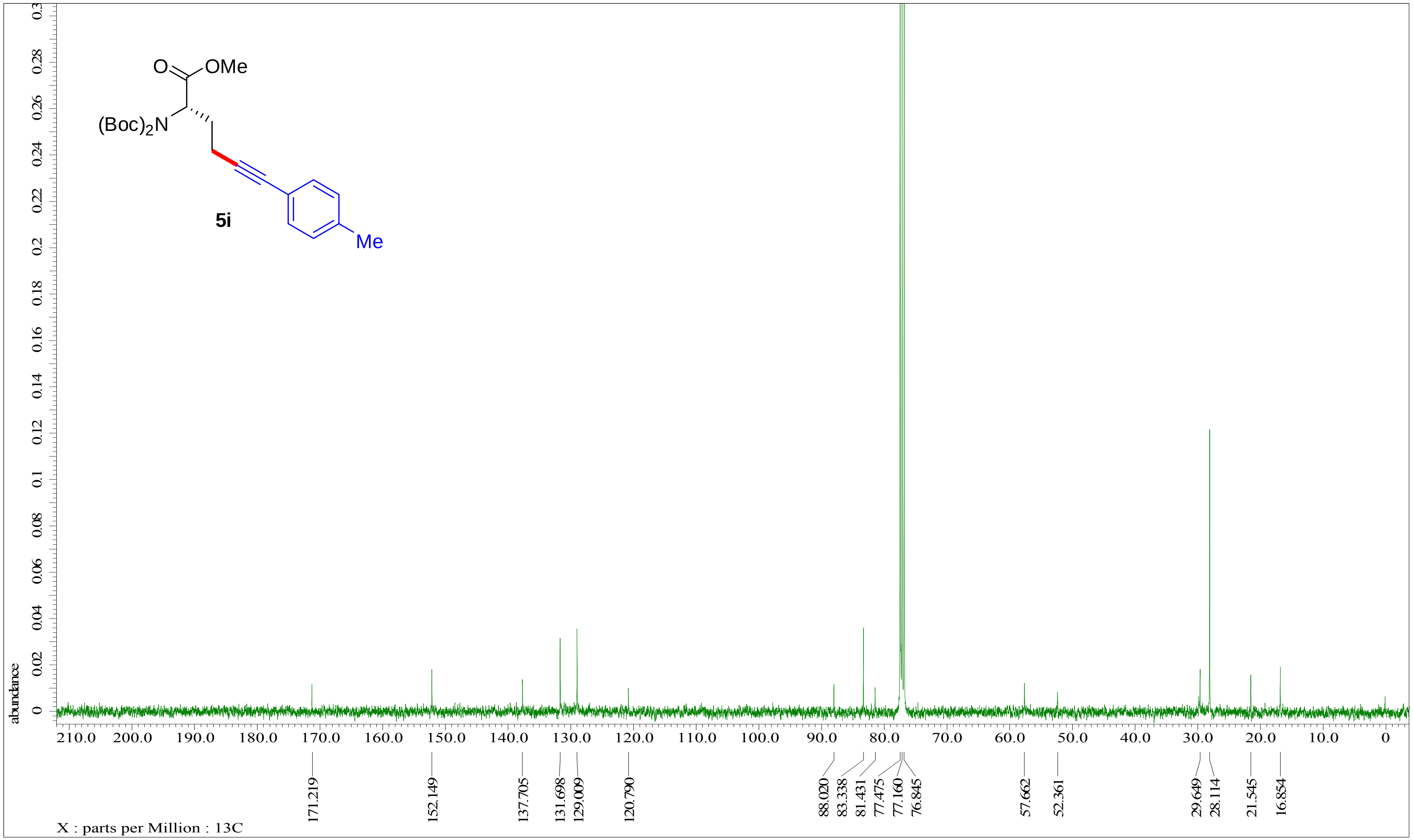

O
OMe
(Boc)2N
5i
Me
abundance
0
0.02
0.04
0.06
0.08
0.1
0.12
0.14
0.16
0.18
0.2
0.22
0.24
0.26
0.28
0.3
210.0
200.0
190.0
180.0
170.0
160.0
150.0
140.0
130.0
120.0
110.0
100.0
90.0
80.0
70.0
60.0
50.0
40.0
30.0
20.0
10.0
0
171.219
152.149
137.705
131.698
129.009
120.790
88.020
83.338
81.431
77.475
77.160
76.845
57.662
52.361
29.649
28.114
21.545
16.854
X : parts per Million : 13C

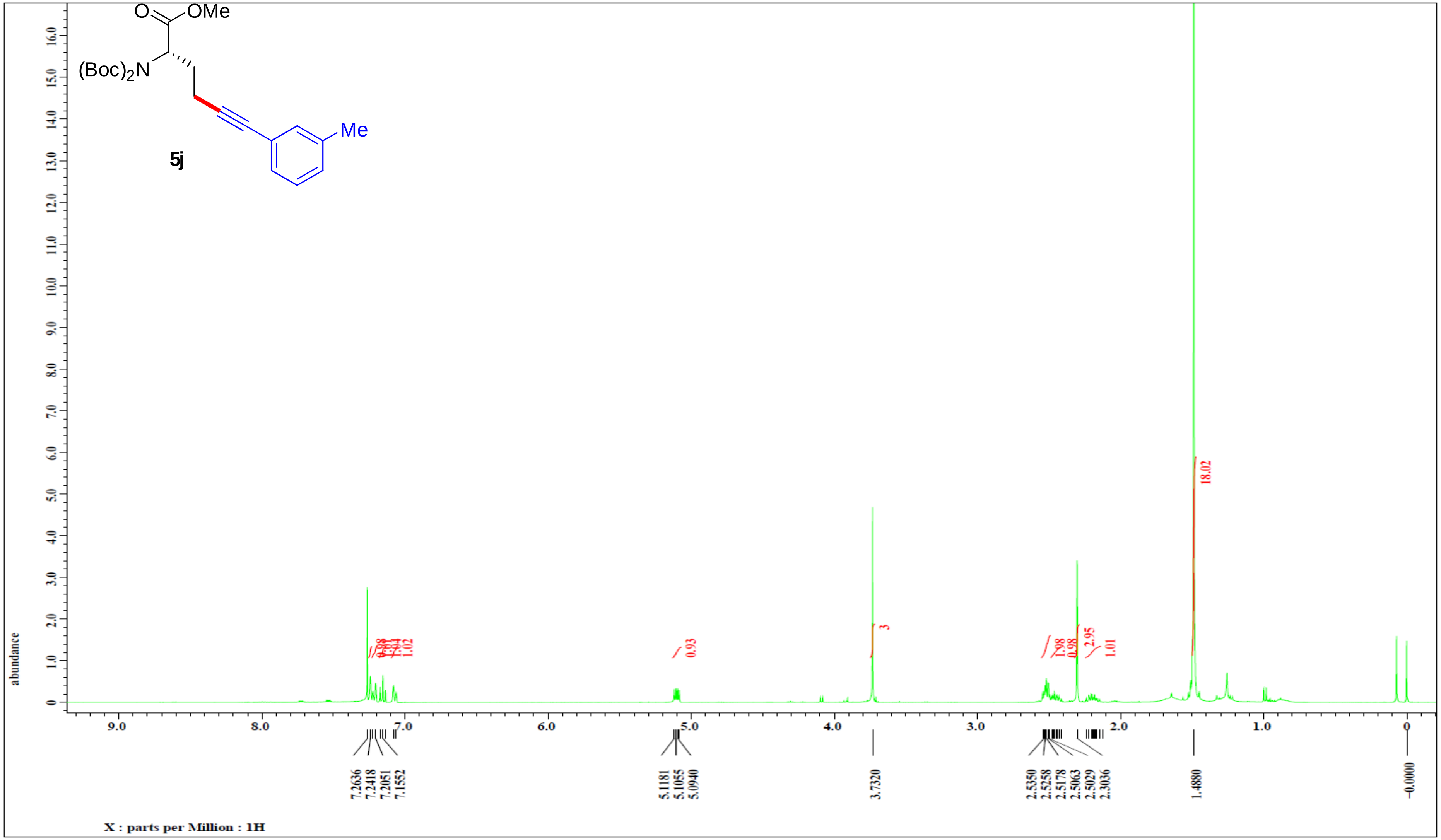

OMe
O
(Boc)2N
Me
5j
abundance
X : parts per Million : 1H
7.2636
7.2418
7.2051
7.1552
5.1181
5.1055
5.0940
3.7320
2.5350
2.5258
2.5178
2.5063
2.5029
2.3036
1.4880
-0.0000
0.93
3
18.02
2.95
1.01
1.02

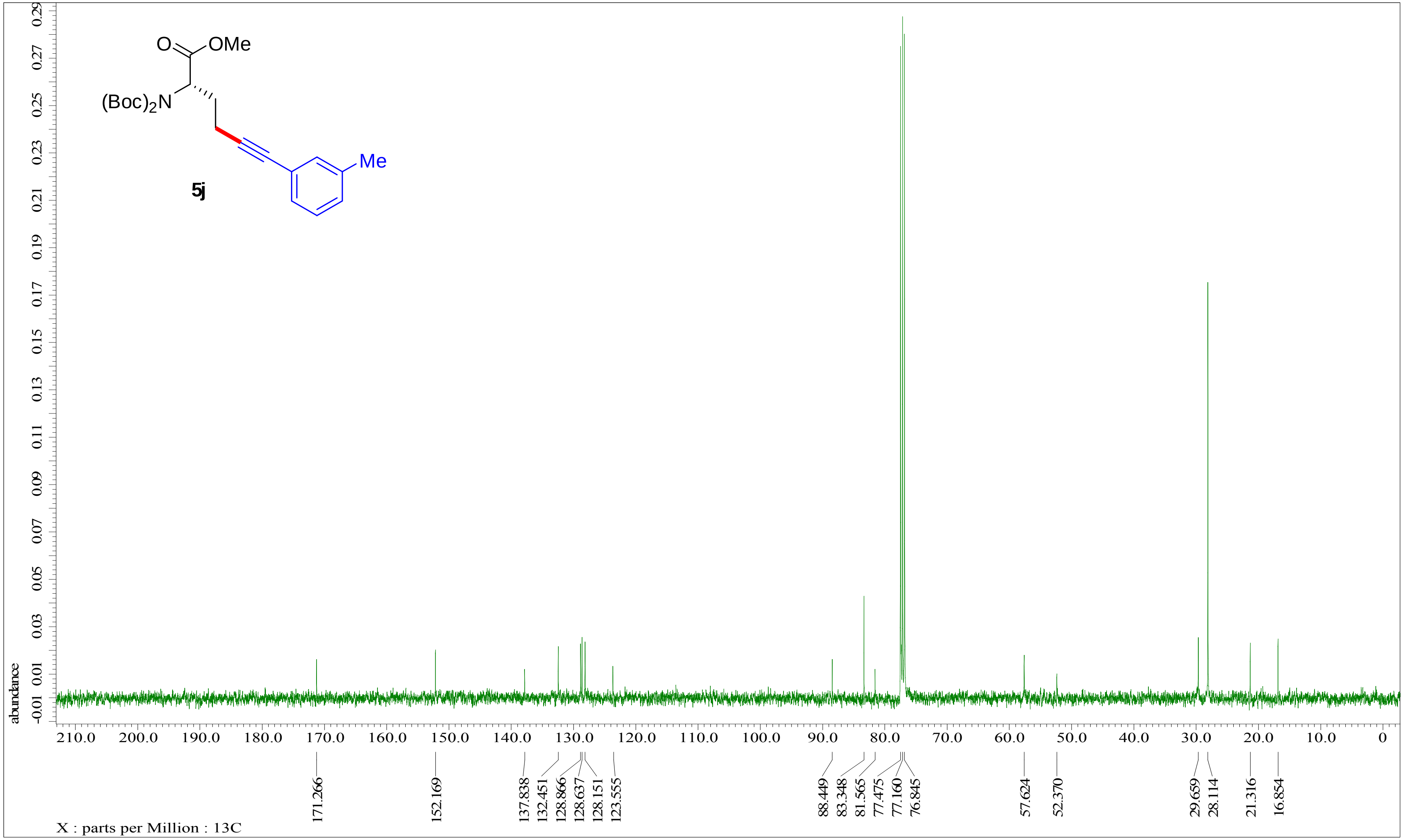

OMe
O
(Boc)2N
Me
5j
abundance
X : parts per Million : 13C
171.266
152.169
137.838
132.451
128.866
128.637
128.151
123.555
88.449
83.348
81.565
77.475
77.160
76.845
57.624
52.370
29.659
28.114
21.316
16.854

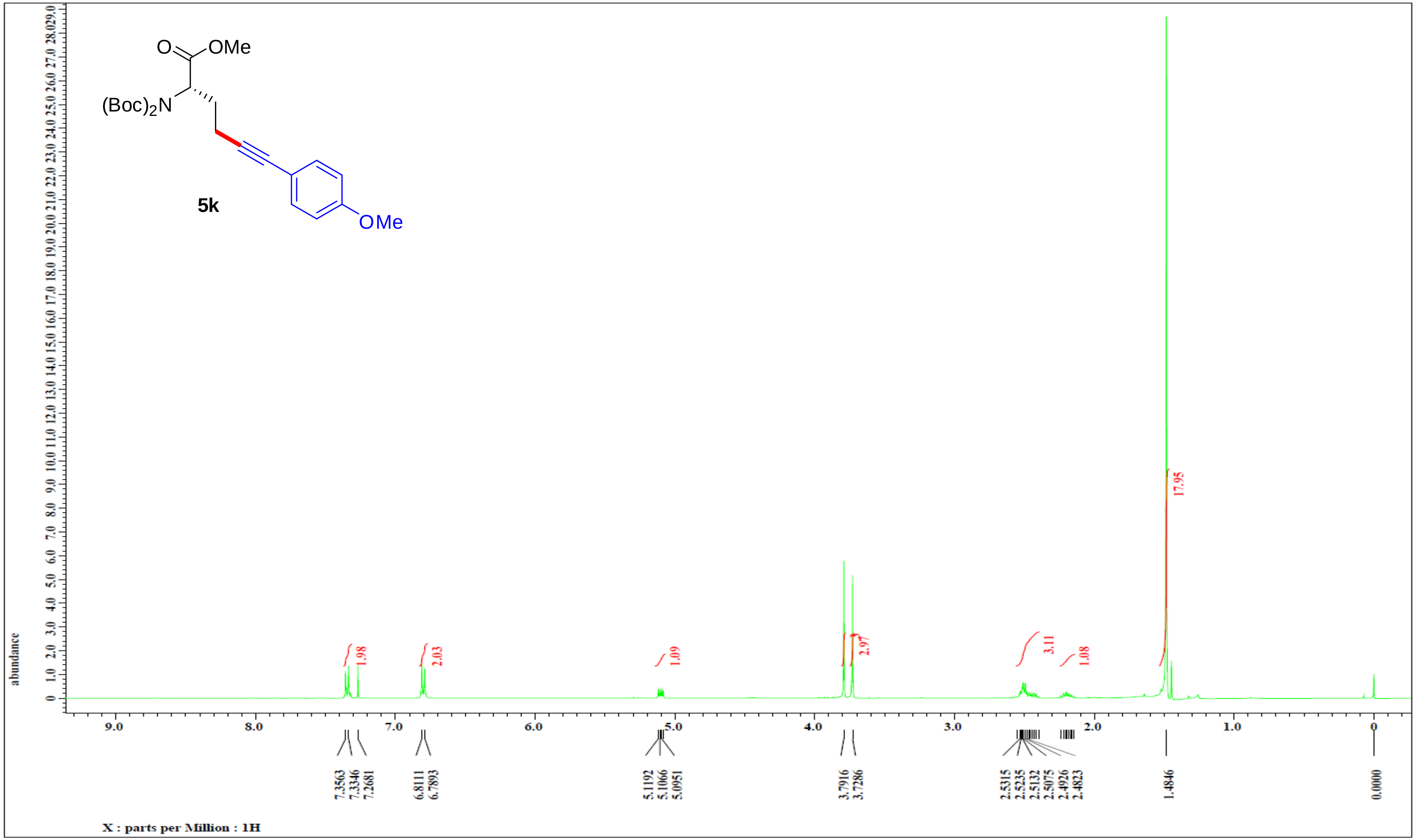

O
OMe
(Boc)2N
5k
OMe
abundance
X : parts per Million : 1H
9.0
8.0
7.0
6.0
5.0
4.0
3.0
2.0
1.0
0
7.3563
7.3346
7.2681
6.8111
6.7893
5.1192
5.1066
5.0951
3.7916
3.7286
2.5315
2.5235
2.5132
2.5075
2.4926
2.4823
1.4846
0.0000
1.98
2.03
1.09
2.97
3.11
1.08
17.95

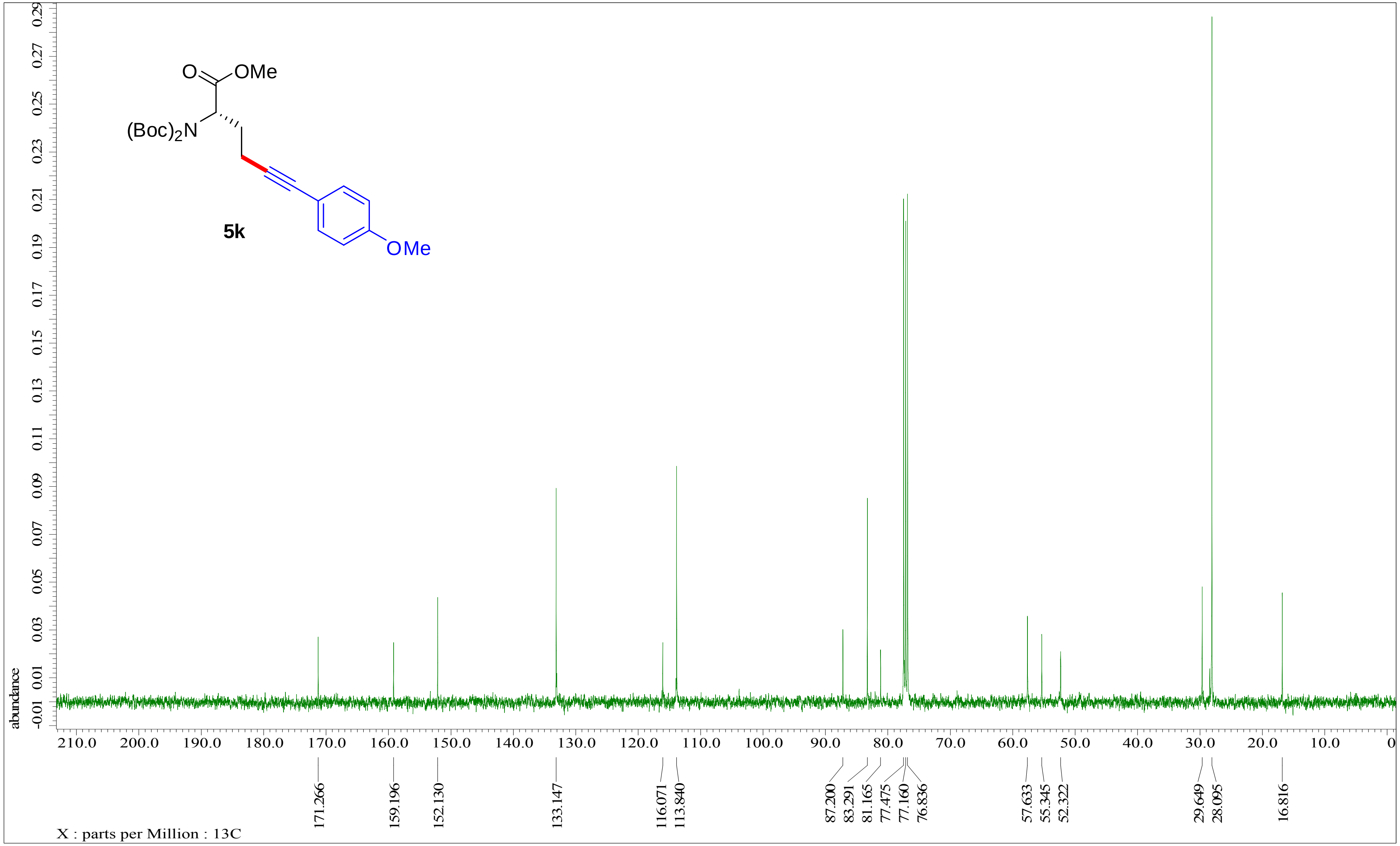
O
OMe
(Boc)2N
5k
OMe
abundance
X : parts per Million : 13C
171.266
159.196
152.130
133.147
116.071
113.840
87.200
83.291
81.165
77.475
77.160
76.836
57.633
55.345
52.322
29.649
28.095
16.816

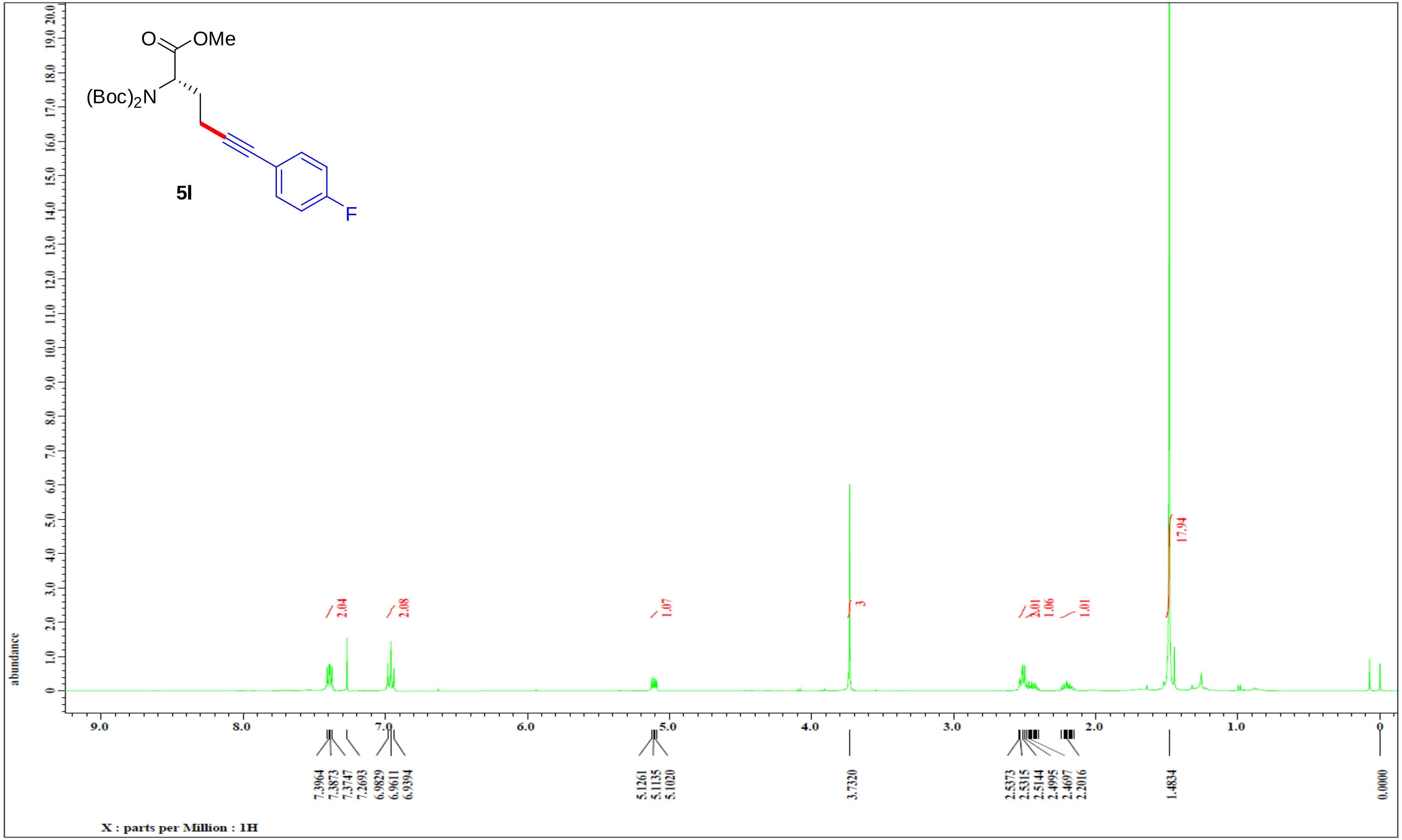

OMe
O
(Boc)2N
F
5l
abundance
X : parts per Million : 1H
2.04
2.08
1.07
3
2.01
1.06
1.01
17.94
7.3964
7.3873
7.3747
7.2693
6.9829
6.9611
6.9394
5.1261
5.1135
5.1020
3.7320
2.5373
2.5315
2.5144
2.4995
2.4697
2.2016
1.4834
0.0000

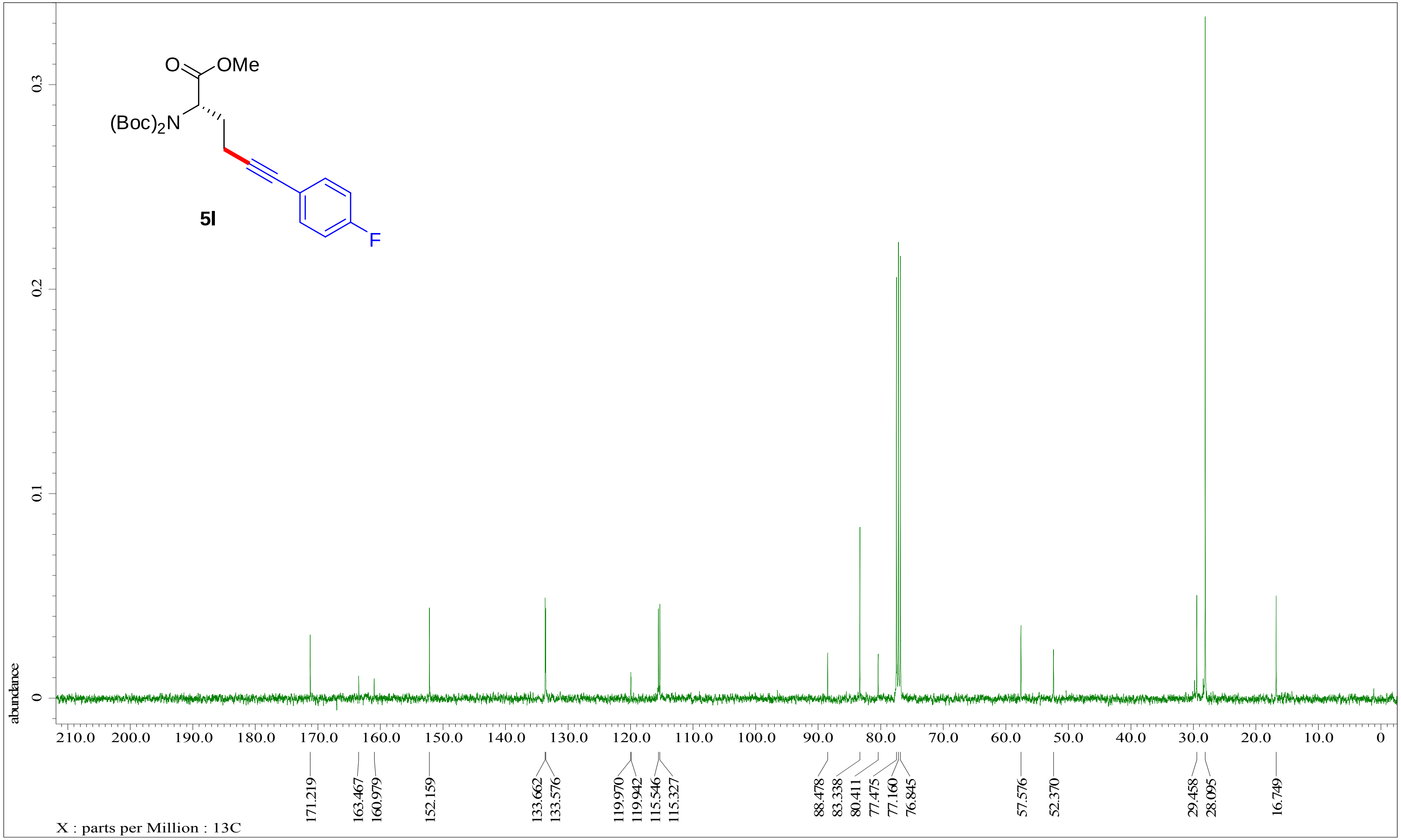
OMe
O
(Boc)2N
F
5l
0.3
0.2
0.1
0
abundance
210.0 200.0 190.0 180.0 170.0 160.0 150.0 140.0 130.0 120.0 110.0 100.0 90.0 80.0 70.0 60.0 50.0 40.0 30.0 20.0 10.0 0
171.219
163.467
160.979
152.159
133.662
133.576
119.970
119.942
115.546
115.327
88.478
83.338
80.411
77.475
77.160
76.845
57.576
52.370
29.458
28.095
16.749
X : parts per Million : 13C

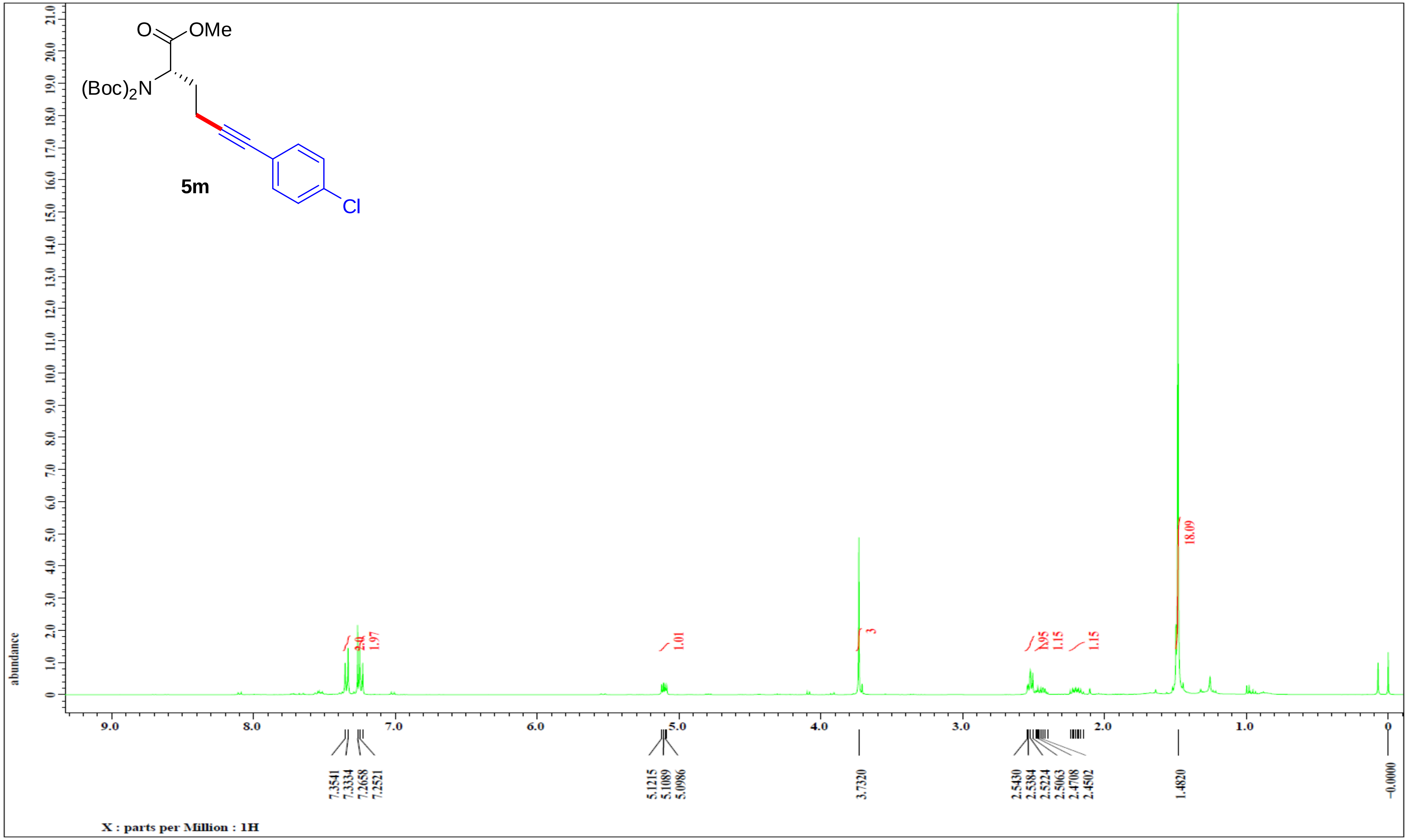

O
OMe
(Boc)$_2$N
Cl
5m
abundance
X : parts per Million : 1H
2.01
1.97
1.01
3
1.95
1.15
1.15
18.09
7.3541
7.3334
7.2658
7.2521
5.1215
5.1089
5.0986
3.7320
2.5430
2.5384
2.5224
2.5063
2.4708
2.4502
1.4820
-0.0000

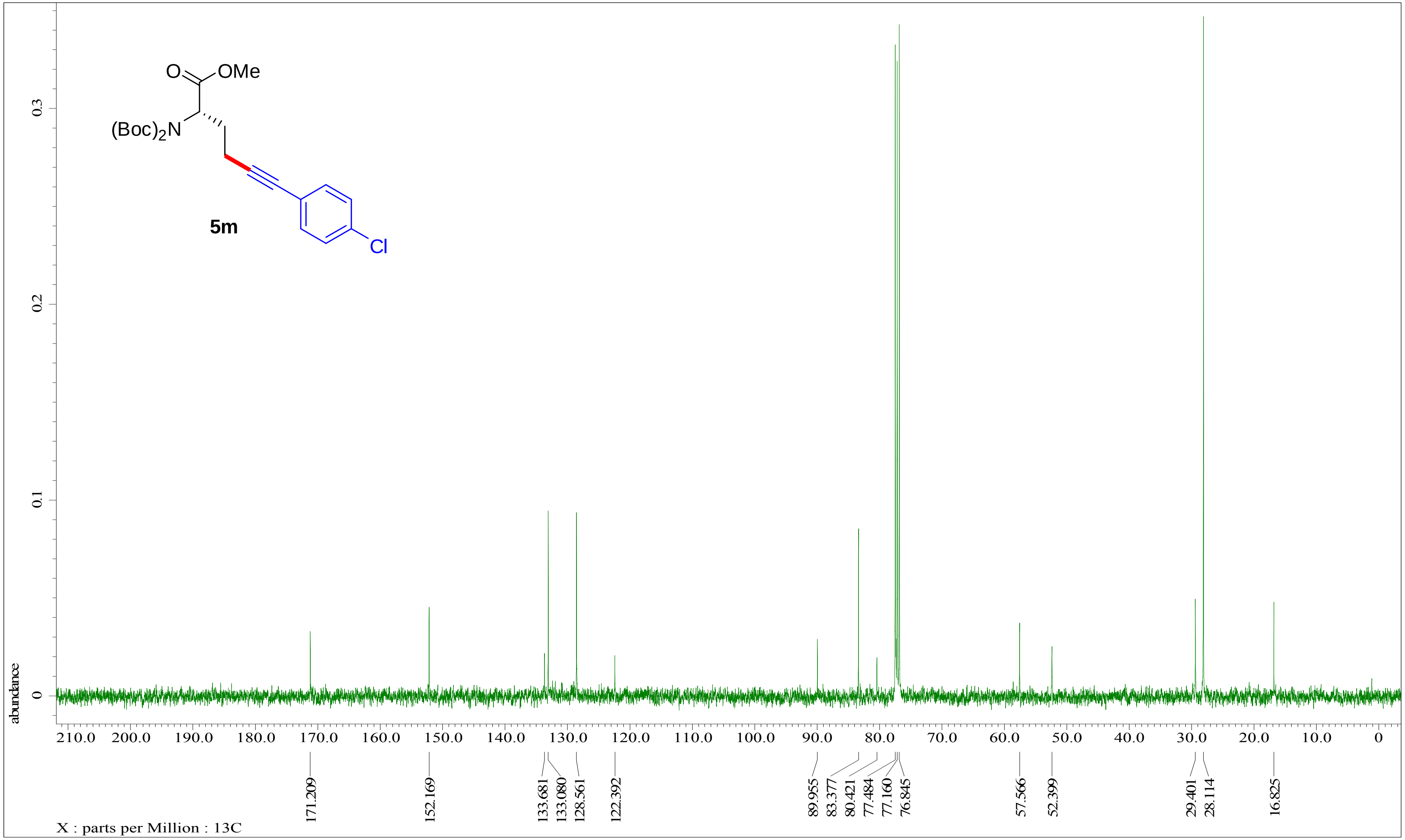

O
OMe
(Boc)2N
Cl
5m
abundance
0.3
0.2
0.1
0
210.0 200.0 190.0 180.0 170.0 160.0 150.0 140.0 130.0 120.0 110.0 100.0 90.0 80.0 70.0 60.0 50.0 40.0 30.0 20.0 10.0 0
171.209
152.169
133.681
133.080
128.561
122.392
89.955
83.377
80.421
77.484
77.160
76.845
57.566
52.399
29.401
28.114
16.825
X : parts per Million : 13C

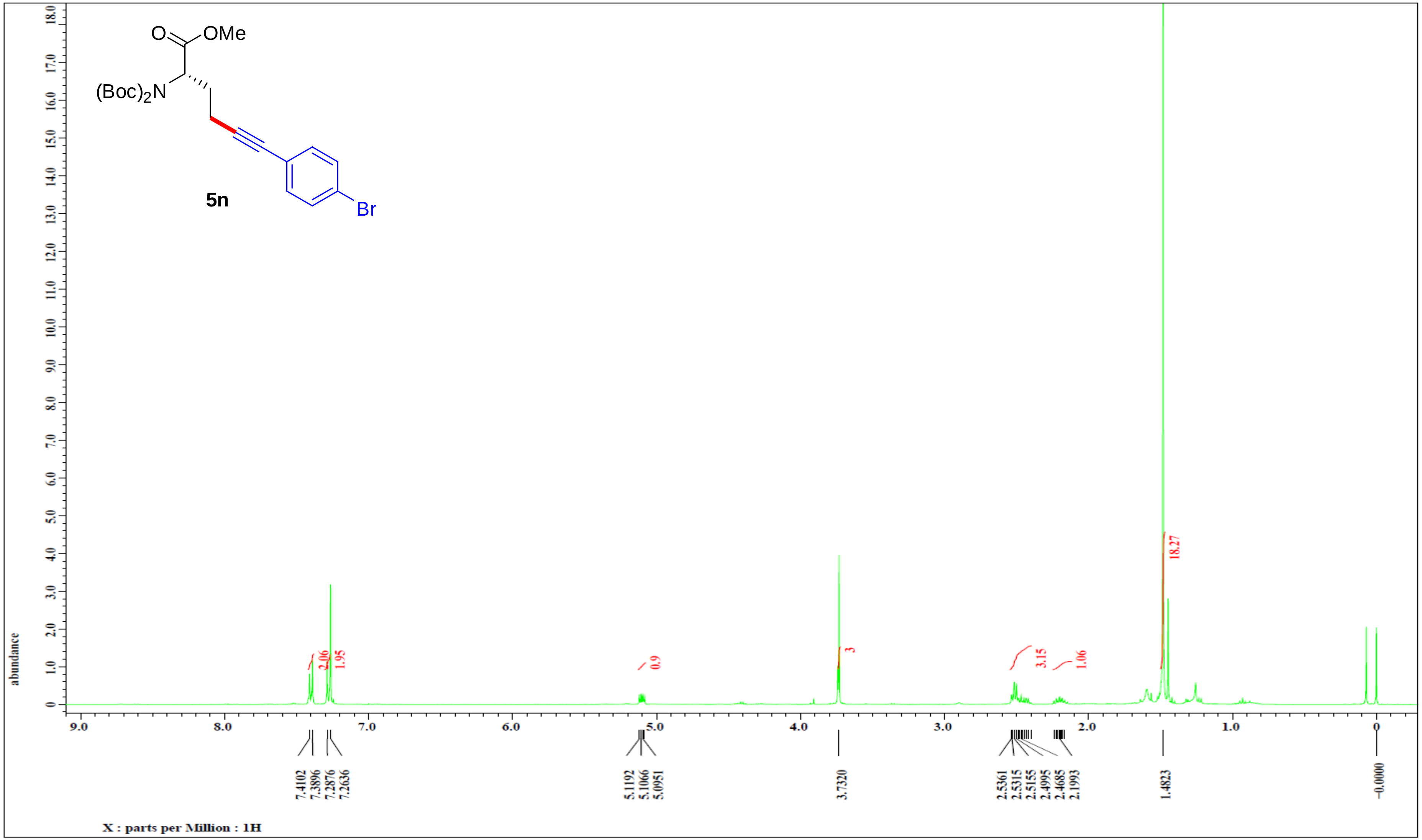

OMe
(Boc)2N
Br
5n
abundance
X : parts per Million : 1H
7.4102
7.3896
7.2876
7.2636
5.1192
5.1066
5.0951
3.7320
2.5361
2.5315
2.5155
2.4995
2.4685
2.1993
1.4823
-0.0000
2.06
1.95
0.9
3
3.15
1.06
18.27

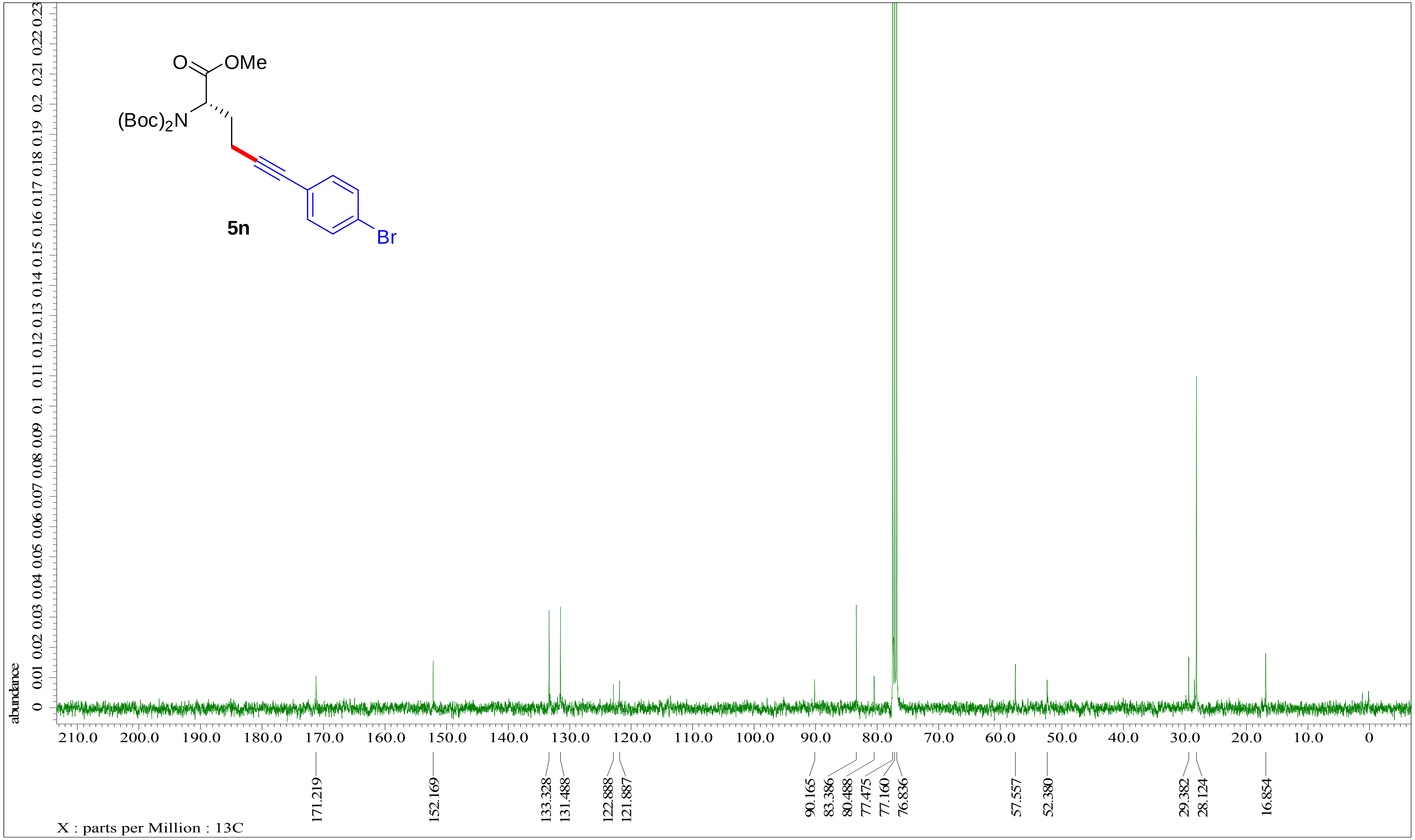

O
OMe
(Boc)2N
5n
Br
abundance
X : parts per Million : 13C
171.219
152.169
133.328
131.488
122.888
121.887
90.165
83.386
80.488
77.475
77.160
76.836
57.557
52.380
29.382
28.124
16.854

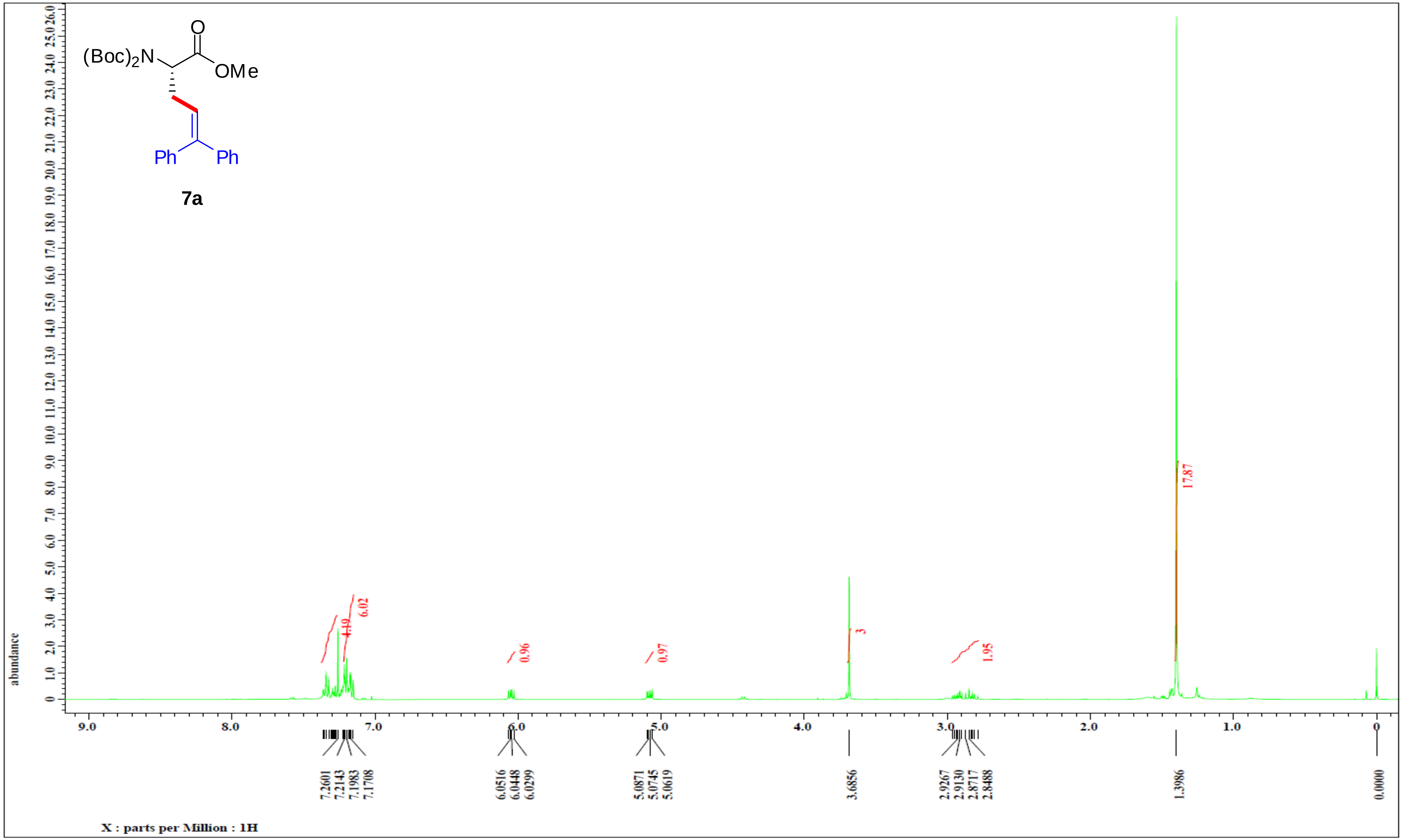
(Boc)2N
O
OMe
Ph
Ph
7a
abundance
X : parts per Million : 1H
6.02
4.19
0.96
0.97
3
1.95
17.87
7.2601
7.2143
7.1983
7.1708
6.0516
6.0448
6.0299
5.0871
5.0745
5.0619
3.6856
2.9267
2.9130
2.8717
2.8488
1.3986
0.0000

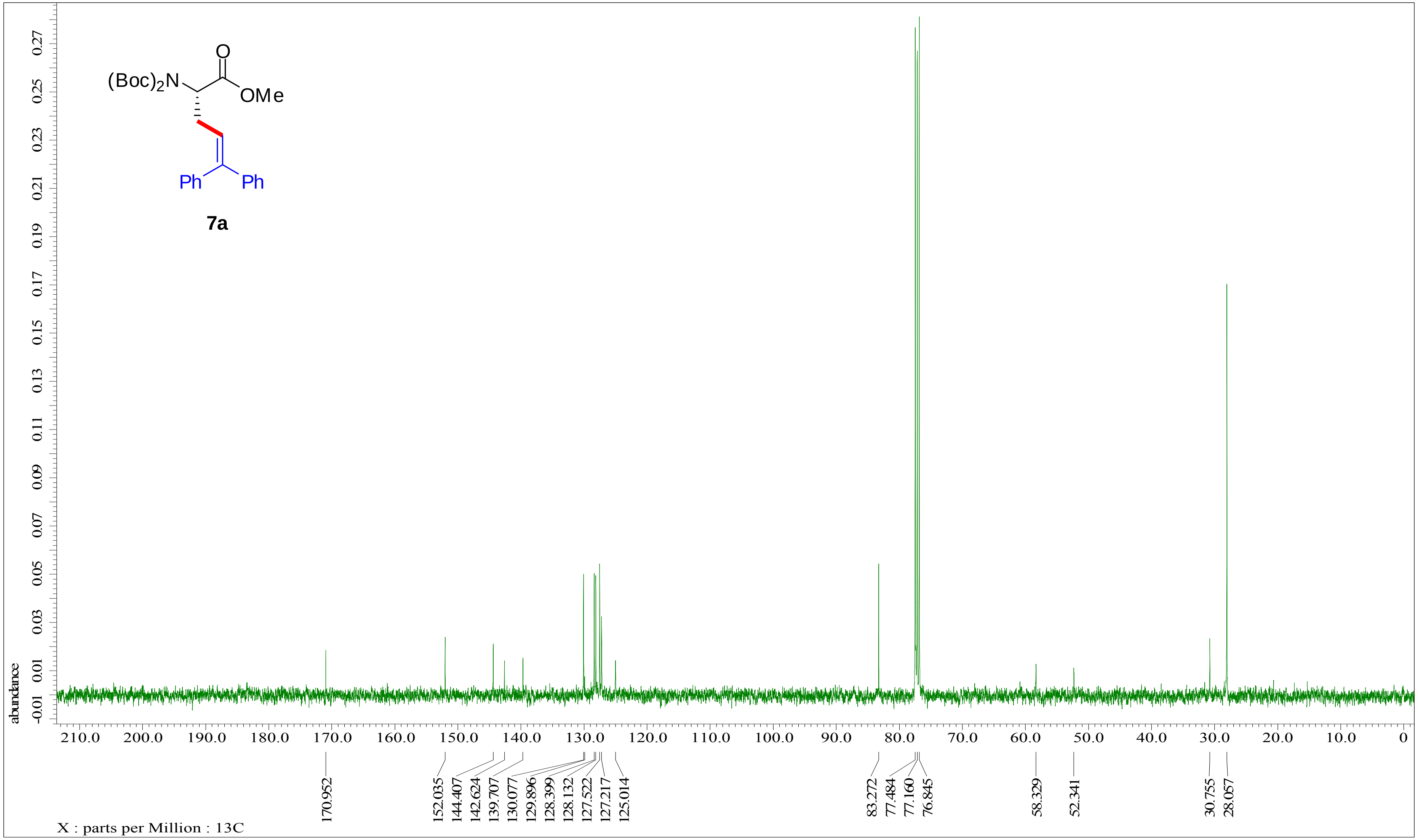
(Boc)2N
O
OMe
Ph
Ph
7a
abundance
X : parts per Million : 13C
170.952
152.035
144.407
142.624
139.707
130.077
129.896
128.399
128.132
127.522
127.217
125.014
83.272
77.484
77.160
76.845
58.329
52.341
30.755
28.057

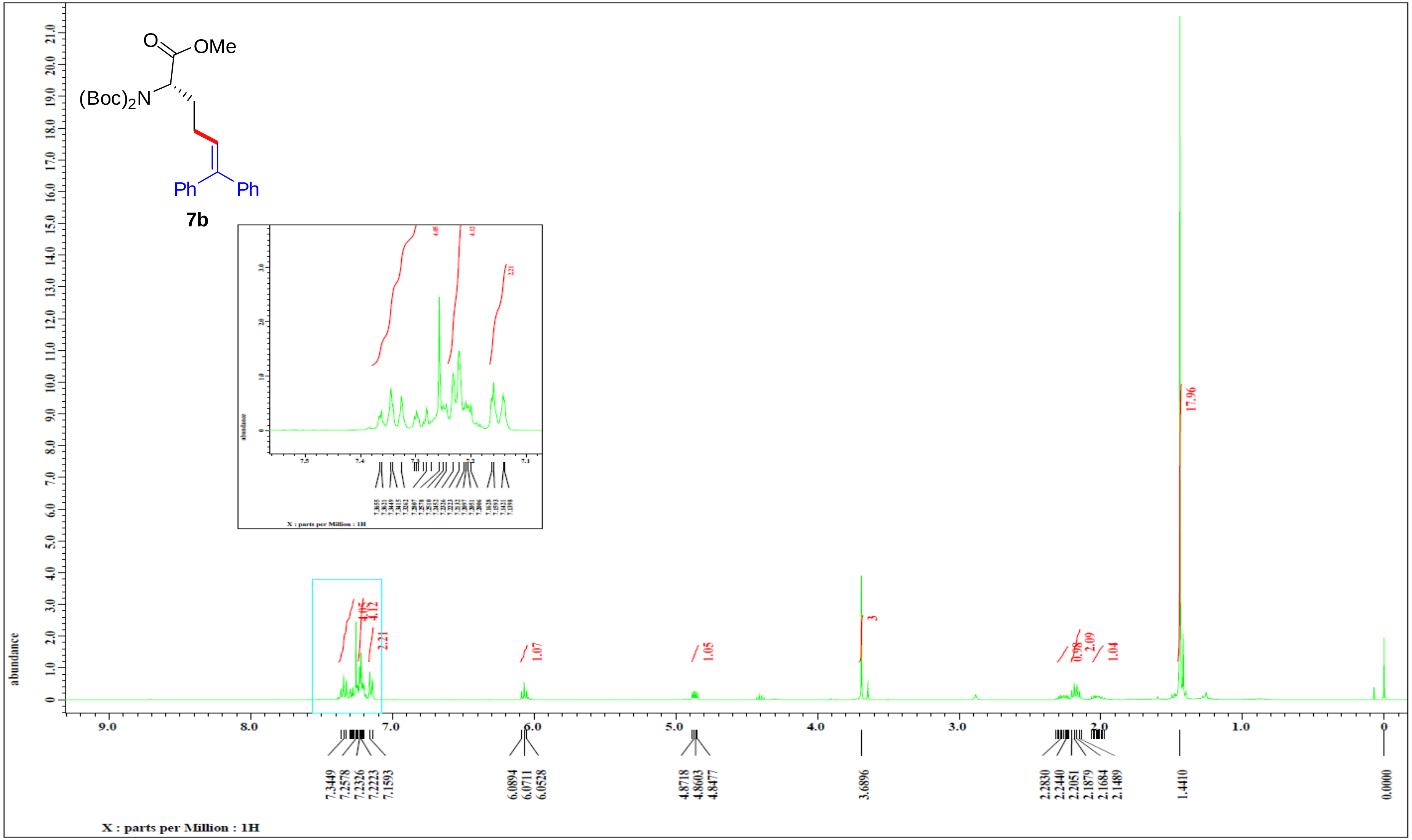

O
OMe
(Boc)2N
Ph
Ph
7b
abundance
X : parts per Million : 1H
7.3449
7.2578
7.2326
7.2223
7.1593
6.0894
6.0711
6.0528
4.8718
4.8603
4.8477
3.6896
2.2830
2.2440
2.2051
2.1879
2.1684
2.1489
1.4410
0.0000
4.05
4.12
2.21
1.07
1.05
3
0.98
2.09
1.04
17.96

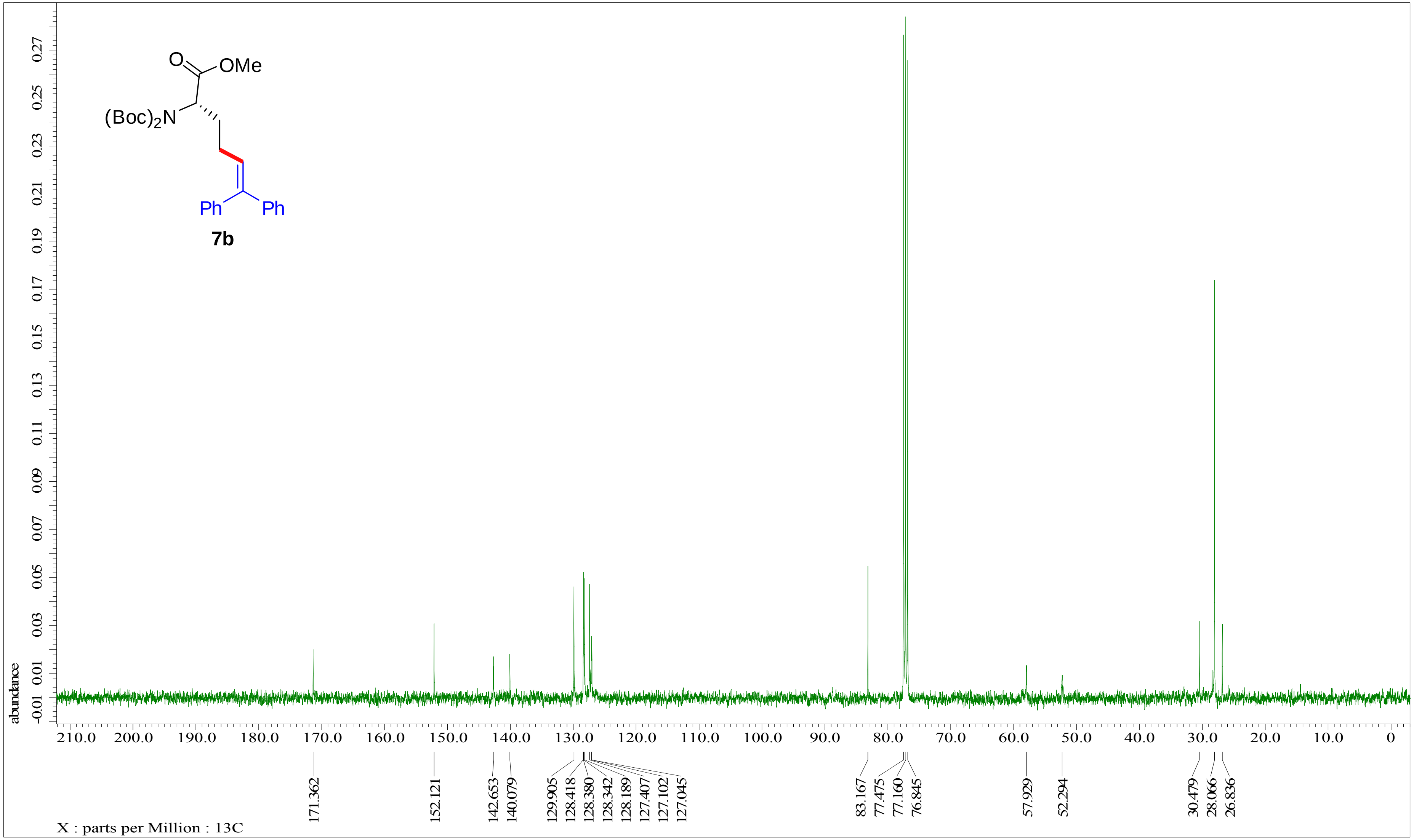

OMe
O
(Boc)2N
Ph
Ph
7b
abundance
171.362
152.121
142.653
140.079
129.905
128.418
128.380
128.342
128.189
127.407
127.102
127.045
83.167
77.475
77.160
76.845
57.929
52.294
30.479
28.066
26.836
X : parts per Million : 13C

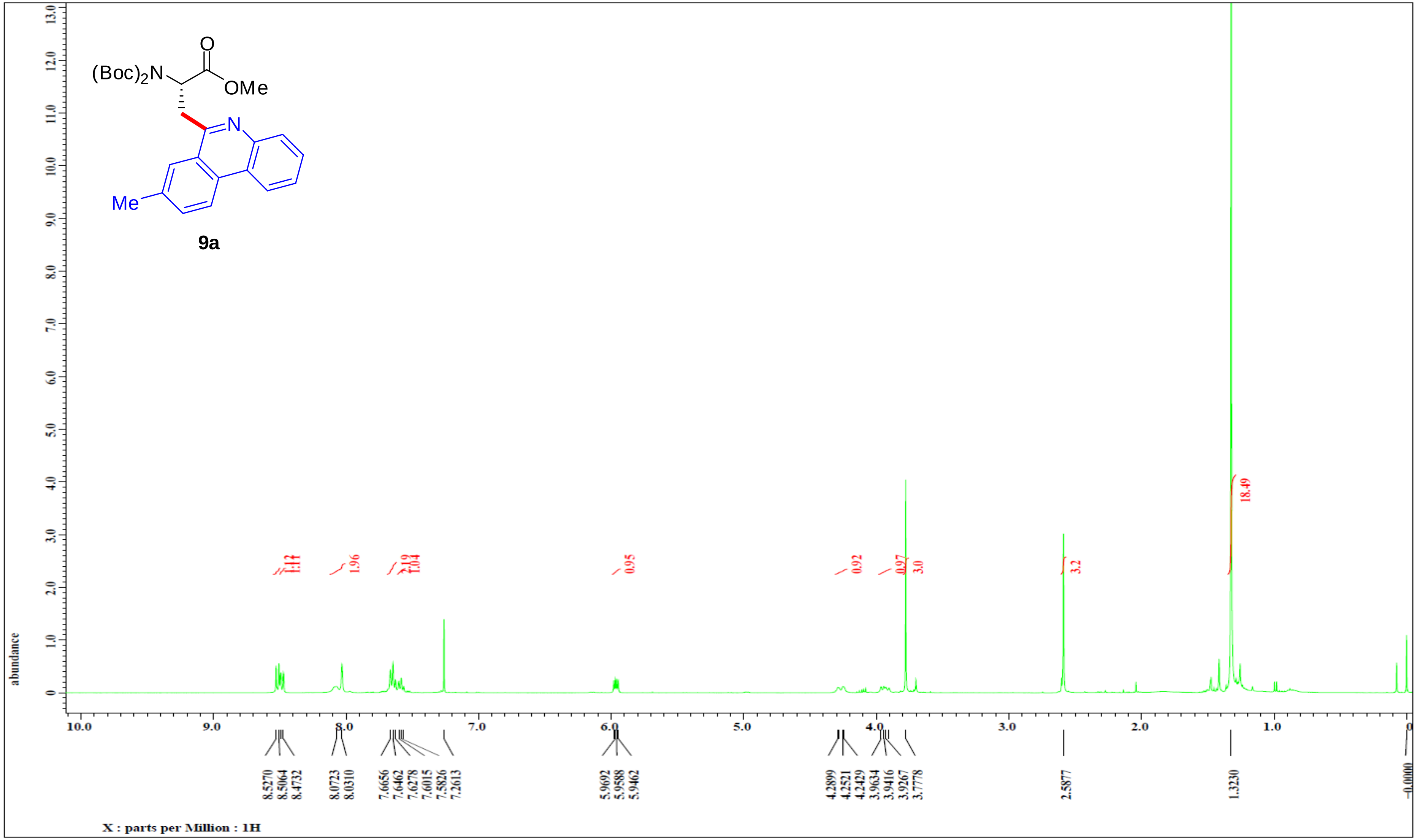
(Boc)2N
O
OMe
N
Me
9a
abundance
X : parts per Million : 1H
8.5270
8.5064
8.4732
8.0723
8.0310
7.6656
7.6462
7.6278
7.6015
7.5826
7.2613
5.9692
5.9588
5.9462
4.2899
4.2521
4.2429
3.9634
3.9416
3.9267
3.7778
2.5877
1.3230
-0.0000
1.96
0.95
0.92
3.0
3.2
18.49

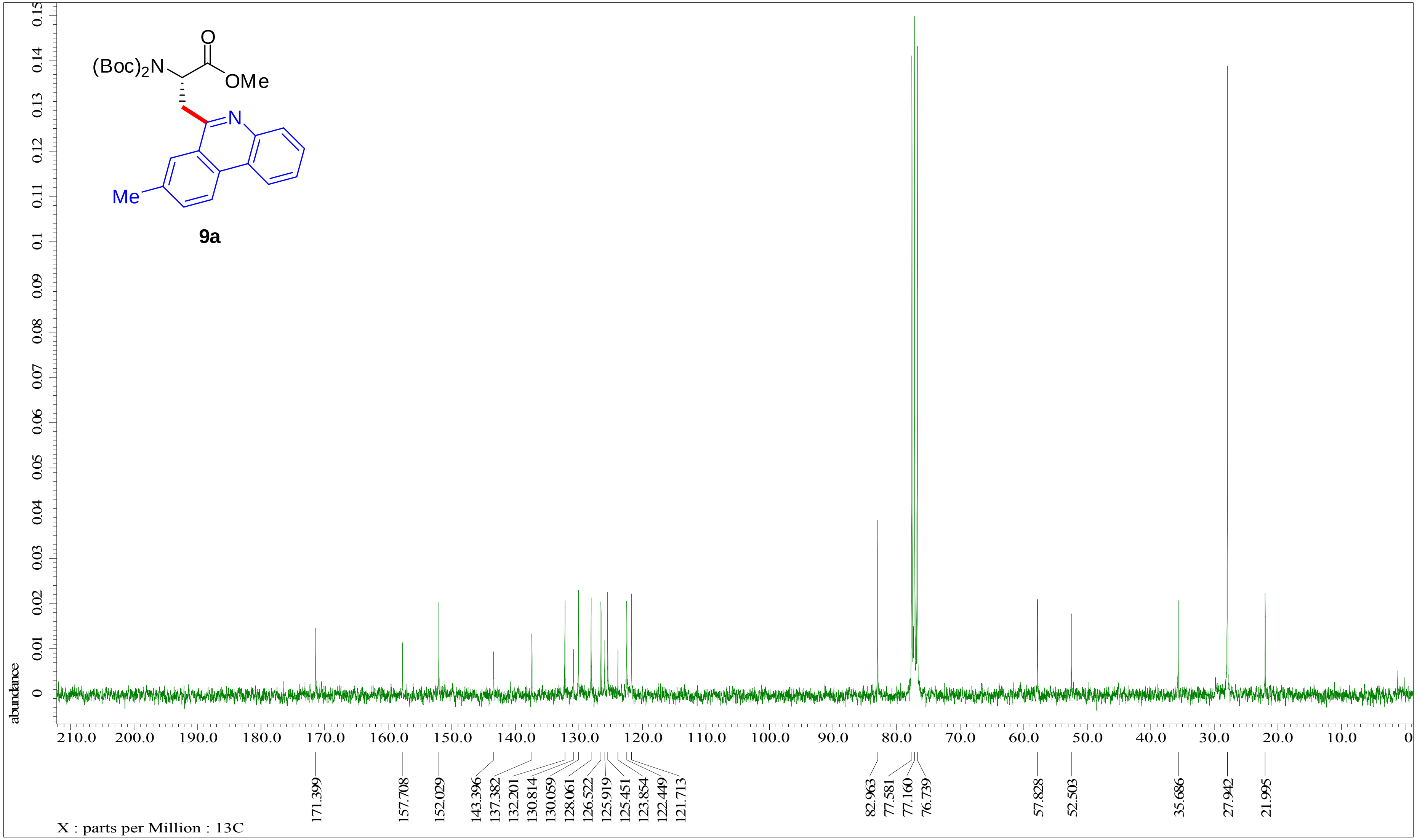

O
(Boc)2N
OMe
N
Me
9a
abundance
0.15
0.14
0.13
0.12
0.11
0.1
0.09
0.08
0.07
0.06
0.05
0.04
0.03
0.02
0.01
0
210.0
200.0
190.0
180.0
170.0
160.0
150.0
140.0
130.0
120.0
110.0
100.0
90.0
80.0
70.0
60.0
50.0
40.0
30.0
20.0
10.0
0
171.399
157.708
152.029
143.396
137.382
132.201
130.814
130.059
128.061
126.522
125.919
125.451
123.854
122.449
121.713
82.963
77.581
77.160
76.739
57.828
52.503
35.686
27.942
21.995
X : parts per Million : 13C

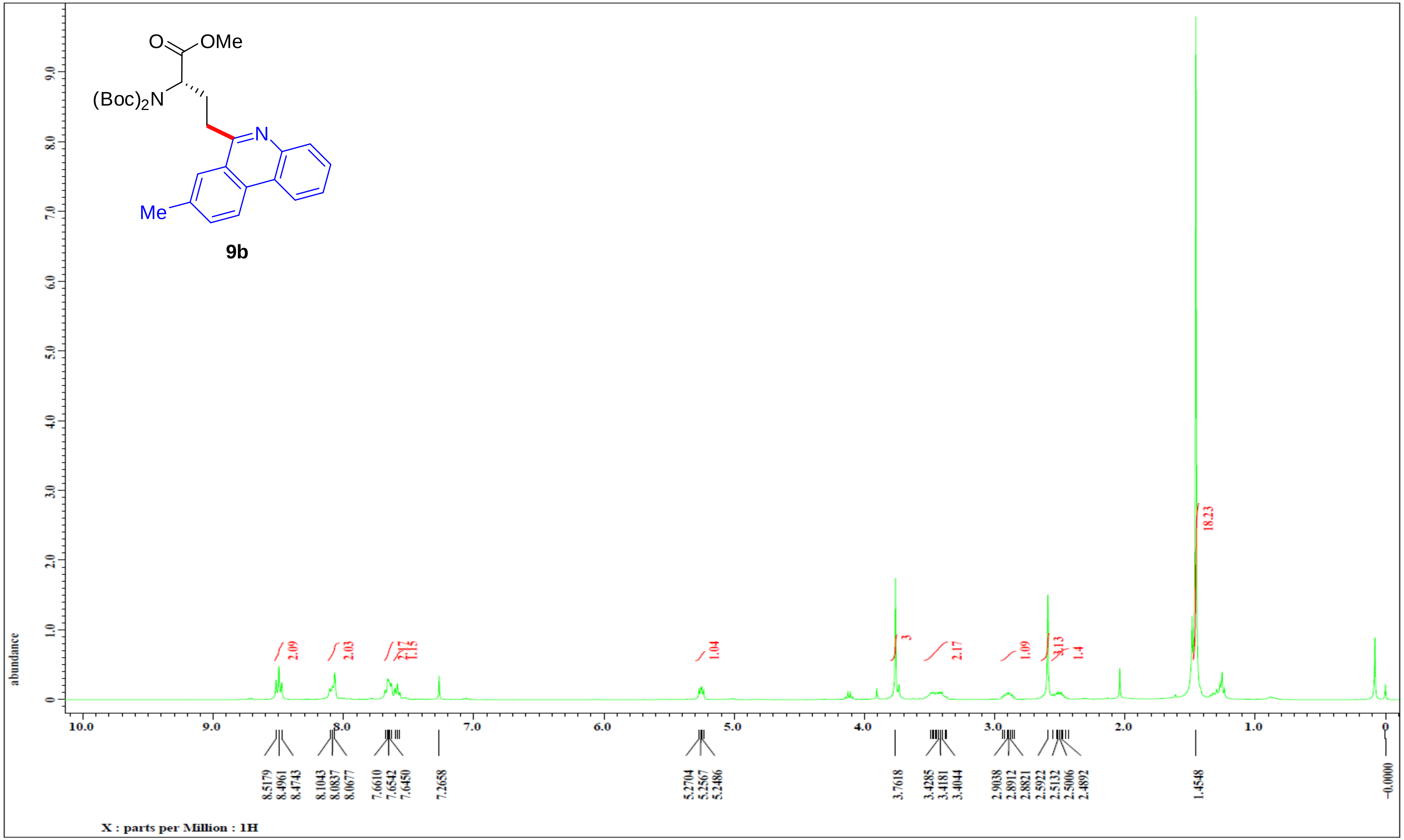
OMe
(Boc)2N
N
Me
9b
abundance
X : parts per Million : 1H
2.09
2.03
1.04
3
2.17
1.09
18.23
8.5179
8.4961
8.4743
8.1043
8.0837
8.0677
7.6610
7.6542
7.6450
7.2658
5.2704
5.2567
5.2486
3.7618
3.4285
3.4181
3.4044
2.9038
2.8912
2.8821
2.5922
2.5132
2.5006
2.4892
1.4548
-0.0000

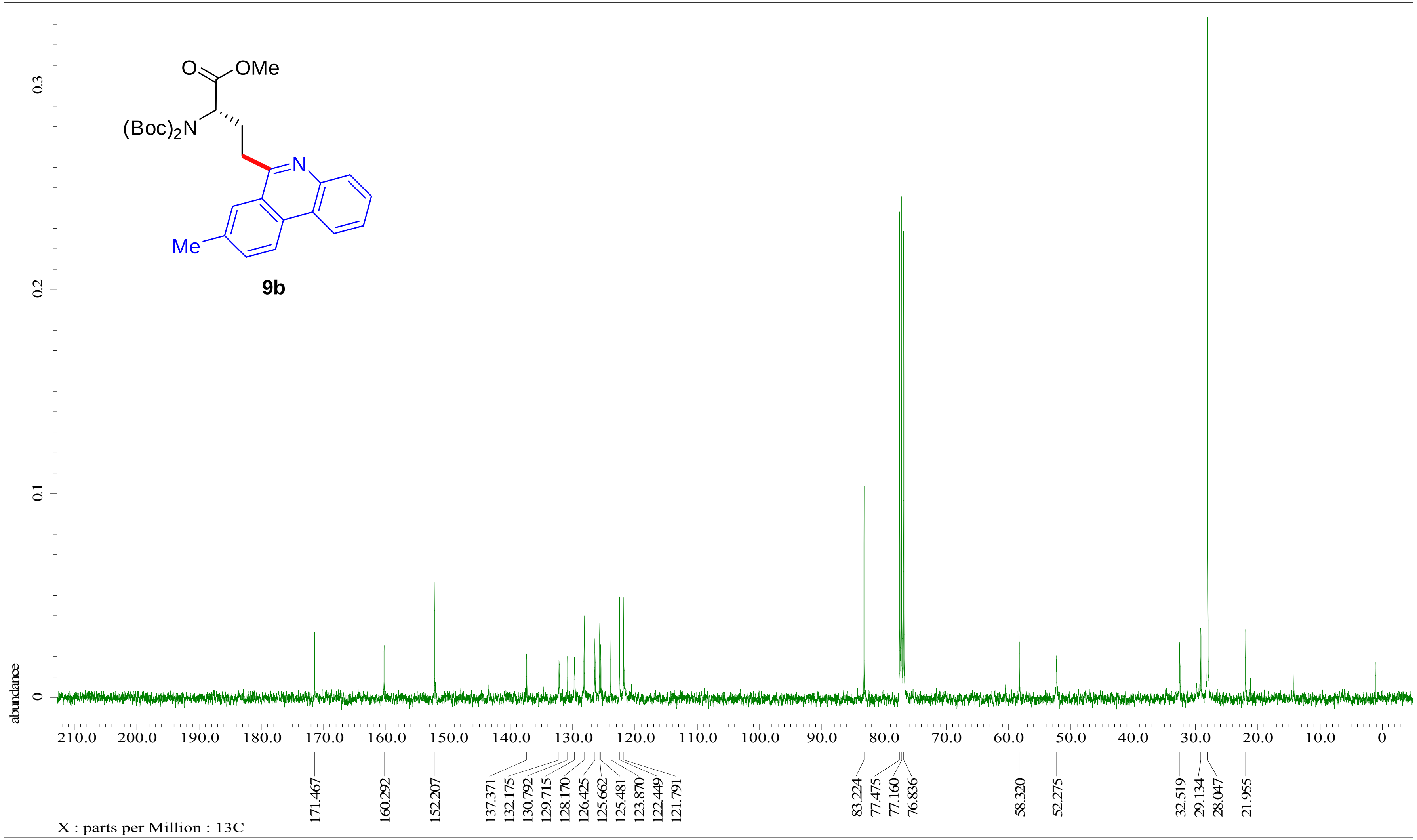
O
OMe
(Boc)2N
N
Me
abundance
0.3
0.2
0.1
0
210.0
200.0
190.0
180.0
170.0
160.0
150.0
140.0
130.0
120.0
110.0
100.0
90.0
80.0
70.0
60.0
50.0
40.0
30.0
20.0
10.0
0
171.467
160.292
152.207
137.371
132.175
130.792
129.715
128.170
126.425
125.662
125.481
123.870
122.449
121.791
83.224
77.475
77.160
76.836
58.320
52.275
32.519
29.134
28.047
21.955
X : parts per Million : 13C

9b

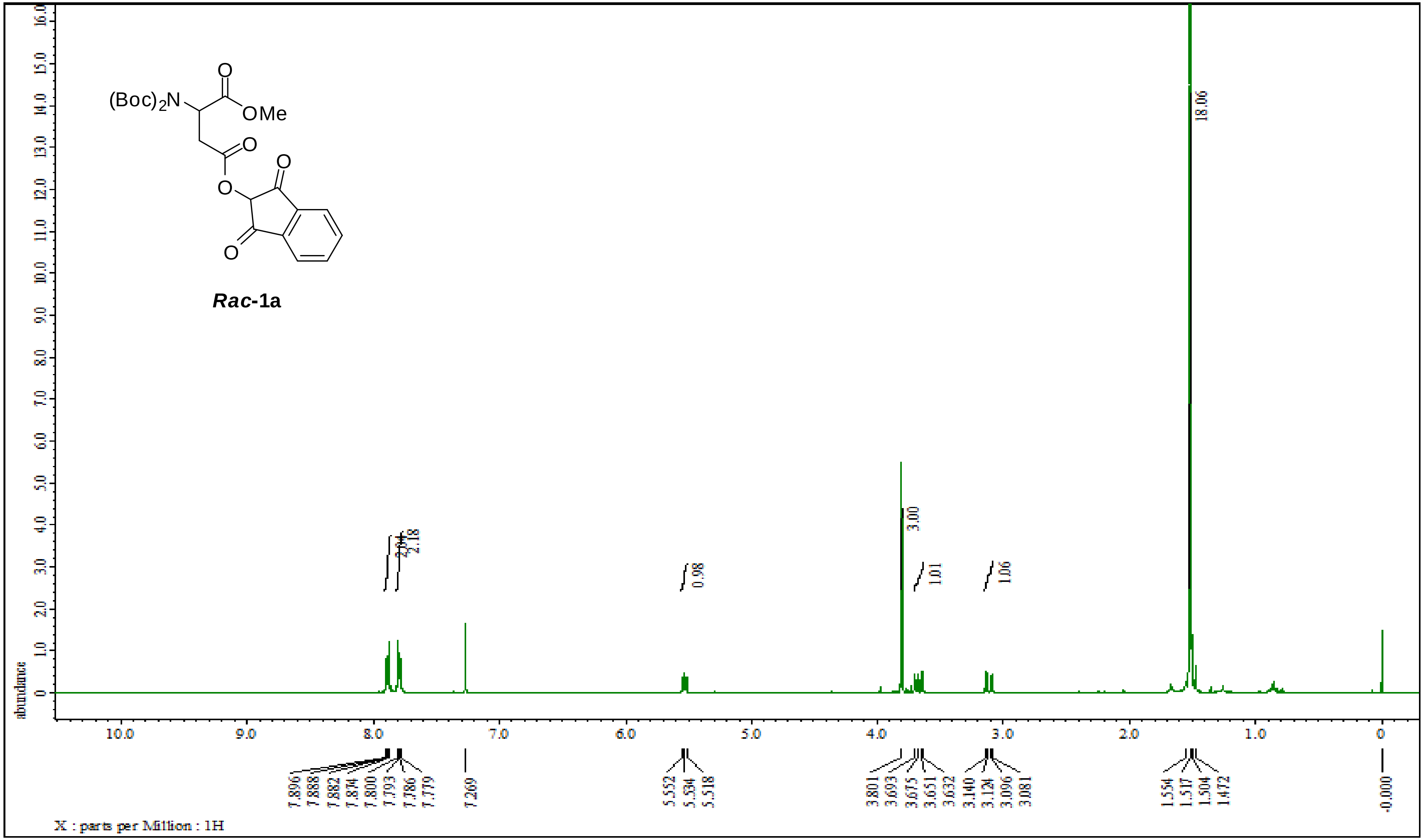
(Boc)2N
OMe
O
Rac-1a
18.06
3.00
2.04
2.18
0.98
1.01
1.06
abundance
X : parts per Million : 1H
7.896
7.888
7.882
7.874
7.800
7.793
7.786
7.779
7.269
5.552
5.534
5.518
3.801
3.693
3.675
3.651
3.632
3.140
3.124
3.096
3.081
1.554
1.517
1.504
1.472
-0.000

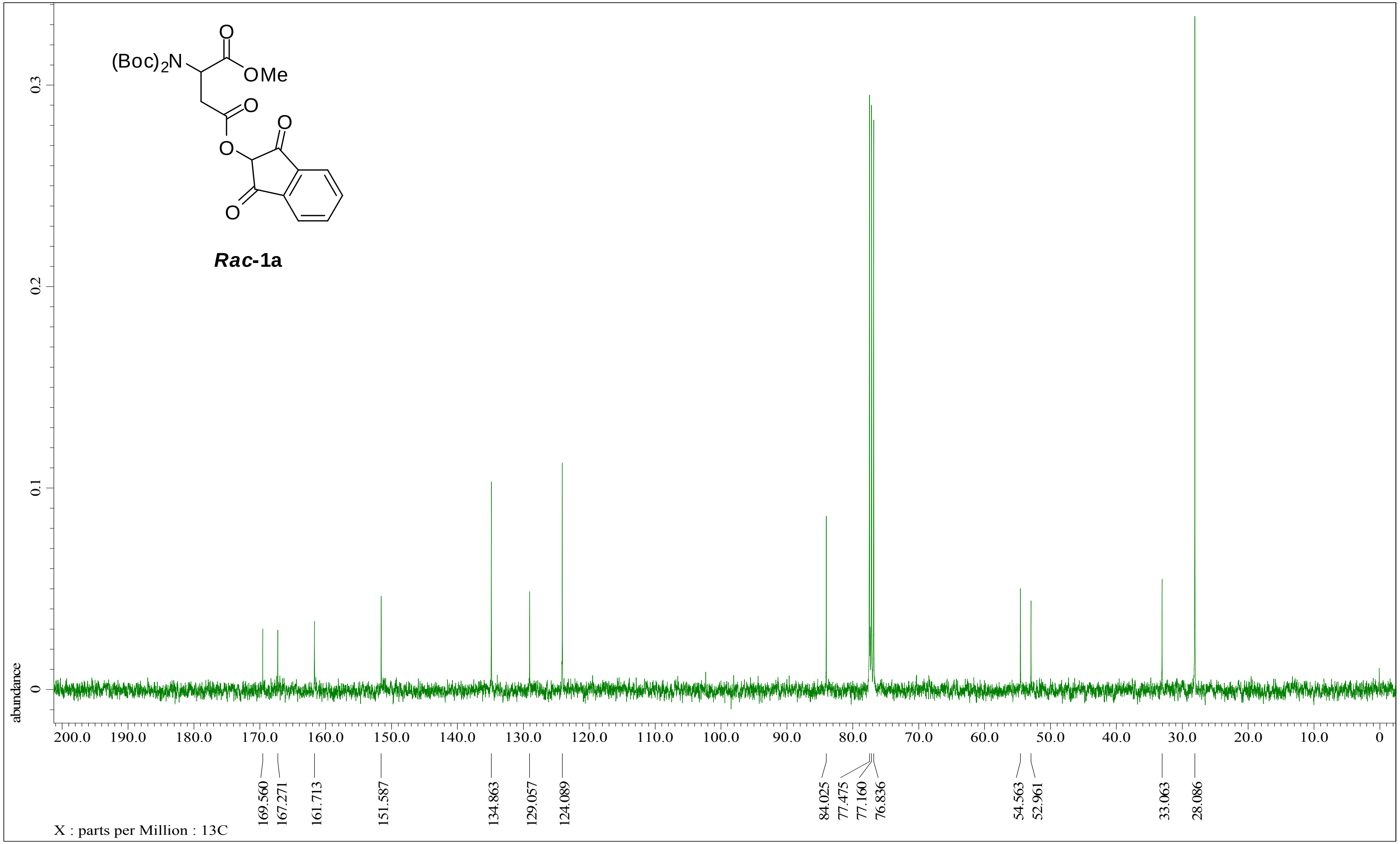

(Boc)2N
O
OMe
O
O
O
O
O
Rac-1a
abundance
0.3
0.2
0.1
0
200.0 190.0 180.0 170.0 160.0 150.0 140.0 130.0 120.0 110.0 100.0 90.0 80.0 70.0 60.0 50.0 40.0 30.0 20.0 10.0 0
169.560
167.271
161.713
151.587
134.863
129.057
124.089
84.025
77.475
77.160
76.836
54.563
52.961
33.063
28.086
X : parts per Million : 13C

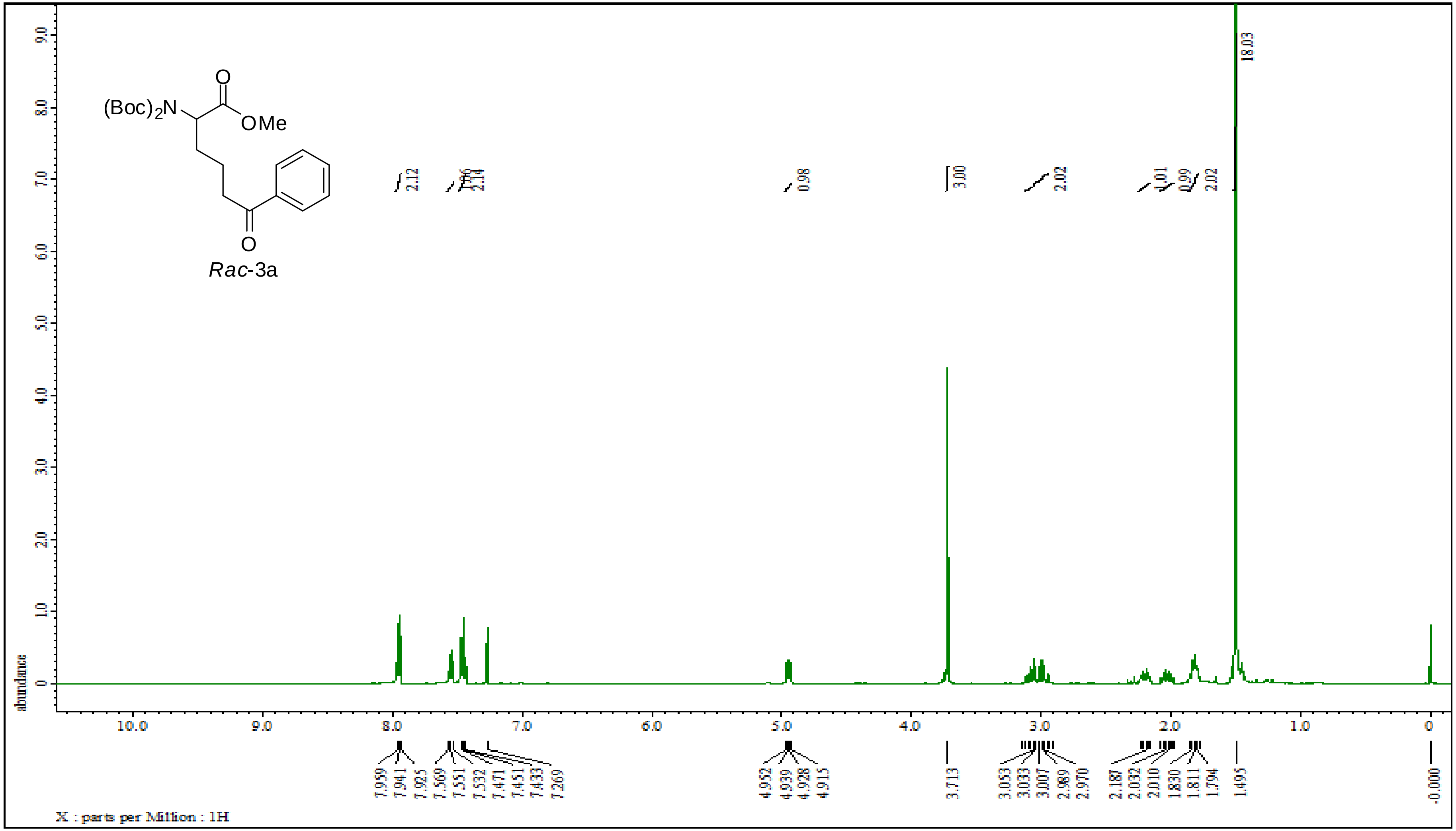
(Boc)2N
O
OMe
O
Rac-3a
abundance
X : parts per Million : 1H
2.12
0.98
3.00
2.02
1.01
0.99
2.02
18.03
10.0
9.0
8.0
7.0
6.0
5.0
4.0
3.0
2.0
1.0
0
7.959
7.941
7.925
7.569
7.551
7.532
7.471
7.451
7.433
7.269
4.952
4.939
4.928
4.915
3.713
3.053
3.033
3.007
2.989
2.970
2.187
2.032
2.010
1.830
1.811
1.794
1.495
-0.000

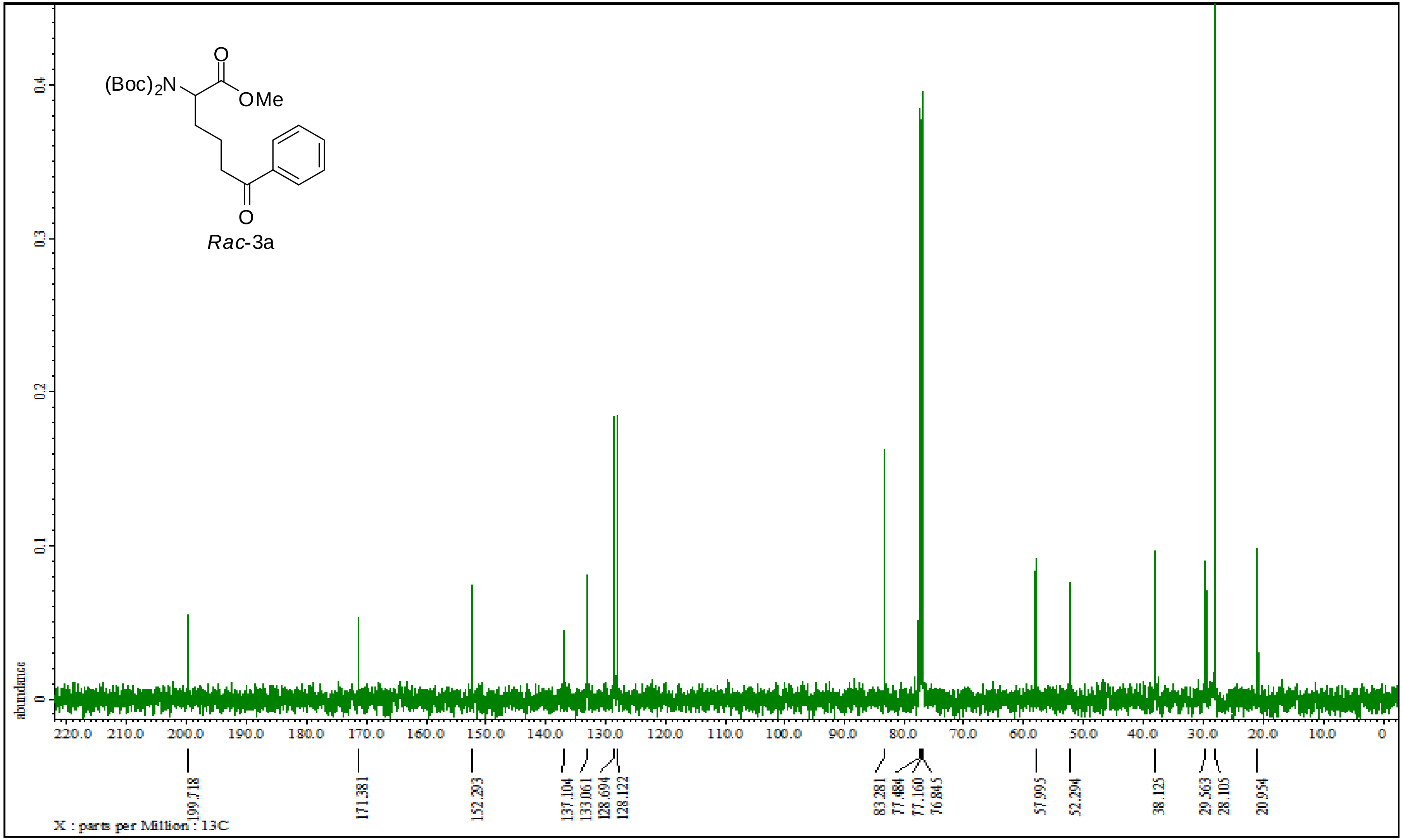
(Boc)2N
O
OMe
O
Rac-3a
abundance
0.4
0.3
0.2
0.1
0
220.0
210.0
200.0
190.0
180.0
170.0
160.0
150.0
140.0
130.0
120.0
110.0
100.0
90.0
80.0
70.0
60.0
50.0
40.0
30.0
20.0
10.0
0
199.718
171.381
152.293
137.104
133.061
128.694
128.122
83.281
77.484
77.160
76.845
57.995
52.294
38.125
29.563
28.105
20.954
X : parts per Million : 13C

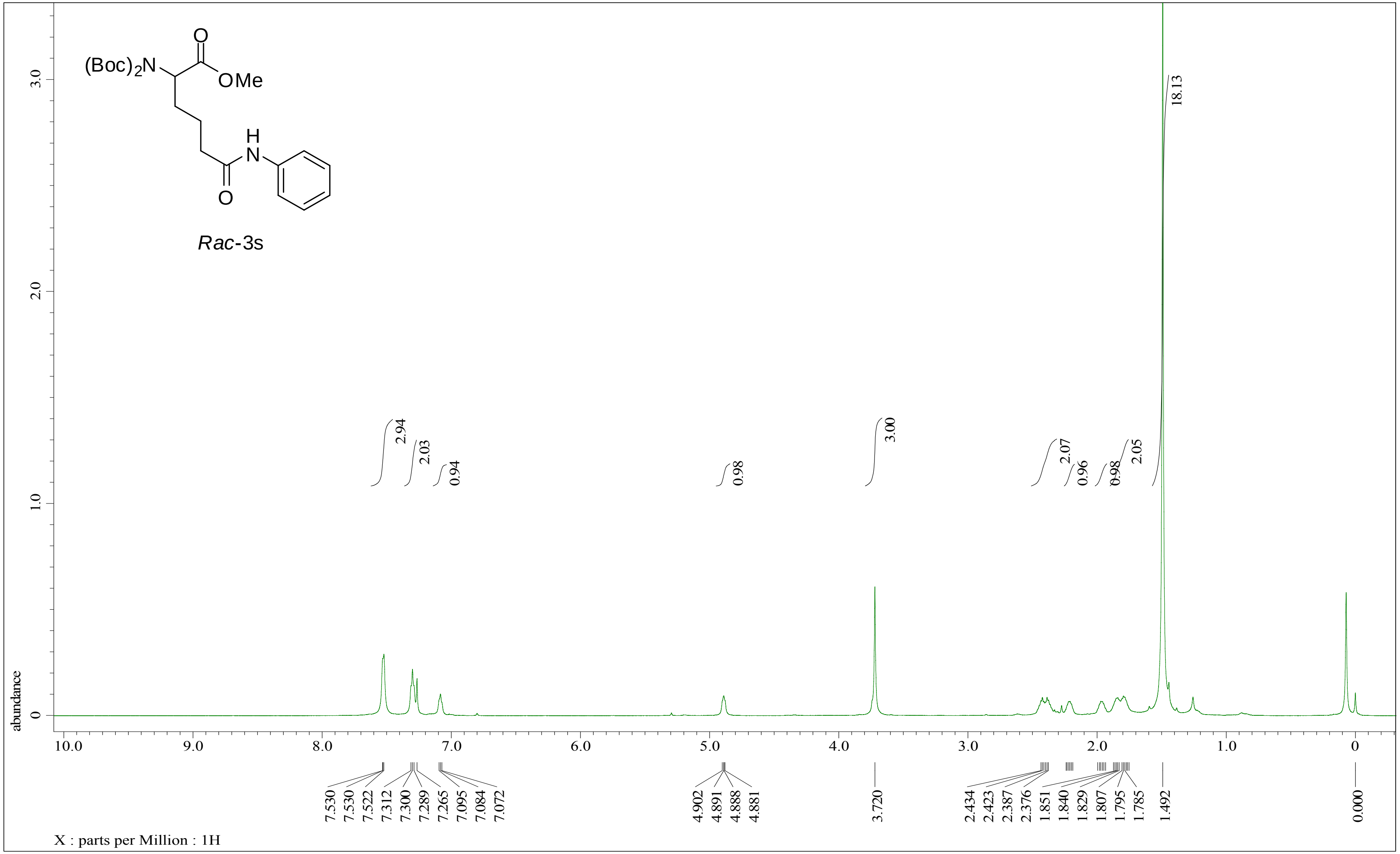
(Boc)2N
O
OMe
H
N
O
Rac-3s
3.0
2.0
1.0
0
abundance
2.94
2.03
0.94
0.98
3.00
2.07
0.96
0.98
2.05
18.13
10.0
9.0
8.0
7.0
6.0
5.0
4.0
3.0
2.0
1.0
0
7.530
7.530
7.522
7.312
7.300
7.289
7.265
7.095
7.084
7.072
4.902
4.891
4.888
4.881
3.720
2.434
2.423
2.387
2.376
1.851
1.840
1.829
1.807
1.795
1.785
1.492
0.000
X : parts per Million : 1H

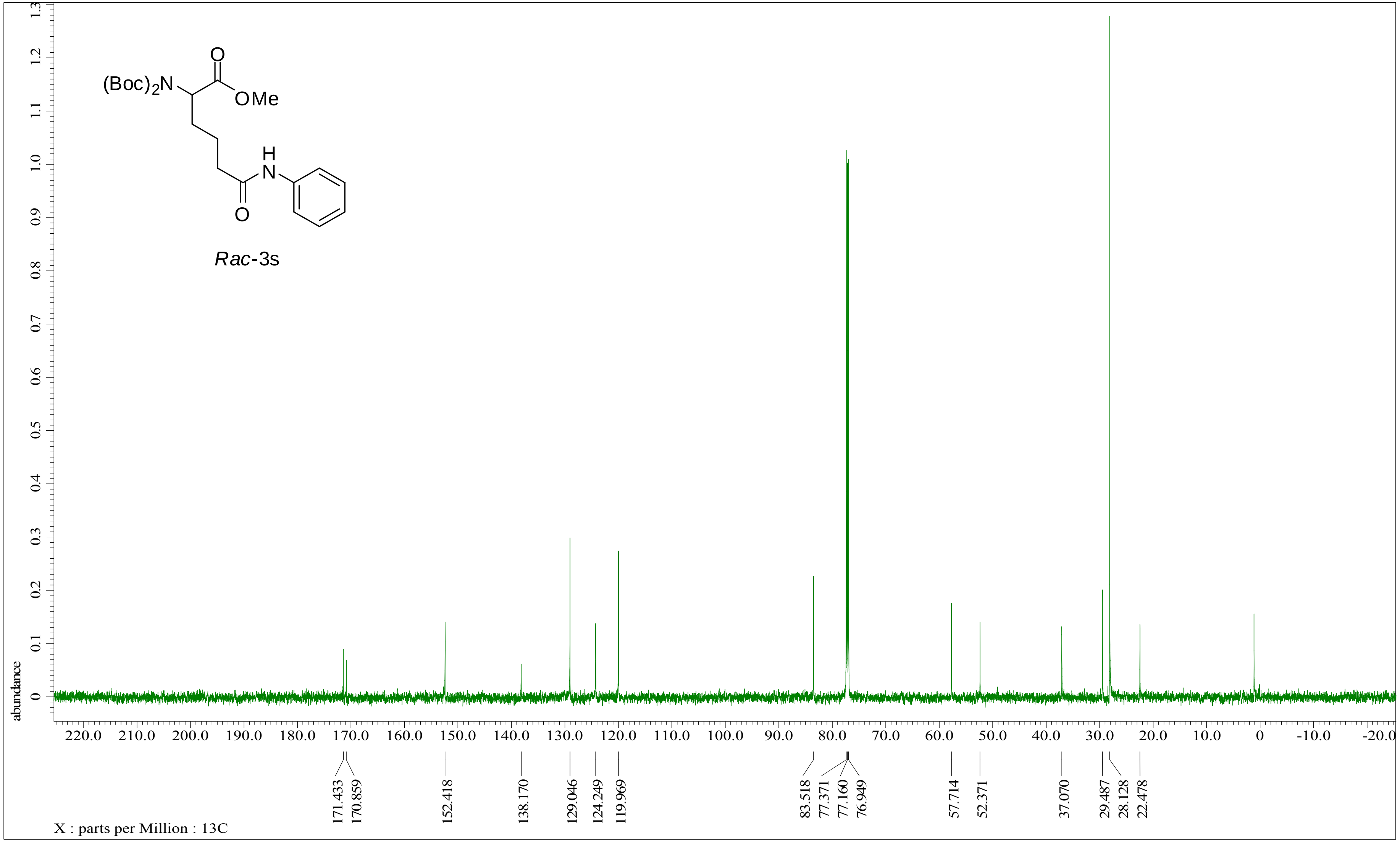

(Boc)2N
O
OMe
H
N
O
Rac-3s
abundance
X : parts per Million : 13C
171.433
170.859
152.418
138.170
129.046
124.249
119.969
83.518
77.371
77.160
76.949
57.714
52.371
37.070
29.487
28.128
22.478

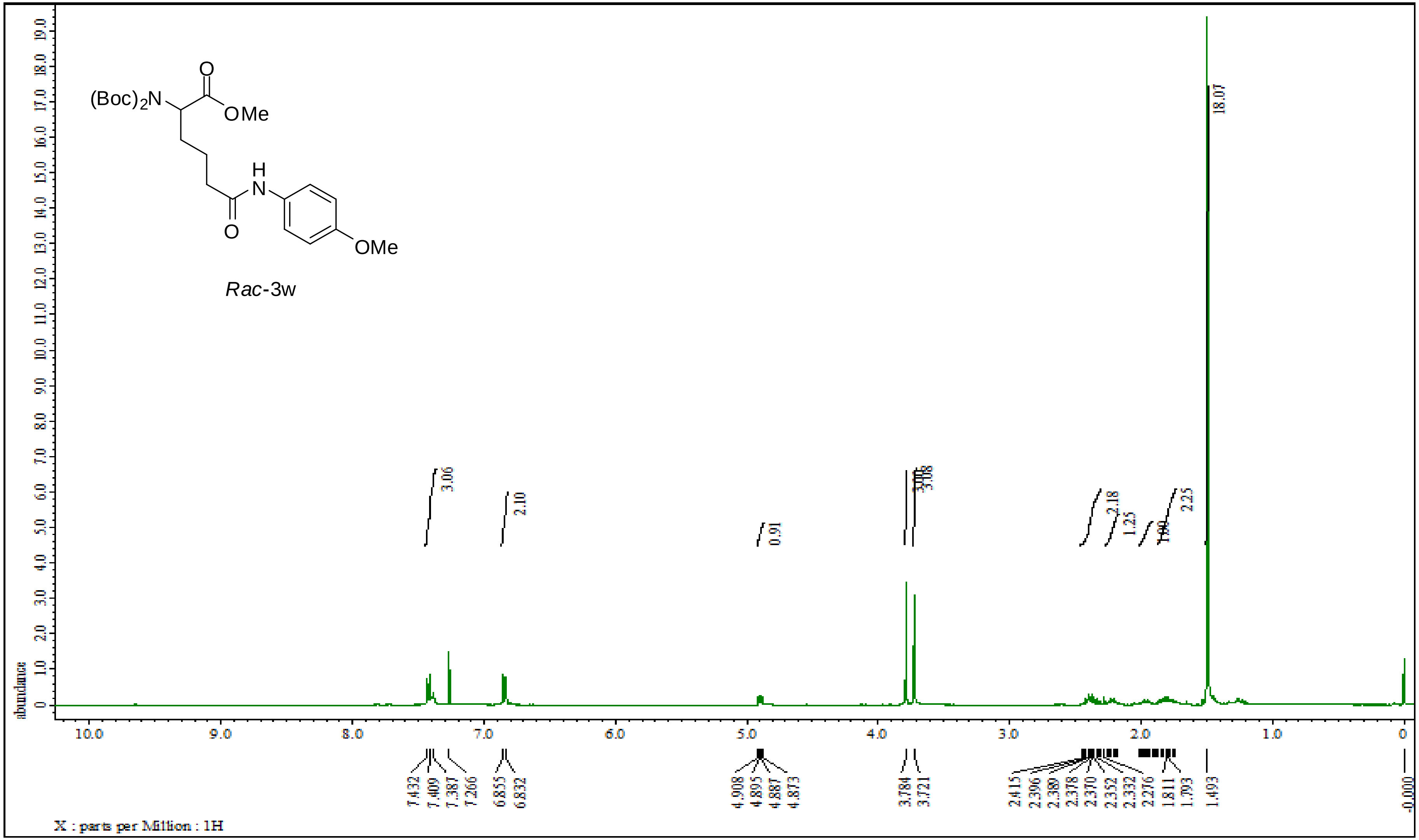
(Boc)2N
OMe
H
N
O
O
OMe
Rac-3w
18.07
3.06
2.10
0.91
2.18
1.25
2.25
7.432
7.409
7.387
7.266
6.855
6.832
4.908
4.895
4.887
4.873
3.784
3.721
2.415
2.396
2.389
2.378
2.370
2.352
2.332
2.276
1.811
1.793
1.493
-0.000
X : parts per Million : 1H
abundance

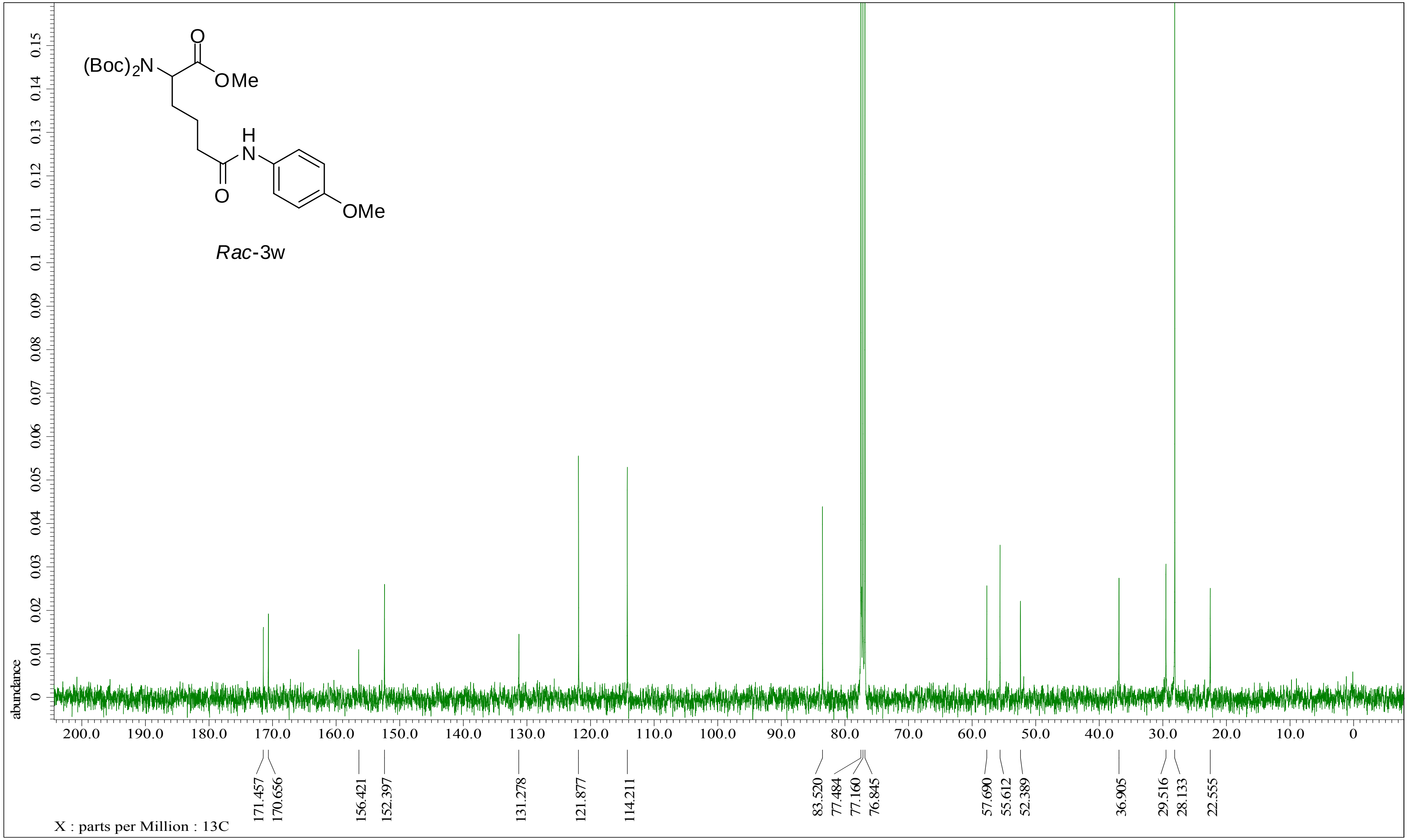

(Boc)2N
O
OMe
H
N
O
OMe
Rac-3w
abundance
X : parts per Million : 13C
171.457
170.656
156.421
152.397
131.278
121.877
114.211
83.520
77.484
77.160
76.845
57.690
55.612
52.389
36.905
29.516
28.133
22.555
